# Nickel-catalyzed coupling reaction of alkyl halides with aryl Grignard reagents in the presence of 1,3-butadiene: mechanistic studies of four-component coupling and competing cross-coupling reactions†
†Electronic supplementary information (ESI) available: Detailed experimental and computational results, procedures, characterization data, copies of NMR charts, and crystallographic data. CCDC 1572238. For ESI and crystallographic data in CIF or other electronic format see DOI: 10.1039/c7sc04675h


**DOI:** 10.1039/c7sc04675h

**Published:** 2018-01-05

**Authors:** Takanori Iwasaki, Asuka Fukuoka, Wataru Yokoyama, Xin Min, Ichiro Hisaki, Tao Yang, Masahiro Ehara, Hitoshi Kuniyasu, Nobuaki Kambe

**Affiliations:** a Department of Applied Chemistry , Graduate School of Engineering , Osaka University , Suita , Osaka 565-0871 , Japan . Email: iwasaki@chem.eng.osaka-u.ac.jp ; Email: kambe@chem.eng.osaka-u.ac.jp; b Department of Material and Life Science , Graduate School of Engineering , Osaka University , Suita , Osaka 565-0871 , Japan; c Department of Theoretical and Computational Molecular Science , Institute for Molecular Science , 38 Nishigo-Naka, Myodaiji , Okazaki , Aichi 444-8585 , Japan . Email: ehara@ims.ac.jp; d Elements Strategy Initiative for Catalysts and Batteries (ESICB) , Kyoto University , Katsura , Kyoto 615-8510 , Japan; e Fachbereich Chemie , Philipps-Universität Marburg , Hans-Meerwein-Strasse, Marburg 35032 , Germany . Email: yangt@staff.uni-marburg.de

## Abstract

We describe the mechanism, substituent effects, and origins of the selectivity of the nickel-catalyzed four-component coupling reactions of alkyl fluorides, aryl Grignard reagents, and two molecules of 1,3-butadiene that affords a 1,6-octadiene carbon framework bearing alkyl and aryl groups at the 3- and 8-positions, respectively, and the competing cross-coupling reaction. Both the four-component coupling reaction and the cross-coupling reaction are triggered by the formation of anionic nickel complexes, which are generated by the oxidative dimerization of two molecules of 1,3-butadiene on Ni(0) and the subsequent complexation with the aryl Grignard reagents. The C–C bond formation of the alkyl fluorides with the γ-carbon of the anionic nickel complexes leads to the four-component coupling product, whereas the cross-coupling product is yielded *via* nucleophilic attack of the Ni center toward the alkyl fluorides. These steps are found to be the rate-determining and selectivity-determining steps of the whole catalytic cycle, in which the C–F bond of the alkyl fluorides is activated by the Mg cation rather than a Li or Zn cation. *ortho*-Substituents of the aryl Grignard reagents suppressed the cross-coupling reaction leading to the selective formation of the four-component products. Such steric effects of the *ortho*-substituents were clearly demonstrated by crystal structure characterizations of ate complexes and DFT calculations. The electronic effects of the *para*-substituent of the aryl Grignard reagents on both the selectivity and reaction rates are thoroughly discussed. The present mechanistic study offers new insight into anionic complexes, which are proposed as the key intermediates in catalytic transformations even though detailed mechanisms are not established in many cases, and demonstrates their synthetic utility as promising intermediates for C–C bond forming reactions, providing useful information for developing efficient and straightforward multicomponent reactions.

## Abstract 2

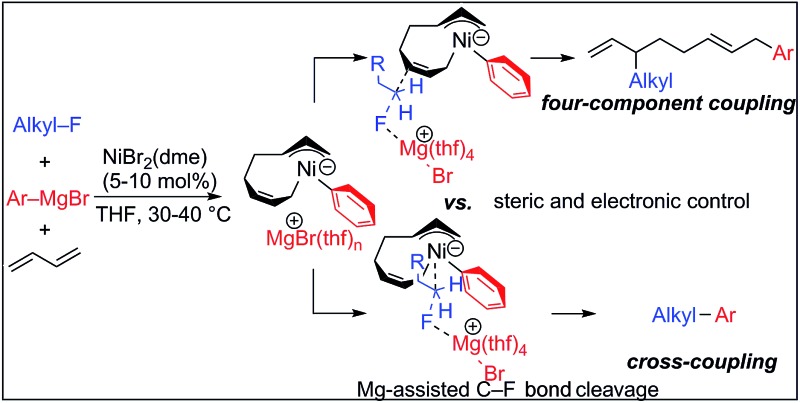
The detailed reaction mechanism of anionic Ni complex-promoted C–C bond forming reactions was clarified by experimental and theoretical methods.

## 1 Introduction

Metal-catalyzed multicomponent coupling reactions are useful and straightforward synthetic methods, because they can construct complex carbon frameworks from relatively simple organic molecules through the concomitant formation of multiple new bonds.^1^ In these transformations, the oxidative dimerization of 1,3-butadiene on low valent group 10 metals is a fundamental process, being often used to produce a variety of C8 carbon frameworks.^2^ A well-known application is telomerization,^3^ in which bis(π-allyl)metal complexes of group 10 metals (Ni and Pd) generated by the oxidative dimerization of two molecules of 1,3-butadiene on the metals react with heteroatom nucleophiles such as water, alcohols, and amines. This process has been used for the industrial production of C8 chemicals including 1-octanol and 1-octene.^4^ In this context, pioneering studies of the Ni-catalyzed multicomponent reaction of two molecules of 1,3-butadiene with carbonyl compounds5a,b and imines5b–d as well as CO and CO_2_ (ref. 6) were reported in the 1970s. Kimura and Tamaru have expanded these multicomponent coupling reactions of 1,3-butadiene to the bifunctionalization of the C8 carbon framework through the combined use of ketones, aldehydes, or imines with organozinc or organoaluminium reagents.^7^,^8^ This reaction could introduce two different carbon moieties into the 2,6-octadiene framework. However, in some cases, three component coupling of these reagents in a 1 : 1 : 1 ratio without dimerization can occur as a competing or preferred reaction7a,c,^9^ due to the competing oxidative cyclization of 1,3-butadiene with carbonyl compounds to form oxanickelacycle intermediates.^10^

**Scheme 1:**
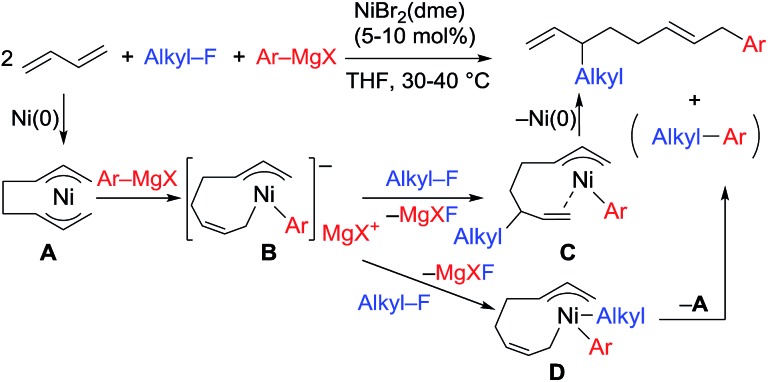
Ni-catalyzed dimerization and alkylarylation of 1,3-dienes.

We have reported the dimerization and silylation of 1,3-butadiene as another application of these transformations.^11^ In addition, aiming at the construction of carbon skeletons, we recently developed the multicomponent coupling reaction of two molecules of 1,3-butadiene, aryl Grignard reagents, and alkyl fluorides to give a 1,6-octadiene bearing an alkyl group arising from the alkyl fluorides at the 3-position, and an aryl group arising from the aryl Grignard reagents at the 8-position (Scheme 1).^12^ It was also revealed that a similar multicomponent coupling reaction using perfluoroarenes instead of alkyl fluorides proceeds smoothly to yield the corresponding 1,6-octadienes with perfluoroaryl and aryl groups.^13^ In these reactions, three-component coupling products consisting of organo fluorides, aryl Grignard reagents, and 1,3-butadiene in a 1 : 1 : 1 ratio were not produced due to the intermediacy of anionic bis(allyl)nickel intermediates. In the alkylation reaction mentioned above, the use of *ortho*-substituted aryl Grignard reagents is essential for selective formation of four-component coupling products. When unsubstituted aryl Grignard reagents were employed instead, the competing reaction of direct cross-coupling of alkyl fluorides with aryl Grignard reagents occurred, resulting in a mixture of cross-coupling and four-component coupling products (Scheme 1).^14^ In these reactions as well as our previously reported cross-coupling reactions,^15^ an anionic nickel complex **B** generated by the reaction of the bis(π-allyl)nickel complex **A** with the Grignard reagent is likely the key intermediate, which reacts with electrophiles at the Ni center to promote the cross-coupling reaction (*via* complex **D**) or at the γ-carbon of the σ-allyl giving rise to four-component coupling products (*via* complex **C**).

Herein, to understand the detailed mechanism of the four-component coupling reaction of alkyl fluorides, aryl Grignard reagents, and two molecules of 1,3-butadiene, we performed mechanistic studies and theoretical calculations to clarify the structures and chemical behavior of the anionic nickel intermediate **B**. In addition, we performed the reaction using various aryl Grignard reagents including *ortho*-unsubstituted ones to reveal not only the substrate scope of the reaction but also the origin of the selectivity of the four-component coupling reaction over the direct cross-coupling reaction. Steric and electronic effects on the selectivity were also investigated by theoretical calculations.

Notably, the structures and chemical characteristics of anionic complexes of transition metals (the so-called ate complexes^16^) have rarely been studied, except for the case of cuprates,^17^ even though ate complexes have been proposed as key intermediates in catalytic C–C bond formations.^18^–^21^ In addition, theoretical calculations of ate complexes have not been well-established because of a lack of structural information of ate complexes (especially the counter cation). Therefore, another challenge in this study is to develop a means for theoretical calculations of ate complex-mediated transformations.

## 2 Results and discussion

### 2.1. Reaction using aryl Grignard reagents

We first examined the reaction using *n*-OctF (**1a**) and *p*-fluorophenylmagnesium bromide (**2a**), and the representative results are summarized in Table 1. When the reaction was conducted in the presence of NiBr_2_(dme) (20 mol%) and 1,3-butadiene (3 equiv.) at 40 °C for 24 h, the cross-coupling product **4aa** was obtained in 33% yield along with **3aa** incorporating two 1,3-butadiene molecules in 50% yield (entry 1). The structure of compound **3aa** was fully assigned by NMR. The alkyl and aryl groups were introduced site-selectively onto the 3- and 8-positions of the 1,6-octadiene framework, respectively, with an *E*-configuration of the internal alkene moiety. The amount of Ni catalyst could be reduced to 10 mol% (entry 2); however, a further reduction to 5 mol% led to incomplete reaction within 24 h (79% conversion of **1a**). We found that the selectivity of the four-component coupling *versus* cross-coupling reactions is temperature-dependent. When the reaction temperature was increased to 50 °C, the selectivity decreased to almost 1 : 1 (entry 3). In contrast, a lower reaction temperature increased the selectivity to 1.7 : 1 (entry 4), although the reaction was sluggish at 0 °C, and 41% of **1a** remained after 24 h (entry 5).

**Table 1:** Coupling reactions of *n*-OctX, *p*-FC_6_H_4_MgBr, and 1,3-butadiene^*a*^

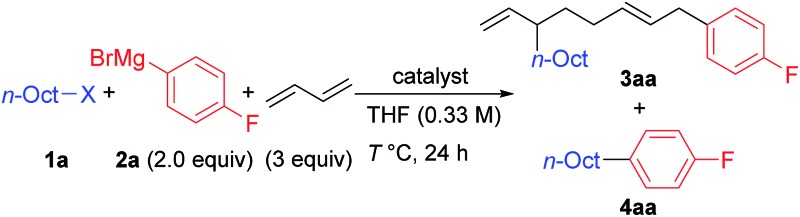					
Entry	Catalyst (mol%)	X	*T* (°C)	**3aa** (%)^*b*^	**4aa** (%)^*b*^
1	NiBr_2_(dme) (20)	F	40	50	33
2	NiBr_2_(dme) (10)	F	40	48	36
3	NiBr_2_(dme) (10)	F	50	49	49
4	NiBr_2_(dme) (10)	F	30	63 (48)	37 (37)
5	NiBr_2_(dme) (10)	F	0	31	19
6	NiBr_2_(dme) (10)	Cl	30	2	10
7	NiBr_2_(dme) (10)	Br	30	15	52
8	NiBr_2_(dme) (10)	I	30	6	58
9	NiBr_2_(dme) (10)	OTs	30	17	68
10	None	F	25	n.d.	n.d.
11	Pd(acac)_2_ (20)	F	25	n.d.	52
12	PtCl_2_ (20)	F	25	n.d.	3

^*a*^Reaction conditions: catalyst, *n*-OctF (1.0 mmol), *p*-F-C_6_H_4_MgBr (2.0 mmol), and 1,3-butadiene (3.0 mmol) in THF (3 mL) for 24 h.

^*b*^Yields were determined by GC. Isolated yields are in parentheses.

Next, we investigated the effect of the leaving group (entries 6–9). The reaction of *n*-OctCl was sluggish (entry 6). Alkyl bromides, iodides, and tosylates (which are good coupling partners in alkyl–alkyl cross-coupling reactions)^15^,20a–c gave **4aa** as the major product in 52 to 68% yields along with small amounts of **3aa** (entries 7–9), where the product ratios **3aa**/**4aa** were 0.20 : 1 (Cl), 0.29 : 1 (Br), 0.10 : 1 (I), and 0.25 : 1 (OTs). These results imply the usefulness of alkyl fluorides as promising alkylating reagents toward 1,3-butadiene.^22^,^23^ Neither four-component coupling nor cross-coupling reactions could proceed without the Ni catalyst (entry 10). It is known that other group 10 metals also promote the oxidative dimerization of 1,3-dienes.^3^,20a However, no desired four-component coupling product **3aa** was yielded by Pd and Pt catalysts, while the cross-coupling product **4aa** was obtained in 52% and 3% yields, respectively (entries 11 and 12).

In order to improve the selectivity of **3aa** over **4aa**, we tested the reaction using various ligands, additives, and solvents. However, these attempts were not fruitful. For instance, the addition of PPh_3_ slightly affected the reaction efficiency without appreciable change in the selectivity. The addition of Mg and Li salts, on the other hand, decreased the selectivity to 1 : 1 and 0.6 : 1, respectively. The reactions in mixed solvents of toluene-THF or hexane-THF depressed both the yields and selectivity.^24^

**Scheme 2:**
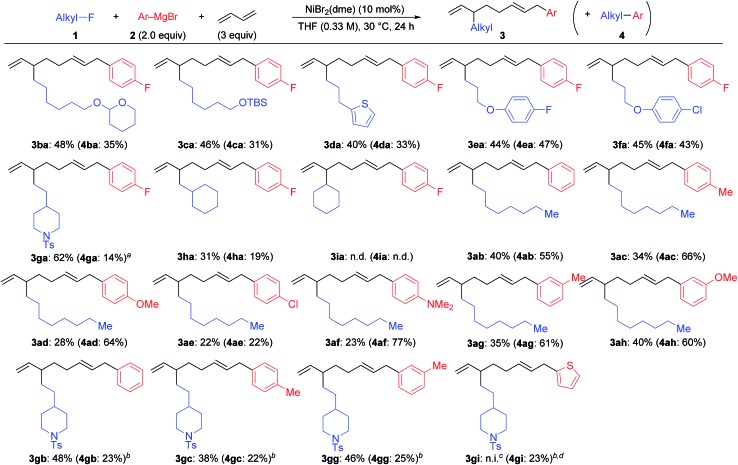
The Ni-catalyzed four-component coupling reaction of alkyl fluorides, aryl Grignard reagents, and 1,3-butadiene. Isolated yields. The yields of cross-coupling products **4** are shown in parentheses. ^*a*^*ca.* 20% of **1g** was recovered. ^*b*^The reaction time was 42 h. ^*c*^n.i. = not isolated. ^*d*^44% of **1g** was recovered.

Scheme 2 summarizes the results of the four-component coupling reaction with various aryl Grignard reagents and alkyl fluorides under the same conditions as entry 4 in Table 1, where the yields of the direct cross-coupling product **4** are given in parentheses. Alkyl fluorides carrying various functional groups produced mixtures of **3** and **4** in a 1.5 : 1 to 1 : 1 ratio. It should be noted that the aromatic C–F and C–Cl bonds remained intact, and the aliphatic C–F bonds were cleaved exclusively. The reaction of γ-branched alkyl fluoride **1g** resulted in better selectivity (**3ga** : **4ga** = 5.2 : 1) although the reaction was slow and did not complete within 24 h (80% conv.). This result may suggest that sterically hindered alkyl fluorides prefer the four-component coupling reaction to give **3** rather than the cross-coupling reaction. However, the reaction of (fluoromethyl)cyclohexane (**1h**) resulted in a low yield, and no reaction took place with fluorocyclohexane (**1i**). Next, we ran the reactions using different aryl Grignard reagents (**2b–i**). PhMgBr (**2b**) afforded the corresponding products **3ab** and **4ab** in 40% and 55% yields, respectively (95% total yield with a 0.7 : 1 ratio). The introduction of electron-donating groups such as Me, MeO, and Me_2_N led to the preferential formation of the cross-coupling product **4**. On the other hand, the *para*-chlorophenyl Grignard reagent (**2e**) gave a 1 : 1 mixture of **3ae** and **4ae** in 44% total yield. Substituents at the *meta*-position showed a similar tendency. The reaction of **1g** with phenyl and *para*- and *meta*-tolyl Grignard reagents afforded four-component coupling products **3gb**, **3gc**, and **3gg** as the major product. The thienyl Grignard reagent **2i** underwent a cross-coupling reaction exclusively to give **4gi** in 23% yield.

### 2.2. Reaction using *ortho*-substituted aryl Grignard reagents

We found a remarkable effect of the *ortho*-substituent in the aryl Grignard reagents, as shown in eqn (1).^12^ When *o*-tolylmagnesium bromide (**2j**) was employed in the reaction with **1g**, the four-component coupling product **3gj** was obtained as the sole product in 85% yield, and no **4gj** was observed.
1

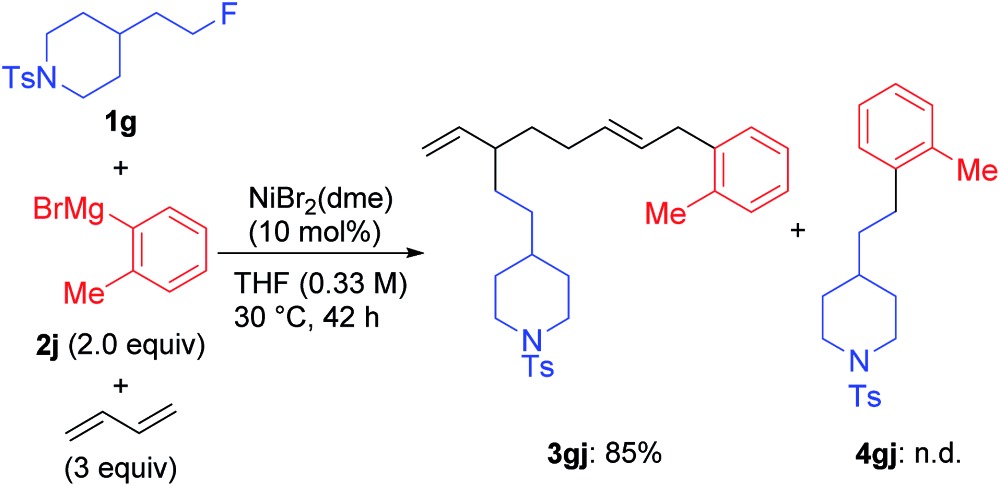




Using **1a** and **2j** under the previously optimized conditions (Table 1, entry 4), the reaction proceeded smoothly to give **3aj** in 92% GC yield within 10 h (Table 2, entry 1). The addition of PPh_3_ or a bidentate phosphine (dppf) hindered the reaction (entries 2 and 3). Other Ni salts such as NiCl_2_ and Ni(acac)_2_ showed a comparable reactivity (entries 4 and 5). The amount of Grignard reagent **2j** could be reduced to 1.5 equiv. without significant loss of yield (entry 6). Although the reaction did not complete within 10 h with 5 mol% of NiBr_2_(dme) (entry 7, 88% conv. of **1a**), the four-component coupling product **3aj** was obtained in 87% yield by elevating the reaction temperature to 40 °C (entry 8).

**Table 2:** The four-component coupling reaction of *n*-Oct–F, *o*-TolMgBr, and 1,3-butadiene

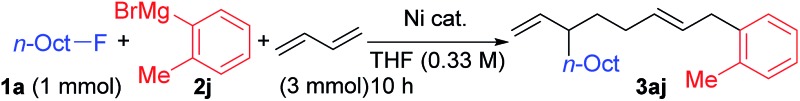				
Entry	Catalyst (mol%)	**2j**	Temp. (°C)	**3aj** (%)^*a*^
1	NiBr_2_(dme) (10)	2.0 equiv.	30	92
2	NiCl_2_(PPh_3_)_2_ (10)	2.0 equiv.	30	43
3	NiCl_2_(dppf) (10)	2.0 equiv.	30	8
4	NiCl_2_ (10)	2.0 equiv.	30	90
5	Ni(acac)_2_ (10)	2.0 equiv.	30	95
6	NiBr_2_(dme) (10)	1.5 equiv.	30	88
7	NiBr_2_(dme) (5)	1.5 equiv.	30	79
8	NiBr_2_(dme) (5)	1.5 equiv.	40	87 (86)

^*a*^Determined by GC (isolated yield is in parentheses). In all cases, *ca.* 5% of **4aj** was observed in the GC analysis.

The results using various alkyl (pseudo)halides are summarized in Table 3. While the C(sp^3^)–Cl bond is weaker than the C(sp^3^)–F bond, *n*-OctCl was less reactive and mostly recovered unreacted (entry 2). Although alkyl bromides and iodides afforded the cross-coupling product predominantly with the *p*-fluorophenyl Grignard reagent **2a**, as shown in Table 1, introducing an *ortho*-substituent into ArMgBr largely suppressed the cross-coupling, giving rise to the selective formation of the four-component coupling product albeit only in moderate yields (entries 3 and 4). Alkyl mesylate was found to be ineffective as an alkylating reagent in the present reaction (entry 5).

**Table 3:** The four-component coupling reaction of alkyl(pseudo)halides

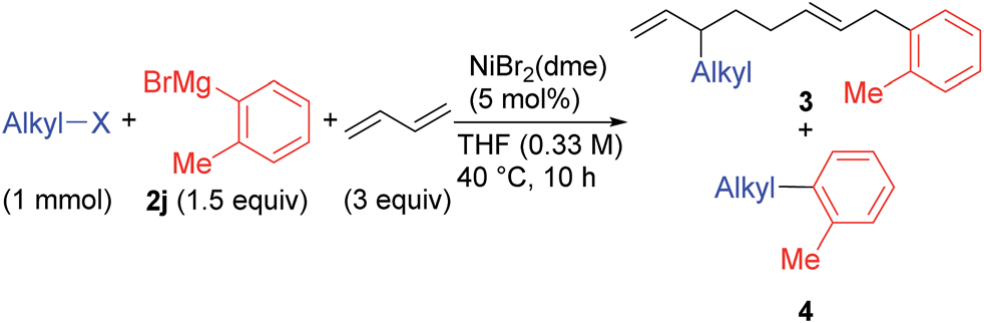				
Entry	Alkyl–X	Conv. (%)^*a*^	**3** (%)^*a*^	**4** (%)^*a*^
1	*n*-Oct–F	98	87	3
2	*n*-Oct–Cl	8	4	2
3	*n*-Non–Br	>99	64	2
4	*n*-Oct–I	>99	45	Trace
5	*n*-Oct–OMs	47	16	18

^*a*^Determined by GC.

**Scheme 3:**
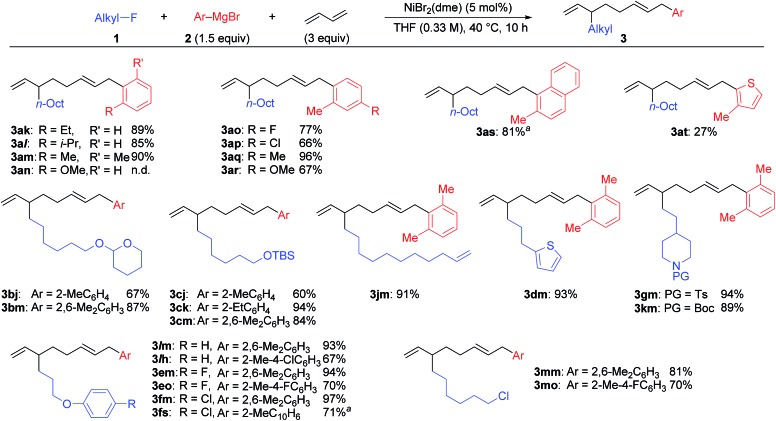
The scope and limitations of the four-component coupling reaction. ^*a*^The reaction was conducted in a 0.1 M concentration of **1** for 20 h.

Under the optimized conditions shown in entry 8 of Table 2, the scope and limitations of the aryl Grignard reagents and alkyl fluorides were further examined, and the results are summarized in Scheme 3.^12^ Sterically hindered aryl Grignard reagents such as 2-ethyl-, 2-isopropyl-, and 2,6-dimethylphenyl Grignard reagents (**2k–m**) underwent the four-component coupling reaction to produce **3ak–am** in good yields. On the other hand, the introduction of a methoxy group at the *ortho*-position (**2n**) completely suppressed the reaction (**3an**). When *o*-tolyl Grignard reagents bearing electron-withdrawing groups at the *para*-position (**2o** and **2p**) were employed, the yields were slightly reduced (**3ao** and **3ap**). The reaction of the 2,4-dimethylphenyl Grignard reagent (**2q**) resulted in an excellent yield (**3aq**) although the *p*-methoxy substituent led to a somewhat lower yield (**3ar**). The thienyl Grignard reagent (**2t**) was less effective, and the naphthyl Grignard reagent (**2s**) afforded **3as** in 81% yield.

Alkyl fluorides (**2b–m**) carrying various functionalities including acetal, silyl ether, *N*-tosyl, and *N*-Boc protecting groups afforded the four-component coupling products in good yields. A terminal olefin moiety in **1j** remained intact to give **3jm** in 91% yield under the selective oxidative dimerization conditions of 1,3-dienes. The alkyl fluoride **1d** bearing an acidic hydrogen at the α-position of thiophene also gave **3dm** in an excellent yield (93%). It is well known that low-valent Ni intermediates can cleave C(sp^2^)–X bonds including the C–F bond.^22^ However, under the reaction conditions, both the C(sp^2^)–Cl and C(sp^2^)–F bonds remained unchanged, and the C(sp^3^)–F bond was selectively cleaved to give **3em**, **3eo**, **3fm**, and **3fs**. In addition, 1-chloro-6-fluorohexane (**1m**) reacted at the C(sp^3^)–F bond rather than the C(sp^3^)–Cl bond to exclusively give **3mm** and **3mo**.

When isoprene was employed as the conjugated diene under the optimized conditions, the corresponding four-component coupling product **3aj′** was obtained as a mixture of four inseparable regioisomers in a 1 : 1.3 : 1.4 : 1.5 ratio in 76% total yield (eqn (2)). In contrast, the reaction of 1,3-pentadiene was sluggish, and no desired four-component coupling products were observed.
2

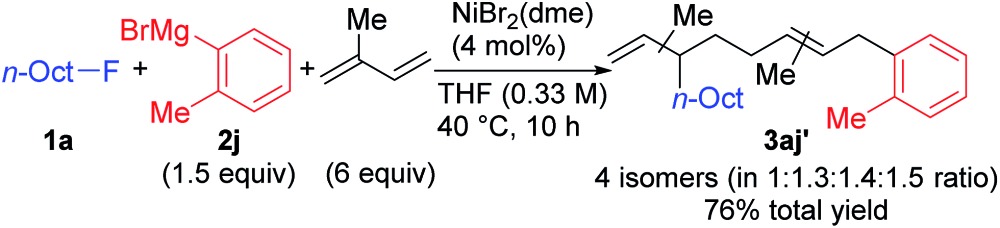




### 2.3. Mechanistic studies

**Scheme 4:**
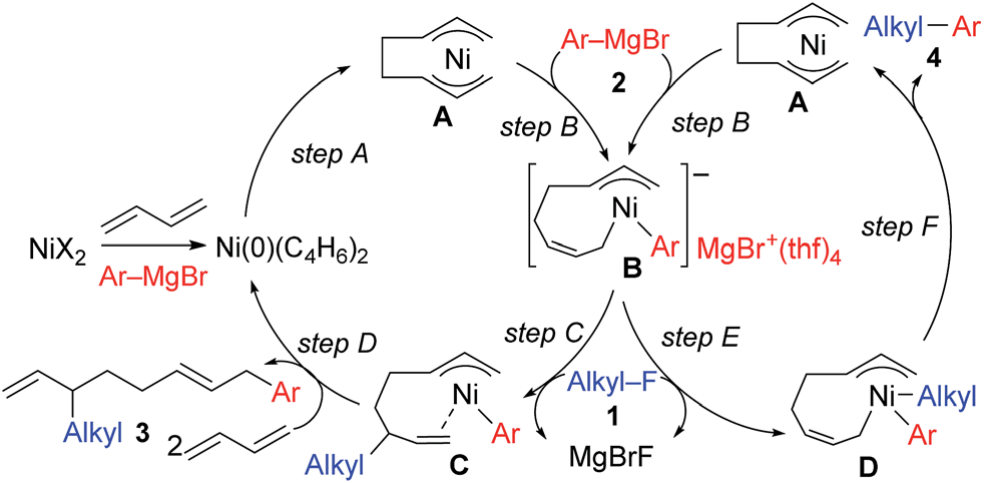
Proposed catalytic cycles of the Ni-catalyzed four-component coupling and cross-coupling reactions.

The mechanistic studies include the observation and isolation of catalytic intermediates, kinetic parameters, electronic effects of the Grignard reagents, and theoretical calculations. Based on the results, plausible catalytic cycles of the Ni-catalyzed coupling reactions of alkyl fluorides with aryl Grignard reagents in the presence of 1,3-butadiene are proposed (Scheme 4). These reactions are triggered by the oxidative dimerization of 1,3-butadiene on Ni(0), generated *in situ* by the reduction of Ni salt with Grignard reagents, to form the bis(π-allyl)nickel **A** (step A). The reaction of **A** with aryl Grignard reagents yields the anionic Ni complex **B**, which has higher nucleophilicity towards alkyl fluorides. Although the four potent nucleophilic centers exist in complex **B**, namely the Ni center, the *ipso* carbon of the Ar group, and the α- and γ-positions of the σ-allyl group, two reaction pathways operate. When the γ-carbon of the σ-allyl group attacks the alkyl fluoride (step C), the four-component coupling product **3** is yielded through the reductive elimination of the thus-formed complex **C** (step D). Alternatively, the Ni center in complex **B** can react with alkyl fluorides to give the cross-coupling product **4** through complex **D** (steps E and F).^25^ Therefore, the reaction courses of four-component coupling *vs.* cross-coupling are determined by the reaction of alkyl fluorides with complex **B** (step C *vs.* step E).

#### 2.3.1. Stoichiometric reaction

To gain insight into the present reaction, we conducted a stoichiometric reaction using NiBr_2_(dme), **1a**, and 2–4 equiv. of **2j** in the presence of excess 1,3-butadiene (Table 4).^12^ However, when 2 equiv. of **2j** was added to reduce Ni(ii) to Ni(0), which then undergoes the oxidative dimerization of 1,3-butadiene to form **A** (step A in Scheme 4), only 14% of **1a** reacted, suggesting that bis(π-allyl)nickel is inert toward alkyl fluorides (entry 1). When 3 equiv. of **2j** was used, 80% of **1a** was consumed to give **3aj** in 75% yield (entry 2). Almost a quantitative yield of **3aj** was obtained with 4 equiv. of **2j** (entry 3). These results indicate that the anionic nickel complex **B** (Scheme 4), generated by the reaction of bis(π-allyl)nickel **A** with Grignard reagents, is the actual intermediate of the reaction of alkyl fluorides.

**Table 4:** Stoichiometric reactions of Ni(ii) with **1a** and **2j**

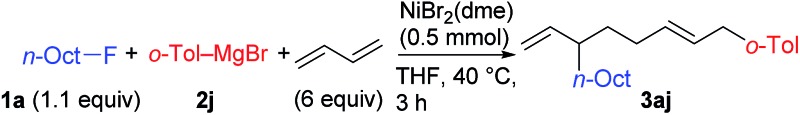			
Entry	*o*-TolMgBr	Conv. **1a** (%)^*a*^	**3aj** (%)^*a*^
1	2 equiv.	14	10
2	3 equiv.	80	75
3	4 equiv.	99	95

^*a*^Determined by GC.

#### 2.3.2. Synthesis and characterization of nickelate complexes

**Fig. 1:**
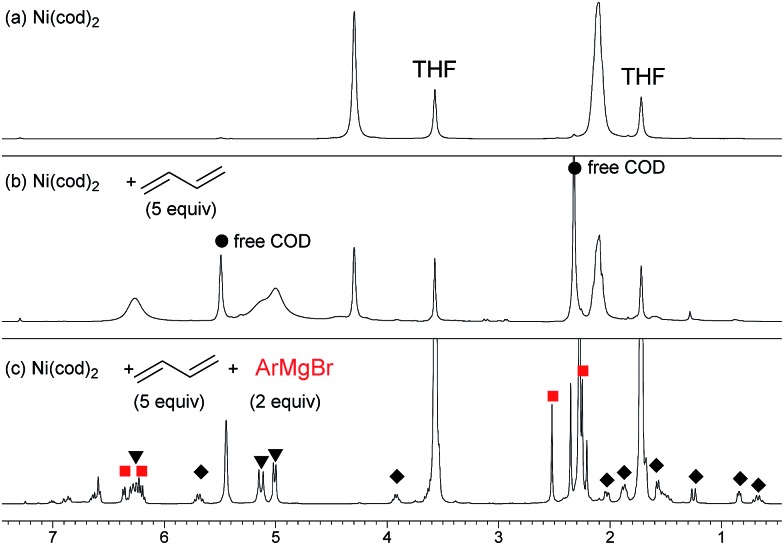
^1^H NMR spectra for the reaction of Ni(0) with 1,3-butadiene and **2m** in THF-*d*_8_ at 20 °C. (a) Ni(cod)_2_, (b) Ni(cod)_2_ and 5 equiv. of 1,3-butadiene, and (c) Ni(cod)_2_, 1,3-butadiene (5 equiv.), and **2m** (2 equiv.).

^1^H NMR studies on the reaction of Ni(cod)_2_ were conducted (eqn (3)).^12^ Although bis(π-allyl)nickel complex **A** has been proposed as the key intermediate in the oligomerization of 1,3-butadiene by a Ni catalyst,^26^,^27^ the reaction of Ni(cod)_2_ with 1,3-butadiene in THF-*d*_8_ did not show clear evidence of its formation, and broadened 1,3-butadiene peaks were observed along with those of free COD (Fig. 1a to b). This is probably due to the rapid equilibria between complex **A** and many possible π-complexes of Ni(0) as well as free 1,3-butadiene.^24^ Variable temperature (VT) NMR results of the mixture indicated that the equilibria are fast even at the low temperature of –55 °C.^24^ When the mixture of Ni(cod)_2_ and 1,3-butadiene was treated with 2,6-dimethylphenylmagnesium bromide (**2m**), the spectrum showed sharp signals including two allyl groups (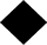) and one 2,6-dimethylphenyl group (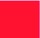) (Fig. 1c), which could be assigned to the corresponding nickelate complex **5**,^28^ along with signals of free 1,3-butadiene (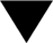). The formed nickelate complex **5** was stable in THF for a day even at room temperature.
3

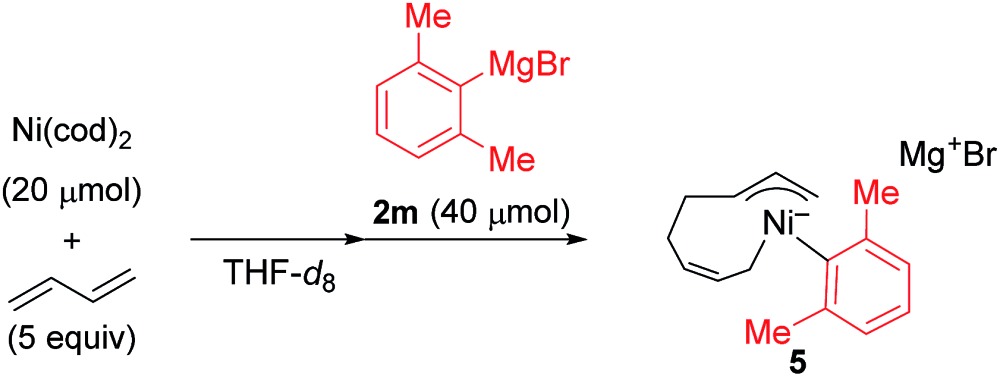




**Scheme 5:**
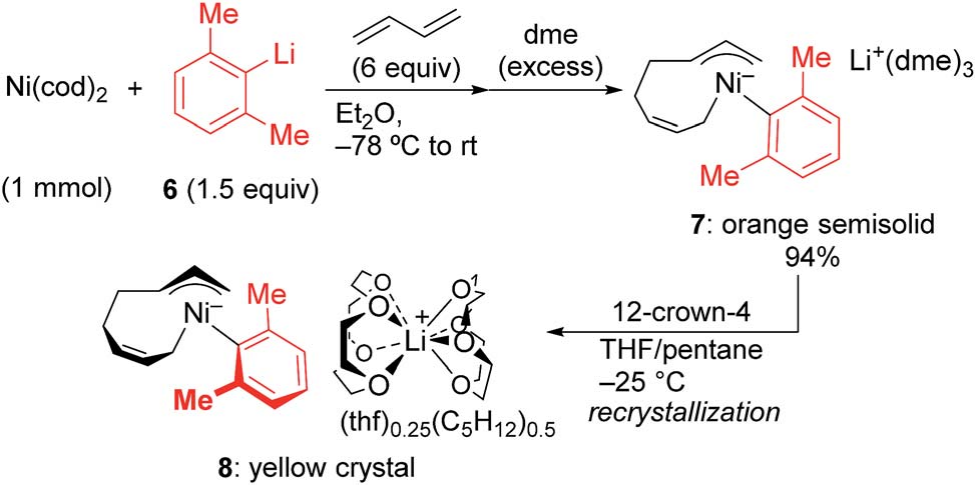
Isolation of anionic Ni complexes.

In order to isolate the nickelate complex, we tested various organometallic reagents and ligands for the counter cation. Lithium bearing polyether ligands such as 1,2-dimethoxyethane (DME) and 12-crown-4 ether were found to be a suitable counter cation. For instance, when Ni(cod)_2_ was treated with 2,6-dimethylphenyllithium (**6**) and 1,3-butadiene in Et_2_O and then DME was added, nickelate complex **7** was obtained as an orange semi-solid in 94% yield (Scheme 5). The ^1^H NMR spectrum of thus formed nickelate **7** is in good agreement with the above-mentioned NMR spectra when using the corresponding Grignard reagent **2m** (Fig. 1c).

**Fig. 2:**
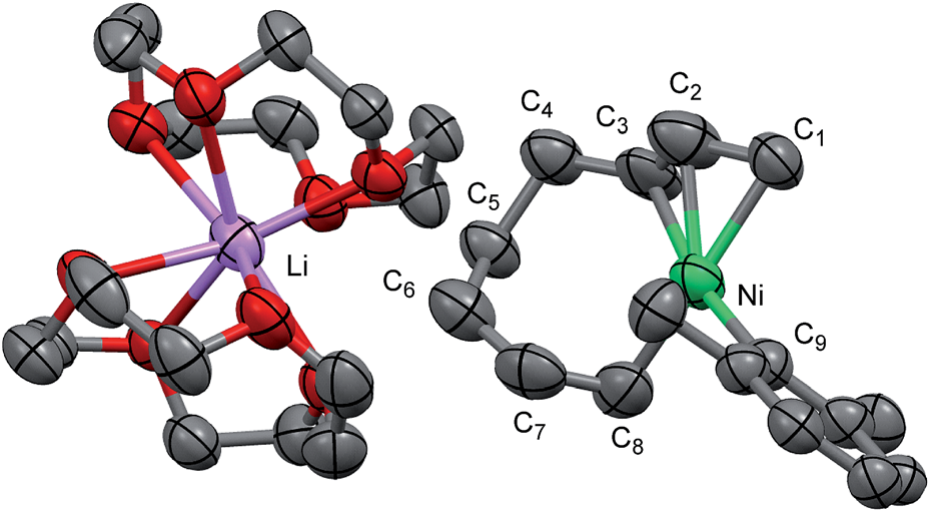
An ORTEP drawing of one of the four asymmetric units of [Ni(C_8_H_9_)(C_8_H_12_)·Li(12-crown-4)_2_]_4_(thf)(C_5_H_12_)_2_ (**8**) with thermal ellipsoids at the 50% probability level. All hydrogen atoms and solvent molecules are omitted for clarity.

Recrystallization of the nickelate complex **7** to obtain crystals suitable for X-ray was not successful. However, fine crystals of **8** were obtained by changing DME to 12-crown-4 (Scheme 5). As shown in Fig. 2,^24^,^29^ the crystal structure of **8** contains anionic Ni and the Li cation, where the octadiene moiety, generated by the oxidative dimerization of 1,3-butadiene, binds to the Ni center in η^1^,η^3^-fashion as expected. It is also revealed that the C–C double bond (C6=C7) of the σ-allyl group has the Z-configuration. The Ni center carries π-allyl, σ-allyl, and aryl groups in a square planar geometry.

#### 2.3.3. Reactivities of nickelate complexes

When the isolated Ni complex **7** was used as the catalyst, the four-component coupling reaction of *n*-OctF (**1a**), 2,6-dimethylmagnesium bromide (**2m**), and two molecules of 1,3-butadiene could proceed to give **3am** in 88% yield (eqn (4)).^12^
4

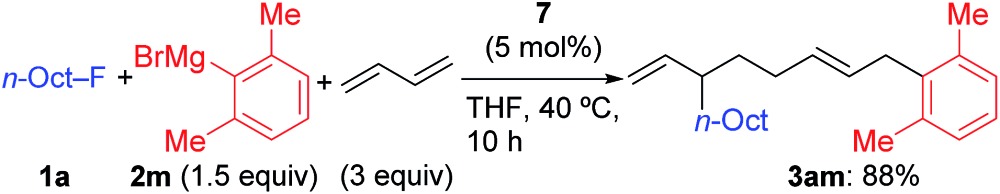




**Scheme 6:**
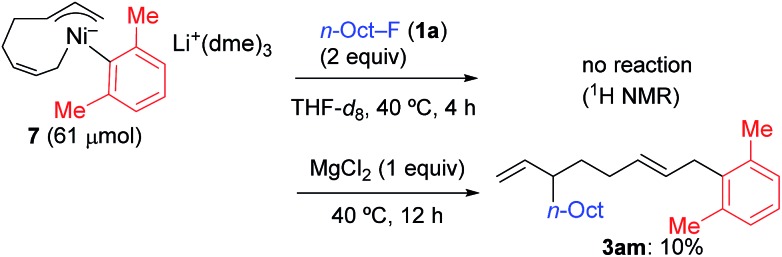
The stoichiometric reaction of nickelate complex **7** with **1a**: effect of the Mg ion.

**Scheme 7:**
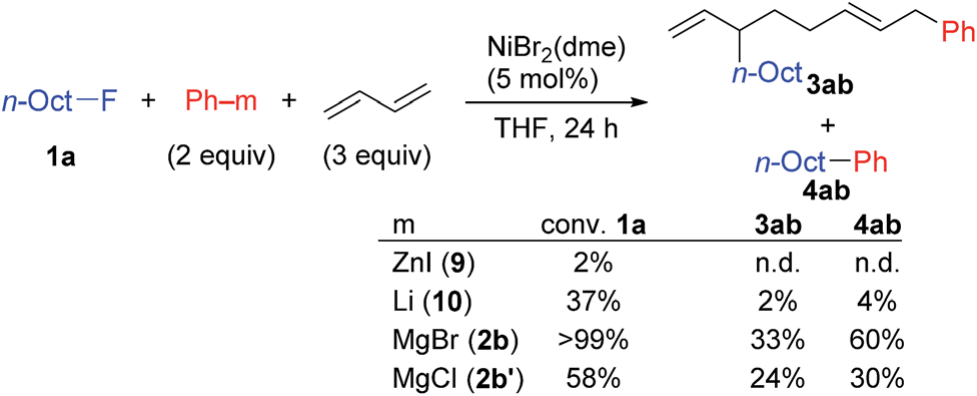
The reaction of arylzinc, aryllithium, and aryl Grignard reagents. Conversion and yields were determined by GC.

In contrast, when **7** was treated with 2 equiv. of *n*-OctF (**1a**) in THF-*d*_8_ at 40 °C for 4 h, no change was observed in its ^1^H NMR spectrum. The addition of MgCl_2_ to the reaction mixture promoted the reaction to give **3am** in 10% yield (Scheme 6).^12^ As shown in Scheme 7, when PhZnI (**9**) and PhLi (**10**) were used instead of Grignard reagents, neither four-component coupling product **3ab** nor cross-coupling product **4ab** was yielded.^12^ PhMgCl (**2b′**) also gave a mixture of **3ab** and **4ab** albeit in somewhat lower total yields compared to PhMgBr (**2b**) (Scheme 7).^30^ These results imply that the Mg cation contributes to the reaction of the nickelate intermediate **B** with alkyl fluorides to activate the C–F bond by coordination to F.^30^

#### 2.3.4. Kinetic studies

**Fig. 3:**
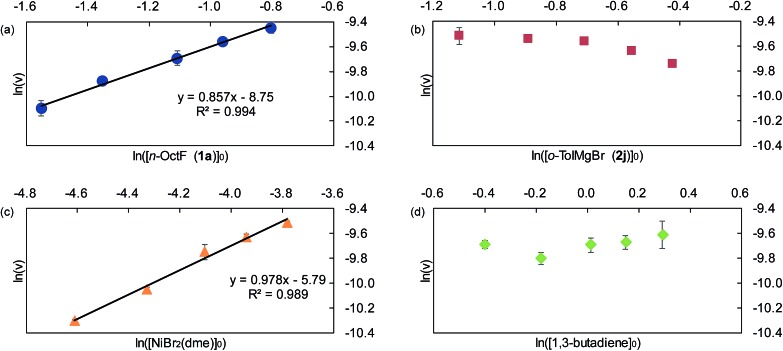
Double logarithm plots of the initial reaction rates against initial concentrations of each substrate. (a) [**1a**]_0_ = 0.21 to 0.45 M, (b) [**2j**]_0_ = 0.33 to 0.66 M, (c) [NiBr_2_(dme)]_0_ = 0.010 to 0.023 M, and (d) [1,3-butadiene]_0_ = 0.67 to 1.34 M.

We performed kinetic studies using *n*-OctF (**1a**) and *o*-TolMgBr (**2j**). Initial reaction rates were measured by changing the concentration of one reagent from the optimized conditions, which is **1a** at 0.33 M, **2j** at 0.50 M, NiBr_2_(dme) at 0.017 M, and 1,3-butadiene at 1.0 M at 40 °C.^31^ The rate of the four-component coupling reaction was plotted against the reagent concentration (Fig. 3), clearly showing first-order kinetics with respect to the catalyst and **1a**. The rate for the four-component coupling reaction can be expressed as d[**3aj**]/d*t* = *k*[NiBr_2_(dme)]^0.98^[**1a**]^0.86^, suggesting that the reaction of nickelate complex **B** with **1a** (step C) is the rate-determining step. Interestingly, the reaction rates do not depend on the concentrations of Grignard reagent **2j** or 1,3-butadiene, indicating steps A and B are relatively fast. At higher concentrations of the Grignard reagent (>*ca.* 0.5 M), the reaction became slower. The reason is not clear yet, but this might be due to the aggregation of Grignard reagents.

**Fig. 4:**
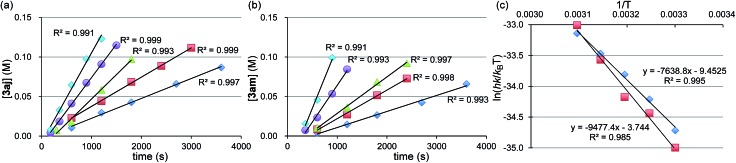
(a) Time-course of the reaction of **1a** with **2j** at 30 °C (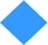), 35 °C (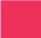), 40 °C (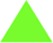), 45 °C (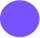), and 50 °C (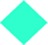). (b) Time-course of the reaction of **1a** with **2m** at 30 °C (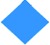), 35 °C (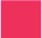), 40 °C (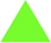), 45 °C (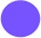), and 50 °C (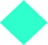). (c) Eyring plot of the reaction using **2j** (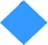) and **2m** (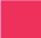).

Next, we conducted the reaction at different temperatures (30–50 °C) using *o*-tolyl- and 2,6-dimethylphenylmagnesium bromide (**2j** and **2m**), and the results are plotted in Fig. 4a and b, respectively. Eyring plots of these data employing the rate law d[**3a**]/d*t* = *k*_obs_[**1a**] showed good linear relationships (Fig. 4c), and the resulting parameters are summarized in Table 5. A similar analysis was also conducted for the reaction of *p*-fluorophenylmagnesium bromide (**2a**).

**Table 5:** Activation parameters of the four-component coupling reaction

ArMgBr	Δ*G*‡273 (kJ mol^–1^)	Δ*H*^‡^ (kJ mol^–1^)	Δ*S*^‡^ (J Kmol^–1^)
**2a**	87.4 ± 7.6	62.9 ± 3.9	–89.9 ± 12.4
**2j**	85.0 ± 5.1	63.5 ± 2.7	–78.6 ± 8.7
**2m**	87.3 ± 10.6	78.8 ± 5.6	–31.1 ± 18.0

These data indicate that the present four-component coupling reaction is mainly controlled by enthalpy factors in all cases employing Grignard reagents (**2a**, **2j**, and **2m**). The introduction of one methyl group into the *ortho*-position (*o*-TolMgBr (**2j**) *vs. p*-FC_6_H_4_MgBr (**2a**)) showed a small effect on the activation parameters, probably because the *ortho*-methyl group is located far from the reacting γ-allylic carbon in the transition state. In contrast, the activation parameters changed significantly upon the introduction of methyl groups into both *ortho*-positions (**2m**), which increased the activation enthalpy.^32^

Activation parameters of the Ni-catalyzed cross-coupling reaction of alkyl(pseudo)halides with *n*-BuMgCl in the presence of 1,3-butadiene were also determined: Δ*G*‡273 = 55.3 ± 6.0 kJ mol^–1^, Δ*H*^‡^ = 51.2 ± 2.6 kJ mol^–1^, and Δ*S*^‡^ = –14.8 ± 12.3 J Kmol^–1^ for iodide; Δ*G*‡273 = 61.9 ± 8.6 kJ mol^–1^, Δ*H*^‡^ = 57.3 ± 4.0 kJ mol^–1^, and Δ*S*^‡^ = –16.9 ± 16.7 J Kmol^–1^ for bromide; and Δ*G*‡273 = 71.1 ± 9.8 kJ mol^–1^, Δ*H*^‡^ = 52.7 ± 4.9 kJ mol^–1^, and Δ*S*^‡^ = –67.6 ± 18.0 J Kmol^–1^ for tosylate.25*b* These data correspond to step E in Scheme 4 and show similar tendency with step C, suggesting similar transition states between the four-component coupling and the cross-coupling reactions.

#### 2.3.5. Reaction mechanisms of the nickelate complex with alkyl fluorides

A unique feature of the present catalytic reaction is the facile cleavage of the C(sp^3^)–F bond.^22^ To elucidate the reaction mechanisms, we conducted the reaction of a radical clock **1n**^33^ with Grignard reagent **2a**, and obtained the four-component coupling product **3na** and the cross-coupling product **4na** in 37% and 38% yields, respectively, with no cyclized products (eqn (5)). Similarly, the reaction using the 2,6-dimethylphenyl Grignard reagent **2m** instead of **2a** selectively afforded **3nm** in 93% yield (eqn (6)).^12^ These results clearly indicate that nickelate intermediates cleave the C(sp^3^)–F bond *via* an ionic mechanism for both the four-component and cross-coupling reactions.
5

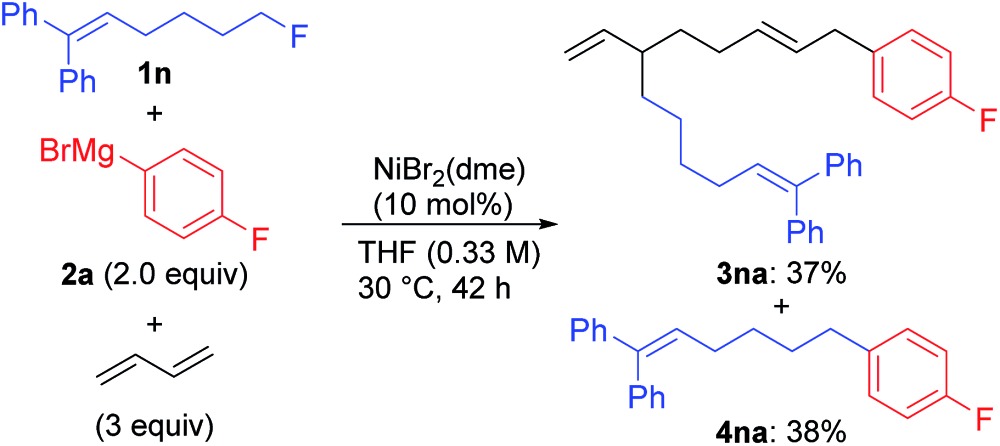



6

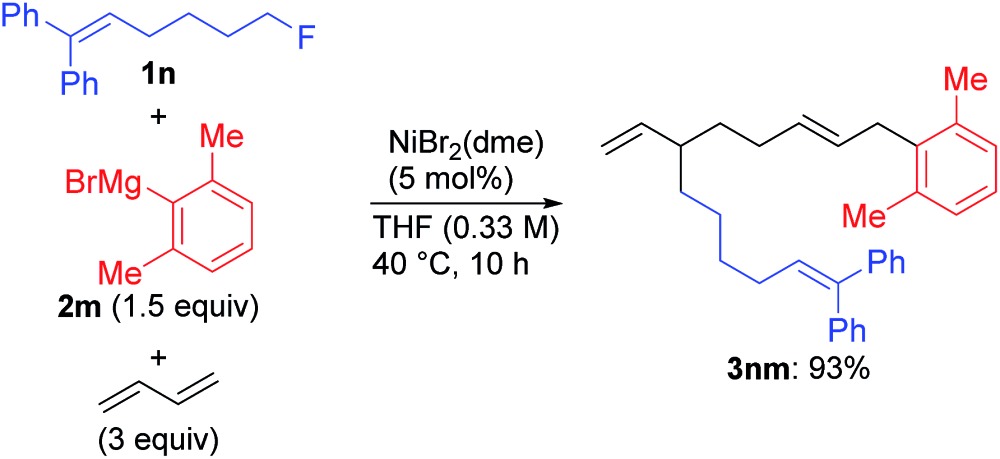




#### 2.3.6. Hammett plots regarding the rate-determining step (step C)

We next investigated the effects of the electron donating/withdrawing groups in the aryl Grignard reagents on the reaction of nickelate intermediates with alkyl fluorides. When a series of substituents (Cl, F, OMe, or Me) were introduced into the 4-position of the 2-methylphenyl Grignard reagent, a moderately large difference in the reaction rates was observed, with the electron-donating groups accelerating the reaction. With the observed initial reaction rates, the rate constant *k*_X_ for each Grignard reagent was calculated (Table 6).

**Table 6:** Reaction rates of different Grignard reagents

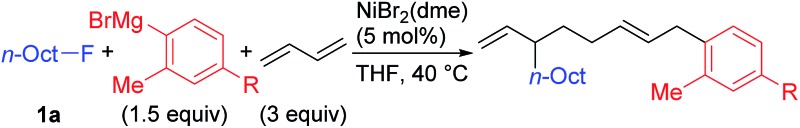					
R	H (**2j**)	Cl (**2p**)	F (**2o**)	OMe (**2q**)	Me (**2r**)
*k*_X_ (10^–3^ s^–1^)	12.7	4.80	7.47	12.8	17.8

**Fig. 5:**
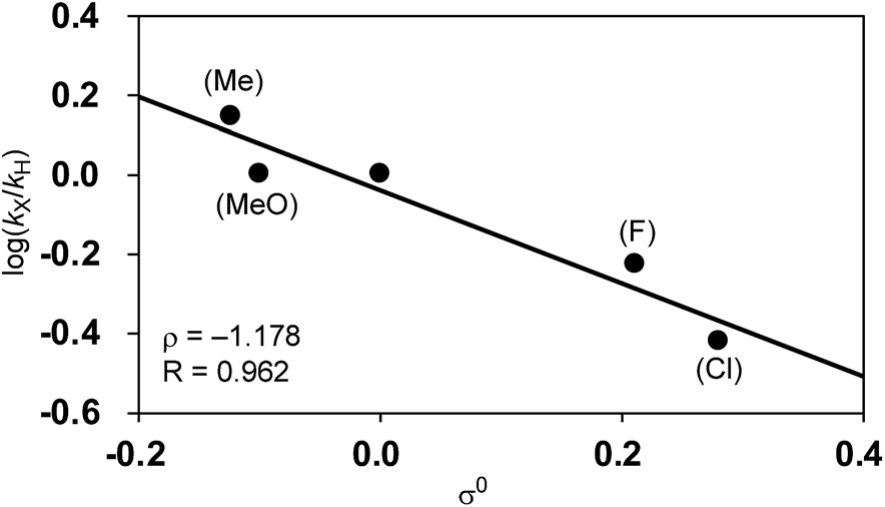
Hammett plot of the relative reaction rates against Yukawa–Tsuno’s *σ*^0^.

Among Hammett plots of various substituent constants, Yukawa–Tsuno’s *σ*^0^, which is determined by the electronic effects of the aromatic substituents on the reaction center connected by methylene tethers, showed a satisfactory linear relationship (*R* = 0.962) with a negative *ρ* value of –1.178 (Fig. 5). This negative *ρ* value is in accord with the S_N_2 mechanism of the rate-determining step, in which electron-donating substituents accelerate the nucleophilic attack by increasing the electron density of the allylnickel intermediates.

This result is also in good agreement with the observed electronic effects on the selectivity of the four-component coupling and cross-coupling reactions. As mentioned in Section 2.1 regarding the results in Scheme 2, electron-deficient Grignard reagents showed higher selectivities for four-component coupling over cross-coupling compared to electron-rich ones. For instance, the ratio between the four-component coupling product **3** and cross-coupling product **4** is 1.7 : 1 for the *p*-fluorophenyl Grignard reagent (**2a**), 1 : 1.9 for the *p*-tolyl Grignard reagent (**2c**), and 1 : 3.3 for the *p*-dimethylaminophenyl Grignard reagent (**2f**). This selectivity is related to the relative reaction rates of the nucleophilic attack by the γ-position of the σ-allyl group of nickelate intermediate **B** (step C) and that by the nickel center (step E). This can be explained by the fact that electron-donating groups on the aryl group increase the electron density on the directly connected Ni more effectively, compared to that on the remote γ-carbon of the σ-allyl group.

**Fig. 6:**
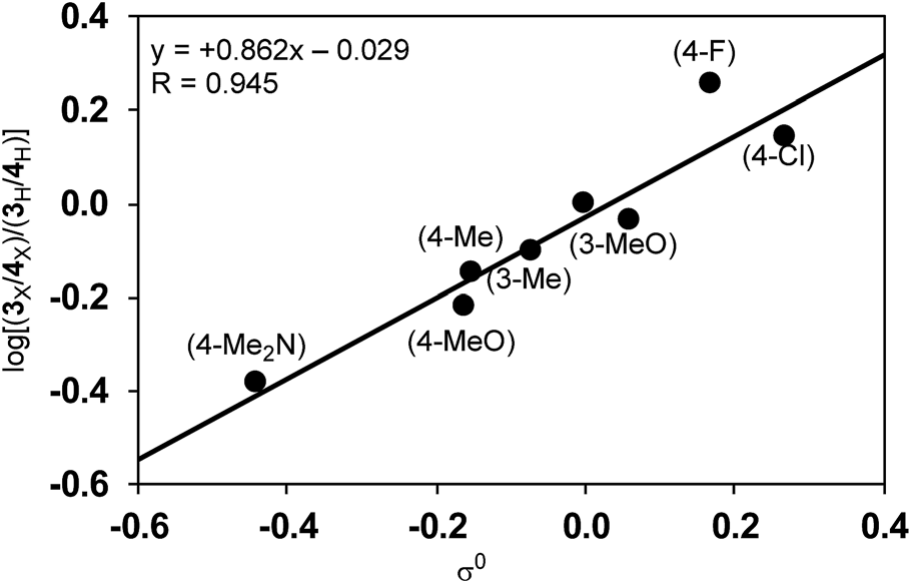
The relationship between the product ratio **3**/**4** of substituted Grignard reagents against *σ*^0^.

Since the selectivity is determined by the relative rates of step C *vs.* step E in Scheme 4, the product ratio **3**/**4** can be expressed by the relative rate constants between step C and step E (*k*_C_/*k*_E_). Therefore, the ratio of selectivity between X and H, (**3**_X_/**4**_X_)/(**3**_H_/**4**_H_), is equal to (*k*_CX_/*k*_EX_)/(*k*_CH_/*k*_EH_) = (*k*_CX_/*k*_CH_)/(*k*_EX_/*k*_EH_) = *ρ*_C_/*ρ*_E_. A plot of log[(**3**_X_/**4**_X_)/(**3**_H_/**4**_H_)] against the substituent constants (Yukawa–Tsuno’s *σ*^0^) shows a good linear relationship (*R* = 0.945) with a slope of +0.862 (Fig. 6).^34^ With *ρ*_C_ = –1.178 for step C, *ρ*_E_ for the cross-coupling reaction (step E) is calculated to be –1.366. The relatively large negative value of *ρ*_E_ in comparison to *ρ*_C_ is also ascribable to the direct connection of the Ni reaction site with the Ar group.

#### 2.3.7. Steric effects on selectivity: four-component coupling *vs.* cross-coupling reactions

**Fig. 7:**
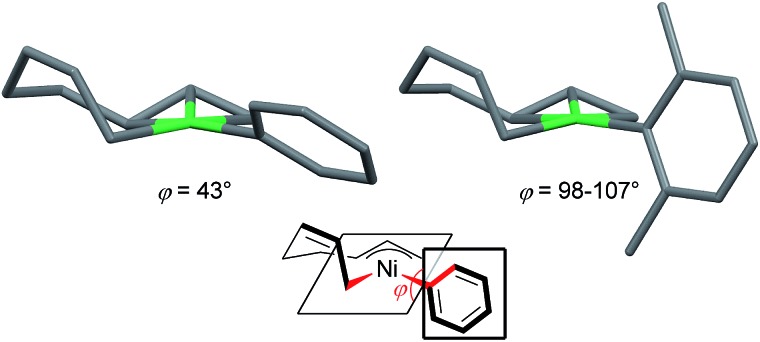
The molecular structures of nickelate complexes **11** (left) and **8** (right). Bottom: the dihedral angle between the square planar Ni plane and the aryl ring.

The effect of the *ortho*-substituent(s) on aryl Grignard reagents to suppress the cross-coupling reaction could be explained by the molecular structure of nickelate intermediates. Fig. 7 shows the crystal structure of the nickelate complexes having phenyl (**11**)^35^ and 2,6-dimethylphenyl (**8**) groups. Although the octadienediyl moiety binds to the Ni center in the same fashion in both cases, the orientation of the aryl ring shows a large difference: the dihedral angle (*φ*) of the α-C of σ-allyl, Ni, *ipso*-C, and *ortho*-C are 43° for **11**, and 98 to 107° for **8**.

**Fig. 8:**
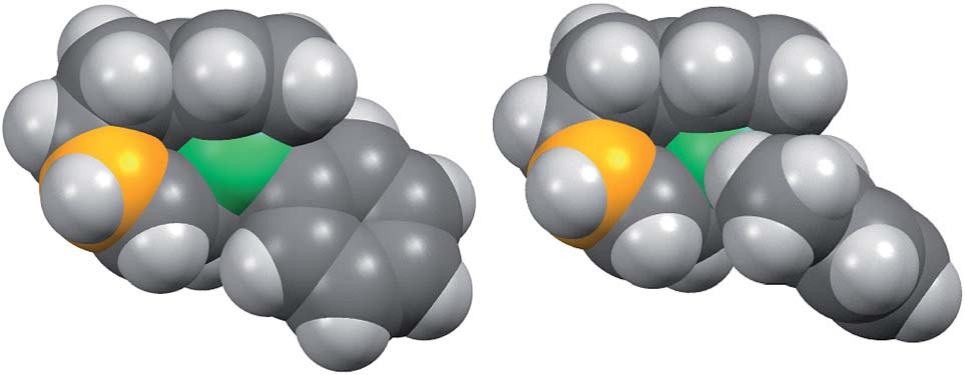
Space-filling models of the nickelate complexes (top view) in Fig. 7. The reacting carbon of the σ-allyl group and Ni atom are shown in orange and green, respectively.

Fig. 8 shows the space-filling model of complexes **11** and **8**. The Ni center (green) of phenyl complex **11** is open to the approach of alkyl fluorides (Fig. 7, left) while such an approach to the Ni of complex **8** is blocked by the orthogonally oriented 2,6-dimethylphenyl group (right). Therefore, alkyl fluorides react with the less hindered γ-carbon of the σ-allyl group (orange), giving rise to the four-component coupling product **3**.

#### 2.3.8. Competitive reactions

**Fig. 9:**
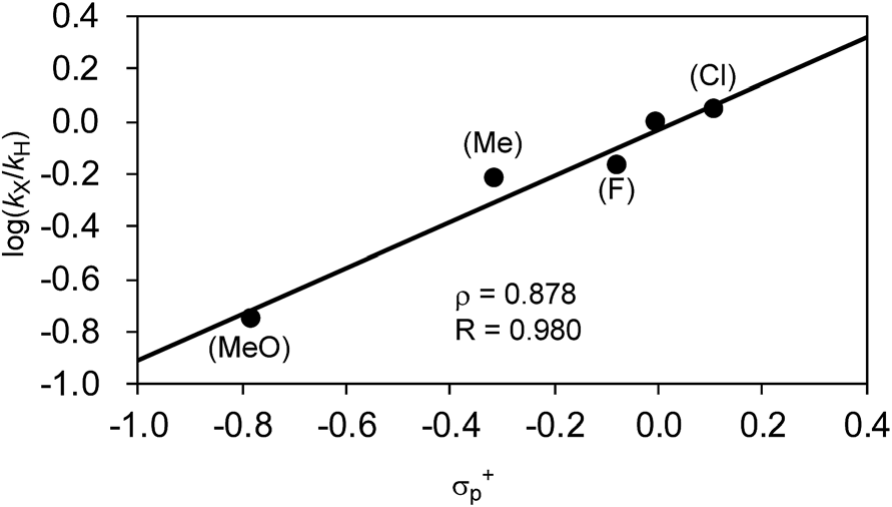
Hammett plot of the relative reaction rates obtained in the competitive reaction against *σ*+p.

Next, we conducted competitive reactions of *o*-tolyl Grignard reagents with/without a *para*-substituent (**2j***vs.***2o–2r**), and the relative rates *k*_X_/*k*_H_ and total yields at the early stage of 40 min reaction time are shown in Table 7. The Hammett plot of log(*k*_X_/*k*_H_) against *σ*+p shows a linear relationship with a relatively small positive *ρ* value of +0.878 (Fig. 9), indicating that electron-withdrawing substituents favor the formation of four-component coupling products in competitive reactions. This is in large contrast to the separate reactions in which the electron-rich Ar group facilitated the reaction as in Table 6 and Fig. 5.

**Table 7:** The competitive reaction of Grignard reagents

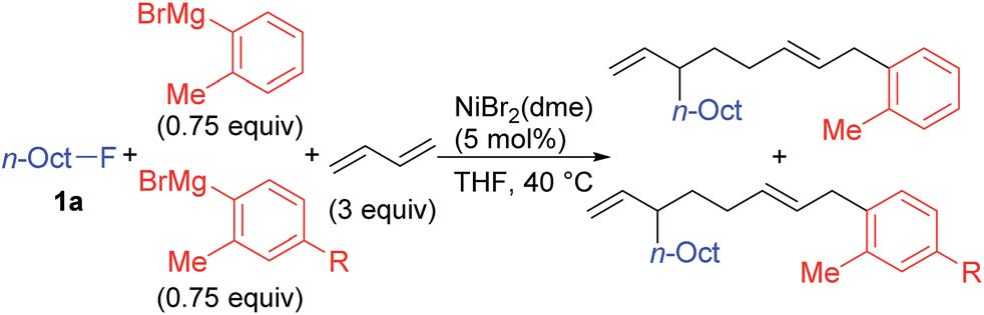			
Entry	R	*k*_X_/*k*_H_	Total yield (%)^*a*^
1	H (**2j**)	—	36
2	Cl (**2p**)	1.12	25
3	F (**2o**)^*b*^	0.68	34
4	Me (**2q**)	0.61	46
5	OMe (**2r**)	0.18	43

^*a*^Total yield of the products at 40 min.

^*b*^Due to the difficulty in analysis, the competitive reaction with 2,4-dimethylphenylmagnesium bromide (**2q**) instead of **2j** was performed to give *k*_F_/*k*_Me_ = 1.12 and calculated with *k*_Me_/*k*_H_ = 0.61.

Another important finding from Table 7 is that electron-withdrawing substituents retard the reaction and produce lower total yields. For instance, when the *o*-tolyl Grignard reagent (**2j**) was used alone, 36% of **3aj** was yielded after 40 min (entry 1). The competitive reaction of **2j** and the electron-deficient **2p** produced a mixture of **3aj** and **3ap** with a combined yield of 25% (entry 2). The use of the electron-rich Grignard reagent **2r** along with **2j** afforded products in 43% total yield (entry 5). These results may indicate that the nickelate complex-forming step (step B) prefers electron-deficient Grignard reagents. However, the formed nickelate with the electron-deficient Ar group reacts with alkyl fluorides at relatively slow rates compared to those formed with electron-rich ones.

### 2.4. Computational studies

The mechanisms of the four-component coupling and cross-coupling reactions were theoretically investigated using density functional theory calculations. Molecular structures were optimized at the M06 (ref. 36)/6-31G(d,p) level of theory using the Gaussian 09 (ref. 37) program, followed by single-point energy calculations at the M06/6-311+G(d,p) level with the SMD^38^ model (THF). The energy reported here includes the electronic energy, zero-point energy, thermal correction at 298 K and 1 atm, and solvent effect correction. The molecular structures were drawn with CYLview.^39^ The experimental findings discussed in Section 2.3 revealed that nickelate complexes are readily formed under the reaction conditions, and the reaction of the nickelate complex with alkyl fluorides is the rate- and selectivity-determining step of the whole catalytic cycle. In order to reveal two competing reaction pathways, we thus focused on the reaction of the nickelate complex with alkyl fluorides to give the four-component coupling and cross-coupling products.

#### 2.4.1. Reaction pathways

Previously, we^40^ and another research group^41^ reported theoretical calculations of the cross-coupling reaction of alkyl halides with alkyl Grignard reagents involving nickelate complexes as catalytic active species.^15^ In both studies, it was revealed that S_N_2 attack of the nickelate complex toward alkyl halides, which corresponds to step E in Scheme 4, is the rate-determining step. However, due to the difficulties of building the initial models of anionic intermediates, the Mg cation was omitted in our calculations.^40^ Chass, Kantchev, and Fang employed a simple model without methylene tethers.^41^ In addition, the reaction of alkyl fluorides was not considered in these reports. In the latter study,^41^ a very large Δ*G*^‡^ value of 138 kJ mol^–1^ was evaluated for the reaction of an anionic Ni complex with EtBr, which is obviously inconsistent with our experimental observations that the activation energy of the corresponding process employing *n*-NonBr is as low as Δ*G*‡273 = 61.9 ± 8.6 kJ mol^–1^.25*b*

One of the most critical reasons to give such inaccurate results is due to the structure and position of the counter cation, which coordinates to not only the leaving group (Br) but also a π-ligand in the anionic part, resulting in the strained structure of the transition states.^41^,^42^ Such cation–π interactions are often encountered in theoretical calculations of ate complex-mediated transformations.^43^,^44^

As mentioned above, the Mg cation contributes to both the four-component coupling and the cross-coupling reactions (Scheme 7), and the cationic part (Mg^+^Br) of the nickelate complex would exist as solvent separated ion pair in THF.^45^ Therefore, we chose models that have four THF molecules on the MgBr cation to fulfill the six coordination sites of Mg in the TSs. The chosen solvent separated ion pair mode led many local minima throughout the theoretical calculations because of not only the many possibilities of the relative position of the anionic Ni moiety and Mg cation^46^ but also the flexibility of the octadienediyl moiety and alkyl fluorides. However, this model provides more reasonable results compared to a model containing MgBr as a naked counter cation. The following discussions are carried out by typical optimized structures employing Mg^+^Br·4THF as the counter cation.

**Fig. 10:**
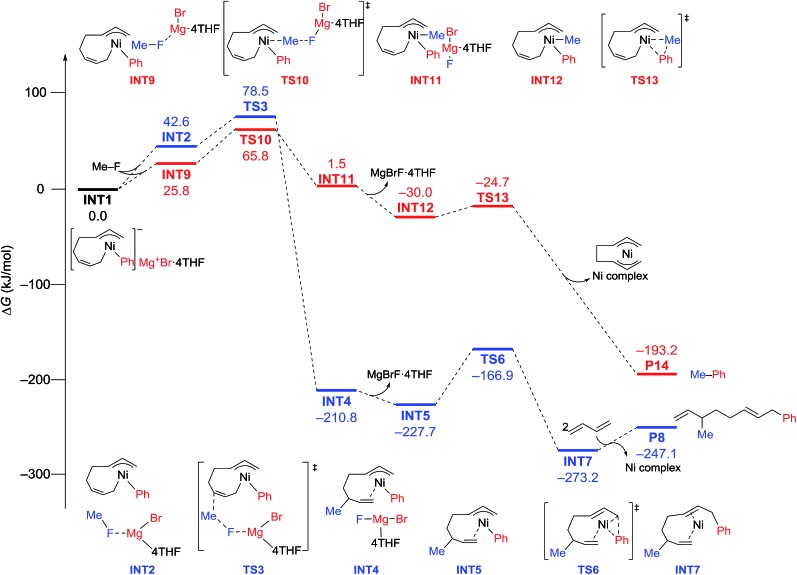
Reaction pathways of nickel-catalyzed four-component coupling (blue) and cross-coupling (red) reactions of methyl fluoride, phenyl Grignard reagent, and 1,3-butadiene.

**Fig. 11:**
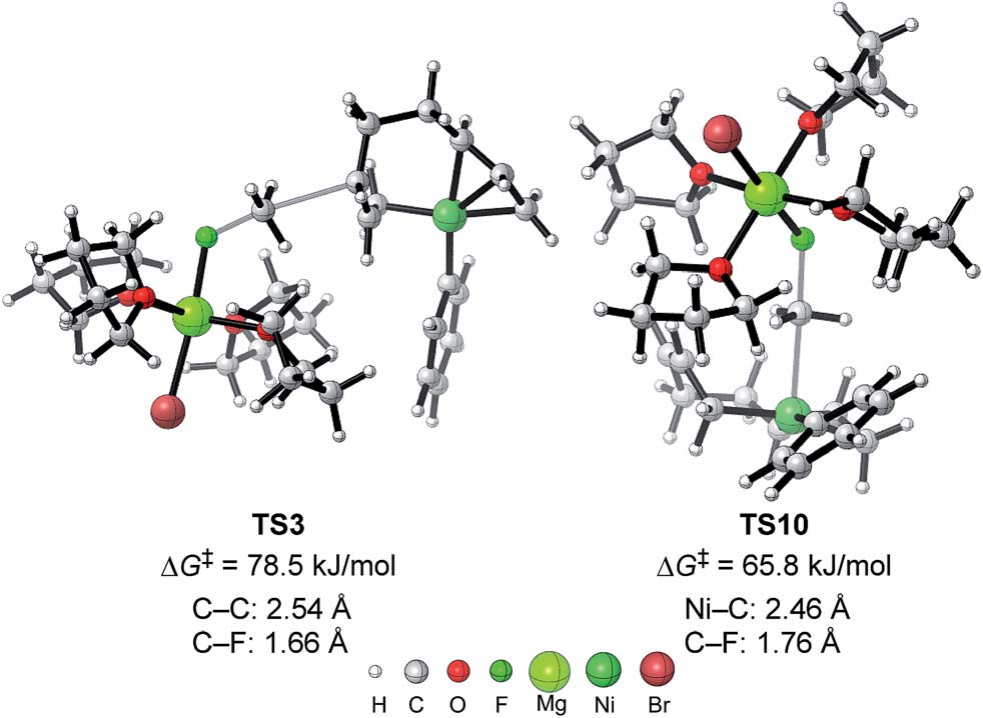
The molecular structures of important transition states in Fig. 10 with selected bond distances.

The energy profiles and the corresponding key transition states are shown in Fig. 10 and 11, respectively. All the other geometric structures are presented in the ESI.† From **INT1**, there are two different pathways that lead to four-component coupling and cross-coupling products. On one hand, the methyl fluoride is inserted with the methyl group pointing to the γ-carbon, forming an intermediate **INT2** with a relative energy of 42.6 kJ mol^–1^. *Via* a transition state **TS3** (78.5 kJ mol^–1^), the methyl group is transferred to the γ-carbon of the σ-allyl group giving rise to **INT4**. The energy of **INT4** is as low as –210.8 kJ mol^–1^, revealing that the bond formation between the methyl group and γ-carbon is largely exothermic. Then, MgBrF·4THF dissociates from the reaction system, and **INT5** is formed with an energy of –227.7 kJ mol^–1^. The subsequent reductive elimination takes place between the phenyl group and the terminal carbon *via***TS6** with a low energy barrier of 60.8 kJ mol^–1^ to produce **INT7**. Finally, **INT7** exchanges with two 1,3-butadiene molecules to form the product **P8**.

On the other hand, MeF can be inserted into **INT1** to form **INT9** (25.8 kJ mol^–1^), with the methyl group pointing to Ni. Next, the methyl group is transferred to Ni *via* a transition state **TS10** (65.8 kJ mol^–1^) to form **INT11** with an energy of 1.5 kJ mol^–1^. After elimination of MgBrF·4THF with an energy release of 31.5 kJ mol^–1^, **INT12** is generated, in which both phenyl and methyl groups are bound to Ni. *Via* a transition state **TS13**, which has an energy barrier of 5.3 kJ mol^–1^, reductive elimination occurs between the phenyl and methyl groups, giving rise to the product **P14**.

The above results revealed that the rate-determining step is the methyl group transfer, which corresponds to **TS3** and **TS10** for the two competing pathways, respectively. The overall energy barriers of the four-component coupling and cross-coupling reactions are 78.5 (**INT1** to **TS3**) and 65.8 kJ mol^–1^ (**INT1** to **TS10**), respectively. This appreciably large difference may be due, in part, to overestimation of the C–H–π interaction between THF on the Mg cation and the Ph group in **TS10** that would not be probable, or would exert only a small effect if possible, in the practical conditions surrounded by THF molecules as the solvent (*vide infra*).

As shown in Fig. 11, in both **TS3** and **TS10** the Mg cation moiety (Mg^+^Br·4THF) binds to the fluorine atom to associate the elimination process, where no other short contact between the Mg^+^Br·4THF moiety and the anionic part is found, and therefore the angle of the participating atoms, C–C–F for **TS3** and Ni–C–F for **TS10**, is 171° and 177°, respectively. These linear structures of the TSs clearly agree with the S_N_2 mechanism of the C–C bond forming step of both reaction pathways.

#### 2.4.2. Steric effects on the rate-determining step

To analyze the effect of *ortho*-substitution on the selectivity, we initially conducted exploratory calculations using a set of models including both isomers arising from the orientation of the unsymmetric *o*-Tol group at the M06/6-31G(d,p) level of theory, where 2-methyl-up means that the methyl group on the *o*-Tol group is in the same direction as the σ-allyl group and 2-methyl-down means the opposite isomer,^47^ and revealed that **TS10-2-methyl-down** is the most energetically favorable of four possible TSs (Fig. S19†). This is due to the short C–H–π interaction between THF on the Mg cation and the *o*-Tol group. When one THF molecule was introduced between the Mg cation and *o*-Tol group to suppress the interaction, **TS3** became much more favorable than **TS10** in both isomers (+33.1 kJ mol^–1^ for up and +14.4 kJ mol^–1^ for down) (Fig. S20†).^24^ As the nickelate is surrounded by solvent molecules (THF) under the practical conditions, the latter models involving five THF molecules seem to be more probable and consist with the experimental results. However, due to the flexibility of the structures, there are many local minima of the model with five THF molecules, thereby, we decided to conduct further theoretical calculations using the models containing four THF molecules on the Mg cation.

Other important findings in the introductory investigation are as follows: (i) a configuration orienting the *ortho*-methyl group toward the reacting face is energetically favored in **TS3** (**TS3-2-methyl-up**) compared to the opposite orientation (**TS3-2-methyl-down**) (ΔΔ*G*^‡^ = 2.5 kJ mol^–1^), (ii) in **TS10**, a configuration orienting the *ortho*-methyl group to the opposite side (**TS10-2-methyl-down**) is more favorable than **TS10-2-methyl-up** (ΔΔ*G*^‡^ = 18.7 kJ mol^–1^), and (iii) the β-hydrogens of the alkyl fluorides are close to the aryl group in both cases of **TS-10-2-methyl-up** (H–H distances with the *ortho*-methyl group are 2.13 and 2.32 Å) and **TS-10-2-methyl-down** (H–H distances with the *ortho*-C–H bond are 2.24 and 2.24 Å). These short H–H distances may contribute to the increased energy barrier of **TS10**, resulting in high four-component coupling selectivity.^24^

**Fig. 12:**
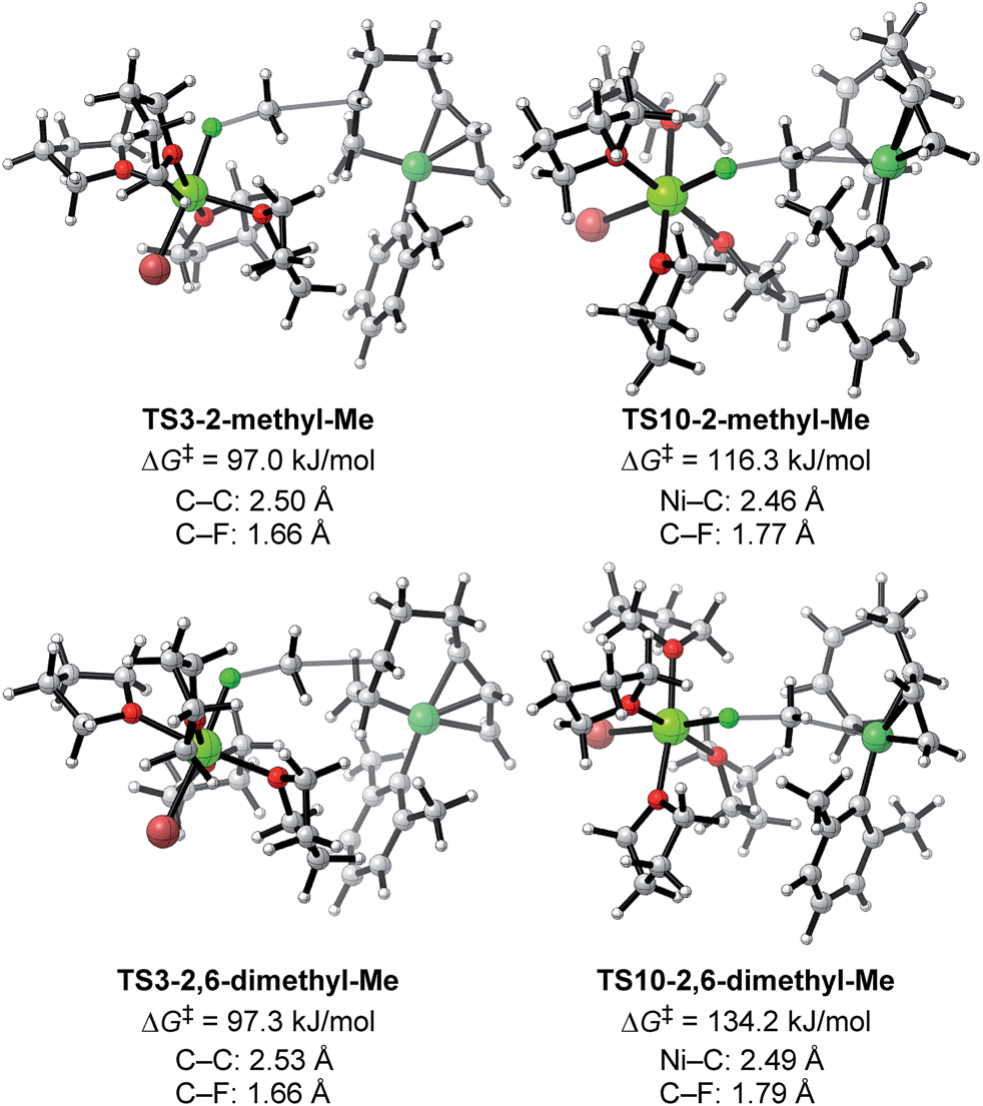
Optimized structures and the corresponding energy barriers for the transition states of γ-carbon attack (**TS3**) and Ni-attack (**TS10**) toward MeF for 2-methylphenyl and 2,6-dimethylphenyl Grignard reagents. Bond distances between key atoms are given.

**Fig. 13:**
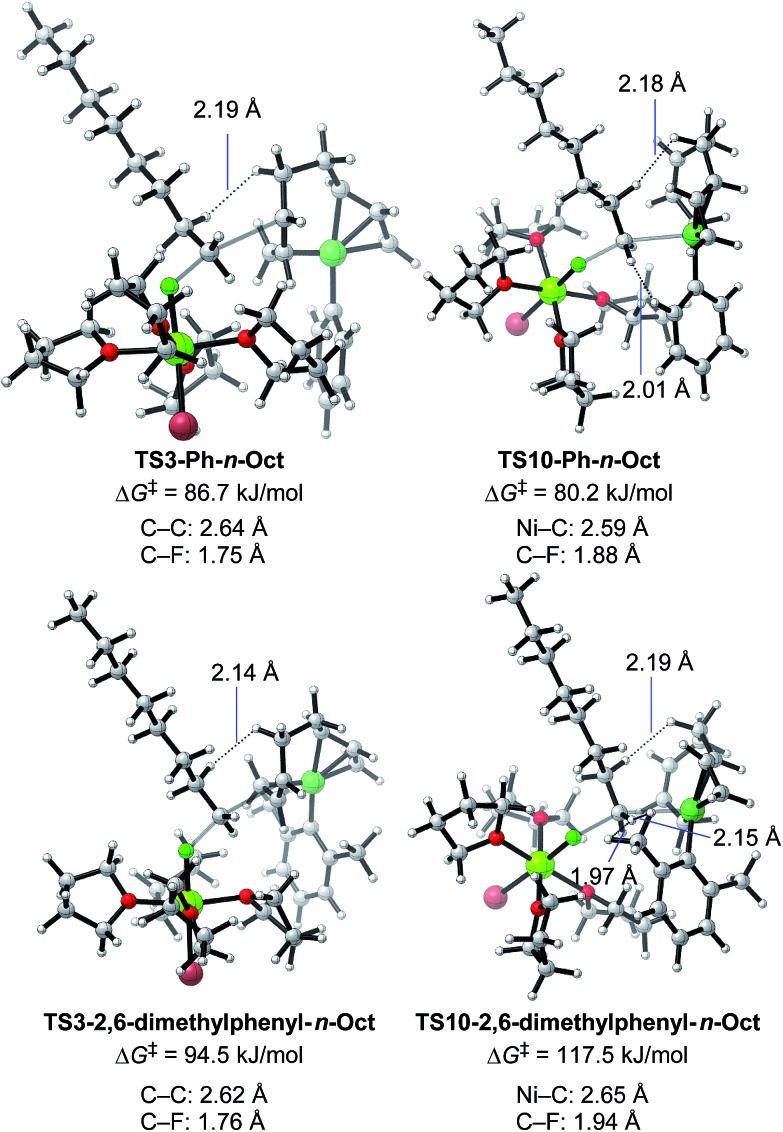
Optimized structures and the corresponding energy barriers for the transition states of γ-carbon attack (**TS3**) and Ni-attack (**TS10**) toward *n*-OctF for phenyl and 2,6-dimethylphenyl Grignard reagents. Bond distances between key atoms and representative short H–H distances are given.

Next, **TS3** and **TS10** with various aryl Grignard reagents and alkyl fluorides were calculated at the higher level of theory (M06/6-311+G(d,p)), and the results are summarized in Table 8, Fig. 12 (for MeF), and Fig. 13 (for *n*-OctF). Introducing the first *ortho*-methyl group significantly increases the energy barriers of both **TS3** and **TS10**. In particular, irrespective to the alkyl fluorides, **TS10** has an energy barrier above 106 kJ mol^–1^, and therefore is clearly disfavored compared with **TS3**. In the case of the 2,6-dimethylphenyl Grignard reagent, the energy barrier of **TS10** increases to 134.2 kJ mol^–1^. However, in this case, the energy barrier of **TS3** is much lower (97.3 kJ mol^–1^). From these results, it is clear that the *ortho*-methyl group significantly influences the selectivity between the four-component coupling and cross-coupling reactions by favoring the former.

**Table 8:** Calculated energy barriers (in kJ mol^–1^) of **TS3** and **TS10** with various aryl Grignard reagents (R^1^–MgBr) and alkyl fluorides (R^2^–F)

R^1^	R^2^	**TS3**	**TS10**	ΔΔ*G*^‡^ (**TS3–TS10**)
Ph	Me	78.5	65.8	12.7
Ph	*n*-Oct	86.7	80.2	6.5
2-Methylphenyl	Me	97.0	116.3	–19.3
2-Methylphenyl	*n*-Oct	98.3	106.9	–8.6
2,6-Dimethylphenyl	Me	97.3	134.2	–36.9
2,6-Dimethylphenyl	*n*-Oct	94.5	117.5	–23.0

We then calculated **TS3** and **TS10** using *n*-OctF instead of MeF (Table 8). The structure of the alkyl fluoride affects the energy barriers at the transition states (especially for **TS10**), but it has no effect on the selectivity once the *ortho*-methyl group is present. Those computational results agree well with the experimental observations (Table 5). In the case of **TS10**, the energy barrier of MeF rises by far more than that of *n*-OctF by increasing steric hindrance of the aryl group. These results are due to their tight structure, where the sum of the C–F and C–Ni bond lengths of **TS10** with MeF is 4.22 Å (R^1^ = Ph, R^2^ = Me: Fig. 11) in sharp contrast to the long bond length of **TS10** with *n*-OctF (4.47 Å for R^1^ = Ph, R^2^ = *n*-Oct: Fig. 13).^24^ These differences in **TS10** by alkyl fluorides reflect the reaction mechanism, where **TS10** with MeF is a pure S_N_2 mechanism due to the lower stability of the Me cation, but **TS10** with *n*-OctF has a somewhat S_N_1 character. The natural bond orbital (NBO)^48^ analysis of these TSs at the M06/6-311+G(d,p)-SMD(THF) level of theory supports the difference in mechanism, where the atomic charge of the reacting carbon in **TS10** with *n*-OctF is more positive than that of **TS10** with MeF.^24^ Therefore, in the case of **TS10** with *n*-OctF, the bond distances of C–F and C–Ni could be elongated to minimize steric repulsions. The **TS10** with *n*-OctF seems to be more likely and well-describes the effect of the counter cation in the C–F bond cleavage step.

As discussed in Section 2.3.7, the *ortho*-substitution effect mainly stems from the steric effects in the nickelate complexes. Due to the *ortho*-substitution(s), the Ni center that acts as the reacting site at transition state **TS10** is partly covered, resulting in an increased energy barrier for **TS10**. To reveal the origin of the steric effect, the structures of **TS3** and **TS10** based on phenyl and 2,6-dimethylphenyl Grignard reagents are shown in Fig. 13 with representative short contacts. A short H–H distance is found between the β–H of the *n*-Oct group and the δ–H of the σ-allyl group in both cases of **TS3-Ph-*n*-Oct** (2.19 Å) and **TS3-2,6-dimethylphenyl-*n*-Oct** (2.14 Å) though a similar short distance is also found in **TS10**, where the β–H of the *n*-Oct is close to a methylene proton (2.18 and 2.19 Å). These short H–H distances are not the principal factor on the observed selectivity. On the other hand, compared with the H–H distance of 2.01 Å in **TS10-Ph-*n*-Oct**, the H–H distance in **TS10-2,6-dimethyl-*n*-Oct** is as short as 1.97 Å, indicating that there is an obvious repulsive interaction between the transferred *n*-Oct group and *ortho*-methyl group, which is responsible for the large increment in the energy barrier of **TS10**.

#### 2.4.3. Electronic effects on the rate-determining step

Besides the steric effects, the electronic effects also affect the rate-determining step and reaction rates. As shown in Section 2.3.6, the electron-donating group at the 4-position of the 2-methylphenyl Grignard reagent accelerates the reaction, whereas the reaction slows down when an electron-withdrawing group such as F and Cl is present at this position. To investigate the electronic effects, the energy barrier of **TS3** based on the 2-methylphenyl Grignard reagent with various substituents at the 4-position were calculated (Table 9). Only **TS3** was considered here, because **TS10** has been revealed to be highly disfavored as shown in Fig. 12. Compared with the case of 2-methylphenyl (R = H), substitution by F and Cl at the 4-position increases the energy barrier of **TS3**, while the energy barrier is significantly reduced when an electron-donating group such as OMe and NMe_2_ is introduced. These theoretical results are consistent with the experimental observations mentioned above.

**Table 9:** Calculated energy barriers (in kJ mol^–1^) of **TS3** based on 2-methyl-4-*R*-phenyl Grignard reagents and methyl fluoride and the charge (|*e*|) of γ-carbon

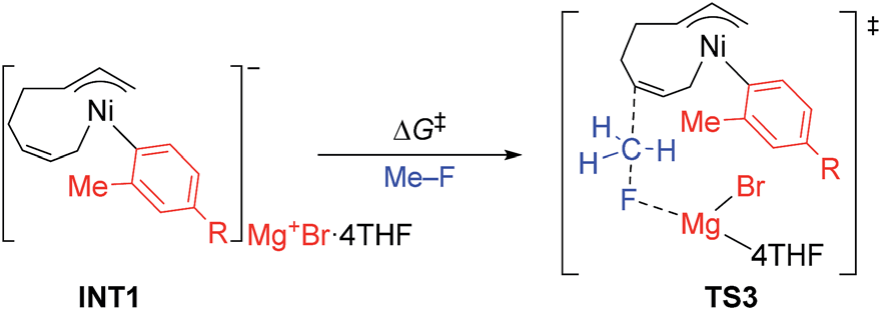						
R	H	F	Cl	Me	OMe	NMe_2_
Δ*G*^‡^	97.0	98.3	97.6	94.9	92.2	91.2
γ-C	–0.388	–0.387	–0.376	–0.390	–0.388	–0.390

NBO analysis was performed to further study the charge on the γ-carbon of the σ-allyl group in **TS3**. As shown in Table 9, the 4-position substitution moderately affects the charge state of the γ-carbon: electron-donating groups here increase the charge while electron-withdrawing groups reduce it.^49^ This is in good agreement with the reaction mechanism and observed electronic effects on the reaction rate (Section 2.3.6).

To further study the electronic effects on the selectivity between four-component coupling and cross-coupling reactions, the energy barriers of **TS3** and **TS10** based on *para*-substituted phenyl Grignard reagents and *n*-OctF were calculated, and ΔΔ*G*^‡^ of the two reaction pathways (**TS3–TS10**) and selectivities based on experimental results are summarized in Table 10. The calculated results generally agree well with the experimental observations. The electron-withdrawing groups such as F and Cl at the 4-position obviously destabilize **TS10** by 2.7 and 1.9 kJ mol^–1^ compared to **TS3**, respectively, and therefore, favor the four-component coupling product **3**. The electron-donating groups (Me and NMe_2_), on the other side, disfavor **TS3** and gives rise to the reverse ratio of **3** : **4**. Those results suggest that the electronic effects play an important role in the reactions with phenyl Grignard reagents (no *ortho*-substitution) though the impact on selectivity is relatively smaller than that from steric effects (Section 2.4.2).

**Table 10:** The difference in calculated energy barriers (in kJ mol^–1^) of **TS3** and **TS10** based on *p*-RC_6_H_4_MgBr and *n*-OctF

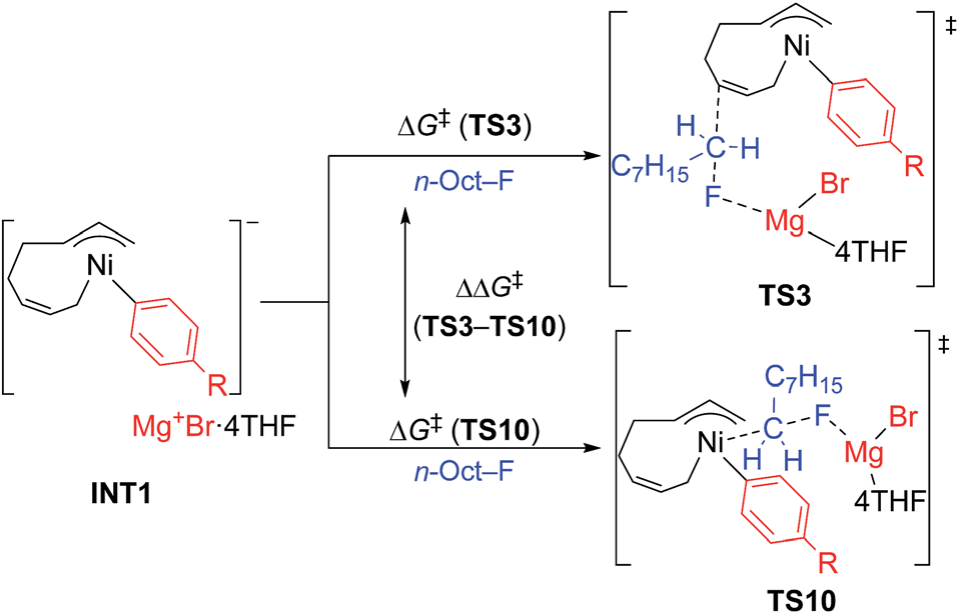						
R	H	F	Cl	Me	OMe	NMe_2_
ΔΔ*G*^‡^	6.5	–2.7	–1.9	5.0	5.0	8.9
Exp. ratio (**4**/**3**)	1.4	0.60	1.0	1.9	2.3	3.3

## 3 Conclusions

We examined the reaction mechanism, substituent effects, and origins of the selectivity of the Ni-catalyzed four-component coupling of alkyl fluorides, aryl Grignard reagents, and two molecules of 1,3-butadiene and the competing cross-coupling reaction. The introduction of *ortho*-substituents into the aryl Grignard reagents efficiently suppresses the cross-coupling reaction, giving four-component coupling products in good yields with perfect regio- and stereoselectivity. The present catalysis selectively provides four-component coupling products and does not form three-component coupling products without the dimerization of 1,3-butadiene, in sharp contrast to previous reports of a similar Ni-catalyzed multicomponent coupling reaction employing 1,3-dienes and carbonyl electrophiles.^7^ This difference arises from the different mechanism in the present reaction: the anionic Ni intermediates generated by the reaction of Ni(0) with two 1,3-butadiene molecules and aryl Grignard reagents are the key intermediate, and they enable the use of alkyl fluorides as the prospective alkylating reagent through C–F bond cleavage.

Mechanistic studies including observation and isolation of the anionic nickel complexes as well as kinetic studies clarified the detail of the reaction mechanism as follows: (i) anionic nickel complexes are the key intermediate at the C–C bond forming step with alkyl fluorides, and the corresponding neutral Ni complex is inert toward alkyl fluorides, (ii) the C–C and C–Ni bond forming steps are the rate-determining step in the four-component coupling and cross-coupling reactions, respectively, (iii) therefore, the selectivity of these two products is determined by the relative reactivity between the γ-carbon of the σ-allyl group and the Ni atom of the anionic nickel complexes, (iv) electron-donating groups on the aryl group accelerate the C–C and C–Ni bond formations with alkyl fluorides due to the S_N_2 type reaction mechanism in both reactions, where Ni is more effectively activated than the remote γ-carbon, (v) the Mg cation plays crucial roles in the activation of the C–F bond by its coordination, whereas other typical metal cations such as Li and Zn are not effective, and (vi) the bis(π-allyl)nickel complex prefers electron-deficient aryl Grignard reagents to form anionic Ni complexes. On the basis of the above experimental observations and DFT calculations, the catalytic cycles of both the four-component coupling and cross-coupling reactions are accomplished, in which the cation Mg^+^Br·4THF rather than Mg^+^Br plays a crucial role to activate the C–F bond. The theoretical calculations with the Mg^+^Br·4THF model not only explain very well the experimental results such as the activation energy barriers but also unravel the origins of the substituent effect (*ortho*-substituent and *para*-substituent) on the selectivity and reaction rates in terms of steric and electronic effects.

Although such anionic species of transition metals have sometimes been proposed as the key intermediates in catalytic bond formation, their structure as well as chemical characteristics are unclear in many cases. Our results demonstrate their synthetic utility as promising intermediates for C–C bond forming reactions. The present study provides useful information in organometallic and synthetic organic chemistry for developing efficient and straightforward multicomponent reactions as well as anionic transition metal complexes as unique and powerful catalytic intermediates.

## Conflicts of interest

There are no conflicts to declare.

## Supplementary Material

Supplementary information

Crystal structure data
