# Supplementary material for: Nickel-catalyzed coupling reaction of alkyl halides with aryl Grignard reagents in the presence of 1,3-butadiene: mechanistic studies of four-component coupling and competing cross-coupling reactions
Source: Chem Sci. 2018 Jan 5;9(8):2195–211. doi: 10.1039/c7sc04675h (PMC5903371; doi:10.1039/c7sc04675h)

Electronic Supplementary Material (ESI) for Chemical Science.
This journal is © The Royal Society of Chemistry 2018

Electronic Supplementary Information (ESI)

Nickel-catalyzed coupling reaction of alkyl halides with aryl Grignard reagents in the presence of 1,3-butadiene: Mechanistic studies of four-component coupling and competing cross-coupling reactions

Takanori Iwasaki,*[a] Asuka Fukuoka,[a] Wataru Yokoyama,[a] Xin Min,[a] Ichiro Hisaki,[b] Tao Yang,*[cde] Masahiro Ehara,*[cd] Hitoshi Kuniyasu,[a] and Nobuaki Kambe*[a]

[a] Department of Applied Chemistry, Graduate School of Engineering, Osaka University, Suita, Osaka 565-0871, Japan. [b] Department of Material and Life Science, Graduate School of Engineering, Osaka University, Suita, Osaka 565-0871, Japan. [c] Department of Theoretical and Computational Molecular Science, Institute for Molecular Science, 38 Nishigo-Naka, Myodaiji, Okazaki, Aichi 444-8585, Japan. [d] Elements Strategy Initiative for Catalysts and Batteries (ESICB), Kyoto University, Katsura, Kyoto 615-8510, Japan. [e] Fachbereich Chemie, Philipps-Universität Marburg, Hans-Meerwein-Strasse, Marburg 35032, Germany.

Table of Contents

Additional Results S2–S30

Materials and Methods S31

Synthesis of Substrates S32–S37

General Procedures S38–S39

Characterization Data of the Isolated Compounds S40–S64

Cartesian Coordinates of Optimized Structures S65–S161

References S162

^{1}H and ^{13}C NMR Spectra of Products S163–S228

Additional Results

1. Reaction condition screening (Table 1)

Using the optimized conditions for *ortho*-unsubstituted aryl Grignard reagents (entry 4 in Table 1), we examined various parameters to improve the selectivity for the four-component coupling product **3aa**. Selected examples are summarized in Table S1. The use of 20 mol% of Ni catalyst and PPh_3 did not change the selectivity, but slightly affected the reaction efficiency (entry 1 vs. 2). 1,1'-Bis(diphenylphosphino)ferrocene (dppf) depressed the selectivity to 0.9 (entry 3. Tetramethylethylenediamine (TMEDA) improved neither the total yield nor the selectivity (entry 4). The addition of LiI largely affected the selectivity, yielding **4aa** as the major product (entry 5). The addition of $MgBr_2$ afforded a 1:1 mixture of **3aa** and **4aa** in 61% total yield (entry 6). When the reaction was conducted in a mixed solvent of toluene and THF in 1:1 ratio, the yields of both **3aa** and **4aa** decreased to 24% and 31%, respectively, whereas the selectivity (**3aa**/**4aa**) dropped to 0.8 (entry 8). A similar tendency was observed in the mixed solvent system of hexane and THF (entry 9). On the other hand, the use of polar solvents such as dioxane and HMPA as co-solvents affected both four-component and cross-coupling reactions, resulting in a low yield (25% total yield) and no reaction, respectively.

Table S1 Additional Results of Screening of Reaction Conditions

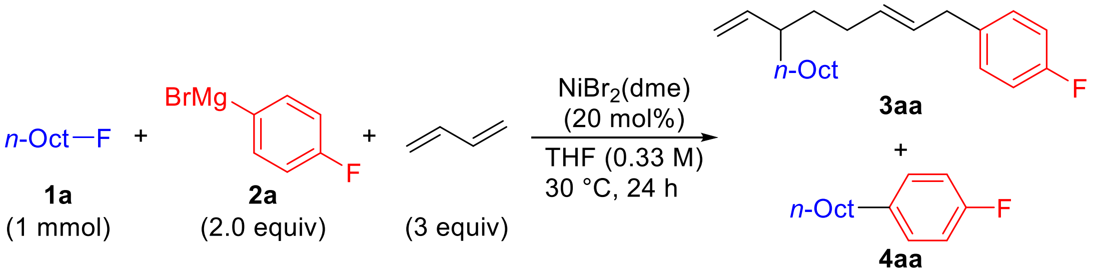

entry	variation form above conditions	**3aa** (%)[a]	**4aa** (%)[a]	**3aa**/**4aa**
1	none	57	42	1.4
2	PPh_3 (20 mol%) was added.	46	34	1.4
3	dppf (20 mol%) was added.	30	35	0.9
4	TMEDA (20 mol%) was added.	40	35	1.1
5	LiI (20 mol%) was added.	29	48	0.6
6	$MgBr_2$ (20 mol%) was added.	30	31	1.0
7	10 mol% of Ni cat.	63	37	1.7
8	10 mol% of Ni cat in toluene/THF (1:1)	24	31	0.8
9	10 mol% of Ni cat in hexane/THF (1:1)	26	32	0.8

[a]Determined by GC.

2. NMR studies of the stoichiometric reaction (eqn 3)[S1]

NMR samples were prepared as follows, and the ^{1}H NMR spectra at 20 °C are shown in Fig. 1.

Sample A (Fig. 1a): In a glove box, a 5-mm NMR tube was charged with Ni(cod)$_2$ (5.5 mg, 0.02 mmol) and THF-d_8 (0.6 mL) and sealed with a screw cap. The NMR tube was taken out of the glove box and sonicated for a few minutes.

Sample B (Fig. 1b): In a glove box, a 5-mm NMR tube was charged with Ni(cod)$_2$ (5.5 mg, 0.02 mmol) and a solution of 1,3-butadiene in THF-d_8 (0.17 M, 0.6 mL) and sealed with a screw cap. The NMR tube was taken out of the glove box and sonicated for a few minutes.

Sample C (Fig. 1c): To a 5-mm NMR tube was added 2,6-dimethylphenylmagnesium bromide solution in THF (0.83 M, 48 μL, 0.04 mmol) under nitrogen atmosphere, and the tube was sealed with a screw cap. Most of the THF was then removed by evaporation under reduced pressure through the screw cap. Ni(cod)$_2$ (5.5 mg, 0.02 mmol) and a solution of 1,3-butadiene in THF-d_8 (0.17 M, 0.6 mL) were added into the NMR tube in a glove box. After sealing with a screw cap, the NMR tube was taken out of the glove box and sonicated for a few minutes.

As shown in Fig. 1b, a set of broad signals and signals of free COD appeared upon the addition of 1,3-butadiene to Ni(cod)$_2$, in conjunction with the disappearance of signals of free 1,3-butadiene (in THF-d_8 at rt: δ 6.28 (m, 2H), 5.13 (dd, J = 16.0, 1.6 Hz, 2H), 5.01 (dd, J = 8.8, 1.6 Hz, 2H)). This is probably due to the fast equilibrium among π-complexes of Ni(0) and 1,3-butadiene (Ni(C_4H_6)$_n$) including ligand exchange with free 1,3-butadiene. The π-complexes would also be in equilibrium with Ni(cod)$_2$, but this step should be slow because the shift of the Ni(cod)$_2$ peaks is negligible. No peaks assignable to bis(π-allyl)nickel complex **A** were observed in Fig. 1b, implying that this complex is not relatively stable thermodynamically in the presence of butadiene or that the complex **A** is in fast equilibrium with a variety of possible π-complexes. The addition of an aryl Grignard reagent to the mixture of Ni(cod)$_2$ and 1,3-butadiene resulted in sharp signals (Fig. 1c). Although some signals were overlapped with the solvent, the signals at δ 6.36 (d, J = 7.2 Hz, 1H, *Ar*), 5.69 (q, J = 8.8 Hz, 1H, β-H of σ-allyl), 3.92 (q, J = 7.2 Hz, 1H, γ-H of σ-allyl), 2.52 (s, 3H, *Me*), 2.21 (s, 3H, *Me*), 2.03 (dd, J = 11.2, 4.0 Hz, 1H, α-H of σ-allyl), 1.24 (d, J = 8.4 Hz, 1H, π-allyl), 0.84 (dd, J = 7.2, 4.4 Hz, 1H, α-H of σ-allyl) could be assigned to the corresponding ate complex **B** (*vide infra*). The NMR spectra did not change at temperatures between 25 °C and –40 °C. In addition, the NMR spectrum after 24 h at rt remained the same, suggesting that the ate complex is thermally stable in THF.

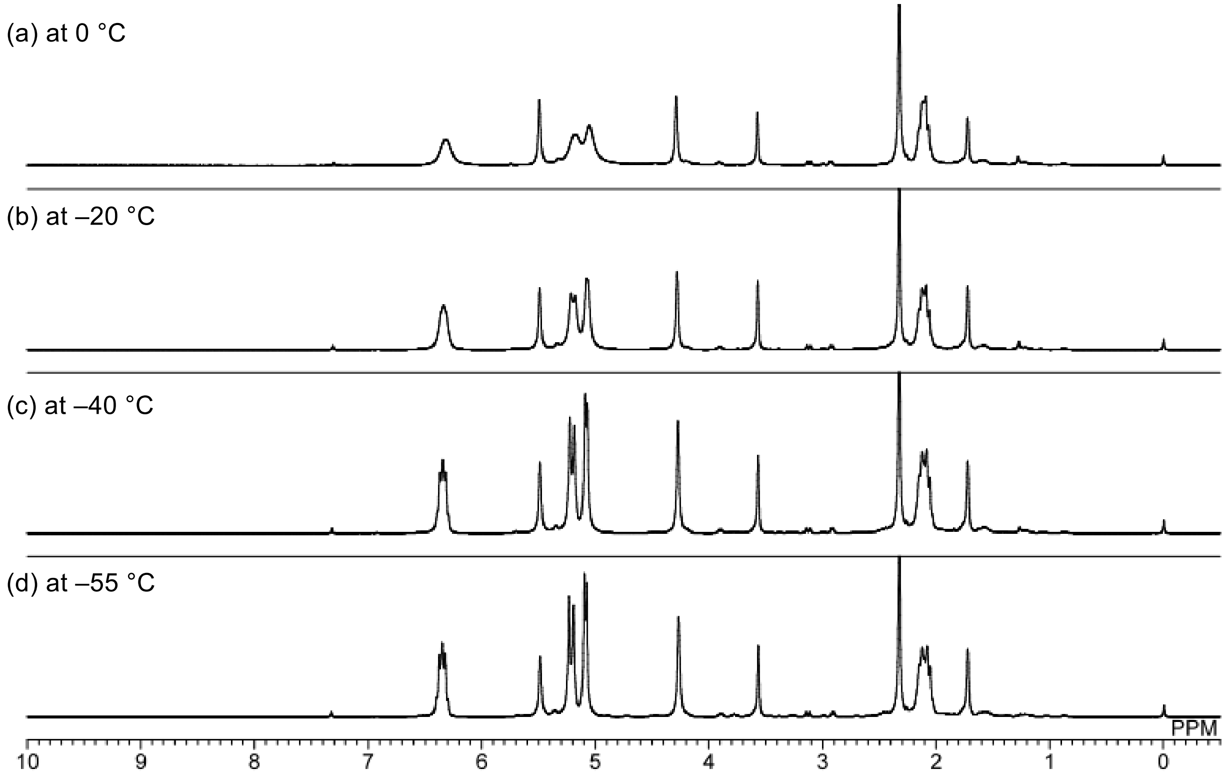

Fig. S1 VT NMR spectra of THF-d_8 solution of Ni(cod)$_2$ (0.02 mmol) and 1,3-butadiene (0.10 mmol).

In the variable temperature (VT) NMR study of the mixture of Ni(cod)$_2$ and 1,3-butadiene (Sample B), peaks assignable to free 1,3-butadiene became sharp upon cooling. The equilibrium mentioned above between Ni(cod)$_2$ and π-complexes of Ni(0) with 1,3-butadiene became slower at –55 °C in THF (Fig. S1). When ArMgBr was added to this mixture, anionic complexes were yielded, exclusively. This is in accord with the report that Ni(cod)$_2$ reacts with two molecules of 1,3-butadiene in the presence of phosphine ligands to form Ni(PR$_3$)(η^1,η^3-C_8H_{12}) which is isoelectronic to the ate complex **B**.[S2]

Possible intermediates and pathways leading to the anionic intermediate **B** from Ni(cod)$_2$ are depicted in Scheme S1. Many possible π-complexes (**I** to **VI**) may be present in rapid equilibria with each other and free 1,3-butadiene. Thus, the above-mentioned broadening of the 1,3-butadiene peaks was observed. After cooling to –55 °C, ligand exchange became slower, and peaks of free 1,3-butadiene became sharp. However, coordinated 1,3-butadiene was not observed due to the rapid equilibria among π-complexes **I** to **VI**. It is reported that Ni(0) species undergo complexation with two molecules of 1,3-butadiene to give bis(η^4-butadiene) complex **IV**, which is in equilibrium with the 16-electron complex **V** and can accept further coordination of 1,3-butadiene to form the tris(η^2-butadiene) complex **VI**.[S3] The activation free energies of oxidative dimerization of 1,3-butadiene on Ni complexes **IV** and **VI** were calculated as 16.2 and 12.6 kcal/mol, respectively.[S3] Therefore, the oxidative dimerization would proceed from complex **VI** rather than **IV**. Thus, the formed complex **VIII** reacts directly with Grignard

reagents or via dissociation of 1,3-butadiene through complex **A** (Scheme S1).

Scheme S1 Equilibrium Between Ni(0) and Anionic Ni Complexes

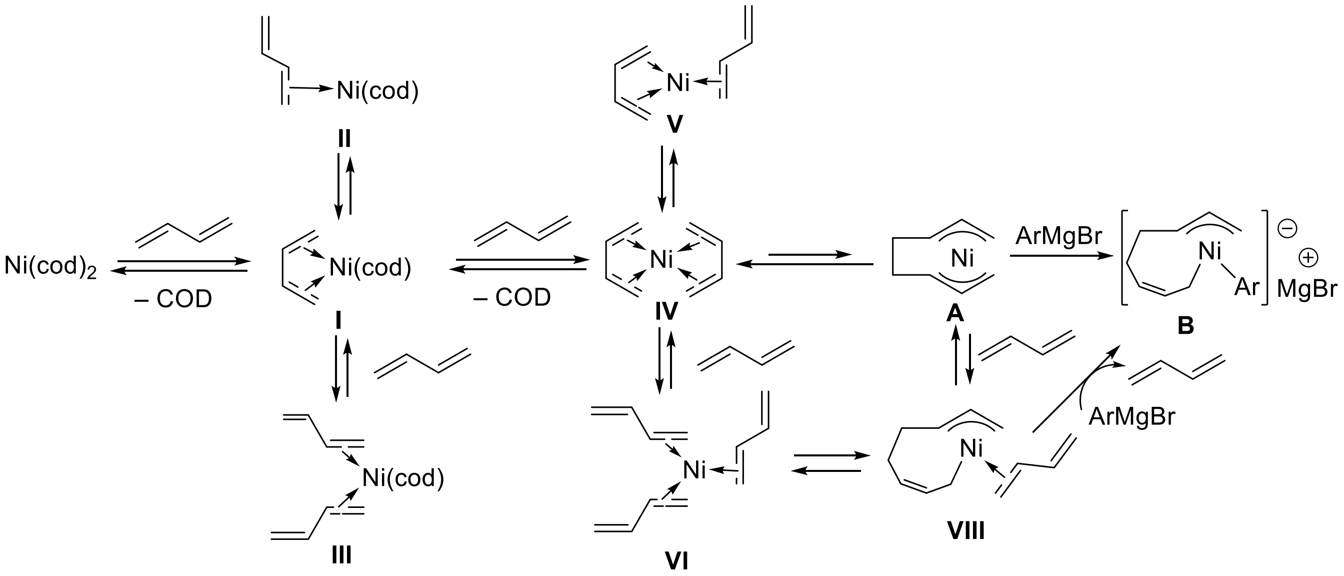

3. Isolation and reactivity of an anionic Ni complex (Scheme 5)

3-1. Synthesis of nickelate complex (7)[S1]

Into a suspension of Ni(cod)$_2$ (277 mg, 1.01 mmol) in Et_2O (5 mL) was added 2,6-dimethylphenyllithium (in Et_2O, 0.54 M, 2.8 mL, 1.51 mmol) and 1,3-butadiene (135 mL as gas, 6.0 mmol) at –78 °C. Then, the mixture was allowed to warm to rt and stirred for 1 h. The resulting orange solution was again cooled to –78 °C, slightly depressurized, and the Schlenk tube was brought into a glove box. Orange oily precipitates were formed upon adding DME (1 mL) followed by pentane (10 mL). The supernatant was removed by decantation, and the orange precipitates were washed with pentane for three times. The precipitates were dissolved in toluene (10 mL) and filtrated to remove insoluble solids. The obtained organic solution was concentrated to give an orange semisolid which was again dissolved in Et_2O. The resulting orange suspension was filtrated and concentrated in vacuo to give an orange semisolid (522 mg, 94%): ^{1}H NMR (400 MHz, THF-d_8): δ 6.35 (d, J = 6.0 Hz, 1H, H_k), 6.21 (d, J = 6.0 Hz, 1H, $H_{k'}$), 6.18 (t, J = 6.0, Hz, 1H, H_l), 5.70 (q like, J = 8.0 Hz, 1H, H_g), 3.90 (q, J = 7.2 Hz, 1H, H_f), 3.60 (td, J = 12.0, 7.2 Hz, 1H, H_b), 3.37 (s, 12H, H_o), 3.21 (s, 18H, H_n), 2.51 (s, 3H, H_m), 2.23 (s, 3H, $H_{m'}$), 2.02 (dd, J = 10.8, 3.2 Hz, 1H, H_h), 1.91–1.81 (m, 2H, H_d & H_e), 1.55 (dd, J = 7.2, 1.6 Hz, 1H, H_c), 1.54–1.46 (m, 2H, H_a & H_e), 1.23 (d, J = 12.8 Hz, 1H, H_a), 0.88 (dd, J = 7.6, 4.8 Hz, 1H, H_h), 0.70–0.60 (m, 1H, H_d): ^{13}C NMR (100 MHz, THF-d_8): δ 186.3 (s, C_i), 143.1 (s, C_j), 142.9 (s, $C_{j'}$), 137.3 (d, C_g), 121.0 (d, C_k), 120.0 (d, $C_{k'}$), 117.2 (d, C_l), 101.2 (d, C_b), 99.6 (d, C_f), 71.7 (t, C_o), 58.7 (d, C_c), 57.9 (q, C_n), 39.2 (t, C_a), 28.9 (t, C_d), 27.0 (q, C_m), 26.1 (q, $C_{m'}$), 25.2 (t, C_e), 12.3 (t, C_h). ^{1}H and ^{13}C NMR as well as COSY and HMQC spectra of complex **7** are shown in Figs. S2-S4. These data are in accord with those of similar compounds Ni(PR$_3$)(η^1,η^3-C_8H_{12})[S2], [Ni(η^3-C_3H_5)(C_4H_8)]$^-$[Li(tmeda)$_2$]$^+$,[S4] and Pd(PMe$_3$)(η^1,η^3-C_8H_{12}).[S5] However, after drying under vacuo at rt for several hours, the orange semisolid turned to black semisolid probably due to decomposition. Therefore, elemental analysis did not give a satisfactory result: Anal. Calcd for $C_{28}H_{51}O_6NiLi$: C, 61.22; H, 9.36. Found: C, 60.21; H, 8.47.

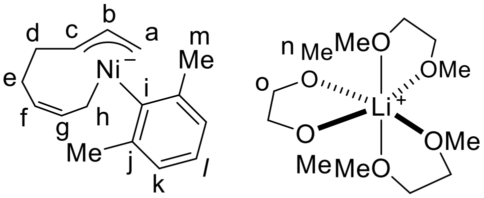

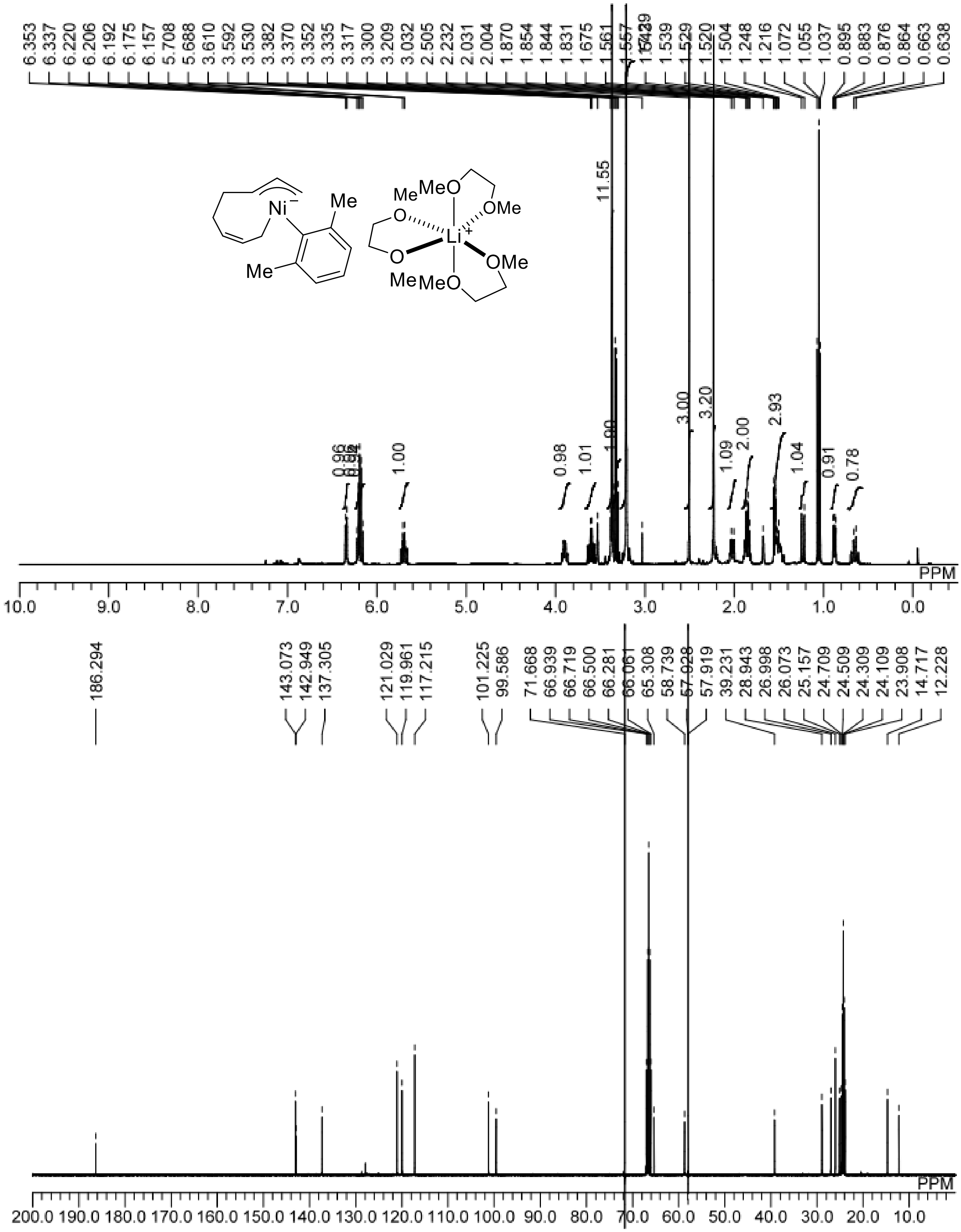

Fig. S2 ^{1}H and ^{13}C NMR spectra of Ni complex **7** in THF-d_8.

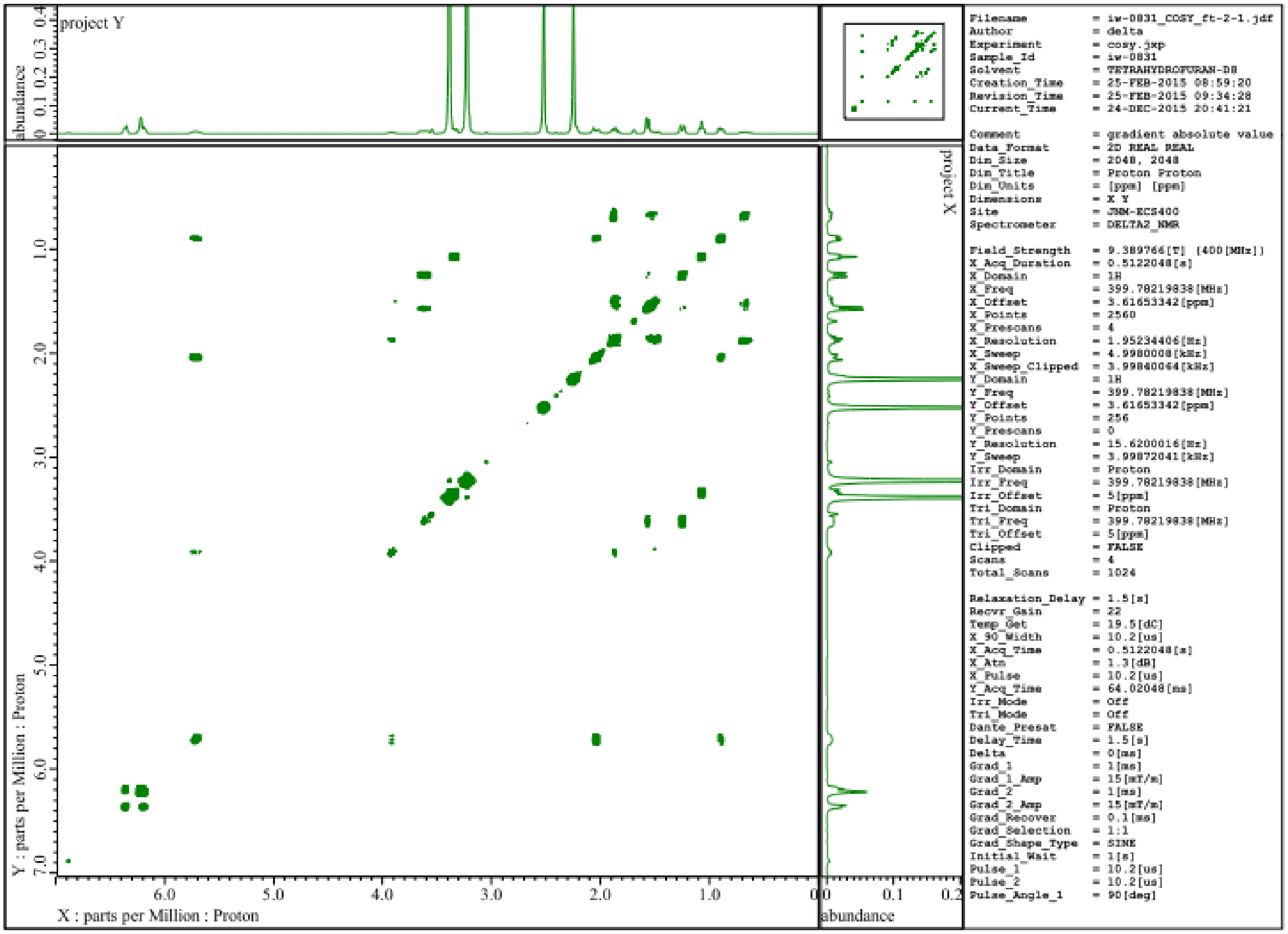

Fig. S3 COSY spectrum of Ni complex **7** in THF-d_8.

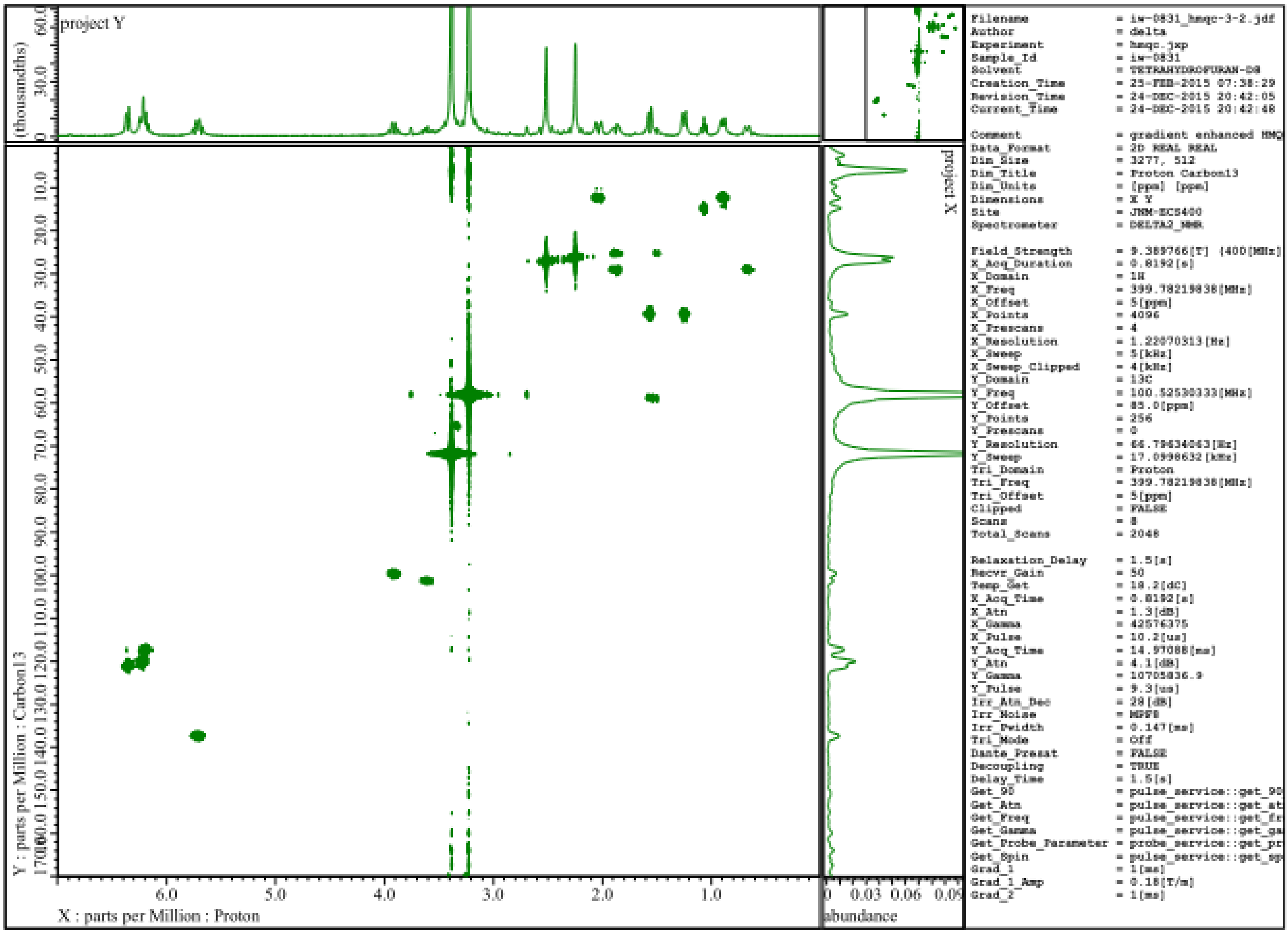

Fig. S4 HMQC spectrum of Ni complex **7** in THF-d_8.

3-2. Four-component coupling reaction using the nickelate complex 7 (eqn 4)[S1]

An oven-dried test tube was charged with the formed nickelate complex **7** (14.1 mg, 5 mol%) and a stirring bar and closed with a septum cap in a glove box. The test tube was brought out of the glove box and into it were added 1-fluorooctane (66.6 mg, 0.5 mmol), THF (0.6 mL), 2,6-dimethylphenylmagnesium bromide (in THF, 0.83 M, 0.9 mL, 0.75 mmol), and 1,3-butadiene (34 mL as gas, 1.5 mmol) by syringe at –78 ºC. The mixture was then stirred at 40 ºC. After 10 h, the reaction mixture was diluted with Et_2O, carefully quenched by 1N HCl aq., and analyzed by GC using dodecane as an internal standard to determine the yield of the desired product **3am** (88%).

3-3. The reaction of complex 11 with alkyl bromides

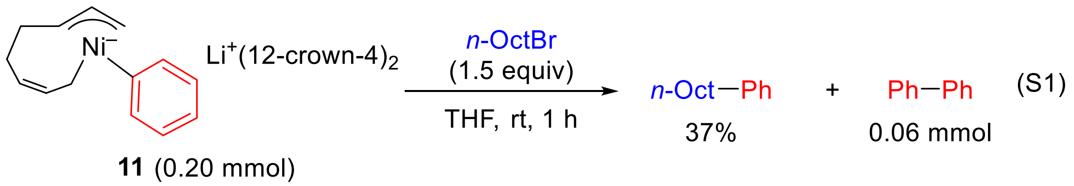

When complex **11** (119 mg, 0.20 mmol) was treated with *n*-OctBr (52 μL, 0.30 mmol) in THF at rt for 1 h, 0.23 mmol of *n*-OctBr was consumed to give the cross-coupling product, *n*-Oct-Ph, in 37% yield along with biphenyl (0.06 mmol) (eqn S1). This result shows that alkyl bromides could react with anionic Ni complex having Li as the cation in sharp contrast with the case of alkyl fluorides (Schemes 6 and 7).

4. X-ray crystallographic analysis (Fig. 2)

Single crystals of $[Ni(C_8H_9)(C_8H_{12})\cdot Li(12\text{-crown-4})_2]_4(thf)(C_5H_{12})_2$ (**8**) suitable for X-ray crystallography were obtained by recrystallization of $Ni(C_8H_9)(C_8H_{12})\cdot Li(dme)_3$ (**7**) from THF containing 12-crown-4 and pentane at –25 °C under N_2 atmosphere. The crystal was mounted on a CryoLoop (Hampton Research Corp.) with a layer of light mineral oil (dried over metallic Na at 100 °C for overnight) and placed in a nitrogen stream at 123(2) K. Measurements were made on a Rigaku RAXIS-RAPID diffractometer with a 2-D area detector using graphite-monochromated Cu-Kα radiation (λ = 1.54187 Å). The structure of the complex **8** was solved by direct methods (SHELXS97[S6]). The structure was refined on F^2 by the full-matrix least-squares method using SHELXL Version 2014/7.[S6] The non-hydrogen atoms were anisotropically refined, while the hydrogen atoms were refined using the riding model. The function being minimized was $[\Sigma w(F_o^2 - F_c^2)^2]$ ($w = 1 / [\sigma^2(F_o^2) + (0.1940P)^2 + 0.0000P]$), where $P = (\mathrm{Max}(F_o^2,0) + 2F_c^2) / 3$ for counting statistics. The functions $R1$ and $wR2$ were $(\Sigma||F_o| - |F_c|| / \Sigma|F_o|)$ and $[\Sigma(w(F_o^2 - F_c^2)^2) / \Sigma w(F_o^2)^2]^{1/2}$, respectively. The Mercury program was used to draw the molecular structure. One of the pentane molecules was disordered in two conformations with a 0.510(17):0.490(17) ratio.

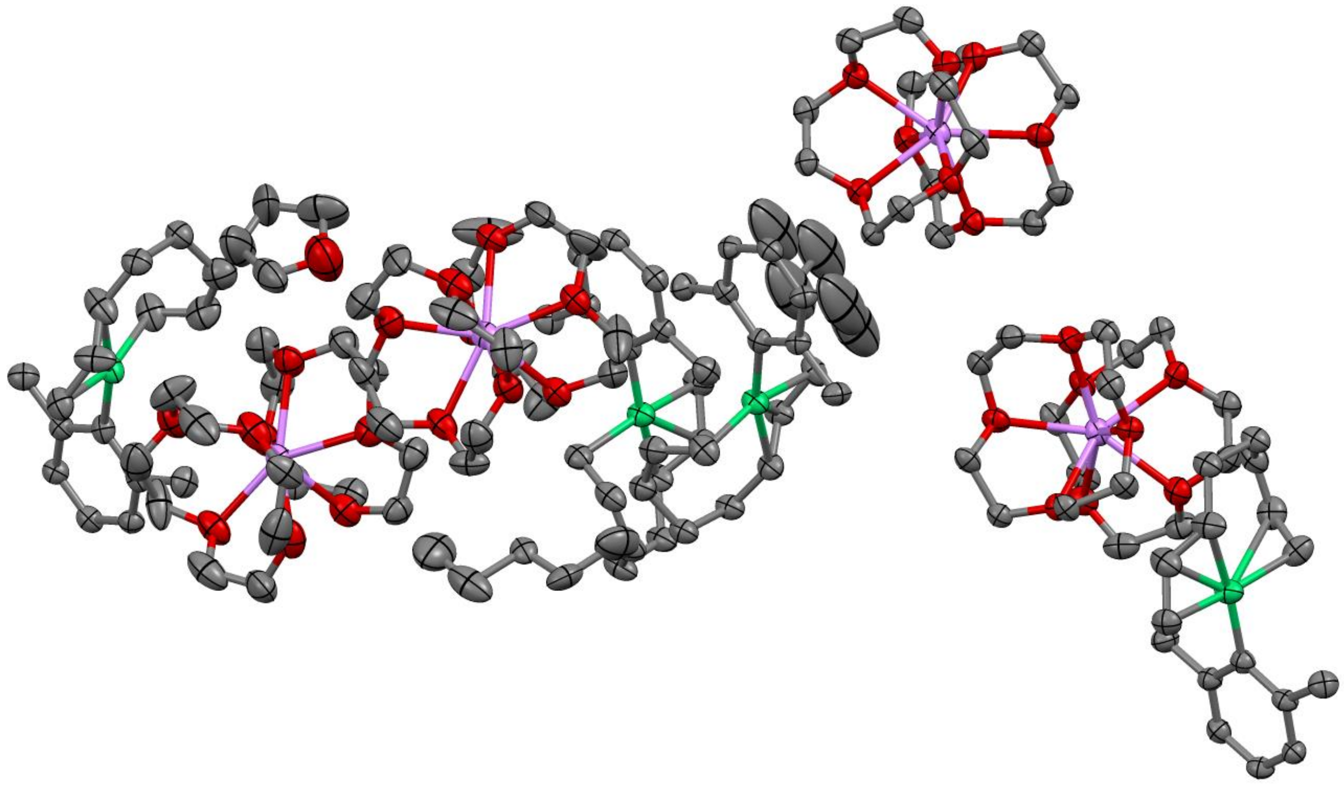

Fig. S5 ORTEP drawing of complex **8** with thermal ellipsoids at the 30% probability level. All hydrogen atoms and disordered carbons of pentane are omitted for clarity.

Table S2 Crystal Data and Data Collection Parameters of $[Ni(C_8H_9)(C_8H_{12})\cdot Li(12\text{-crown-4})_2]_4(thf)(C_5H_{12})_2$ (8)

empirical formula	$C_{142}H_{244}Li_4Ni_4O_{33}$
formula weight	2742.03
color, description	yellow, block
temperature, K	123(2)
crystal system	Triclinic
space group	P-1 (#2)
a, Å	11.4664(2)
b, Å	24.2697(4)
c, Å	28.3765(5)
α, °	67.4550(7)
β, °	88.5760(7)
γ, °	85.7540(7)
V, Å^3	7273.2(2)
Z	2
Dcalcd, g/cm^{-3}	1.252
$2\theta_{max}$, deg	143.7
limiting indices	$-14\leq h\leq 14$, $-29\leq k\leq 29$, $-34\leq l\leq 34$
absorption coefficient,	1.157
max. and min.	0.891 (max)/0.664 (min)
F(000)	3968.00
crystal size, mm	0.30 × 0.15 × 0.10
goodness-of-fit on F^2	1.052
no. of reflections measured	177793
unique data (R_{int})	27611 (0.0795)
$R1$, $wR2$ [$I > 2\sigma(I)$]	0.1017, 0.2986
$R1$, $wR2$ (all data)	0.1745, 0.3607
residual electron density, e Å^{-3}	0.99 (max), –0.68 (min)

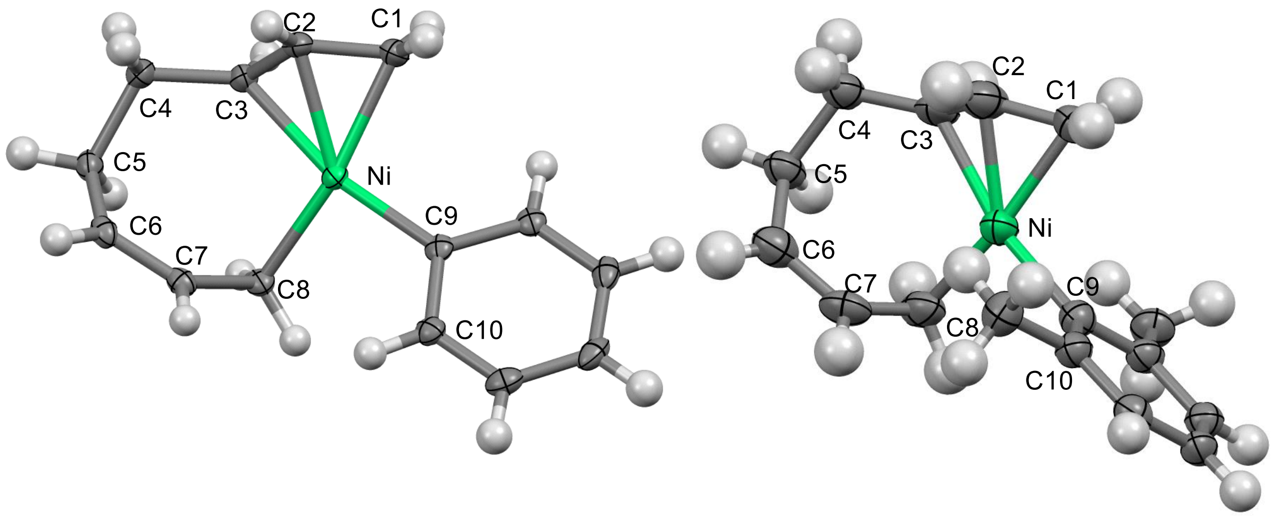

Fig. S6 ORTEP drawing of the anionic parts of complexes **11** (left)[S7] and **8** (right, one of the four asymmetric units).

Table S3 Selected Bond Lengths and Angles of Complex 11 and Four Asymmetric Units of Complex 8

bond length (Å)		**8**			
/angle (°)	**11**	Unit 1	Unit2	Unit 3	Unit 4
Ni–C1	2.012(2)	2.016(7)	2.011(7)	2.006(7)	2.017(7)
Ni–C2	2.0032(19)	1.964(9)	1.982(6)	1.979(8)	1.941(6)
Ni–C3	2.123(2)	2.121(7)	2.127(6)	2.110(6)	2.115(8)
Ni–C8	1.979(2)	2.020(7)	2.007(7)	1.982(8)	2.055(7)
Ni–C9	1.928(2)	1.912(6)	1.917(5)	1.889(6)	1.920(6)
C6–C7	1.346(3)	1.406(10)	1.365(9)	1.371(10)	1.410(12)
C1–Ni–C3	73.02(9)	74.1(3)	73.7(3)	73.4(3)	74.3(3)
C3–Ni–C8	93.64(9)	93.2(3)	94.3(3)	94.8(3)	91.4(3)
C8–Ni–C9	95.76(9)	95.9(3)	95.2(2)	95.2(3)	96.9(3)
C9–Ni–C1	96.48(9)	95.7(3)	95.9(3)	95.6(3)	96.1(3)
SUM[a]	358.9	358.9	359.1	359.0	358.7
Torsion angle of C8-Ni-C9-C10	42.51(13)	106.7(4)	97.7(5)	99.9(4)	104.8(5)

[a]Sum of angles around Ni atom.

5. Kinetic studies

5-1. Determination of rate law (Fig. 3)

Plots of concentration of product **3aj** against reaction time under the various initial concentrations of catalyst and substrates (Standard conditions: *n*-OctF = 0.33 M, *o*-TolMgBr = 0.50 M, $NiBr_2$(dme) = 0.017 M, and 1,3-butadiene = 1.0 M (eqn S2)) are shown in Figs. S7-10, where a short induction period was observed. This is probably due to the reduction of Ni salt by Grignard reagent. Therefore, the fitting lines do not cross the origin. Reaction rates were estimated from the slope of the plots at 300 to 1800 seconds. The obtained reaction rates are summarized in Tables S4-7. The double logarithm plots of obtained data are shown in Fig. 3.

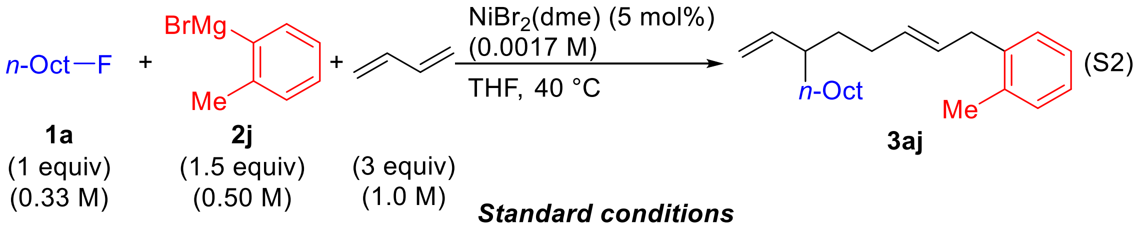

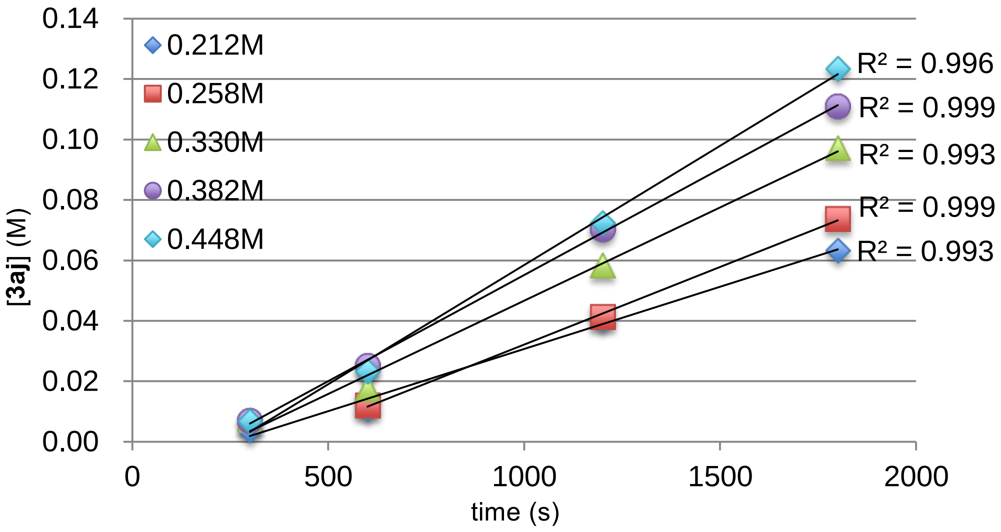

Fig. S7 Plot of product concentration against reaction time using *n*-OctF at 0.212 to 0.448 M.

Table S4 Kinetics on *n*-OctF (1a)

entry	initial concentration of *n*-OctF [M]	reaction rate [10^{-5} M/s]
1	0.212	4.12 ± 0.25
2	0.258	5.13 ± 0.15
3	0.330	6.17 ± 0.37
4	0.382	7.03 ± 0.15
5	0.448	7.89 ± 0.35

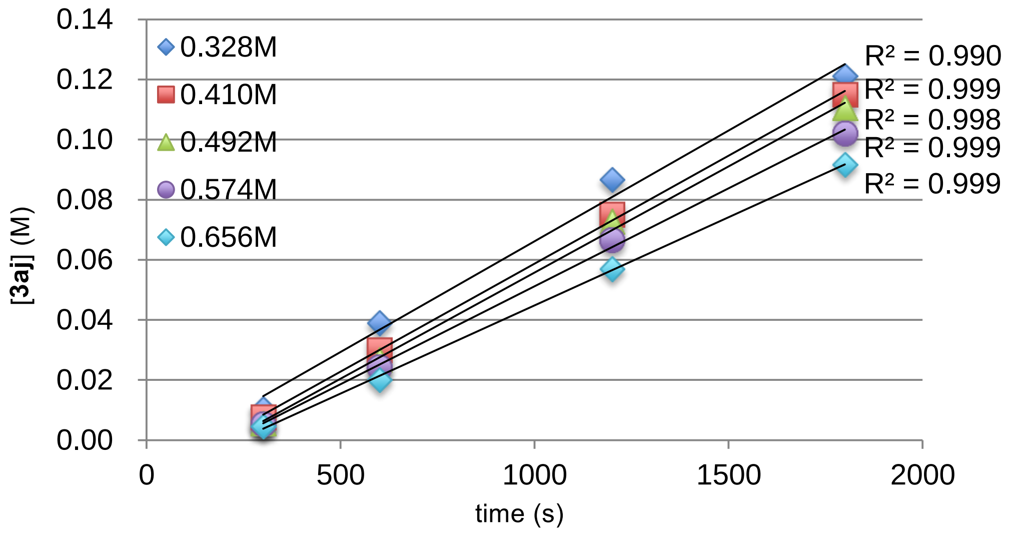

Fig. S8 Plot of product concentration against reaction time using *o*-TolMgBr at 0.328 to 0.656 M.

Table S5 Kinetics on *o*-TolMgBr (2j)

entry	initial concentration of *o*-TolMgBr [M]	reaction rate [10^{-5} M/s]
1	0.328	7.37 ± 0.52
2	0.410	7.18 ± 0.16
3	0.492	7.06 ± 0.21
4	0.574	6.51 ± 0.17
5	0.656	5.86 ± 0.09

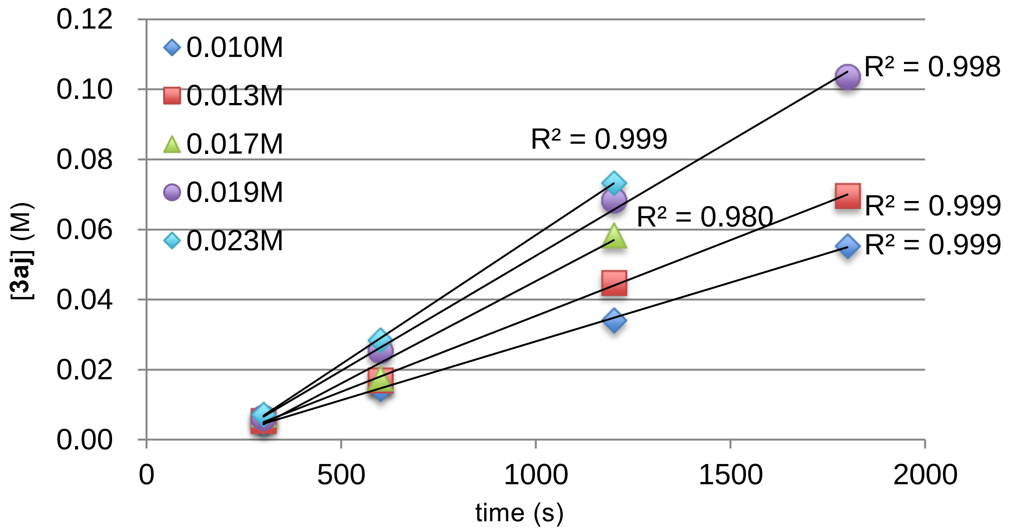

Fig. S9 Plot of product concentration against reaction time using $NiBr_2$(dme) at 0.010 to 0.023 M.

Table S6 Kinetics on $NiBr_2$(dme)

entry	initial concentration of $NiBr_2$(dme) [M]	reaction rate [10^{-5} M/s]
1	0.010	3.36 ± 0.05
2	0.013	4.33 ± 0.08
3	0.017	5.84 ± 0.37
4	0.019	6.56 ± 0.19
5	0.023	6.98 ± 0.19

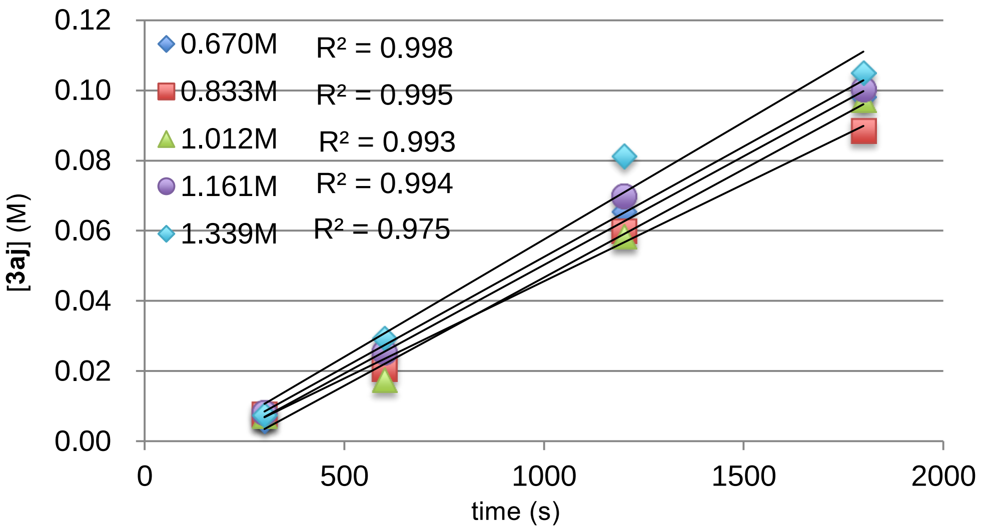

Fig. S10. Plot of product concentration against reaction time using 1,3-butadiene at 0.670 to 1.339 M.

Table S7 Kinetics on 1,3-Butadiene

entry	initial concentration of 1,3-butadiene [M]	reaction rate [10^{-5} M/s]
1	0.670	6.19 ± 0.21
2	0.833	5.54 ± 0.28
3	1.012	6.17 ± 0.37
4	1.161	6.29 ± 0.35
5	1.339	6.69 ± 0.77

5-2. Activation parameters (Fig. 4)

The reactions using **2j** and **2m** were conducted between 30 °C and 50 °C, and time-course of the reactions was traced as shown in Figs. 4a and 4b. Under the conditions with a constant amount of Ni catalyst, the reaction rate obeys pseudo-first-order kinetics for **1a** with $v = k$[**1a**][$NiBr_2$(dme)] = k_{obs}[**1a**]. The rate constants k_{obs} for Grignard reagents **2j** and **2m** were estimated by plotting ln([**1a**]/[**1a**]$_0$) against reaction time as shown in Figs. S11 and S12.

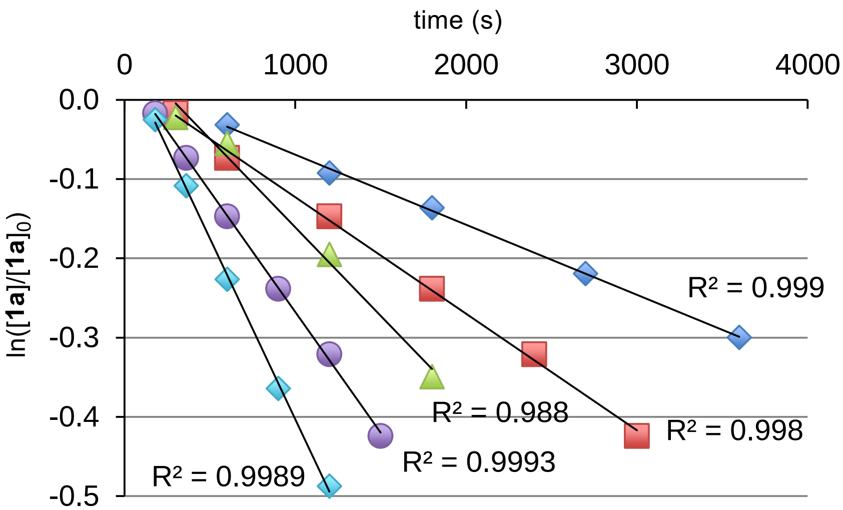

Fig. S11 (a) Time-course of the reaction of **1a** with **2j** at 30 °C (◆), 35 °C (■), 40 °C (▲), 45 °C (●), and 50 °C (◆).

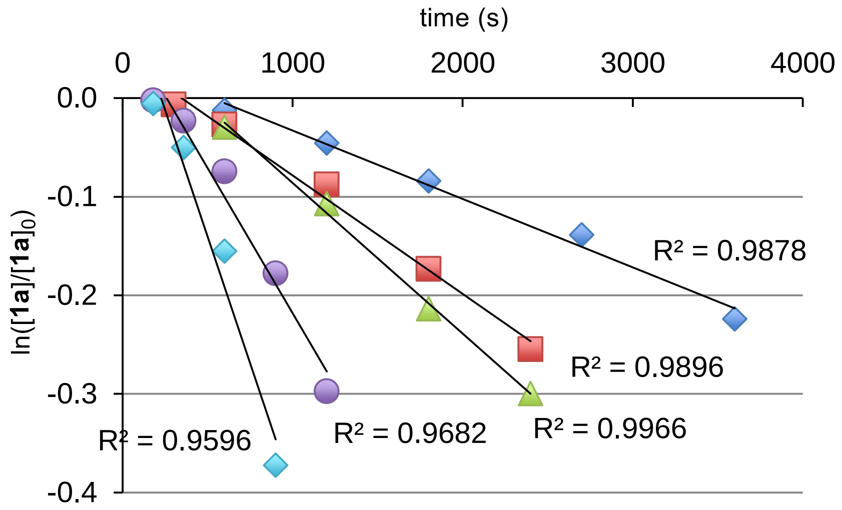

Fig. S12 Time-course of the reaction of **1a** with **2m** at 30 °C (◆), 35 °C (■), 40 °C (▲), 45 °C (●), and 50 °C (◆).

From the slope of these plots, k_{obs} at each reaction temperatures were determined, and the k (= k_{obs}[$NiBr_2$(dme)]) are summarized in Table S8. Eyring plot of these data are shown in Fig. 4c.

Table S8 Kinetic Parameters of the Four-component Coupling Reaction using Grignard Reagents 2j and 2m at Different Temperatures

	k_{obs} (10^{-4} s^{-1})		k (10^{-3} s^{-1})	
temp (°C)	**2j**	**2m**	**2j**	**2m**
30	0.885 ± 0.016	0.695 ± 0.010	5.28 ± 0.10	3.99 ± 0.06
35	1.47 ± 0.03	1.20 ± 0.10	8.90 ± 0.20	7.08 ± 0.60
40	2.23 ± 0.17	1.53 ± 0.06	13.5 ± 1.1	9.37 ± 0.39
45	3.25 ± 0.06	2.94 ± 0.36	19.3 ± 0.4	17.5 ± 2.1
50	4.56 ± 0.04	5.14 ± 0.75	27.3 ± 0.3	31.1 ± 4.5

5-3. Hammett plot regarding RDS (Section 3.6)

The reaction of *n*-OctF (**1a**) with aryl Grignard reagents having a methyl group at the *ortho*-position and various substituents at the *para*-position was conducted, and the obtained results are shown in Table S9.

Table S9 Time-course of the Four-component Coupling Reaction using Various Aryl Grignard Reagents

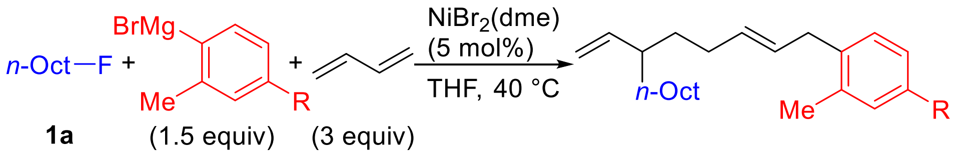

R = H (**2j**), F (**2o**), Cl (**2p**), MeO (**2r**), Me (**2q**)

	concentration of product **3** (M)				
R/time (s)	300	600	1200	1800	2400
H	0.0073	0.0176	0.0584	0.0973	0.1197
Cl	0.0018	0.0067	0.0221	0.0368	0.0533
F	0.0030	0.0122	0.0386	0.0594	0.0774
OMe	0.0113	0.0355	0.0765	0.1018	0.1281
Me	0.0130	0.0507	0.1019	0.1374	0.1617

$[\mathbf{1a}]_0$ and $[NiBr_2(dme)]_0$ = 0.335 M and 1.65 × 10^{-2} M (R = H), 0.335 M and 1.65 × 10^{-2} M (R = F), 0.338 M and 1.69 × 10^{-2} M (R = Cl), 0.335 M and 1.65 × 10^{-2} M (R = OMe), and 0.335 M and 1.65 × 10^{-2} M (R= Me).

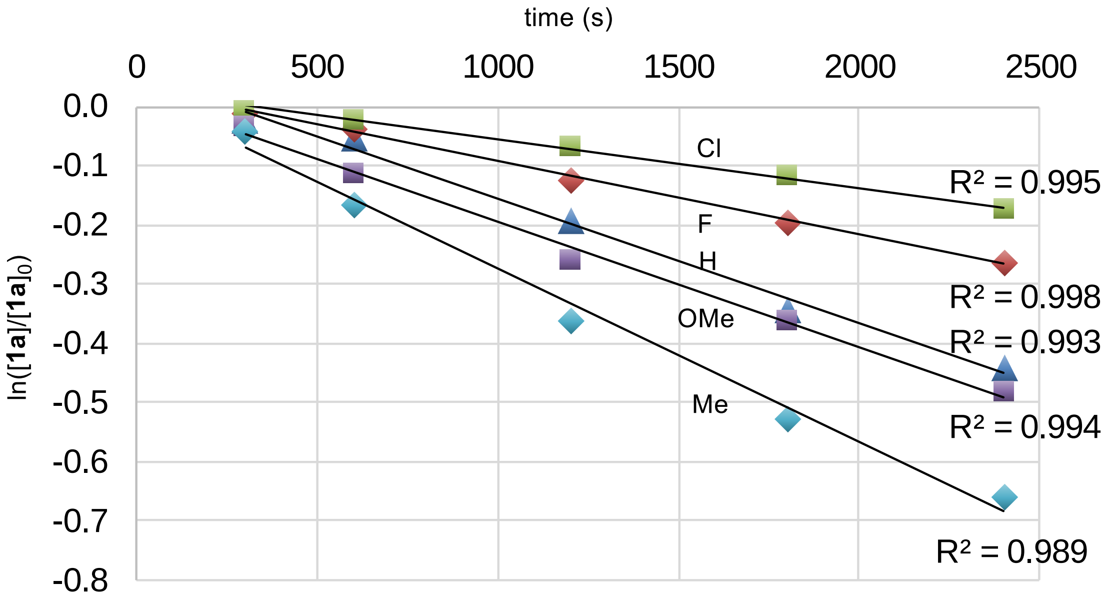

Fig. S13 Fitting the results to the pseudo-first-order line.

Under conditions using excess amounts of Grignard reagents and a constant amount of Ni catalyst, the reaction rate obeys pseudo-first-order kinetics for **1a** with $v = k$[**1a**][$NiBr_2$(dme)] = k_{obs}[**1a**]. Fig. S13 shows plots of ln([**1a**]/[**1a**]$_0$) against reaction time. From the slope of the plots, k_{obs} was obtained. The rate parameters and Yukawa-Tsuno's σ^0 are summarized in Table S10. Hammett plot of these data is shown in Fig. 5.

Table S10 Rate Constants and Hammett Constant σ^0

R	H	Cl	F	OMe	Me
k_{obs} (10^{-4} M/s)	2.10 ± 0.10	0.809 ± 0.033	1.23 ± 0.03	2.11 ± 0.09	2.94 ± 0.18
k (10^{-3} s^{-1})	12.7 ± 0.6	4.80 ± 0.20	7.47 ± 0.19	12.8 ± 0.5	17.8 ± 1.1
log(k_X/k_H)	0	–0.423	–0.232	+0.001	+0.146
σ^0	0	+0.281	+0.212	–0.100	–0.124

5-4. Competitive reactions (Section 3.8)

The competitive reactions shown in Table 7 were conducted, and the amounts of the product formed are plotted against reaction time as shown in Fig. S14, where the competitive reaction of *o*-TolMgBr (**2j**) and 4-fluoro-2-methylphenylmagnesium bromide (**2o**) was difficult to analyze by GC. Therefore, the relative reactivity of **2o** against **2j** was estimated by the competitive reaction with **2q** (Fig. S14b). The ratio of reaction constants, k_X/k_H, was directly calculated from the slope of the plots, and the obtained data are summarized in Table 7. Among substrate constants tested, σ_p^+ showed the best linearity. Hammett plot of the data against σ_p^+ is shown in Fig. 9.

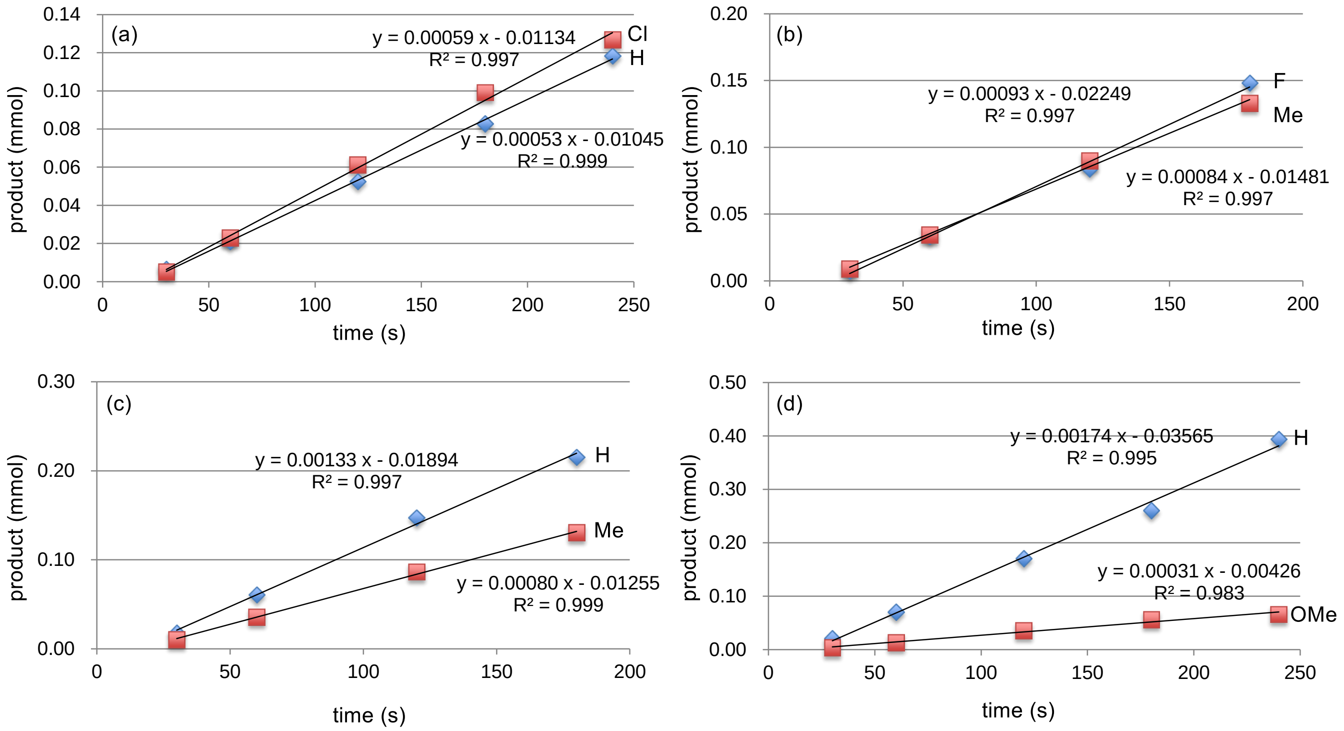

Fig. S14 Competitive reactions using two Grignard reagents. Time-course of the competitive reactions of **2j** and **2p** (a), **2o** and **2q** (b), **2j** and **2q** (c), and **2j** and **2r** (d).

6. Computational studies

6-1. Optimized structures

The optimized structures of the intermediates, transition states, and products in Fig. 10 are summarized in Fig. S15. For those of **TS3** and **TS10**, see Fig. 11.

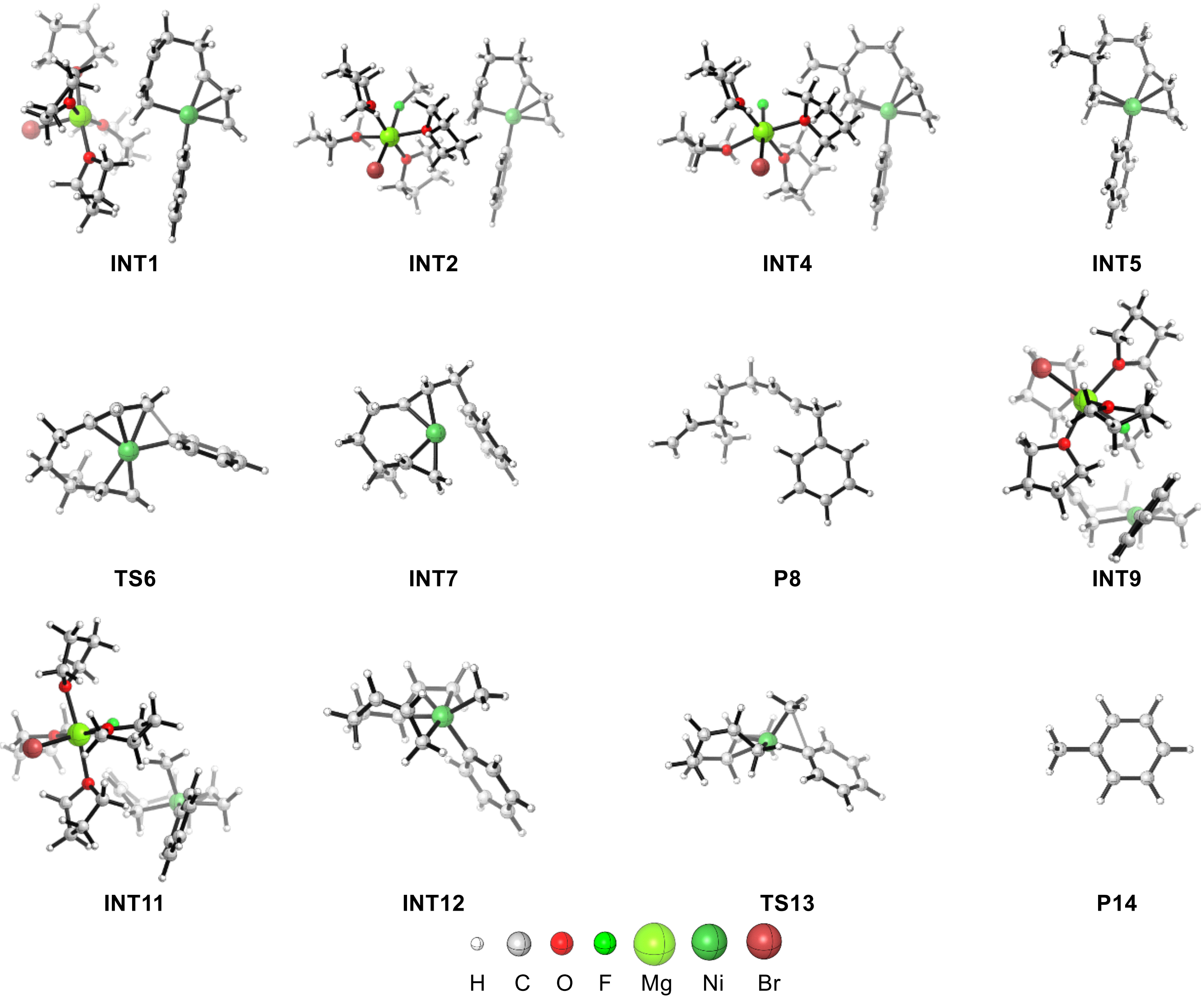

Fig. S15 Optimized structures of the intermediates, transition states, and products in Fig. 10.

6-2. Optimized structure and dihedral angle of INT1 with different aryl groups

The structures of the anionic part of **INT1** and their dihedral angles between the aryl and the coordination plane of the Ni are shown in Fig. S16. In the case of **INT1-2,6-dimethylphenyl**, the 2,6-dimethylphenyl group is orthogonally bound to Ni, in good agreement with the X-ray structure. In the case of **INT1-Ph**, the dihedral angle of the optimized structure is 118.2°, in sharp contrast to the crystal structure.[S7] NMR studies of the nickelate complexes bearing Ph and 2,6-diemthylphenyl groups at –20 °C show symmetric Ph group and unsymmetric 2,6-dimethylphenyl group (*vide supra*). These results indicate that the phenyl group easily rotates even at low temperatures. However, when methyl groups are introduced into the *ortho*-positions, the rotation becomes slow or restrained due to the steric interaction.

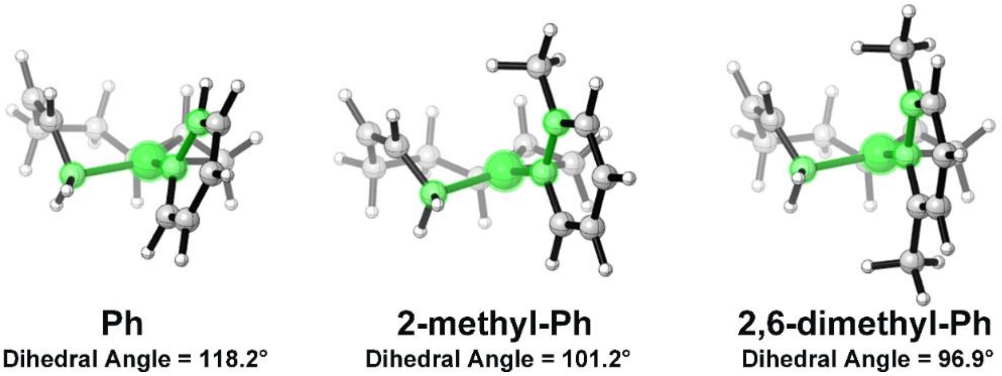

Fig. S16 Geometry of nickelate complexes having Ph (left), 2-methylphenyl (middle), and 2,6-dimethylphenyl (right) groups.

6-3. Rotation of aryl group in anionic Ni complexes

As mentioned above, ^{1}H NMR of isolated anionic Ni complexes **11** (Ph) and **8** (2,6-dimethylphenyl) showed symmetric Ph and unsymmetric 2,6-dimethylphenyl groups at –20 °C, respectively. These observations clearly indicate that the rotation of aryl ring is restrained by *ortho*-substituent(s). To evaluate rotation barrier of the anionic Ni complex having 2-methylphenyl group, we conducted theoretical calculation with the dihedral angle restriction. The initial structure with the dihedral angle of 98.6° is obtained directly from **INT1** structure by removing MgBr·4THF. Plot of relative energy against dihedral angle is shown in Fig. S17, revealing rotation barrier to be ca. 55 kJ/mol.

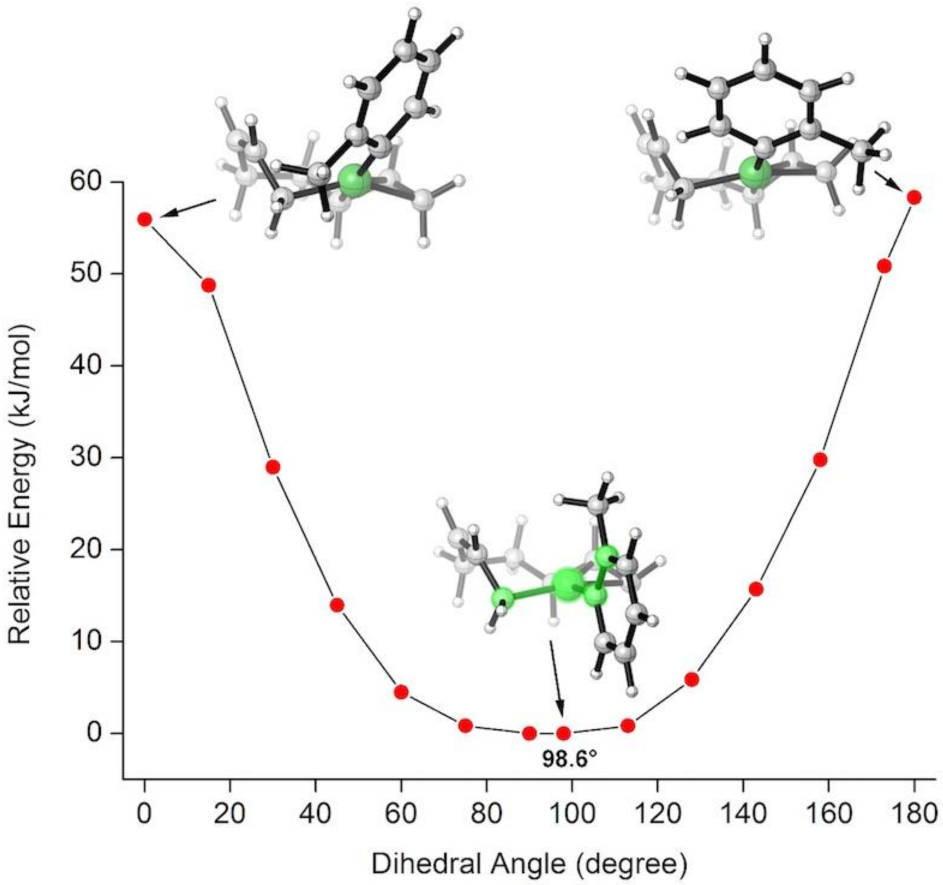

Fig. S17 Rotation barrier of anionic Ni complex having 2-methylphenyl moiety.

To confirm the rotation barrier, we further investigated by VT NMR. The anionic Ni complex having 2-methylphenyl group was synthesized by the reaction of $Ni(cod)_2$ with 2-methylphenyllithium reagent in the same way as the case of complexes **7** and **11**. ^{1}H NMR spectrum of the isolated complex showed two signals arising from *ortho*-methyl group at 4.99 and 4.47 ppm at –20 °C in THF-d_8.

Rotation rate was estimated by line-shape analysis of the ^{1}H NMR spectra recorded at –60 to –20 °C using gNMR simulation software[S8]. The obtained rate constants are summarized in Table S11. Eyring

plot of the obtained data is shown in Fig. S18. From the slope and intercept, $\Delta H^{\ddagger}$ and $\Delta S^{\ddagger}$ are determined to be 24.2 ± 2.8 kJ/mol and –104 ± 12 J/Kmol, respectively. Therefore, the rotation barrier $\Delta G^{\ddagger}_{273}$ is 52.7 ± 6.0 kJ/mol, the value which is consistent with the calculated value of 55 kJ/mol.

Table S11 Rate Constants of the Rotation

Temp. (K)	213	223	233	243	253
k (s^{-1})	16.66	34.88	86.12	118.3	162.6

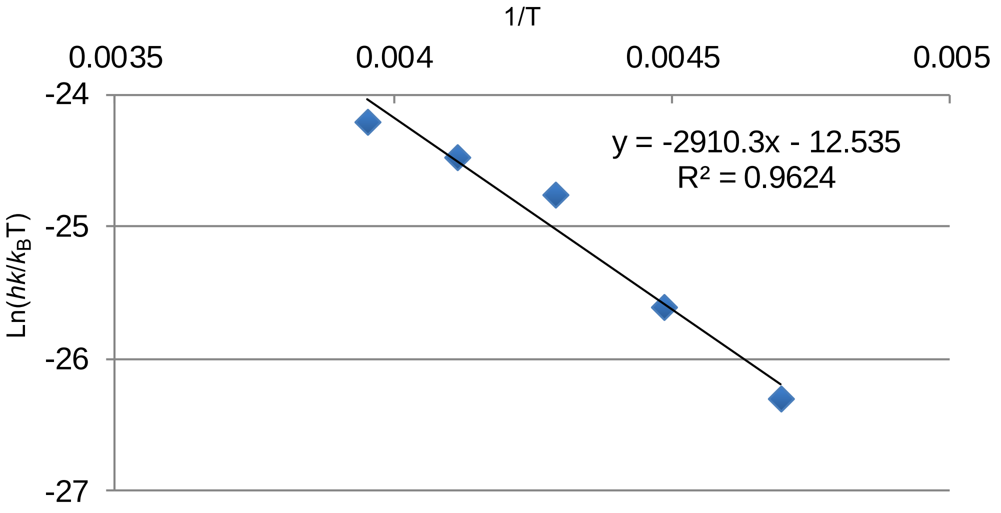

Fig. S18 Eyring plot of the rotation.

6-4. Geometry of Ni complexes bearing 2-methylphenyl group

Because of the orthogonal orientation of the aryl group, Ni complex bearing 2-methylphenyl group has two possible geometries, in which the σ-allyl group and methyl group on the 2-methylphenyl group point in either the same (up) or opposite (down) directions. We thus calculated **TS3** and **TS10** with *n*-Pr-F for both geometries at M06/6-31G(d,p) level of theory, and the results are summarized in Fig. S19. In **TS3**, **TS3-2-methyl-up** is more favorable compared to **TS3-2-methyl-down**. In contrast, in **TS10**, **TS10-2-methyl-down** is favored rather than **TS10-2-methyl-down** due to the steric repulsion between *ortho*-methyl group and *n*-Pr moiety. It should be noted that **TS10-2-methyl-down** is the most favorable in energy, being inconsistent with the experimental results. In both **TS10-2-methyl-up** and **TS10-2-methyl-down**, a short C-H-π interaction (ca. 2.5 Å) is found between THF and aryl ring, presumably contributing stabilization of these TSs. To avoid the C-H-π interaction, we chosen a model containing the additional THF molecule between cationic moiety and the 2-methylphenyl ring as shown in Fig. S20. The addition of the THF molecule suppresses the short C-H-π interaction and gives reasonable structure of TSs, where **TS3-2-methyl-up·THF** is the most energetically favorable in four

possible TSs.

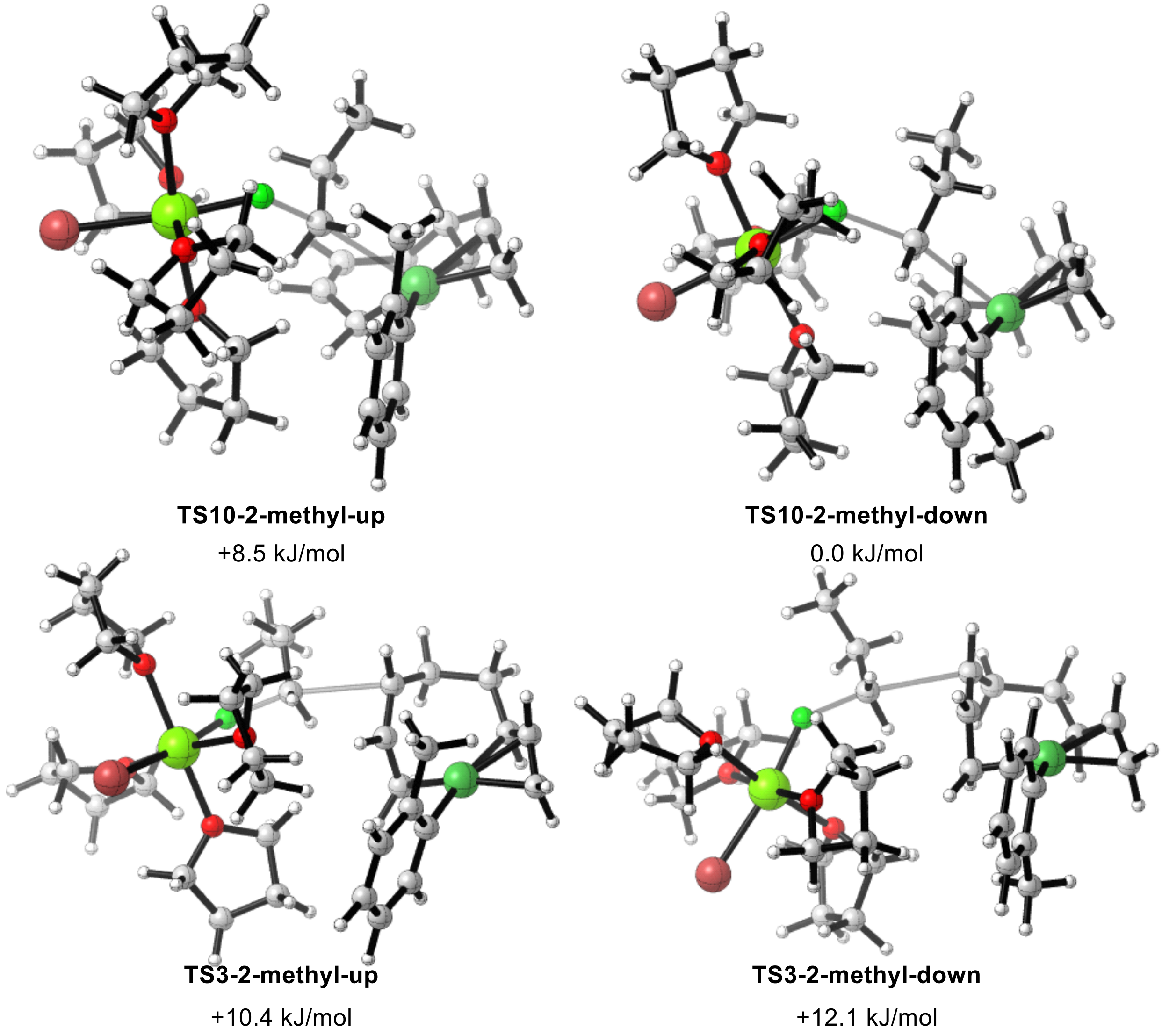

Fig. S19 Optimized geometries and corresponding relative energies of **TSS** and **TS10** with up- and down-configurations at M06/6-31G(d,p) level of theory.

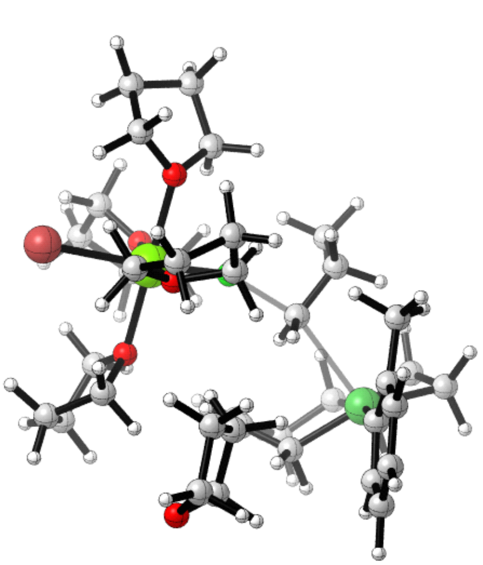

TS10-2-methyl-up·THF

+33.1 kJ/mol

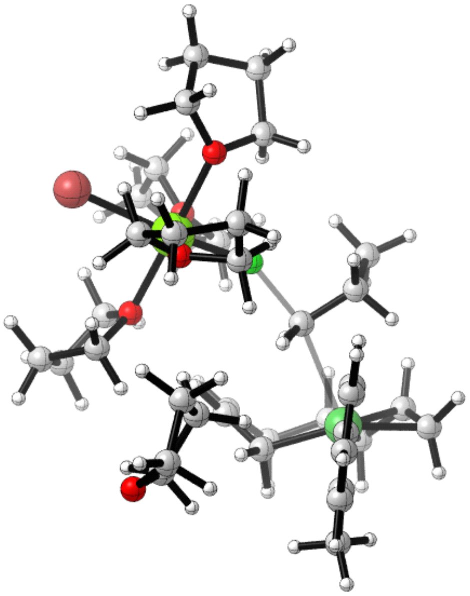

TS10-2-methyl-down·THF

+14.4 kJ/mol

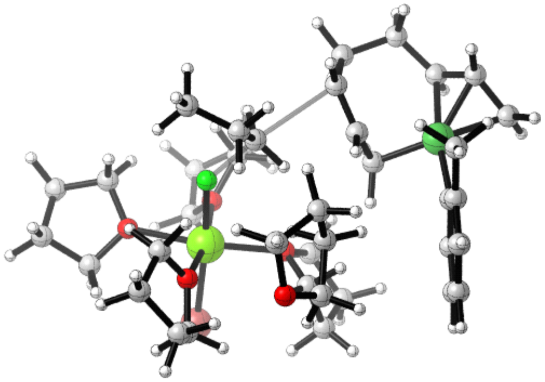

TS3-2-methyl-up·THF

0.0 kJ/mol

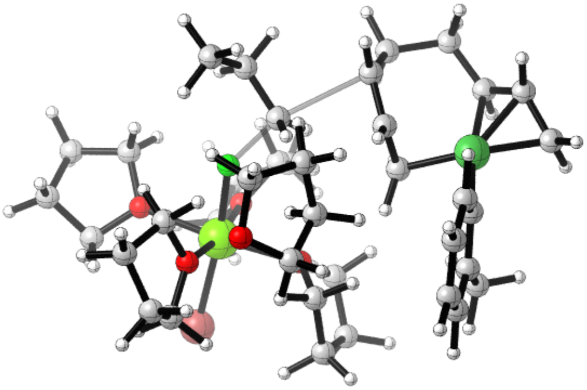

TS3-2-methyl-down·THF

+2.5 kJ/mol

Fig. S20 Optimized geometries and corresponding relative energies of **TS3** and **TS10** containing the additional THF molecule between *o*-Tol group and the cation with up- and down-configurations at M06/6-31G(d,p) level of theory.

6-5. Structure differences in TS10

Table S12 summarizes important bond distances in **TS10** with various Grignard reagents and alkyl fluorides, e.g. in **TS10** with Ph and MeF, the C–F bond is 1.28 times longer than MeF (C–F = 1.379 Å), and the C–Ni bond is 1.28 times longer than **INT11** (C–Ni = 1.914 Å). The C–F and C–Ni distances in **TS10** with Ph and *n*-OctF are longer than those in **TS10** with MeF, where both bonds are 1.36 times and 1.35 times elongated, respectively. Although this step is the nucleophilic substitution of alkyl fluorides with anionic Ni center by the aid of the Mg cation, the details of the reaction mechanism of this step differs according to alkyl fluorides. In the case of MeF, the reaction proceeds through pure S_N2 mechanism. On the other hand, the process of *n*-OctF proceeds through S_N2 mechanism with a somewhat S_N1 character. This difference may arise from the stability of the carbocation, where methyl cation is unfavorable than *n*-Oct cation. Therefore, the C–F and C–Ni distances of **TS10** with MeF are shorter than that of **TS10** with *n*-OctF.

When methyl group(s) was introduced into the *ortho*-positions, both C–F and C–Ni bonds are elongated probably due to the steric repulsion, and the elongation of these bond distances is more significant in the case of *n*-OctF. For instance, the change of the sum of C–F and C–Ni bonds is 0.112 Å for *n*-Oct but only 0.051 Å for MeF by changing Ph to 2,6-dimethylphenyl. This result could be explained by the stability of the carbocation as mentioned above. In the case of MeF, the corresponding Me cation is relatively unstable and therefore elongation of C–F and C–Ni bond in **TS10** is impermissible, resulting in the increase of the energy barrier by steric repulsions. On the other hand, the steric repulsion is loosened by elongation of these bonds in the case of **TS10** with *n*-OctF. Therefore, these TSs with *n*-OctF are not sensitive to steric hindrances compared to TSs with MeF.

Table S12 Important distances (in Å) and energy barrier (in kJ/mol) in TS10 with three different Grignard reagents and two alky fluorides

alky fluoride	MeF				*n*-OctF			
Grignard reagent	C–F	C–Ni	*sum*[a]	$\Delta G^{\ddagger}$	C–F	C–Ni	*sum*[a]	$\Delta G^{\ddagger}$
Ph	1.763	2.457	4.220	65.8	1.882	2.590	4.472	80.2
2-methylphenyl	1.771	2.461	4.232	116.3	1.920	2.602	4.522	106.9
2,6-dimethylphenyl	1.785	2.486	4.271	134.2	1.936	2.648	4.584	117.5

[a]Sum of C–F and C–Ni bonds

NBO analysis of **TS10** with PhMgBr and MeF or *n*-OctF is shown in Table S13. Among key atoms in the bond cleavage and formation, the NBO charge of the reacting carbon is drastically altered by changing MeF to *n*-OctF (from –0.137 to +0.101). This large difference in NBO charge clearly supports the above mentioned difference in mechanism and the S_N1 character of **TS10** with *n*-OctF. The relatively

small difference of Ni in NBO charge is due to the π-allyl group in *trans*-position (*vide infra*).

Table 13 NBO atomic charges (|e|) of TS10-Ph with Me-F and *n*-Oct-F

Alkyl fluoride	Mg	F	C	Ni
MeF	+1.791	-0.679	**-0.137**	+0.219
n-OctF	+1.799	-0.747	**+0.101**	+0.231

6-6. NBO charge of nickelate complexes

NBO charges of nickelate complexes having Me on the *ortho*-position and various substituents on the *para*-position of Ar are summarized in Table S14. It should be noted that electronic effects of the substituents affect the NBO charge of the γ-carbon in the σ-allyl group. However, that of Ni atom is constant in all cases. This is probably due to the electronic transfer between Ni and the π-allyl moiety at the *trans*-position. Indeed, the total NBO charge of Ni and the π-allyl depends on the substituents.

Table S14. Corresponding Charges (|e|) of γ-Carbon of σ-Allyl, π-Allyl, and Ni Center, and the Sum of Charges on Allyl Carbons C1, C2, and C3 coordinated to Ni center

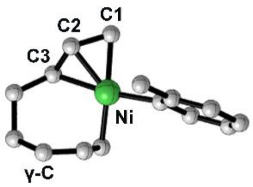

R	H	F	Cl	Me	OMe	NMe_2
**γ-C**	-0.388	-0.387	-0.376	-0.390	-0.388	-0.390
**Ni**	0.233	0.232	0.233	0.233	0.233	0.232
**π-allyl**[a]	-1.051	-1.047	-1.048	-1.053	-1.052	-1.053
**sum of Ni and π-allyl**	-0.818	-0.815	-0.815	-0.820	-0.819	-0.820

[a]Sum of NBO charges on C1, C2, and C3.

Materials and Methods

Nuclear magnetic resonance (^{1}H NMR, ^{13}C NMR, and ^{19}F NMR) spectra were recorded on a JEOL JNM-Alice 400 spectrometer operating at 400 MHz (^{1}H NMR), 100 MHz (^{13}C NMR) and 376 MHz (^{19}F NMR) in 5-mm NMR tubes. All ^{1}H NMR chemical shifts were reported in ppm relative to an internal reference tetramethylsilane at δ 0.00 or residual protonated solvent (THF-d_7 at δ 3.53). All ^{13}C NMR chemical shifts were reported in ppm relative to the carbon of chloroform-d_1 as δ 77.00 and THF-d_8 as δ 66.50. The ^{19}F NMR chemical shifts were reported in ppm relative to external references of α,α,α-trifluorotoluene as δ –63.90. HPLC separations were performed on a recycling preparative HPLC (Shimadzu Prominence) equipped with Shodex K2001 and 2002 columns (GPC) using $CHCl_3$ as an eluent. GC analyses were performed on a Shimadzu GC-2014 instrument equipped with a GL Sciences InertCap 5 capillary column (I.D. 0.25 mm, Length 30 m, df 0.25 μm) using dodecane as an internal standard. Conventional and high resolution mass spectra were recorded with a JEOL JMS-DX303HF spectrometer (EI and CI) with a double-focusing magnetic sector mass instrument or JEOL JMS-T100TD (DART and ESI) with a time-of-flight mass instrument. Melting points were measured using Stanford Research Systems OptiMelt MPA 100 with a glass capillary. Elemental analyses were performed on JM10 (J-Science Lab Co. Ltd.).

All manipulations involving air- and moisture-sensitive compounds were carried out by the standard Schlenk techniques under a nitrogen atmosphere unless otherwise noted. Dehydrated THF and Et_2O were purchased from Kanto Chemical Company and purified by SPS[S9] prior to use. Dehydrated toluene and pentane were purchased from Wako Pure Chemical Industries and used as received. THF-d_8 and 1,2-dimethoxyethane (DME) were distilled from sodium benzophenone ketyl. 12-Crown-4 was distilled from sodium metal. Grignard reagents **2f, 2k, 2*l***, and **2m** were prepared by a standard procedure. Other THF solutions of Grignard reagents were purchased from Aldrich or TCI. A cyclohexane/ether solution of phenyllithium was purchased from Kanto. Ether solutions of 2,6-dimethylphenyllithium and 2-methylphneyllithium were prepared by the reaction of the corresponding bromide with lithium under an Ar atmosphere. These organomagnesium and lithium reagents were used after titration using I_2. $NiBr_2$(dme) (Aldrich), $Ni(cod)_2$ (Kanto), 1,3-butadiene (TCI), and all other commercially available reagents were used as received.

Synthesis of Substrates

2-(6-Fluorohexan-1-yloxy)tetrahydropyran (1b)[S10]

A mixture of 6-chloro-1-hexanol (5.43 g, 39.7 mmol) and *n*-$Bu_4N{\bullet}F(H_2O)_5$ (27.10 g, 77.1 mmol) was stirred at 80 ºC for 7 h. After addition of H_2O (20 mL), products were extracted by EtOAc for three times. The combined organic layer was dried over Na_2SO_4, through a short pad of silica gel, and concentrated to give an orange oil. The fluorinated product was purified by distillation (bp = 82-85 ºC, 1.1 kPa) to give 6-fluoro-1-hexanol as a colorless oil (2.97 g, 24.7 mmol, 62%): ^{1}H NMR (400 MHz, $CDCl_3$): δ 4.45 (dt, *J* = 47.2, 6.0 Hz, 2H, F*CH2*), 3.63 (t, *J* = 7.2 Hz, 2H, C*H2*OH), 2.15 (br s, 1H, O*H*), 1.75–1.66 (m, 2H, *methylene*), 1.58 (quin, *J* = 6.8 Hz, 2H, *methylene*), 1.46–1.39 (m, 4H, *methylene*); ^{13}C NMR (100 MHz, $CDCl_3$): δ 84.0 (d, *J* = 163.0 Hz), 62.5, 32.4 (d, *J* = 5.4 Hz), 30.2 (d, *J* = 19.1 Hz), 25.3, 24.9 (d, *J* = 5.7 Hz); ^{19}F NMR (376 MHz, $CDCl_3$): δ –218.1 (sept, *J* = 23.3 Hz); MS (CI): *m/z* (relative intensity, %): 121 ($[M+H]^+$, 100), 103 (70), 83 (17); HRMS (CI) *m/z* $[M+H]^+$ Calcd for $C_6H_{14}FO$ 121.1029; Found 121.1028.

To a solution of 6-fluoro-1-hexanol (1.20 g, 10.0 mmol), pyridinium *p*-toluenesulfonate (53.4 mg, 2 mol%), and CH_2Cl_2 (10 mL) was added dihydropyran (1.08 g, 12.8 mmol) at rt. After stirring at rt for 16 h, the reaction mixture was diluted with EtOAc (200 mL) and washed with H_2O (50 mL x 2), 1N HCl aq. (50 mL), sat. $NaHCO_3$ aq. (50 mL), and brine (50 mL). The organic layer was dried over Na_2SO_4 and concentrated. The fluorinated product was purified by silica gel column chromatography (hexane/EtOAc = 90/10) to give the title compound as a pale yellow colorless oil (2.03 g, 9.95 mmol, 92%): ^{1}H NMR (400 MHz, $CDCl_3$): δ 4.50 (q, *J* = 2.4 Hz, 1H, OC*H*O), 4.36 (dt, *J* = 47.6, 6.4 Hz, 2H, FC*H2*), 3.82–3.77 (m, 1H, OC*H2*), 3.67 (dt, *J* = 9.6, 6.8 Hz, 1H, OC*H2*), 3.46–3.40 (m, 1H, OC*H2*), 3.33 (dt, *J* = 9.6, 6.8 Hz, 1H, OC*H2*), 1.88–1.70 (m, 1H, *methylene*), 1.68–1.43 (m, 9H, *methylene*), 1.37–1.33 (m, 4H, *methylene*); ^{13}C NMR (100 MHz, $CDCl_3$): δ 98.8, 83.9 (d, *J* = 163.0 Hz), 67.3, 62.2, 30.7, 30.3, 30.1, 29.5, 25.8, 25.4, 25.0, 24.9, 19.6; ^{19}F NMR (376 MHz, $CDCl_3$): δ –218.2 (sept, *J* = 23.3 Hz); MS (DART) *m/z* (relative intensity, %): 205 ($[M]^+$, 15), 203 (75), 195 (100), 187 (28); HRMS (DART) *m/z* $[M+H]^+$ Calcd for $C_{11}H_{22}FO_2$ 205.1604; Found 205.1606.

1-*tert*-Butyldimethylsiloxy-6-fluorohexane (1c)[S1]

To a solution of 6-fluoro-1-hexanol (1.21 g, 10.1 mmol), triethylamine (1 mL), and DMF (5 mL) was added *tert*-butyldimethylsilyl chloride (TBDMSCl , 1.81 g, 12.0 mmol) at 0 ˚C. After stirring at rt for 12 h, the reaction mixture was diluted with hexane (200 mL) and washed with H_2O (50 mL x 3) and brine (50 mL). The organic layer was dried over Na_2SO_4 and concentrated. The fluorinated product was purified by silica gel column chromatography (hexane/EtOAc = 100/0 to 80/20) to give the title compound as a colorless oil (1.76 g, 7.52 mmol, 75%): ^{1}H NMR (400 MHz, $CDCl_3$): δ 4.37 (dt, *J* = 47.2, 6.0 Hz, 2H, FC*H2*), 3.54 (t, *J* = 6.8 Hz, 2H, C*H2*CO), 1.69–1.58 (m, 2H, *methylene*), 1.46 (quin, *J* = 6.8 Hz, 2H, *methylene*), 1.36–1.30 (m, 4H, *methylene*), 0.83 (s, 9H, *t-Bu*), –0.02 (s, 6H, *Me*): ^{13}C NMR (100

MHz, $CDCl_3$): δ 84.1 (d, *J* = 163.1 Hz), 63.1, 32.7, 30.4 (d, *J* = 19.1 Hz), 25.9, 25.5, 25.0 (d, *J* = 4.8 Hz), 18.3, –5.3: ^{19}F NMR (376 MHz, $CDCl_3$): δ –218.0 (sept, *J* = 23.3 Hz): MS (CI) *m/z* (relative intensity, %): 235 ($[M+H]^+$, 100): HRMS (CI) *m/z* $[M+H]^+$ Calcd for $C_{12}H_{28}FOSi$ 235.1893; Found 235.1896.

2-(3-Fluoropropyl)thiophene (1d)[S10]

To a solution of thiophene (3.31, 39.4 mmol) in THF (20 mL) was added *n*-BuLi (1.6 M in hexane, 27.5 mL, 44.0 mmol) at 0 ºC and the mixture was stirred at the same temperature for 1 h. To the resulting mixture was added 1,3-dibromopropane (8.2 mL, 80.4 mmol) at –18 ºC and the mixture was allowed to warm to rt. After stirring for 7 h at rt, the reaction mixture was concentrated and quenched by addition of H_2O. The product was extracted with Et_2O for three times. The combined organic layer was dried over Na_2SO_4 and concentrated and the product was purified by distillation (bp = 92-94 ºC, 0.4 kPa) to give 2-(3-bromopropyl)thiophene as a colorless oil (4.63 g, 22.6 mmol, 57%).

A mixture of 2-(3-bromopropyl)thiophene (2.05 g, 10.0 mmol) and *n*-Bu_4N•$F(H_2O)_5$ (6.91 g, 19.4 mmol) was stirred at 80 ºC for 8 h. After addition of H_2O (10 mL), products were extracted by EtOAc for three times. The combined organic layer was dried over Na_2SO_4, through a short pad of silica gel, and concentrated to give an orange oil. The fluorinated product was purified by silica gel column chromatography (hexane/EtOAc = 100/0 to 80/20) to give the title compound as a colorless oil (0.87 g, 6.03 mmol, 60%): ^{1}H NMR (400 MHz, $CDCl_3$): δ 7.13 (dd, *J* = 5.2, 1.2 Hz, 1H, *thiophene*), 6.93 (dd, *J* = 5.2, 3.2 Hz, 1H, *thiophene*), 6.82 (dd, *J* = 3.2, 1.2 Hz, 1H, *thiophene*), 4.48 (dt, *J* = 47.2, 6.0 Hz, 2H, FCH_2), 2.98 (t, *J* = 7.6 Hz, 2H, $FCH_2CH_2CH_2$), 2.13–1.99 (m, 2H, *methylene*); ^{13}C NMR (100 MHz, $CDCl_3$): δ 143.7, 126.8, 124.6, 123.3, 82.8 (d, *J* = 164.0 Hz), 32.3 (d, *J* = 19.1 Hz), 25.4 (d, *J* = 5.7 Hz); ^{19}F NMR (376 MHz, $CDCl_3$): δ –220.6 (sept, *J* = 23.3 Hz); MS (EI) *m/z* (relative intensity, %): 144 ($[M]^+$, 63), 111 (4), 97 (100); HRMS (EI) *m/z* $[M]^+$ Calcd for C_7H_9FS 144.0409; Found 144.0411.

4-Fluorophenyl 3-fluoropropyl ether (1e)[S10]

To a solution of *p*-fluorophenol (2.28 g, 20.4 mmol) in THF (20 mL) was added NaH (60% in mineral oil, 1.0 g, 25 mmol) at 0 ºC followed by addition of 1,3-dichloropropane (4.52 g, 40.0 mmol). The resulting mixture was refluxed for 2 days. However, reaction was not completed, therefore solvent was changed to DMF (5 mL) and reaction was continued at 100 ºC for 40 h. The reaction mixture was cooled to ambient temperature, diluted with Et_2O and washed with water for three times and brine. The organic layer was dried over Na_2SO_4 and concentrated. Purification by silica gel column chromatography (hexane/EtOAc = 100/0 to 95/5) gave 3-chloropropyl 4-fluorophenyl ether as a colorless oil (2.35 g, 13.6 mmol, 67%): ^{1}H NMR (400 MHz, $CDCl_3$): δ 6.90 (dd, *J* = 9.6, 7.6 Hz, 2H, Ar), 6.78–6.75 (m, 2H, Ar), 3.99 (t, *J* = 6.0 Hz, 2H, OCH_2), 3.67 (t, *J* = 6.4 Hz, 2H, $ClCH_2$), 2.14 (quin, *J* = 6.0 Hz, 2H, *methylene*); ^{13}C NMR (100 MHz, $CDCl_3$): δ 158.5, 156.1, 154.8, 115.9, 115.7, 115.5, 115.4,

64.8, 41.4, 32.2; ^{19}F NMR (376 MHz, $CDCl_3$): δ –123.7 (s); MS (DART): *m/z* 188 ($[M]^+$).

A mixture of the chloride (1.88 g, 10.0 mmol) and *n*-Bu_4N•$F(H_2O)_5$ (6.90 g, 19.6 mmol) was stirred at 80 ºC for 8 h. After addition of H_2O (10 mL), products were extracted by EtOAc for three times. The combined organic layer was dried over Na_2SO_4, filtrated through a short pad of silica gel, and concentrated to give an orange oil. The fluorinated product was purified silica gel column chromatography (hexane/EtOAc = 100/0 to 90/10) to give the title compound as a colorless oil (1.317 g, 7.65 mmol, 77%): ^{1}H NMR (400 MHz, $CDCl_3$): δ 6.90 (dd, *J* = 9.6, 7.6 Hz, 2H, *Ar*), 6.78–6.75 (m, 2H, *Ar*), 3.99 (t, *J* = 6.0 Hz, 2H, OC*H₂*), 3.67 (t, *J* = 6.4 Hz, 2H, ClC*H₂*), 2.14 (quin, *J* = 6.0 Hz, 2H, *methylene*); ^{13}C NMR (100 MHz, $CDCl_3$): δ 158.4, 156.1, 154.9, 115.9, 115.7, 115.43, 115.36, 80.6 (d, *J* = 163.1 Hz), 64.1 (d, *J* = 4.7 Hz), 30.4 (d, *J* = 20.0 Hz); ^{19}F NMR (376 MHz, $CDCl_3$): δ –123.9 (s, 1F), –222.2 (sept, *J* = 22.9 Hz, 1F); MS (EI) *m/z* (relative intensity, %): 172 ($[M]^+$, 38), 112 (100); HRMS (EI) *m/z* $[M]^+$ Calcd for $C_9H_{10}F_2O$ 172.0700; Found 172.0699.

4-Chlorophenyl 3-fluoropropyl ether (1f)[S10]

To a solution of *p*-chlorophenol (2.59 g, 20.2 mmol) in THF (20 mL) was added NaH (60% in mineral oil, 1.0 g, 25 mmol) at 0 ºC followed by addition of 1,3-dichloropropane (4.52 g, 40.0 mmol). The resulting mixture was refluxed for 2 days. Since reaction was not completed, solvent was changed to DMF (5 mL) and reaction was continued at 100 ºC for 40 h. The reaction mixture was cooled to ambient temperature, diluted with Et_2O and washed with water for three times and brine. The organic layer was dried over Na_2SO_4 and concentrated. Purification by silica gel column chromatography (hexane/EtOAc = 100/0 to 95/5) gave 4-chlorophenyl 3-chloropropyl ether as a white solid (2.27 g, 12.0 mmol, 60%): ^{1}H NMR (400 MHz, $CDCl_3$): δ 7.15 (d, *J* = 9.2 Hz, 2H, *Ar*), 6.75 (d, *J* = 9.2 Hz, 2H, *Ar*), 4.00 (t, *J* = 6.0 Hz, 2H, OC*H₂*), 3.66 (t, *J* = 6.4 Hz, 2H, ClC*H₂*), 2.14 (quin, *J* = 6.4 Hz, 2H, *methylene*); ^{13}C NMR (100 MHz, $CDCl_3$): δ 157.3, 129.3, 125.7, 115.7, 64.5, 41.4, 32.1; MS (DART): *m/z* 205 ($[M]^+$).

A mixture of the chloride (2.022 g, 9.86 mmol) and *n*-Bu_4N•$F(H_2O)_5$ (6.94 g, 19.7 mmol) was stirred at 80 ºC for 8 h. After addition of H_2O (10 mL), products were extracted by EtOAc for three times. The combined organic layer was dried over Na_2SO_4, through a short pad of silica gel, and concentrated to give an orange oil. The fluorinated product was purified silica gel column chromatography (hexane/EtOAc = 100/0 to 90/10) to give the title compound as a colorless oil (1.138 g, 6.03 mmol, 61%): ^{1}H NMR (400 MHz, $CDCl_3$): δ 7.15 (d, *J* = 8.8 Hz, 2H, *Ar*), 6.74 (d, *J* = 8.8 Hz, 2H, *Ar*), 4.55 (dt, *J* = 46.8, 6.0 Hz, 2H, FC*H₂*), 3.97 (t, *J* = 6.0 Hz, 2H, OC*H₂*), 2.07 (d,quin, *J* = 26.0, 6.0 Hz, 2H, *methylene*); ^{13}C NMR (100 MHz, $CDCl_3$): δ 157.3, 129.3, 125.6, 115.7, 80.5 (d, *J* = 164.0 Hz), 63.7 (d, *J* = 5.7 Hz), 30.3 (d, *J* = 20.1 Hz); ^{19}F NMR (376 MHz, $CDCl_3$): δ –222.2 (sept, *J* = 22.9 Hz); MS (EI) *m/z* (relative intensity, %): 188 ($[M]^+$, 33), 130 (32), 128 (100); HRMS (EI) *m/z* $[M]^+$ Calcd for $C_9H_{10}ClFO$ 188.0404; Found 188.0404.

4-(2-Fluoroethyl)-1-tosylpiperidine (1g)[S10]

To a solution of piperidineethanol (2.550 g, 19.74 mmol) and Et_3N (4.0 mL) in CH_2Cl_2 (20 mL) was added TsCl (4.261 g, 22.35 mmol) at 0 ºC and then stirred at rt for 2 h. The resulting mixture was diluted with CH_2Cl_2 and washed with water, 1N HCl, sat. $NaHCO_3$ aq., and brine. The organic layer was dried over Na_2SO_4 and concentrated. The crude mixture was used in the next step without further purifications. To the crude mixture dissolved in THF (30 mL) were added PPh_3 (5.788 g, 22.07 mmol) and CCl_4 (3 mL) and the mixture was stirred at 50 ºC for 24 h. The resulting mixture was concentrated and purified by silica gel column chromatograph (hexane/EtOAc = 5/1) to give 4-(2-chloroethyl)-1-tosylpiperidine as a white solid (5.256 g, 17.41 mmol, 88%).

A mixture of the chloride (5.256 g, 17.41 mmol) and n-Bu_4N•$F(H_2O)_5$ (14.16 g, 40.22 mmol) was stirred at 80 ºC for 12 h. After addition of H_2O (10 mL), products were extracted by EtOAc for three times. The combined organic layer was dried over Na_2SO_4, through a short pad of silica gel, and concentrated to give an orange oil. The fluorinated product was purified silica gel column chromatography (hexane/EtOAc = 100/0 to 70/30) to give the title compound as a white solid (2.576 g, 9.027 mmol, 52%): mp = 112 °C; ^{1}H NMR (400 MHz, $CDCl_3$): δ 7.64 (d, J = 8.0 Hz, 2H, *Ar*), 7.32 (d, J = 8.0 Hz, 2H, *Ar*), 4.46 (dt, J = 47.2, 6.0 Hz, 2H, CH_2F), 3.77 (d, J = 12.0 Hz, 2H, *piperidine*), 2.43 (s, 3H, *Me*), 2.23 (t, J = 10.8 Hz, 2H, *piperidine*), 1.77 (d, J = 12.0 Hz, 2H, *piperidine*), 1.69–1.55 (m, 2H, methylene), 1.50–1.30 (m, 3H, *piperidine*); ^{13}C NMR (100 MHz, $CDCl_3$): δ 143.4, 132.9, 129.5, 127.7, 82.4, 80.8, 46.3, 36.5, 36.3, 31.61, 31.57, 31.2, 21.5; ^{19}F NMR (376 MHz, $CDCl_3$): δ –117.6 (sept, J = 22.9 Hz); MS (EI) *m/z* (relative intensity, %): 285 ($[M]^+$, 43), 284 (35), 155 (31), 130 (100); HRMS (EI) *m/z* $[M]^+$ Calcd for $C_{14}H_{20}FNO_2S$ 285.1199; Found 285.1197.

11-Fluoro-1-undecene (1j)[S10]

A mixture of 11-chloro-1-undecene (2.39 g, 10.3 mmol) and n-Bu_4N•$F(H_2O)_5$ (7.13 g, 20.3 mmol) was stirred at 80 ºC for 8 h. After addition of H_2O (10 mL), products were extracted by EtOAc for three times. The combined organic layer was dried over Na_2SO_4, through a short pad of silica gel, and concentrated to give an orange oil. The fluorinated product was purified by distillation (bp = 84 ºC, 0.9 kPa) to give a colorless oil. Further purification by GPC ($CHCl_3$ as an eluent) gave the title compound as a colorless oil (0.535 g, 3.11 mmol, 30%): ^{1}H NMR (400 MHz, $CDCl_3$): δ 5.87–5.74 (m, 1H, CH=CH_2), 5.02–4.91 (m, 2H, CH=CH_2), 4.43 (dt, J = 48.0, 6.8 Hz, 2H, FCH_2), 2.07–2.01 (m, 2H, *methylene*), 1.75–1.62 (m, 2H, FCH_2CH_2), 1.39–1.29 (m, 12H, *methylene*); ^{13}C NMR (100 MHz, $CDCl_3$): δ 139.2, 114.1, 84.2 (d, J = 163.1 Hz), 33.8, 30.4 (d, J = 19.1 Hz), 29.44, 29.37, 29.2, 29.1, 28.9, 25.1 (d, J = 4.7 Hz); ^{19}F NMR (376 MHz, $CDCl_3$): δ –217.9 (t, J = 52.3 Hz); MS (DART) *m/z* (relative intensity, %): 173 ($[M+H]^+$, 2), 151 (7), 102 (100); HRMS (DART) *m/z* $[M+H]^+$ Calcd for $C_{11}H_{22}F$ 173.1706; Found 173.1724.

1-*tert*-Butoxycarbony-4-(2-fluoroethyl)piperidine (1k)[S1]

The title compound was synthesized from 1-*tert*-butoxycarbonyl-4-(2-fluoroethyl)piperidine by a similar procedure employed for the synthesis of 4-(2-fluoroethyl)-1-tosylpiperidine. White solid: mp 36 ºC; ^{1}H NMR (400 MHz, $CDCl_3$): δ 4.52 (dt, *J* = 47.2, 6.0 Hz, 2H, CH_2F), 4.10 (br, 2H), 2.70 (br, 2H), 1.71–1.60 (m, 5H), 1.46 (s, 9H, *t-Bu*), 1.18–1.10 (m, 2H); ^{13}C NMR (100 MHz, $CDCl_3$): δ 154.8, 81.9 (d, *J* = 163.0 Hz), 79.3, 44.0 (br), 36.9 (d, *J* = 19.0 Hz), 32.4 (d, *J* = 3.8 Hz), 31.9, 28.4; ^{19}F NMR (376 MHz, $CDCl_3$): δ –218.4—218.8 (m); MS (DART) *m/z* (relative intensity, %): 232 ($[M+H]^+$, 89), 195 (12), 176 (100), 132 (35); HRMS (DART) *m/z* $[M+H]^+$ Calcd for $C_{12}H_{23}NO_2F$ 232.1713; Found 232.1699.

3-Fluoropropyl phenyl ether (1*l*)[S10]

A mixture of 3-bromopropyl phenyl ether (2.23 g, 10.37 mmol) and *n*-Bu_4N•$F(H_2O)_5$ (7.00 g, 19.9 mmol) was stirred at 80 ºC for 8 h. After addition of H_2O (10 mL), products were extracted by EtOAc for three times. The combined organic layer was dried over Na_2SO_4, filtrated through a short pad of silica gel, and concentrated to give an orange oil. The fluorinated product was purified silica gel column chromatography (hexane/EtOAc = 100/0 to 90/10) to give the title compound as a colorless oil (0.628 g, 6.49 mmol, 63%): ^{1}H NMR (400 MHz, $CDCl_3$): δ 7.32–7.27 (m, 12, *Ph*), 6.97–6.90 (m, 3H, *Ph*), 4.65 (dd, *J* = 46.8, 5.6 Hz, 2H, FCH_2), 4.09 (t, *J* = 5.6 Hz, 2H, OCH_2), 2.17 (dt, *J* = 26.0, 5.6 Hz, 2H, *methylene*): ^{13}C NMR (100 MHz, $CDCl_3$): δ 158.7, 129.5, 120.8, 114.4, 80.8 (d, *J* = 163.0 Hz), 63.3 (d, *J* = 4.7 Hz), 30.4 (d, *J* = 20.0 Hz): ^{19}F NMR (376 MHz, $CDCl_3$): δ –222.2 (q, *J* = 51.9 Hz): MS (EI) *m/z* (relative intensity, %): 154 ($[M]^+$, 44), 94 (100): HRMS (EI) *m/z* $[M]^+$ Calcd for $C_9H_{11}FO$ 154.0794; Found 154.0796.

1-Chloro-6-fluorohexane (1m)[S1]

To a solution of 6-fluoro-1-hexanol (1.21 g, 10.1 mmol) and PPh_3 (2.90 g, 11.1 mmol) in CH_2Cl_2 (10 mL) was added CCl_4 (1.2 mL, 12.4 mmol) at rt and then refluxed for 3 h. After cooling to ambient temperature, pentane (50 mL) was added to give a white suspension. The mixture was filtrated through a short pad of silica gel and concentrated. The fluorinated product was purified by distillation (bp = 73-76 ºC, 4.1 kPa) to give the title compound as a colorless oil (0.751 g, 5.42 mmol, 54%): ^{1}H NMR (400 MHz, $CDCl_3$): δ 4.45 (dt, *J* = 47.26, 6.0 Hz, 2H, FCH_2), 3.55 (t, *J* = 6.4 Hz, 2H, $ClCH_2$), 1.83–1.65 (m, 4H, *methylene*), 1.53–1.43 (m, 4H, *methylene*); ^{13}C NMR (100 MHz, $CDCl_3$): δ 83.9 (d, *J* = 163.1 Hz), 44.9, 32.4, 30.2 (d, *J* = 20.0 Hz), 26.5, 24.5 (d, *J* = 5.7 Hz); ^{19}F NMR (376 MHz, $CDCl_3$): δ –218.4 (t, *J* = 69.2 Hz); MS (CI) *m/z* (relative intensity, %): 119 ($[M–F]^+$, 55), 103 ($[M–F]^+$, 100), 83 (99).

1,1-Diphenyl-6-fluorohex-1-ene (1n)[S10]

To a solution of 6,6-diphenylhex-5-en-1ol (1.54 g, 6.09 mmol) in CH_2Cl_2 (2 mL) was added DAST (1.30 mL, 9.84 mmol) at 0 ˚C. After stirring for 18 h, the reaction mixture was diluted with CH_2Cl_2 and washed with H_2O (50 mL x 3). The organic layer was dried over Na_2SO_4 and concentrated. The fluorinated product was purified by silica gel column chromatography (hexane/EtOAc = 100/0 to 98/2) to give the title compound as a colorless oil (0.887 g, 3.49 mmol, 57%). ^{1}H NMR (400 MHz, $CDCl_3$): δ 7.39–7.12 (m, 10H, *Ph*), 6.07 (t, *J* = 7.6 Hz, 1H, =C*H*), 4.39 (dt, *J* = 47.2, 6.0 Hz, 2H, FCH_2), 2.16 (q, *J* = 7.6 Hz, 2H, CH_2CH=), 1.76–1.52 (m, 4H, *methylene*): ^{13}C NMR (100 MHz, $CDCl_3$): δ 142.6, 142.1, 140.1, 129.8, 129.2, 128.2, 128.1, 127.2, 126.90, 126.87, 83.9 (d, *J* = 164.0 Hz), 29.9 (d, *J* = 19.1 Hz), 29.2, 25.4 (d, *J* = 5.7 Hz): ^{19}F NMR (376 MHz, $CDCl_3$): δ –218.2 (sept, *J* = 25.9 Hz): MS (EI) *m/z* (relative intensity, %): 254 ($[M]^+$, 51), 193 (100): HRMS (EI) *m/z* $[M]^+$ Calcd for $C_{18}H_{19}F$ 254.1471; Found 254.1468.

General procedure for optimization of the four-component coupling of *n*-OctF, 4-fluorophenyl Grignard reagent, and 1,3-butadiene (Table 1)

An oven-dried test tube was charged with catalyst and a stirring bar and closed with a septum cap. The test tube was evacuated and back-filled with nitrogen three times. 1-Fluorooctane (**1a**) (132 mg, 1.0 mmol) and THF (0.50 mL) were added via syringe at ambient temperature. 4-Fluorophenylmagnesium bromide (**2a**) (in THF, 0.80 M, 2.50 mL, 2.0 mmol) and 1,3-butadiene (67 mL as gas, 3.0 mmol) were added at –78 ºC and then stirred in an oil bath. After 24 h, the reaction mixture was diluted with Et_2O and carefully quenched by 1N HCl aq. After addition of dodecane (30 μL) as an internal standard, organic layer was analyzed by GC to determine the yield of the products **3aa** and **4aa**.

General procedure for Ni-catalyzed four-component coupling of alkyl fluorides, Grignard reagents and 1,3-butadiene (Scheme 2, eqn 1, and eqn 5)

In a glove box, $NiBr_2$(dme) (30.9 mg, 10 mol%) was charged to a test tube and capped with a septum. The test tube was brought out and added alkyl fluoride (1 mmol) and THF (total 3.0 mL) via syringe at ambient temperature. Grignard reagent (in THF, 2.0 mmol) and 1,3-butadiene (67 mL as gas, 3.0 mmol) were added at –78 ºC and then stirred at 30 ºC. After 24 h, the reaction mixture was carefully quenched by 1N HCl aq. and the products were extracted by Et_2O for three times, dried over Na_2SO_4, concentrated, filtered through a short pad of silica gel (hexane or Et_2O as eluent), and purified by GPC ($CHCl_3$ as eluent) to give the desired product.

General procedure for optimization of the four-component coupling of *n*-OctF, 2-methylphenyl Grignard reagent, and 1,3-butadiene (Table 2)

An oven-dried test tube was charged with catalyst and a stirring bar and closed with a septum cap. The test tube was evacuated and back-filled with nitrogen three times. 1-Fluorooctane (**1a**) (132 mg, 1.0 mmol) and THF (1.17 mL) were added via syringe at ambient temperature. 2-Methylphenylmagnesium bromide (**2a**) (in THF, 0.82 M, 1.83 mL, 1.5 mmol) and 1,3-butadiene (67 mL as gas, 3.0 mmol) were added at –78 ºC and then stirred in an oil bath. After 10 h, the reaction mixture was diluted with Et_2O and carefully quenched by 1N HCl aq. After addition of dodecane (30 μL) as an internal standard, organic layer was analyzed by GC to determine the yield of the product **3aj**.

General procedure for Ni-catalyzed four-component coupling of alkyl fluorides, *ortho*-substituted Grignard reagents, and 1,3-butadiene (Scheme 3, and eqn 6)

An oven-dried test tube was charged with $NiBr_2$(dme) (7.7 mg, 5 mol%) and a stirring bar and closed with a septum cap. The test tube was evacuated and back-filled with nitrogen three times. Alkyl fluoride (0.5 mmol) and THF were added via syringe at ambient temperature. Grignard reagent (in THF, 0.75 mmol), and 1,3-butadiene (34 mL as gas, 1.5 mmol) were added at –78 ºC and then stirred at 40 ºC.

After 10 h, the reaction mixture was diluted with Et_2O and carefully quenched by 1N HCl aq. The products were extracted by Et_2O for three times, dried over Na_2SO_4, concentrated, filtered through a short pad of silica gel (hexane or Et_2O as eluent), and purified by GPC ($CHCl_3$ as eluent) to give the desired product.

Characterization Data for the Isolated Compounds

The reaction of 1-fluorooctane (1a) with 4-fluorophenylmagnesium bromide (2a)

The representative general procedure was followed using 1-fluorooctane (141.0 mg, 1.07 mmol), 4-fluorophenylmagnesium bromide (in THF, 0.80 M, 2.5 mL, 2.0 mmol), and 1,3-butadiene (68 mL as gas, 3 mmol) in THF (total 3 mL) at 30 °C for 24 h. Purification by GPC gave the desired product **3aa** as a colorless oil (163.2 mg, 48%) accompanied by the cross-coupling product **4aa** as a colorless oil (82.8 mg, 37%).

(*E*)-1-(4-Fluorophenyl)-6-vinyltetradec-2-ene (3aa):[S1]

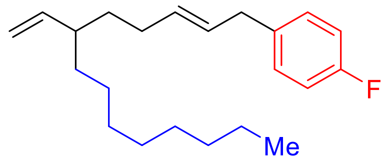

^{1}H NMR (400 MHz, $CDCl_3$): δ 7.12 (dd, *J* = 8.4, 5.6 Hz, 2H), 6.96 (t, *J* = 8.4 Hz, 2H), 5.54–5.44 (m, 3H), 4.97–4.89 (m, 2H), 3.28 (d, *J* = 5.2 Hz, 2H), 2.10–1.89 (m, 3H), 1.48–1.38 (m, 1H), 1.36–1.17 (m, 15H), 0.88 (t, *J* = 6.8 Hz, 3H); ^{13}C NMR (100 MHz, $CDCl_3$): δ 162.5, 160.1, 143.2, 136.7, 136.6, 132.2, 129.8, 129.7, 128.6, 115.1, 114.9, 114.2, 43.6, 38.2, 35.0, 34.7, 31.9, 30.1, 29.8, 29.6, 29.3, 27.1, 22.7, 14.1; ^{19}F NMR (376 MHz, $CDCl_3$): δ –117.8 (s); MS (EI) *m/z* (relative intensity, %): 316 ($[M]^+$, 2), 287 (1), 162 (22), 147 (28), 122 (37), 109 (100); HRMS (EI) *m/z* $[M]^+$ Calcd for $C_{22}H_{33}F$ 316.2566; Found 316.2573.

1-Fluoro-4-octylbenzene (4aa):[S1]

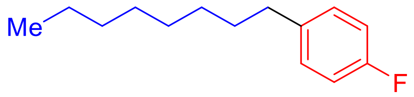

^{1}H NMR (400 MHz, $CDCl_3$): δ 7.11 (dd, *J* = 8.8, 5.6 Hz, 2H), 6.95 (t, *J* = 8.8 Hz, 2H), 2.56 (t, *J* = 7.6 Hz, 2H), 1.59–1.56 (m, 2H), 1.30–1.26 (m, 10H), 0.88 (t, *J* = 6.8 Hz, 3H); ^{13}C NMR (100 MHz, $CDCl_3$): δ 162.3, 159.9, 138.49, 138.46, 129.7, 129.6, 115.0, 114.8, 35.1, 31.9, 31.6, 29.4, 29.24, 29.20, 22.7, 14.1; ^{19}F NMR (376 MHz, $CDCl_3$): δ –118.2 (s); MS (EI) *m/z* (relative intensity, %): 208 ($[M]^+$, 15), 109 (100); HRMS (EI) *m/z* $[M]^+$ Calcd for $C_{14}H_{21}F$ 208.1627; Found 208.1625.

The reaction of 2-(6-fluorohexan-1-yloxy)tetrahydropyran (1b) with 4-fluorophenylmagnesium bromide (2a)

The representative general procedure was followed using 2-(6-fluorohexan-1-yloxy)tetrahydropyran (204.4 mg, 1.00 mmol), 4-fluorophenylmagnesium bromide (in THF, 0.80 M, 2.5 mL, 2.0 mmol), and 1,3-butadiene (68 mL as gas, 3 mmol) in THF (total 3 mL) at 30 °C for 24 h. Purification by GPC gave the desired product **3ba** as a colorless oil (188.3 mg, 48%) accompanied by the cross-coupling product **4ba** as a colorless oil (97.3 mg, 35%).

(*E*)-2-[12-(4-Fluorophenyl)-7-vinyl-10-dodecen-1-yloxy]tetrahydropyran (3ba):

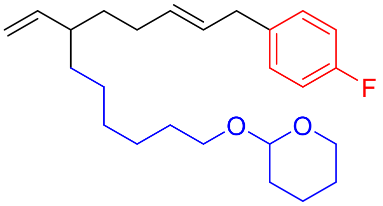

^{1}H NMR (400 MHz, $CDCl_3$): δ 7.12 (dd, *J* = 8.4, 5.6 Hz, 2H), 6.96 (t, *J* = 8.4 Hz, 2H), 5.54–5.44 (m, 3H), 4.97–4.89 (m, 2H), 4.57 (q, *J* = 2.4 Hz, 1H), 3.89–3.84 (m, 1H), 3.75–3.70 (m, 1H), 3.51–3.47 (m, 1H), 3.40–3.35 (m, 1H), 3.28 (d, *J* = 5.6 Hz, 2H), 2.09–1.22 (m, 21H); ^{13}C NMR (100 MHz, $CDCl_3$): δ 162.5,

160.1, 143.1, 136.63, 136.60, 132.1, 129.8, 129.7, 128.6, 115.1, 114.9, 114.3 67.7, 62.3, 43.6, 38.2, 34.9, 34.7, 30.8, 30.1, 29.7, 29.5, 27.0, 26.2, 25.5, 18.7; ^{19}F NMR (376 MHz, $CDCl_3$): δ –117.8 (s); MS (EI) *m/z* (relative intensity, %): 388 ($[M]^+$, 1), 161 (11), 149 (12), 148 (16), 109 (34), 85 (100); HRMS (EI) *m/z* $[M]^+$ Calcd for $C_{25}H_{37}FO_2$ 388.2778; Found 388.2775.

2-[6-(4-Fluorophenyl)-hexan-1-yloxy]tetrahydropyran (4ba):

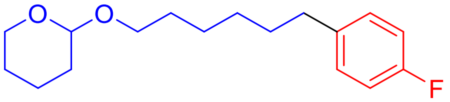

^{1}H NMR (400 MHz, $CDCl_3$): δ 7.11 (dd, *J* = 8.4, 5.6 Hz, 2H), 6.95 (t, *J* = 8.4 Hz, 2H), 4.57 (q, *J* = 2.8 Hz, 1H), 3.89–3.83 (m, 1H), 3.76–3.70 (m, 1H), 3.52–3.47 (m, 1H), 3.40–3.35 (m, 1H), 2.57 (t, *J* = 7.6 Hz, 2H), 1.84–1.79 (m, 1H), 1.74–1.68 (m, 1H), 1.63–1.50 (m, 8H), 1.41–1.32 (m, 4H); ^{13}C NMR (100 MHz, $CDCl_3$): δ 162.3, 159.9, 138.33, 138.29, 129.64, 129.62, 129.57, 129.55, 115.0, 114.8, 98.8, 67.6, 62.3, 35.0, 31.5, 30.75, 30.73, 29.6, 29.0, 26.1, 25.5, 19.7; ^{19}F NMR (376 MHz, $CDCl_3$): δ –118.2 (s); MS (CI) *m/z* (relative intensity, %): 281 ($[M+H]^+$, 63), 179 (33), 109 (11), 85 (100); HRMS (CI) *m/z* $[M+H]^+$ Calcd for $C_{17}H_{26}O_2$ 281.1917; Found 281.1916.

The reaction of 6-*tert*-butyldimethylsiloxy-1-fluorohexane (1c) with 4-fluorophenylmagnesium bromide (2a)

The representative general procedure was followed using 6-*tert*-butyldimethylsiloxy-1-fluorohexane (232.4 mg, 0.99 mmol), 4-fluorophenylmagnesium bromide (in THF, 0.80 M, 2.5 mL, 2.0 mmol), and 1,3-butadiene (68 mL as gas, 3 mmol) in THF (total 3 mL) at 30 °C for 24 h. Purification by GPC gave the desired product **3ca** as a colorless oil (192.0 mg, 46%) accompanied by the cross-coupling product **4ca** as a colorless oil (94.8 mg, 31%).

(*E*)-12-*tert*-Butyldimethylsiloxy-1-(4-fluorophenyl)-6-vinyldodec-2-ene (3ca):

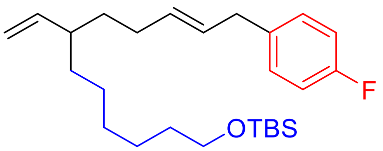

^{1}H NMR (400 MHz, $CDCl_3$): δ 7.12 (dd, *J* = 8.4, 5.6 Hz, 2H), 6.96 (t, *J* = 8.4 Hz, 2H), 5.54–5.45 (m, 3H), 4.97–4.89 (m, 2H), 3.59 (t, *J* = 6.8 Hz, 2H), 3.28 (d, *J* = 5.6 Hz, 2H), 2.09–1.92 (m, 3H), 1.61–1.23 (m, 12H), 0.89 (s, 9H), 0.05 (s, 6H); ^{13}C NMR (100 MHz, $CDCl_3$): δ 162.5, 160.1, 143.2, 136.65, 136.62, 132.2, 129.8, 129.7, 128.6, 115.1, 114.9, 114.3 63.3, 43.6, 38.2, 34.9, 34.7, 32.8, 30.1, 29.5, 27.1, 26.0, 25.8, 18.4. –5.3; ^{19}F NMR (376 MHz, $CDCl_3$): δ –117.8 (s); MS (CI) *m/z* (relative intensity, %): 419 ($[M+H]^+$, 100), 361 (80), 287 (34), 183 (67); HRMS (CI) *m/z* $[M+H]^+$ Calcd for $C_{26}H_{44}FOSi$ 419.3145; Found 419.3146.

1-*tert*-Butyldimethylsiloxy-6-(4-Fluorophenyl)hexane (4ca):

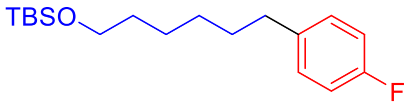

^{1}H NMR (400 MHz, $CDCl_3$): δ 7.11 (dd, *J* = 8.8, 5.6 Hz, 2H), 6.95 (t, *J* = 8.8 Hz, 2H), 3.59 (t, *J* = 6.4 Hz, 2H), 2.57 (t, *J* = 7.6 Hz, 2H), 1.61–1.57 (m, 2H), 1.52–1.49 (m, 2H), 1.35–1.32 (m, 4H), 0.89 (s, 9H), 0.04 (s, 6H); ^{13}C NMR (100 MHz, $CDCl_3$): δ 162.3, 159.9, 138.4, 138.3, 129.7, 129.6, 115.0, 114.8, 63.2, 35.0, 32.8, 31.6, 28.9, 26.0, 25.6, 18.4. –5.3; ^{19}F NMR (376 MHz, $CDCl_3$): δ –118.1 (s); MS (CI) *m/z* (relative intensity, %): 311

([M+H]$^+$, 100), 253 (20), 183 (9), 179 (9); HRMS (CI) *m/z* [M+H]$^+$ Calcd for $C_{18}H_{32}FOSi$ 311.2206; Found 311.2207.

The reaction of 2-(3-fluoropropyl)thiophene (1d) with 4-fluorophenylmagnesium bromide (2a)

The representative general procedure was followed using 2-(3-fluoropropyl)thiophene (141.1 mg, 0.98 mmol), 4-fluorophenylmagnesium bromide (in THF, 0.80 M, 2.5 mL, 2.0 mmol), and 1,3-butadiene (68 mL as gas, 3 mmol) in THF (total 3 mL) at 30 °C for 24 h. Purification by GPC gave the desired product **3da** as a colorless oil (127.9 mg, 40%) accompanied by the cross-coupling product **4da** as a white semi-solid (71.7 mg, 33%).

(*E*)-2-[9-(4-Fluorophenyl)-4-vinylnon-7-en-1-yl]thiophene (3da):

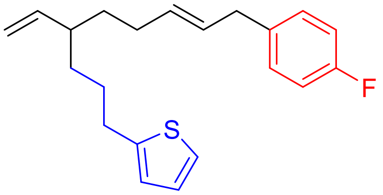

^{1}H NMR (400 MHz, $CDCl_3$): δ 7.13–7.09 (m, 3H), 6.95 (t, *J* = 8.4 Hz, 2H), 6.90 (dd, *J* = 5.6, 3.6 Hz, 1H), 6.76 (d, *J* = 3.2 Hz, 1H), 5.51–5.47 (m, 3H), 4.98 (dd, *J* = 10.4, 2.0 Hz, 1H), 4.94 (d, *J* = 18.0 Hz, 1H), 3.28 (d, *J* = 5.6 Hz, 2H), 2.81–2.78 (m, 2H), 2.10–1.90 (m, 3H), 1.74–1.56 (m, 2H), 1.47–1.39 (m, 2H), 1.35–1.25 (m, 2H); ^{13}C NMR (100 MHz, $CDCl_3$): δ 162.5, 160.1, 145.6, 142.7, 136.61, 136.57, 132.0, 129.8, 129.7, 128.8, 126.6, 123.9, 122.8, 115.1, 114.9, 114.7, 43.4, 38.2, 34.6, 34.3, 30.0, 29.9, 29.4; ^{19}F NMR (376 MHz, $CDCl_3$): δ –117.7 (s); MS (EI) *m/z* (relative intensity, %): 328 ([M]$^+$, 23), 219 (12), 163 (12), 149 (13), 148 (16), 147 (11), 135 (13), 123 (17), 111 (25), 110 (100); HRMS (EI) *m/z* [M]$^+$ Calcd for $C_{21}H_{25}FS$ 328.1661; Found 328.1663.

2-[3-(4-Fluorophenyl)propyl]thiophene (4da):

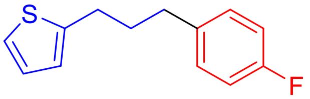

^{1}H NMR (400 MHz, $CDCl_3$): δ 7.13 (d, *J* = 5.6 Hz, 1H), 7.11 (dd, *J* = 5.6, 1.2 Hz, 2H), 6.98–6.78 (m, 3H), 6.77 (dd, *J* = 2.4, 0.8 Hz, 1H), 2.83 (t, *J* = 7.6 Hz, 2H), 2.64 (t, *J* = 7.6 Hz, 2H), 1.97 (quin, *J* = 7.6 Hz, 2H); ^{13}C NMR (100 MHz, $CDCl_3$): δ 162.4, 160.0, 144.9, 137.44, 137.40, 129.8, 129.7, 126.7, 124.20, 124.18, 123.0, 115.1, 114.9, 34.2, 33.3, 29.2; ^{19}F NMR (376 MHz, $CDCl_3$): δ –117.5 (s); MS (EI) *m/z* (relative intensity, %): 220 ([M]$^+$, 44), 123 (11), 111 (14), 110 (15), 109 (24), 98 (100); HRMS (EI) *m/z* [M]$^+$ Calcd for $C_{13}H_{13}FS$ 220.0722; Found 220.0721.

The reaction of 1-fluoro-3-(4-fluorophenoxy)propane (1e) with 4-fluorophenylmagnesium bromide (2a)

The representative general procedure was followed using 1-fluoro-3-(4-fluorophenoxy)propane (182.4 mg, 1.06 mmol), 4-fluorophenylmagnesium bromide (in THF, 0.80 M, 2.5 mL, 2.0 mmol), and 1,3-butadiene (68 mL as gas, 3 mmol) in THF (total 3 mL) at 30 °C for 24 h. Purification by GPC gave the desired product **3ea** as a colorless oil (165.2 mg, 44%) accompanied by the cross-coupling product **4ea** as a white solid (127.1 mg, 47%).

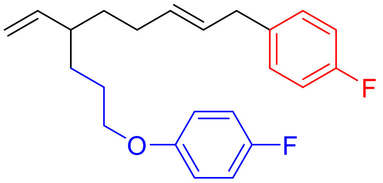

(*E*)-9-(4-Fluorophenoxy)-1-(4-fluorophenyl)-6-vinylnon-2-ene (3ea):

^{1}H NMR (400 MHz, $CDCl_3$): δ 7.11 (td, *J* = 5.6, 2.4 Hz, 2H), 6.95 (td, *J*

= 8.4, 1.6 Hz, 4H), 6.82–6.79 (m, 2H), 5.52–5.47 (m, 3H), 5.02 (m, 2H), 3.88 (t, *J* = 6.4 Hz, 2H), 3.28 (d, *J* = 5.6 Hz, 2H), 2.10–1.96 (m, 3H), 1.81–1.65 (m, 2H), 1.57–1.42 (m, 2H), 1.41–1.29 (m, 2H); ^{13}C NMR (100 MHz, $CDCl_3$): δ 162.5, 160.1, 158.3, 155.9, 155.2, 142.5, 136.60, 136.57, 131.9, 129.8, 129.7, 128.9, 115.8, 115.6, 115.4, 115.3, 115.1, 115.0, 114.9, 68.6, 43.4, 38.2, 34.7, 31.2, 30.1, 27.0; ^{19}F NMR (376 MHz, $CDCl_3$): δ –117.7 (s, 1F), –124.3 (s, 1F); MS (EI) *m/z* (relative intensity, %): 356 ($[M]^+$, 9), 149 (39), 148 (21), (147 (14), 138 (14), 135 (25), 112 (22), 109 (100); HRMS (EI) *m/z* $[M]^+$ Calcd for $C_{23}H_{26}F_2O$ 356.1952; Found 356.1951.

1-(4-Fluorophenoxyl)-3-(4-fluorophenyl)propane (4ea):

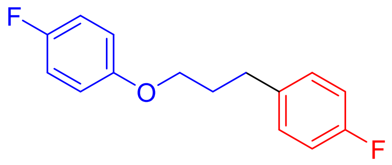

mp = 56–58 °C; ^{1}H NMR (400 MHz, $CDCl_3$): δ 7.16–7.10 (m, 2H), 6.96 (td, *J* = 8.8, 2.4 Hz, 4H), 6.82–6.79 (m, 2H), 3.89 (t, *J* = 6.4 Hz, 2H), 2.77 (t, *J* = 7.6 Hz, 2H), 2.05 (tt, *J* = 7.6, 6.4 Hz, 2H); ^{13}C NMR (100 MHz, $CDCl_3$): δ 162.5, 160.1, 158.4, 156.0, 155.1, 138.8, 138.7, 137.04, 137.00, 131.8, 130.7, 129.9, 129.8, 129.79, 115.9, 115.7, 115.5, 115.4, 115.3, 115.1, 67.3, 31.3, 30.9; ^{19}F NMR (376 MHz, $CDCl_3$): δ –117.4 (s, 1F), –124.1 (s, 1F); MS (EI) *m/z* (relative intensity, %): 248 ($[M]^+$, 44), 137 (15), 136 (18), 135 (13), 112 (17), 109 (100); HRMS (EI) *m/z* $[M]^+$ Calcd for $C_{15}H_{14}F_2O$ 248.1013; Found 248.1012.

The reaction of 3-(4-chlorophenoxy)-1-fluoropropane (1f) with 4-fluorophenylmagnesium bromide (2a)

The representative general procedure was followed using 3-(4-chlorophenoxy)-1-fluoropropane (192.0 mg, 1.02 mmol), 4-fluorophenylmagnesium bromide (in THF, 0.80 M, 2.5 mL, 2.0 mmol), and 1,3-butadiene (68 mL as gas, 3 mmol) in THF (total 3 mL) at 30 °C for 24 h. Purification by GPC gave the desired product **3fa** as a colorless oil (171.7 mg, 45%) accompanied by the cross-coupling product **4fa** as a colorless oil (115.9 mg, 43%).

(*E*)-9-(4-Chlorophenoxy)-1-(4-fluorophenyl)-6-vinylnon-2-ene (3fa):

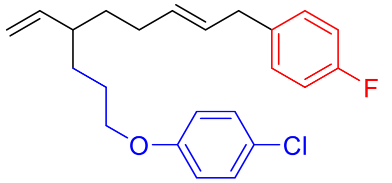

^{1}H NMR (400 MHz, $CDCl_3$): δ 7.21 (d, *J* = 8.8 Hz, 2H), 7.11 (dd, *J* = 8.8, 5.6 Hz, 2H), 6.95 (t, *J* = 8.8 Hz, 2H), 6.80 (d, *J* = 8.8 Hz, 2H), 5.56–5.47 (m, 3H), 5.01 (dd, *J* = 10.0, 2.4 Hz, 1H), 4.96 (d, *J* = 17.2 Hz, 1H), 3.88 (t, *J* = 6.4 Hz, 2H), 3.28 (d, *J* = 5.6 Hz, 2H), 2.11–1.92 (m, 3H), 1.83–1.68 (m, 2H), 1.63–1.46 (m, 2H), 1.42–1.29 (m, 2H); ^{13}C NMR (100 MHz, $CDCl_3$): δ 162.5, 160.1, 157.7, 142.5, 136.6, 131.9, 129.8, 129.7, 128.9, 125.3, 115.7, 115.13, 115.08, 114.9, 68.3, 43.4, 38.2, 34.7, 31.1, 30.0, 26.9; ^{19}F NMR (376 MHz, $CDCl_3$): δ –117.7 (s); MS (EI) *m/z* (relative intensity, %): 372 ($[M]^+$, 9), 154 (11), 149 (39), 148 (22), 147 (13), 135 (27), 128 (23), 109 (100); HRMS (EI) *m/z* $[M]^+$ Calcd for $C_{23}H_{26}ClFO$ 372.1659; Found 372.1659.

1-(4-Chlorophenoxyl)-3-(4-fluorophenyl)propane (4fa):

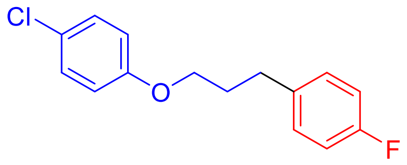

^{1}H NMR (400 MHz, $CDCl_3$): δ 7.21 (d, *J* = 9.2 Hz, 2H), 7.13 (dd, *J* =

8.4, 5.6 Hz, 2H), 6.96 (t, *J* = 8.4, 2H), 6.79 (d, *J* = 8.4 Hz, 2H), 3.89 (t, *J* = 6.4 Hz, 2H), 2.76 (t, *J* = 7.6 Hz, 2H), 2.05 (quin, *J* = 7.6 Hz, 2H); ^{13}C NMR (100 MHz, $CDCl_3$): δ 162.5, 160.1, 157.6, 136.95, 136.91, 129.9, 129.8, 129.3, 125.5, 115.7, 115.3, 115.1, 66.9, 31.3, 30.8; ^{19}F NMR (376 MHz, $CDCl_3$): δ –117.3; MS (EI) *m/z* (relative intensity, %): 264 ($[M]^+$, 37), 137 (19), 136 (19), 135 (13), 128 (19), 109 (100); HRMS (EI) *m/z* $[M]^+$ Calcd for $C_{15}H_{14}ClFO$ 264.0717; Found 264.0719.

The reaction of 4-(2-fluoroethyl)-1-tosylpiperidine (1g) with 4-fluorophenylmagnesium bromide (2a)

The representative general procedure was followed using 4-(2-fluoroethyl)-1-tosylpiperidine (285.7 mg, 1.00 mmol), 4-fluorophenylmagnesium bromide (in THF, 0.80 M, 2.5 mL, 2.0 mmol), and 1,3-butadiene (68 mL as gas, 3 mmol) in THF (total 3 mL) at 30 °C for 24 h. Purification by GPC gave the desired product **3ga** as a colorless oil (292.9 mg, 62%) accompanied by the cross-coupling product **4ga** as a white solid (50.3 mg, 14%).

(*E*)-4-[8-(4-Fluorophenyl)-3-vinyloct-6-en-1-yl]-1-tosylpiperidine (3ga):

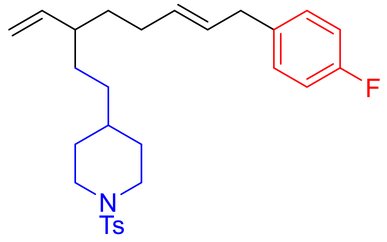

^{1}H NMR (400 MHz, $CDCl_3$): δ 7.64 (d, *J* = 8.0 Hz, 2H), 7.31 (d, *J* = 8.0 Hz, 2H), 7.10 (dd, *J* = 8.4, 5.2 Hz, 2H), 6.95 (t, *J* = 8.4 Hz, 2H), 5.54–5.40 (m, 3H), 4.97–4.86 (m, 2H), 3.75 (d, *J* = 11.2 Hz, 2H), 3.27 (d, *J* = 6.0 Hz, 2H), 2.43 (s, 3H), 2.18 (t, *J* = 12.0 Hz, 2H), 2.10-1.83 (m, 3H), 1.68 (d, *J* = 12.0 Hz, 2H), 1.45-1.05 (m, 9H); ^{13}C NMR (100 MHz, $CDCl_3$): δ 162.5, 160.0, 143.3, 142.7, 136.6, 136.5, 133.1, 131.9, 129.8, 129.7, 129.5, 128.7, 127.7, 115.1, 114.9, 114.7, 46.5, 43.7, 38.2, 35.2, 34.6, 33.5, 31.8, 31.6, 31.4, 30.0, 21.5; ^{19}F NMR (376 MHz, $CDCl_3$): δ –117.6 (s); MS (EI) *m/z* (relative intensity, %): 469 ($[M]^+$, 12), 315 (17), 314 (69), 262 (15), 249 (27), 236 (100); HRMS (EI) *m/z* $[M]^+$ Calcd for $C_{28}H_{36}FNO_2S$ 469.2451; Found 469.2449.

4-[2-(4-Fluorophenyl)ethyl]-1-tosylpiperidine (4ga):

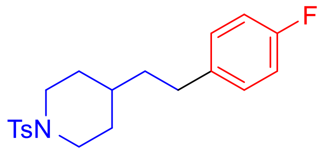

mp = 137–139 °C; ^{1}H NMR (400 MHz, $CDCl_3$): δ 7.63 (d, *J* = 8.0 Hz, 2H), 7.31 (d, *J* = 8.0 Hz, 2H), 7.07 (dd, *J* = 8.4, 6.8 Hz, 2H), 6.93 (t, *J* = 8.4 Hz, 2H), 3.76 (d, *J* = 12.0 Hz, 2H), 2.55 (t, *J* = 8.0 Hz, 2H), 2.42 (s, 3H), 2.18 (td, *J* = 12.0, 2.4 Hz, 2H), 1.75 (d, *J* = 12.4 Hz, 2H), 1.51 (dd, *J* = 15.6, 6.8 Hz, 2H), 1.33 (qd, *J* = 12.4, 3.6 Hz, 2H), 1.19-1.14 (m, 1H); ^{13}C NMR (100 MHz, $CDCl_3$): δ 162.3, 159.9, 143.3, 137.60, 137.57, 133.0, 129.5, 129.4, 127.7, 115.1, 114.9, 46.3, 37.7, 34.3, 31.9, 31.3, 21.5; ^{19}F NMR (376 MHz, $CDCl_3$): δ –117.6 (s); MS (EI) *m/z* (relative intensity, %): 361 ($[M]^+$, 30), 236 (13), 207 (15), 206 (100); HRMS (EI) *m/z* $[M]^+$ Calcd for $C_{20}H_{24}FNO_2S$ 361.1512; Found 361.1514.

The reaction of fluoromethylcyclohexane (1h) with 4-fluorophenylmagnesium bromide (2a)

The representative general procedure was followed in a 0.4 mmol scale using fluoromethylcyclohexane

(48.2 mg, 0.42 mmol), 4-fluorophenylmagnesium bromide (in THF, 0.80 M, 1.0 mL, 0.84 mmol), and 1,3-butadiene (28 mL as gas, 1.3 mmol) in THF (total 1.2 mL) at 30 °C for 24 h. Purification by GPC gave the desired product **3ha** as a colorless oil (39.1 mg, 31%) accompanied by the cross-coupling product **4ha** as a colorless oil (15.2 mg, 19%).

(*E*)-6-(Cyclohexylmethyl)-8-(4-fluorophenyl)octa-1,6-diene (3ha):

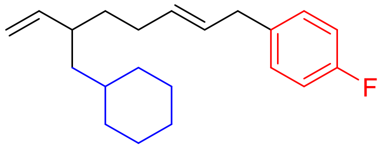

^{1}H NMR (400 MHz, $CDCl_3$): δ 7.11 (dd, *J* = 8.8, 5.6 Hz, 2H), 6.95 (t, *J* = 8.8 Hz, 2H), 5.51–5.42 (m, 3H), 4.96–4.88 (m, 2H), 3.28 (d, *J* = 6.0 Hz, 2H), 2.15–1.90 (m, 3H), 1.78–1.55 (m, 5H), 1.45–1.32 (m, 1H), 1.32–1.08 (m, 7H), 0.92–0.72 (m, 2H); ^{13}C NMR (100 MHz, $CDCl_3$): δ 162.5, 160.1, 143.4, 136.7, 136.6, 132.2, 131.4, 129.8, 129.7, 129.2, 128.7, 115.14, 115.11, 115.09, 114.94, 114.91, 114.88, 114.1, 43.0, 40.5, 38.1, 35.0, 34.7, 34.2, 32.7, 30.0, 26.7, 26.4, 26.3; ^{19}F NMR (376 MHz, $CDCl_3$): δ –117.7 (s); MS (EI) *m/z* (relative intensity, %): 300 ([M]$^+$, 4), 191 (10), 162 (31), 161 (13), 149 (44), 148 (42), 147 (34), 135 (27), 122 (42), 109 (100); HRMS (EI) *m/z* [M]$^+$ Calcd for $C_{21}H_{29}F$ 300.2253; Found 300.2254.

1-Fluoro-4-(cyclohexylmethyl)benzene (4ha):

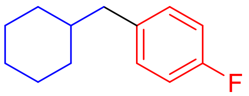

^{1}H NMR (400 MHz, $CDCl_3$): δ 7.07 (dd, *J* = 8.8, 6.0 Hz, 2H), 6.94 (t, *J* = 8.8 Hz, 2H), 2.44 (d, *J* = 7.2 Hz, 2H), 1.69–1.63 (m, 5H), 1.49–1.44 (m, 1H), 1.20–1.12 (m, 3H), 0.95-0.87 (m, 2H); ^{13}C NMR (100 MHz, $CDCl_3$): δ 162.3, 159.9, 136.9, 136.8, 130.4, 130.3, 128.6, 128.5, 127.4, 115.8, 115.6, 114.8, 114.6, 43.2, 39.8, 33.0, 26.5, 26.2; ^{19}F NMR (376 MHz, $CDCl_3$): δ –118.2 (s); MS (EI) *m/z* (relative intensity, %): 192 ([M]$^+$, 54), 111 (8), 110 (100), 109 (56); HRMS (EI) *m/z* [M]$^+$ Calcd for $C_{13}H_{17}F$ 192.1314; Found 192.1313.

The reaction of 1-fluorooctane (1a) with phenylmagnesium bromide (2b)

The representative general procedure was followed using 1-fluorooctane (125.5 mg, 0.95 mmol), phenylmagnesium bromide (in THF, 0.82 M, 2.4 mL, 2.0 mmol), and 1,3-butadiene (68 mL as gas, 3 mmol) in THF (total 3 mL) at 30 °C for 24 h. Purification by GPC gave the desired product **3ab** as a colorless oil (113.6 mg, 40%) accompanied by the cross-coupling product **4ab** as a colorless oil (99.7 mg, 55%).

(*E*)-1-Phenyl-6-vinyltetradec-2-ene (3ab):

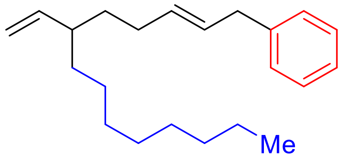

^{1}H NMR (400 MHz, $CDCl_3$): δ 7.30–7.25 (m, 2H), 7.20–7.17 (m, 3H), 5.56–5.46 (m, 3H), 4.97–4.89 (m, 2H), 3.32 (d, *J* = 6.0 Hz, 2H), 2.10–1.90 (m, 3H), 1.48–1.38 (m, 1H), 1.35–1.17 (m, 15H), 0.88 (t, *J* = 6.8 Hz, 3H); ^{13}C NMR (100 MHz, $CDCl_3$): δ 143.3, 141.1, 132.0, 128.7, 128.4, 128.3, 125.8, 114.2, 43.6, 39.1, 35.0, 34.7, 31.9, 30.1, 29.7, 29.6, 29.3, 27.1, 22.7, 14.1; MS (DART) *m/z* (relative intensity, %): 299 ([M+H]$^+$, 92), 239 (31), 195 (30), 131 (36); HRMS (DART) *m/z* [M+H]$^+$ Calcd for $C_{22}H_{35}$ 299.2739; Found 299.2716.

Octylbenzene (4ab):[S11]

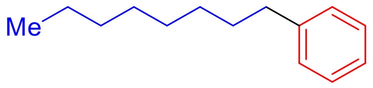

^{1}H NMR (400 MHz, $CDCl_3$): δ 7.29–7.25 (m, 2H), 7.18–7.17 (m, 3H), 2.60 (t, *J* = 7.6 Hz, 2H), 1.61 (quin, *J* = 7.6 Hz, 2H), 1.35–1.20 (m, 10H), 0.88 (t, *J* = 6.8 Hz, 3H); ^{13}C NMR (100 MHz, $CDCl_3$): δ 143.0, 128.4, 128.2, 125.5, 36.0, 31.9, 31.5, 29.5, 29.4, 29.3, 22.7, 14.1; MS (DART) *m/z* (relative intensity, %): 191 ($[M+H]^+$, 15), 187 (90), 185 (100), 131 (42), 117 (38), 105 (75); HRMS (DART) *m/z* $[M+H]^+$ Calcd for $C_{14}H_{23}$ 191.1800; Found 191.1782.

The reaction of 1-fluorooctane (1a) with 4-methylphenylmagnesium bromide (2c)

The representative general procedure was followed using 1-fluorooctane (133.0 mg, 1.01 mmol), 4-methylphenylmagnesium bromide (in THF, 0.73 M, 2.7 mL, 2.0 mmol), and 1,3-butadiene (68 mL as gas, 3 mmol) in THF (total 3 mL) at 30 °C for 24 h. Purification by GPC gave the desired product **3ac** as a pale yellow oil (109.0 mg, 34%) accompanied by the cross-coupling product **4ac** as a colorless oil (136.9 mg, 66%).

(*E*)-1-(4-Methylphenyl)-6-vinyltetradec-2-ene (3ac):

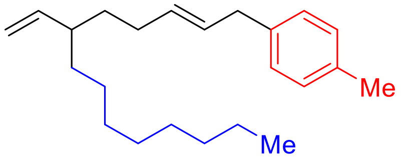

^{1}H NMR (400 MHz, $CDCl_3$): δ 7.09 (d, *J* = 8.0 Hz, 2H), 7.06 (d, *J* = 8.0 Hz, 2H), 5.56–5.45 (m, 3H), 4.96–4.90 (m, 2H), 3.28 (d, *J* = 6.0 Hz, 2H), 2.32 (s, 3H), 2.10–1.89 (m, 3H), 1.48–1.38 (m, 1H), 1.35–1.17 (m, 15H), 0.88 (t, *J* = 6.8 Hz, 3H); ^{13}C NMR (100 MHz, $CDCl_3$): δ 143.3, 138.0, 135.3, 131.7, 129.02, 128.99, 128.3, 114.2, 43.6, 38.6, 35.0, 34.7, 31.9, 30.1, 29.7, 29.6, 29.3, 27.1, 22.7, 21.0, 14.1; MS (DART) *m/z* (relative intensity, %): 313 ($[M+H]^+$, 100), 311 (70), 195 (33), 147 (77); HRMS (DART) *m/z* $[M+H]^+$ Calcd for $C_{23}H_{37}$ 313.2895; Found 313.2874.

4-Methyl-1-octylbenzene (4ac):[S11]

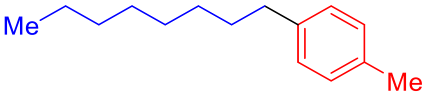

^{1}H NMR (400 MHz, $CDCl_3$): δ 7.07 (t, *J* = 8.8 Hz, 4H), 2.55 (t, *J* = 8.0 Hz, 2H), 2.31 (s, 3H), 1.58 (quin, *J* = 7.6 Hz, 2H), 1.35–1.20 (m, 10H), 0.88 (t, *J* = 6.8 Hz, 3H); ^{13}C NMR (100 MHz, $CDCl_3$): δ 139.9, 134.9, 128.9, 128.3, 35.5, 31.9, 31.7, 29.5, 29.4, 29.3, 22.7, 21.0, 14.1; MS (DART) *m/z* (relative intensity, %): 195 ($[M+H]^+$, 100), 151 (46), 133 (22); HRMS (DART) *m/z* $[M+H]^+$ Calcd for $C_{15}H_{25}$ 205.1956; Found 205.1979.

The reaction of 1-fluorooctane (1a) with 4-methoxyphenylmagnesium bromide (2d):

The representative general procedure was followed using 1-fluorooctane (126.0 mg, 0.95 mmol), 4-methoxyphenylmagnesium bromide (in THF, 0.67 M, 3.0 mL, 2.0 mmol), and 1,3-butadiene (68 mL as gas, 3 mmol) in THF (total 3 mL) at 30 °C for 24 h. Purification by GPC gave the desired product **3ad** as a colorless oil (88.3 mg, 28%) accompanied by the cross-coupling product **4ad** as a colorless oil (134.9 mg, 64%).

(*E*)-1-(4-Methoxyphenyl)-6-vinyltetradec-2-ene (3ad):

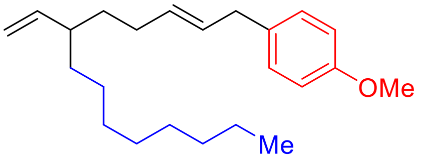

^{1}H NMR (400 MHz, $CDCl_3$): δ 7.09 (d, *J* = 8.8 Hz, 2H), 6.83 (d, *J* = 8.8 Hz, 2H), 5.55–5.44 (m, 3H), 4.97–4.90 (m, 2H), 3.78 (s, 3H), 3.26

(d, *J* = 6.4 Hz, 2H), 2.10–1.89 (m, 3H), 1.48–1.38 (m, 1H), 1.35–1.17 (m, 15H), 0.88 (t, *J* = 6.8 Hz, 3H); ^{13}C NMR (100 MHz, $CDCl_3$): δ 157.8, 143.3, 133.1, 131.6, 129.3, 129.2, 114.2, 113.7, 55.2, 43.6, 38.1, 35.0, 34.7, 31.2, 30.1, 29.7, 29.6, 29.3, 27.1, 22.7, 14.1; MS (DART) *m/z* (relative intensity, %): 329 ($[M+H]^+$, 100), 227 (10), 121 (63); HRMS (DART) *m/z* $[M+H]^+$ Calcd for $C_{23}H_{37}O$ 329.2844; Found 329.2821.

1-Methoxy-4-octylbenzene (4ad):[S12]

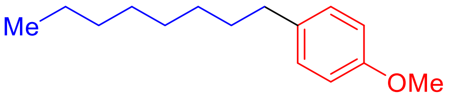

^{1}H NMR (400 MHz, $CDCl_3$): δ 7.09 (d, *J* = 8.4 Hz, 2H), 6.82 (d, *J* = 8.4 Hz, 2H), 3.78 (s, 3H), 2.54 (t, *J* = 8.0 Hz, 2H), 1.60–1.53 (m, 2H), 1.35–1.20 (m, 10H), 0.88 (t, *J* = 6.8 Hz, 3H); ^{13}C NMR (100 MHz, $CDCl_3$): δ 157.5, 135.0, 129.2, 113.6, 55.2, 35.0, 31.9, 31.8, 29.5, 29.3 (2C), 22.7, 14.1; MS (DART) *m/z* (relative intensity, %): 221 ($[M+H]^+$, 100), 195 (29), 121 (31); HRMS (DART) *m/z* $[M+H]^+$ Calcd for $C_{15}H_{25}O$ 221.1905; Found 221.1906.

The reaction of 1-fluorooctane (1a) with 4-chlorophenylmagnesium bromide (2e)

The representative general procedure was followed using 1-fluorooctane (128.3 mg, 0.97 mmol), 4-chlorophenylmagnesium bromide (in THF, 0.65 M, 3.1 mL, 2.0 mmol), and 1,3-butadiene (68 mL as gas, 3 mmol) in THF (total 3 mL) at 30 °C for 24 h. Purification by GPC gave the desired product **3ae** as a colorless oil (69.5 mg, 22%) accompanied by the cross-coupling product **4ae** as a colorless oil (47.8 mg, 22%).

(*E*)-1-(4-Chlorophenyl)-6-vinyltetradec-2-ene (3ae):

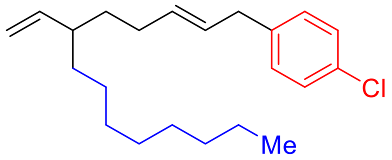

^{1}H NMR (400 MHz, $CDCl_3$): δ 7.23 (d, *J* = 8.0 Hz, 2H), 7.09 (d, *J* = 8.0 Hz, 2H), 5.54–5.45 (m, 3H), 4.97–4.89 (m, 2H), 3.27 (d, *J* = 4.8 Hz, 2H), 2.10–1.90 (m, 3H), 1.48–1.38 (m, 1H), 1.35–1.17 (m, 15H), 0.88 (t, *J* = 6.8 Hz, 3H); ^{13}C NMR (100 MHz, $CDCl_3$): δ 143.2, 139.5, 132.5, 131.6, 129.8, 128.4, 128.2, 114.3, 43.6, 38.3, 35.0, 34.6, 31.9, 30.1, 29.7, 29.6, 29.3, 27.1, 22.7, 14.1; MS (DART) *m/z* (relative intensity, %): 333 ($[M+H]^+$, 70), 297 (69), 167 (100); HRMS (DART) *m/z* $[M+H]^+$ Calcd for $C_{22}H_{34}Cl$ 333.2349; Found 333.2362.

1-Chloro-4-octylbenzene (4ae):[S11]

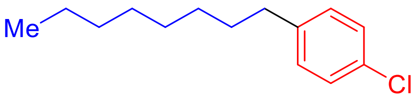

^{1}H NMR (400 MHz, $CDCl_3$): δ 7.23 (d, *J* = 8.0 Hz, 2H), 7.09 (d, *J* = 8.0 Hz, 2H), 2.56 (t, *J* = 8.0 Hz, 2H), 1.60–1.53 (m, 2H), 1.35–1.20 (m, 10H), 0.88 (t, *J* = 6.8 Hz, 3H); ^{13}C NMR (100 MHz, $CDCl_3$): δ 141.3, 131.2, 129.7, 128.3, 35.3, 31.9, 31.4, 29.4, 29.23, 29.18, 22.7, 14.1; MS (DART) *m/z* (relative intensity, %): 225 ($[M+H]^+$, 1), 221 (100); HRMS (DART) *m/z* $[M+H]^+$ Calcd for $C_{14}H_{22}Cl$ 225.1410; Found 225.1401.

The reaction of 1-fluorooctane (1a) with 4-dimethylaminophenylmagnesium bromide (2f)

The representative general procedure was followed using 1-fluorooctane (132.0 mg, 1.00 mmol),

4-dimethylaminophenylmagnesium bromide (in THF, 0.52 M, 3.8 mL, 2.0 mmol), and 1,3-butadiene (68 mL as gas, 3 mmol) in THF (total 3 mL) at 30 °C for 24 h. Purification by GPC gave the desired product **3af** as a yellow oil (79.7 mg, 23%) accompanied by the cross-coupling product **4af** as a pale yellow oil (180.5 mg, 77%).

(*E*)-1-(4-Dimethylaminophenyl)-6-vinyltetradec-2-ene (3af):

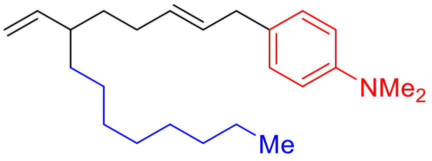

^{1}H NMR (400 MHz, $CDCl_3$): δ 7.05 (d, *J* = 8.4 Hz, 2H), 6.70 (d, *J* = 8.4 Hz, 2H), 5.55–5.43 (m, 3H), 4.97–4.90 (m, 2H), 3.22 (d, *J* = 6.0 Hz, 2H), 2.90 (s, 6H), 2.10–1.90 (m, 3H), 1.48–1.38 (m, 1H), 1.35–1.17 (m, 15H), 0.88 (t, *J* = 6.8 Hz, 3H); ^{13}C NMR (100 MHz, $CDCl_3$): δ 149.1, 143.3, 131.2, 129.6, 129.3, 129.0, 114.1, 113.1, 43.6, 40.9, 38.0, 35.0, 34.8, 31.9, 30.1, 29.8, 29.6, 29.3, 27.1, 22.7, 14.1; MS (DART) *m/z* (relative intensity, %): 342 ($[M+H]^+$, 100), 332 (14), 176 (16); HRMS (DART) *m/z* $[M+H]^+$ Calcd for $C_{24}H_{40}N$ 342.3161; Found 342.3152.

1-Dimethylamino-4-octylbenzene (4af):[S13]

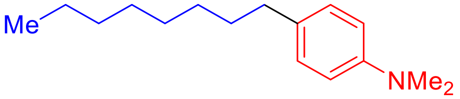

^{1}H NMR (400 MHz, $CDCl_3$): δ 7.05 (d, *J* = 8.4 Hz, 2H), 6.70 (d, *J* = 8.4 Hz, 2H), 2.90 (s, 6H), 2.50 (t, *J* = 7.6 Hz, 2H), 1.56 (quint, *J* = 7.6 Hz, 2H), 1.35–1.22 (m, 10H), 0.88 (t, *J* = 6.8 Hz, 3H); ^{13}C NMR (100 MHz, $CDCl_3$): δ 148.9, 131.3, 128.9, 113.0, 41.0, 34.9, 31.9, 31.8, 29.5, 29.4, 29.3, 22.7, 14.1; MS (DART) *m/z* (relative intensity, %): 234 ($[M+H]^+$, 100), 230 (25); HRMS (DART) *m/z* $[M+H]^+$ Calcd for $C_{16}H_{28}N$ 234.2222; Found 234.2227.

The reaction of 1-fluorooctane (1a) with 3-methylphenylmagnesium bromide (2g)

The representative general procedure was followed using 1-fluorooctane (129.7 mg, 0.98 mmol), 3-methylphenylmagnesium bromide (in THF, 0.83 M, 2.4 mL, 2.0 mmol), and 1,3-butadiene (68 mL as gas, 3 mmol) in THF (total 3 mL) at 30 °C for 24 h. Purification by GPC gave the desired product **3ag** as a colorless oil (105.7 mg, 35%) accompanied by the cross-coupling product **4ag** as a colorless oil (122.6 mg, 61%).

(*E*)-1-(3-Methylphenyl)-6-vinyltetradec-2-ene (3ag):

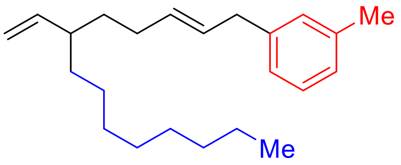

^{1}H NMR (400 MHz, $CDCl_3$): δ 7.17 (t, *J* = 7.6 Hz, 1H), 7.00–6.97 (m, 3H), 5.56–5.46 (m, 3H), 4.97–4.89 (m, 2H), 3.28 (d, *J* = 6.0 Hz, 2H), 2.32 (s, 3H), 2.10–1.90 (m, 3H), 1.48–1.38 (m, 1H), 1.35–1.17 (m, 15H), 0.88 (t, *J* = 6.8 Hz, 3H); ^{13}C NMR (100 MHz, $CDCl_3$): δ 143.3, 141.0, 137.9, 131.8, 129.2, 128.9, 128.2, 126.6, 125.4, 114.2, 43.6, 39.0, 35.0, 34.7, 31.9, 30.1, 29.8, 29.6, 29.3, 27.1, 22.7, 21.4, 14.1; MS (DART) *m/z* (relative intensity, %): 313 ($[M+H]^+$, 100), 195 (19), 156 (35), 105 (27); HRMS (DART) *m/z* $[M+H]^+$ Calcd for $C_{23}H_{37}$ 313.2895; Found 313.2880.

3-Methyl-1-octylbenzene (4ag):[S11]

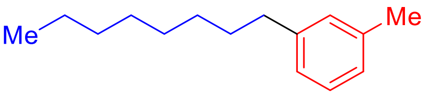

^{1}H NMR (400 MHz, $CDCl_3$): δ 7.16 (t, *J* = 7.6 Hz, 1H), 6.99–6.97

(m, 3H), 2.55 (t, J = 7.6 Hz, 2H), 2.32 (s, 3H), 1.59 (quin, J = 7.6 Hz, 2H), 1.35–1.22 (m, 10H), 0.88 (t, J = 6.8 Hz, 3H); ^{13}C NMR (100 MHz, $CDCl_3$): δ 142.9, 137.7, 129.2, 128.1, 126.3, 125.4, 35.9, 31.9, 31.6, 29.5, 29.4, 29.3, 22.7, 21.4, 14.1; MS (DART) m/z (relative intensity, %): 195 ($[M+H]^+$, 11), 145 (20), 131 (17), 119 (19), 105 (42); HRMS (DART) m/z $[M+H]^+$ Calcd for $C_{15}H_{25}$ 205.1956; Found 205.1975.

The reaction of 1-fluorooctane (1a) with 3-methoxyphenylmagnesium bromide (2h)

The representative general procedure was followed using 1-fluorooctane (130.5 mg, 0.99 mmol), 3-methoxyphenylmagnesium bromide (in THF, 0.97 M, 2.1 mL, 2.0 mmol), and 1,3-butadiene (68 mL as gas, 3 mmol) in THF (total 3 mL) at 30 °C for 24 h. Purification by GPC gave the desired product **3ah** as a colorless oil (131.6 mg, 40%) accompanied by the cross-coupling product **4ah** as a colorless oil (131.3 mg, 60%).

(*E*)-1-(3-Methoxyphenyl)-6-vinyltetradec-2-ene (3ah):

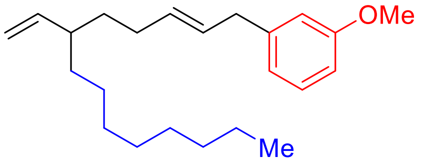

^{1}H NMR (400 MHz, $CDCl_3$): δ 7.22–7.18 (m, 1H), 6.77 (d, J = 8.0 Hz, 1H), 6.74–6.73 (m, 2H), 5.57–5.46 (m, 3H), 4.97–4.89 (m, 2H), 3.79 (s, 3H), 3.30 (d, J = 5.6 Hz, 2H), 2.11–1.90 (m, 3H), 1.48–1.38 (m, 1H), 1.35–1.17 (m, 15H), 0.88 (t, J = 6.8 Hz, 3H); ^{13}C NMR (100 MHz, $CDCl_3$): δ 159.6, 143.2, 142.8, 132.1, 129.2, 128.5, 120.9, 114.21, 114.16, 111.1, 55.1, 43.6, 39.1, 35.0, 34.7, 31.9, 30.1, 29.8, 29.6, 29.3, 27.1, 22.7, 14.1; MS (DART) m/z (relative intensity, %): 329 ($[M+H]^+$, 100), 206 (15), 147 (12), 121 (41); HRMS (DART) m/z $[M+H]^+$ Calcd for $C_{23}H_{37}O$ 329.2844; Found 329.2820.

1-Methoxy-3-octylbenzene (4ah):[S14]

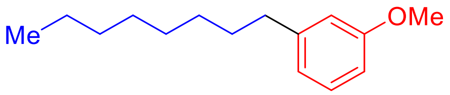

^{1}H NMR (400 MHz, $CDCl_3$): δ 7.19 (t, J = 8.0 Hz, 1H), 6.77 (d, J = 8.0 Hz, 1H), 6.73–6.71 (m, 2H), 3.79 (s, 3H), 2.57 (t, J = 8.0 Hz, 2H), 1.62–1.58 (m, 2H), 1.35–1.22 (m, 10H), 0.88 (t, J = 6.8 Hz, 3H); ^{13}C NMR (100 MHz, $CDCl_3$): δ 159.5, 144.6, 129.1, 120.8, 114.1, 110.8, 55.1, 36.0, 31.9, 31.4, 29.5, 29.34, 29.25, 22.7, 14.1; MS (DART) m/z (relative intensity, %): 221 ($[M+H]^+$, 100), 217 (17), 121 (11); HRMS (DART) m/z $[M+H]^+$ Calcd for $C_{15}H_{25}O$ 221.1905; Found 221.1908.

The reaction of 4-(2-fluoroethyl)-1-tosylpiperidine (1g) with phenylmagnesium bromide (2b)

The representative general procedure was followed in 0.5 mmol scale using 4-(2-fluoroethyl)-1-tosylpiperidine (142.5 mg, 0.50 mmol), phenylmagnesium bromide (in THF, 0.82 M, 1.2 mL, 1.0 mmol), and 1,3-butadiene (34 mL as gas, 1.5 mmol) in THF (total 1.5 mL) at 30 °C for 42 h. Purification by GPC gave the desired product **3gb** as a colorless oil (107.8 mg, 48%) accompanied by the cross-coupling product **4gb** as a white solid (40.2 mg, 23%).

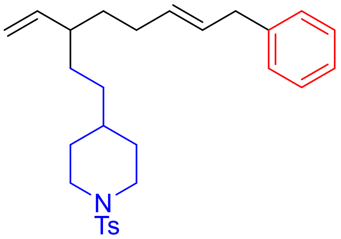

(*E*)-4-(8-Phenyl-3-vinyloct-6-en-1-yl)-1-tosylpiperidine (3gb):

^{1}H NMR (400 MHz, $CDCl_3$): δ 7.63 (d, *J* = 8.4 Hz, 2H), 7.31 (d, *J* = 8.4 Hz, 2H), 7.28 (d, *J* = 7.6 Hz, 2H), 7.25–7.15 (m, 3H), 5.52–5.42 (m, 3H), 4.96–4.86 (m, 2H), 3.75 (d, *J* = 11.6 Hz, 2H), 3.30 (d, *J* = 6.0 Hz, 2H), 2.43 (s, 3H), 2.18 (t, *J* = 11.6 Hz, 2H), 2.10–1.83 (m, 3H), 1.68 (d, *J* = 12.8 Hz, 2H), 1.45–1.03 (m, 9H); ^{13}C NMR (100 MHz, $CDCl_3$): δ 143.3, 142.7, 141.0, 133.1, 131.7, 129.5, 128.9, 128.4, 128.3, 127.7, 125.8, 114.7, 46.5, 43.6, 39.0, 35.2, 34.6, 33.5, 31.8, 31.6, 31.4, 30.0, 21.5; MS (EI) *m/z* (relative intensity, %): 451 ($[M]^+$, 15), 297 (20), 296 (87), 262 (17), 249 (28), 237 (14), 236 (100); HRMS (EI) *m/z* $[M]^+$ Calcd for $C_{28}H_{37}NO_2S$ 451.2545; Found 451.2547.

4-(2-Phenylethyl)-1-tosylpiperidine (4gb):

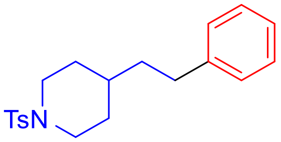

mp = 130–131 °C; ^{1}H NMR (400 MHz, $CDCl_3$): δ 7.63 (d, *J* = 8.4 Hz, 2H), 7.31 (d, *J* = 8.4 Hz, 2H), 7.27 (d, *J* = 8.4 Hz, 2H), 7.23–7.12 (m, 3H), 3.76 (d, *J* = 11.2 Hz, 2H), 2.58 (t, *J* = 8.0 Hz, 2H), 2.43 (s, 3H), 2.19 (td, *J* = 12.0, 2.4 Hz, 2H), 1.76 (dd, *J* = 12.8, 2.0 Hz, 2H), 1.58–1.52 (m, 2H), 1.34 (qd, *J* = 11.6, 4.0 Hz, 2H), 1.19–1.14 (m, 1H); ^{13}C NMR (100 MHz, $CDCl_3$): δ 143.4, 142.1, 133.0, 129.5, 128.4, 128.3, 127.7, 125.8, 46.4, 37.7, 34.4, 32.7, 31.4, 21.5; MS (EI) *m/z* (relative intensity, %): 343 ($[M]^+$, 39), 236 (11), 189 (15), 188 (100); HRMS (EI) *m/z* $[M]^+$ Calcd for $C_{20}H_{25}NO_2S$ 343.1606; Found 343.1603.

The reaction of 4-(2-fluoroethyl)-1-tosylpiperidine (1g) with 4-methylphenylmagnesium bromide (2c)

The representative general procedure was followed in 0.5 mmol scale using 4-(2-fluoroethyl)-1-tosylpiperidine (142.5 mg, 0.50 mmol), 4-methylphenylmagnesium bromide (in THF, 0.73 M, 1.4 mL, 1.0 mmol), and 1,3-butadiene (34 mL as gas, 1.5 mmol) in THF (total 1.5 mL) at 30 °C for 42 h. Purification by GPC gave the desired product **3gc** as a colorless oil (88.7 mg, 38%) accompanied by the cross-coupling product **4gc** as a white solid (39.3 mg, 22%).

(*E*)-4-[8-(4-Methylphenyl)-3-vinyloct-6-en-1-yl]-1-tosylpiperidine (3gc):

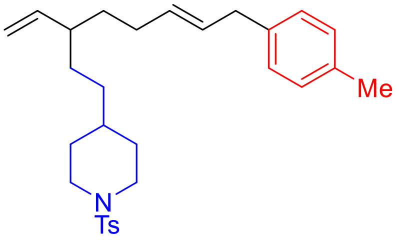

^{1}H NMR (400 MHz, $CDCl_3$): δ 7.63 (d, *J* = 8.0 Hz, 2H), 7.31 (d, *J* = 8.0 Hz, 2H), 7.09 (d, *J* = 8.0 Hz, 2H), 7.05 (d, *J* = 8.0 Hz, 2H), 5.50–5.42 (m, 3H), 4.96–4.86 (m, 2H), 3.75 (d, *J* = 12.4 Hz, 2H), 3.26 (d, *J* = 6.8 Hz, 2H), 2.43 (s, 3H), 2.31 (s, 3H), 2.18 (td, *J* = 12.0, 2.0 Hz, 2H), 2.08–1.83 (m, 3H), 1.68 (d, *J* = 12.8 Hz, 2H), 1.43–1.03 (m, 9H); ^{13}C NMR (100 MHz, $CDCl_3$): δ 143.3, 142.8, 137.9, 135.3, 133.1, 131.5, 129.5, 129.2, 129.0, 128.3, 127.7, 114.7, 46.5, 43.7, 38.6, 35.2, 34.7, 33.5, 31.8, 31.6, 31.4, 30.1, 21.5, 21.0; MS (EI) *m/z* (relative intensity, %): 465 ($[M]^+$, 12), 311 (25), 310 (100); HRMS (EI) *m/z* $[M]^+$ Calcd for $C_{29}H_{39}NO_2S$ 465.2702; Found 465.2698.

4-[2-(4-Methylphenyl)ethyl]-1-tosylpiperidine (4gc):

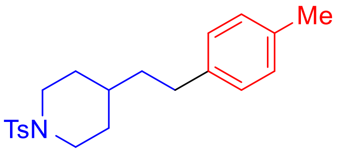

mp = 135–137 °C; ^{1}H NMR (400 MHz, $CDCl_3$): δ 7.63 (d, *J* = 8.0 Hz, 2H), 7.31 (d, *J* = 8.0 Hz, 2H), 7.06 (d, *J* = 8.0 Hz, 2H), 7.01 (d, *J* = 8.0 Hz, 2H),

3.76 (d, J = 12.0 Hz, 2H), 2.54 (t, J = 8.0 Hz, 2H), 2.42 (s, 3H), 2.29 (s, 3H), 2.18 (td, J = 12.0, 2.4 Hz, 2H), 1.75 (dd, J = 12.8, 2.4 Hz, 2H), 1.64–1.49 (m, 2H), 1.33 (qd, J = 12.0, 4.0 Hz, 2H), 1.19–1.14 (m, 1H); ^{13}C NMR (100 MHz, $CDCl_3$): δ 143.3, 138.9, 135.2, 133.0, 129.5, 129.0, 128.1, 127.7, 46.4, 37.7, 34.3, 32.2, 31.3, 21.5, 20.9; MS (EI) *m/z* (relative intensity, %): 357 ([M]$^+$, 21), 236 (11), 203 (16), 202 (100); HRMS (EI) *m/z* [M]$^+$ Calcd for $C_{21}H_{27}NO_2S$ 357.1762; Found 357.1765.

The reaction of 4-(2-fluoroethyl)-1-tosylpiperidine (1g) with 3-methylphenylmagnesium bromide (2g)

The representative general procedure was followed in 0.5 mmol scale using 4-(2-fluoroethyl)-1-tosylpiperidine (143.0 mg, 0.50 mmol), 3-methylphenylmagnesium bromide (in THF, 0.83 M, 1.2 mL, 1.0 mmol), and 1,3-butadiene (34 mL as gas, 1.5 mmol) in THF (total 1.5 mL) at 30 °C for 42 h. Purification by GPC gave the desired product **3gg** as a colorless oil (107.1 mg, 46%) accompanied by the cross-coupling product **4gg** as a white solid (44.3 mg, 25%).

(*E*)-4-[8-(3-Methylphenyl)-3-vinyloct-6-en-1-yl]-1-tosylpiperidine (3gg):

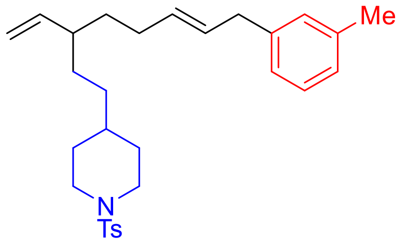

^{1}H NMR (400 MHz, $CDCl_3$): δ 7.63 (d, J = 8.4 Hz, 2H), 7.31 (d, J = 8.4 Hz, 2H), 7.15 (d, J = 7.6 Hz, 1H), 7.00–6.95 (m, 3H), 5.51–5.45 (m, 3H), 4.97–4.87 (m, 2H), 3.75 (d, J = 11.6 Hz, 2H), 3.26 (d, J = 6.4 Hz, 2H), 2.43 (s, 3H), 2.32 (s, 3H), 2.18 (td, J = 12.4, 2.0 Hz, 2H), 2.04–1.83 (m, 3H), 1.68 (d, J = 12.8 Hz, 2H), 1.43–1.03 (m, 9H); ^{13}C NMR (100 MHz, $CDCl_3$): δ 143.3, 142.7, 140.9, 137.9, 133.1, 131.6, 129.5, 129.2, 129.0, 128.2, 127.7, 126.6, 125.4, 114.7, 46.5, 43.6, 39.0, 35.2, 34.6, 33.5, 31.8, 31.6, 31.4, 30.0, 21.5, 21.4; MS (EI) *m/z* (relative intensity, %): 465 ([M]$^+$, 12), 311 (25), 310 (100); HRMS (EI) *m/z* [M]$^+$ Calcd for $C_{29}H_{39}NO_2S$ 465.2702; Found 465.2703.

4-[2-(3-Methylphenyl)ethyl]-1-tosylpiperidine (4gg):

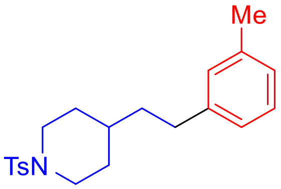

mp = 104–106 °C; ^{1}H NMR (400 MHz, $CDCl_3$): δ 7.63 (d, J = 8.0 Hz, 2H), 7.31 (d, J = 8.0 Hz, 2H), 7.14 (t, J = 7.6 Hz, 1H), 6.98–6.91 (m, 3H), 3.75 (d, J = 11.6 Hz, 2H), 2.54 (t, J = 7.6 Hz, 2H), 2.42 (s, 3H), 2.30 (s, 3H), 2.19 (td, J = 12.0, 2.4 Hz, 2H), 1.76 (dd, J = 12.8, 2.4 Hz, 2H), 1.60–1.50 (m, 2H), 1.33 (qd, J = 12.0, 4.0 Hz, 2H), 1.21–1.14 (m, 1H); ^{13}C NMR (100 MHz, $CDCl_3$): δ 143.3, 142.0, 137.9, 133.0, 129.5, 129.0, 128.2, 127.7, 126.5, 125.2, 46.4, 37.6, 34.4, 32.6, 31.3, 21.5, 21.3; MS (EI) *m/z* (relative intensity, %): 357 ([M]$^+$, 29), 236 (15), 203 (16), 202 (100); HRMS (EI) *m/z* [M]$^+$ Calcd for $C_{21}H_{27}NO_2S$ 357.1762; Found 357.1760.

The reaction of 4-(2-fluoroethyl)-1-tosylpiperidine (1g) with 2-thienylmagnesium bromide (2i)

The representative general procedure was followed in 0.5 mmol scale using 4-(2-fluoroethyl)-1-tosylpiperidine (142.6 mg, 0.50 mmol), 2-thienylmaggnesium bromide (in THF, 0.92

M, 1.1 mL, 1.0 mmol), and 1,3-butadiene (34 mL as gas, 1.5 mmol) in THF (total 1.5 mL) at 30 °C for 42 h. Purification by GPC gave cross-coupling product **4gi** as a white solid (39.8 mg, 23%) and unreacted **1g** (62.5 mg, 44%).

4-[2-(2-Thienyl)ethyl]-1-tosylpiperidine (4gi):

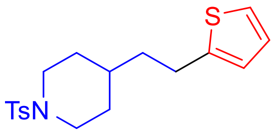

mp = 84–85 °C; ^{1}H NMR (400 MHz, $CDCl_3$): δ 7.63 (d, *J* = 8.0 Hz, 2H), 7.31 (d, *J* = 8.0 Hz, 2H), 7.08 (dd, *J* = 4.8, 1.2 Hz, 1H), 6.88 (dd, *J* = 4.8, 3.2 Hz, 1H), 6.73 (dd, *J* = 3.2, 1.2 Hz, 1H), 3.76 (d, *J* = 12.0 Hz, 2H), 2.81 (t, *J* = 7.6 Hz, 2H), 2.43 (s, 3H), 2.19 (td, *J* = 12.0, 2.4 Hz, 2H), 1.75 (dd, *J* = 12.8, 2.4 Hz, 2H), 1.61 (q, *J* = 7.2 Hz, 2H), 1.34 (qd, *J* = 12.8, 4.0 Hz, 2H), 1.25–1.16 (m, 1H); ^{13}C NMR (100 MHz, $CDCl_3$): δ 144.8, 143.3, 132.9, 129.5, 127.6, 126.6, 124.0, 122.9, 46.3, 37.7, 34.1, 31.2, 26.8, 21.5; MS (EI) *m/z* (relative intensity, %): 349 ($[M]^+$, 1), 285 (42), 284 (33), 266 (59), 155 (43), 130 (100); HRMS (EI) *m/z* $[M]^+$ Calcd for $C_{18}H_{23}NO_2S_2$ 349.1170; Found 349.1168.

The reaction of 4-(2-fluoroethyl)-1-tosylpiperidine (1g) with 2-methylphenylmagnesium bromide (2j)

The representative general procedure was followed in 0.5 mmol scale using 4-(2-fluoroethyl)-1-tosylpiperidine (143.6 mg, 0.50 mmol), 2-methylphenylmagnesium bromide (in THF, 0.82 M, 1.2 mL, 1.0 mmol), and 1,3-butadiene (34 mL as gas, 1.5 mmol) in THF (total 1.5 mL) at 30 °C for 42 h. Purification by GPC gave the desired product **3gj** as a colorless oil (200.2 mg, 85%).

(*E*)-4-[8-(2-Methylphenyl)-3-vinyloct-6-en-1-yl]-1-tosylpiperidine (3gj): [S1]

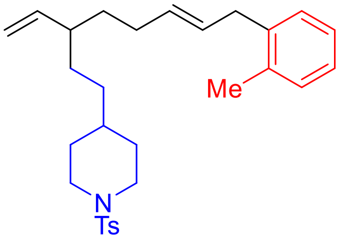

^{1}H NMR (400 MHz, $CDCl_3$): δ 7.63 (d, *J* = 8.4 Hz, 2H), 7.31 (d, *J* = 8.4 Hz, 2H), 7.11 (br s, 4H), 5.52–5.35 (m, 3H), 4.96–4.85 (m, 2H), 3.74 (d, *J* = 11.2 Hz, 2H), 3.27 (d, *J* = 6.4 Hz, 2H), 2.42 (s, 3H), 2.27 (s, 3H), 2.17 (td, *J* = 12.0, 2.0 Hz, 2H), 2.04–1.81 (m, 3H), 1.68 (d, *J* = 12.8 Hz, 2H), 1.42–1.01 (m, 9H); ^{13}C NMR (100 MHz, $CDCl_3$): δ 143.3, 142.7, 139.0, 136.2, 133.0, 131.5, 130.0, 129.5, 128.8, 128.0, 127.7, 126.0, 125.9, 114.6, 46.5, 43.6, 36.5, 35.1, 34.6, 33.5, 31.7, 31.6, 31.3, 30.1, 21.5, 19.3; MS (EI) *m/z* (relative intensity, %): 465 ($[M]^+$, 16), 311 (24), 310 (97), 262 (21), 249 (42), 237 (14), 236 (100); HRMS (EI) *m/z* $[M]^+$ Calcd for $C_{29}H_{39}NO_2S$ 465.2699; Found 465.2698.

(*E*)-1-(2-Methylphenyl)-6-vinyltetradec-2-ene (3aj): [S1]

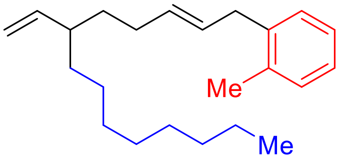

Following the general procedure, reaction was conducted in 1 mmol scale using 1-fluorooctane (130.4 mg, 0.99 mmol) and 2-methylphenylmagnesium bromide (in THF, 0.82 M, 1.83 mL, 1.5 mmol). Purification by GPC gave the desired product as a colorless oil (265.1 mg, 86%): ^{1}H NMR (400 MHz, $CDCl_3$): δ 7.12–7.10 (m, 4H), 5.54–5.38 (m, 3H), 4.96–4.88 (m, 2H), 3.29 (d, *J* = 6.0 Hz, 2H), 2.28 (s,

3H), 2.08–1.87 (m, 3H), 1.47–1.17 (m, 16H), 0.88 (t, J = 6.8 Hz, 3H): ^{13}C NMR (100 MHz, $CDCl_3$): δ 143.3, 139.1, 136.2, 131.8, 130.0, 128.9, 127.9, 126.0, 125.9, 114.2, 43.6, 36.6, 35.0, 34.8, 31.9, 30.2, 29.7, 29.6, 29.3, 27.1, 22.7, 19.3, 14.1: MS (DART) *m/z* (relative intensity, %): 313 ([M+H]$^+$, 5), 283 (60), 265 (32), 225 (20), 195 (13), 118 (56), 102 (100): HRMS (DART) *m/z* [M+H]$^+$ Calcd for $C_{23}H_{37}$ 313.2895; Found 313.2912.

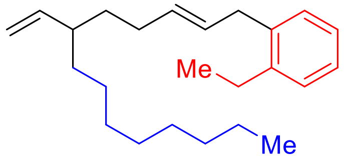

(*E*)-1-(2-Ethylphenyl)-6-vinyltetradec-2-ene (3ak):[S1]

Following the general procedure, reaction was conducted in 1 mmol scale using 1-fluorooctane (130.7 mg, 0.99 mmol) and 2-ethylphenylmagnesium bromide (in THF, 1.03 M, 1.46 mL, 1.5 mmol). Purification by GPC gave the desired product as a colorless oil (288.3 mg, 89%): ^{1}H NMR (400 MHz, $CDCl_3$): δ 7.16–7.14 (m, 4H), 5.56–5.39 (m, 3H), 4.96–4.88 (m, 2H), 3.33 (d, J = 6.6 Hz, 2H), 2.64 (q, J = 7.6 Hz, 2H), 2.08–1.89 (m, 3H), 1.45–1.36 (m, 1H), 1.32–1.19 (m, 15H), 1.20 (t, J = 7.6 Hz, 3H), 0.88 (t, J = 6.8 Hz, 3H); ^{13}C NMR (100 MHz, $CDCl_3$): δ 143.3, 142.0, 138.4, 131.7, 129.3, 128.6, 128.3, 126.2, 125.8, 114.2, 43.6, 35.8, 35.0, 34.7, 31.9, 30.2, 29.7, 29.6, 29.3, 27.1, 25.5, 22.7, 15.1, 14.1; MS (DART) *m/z* (relative intensity, %): 327 ([M+H]$^+$, 100), 251 (92), 195 (35), 151 (24), 133 (87); HRMS (DART) *m/z* [M+H]$^+$ Calcd for $C_{24}H_{39}$ 327.3052; Found 327.3057.

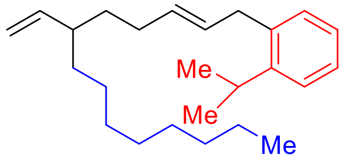

(*E*)-1-(2-Isopropylphenyl)-6-vinyltetradec-2-ene (3a*l*):[S1]

Following the general procedure, reaction was conducted in 1 mmol scale using 1-fluorooctane (137.9 mg, 1.04 mmol) and 2-isopropylphenylmagnesium bromide (in THF, 0.97 M, 1.55 mL, 1.5 mmol). Purification by GPC gave the desired product as a colorless oil (303.2 mg, 85%): ^{1}H NMR (400 MHz, $CDCl_3$): δ 7.26 (d, J = 7.2 Hz, 1H), 7.19 (td, J = 6.4 Hz, 1H), 7.12–7.10 (m, 2H), 5.53–5.44 (m, 3H), 4.96–4.88 (m, 2H), 3.36 (d, J = 5.6 Hz, 2H), 3.18 (sept, J = 6.8 Hz, 1H), 2.05–1.90 (m, 3H), 1.44–1.35 (m, 1H), 1.36–1.17 (m, 15H), 1.21 (d, J = 6.8 Hz, 6H), 0.88 (t, J = 6.8 Hz, 3H); ^{13}C NMR (100 MHz, $CDCl_3$): δ 146.7, 143.2, 137.5, 131.5, 129.5, 129.0, 126.4, 125.6, 125.2, 114.2, 43.6, 35.9, 35.0, 34.7, 31.9, 30.2, 29.8, 29.6, 29.3, 28.6, 27.1, 23.8, 22.7, 14.1; MS (DART) *m/z* (relative intensity, %): 341 ([M+H]$^+$, 32), 327 (17), 251 (34), 195 (18), 163 (17), 147 (45), 102 (100); HRMS (DART) *m/z* [M+H]$^+$ Calcd for $C_{25}H_{41}$ 341.3208; Found 341.3237.

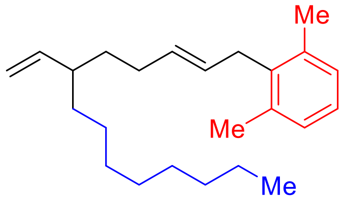

(*E*)-1-(2,6-Dimethylphenyl)-6-vinyltetradec-2-ene (3am):[S1]

Following the general procedure, reaction was conducted in 1 mmol scale using 1-fluorooctane (136.0 mg, 1.03 mmol) and 2,6-dimethylphenylmagnesium bromide (in THF, 0.83 M, 1.81 mL, 1.5 mmol). Purification by GPC gave the desired product as a colorless oil

(302.7 mg, 90%): ^{1}H NMR (400 MHz, $CDCl_3$): δ 7.00 (br s, 3H), 5.51–5.40 (m, 2H), 5.30–5.23 (m, 1H), 4.94–4.86 (m, 2H), 3.31 (d, *J* = 5.6 Hz, 2H), 2.29 (s, 6H), 2.04–1.85 (m, 3H), 1.36–1.17 (m, 16H), 0.88 (t, *J* = 6.8 Hz, 3H); ^{13}C NMR (100 MHz, $CDCl_3$): δ 143.3, 137.1, 136.5, 130.9, 127.9, 126.4, 125.8, 114.1, 43.6, 34.9, 34.7, 32.6, 31.9, 30.1, 29.7, 29.6, 29.3, 27.1, 22.7, 19.9, 14.1; MS (DART) *m/z* (relative intensity, %): 327 ($[M+H]^+$, 45), 215 (17), 207 (12), 195 (15), 133 (37), 102 (100); HRMS (DART) *m/z* $[M+H]^+$ Calcd for $C_{24}H_{39}$ 327.3052; Found 327.3045.

(*E*)-1-(4-Fluoro-2-methylphenyl)-6-vinyltetradec-2-ene (3ao):[S1]

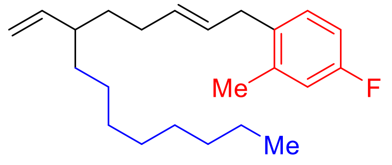

Following the general procedure, reaction was conducted in 1 mmol scale using 1-fluorooctane (125.8 mg, 0.95 mmol) and 4-fluoro-2-methylphenylmagnesium bromide (in THF, 0.48 M, 3.1 mL, 1.5 mmol). Purification by GPC gave the desired product as a colorless oil (242.9 mg, 77%): ^{1}H NMR (400 MHz, $CDCl_3$): δ 7.05 (dd, *J* = 8.0, 6.0 Hz, 1H), 6.85–6.79 (m, 2H), 5.54–5.45 (m, 2H), 5.41–5.34 (m, 1H), 4.96–4.88 (m, 2H), 3.24 (d, *J* = 6.0 Hz, 2H), 2.26 (s, 3H), 2.10–1.89 (m, 3H), 1.44–1.36 (m, 1H), 1.31–1.17 (m, 15H), 0.88 (t, *J* = 6.8 Hz, 3H); ^{13}C NMR (100 MHz, $CDCl_3$): δ 162.4, 160.0, 143.2, 138.4, 138.3, 134.7, 134.6, 131.9, 130.2, 130.1, 127.7, 116.7, 116.5, 114.23, 114.21, 112.5, 112.2, 43.61, 43.59, 35.8, 35.0, 34.7, 31.9, 30.1, 29.7, 29.6, 29.3, 27.1, 22.7, 19.5, 19.4, 14.1; ^{19}F NMR (376 MHz, $CDCl_3$): δ –118.1 (s); MS (DART) *m/z* (relative intensity, %): 331 ($[M+H]^+$, 35), 273 (32), 251 (32), 197 (100), 195 (50), 153 (54); HRMS (DART) *m/z* $[M+H]^+$ Calcd for $C_{23}H_{36}F$ 331.2801; Found 331.2792.

(*E*)-1-(4-Chloro-2-methylphenyl)-6-vinyltetradec-2-ene (3ap):[S1]

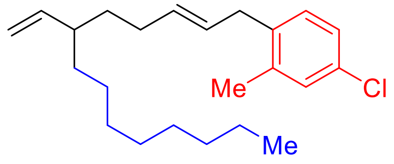

Following the general procedure, reaction was conducted in 1 mmol scale using 1-fluorooctane (137.4 mg, 1.04 mmol) and 4-chloro-2-methylphenylmagnesium bromide (in THF, 0.48 M, 3.1 mL, 1.5 mmol). Purification by GPC gave the desired product as a colorless oil (236.5 mg, 66%): ^{1}H NMR (400 MHz, $CDCl_3$): δ 7.12–7.09 (m, 2H), 7.04 (d, *J* = 8.4 Hz, 1H), 5.53–5.44 (m, 2H), 5.41–5.34 (m, 1H), 4.97–4.88 (m, 2H), 3.24 (d, *J* = 6.0 Hz, 2H), 2.25 (s, 3H), 2.10–1.89 (m, 3H), 1.44–1.36 (m, 1H), 1.31–1.17 (m, 15H), 0.88 (t, *J* = 6.8 Hz, 3H); ^{13}C NMR (100 MHz, $CDCl_3$): δ 143.2, 138.1, 137.5, 132.2, 131.4, 130.2, 129.8, 127.3, 125.9, 114.2, 43.6, 35.9, 35.0, 34.7, 31.9, 30.2, 29.7, 29.6, 29.3, 27.1, 22.7, 19.2, 14.1; MS (DART) *m/z* (relative intensity, %): 347 ($[M+H]^+$, 65), 331 (100), 305 (25), 273 (40), 251 (50), 197 (24); HRMS (DART) *m/z* $[M+H]^+$ Calcd for $C_{23}H_{36}Cl$ 347.2506; Found 347.2495.

(*E*)-1-(2,4-Dimethylphenyl)-6-vinyltetradec-2-ene (3aq):[S1]

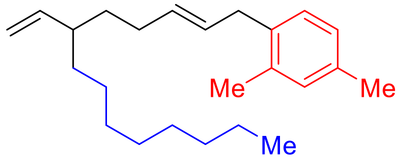

Following the general procedure, reaction was conducted in 1 mmol scale using 1-fluorooctane (129.2 mg, 0.98 mmol) and 2,4-dimethylphenylmagnesium bromide (in THF, 0.54 M, 2.8 mL, 1.5

mmol). Purification by GPC gave the desired product as a colorless oil (304.8 mg, 96%): ^{1}H NMR (400 MHz, $CDCl_3$): δ 7.01 (d, J = 7.6 Hz, 1H), 6.95 (s, 1H), 6.94 (d, J = 7.6 Hz, 1H), 5.53–5.46 (m, 2H), 5.44–5.35 (m, 1H), 4.96–4.88 (m, 2H), 3.25 (d, J = 6.4 Hz, 2H), 2.28 (s, 3H), 2.24 (s, 3H), 2.10–1.85 (m, 3H), 1.43–1.36 (m, 1H), 1.31–1.17 (m, 15H), 0.88 (t, J = 6.8 Hz, 3H): ^{13}C NMR (100 MHz, $CDCl_3$): δ 143.3, 136.0, 135.4, 131.5, 130.9, 128.9, 128.1, 126.6, 114.2, 43.6, 36.2, 35.0, 34.7, 31.9, 30.2, 29.8, 29.6, 29.4, 27.1, 22.7, 20.9, 19.3, 14.1; MS (EI) *m/z* (relative intensity, %): 326 ($[M]^+$, 6), 172 (9), 157 (22), 143 (18), 132 (69), 119 (100); HRMS (EI) *m/z* $[M]^+$ Calcd for $C_{24}H_{38}$ 326.2974; Found 326.2970.

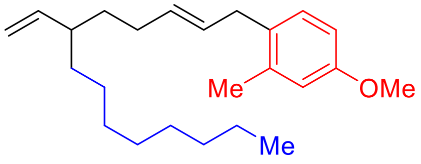

(*E*)-1-(4-Methoxy-2-methylphenyl)-6-vinyltetradec-2-ene (3ar):[S1]
Following the general procedure, reaction was conducted in 1 mmol scale using 1-fluorooctane (131.7 mg, 1.00 mmol) and 4-methoxy-2-methylphenylmagnesium bromide (in THF, 0.51 M, 3.0 mL, 1.5 mmol). Purification by GPC gave the desired product as a colorless oil (227.1 mg, 67%): ^{1}H NMR (400 MHz, $CDCl_3$): δ 7.03 (d, J = 8.0 Hz, 1H), 6.70–6.67 (m, 2H), 5.54–5.45 (m, 2H), 5.41–5.34 (m, 1H), 4.96–4.88 (m, 2H), 3.77 (s, 3H), 3.23 (d, J = 6.0 Hz, 2H), 2.26 (s, 3H), 2.10–1.89 (m, 3H), 1.44–1.36 (m, 1H), 1.31–1.16 (m, 15H), 0.88 (t, J = 6.8 Hz, 3H); ^{13}C NMR (100 MHz, $CDCl_3$): δ 157.8, 143.3, 137.5, 131.4, 131.3, 129.8, 128.3, 115.8, 114.2, 110.8, 55.2, 43.6, 35.8, 34.9, 34.7, 31.9, 30.1, 29.7, 29.6, 29.3, 27.1, 22.7, 19.6, 14.1; MS (DART) *m/z* (relative intensity, %): 343 ($[M+H]^+$, 95), 331 (30), 301 (10), 251 (12), 239 (14), 195 (16), 135 (100); HRMS (DART) *m/z* $[M+H]^+$ Calcd for $C_{24}H_{39}O$ 343.3001; Found 343.2988.

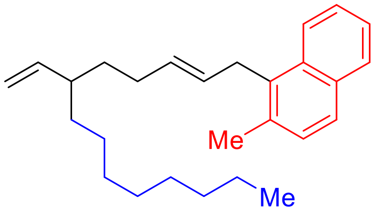

(*E*)-1-(2-Methyl-1-naphthyl)-6-vinyltetradec-2-ene (3as):[S1]
Following the general procedure, reaction was conducted in 1 mmol scale using 1-fluorooctane (128.2 mg, 0.97 mmol) and 2-methyl-1-naphthylmagnesium bromide (in THF, 0.15 M, 10.0 mL, 1.5 mmol) for 20 h. Purification by GPC gave the desired product as a pale orange oil (284.5 mg, 81%): ^{1}H NMR (400 MHz, $CDCl_3$): δ 8.01 (d, J = 8.4 Hz, 1H), 7.79 (d, J = 7.2 Hz, 1H), 7.64 (d, J = 8.0 Hz, 1H), 7.46 (td, J = 7.2, 2.0 Hz, 1H), 7.39 (td, J = 8.0, 1.2 Hz, 1H), 7.30 (d, J = 8.4 Hz, 1H), 5.57 (dt, J = 14.8, 5.6 Hz, 1H), 5.48–5.39 (m, 1H), 5.34 (dt, J = 15.6, 6.4 Hz, 1H), 4.91–4.81 (m, 2H), 3.76 (d, J = 4.4 Hz, 2H), 2.47 (s, 3H), 2.02–1.81 (m, 3H), 1.38–1.12 (m, 16H), 0.87 (t, J = 6.8 Hz, 3H); ^{13}C NMR (100 MHz, $CDCl_3$): δ 143.2, 133.5, 133.4, 132.5, 132.3, 131.4, 129.2, 128.4, 127.2, 126.2, 125.8, 124.4, 123.9, 114.1, 43.5, 34.9, 34.6, 31.9, 31.6, 30.1, 29.7, 29.6, 29.3, 27.1, 22.7, 20.1, 14.1; MS (DART) *m/z* (relative intensity, %): 363 ($[M+H]^+$, 100), 331 (65), 239 (14), 213 (15), 195 (48), 168 (24), 155 (56), 135 (15); HRMS (DART) *m/z* $[M+H]^+$ Calcd for $C_{27}H_{39}$ 363.3052; Found 363.3040.

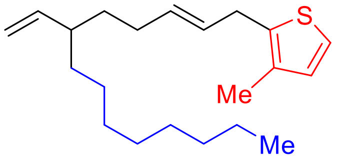

(*E*)-1-(3-Methyl-2-thienyl)-6-vinyltetradec-2-ene (3at):[S1]
Following the general procedure, reaction was conducted in 1 mmol scale

using 1-fluorooctane (126.0 mg, 0.95 mmol) and 3-methyl-2-thienylmagnesium bromide (in THF, 0.53 M, 2.8 mL, 1.5 mmol). Purification by GPC gave the desired product as a pale yellow oil (80.4 mg, 27%): ^{1}H NMR (400 MHz, $CDCl_3$): δ 7.02 (d, *J* = 4.8 Hz, 1H), 6.79 (d, *J* = 4.8 Hz, 1H), 5.55–5.45 (m, 3H), 4.97–4.90 (m, 2H), 3.39 (d, *J* = 5.2 Hz, 2H), 2.14 (s, 3H), 2.11–1.80 (m, 3H), 1.57–1.38 (m, 1H), 1.33–1.18 (m, 15H), 0.88 (t, *J* = 6.8 Hz, 3H); ^{13}C NMR (100 MHz, $CDCl_3$): δ 143.2, 136.9, 132.6, 132.1, 130.0, 127.6, 121.3, 114.3, 43.5, 35.0, 34.6, 31.9, 31.1, 30.0, 29.7, 29.6, 29.3, 27.1, 22.7, 14.1, 13.5; MS (DART) *m/z* (relative intensity, %): 319 ($[M+H]^+$, 25), 283 (57), 239 (85), 195 (100), 177 (10), 151 (27), 133 (31), 121 (13); HRMS (DART) *m/z* $[M+H]^+$ Calcd for $C_{21}H_{35}S$ 319.2460; Found 319.2460.

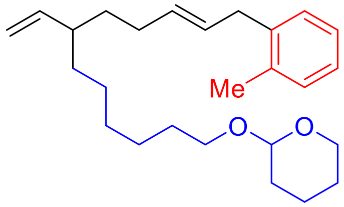

(*E*)-2-[12-(2-Methylphenyl)-7-vinyl-10-dodecen-1-yloxy]tetrahydropyran (3bj):[S1]

Following the general procedure, reaction was conducted using 2-(6-fluorohexan-1-yloxy)tetrahydropyran (105.9 mg, 0.52 mmol) and 2-methylphenylmagnesium bromide (in THF, 0.82 M, 0.91 mL, 0.75 mmol). Purification by GPC gave the desired product as a colorless oil (133.3 mg, 67%): ^{1}H NMR (400 MHz, $CDCl_3$): δ 7.12 (br s, 4H), 5.50–5.35 (m, 3H), 4.96–4.87 (m, 2H), 4.58–4.56 (m, 1H), 3.90–3.84 (m, 1H), 3.72 (dt, *J* = 9.6, 6.8 Hz, 1H), 3.53–3.47 (m, 1H), 3.37 (dt, *J* = 9.6, 6.8 Hz, 1H), 3.29 (d, *J* = 5.6 Hz, 2H), 2.28 (s, 3H), 2.09–1.67 (m, 5H), 1.60–1.51 (m, 6H), 1.36–1.17 (m, 10H); ^{13}C NMR (100 MHz, $CDCl_3$): δ 143.2, 139.1, 136.2, 131.7, 130.0, 128.9, 127.8, 126.0, 125.9, 114.2, 98.8, 67.6, 62.3, 43.6, 36.5, 34.9, 34.7, 30.7, 30.1, 29.7, 29.5, 27.0, 26.2, 25.5, 19.7, 19.3; MS (DART) *m/z* (relative intensity, %): 385 ($[M+H]^+$, 100), 315 (22), 299 (14), 283 (13), 195 (22), 147 (32), 102 (52); HRMS (DART) *m/z* $[M+H]^+$ Calcd for $C_{26}H_{41}O_2$ 385.3107; Found 385.3099.

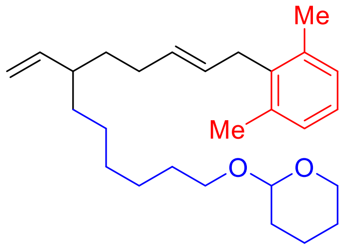

(*E*)-2-[12-(2,6-Dimethylphenyl)-7-vinyl-10-dodecen-1-yloxy]tetrahydropyran (3bm):[S1]

Following the general procedure, reaction was conducted using 2-(6-fluorohexan-1-yloxy)tetrahydropyran (106.3 mg, 0.52 mmol) and 2,6-dimethylphenylmagnesium bromide (in THF, 0.83 M, 0.90 mL, 0.75 mmol). Purification by GPC gave the desired product as a colorless oil (179.8 mg, 87%): ^{1}H NMR (400 MHz, $CDCl_3$): δ 7.01–7.00 (m, 3H), 5.51–5.40 (m, 2H), 5.30–5.21 (m, 1H), 4.94–4.85 (m, 2H), 4.58–4.56 (m, 1H), 3.91–3.84 (m, 1H), 3.72 (dt, *J* = 9.6, 6.8 Hz, 1H), 3.53–3.47 (m, 1H), 3.37 (dt, *J* = 9.6, 6.8 Hz, 1H), 3.31 (d, *J* = 5.2 Hz, 2H), 2.29 (s, 6H), 2.02–1.67 (m, 5H), 1.60–1.48 (m, 6H), 1.40–1.15 (m, 10H); ^{13}C NMR (100 MHz, $CDCl_3$): δ 143.2, 137.1, 136.5, 130.8, 127.9, 126.4, 125.8, 114.2, 98.8, 67.7, 62.3, 43.5, 34.8, 34.7, 32.6, 30.8, 30.1, 29.7, 29.5, 27.0, 26.2, 25.5, 19.8, 19.7; MS (DART) *m/z* (relative intensity, %): 399 ($[M+H]^+$, 35), 119 (10), 102 (100); HRMS (DART) *m/z* $[M+H]^+$ Calcd for $C_{27}H_{43}O_2$ 399.3263; Found 399.3262.

(*E*)-12-*tert*-Butyldimethylsiloxy-1-(2-methylphenyl)-6-vinyldodec-2-ene (3cj):[S1]

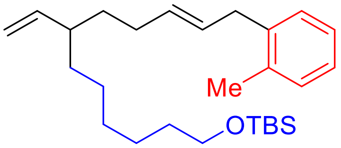

Following the general procedure, reaction was conducted using 1-*tert*-butyldimethylsiloxy-6-fluorohexane (116.4 mg, 0.50 mmol) and 2-methylphenylmagnesium bromide (in THF, 0.82 M, 0.91 mL, 0.75 mmol). Purification by GPC gave the desired product as a colorless oil (123.8 mg, 60%): ^{1}H NMR (400 MHz, $CDCl_3$): δ 7.13 (br s, 4H), 5.55–5.36 (m, 3H), 4.96–4.88 (m, 2H), 3.59 (t, J = 6.8 Hz, 2H), 3.30 (d, J = 6.0 Hz, 2H), 2.28 (s, 3H), 2.04–1.89 (m, 3H), 1.55–1.17 (m, 12H), 0.89 (s, 9H), 0.04 (s, 6H); ^{13}C NMR (100 MHz, $CDCl_3$): δ 143.2, 139.1, 136.2, 131.8, 130.0, 128.9, 127.8, 126.0, 125.9, 114.3, 63.3, 43.6, 36.6, 34.9, 34.7, 32.9, 30.2, 29.5, 27.1, 26.0, 25.8, 19.4, 18.4, –5.3; MS (DART) *m/z* (relative intensity, %): 415 ([M+H]$^+$, 100), 283 (58), 195 (25), 131 (10), 102 (13); HRMS (DART) *m/z* [M+H]$^+$ Calcd for $C_{27}H_{47}OSi$ 415.3396; Found 415.3381.

(*E*)-12-*tert*-Butyldimethylsiloxy-1-(2-ethylphenyl)-6-vinyldodec-2-ene (3ck):[S1]

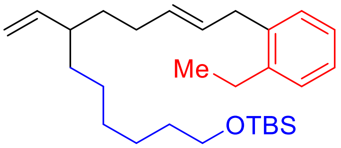

Following the general procedure, reaction was conducted using 1-*tert*-butyldimethylsiloxy-6-fluorohexane (112.1 mg, 0.48 mmol) and 2-ethylphenylmagnesium bromide (in THF, 1.03 M, 0.73 mL, 0.75 mmol). Purification by GPC gave the desired product as a colorless oil (192.7 mg, 94%): ^{1}H NMR (400 MHz, $CDCl_3$): δ 7.13–7.09 (m, 4H), 5.49–5.38 (m, 3H), 4.92–4.84 (m, 2H), 3.54 (t, J = 6.4 Hz, 2H), 3.29 (d, J = 6.0 Hz, 2H), 2.60 (q, J = 7.6 Hz, 2H), 2.04–1.83 (m, 3H), 1.55–1.17 (m, 12H), 1.16 (t, J = 7.6 Hz, 3H), 0.85 (s, 9H), 0.04 (s, 6H); ^{13}C NMR (100 MHz, $CDCl_3$): δ 143.2, 142.0, 138.4, 131.7, 129.3, 128.6, 128.3, 126.2, 125.8, 114.3, 63.3, 43.6, 35.8, 34.9, 34.7, 32.8, 30.2, 29.5, 27.1, 26.0, 25.7, 25.5, 18.4, 15.1, –5.3; MS (DART) *m/z* (relative intensity, %): 429 ([M+H]$^+$, 25), 371 (15), 327 (44), 283 (42), 239 (95), 195 (100); HRMS (DART) *m/z* [M+H]$^+$ Calcd for $C_{28}H_{49}OSi$ 429.3553; Found 429.3581.

(*E*)-12-*tert*-Butyldimethylsiloxy-1-(2,6-dimethylphenyl)-6-vinyldodec-2-ene (3cm):[S1]

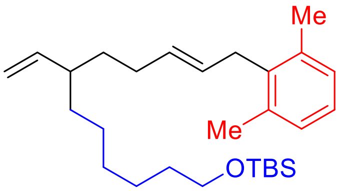

Following the general procedure, reaction was conducted using 1-*tert*-butyldimethylsiloxy-6-fluorohexane (114.4 mg, 0.49 mmol) and 2,6-dimethylphenylmagnesium bromide (in THF, 0.83 M, 0.90 mL, 0.75 mmol). Purification by GPC gave the desired product as a colorless oil (175.5 mg, 84%): ^{1}H NMR (400 MHz, $CDCl_3$): δ 7.00 (br s, 3H), 5.54–5.40 (m, 2H), 5.33–5.25 (m, 1H), 4.94–4.85 (m, 2H), 3.58 (t, J = 6.8 Hz, 2H), 3.32 (d, J = 5.6 Hz, 2H), 2.29 (s, 6H), 2.03–1.84 (m, 3H), 1.49 (quin, J = 6.8 Hz, 2H), 1.40–1.18 (m, 10H), 0.89 (s, 9H), 0.04 (s, 6H); ^{13}C NMR (100 MHz, $CDCl_3$): δ 143.2, 137.1, 136.5, 130.9, 127.9, 126.4, 125.8, 114.2, 63.3, 43.5, 34.8, 34.7, 32.8, 32.6, 30.1, 29.5, 27.1, 26.0, 25.7, 19.9, 18.4, –5.3; MS (DART) *m/z* (relative

intensity, %): 429 ([M+H]$^+$, 100), 347 (13), 327 (12), 297 (24), 283 (19), 239 (42), 195 (63), 133 (18), 119 (52); HRMS (DART) *m/z* [M+H]$^+$ Calcd for $C_{28}H_{49}OSi$ 429.3553; Found 429.3540.

(*E*)-17-(2,6-Dimethylphenyl)-12-vinylheptadec-1,15-diene (3jm):[S1]

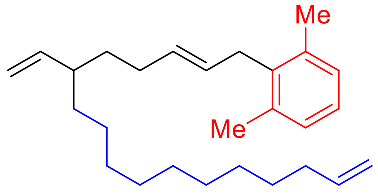

Following the general procedure, reaction was conducted using 11-fluoro-1-undecene (85.7 mg, 0.50 mmol) and 2,6-dimethylphenylmagnesium bromide (in THF, 0.83 M, 0.9 mL, 0.75 mmol). Purification by GPC gave the desired product as a colorless oil (166.3 mg, 91%): ^{1}H NMR (400 MHz, $CDCl_3$): δ 7.00 (br s, 3H), 5.86–5.76 (m, 1H), 5.51–5.40 (m, 2H), 5.30–5.23 (m, 1H), 5.01–4.85 (m, 4H), 3.31 (d, *J* = 5.2 Hz, 2H), 2.29 (s, 6H), 2.03 (q, *J* = 6.8 Hz, 2H), 2.04–1.85 (m, 3H), 1.40–1.15 (m, 18H); ^{13}C NMR (100 MHz, $CDCl_3$): δ 143.2, 139.2, 137.1, 136.5, 130.9, 127.9, 126.4, 125.8, 114.15, 114.08, 43.6, 34.9, 34.7, 33.8, 32.6, 30.1, 29.7, 29.6, 29.5, 29.1, 28.9, 27.1, 19.9; MS (DART) *m/z* (relative intensity, %): 367 ([M+H]$^+$, 58), 283 (25), 239 (100), 195 (77); HRMS (DART) *m/z* [M+H]$^+$ Calcd for $C_{27}H_{43}$ 367.3365; Found 367.3366.

(*E*)-2-(9-(2,6-Dimethylphenyl)-4-vinyl-7-nonen-1-yl)thiophene (3dm):[S1]

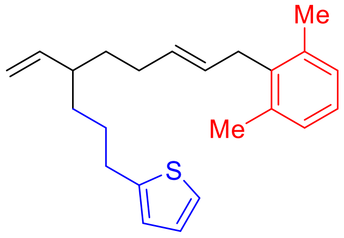

Following the general procedure, reaction was conducted using 2-(3-fluoropropyl)thiophene (84.6 mg, 0.59 mmol) and 2,6-dimethylphenylmagnesium bromide (in THF, 0.83 M, 1.0 mL, 0.83 mmol). Purification by GPC gave the desired product as a pale yellow oil (185.2 mg, 93%): ^{1}H NMR (400 MHz, $CDCl_3$): δ 7.08 (dd, *J* = 4.8, 1.2 Hz, 1H), 7.00 (br s, 3H), 6.89 (dd, *J* = 4.8, 3.2 Hz, 1H), 6.74 (dd, *J* = 3.2, 1.2 Hz, 1H), 5.51–5.40 (m, 2H), 5.29–5.22 (m, 1H), 4.96–4.87 (m, 2H), 3.31 (d, *J* = 5.6 Hz, 2H), 2.77 (q, *J* = 6.4 Hz, 2H), 2.28 (s, 6H), 2.02-1.83 (m, 3H), 1.72–1.52 (m, 2H), 1.42-1.19 (m, 4H); ^{13}C NMR (100 MHz, $CDCl_3$): δ 145.6, 142.7, 137.0, 136.4, 130.7, 127.9, 126.6, 125.8, 123.9, 122.7, 114.6, 43.4, 34.7, 34.2, 32.6, 30.1, 29.9, 29.3, 19.9; MS (DART) *m/z* (relative intensity, %): 339 ([M+H]$^+$, 100), 285 (12), 251 (15), 233 (18), 133 (14), 119 (58); HRMS (DART) *m/z* [M+H]$^+$ Calcd for $C_{23}H_{31}S$ 339.2147; Found 339.2133.

(*E*)-4-(8-(2,6-Dimethylphenyl)-3-vinyl-6-octen-1-yl)-1-tosylpiperidine (3gm):[S1]

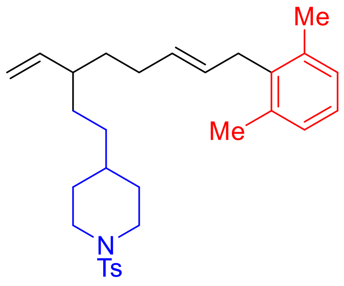

Following the general procedure, reaction was conducted using 4-(2-fluoroethyl)-1-tosylpiperidine (142.3 mg, 0.50 mmol) and 2,6-dimethylphenylmagnesium bromide (in THF, 0.83 M, 0.90 mL, 0.75 mmol). Purification by GPC gave the desired product as a colorless oil (225.4 mg, 94%): ^{1}H NMR (400 MHz, $CDCl_3$): δ 7.63 (d, *J* = 8.4 Hz, 2H), 7.31 (d, *J* = 8.4 Hz, 2H), 6.99 (br s, 3H), 5.47–5.35 (m, 2H), 5.28–5.20 (m, 1H), 4.94–4.82 (m, 2H), 3.74 (d, *J* = 11.6 Hz, 2H), 3.29 (d,

J = 6.0 Hz, 2H), 2.42 (s, 3H), 2.27 (s, 6H), 2.17 (tt, J = 12.0, 1.6 Hz, 2H), 2.01-1.78 (m, 3H), 1.66 (d, J = 12.8 Hz, 2H), 1.37-1.01 (m, 9H); ^{13}C NMR (100 MHz, $CDCl_3$): δ 143.3, 142.7, 137.0, 136.4, 133.1, 130.6, 129.5, 127.9, 127.7, 126.6, 125.8, 114.5, 46.5, 43.5, 35.1, 34.6, 33.4, 32.5, 31.7, 31.6, 31.3, 30.0, 21.5, 19.8; MS (DART) *m/z* (relative intensity, %): 480 ([M+H]$^+$, 100), 415 (17), 339 (90), 324 (38), 283 (22), 245 (15), 119 (39); HRMS (DART) *m/z* [M+H]$^+$ Calcd for $C_{30}H_{42}NO_2S$ 480.2936; Found 480.2935.

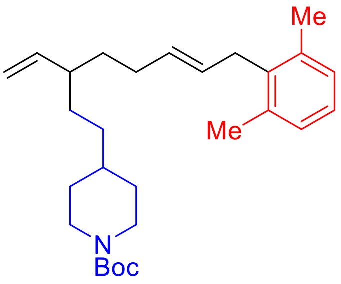

(*E*)-1-*tert*-Butoxycarbonyl-4-(8-(2,6-dimethylphenyl)-3-vinyl-6-octen-1-yl)piperidine (3km):[S1]
Following the general procedure, reaction was conducted using 1-*tert*-butoxycarbonyl-4-(2-fluoroethyl)piperidine (115.0 mg, 0.50 mmol) and 2,6-dimethylphenylmagnesium bromide (in THF, 0.83 M, 0.90 mL, 0.75 mmol). Purification by GPC gave the desired product as a colorless oil (187.6 mg, 89%): ^{1}H NMR (400 MHz, $CDCl_3$): δ 7.01 (br s, 3H), 5.51–5.40 (m, 2H), 5.30–5.22 (m, 1H), 4.96–4.86 (m, 2H), 4.04 (br, 2H), 3.32 (dd, J = 6.0, 1.2 Hz, 2H), 2.64 (t, J = 12.0 Hz, 2H), 2.29 (s, 6H), 2.03–1.84 (m, 3H), 1.62–1.57 (m, 2H), 1.45 (s, 9H), 1.37-1.01 (m, 9H); ^{13}C NMR (100 MHz, $CDCl_3$): δ 154.9, 143.0, 137.1, 136.5, 130.7, 127.9, 126.6, 125.8, 114.5, 79.1, 43.7, 36.1, 34.7, 33.9, 32.6, 31.8, 30.1, 28.5, 19.9; MS (ESI) *m/z* (relative intensity, %): 448 ([M+Na]$^+$, 100), 392 (30), 326 (12); HRMS (ESI) *m/z* [M+Na]$^+$ Calcd for $C_{28}H_{43}NO_2Na$ 448.3191; Found 448.3191.

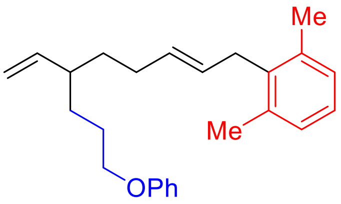

(*E*)-1-(2,6-Dimethylphenyl)-9-phenoxy-6-vinylnon-2-ene (3*l*m):[S1]
Following the general procedure, reaction was conducted using 3-fluoropropyl phenyl ether (83.3 mg, 0.54 mmol) and 2,6-dimethylphenylmagnesium bromide (in THF, 0.83 M, 1.0 mL, 0.83 mmol). Purification by GPC gave the desired product as a colorless oil (174.7 mg, 93%): ^{1}H NMR (400 MHz, $CDCl_3$): δ 7.25 (t, J = 8.0 Hz, 2H), 7.00 (br s, 3H), 6.91 (t, J = 6.8 Hz, 1H), 6.86 (dd, J = 8.8, 0.8 Hz, 2H), 5.53–5.41 (m, 2H), 5.29–5.24 (m, 1H), 4.98–4.90 (m, 2H), 3.88 (t, J = 6.4 Hz, 2H), 3.30 (d, J = 5.6 Hz, 2H), 2.28 (s, 6H), 2.05–1.22 (m, 9H); ^{13}C NMR (100 MHz, $CDCl_3$): δ 159.0, 142.5, 137.0, 136.4, 130.6, 129.3, 127.9, 126.6, 125.8, 120.4, 114.9, 114.4, 67.7, 43.4, 34.7, 32.5, 31.1, 30.1, 26.9, 19.8; MS (DART) *m/z* (relative intensity, %): 349 ([M+H]$^+$, 13), 255 (30), 239 (12), 195 (20), 119 (18), 102 (100); HRMS (DART) *m/z* [M+H]$^+$ Calcd for $C_{25}H_{33}O$ 349.2531; Found 349.2558.

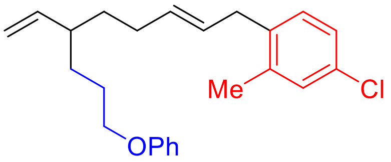

(*E*)-1-(4-Chloro-2-methylphenyl)-9-phenoxy-6-vinylnon-2-ene (3*l*h):[S1]
Following the general procedure, reaction was conducted using 3-fluoropropyl phenyl ether (72.7 mg, 0.47 mmol) and 4-chloro-2-methylphenylmagnesium bromide (in

THF, 0.48 M, 1.5 mL, 0.72 mmol). Purification by GPC gave the desired product as a colorless oil (116.8 mg, 67%): ^{1}H NMR (400 MHz, $CDCl_3$): δ 7.27 (dd, *J* = 8.8, 7.2 Hz, 2H), 7.11–7.08 (m, 2H), 7.03 (d, *J* = 7.2 Hz, 1H), 6.92 (t, *J* = 7.2 Hz, 1H), 6.88 (dd, *J* = 8.8, 0.8 Hz, 2H), 5.56–5.45 (m, 2H), 5.41–5.34 (m, 1H), 5.02–4.93 (m, 2H), 3.92 (t, *J* = 6.4 Hz, 2H), 3.24 (d, *J* = 6.0 Hz, 2H), 2.24 (s, 3H), 2.12–1.25 (m, 9H); ^{13}C NMR (100 MHz, $CDCl_3$): δ 159.0, 142.5, 138.1, 137.5, 132.0, 131.4, 130.2, 129.8, 129.4, 127.5, 125.9, 120.5, 115.0, 114.4, 67.8, 43.4, 35.9, 34.7, 31.2, 30.1, 27.0, 19.2; MS (DART) *m/z* (relative intensity, %): 369 ($[M+H]^+$, 5), 342 (16), 239 (25), 195 (24); HRMS (DART) *m/z* $[M+H]^+$ Calcd for $C_{24}H_{30}ClO$ 369.1985; Found 369.2008.

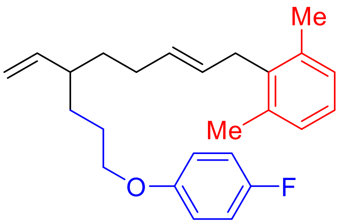

(*E*)-1-(2,6-Dimethylphenyl)-9-(4-fluorophenoxy)-6-vinylnon-2-ene (3em):[S1]

Following the general procedure, reaction was conducted using 4-fluorophenyl 3-fluoropropyl ether (90.0 mg, 0.52 mmol) and 2,6-dimethylphenylmagnesium bromide (in THF, 0.83 M, 0.90 mL, 0.75 mmol). Purification by GPC gave the desired product as a colorless oil (179.6 mg, 94%): ^{1}H NMR (400 MHz, $CDCl_3$): δ 7.00 (br s, 3H), 6.94 (t, *J* = 8.8 Hz, 2H), 6.79 (dd, *J* = 8.8, 4.0 Hz, 2H), 5.54–5.40 (m, 2H), 5.30–5.21 (m, 1H), 4.99–4.90 (m, 2H), 3.86 (t, *J* = 6.8 Hz, 2H), 3.31 (d, *J* = 5.2 Hz, 2H), 2.28 (s, 6H), 2.05–1.22 (m, 9H); ^{13}C NMR (100 MHz, $CDCl_3$): δ 158.3, 155.9, 155.2, 142.5, 137.0, 136.5, 130.6, 127.9, 126.7, 125.8, 115.8, 115.6, 115.4, 115.3, 114.9, 68.6, 43.4, 34.8, 32.6, 31.1, 30.1, 26.9, 19.8; ^{19}F NMR (376 MHz, $CDCl_3$): δ –124.3 (s); MS (DART) *m/z* (relative intensity, %): 367 ($[M+H]^+$, 4), 283 (19), 251 (32), 239 (100), 195 (65); HRMS (DART) *m/z* $[M+H]^+$ Calcd for $C_{25}H_{32}FO$ 367.2437; Found 367.2466.

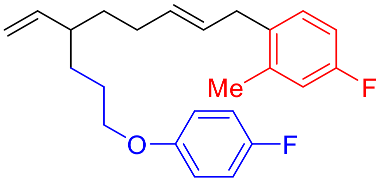

(*E*)-1-(4-Fluoro-2-methylphenyl)-9-(4-fluorophenoxy)-6-vinylnon-2-ene (3eo):[S1]

Following the general procedure, reaction was conducted using 4-fluorophenyl 3-fluoropropyl ether (82.4 mg, 0.48 mmol) and 4-fluoro-2-methylphenylmagnesium bromide (in THF, 0.48 M, 1.56 mL, 0.75 mmol). Purification by GPC gave the desired product as a colorless oil (124.7 mg, 70%): ^{1}H NMR (400 MHz, $CDCl_3$): δ 7.05 (dd, *J* = 8.0, 6.0 Hz, 1H), 6.94 (t, *J* = 8.8 Hz, 2H), 6.84–6.78 (m, 4H), 5.55–5.46 (m, 2H), 5.40–5.35 (m, 1H), 5.02–4.93 (m, 2H), 3.87 (t, *J* = 6.8 Hz, 2H), 3.24 (d, *J* = 6.4 Hz, 2H), 2.25 (s, 3H), 2.10–1.90 (m, 3H), 1.84–1.27 (m, 6H); ^{13}C NMR (100 MHz, $CDCl_3$): δ 162.4, 160.0, 158.2, 155.9, 155.2, 155.1, 142.5, 138.35, 138.27, 134.6, 134.5, 131.6, 130.15, 130.07, 127.9, 116.7, 116.5, 115.8, 115.6, 115.33, 115.25, 115.0, 112.4, 112.2, 68.5, 43.4, 35.7, 34.7, 31.1, 30.1, 27.0, 19.4; ^{19}F NMR (376 MHz, $CDCl_3$): δ –118.0 (s, 1F), –124.3 (s, 1F); MS (DART) *m/z* (relative intensity, %): 371 ($[M+H]^+$, 15), 283 (15), 259 (54), 239 (100), 195 (73), 102 (94); HRMS (DART) *m/z* $[M+H]^+$ Calcd for $C_{24}H_{29}F_2O$ 371.2187; Found

371.2205.

(*E*)-9-(4-Chlorophenoxy)-1-(2,6-dimethylphenyl)-6-vinylnon-2-ene (3fm):[S1]

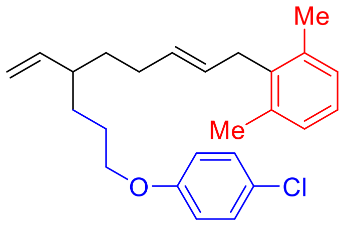

Following the general procedure, reaction was conducted using 4-chlorophenyl 3-fluoropropyl ether (95.3 mg, 0.51 mmol) and 2,6-dimethylphenylmagnesium bromide (in THF, 0.83 M, 0.90 mL, 0.75 mmol). Purification by GPC gave the desired product as a colorless oil (188.1 mg, 97%): ^{1}H NMR (400 MHz, $CDCl_3$): δ 7.21 (d, *J* = 8.8 Hz, 2H), 7.00 (br s, 3H), 6.79 (d, *J* = 8.8 Hz, 2H), 5.53–5.41 (m, 2H), 5.30–5.23 (m, 1H), 4.99–4.90 (m, 2H), 3.86 (t, *J* = 6.4 Hz, 2H), 3.31 (d, *J* = 5.2 Hz, 2H), 2.28 (s, 6H), 2.05–1.22 (m, 9H); ^{13}C NMR (100 MHz, $CDCl_3$): δ 157.7, 142.5, 137.0, 136.5, 130.6, 129.2, 127.9, 126.7, 125.8, 125.3, 115.3, 114.9, 68.2, 43.4, 34.8, 32.6, 31.1, 30.1, 26.9, 19.9; MS (DART) *m/z* (relative intensity, %): 383 ($[M+H]^+$, 2), 342 (16), 283 (32), 251 (35), 239 (98), 195 (100); HRMS (DART) *m/z* $[M+H]^+$ Calcd for $C_{25}H_{32}ClO$ 383.2142; Found 383.2168.

(*E*)-9-(4-Chlorophenoxy)-1-(2-methyl-1-naphthyl)-6-vinylnon-2-ene (3fs):[S1]

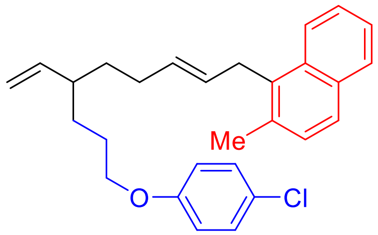

Following the general procedure, reaction was conducted using 4-chlorophenyl 3-fluoropropyl ether (100.2 mg, 0.53 mmol) and 2-methyl-1-naphthylmagnesium bromide (in THF, 0.15 M, 5.0 mL, 0.75 mmol) for 20 h. Purification by GPC gave the desired product as a colorless oil (158.7 mg, 71%): ^{1}H NMR (400 MHz, $CDCl_3$): δ 8.00 (d, *J* = 8.8 Hz, 1H), 7.79 (d, *J* = 7.2 Hz, 1H), 7.64 (d, *J* = 8.0 Hz, 1H), 7.46 (td, *J* = 8.4, 1.6 Hz, 1H), 7.39 (t, *J* = 8.0 Hz, 1H), 7.30 (d, *J* = 8.8 Hz, 1H), 7.20 (d, *J* = 8.4 Hz, 2H), 6.77 (d, *J* = 8.4 Hz, 2H), 5.59 (dt, *J* = 14.8, 5.6 Hz, 1H), 5.49–5.40 (m, 1H), 5.33 (dt, *J* = 15.6, 6.4 Hz, 1H), 4.96–4.85 (m, 2H), 3.83 (t, *J* = 6.4 Hz, 2H), 3.76 (d, *J* = 4.8 Hz, 2H), 2.47 (s, 3H), 2.05–1.20 (m, 9H); ^{13}C NMR (100 MHz, $CDCl_3$): δ 157.6, 142.5, 133.44, 133.42, 132.5, 132.3, 131.1, 129.21, 129.17, 128.4, 127.4, 126.2, 125.8, 125.2, 124.4, 123.9, 115.7, 114.9, 68.2, 43.3, 34.6, 31.6, 31.0, 30.1, 26.8, 20.1; MS (DART) *m/z* (relative intensity, %): 419 ($[M+H]^+$, 40), 386 (15), 342 (16), 283 (24), 239 (100), 195 (95), 177 (24); HRMS (DART) *m/z* $[M+H]^+$ Calcd for $C_{28}H_{32}ClO$ 419.2142; Found 419.2156.

(*E*)-12-Chloro-1-(2,6-dimethylphenyl)-6-vinyldodec-2-ene (3mm):[S1]

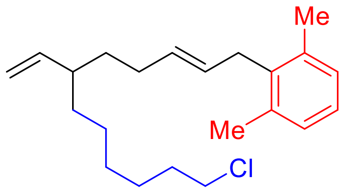

Following the general procedure, reaction was conducted using 1-chloro-6-fluorohexane (69.6 mg, 0.50 mmol) and 2,6-dimethylphenylmagnesium bromide (in THF, 0.83 M, 0.9 mL, 0.75 mmol). Purification by GPC gave the desired product as a pale yellow oil (135.4 mg, 81%): ^{1}H NMR (400 MHz, $CDCl_3$): δ 7.02 (br s, 3H), 5.50–5.42 (m, 2H), 5.30–5.23 (m, 1H), 4.95–4.86 (m, 2H), 3.52 (t,

J = 7.2 Hz, 2H), 3.31 (d, J = 5.6 Hz, 2H), 2.29 (s, 6H), 2.03–1.85 (m, 3H), 1.74 (quin, J = 7.6 Hz, 2H), 1.44–1.17 (m, 10H); ^{13}C NMR (100 MHz, $CDCl_3$): δ 143.1, 137.1, 136.5, 130.8, 127.9, 126.5, 125.8, 114.3, 45.2, 43.5, 34.7, 32.6, 30.1, 28.9, 26.9, 26.8, 19.9; MS (DART) *m/z* (relative intensity, %): 333 ($[M+H]^+$, 12), 283 (14), 239 (80), 195 (100); HRMS (DART) *m/z* $[M+H]^+$ Calcd for $C_{22}H_{34}Cl$ 333.2349; Found 333.2344.

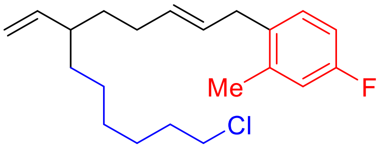

(*E*)-12-Chloro-1-(4-fluoro-2-methylphenyl)-6-vinyldodec-2-ene (3mo):[S1]

Following the general procedure, reaction was conducted using 1-chloro-6-fluorohexane (76.0 mg, 0.55 mmol) and 4-fluoro-2-methylphenylmagnesium bromide (in THF, 0.48 M, 1.56 mL, 0.75 mmol). Purification by GPC gave the desired product as a pale yellow oil (129.7 mg, 70%): ^{1}H NMR (400 MHz, $CDCl_3$): δ 7.05 (dd, J = 8.0, 6.0 Hz, 1H), 6.86–6.78 (m, 2H), 5.53–5.43 (m, 2H), 5.40–5.33 (m, 1H), 4.97–4.88 (m, 2H), 3.52 (t, J = 6.8 Hz, 2H), 3.24 (d, J = 6.0 Hz, 2H), 2.26 (s, 3H), 2.10–1.89 (m, 3H), 1.74 (quin, J = 6.8 Hz, 2H), 1.44–1.17 (m, 10H); ^{13}C NMR (100 MHz, $CDCl_3$): δ 162.4, 160.0, 143.0, 138.35, 138.27, 134.61, 134.58, 131.8, 130.2, 130.1, 127.7, 116.7, 116.5, 114.4, 112.4, 112.2, 45.1, 43.6, 35.8, 34.8, 34.7, 32.6, 30.1, 28.9, 26.9, 26.8, 19.45, 19.43; ^{19}F NMR (376 MHz, $CDCl_3$): δ –118.1 (s); MS (DART) *m/z* (relative intensity, %): 337 ($[M+H]^+$, 10), 283 (18), 256 (13), 239 (100), 195 (95), 151 (12), 102 (43); HRMS (DART) *m/z* $[M+H]^+$ Calcd for $C_{21}H_{31}ClF$ 337.2098; Found 337.2112.

The four-component coupling reaction of *n*-OctF (1a), *o*-TolMgBr (2j), and isoprene

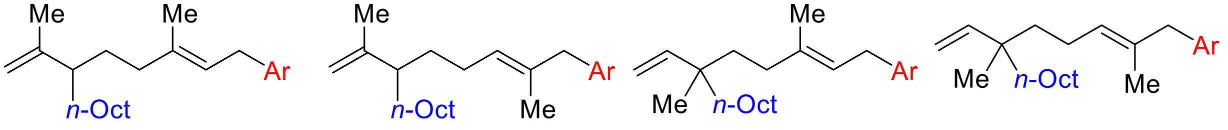

1 1.3 1.4 1.5

An oven-dried test tube was charged with $NiBr_2$(dme) (7.4 mg, 4 mol%) and a stirring bar and closed with a septum cap. The test tube was evacuated and back-filled with nitrogen for three times. 1-Fluorooctane (75.3 mg, 0.57 mmol) and THF (0.46 mL) were added via syringe at ambient temperature. 2-Methylphenylmagnesium bromide (in THF, 0.82 M, 1.04 mL, 0.86 mmol) and isoprene (170 mL, 1.7 mmol) were added at –78 °C and then stirred at 40 °C. After 10 h, aqueous work-up was carried out according to the general procedure. Purification by GPC gave the desired product **3aj'** as an inseparable mixture of four isomers (146.5 mg, 76%). Ratio of isomers was determined to be 1:1.3:1.4:1.5 by GC and NMR analyses: ^{1}H NMR (400 MHz, $CDCl_3$): δ 7.13–7.09 (m, 4H), 5.67 (dd, J = 17.6, 10.8 Hz, 0.57H), 5.22 (t, J = 7.2 Hz, 0.54H), 5.05-4.95 (m, 0.46H), 4.96 (d, J = 10.8 Hz, 0.57H), 4.87 (d, J = 17.6 Hz, 0.57H), 4.71 (s, 0.43H), 4.63 (s, 0.43H), 3.29 (d, J = 6.8 Hz, 1.10H), 3.26 (s, 0.90H), 2.28 (s, 1.6H), 2.26 (s, 0.5H), 2.25 (s, 0.9H), 2.02-1.96 (m, 2.5H), 1.70 (s, 1.6H), 1.57 (s, 2.7H),

1.45-1.15 (m, 15H), 0.94 (s, 1.6H), 0.88 (t, *J* = 6.8 Hz, 3H); ^{13}C NMR (100 MHz, $CDCl_3$): δ 147.69, 147.66, 147.5, 140.0, 138.4, 138.3, 136.95, 136.88, 136.85, 136.4, 136.21, 136.17, 133.1, 132.8, 130.02, 129.98, 129.68, 129.66, 128.56, 128.55, 126.6, 126.4, 126.0. 125.91, 125.87, 125.7, 122.1, 121.7, 111.42, 111.38, 111.36, 111.33, 47.03, 46.96, 43.22, 43.20, 40.84, 40.81, 39.5, 39.4, 39.3, 37.6, 34.3, 33.5, 33.4, 32.12, 32.08, 31.9, 31.8, 30.5, 29.7, 29.63, 29.60, 29.4, 27.5, 26.0, 24.00, 23.98, 22.8, 22.7, 22.48, 22.45, 19.5, 19.4, 17.9, 17.8, 16.41, 16.36, 16.2, 14.1; MS (EI) *m/z* (relative intensity, %): 340 ($[M]^+$, 10), 235 (10), 207 (19), 157 (50), 105 (100); HRMS (EI) *m/z* $[M]^+$ Calcd for $C_{25}H_{40}$ 340.3130; Found 340.3123.

The reaction of 1,1-diphenyl-6-fluorohex-1-ene (1n) with 4-fluorophenylmagnesium bromide (2a)

The representative general procedure was followed in a 0.6 mmol scale using 1,1-diphenyl-6-fluorohex-1-ene (152.5 mg, 0.60 mmol), 4-fluorophenylmagnesium bromide (in THF, 0.80 M, 1.5 mL, 1.2 mmol) and 1,3-butadiene (40 mL as gas, 1.8 mmol) in THF (total 1.8 mL) at 30 °C for 42 h. Purification by GPC gave the desired product **3na** as a colorless oil (98.2 mg, 37%) accompanied by cross-coupling product **4na** as a colorless oil (74.9 mg, 38%).

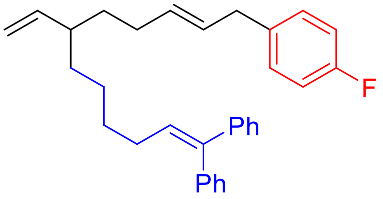

(*E*)-1,1-Diphenyl-12-(4-fluorophenyl)-7-vinyldodec-1,10-diene (3na):
^{1}H NMR (400 MHz, $CDCl_3$): δ 7.35 (t, *J* = 7.2 Hz, 2H), 7.30–7.19 (m, 6H), 7.17–7.15 (m, 2H), 7.12–7.09 (m, 2H), 6.95 (t, *J* = 8.8 Hz, 2H), 6.06 (t, *J* = 7.6 Hz, 1H), 5.49–5.47 (m, 3H), 4.96–4.88 (m, 2H), 3.27 (d, *J* = 5.2 Hz, 2H), 2.09 (q, *J* = 7.6 Hz, 2H), 2.03–1.88 (m, 3H), 1.45–1.16 (m, 8H); ^{13}C NMR (100 MHz, $CDCl_3$): δ 162.5, 160.1, 143.0, 142.9, 141.5, 140.3, 136.64, 136.61, 132.1, 130.2, 130.0, 129.8, 129.7, 128.7, 128.11, 128.06, 127.2, 126.81, 126.75, 115.1, 114.9, 114.4, 43.6, 38.2, 34.72, 34.69, 30.1, 30.0, 29.7, 26.8; ^{19}F NMR (376 MHz, $CDCl_3$): δ –117.6 (s); MS (EI) *m/z* (relative intensity, %): 438 ($[M]^+$, 33), 329 (11), 316 (12), 233 (12), 219 (14), 206 (31), 205 (49), 194 (18), 193 (100); HRMS (EI) *m/z* $[M]^+$ Calcd for $C_{32}H_{35}F$ 438.2723; Found 438.2720.

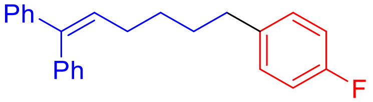

1,1-Diphenyl-6-(4-fluorophenyl)hex-1-ene (4na):
^{1}H NMR (400 MHz, $CDCl_3$): δ 7.38–7.34 (m, 2H), 7.31–7.19 (m, 6H), 7.17–7.14 (m, 2H), 7.08–7.04 (m, 2H), 6.93 (t, *J* = 8.8 Hz, 2H), 6.05 (t, *J* = 7.6 Hz, 1H, C=C*H*), 2.51 (t, *J* = 7.6 Hz, 2H), 2.13 (q, *J* = 7.6 Hz, 2H), 1.59 (quin, *J* = 7.6 Hz, 2H), 1.45 (quin, *J* = 7.6 Hz, 2H); ^{13}C NMR (100 MHz, $CDCl_3$): δ 162.3, 142.7, 141.7, 140.2, 138.0, 129.9, 129.8, 129.7, 129.6, 128.1, 128.0, 127.2, 126.85, 126.79, 115.0, 114.8, 34.8, 31.0, 29.5, 29.3; ^{19}F NMR (376 MHz, $CDCl_3$): δ –117.9 (s); MS (EI) *m/z* (relative intensity, %): 330 ($[M]^+$, 66), 194 (17), 193 (100); HRMS (EI) *m/z* $[M]^+$ Calcd for $C_{24}H_{23}F$ 330.1784; Found 330.1782.

(*E*)-12-(2,6-Dimethylphenyl)-1,1-diphenyl-7-vinyldodec-1,10-diene (3nm):[S1]

Following the general procedure, reaction was conducted using 1,1-diphenyl-6-fluoro-1-hexene (141.7 mg, 0.56 mmol) and 2,6-dimethylphenylmagnesium bromide (in THF, 0.83 M, 1.0 mL, 0.83 mmol). Purification by GPC gave the desired product as a pale yellow oil (233.0 mg, 93%): ^{1}H NMR (400 MHz, $CDCl_3$): δ 7.37–7.14 (m, 10H), 6.99 (br s, 3H), 6.05 (t, J = 7.6 Hz, 1H), 5.48–5.39 (m, 2H), 5.30–5.21 (m, 1H), 4.93–4.84 (m, 2H), 3.31 (d, J = 4.8 Hz, 2H), 2.28 (s, 6H), 2.08 (q, J = 7.6 Hz, 2H), 2.00–1.82 (m, 3H), 1.45–1.10 (m, 8H); ^{13}C NMR (100 MHz, $CDCl_3$): δ 143.1, 142.9, 141.4, 140.3, 137.1, 136.5, 130.8, 130.2, 129.9, 128.1, 128.0, 127.9, 127.2, 126.8, 126.7, 126.5, 125.8, 114.3, 43.5, 34.7, 34.6, 32.6, 30.1, 29.9, 29.7, 26.7, 19.9; MS (DART) *m/z* (relative intensity, %): 449 ($[M+H]^+$, 86), 419 (20), 371 (42); HRMS (DART) *m/z* $[M+H]^+$ Calcd for $C_{34}H_{41}$ 449.3208; Found 449.3226.

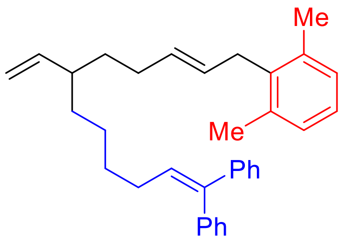

Cartesian coordinates (x, y, z) of optimized structures

Figures 10 and 11

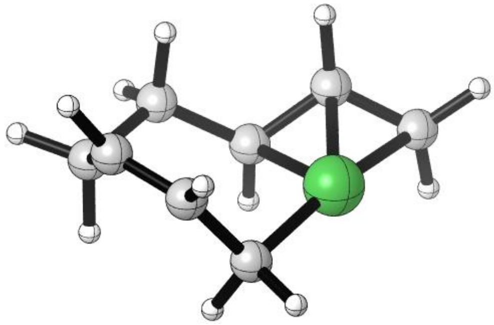

Ni complex

Ni	7.82182800	11.51124300	-0.19678300
C	6.30567000	10.34705100	0.36735000
C	7.32104200	10.43260200	1.32106500
C	7.80983700	11.73490100	1.68223100
C	9.03851000	11.85271000	2.53109300
C	10.04243300	12.95690600	2.13191800
C	10.86499700	12.56338300	0.94827000
C	10.37210900	12.52595200	-0.30020000
C	9.00151700	12.91344500	-0.67033700
H	5.47438800	11.05762200	0.37488200
H	6.06613200	9.37368300	-0.05746300
H	7.94208600	9.56022600	1.54122000
H	7.06370300	12.51912000	1.86831900
H	9.57294200	10.89112600	2.54054200
H	8.71041500	12.03681300	3.56520400
H	9.48265400	13.88680900	1.94470700
H	10.68977400	13.15934100	2.99378200
H	11.88300400	12.22290300	1.13440600
H	11.02156600	12.15018500	-1.09435300
H	8.90602500	13.05201900	-1.76218700
H	8.69076800	13.84856000	-0.18386600

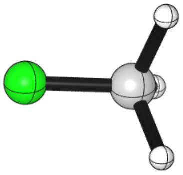

MeF

C	-0.26568700	0.05489700	0.09251400
H	-0.20497400	0.45747000	-0.92470600
H	-0.17096400	-1.03643800	0.06874500
H	0.52412500	0.48777500	0.71603300
F	-1.49078200	0.38613700	0.63198000

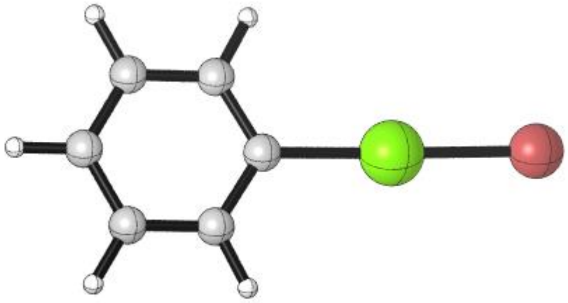

PhMgBr

Mg	1.51629300	0.16411400	-0.26385300
Br	3.49858800	0.49411600	0.89375300
C	-0.22890600	-0.11600600	-1.32706900
C	-0.30236500	0.21133100	-2.69254200
C	-1.39874400	-0.63436700	-0.74528600
C	-1.46708900	0.03520900	-3.43445500
H	0.56949600	0.61886600	-3.20848900
C	-2.56959700	-0.81561700	-1.47601200
H	-1.41198900	-0.91036400	0.31079000
C	-2.60545400	-0.47999900	-2.82507200
H	-1.48573900	0.30077500	-4.48974400
H	-3.45628900	-1.22040400	-0.99165800
H	-3.51858300	-0.62004100	-3.39952100

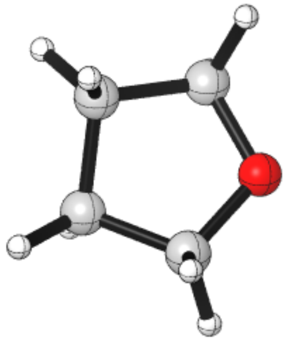

THF

O	0.68414200	2.64743300	0.40283900
C	1.84408000	3.19378200	1.00814000
C	0.46138100	3.26319600	-0.85584400
C	2.17056600	4.45933600	0.23124600
H	1.64066500	3.37456700	2.07119000
H	2.67622700	2.47156600	0.94427800
C	1.71680700	4.06427000	-1.16896600
H	0.24526200	2.48879900	-1.60313200
H	-0.41984900	3.92390700	-0.79440100
H	3.22604200	4.74160900	0.29300000
H	1.56957900	5.30063600	0.60066200
H	2.47186200	3.42409600	-1.64394700
H	1.52816900	4.91443900	-1.83165100

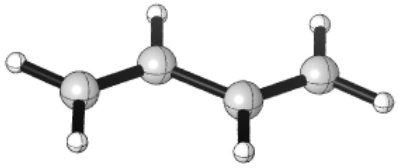

1,3-butadiene

C	-1.14956300	1.40181200	0.00303800
C	-1.98367000	1.21461800	1.02898800
H	-1.53044900	1.36468700	-1.01984500
H	-1.62743600	1.24665000	2.05748500
H	-3.04273300	1.02435900	0.88276000
C	0.27177600	1.65907500	0.14967600
C	1.10556300	1.84591800	-0.87649500
H	0.65285400	1.69612600	1.17247500
H	2.16474600	2.03605900	-0.73095500
H	0.74881100	1.81368100	-1.90482300

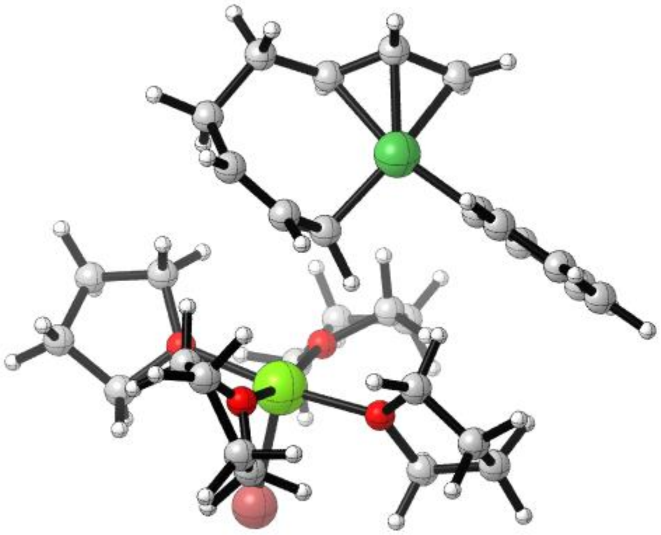

INT1

Ni	9.59696200	13.09589300	-0.81780500
C	7.71356900	13.60616500	-0.66650700
C	8.23706700	13.23686300	0.58760100
C	9.42501100	13.81794000	1.07297500
C	10.06712300	13.29747000	2.32244000
C	11.59912400	13.14836900	2.23280500
C	12.01094200	11.99502500	1.37304800
C	11.93272200	11.99606400	0.02182100
C	11.57156100	13.10282300	-0.85567700
C	9.33358300	12.52634500	-2.60000900
C	9.12502500	13.35220200	-3.72047800

C	8.93185400	12.85396000	-5.00839200	H	10.52811900	17.13806300	-3.60618100
C	8.91429400	11.47913400	-5.23438700	O	15.02604900	14.40992800	-0.57419600
C	9.08276500	10.62399600	-4.14736300	C	16.44626800	14.57390500	-0.34863200
C	9.29092000	11.14263800	-2.86988800	C	14.29168600	14.81066200	0.60402000
H	7.71895500	14.65486900	-0.98083600	C	16.57693200	14.74033800	1.14975500
H	6.89797100	13.02191700	-1.08625600	H	16.96188700	13.70243200	-0.76625800
H	7.91826200	12.28859200	1.03291200	H	16.78405000	15.46236800	-0.89846700
H	9.58812900	14.88941500	0.88935800	C	15.30679500	15.51351300	1.48009900
H	9.63725300	12.31556300	2.57386700	H	13.45271800	15.44276800	0.28781300
H	9.83812500	13.96262100	3.17052100	H	13.89311500	13.90416800	1.08048200
H	11.99196900	14.10635800	1.85376600	H	17.49742800	15.25911200	1.43166600
H	12.00920100	13.02966500	3.24566600	H	16.56518700	13.76199200	1.64923800
H	12.20721700	11.04947000	1.88130700	H	15.41458600	16.56531800	1.18202900
H	12.10919300	11.03625900	-0.48206700	H	15.02990700	15.48440500	2.53761200
H	11.93430700	12.95951400	-1.88410800	O	14.96289800	11.77799900	-1.81842000
H	11.86829500	14.08874900	-0.47122900	C	15.76157800	10.99303900	-2.72084800
H	9.08903400	14.43753200	-3.58216600	C	15.18899900	11.22067300	-0.51699900
H	8.77506700	13.54252300	-5.83979200	C	15.65780000	9.56048100	-2.19471600
H	8.75289400	11.08203200	-6.23541000	H	15.36563800	11.15234900	-3.72913600
H	9.05108400	9.54411800	-4.29749900	H	16.79332000	11.37371700	-2.69297600
H	9.43622900	10.43463100	-2.04889000	C	15.11786300	9.72309600	-0.75985900
Mg	14.27386200	13.67457300	-2.39796600	H	16.18973800	11.53057200	-0.16877600
Br	16.22546600	14.41882400	-3.73150500	H	14.41712000	11.60462600	0.16085700
O	13.10316800	15.35999900	-2.60699500	H	16.63515500	9.06869900	-2.21638500
C	13.70013600	16.67309300	-2.67885900	H	14.97866500	8.95713700	-2.80629600
C	11.73655900	15.40252000	-3.09355000	H	15.68810000	9.15627200	-0.01851900
C	12.51665300	17.61069200	-2.77033300	H	14.07356300	9.39471600	-0.69851000
H	14.33791000	16.79384200	-1.79396700	O	13.33530900	12.69272500	-3.99091600
H	14.34276500	16.71871200	-3.56977400	C	12.53094800	11.48489100	-3.93995100
C	11.56792600	16.80147800	-3.64925300	C	13.50685800	13.11225300	-5.36656500
H	11.60255000	14.59861400	-3.82732300	C	12.51208400	10.97416100	-5.36315500
H	11.06700200	15.19137400	-2.24783800	H	12.99184600	10.81053600	-3.20701900
H	12.78423400	18.58384600	-3.19144200	H	11.52331600	11.74614900	-3.59524800
H	12.07490000	17.77093500	-1.77776800	C	12.51002300	12.28210100	-6.14683100
H	11.89866000	16.84531900	-4.69584300	H	13.34829200	14.19735800	-5.40993900

H	14.54696000	12.91387500	-5.65945000
H	11.62719900	10.35901300	-5.55250100
H	13.41489000	10.38734500	-5.58808400
H	11.50711400	12.72843900	-6.09932800
H	12.79631300	12.17032400	-7.19651300

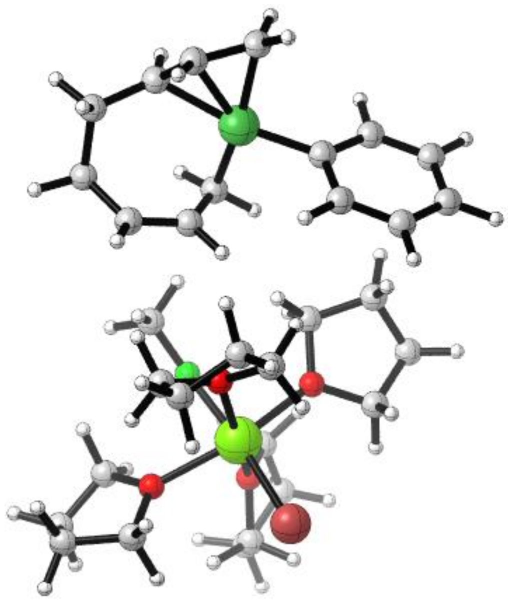

INT2

Ni	8.57574900	11.32548200	-0.48656200
C	7.13456300	10.03129000	-0.22507200
C	7.77316500	10.32570700	0.99872100
C	7.96441800	11.65410900	1.42960700
C	8.86072400	11.93110600	2.60427400
C	10.13151900	12.77935800	2.32658000
C	11.01812100	12.16781800	1.29464300
C	10.69391600	12.23823700	-0.03261300
C	9.60874000	12.98290600	-0.62471700
C	8.87368400	10.84372100	-2.29343000
C	8.03653400	11.20930500	-3.36539300
C	8.28813300	10.84048300	-4.68526200
C	9.40783800	10.07403100	-5.00413400
C	10.26435000	9.69093800	-3.97487200
C	9.99648000	10.07497800	-2.65983000
H	6.24831100	10.59341600	-0.53536600
H	7.16166300	9.00772700	-0.59241800
H	8.41020000	9.55756300	1.45113900
H	7.16346300	12.38143100	1.25666300
H	9.20648700	10.97050300	3.01840900
H	8.29348100	12.42303900	3.40946800
H	9.81917800	13.78993200	2.01261100
H	10.66322700	12.90749400	3.28085100
H	11.73129500	11.40785200	1.62671800
H	11.31898000	11.66845900	-0.72944900
H	9.73112800	13.29158900	-1.66660000
H	9.17907800	13.77773600	-0.00604000
H	7.13920600	11.79723700	-3.15593700
H	7.59826000	11.14423000	-5.47356600
H	9.60181800	9.77190300	-6.03215100
H	11.14086800	9.07818800	-4.19452500
H	10.67931700	9.72356200	-1.87901600
C	12.26256700	15.10857400	-0.29359800
H	12.38835000	15.97533400	0.35883300
H	12.47531900	14.16643000	0.22160000
H	11.25061300	15.05598700	-0.70373100
F	13.17020500	15.27313500	-1.37542900
Mg	14.63454900	14.09284200	-2.48750600
Br	16.43454000	12.90900100	-3.65821500
O	14.92781600	16.01200500	-3.37825500
C	16.21655000	16.48170600	-3.81595400
C	13.89329300	16.91628100	-3.80878200
C	15.99776300	17.95052800	-4.10612100
H	16.93686900	16.26648300	-3.01885400
H	16.51612700	15.91881600	-4.71114900
C	14.59289500	17.92766400	-4.69779900
H	13.11450300	16.33533000	-4.31652800
H	13.45204000	17.37876400	-2.91422900

H	16.75160100	18.36593100	-4.78033300
H	16.01144800	18.53113300	-3.17313700
H	14.62926100	17.56357700	-5.73266700
H	14.09491300	18.90105100	-4.69789800
O	15.93322700	14.83464900	-0.94945300
C	17.24090800	14.30808000	-0.61324900
C	15.64007400	15.98569800	-0.13615500
C	17.57671600	14.95525400	0.71285700
H	17.17485800	13.21575200	-0.59297100
H	17.94705300	14.58847300	-1.40639100
C	16.94657100	16.33233600	0.54787400
H	15.24630500	16.77662600	-0.78617200
H	14.86292700	15.70653700	0.59137800
H	18.65259600	14.98126000	0.90515600
H	17.09385600	14.41315500	1.53752800
H	17.56764700	16.95831000	-0.10730200
H	16.79739300	16.86822900	1.48924600
O	13.86930100	12.47928800	-1.41454000
C	13.59181700	11.24229900	-2.14135500
C	14.31758200	12.15659000	-0.07907100
C	13.81185800	10.13003500	-1.13304200
H	12.56566300	11.28526900	-2.53110800
H	14.30881900	11.18564900	-2.97154600
C	14.84128900	10.74330200	-0.19020300
H	15.06355500	12.90620500	0.21171900
H	13.45152600	12.22237500	0.59859300
H	14.15319300	9.20917000	-1.61389700
H	12.88371500	9.90806800	-0.58894800
H	15.83617800	10.74166200	-0.65721600
H	14.90113800	10.23950200	0.77891100
O	13.06130900	13.87835900	-3.82761700
C	11.65701500	13.85097900	-3.47293400
C	13.22679000	13.58380800	-5.23347200
C	10.96494200	13.24329500	-4.66963200
H	11.54052200	13.27162900	-2.55209900
H	11.33431600	14.88685600	-3.27962900
C	11.83743100	13.73688300	-5.81867700
H	13.98598300	14.27063100	-5.62962400
H	13.60542900	12.55776000	-5.33731100
H	9.91356400	13.53607700	-4.73724300
H	10.98619000	12.14684600	-4.60449500
H	11.62631800	14.79259000	-6.04326400
H	11.71018400	13.16212700	-6.74013900

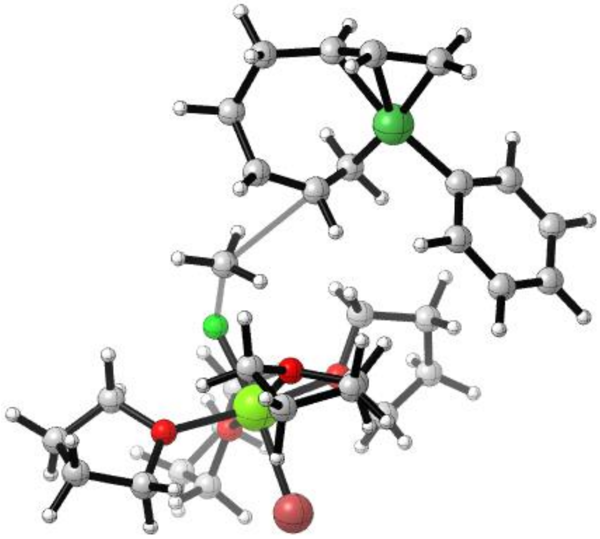

TS3

Ni	8.42964400	11.32610300	-0.44008500
C	7.14836300	9.95326800	0.09245600
C	7.66900600	10.61146100	1.22983800
C	7.66662200	12.01461400	1.32731800
C	8.44954900	12.69969100	2.41111700
C	9.61857200	13.61110500	1.94712100
C	10.61598400	12.87392800	1.11579600
C	10.28104500	12.50326500	-0.18415700
C	9.23617400	13.03307700	-0.98926000
C	8.87589900	10.53815400	-2.10842600
C	8.09442600	10.67429000	-3.27048800

C	8.51064700	10.21780800	-4.51889600	H	12.94010300	16.53249600	-3.90239000
C	9.74655100	9.59030800	-4.66503200	H	13.40652500	17.44496200	-2.44735100
C	10.54355600	9.42091200	-3.53655900	H	16.50084000	18.67459600	-4.49518800
C	10.10915000	9.88710400	-2.29456800	H	15.90369300	18.66390600	-2.82128500
H	6.21863400	10.30445500	-0.36550400	H	14.30783300	17.92615200	-5.33311000
H	7.32315100	8.88607800	-0.02026400	H	13.85616300	19.14465500	-4.12605800
H	8.37093300	10.06829500	1.87152100	O	16.06664500	14.73470200	-0.90319700
H	6.80451700	12.56495900	0.93373500	C	17.40439700	14.20801800	-0.76914400
H	8.88827300	11.93260800	3.06860000	C	15.86200000	15.81512200	0.02637200
H	7.77662100	13.29716400	3.04469900	C	17.88342900	14.73259800	0.56734400
H	9.20372800	14.46546600	1.38700900	H	17.35186900	13.11808100	-0.84869000
H	10.08770900	14.03757900	2.84562800	H	18.01522500	14.57235000	-1.60575300
H	11.31717700	12.22872900	1.65643400	C	17.23430100	16.11054600	0.59896500
H	10.98245600	11.82641600	-0.69240900	H	15.40401000	16.65087000	-0.51430900
H	9.37532700	13.04974100	-2.07073400	H	15.15696300	15.47793800	0.79975700
H	8.68023400	13.88885400	-0.59976400	H	18.97385400	14.75445100	0.64461500
H	7.11894500	11.16153500	-3.19738400	H	17.49365900	14.11023300	1.38477700
H	7.86408200	10.34910400	-5.38671900	H	17.78310600	16.80031600	-0.05678400
H	10.07343500	9.22422600	-5.63674800	H	17.18547800	16.55735900	1.59595300
H	11.50604900	8.91138100	-3.61986900	O	13.94720200	12.32001600	-1.57714500
H	10.75639000	9.70931000	-1.42895100	C	13.65470400	11.18661300	-2.44400700
C	12.28760900	14.36255200	-0.08705900	C	14.53226200	11.84476200	-0.35330400
H	12.72419000	14.54278400	0.89047100	C	14.11014100	9.95906300	-1.67172500
H	12.41949100	13.38348800	-0.50899200	H	12.57822700	11.18105800	-2.66369900
H	11.37004200	14.88853500	-0.32379900	H	14.23247400	11.33165600	-3.36444300
F	13.34744200	15.15903900	-1.08054300	C	15.17022200	10.52831100	-0.73369800
Mg	14.57683500	14.21224200	-2.37868600	H	15.22438000	12.61764200	0.00768000
Br	16.33188100	13.14214800	-3.79142400	H	13.73410000	11.71010800	0.39645000
O	14.83811200	16.15570900	-3.18854600	H	14.49182700	9.17917500	-2.33647300
C	16.07573800	16.69220400	-3.68217200	H	13.27878000	9.53476200	-1.09259100
C	13.76008300	17.07895700	-3.42034100	H	16.10425900	10.71300100	-1.28165200
C	15.81458500	18.17856700	-3.80331100	H	15.38092000	9.89030700	0.12930100
H	16.86359500	16.41244900	-2.97399800	O	12.99149500	13.99473100	-3.75664400
H	16.30656700	16.23247300	-4.65387900	C	11.60306200	13.79725100	-3.40884000
C	14.36197200	18.18548200	-4.26757100	C	13.17638500	13.82409300	-5.17858000

C	10.96682700	13.20040200	-4.64561500
H	11.54328600	13.14559600	-2.53224400
H	11.16798200	14.77409600	-3.14359200
C	11.77549100	13.86624000	-5.75417400
H	13.85376100	14.61598400	-5.52157600
H	13.66849100	12.85859100	-5.35874700
H	9.89109900	13.39231800	-4.69473000
H	11.10067200	12.10848800	-4.65588500
H	11.44734700	14.90590600	-5.89504900
H	11.70634900	13.35426700	-6.71794400

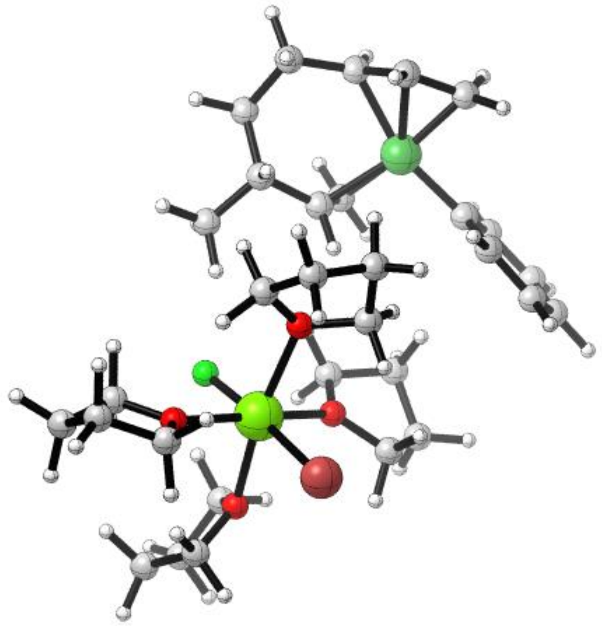

INT4

Ni	8.97481800	11.14443300	-0.58727200
C	8.31182800	9.34299300	-0.25027700
C	8.83641400	9.87019500	0.95339800
C	8.42539400	11.13166400	1.38596100
C	9.10402900	11.86357400	2.50041700
C	9.61474800	13.24051300	2.08638300
C	10.66922200	13.17332600	0.98962800
C	10.17630700	12.79273600	-0.38193100
C	8.95304500	13.12892100	-0.92213500
C	9.10678400	10.70894000	-2.43940200
C	8.18591000	11.13437400	-3.40817400
C	8.34954000	10.84657400	-4.76201200
C	9.46314700	10.13441700	-5.19763200
C	10.39050200	9.68909800	-4.26051300
C	10.19251300	9.95300700	-2.90585400
H	7.24336600	9.43894100	-0.45949000
H	8.77396800	8.46732100	-0.69836200
H	9.78093900	9.47975500	1.34547900
H	7.39254800	11.43966600	1.17392200
H	9.95876900	11.26682600	2.85562700
H	8.42223900	11.96540900	3.35675700
H	8.77827800	13.88264300	1.76824100
H	10.05570300	13.74173100	2.95886100
H	11.40887200	12.41035700	1.29840900
H	10.99545900	12.57365500	-1.07711800
H	8.83351700	13.25857800	-1.99577600
H	8.18646800	13.59737200	-0.30136800
H	7.31273800	11.71677700	-3.10490700
H	7.60845400	11.19075400	-5.48215400
H	9.60180200	9.91979100	-6.25533600
H	11.26192200	9.11915800	-4.58099200
H	10.91519000	9.55249300	-2.18996300
C	11.39503000	14.51594000	0.84736800
H	11.88452700	14.78646600	1.79228700
H	12.15234400	14.53198300	0.05096300
H	10.66515800	15.30280800	0.61193100
F	13.44398700	15.45220900	-1.45015800
Mg	14.44633200	14.22930300	-2.40640600
Br	16.06521400	12.62738200	-3.51117400
O	15.02122000	15.78503700	-3.83529000
C	16.34294800	16.33279200	-3.86261500
C	14.04290400	16.81816900	-4.04625500

C	16.15272000	17.83644400	-3.77376900
H	16.90481200	15.90913200	-3.02269100
H	16.83432500	16.02516300	-4.79640000
C	14.83231900	18.02718100	-4.51187500
H	13.31905500	16.44745700	-4.78470300
H	13.52172100	16.97509500	-3.09141900
H	16.98657700	18.39654900	-4.20761400
H	16.04435200	18.14153300	-2.72338200
H	14.99749800	17.98863000	-5.59656200
H	14.33096500	18.97122200	-4.27845500
O	16.06952300	14.75805800	-1.00483200
C	17.35938100	14.16343800	-0.77514600
C	15.85608600	15.85859800	-0.10328100
C	17.84577400	14.77713800	0.52392100
H	17.24393000	13.07424200	-0.75700600
H	18.01714200	14.40814900	-1.62147100
C	17.22980300	16.16924400	0.45375300
H	15.37651900	16.66726200	-0.66168100
H	15.15154500	15.53894000	0.67970800
H	18.93622100	14.77332200	0.60990700
H	17.43351800	14.22930300	1.38309700
H	17.79343200	16.79774800	-0.25104300
H	17.19040200	16.68819400	1.41614100
O	13.56044500	12.57187600	-1.28351700
C	13.24508700	11.33112400	-1.95378400
C	14.14725100	12.29845700	-0.00289800
C	13.31574800	10.27526700	-0.87062000
H	12.25816900	11.43611000	-2.42190700
H	14.00401600	11.16383200	-2.73221700
C	14.45153100	10.81257800	-0.00604100
H	15.02733600	12.93973500	0.11944200
H	13.42188700	12.56454300	0.78122100
H	13.50662500	9.27659000	-1.27570900
H	12.37681300	10.23588700	-0.29416200
H	15.41468100	10.62748400	-0.49973600
H	14.48611800	10.38567700	1.00099500
O	12.83950400	13.90689500	-3.75858600
C	11.51591200	14.39074100	-3.48819800
C	12.89767700	13.29439300	-5.06004600
C	10.63932000	13.58429700	-4.41839200
H	11.32139200	14.26393500	-2.41876800
H	11.47077600	15.46872600	-3.71254000
C	11.51814800	13.50873300	-5.66199100
H	13.72139300	13.75501600	-5.61773500
H	13.12716100	12.22767700	-4.92882000
H	9.65975200	14.04090600	-4.59580100
H	10.47488700	12.57927500	-4.00416800
H	11.47841000	14.45905500	-6.21266800
H	11.23309500	12.70563500	-6.34837800

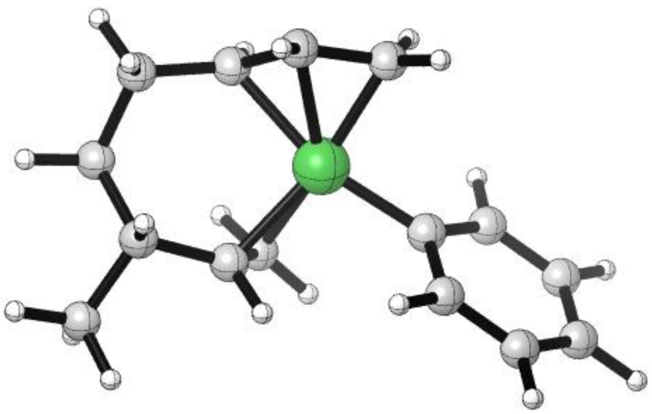

INT5

Ni	8.60993500	11.36927200	-0.48948200
C	7.30545200	9.99563800	-0.03448000
C	7.77371400	10.59655500	1.15755700
C	7.78298100	11.98736500	1.27847000
C	8.50959200	12.69228200	2.38100900
C	9.54678500	13.69039500	1.87179100
C	10.64548700	13.03915600	1.04052500
C	10.24382700	12.56614500	-0.33353600

C	9.29277900	13.14260800	-1.14973700
C	8.92198100	10.52834400	-2.16464800
C	8.00697200	10.57766300	-3.22523900
C	8.28649500	10.01146800	-4.46614700
C	9.49780500	9.36121100	-4.68152300
C	10.41922700	9.28666300	-3.64389500
C	10.13161100	9.86460000	-2.40822800
H	6.40474100	10.38424000	-0.51900400
H	7.48487700	8.93913600	-0.20933500
H	8.43613900	10.02580900	1.81527900
H	6.97417500	12.55547200	0.80006500
H	9.02245900	11.94696200	3.00881400
H	7.79789100	13.21051400	3.03919000
H	9.05969100	14.49005500	1.29152500
H	10.01379900	14.19292300	2.73023000
H	11.00836800	12.16060400	1.60355600
H	11.01824500	11.97246900	-0.83141300
H	9.37127200	13.06760300	-2.23130100
H	8.64396400	13.93593600	-0.77377900
H	7.04292700	11.07269500	-3.08564300
H	7.55254700	10.07524500	-5.26828800
H	9.71863100	8.91401500	-5.64844400
H	11.36964500	8.77649000	-3.79490300
H	10.87742200	9.77607700	-1.61404900
C	11.82153500	14.00223300	0.86028700
H	12.24030000	14.29950900	1.82866800
H	12.62606200	13.55556400	0.26538800
H	11.48686300	14.90859600	0.33883200

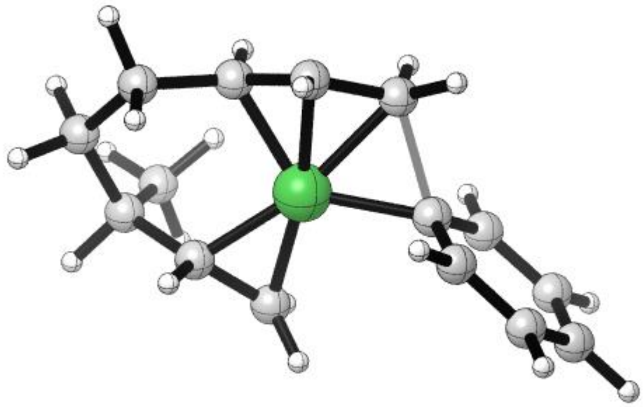

TS6

Ni	8.65606200	11.03932700	0.06690900
C	6.70722000	10.42732600	0.00644400
C	7.26584800	10.60890200	1.34537900
C	7.83856100	11.84695700	1.66470400
C	8.72010800	12.11004300	2.84259400
C	9.77669000	13.18744000	2.53522000
C	10.34874700	13.19762900	1.09324800
C	10.36880200	11.81743800	0.47664500
C	10.27760600	11.51200400	-0.89628400
C	7.88414300	9.90637800	-1.34425000
C	7.57684000	10.40861300	-2.61448200
C	7.76953000	9.63995700	-3.75528000
C	8.25729100	8.34003700	-3.65115900
C	8.53174900	7.81021400	-2.39256000
C	8.32294900	8.57661800	-1.25366200
H	6.17782100	11.29080500	-0.41091700
H	6.10452000	9.52931100	-0.11722700
H	7.47243900	9.72638100	1.95648400
H	7.35349600	12.74167000	1.25128800
H	9.21154900	11.16891500	3.12957800
H	8.13758700	12.43224900	3.71833900
H	9.34612100	14.17921400	2.73582900
H	10.59596700	13.07342800	3.25717200
H	11.40306300	13.51122400	1.18376500
H	10.90049000	11.08871700	1.10398800

H	10.77036400	10.62180400	-1.28795200
H	10.11485300	12.29290500	-1.64124600
H	7.20312700	11.42902400	-2.71019000
H	7.54313500	10.05931700	-4.73359500
H	8.40836300	7.73803100	-4.54385900
H	8.89740000	6.78951600	-2.29919800
H	8.50419800	8.14077900	-0.26906800
C	9.67222700	14.24006400	0.21109200
H	9.68577300	15.22437400	0.69340400
H	10.18130200	14.33840000	-0.75430900
H	8.62513000	13.98085200	0.00536400

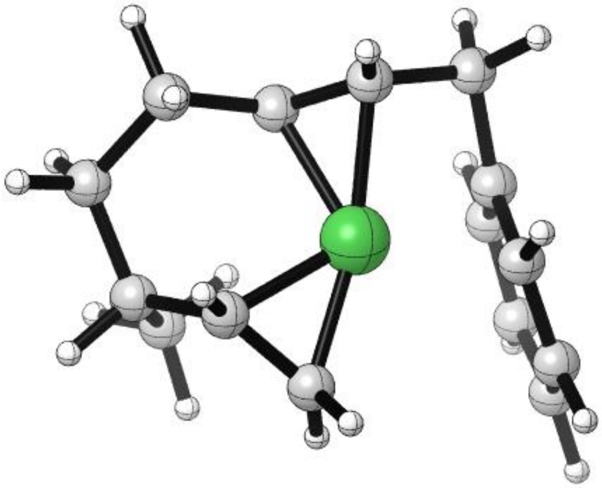

INT7

Ni	8.95824600	10.53311300	0.02186600
C	6.43337000	9.81008000	0.41967000
C	7.47971800	10.35415600	1.36267400
C	7.90751700	11.67208300	1.26384200
C	8.71481900	12.35502500	2.32528000
C	9.70598900	13.38935100	1.77983700
C	10.46401500	12.96594900	0.49786500
C	10.60412000	11.46236800	0.41615500
C	10.72296200	10.70860800	-0.76935200
C	7.17537900	9.80129400	-0.89473400
C	6.80725500	10.63960100	-1.97311800
C	7.42545500	10.52630100	-3.20111100
C	8.42476500	9.56030700	-3.41473900
C	8.81401400	8.74880300	-2.37330300
C	8.25627900	8.90185100	-1.08477000
H	5.53140200	10.43574600	0.36685800
H	6.11408600	8.79899100	0.70035400
H	7.79616600	9.74849000	2.21596000
H	7.32376800	12.34377300	0.61889700
H	9.25044100	11.58509600	2.89994300
H	8.04717400	12.85473400	3.04399700
H	9.17183100	14.32925900	1.57474000
H	10.42295800	13.62795100	2.57692200
H	11.48384600	13.38024400	0.57919700
H	11.05975400	11.03233700	1.31800500
H	11.28334000	9.77253000	-0.74634400
H	10.69938400	11.18036400	-1.75428700
H	5.99933200	11.35629500	-1.82736400
H	7.11415200	11.17059900	-4.02044800
H	8.87732300	9.45221800	-4.39689900
H	9.58350800	7.99501900	-2.52748600
H	8.41629800	8.10894500	-0.34954700
C	9.84761800	13.58135100	-0.75246800
H	9.78845100	14.67223900	-0.65902900
H	10.43960100	13.35710100	-1.64715200
H	8.83234800	13.20093900	-0.92809500

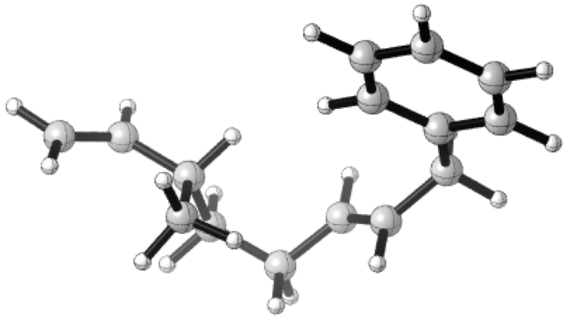

P8

C	3.33286800	0.21195300	0.31856700
C	3.61392700	1.72021300	0.36341800
H	2.41881000	0.03983700	0.91732100
C	2.50192800	2.60220300	-0.20765000
H	3.79594000	2.01002700	1.41054200
H	4.55325000	1.91884000	-0.17469200
C	1.18232600	2.36177500	0.44966800
H	2.79945700	3.65328300	-0.07509400
H	2.40501900	2.44924500	-1.29185500
H	1.16032900	2.45460600	1.54151700
C	4.45599000	-0.50679600	1.00767100
C	5.24588000	-1.44212300	0.48866200
H	4.63511500	-0.18663700	2.03809900
H	6.04772100	-1.89012300	1.06964700
H	5.12844300	-1.80396900	-0.53082000
C	0.06022700	2.02216200	-0.18343600
C	-1.24636500	1.73744300	0.48604300
H	0.07995300	1.90831000	-1.27167300
H	-2.01342500	2.44733000	0.14222100
H	-1.13675600	1.90785500	1.56817800
C	-1.74345300	0.33175000	0.23959000
C	3.07067200	-0.28255400	-1.09371200
C	-3.07997900	0.08939600	-0.07508000
C	-3.54253400	-1.20705300	-0.27467500
C	-2.66859500	-2.28296100	-0.16673800
C	-1.33122700	-2.05307500	0.14168900
C	-0.87395500	-0.75632800	0.34091000
H	-3.76651800	0.93102500	-0.16356100
H	-4.58875400	-1.37665100	-0.51956600
H	-3.02746900	-3.29719300	-0.32535100
H	-0.63947300	-2.88840300	0.22550400
H	0.17477700	-0.57521300	0.57793200
H	3.91694500	-0.04728600	-1.75378600
H	2.17205500	0.17930200	-1.51685700
H	2.91798200	-1.36770800	-1.11362700

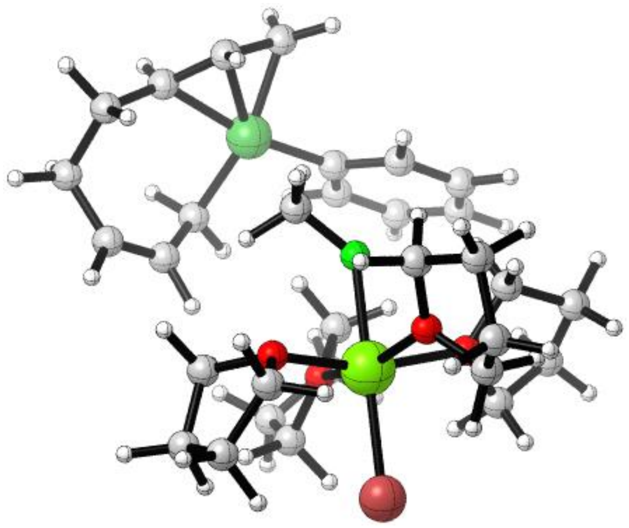

INT9

Ni	8.37965400	11.72845800	0.17481000
C	6.63660500	11.36544700	-0.65955500
C	6.59404100	11.09584000	0.72306700
C	7.03677700	12.05247300	1.65466100
C	7.13214600	11.71936100	3.11281300
C	8.32816500	12.34833400	3.85462500
C	9.63053300	11.68091900	3.55093400
C	10.26092500	11.79978500	2.36595800
C	9.83498100	12.57180100	1.19594100
C	9.33470300	11.53398600	-1.46457400
C	10.11763100	12.57379700	-2.00800400
C	10.83426700	12.45638400	-3.19808900

C	10.80821000	11.26825100	-3.92391600	O	11.50821200	7.34622200	2.75559900
C	10.02528300	10.22110100	-3.44357500	C	11.67849100	6.16609000	3.56159200
C	9.31080700	10.36414100	-2.25404500	C	11.71433200	8.45037100	3.65619000
H	6.33942700	12.34808300	-1.03445700	C	12.79387400	6.49129000	4.55285400
H	6.44437800	10.55341200	-1.35943400	H	10.72284600	5.96892400	4.07169200
H	6.52238300	10.05408800	1.06147400	H	11.90588800	5.33543000	2.88465800
H	6.81946000	13.10861400	1.44592500	C	12.88826700	8.02847100	4.52499100
H	7.18163500	10.62374800	3.23741900	H	11.87928200	9.34651900	3.04811000
H	6.20954400	12.04199800	3.62230000	H	10.79382600	8.59268600	4.24260000
H	8.36841200	13.41762400	3.58734500	H	13.73714500	6.04930200	4.21927700
H	8.13122800	12.31335800	4.93451500	H	12.55841200	6.10338800	5.54859300
H	10.06271800	11.04430200	4.32651000	H	13.83487600	8.33392500	4.06474400
H	11.18391100	11.21262500	2.24710000	H	12.83344100	8.48177600	5.51887700
H	10.69832700	12.81071200	0.55986900	O	11.33032300	5.49532200	0.49737400
H	9.37841900	13.52620300	1.50619200	C	9.94690900	5.14884700	0.69257900
H	11.41834600	13.30163300	-3.56334300	C	12.07216300	4.34885100	0.02982400
H	9.94565900	9.29421000	-4.01702100	C	9.82456400	3.69815300	0.25203300
C	9.26558700	8.75039600	1.02370200	H	9.71077100	5.28566000	1.75856800
H	9.70702900	9.23873400	1.89795000	H	9.32625000	5.83743000	0.10831100
F	10.29471200	8.08765700	0.29777000	C	11.23746200	3.16838000	0.47172900
Mg	12.27489200	7.41694300	0.77403400	H	12.15849100	4.40356300	-1.06812800
Br	14.52327700	6.57000200	1.36169500	H	13.07493600	4.41460900	0.46488700
O	12.74679200	9.44919400	0.70796900	H	9.56623800	3.64542600	-0.81384100
C	13.95084100	10.05643200	1.23697300	H	9.05551800	3.15658600	0.80964800
C	12.15189700	10.33533400	-0.29331200	H	11.45637300	2.25854100	-0.09385500
C	13.87083400	11.50862100	0.81724600	H	11.41385400	2.95996300	1.53563700
H	13.96713100	9.89080500	2.32085300	O	12.46368900	7.32872800	-1.32336300
H	14.82382900	9.54752000	0.80507900	C	11.40917700	7.10414300	-2.28218900
C	13.19641700	11.39846200	-0.54489200	C	13.70549800	7.61140500	-1.99897500
H	11.89509100	9.73644100	-1.17118100	C	12.11585300	6.99204500	-3.61788200
H	11.22691900	10.75556400	0.11683400	H	10.86359200	6.20129300	-1.97887700
H	14.85679700	11.98124700	0.78495200	H	10.72667700	7.96491600	-2.25615300
H	13.23531800	12.07927400	1.50704100	C	13.29631700	7.93809400	-3.42022100
H	13.91545200	11.06912000	-1.30897000	H	14.20251900	8.42574900	-1.45933800
H	12.73505000	12.32614300	-0.89551400	H	14.34431600	6.71863900	-1.93901600

H	11.46766200	7.27125100	-4.45379600
H	12.47231900	5.96628600	-3.78467100
H	12.95649000	8.98113900	-3.49602900
H	14.10741500	7.78366600	-4.13707500
H	11.35818100	11.17117900	-4.85889400
H	8.83848400	9.51386000	0.35911000
H	8.53545900	7.99092000	1.31677700
H	10.17888800	13.52327400	-1.47095200
H	8.70220200	9.51172800	-1.93170200

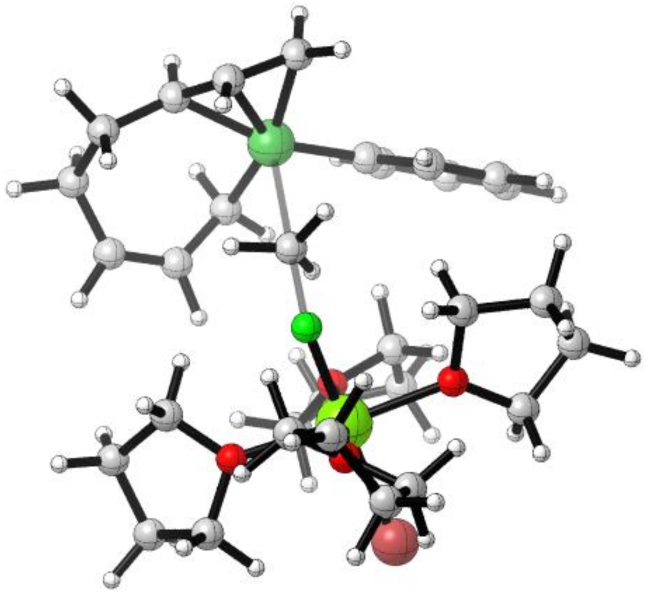

TS10

Ni	8.52439700	11.62431900	0.13657900
C	6.85221000	11.42510800	-0.90664800
C	6.64423300	11.01293500	0.42385300
C	7.02257400	11.84155100	1.49105200
C	6.93002000	11.39933700	2.91741000
C	8.02454500	11.97205200	3.83819100
C	9.35085200	11.30080800	3.66937700
C	10.13511900	11.45497000	2.58779200
C	9.88009200	12.27816500	1.40182500
C	9.61387700	11.76981300	-1.42825700
C	10.45488600	12.88362400	-1.62102000
C	11.30037700	13.01673800	-2.72187100
C	11.34813500	12.02602100	-3.69801400
C	10.50276100	10.92752900	-3.56965600
C	9.65619400	10.82103900	-2.46738500
H	6.64585700	12.45865300	-1.19489000
H	6.70817300	10.70592600	-1.71117300
H	6.50316400	9.94684800	0.63639800
H	6.91177600	12.92638500	1.35111200
H	6.95411500	10.29838100	2.97120200
H	5.95139100	11.70114300	3.32329100
H	8.10582300	13.05298900	3.63669100
H	7.69143700	11.88336900	4.88015200
H	9.66432000	10.61907300	4.46335600
H	11.05366400	10.85423900	2.55344900
H	10.82229500	12.47125400	0.87279600
H	9.44694800	13.25261500	1.68742400
H	11.93173300	13.90030800	-2.81360200
H	10.48494800	10.15967100	-4.34578200
C	9.48175500	9.38034000	0.42860000
H	10.40209100	9.90408800	0.23716300
F	10.24116200	7.80201600	0.63028100
Mg	12.17463900	7.38533500	0.61408900
Br	14.61982800	6.81105500	0.52136100
O	12.59911000	9.47924700	0.63381900
C	13.54594600	10.00337300	1.59527700
C	12.76487900	10.17529000	-0.63193600
C	14.05485800	11.28902400	0.97885700
H	13.01958400	10.14336300	2.54746900
H	14.34685100	9.25919900	1.71832900
C	14.06688600	10.93195600	-0.50316100
H	12.74110200	9.42981900	-1.43304900
H	11.91805400	10.86154600	-0.77340500
H	15.03401800	11.57785300	1.37146000

H	13.34698900	12.10894300	1.16564300
H	14.92020200	10.27961300	-0.73291800
H	14.09306300	11.80231200	-1.16595000
O	12.04198800	7.35610300	2.76387900
C	12.96813100	6.70987100	3.64481400
C	11.07393700	7.99683700	3.60187600
C	13.21467900	7.75764800	4.71203100
H	12.49124700	5.80670200	4.06373400
H	13.84015200	6.42777200	3.04648700
C	11.83890800	8.43048700	4.85997500
H	10.62167200	8.82116700	3.04317700
H	10.28151000	7.26982000	3.84114200
H	13.96149900	8.47240900	4.34648600
H	13.58823900	7.33173800	5.64774400
H	11.92468900	9.52054900	4.92795900
H	11.31897700	8.09275100	5.76262900
O	11.57625100	5.33685100	0.75246300
C	10.42141400	4.89030500	1.48106900
C	12.02517600	4.32027000	-0.15900400
C	9.96730500	3.63698400	0.75841900
H	10.72429500	4.67740900	2.51806300
H	9.68916900	5.70349200	1.48562100
C	11.29315600	3.06452700	0.26885000
H	11.74787100	4.62609600	-1.18069000
H	13.11747200	4.27525700	-0.09539400
H	9.33100400	3.90272000	-0.09698100
H	9.40162900	2.95801200	1.40291200
H	11.18671300	2.34172000	-0.54509900
H	11.82491000	2.57334000	1.09377400
O	11.99107800	7.14412700	-1.51072600
C	10.68643300	7.20510500	-2.12302500
C	13.01149200	7.04232800	-2.52723700
C	10.91427100	6.87972700	-3.58547500
H	10.02999500	6.50476700	-1.59215100
H	10.29822700	8.22499000	-1.99524500
C	12.31161800	7.44591500	-3.80768900
H	13.85370200	7.68100300	-2.24218200
H	13.37338900	6.00402900	-2.55618300
H	10.15023400	7.32452500	-4.23077600
H	10.91133200	5.79337000	-3.74952000
H	12.26315700	8.54194300	-3.88304000
H	12.81092000	7.05898900	-4.70041700
H	12.01180900	12.12081200	-4.55518300
H	8.85088100	9.09287100	-0.40328600
H	9.05280900	9.44074800	1.42277000
H	10.46850900	13.67836400	-0.87309000
H	8.99006300	9.95313900	-2.43341300

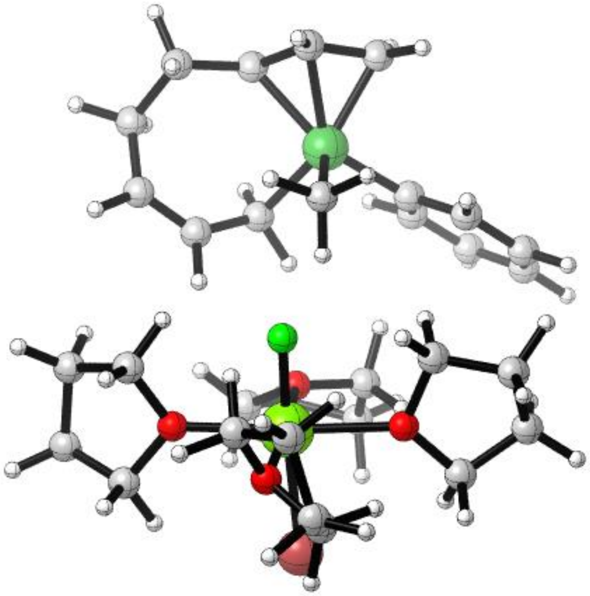

INT11

Ni	8.52576200	11.68571200	0.10552200
C	6.86726100	12.30130700	-0.87795700
C	6.50649800	11.94708700	0.43247100
C	7.21145200	12.46853700	1.52148400
C	6.98032200	12.05676500	2.93821400
C	8.23269300	12.12113600	3.83718600

C	9.15511300	10.96048900	3.64071000	H	12.80827300	9.19359400	-1.40903800
C	9.91319200	10.77757600	2.54848500	H	11.83630700	10.61765000	-0.96818500
C	10.00065000	11.66921900	1.39140100	H	14.77532700	11.67724500	1.32818900
C	9.53611800	11.67854800	-1.49762900	H	13.11190200	12.17209800	0.94614800
C	10.44414800	12.74613000	-1.55496100	H	14.84870800	10.20678200	-0.64189100
C	11.27696100	12.93440600	-2.65720800	H	14.01504700	11.64898800	-1.28330000
C	11.23571200	12.04399500	-3.72490300	O	12.25814400	7.38124100	2.85636200
C	10.33009300	10.98761900	-3.69184200	C	13.32123500	7.09465800	3.76250100
C	9.48841900	10.81665200	-2.59456800	C	11.09472300	7.50463500	3.67330100
H	7.16775300	13.33043300	-1.09591300	C	13.07405900	8.03873100	4.93929900
H	6.44827200	11.76368700	-1.72464200	H	13.24538700	6.03599200	4.06333200
H	5.94119800	11.02431300	0.59204400	H	14.25875500	7.23085800	3.21651800
H	7.62404500	13.48308500	1.39756200	C	11.56570600	8.36202200	4.84219800
H	6.56308300	11.03873200	2.96527600	H	10.29569600	7.90618900	3.04268900
H	6.21409400	12.71618300	3.37352300	H	10.79741000	6.49725500	4.01363700
H	8.75007600	13.07368400	3.63790100	H	13.67338100	8.94940800	4.83381800
H	7.90887100	12.16772400	4.88353600	H	13.34759100	7.57489300	5.89216900
H	9.15133500	10.18654700	4.41041500	H	11.40757300	9.42458400	4.61763600
H	10.48086000	9.84735200	2.45521800	H	11.01915500	8.13426800	5.76320300
H	10.89644000	11.45472000	0.80513300	O	12.06361600	5.07093100	1.02120900
H	9.98358400	12.73532600	1.68116200	C	10.82425100	4.57847400	1.55897100
H	11.97252100	13.77198400	-2.67094500	C	12.54914100	4.18128700	0.00164100
H	10.26997500	10.29497900	-4.53117000	C	10.35211900	3.54212500	0.55923100
C	8.62036300	9.77451200	0.12298200	H	11.02437900	4.12529800	2.54371300
H	9.62435700	9.34616200	0.09685000	H	10.15628500	5.43828800	1.66408900
F	10.37009800	7.38520400	0.79388000	C	11.67569500	2.94406900	0.09738500
Mg	12.21740700	7.24636500	0.71658200	H	12.42495100	4.68218200	-0.97110300
Br	14.73375100	7.01225000	0.46427000	H	13.61728500	4.01112300	0.16976700
O	12.46882200	9.43660300	0.61261700	H	9.84720600	4.04121200	-0.28026600
C	13.28407800	10.10959000	1.59238100	H	9.66008900	2.81292400	0.99151200
C	12.70795800	10.01017700	-0.68973500	H	11.61074400	2.40429500	-0.85243500
C	13.82945800	11.34098200	0.89264500	H	12.06385800	2.25145900	0.85578700
H	12.65412900	10.33214500	2.46345800	O	11.98753400	7.00989400	-1.46794600
H	14.09800700	9.43259200	1.88849800	C	10.67364200	7.26280800	-2.00691300
C	13.96153600	10.84510500	-0.54202300	C	12.95041100	6.91506000	-2.53532900

C	10.79206600	6.94833400	-3.48257600
H	9.95562100	6.66290500	-1.44278000
H	10.42908600	8.32411200	-1.84237300
C	12.21724300	7.40405200	-3.77063500
H	13.83846300	7.49553600	-2.26143800
H	13.26477400	5.86444800	-2.63152100
H	10.03278600	7.46247400	-4.08151100
H	10.69334300	5.86803300	-3.65785200
H	12.25256400	8.50224600	-3.82588800
H	12.63812900	7.00086500	-4.69657200
H	11.89506800	12.17829700	-4.57924200
H	8.01713700	9.45536900	-0.73504800
H	8.13967500	9.51790600	1.07498200
H	10.53409200	13.44239700	-0.71698000
H	8.78463900	9.98269900	-2.60523700

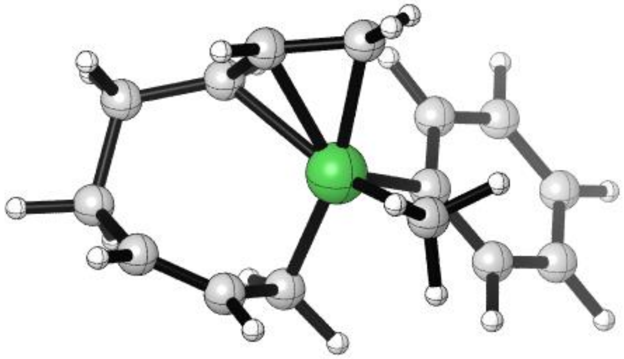

INT12

Ni	8.36219100	10.95160800	0.15327800
C	6.68814000	9.86477100	0.48693300
C	6.96418200	10.58964800	1.65976300
C	7.17463500	11.96935400	1.69548000
C	7.68686500	12.58500700	2.96191500
C	9.21224500	12.88608300	3.00419200
C	10.04693000	11.71673600	2.63402800
C	10.35825400	11.41570200	1.34874200
C	9.88954500	12.15448700	0.19087700
C	8.03579900	11.66827000	-1.58135600
C	6.79979200	12.30537700	-1.73180200
C	6.41734300	12.85410800	-2.95589000
C	7.25829400	12.77711800	-4.05705400
C	8.48873500	12.14456800	-3.91952500
C	8.86892100	11.59928100	-2.69753500
H	6.03187700	10.26565300	-0.28452600
H	6.71247000	8.77939200	0.54015200
H	7.33953300	10.02183100	2.51706200
H	6.63897500	12.62309600	1.00688300
H	7.46121400	11.91439200	3.80301700
H	7.16137700	13.52471500	3.17165200
H	9.42015500	13.72649600	2.32968800
H	9.45480300	13.22634900	4.01798800
H	10.39942200	11.06261900	3.43152500
H	10.98075200	10.53774200	1.16585300
H	10.49688300	12.04056000	-0.70717700
H	9.61904000	13.19806200	0.35860200
H	6.09874000	12.38885700	-0.90325300
H	5.44809500	13.34264900	-3.03889100
H	6.95871900	13.20286200	-5.01165400
H	9.16458500	12.07107100	-4.76951000
H	9.84003800	11.11089100	-2.63111700
C	9.06660900	9.38803200	-0.71282000
H	9.20280000	8.65532100	0.09643400
H	8.45183200	8.94031400	-1.49719300
H	10.04306800	9.66163000	-1.12947800

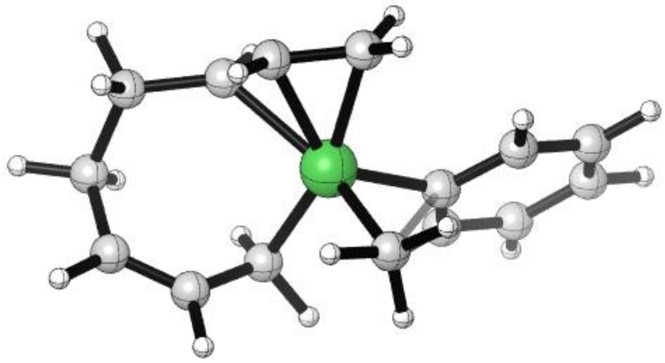

TS13

Ni	8.24608400	10.89953300	0.13421200
C	6.33603200	10.18467000	0.38724300
C	6.81828700	10.65698100	1.61092100
C	7.34741200	11.94785900	1.72878800
C	7.87651400	12.45573200	3.03205700
C	9.31413600	13.02431100	3.04094700
C	10.31508400	11.94420400	2.83288700
C	10.55612900	11.41424600	1.62107500
C	9.94770200	11.85626900	0.36553500
C	8.17432200	10.85918200	-1.76238800
C	7.25278600	10.09860200	-2.49467900
C	6.95467500	10.39453800	-3.81858500
C	7.59092600	11.45141400	-4.46113000
C	8.53046400	12.19549400	-3.76220700
C	8.83393600	11.89496800	-2.43469400
H	5.77602200	10.85450300	-0.26546100
H	6.08907900	9.12972200	0.28627000
H	7.07324700	9.94093700	2.39878200
H	6.96286400	12.71140100	1.03829800
H	7.83202500	11.64501600	3.77396900
H	7.19600400	13.23951100	3.39454100
H	9.39549300	13.80174900	2.26716000
H	9.47304600	13.52516300	4.00342300
H	10.79611400	11.51885100	3.71332400
H	11.24360700	10.56620900	1.55898700
H	10.58505700	11.60237600	-0.48783300
H	9.73833800	12.93429600	0.35615000
H	6.74910300	9.25535500	-2.02806600
H	6.22334000	9.78893600	-4.34986900
H	7.36294000	11.68413200	-5.49859300
H	9.04366100	13.02333500	-4.24760700
H	9.58197700	12.50301300	-1.93642900
C	9.11014500	9.29445800	-0.45553800
H	9.31526400	9.04982300	0.60182100
H	8.49686300	8.51483100	-0.90774600
H	10.05316700	9.40420000	-0.99470700

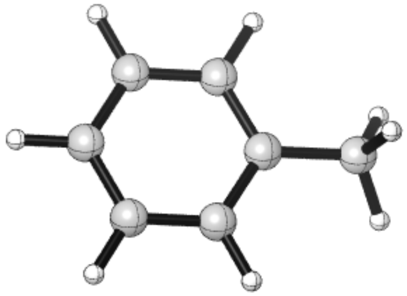

P14

C	-2.47619900	-0.74985400	-0.08564100
C	-3.29727400	-1.82215600	-0.43962800
C	-4.16023300	-2.39684400	0.48587400
C	-4.21500600	-1.90887800	1.78736500
C	-3.39937100	-0.84389000	2.15420700
C	-2.53784700	-0.27299100	1.22438700
H	-3.25491000	-2.21029900	-1.45699000
H	-4.78961700	-3.23366900	0.19090300
H	-4.88788900	-2.35928000	2.51319900
H	-3.43032800	-0.45816600	3.17084100
H	-1.89799200	0.55920200	1.51661200
C	-1.56894400	-0.11118200	-1.09461900
H	-1.18995700	-0.84258400	-1.81684600
H	-0.70843600	0.36756100	-0.61495500

H	-2.09306200	0.66498800	-1.66709200

Table 8

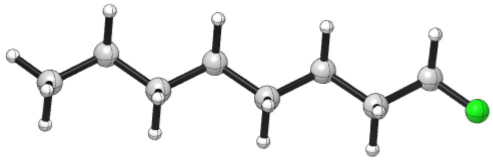

***n*-Oct-F**

C	-1.19723300	0.56006600	0.02857400
H	-0.83322800	1.09652300	-0.85945000
H	-2.29501300	0.60660800	-0.00858700
C	-0.71972500	1.28450000	1.26134900
H	0.37954800	1.27520200	1.31779200
H	-1.10405300	0.80137800	2.17283800
F	-1.14818700	2.59160800	1.24220100
C	-0.72996100	-0.88590300	-0.00311500
H	-1.07741600	-1.40866300	0.90301200
H	0.37106800	-0.91916000	0.03752700
C	-1.21015000	-1.64031000	-1.23227400
H	-2.31148300	-1.62210400	-1.26522700
H	-0.87657600	-1.11027300	-2.13910200
C	-0.72494300	-3.07969800	-1.27963100
H	-1.04897800	-3.60655800	-0.36705000
H	0.37709100	-3.09430800	-1.25541200
C	-1.21307000	-3.84088300	-2.50109900
H	-0.90112200	-3.30768300	-3.41429900
H	-2.31534000	-3.83879100	-2.51873800
C	-0.71081600	-5.27511100	-2.55823000
H	-1.01711200	-5.80241500	-1.64189000
H	0.38968300	-5.27244300	-2.54684300
C	-1.21405600	-6.02515200	-3.77935600
H	-0.90068000	-5.52554200	-4.70460700
H	-0.83920400	-7.05348700	-3.81389100
H	-2.31024200	-6.07070900	-3.78953000

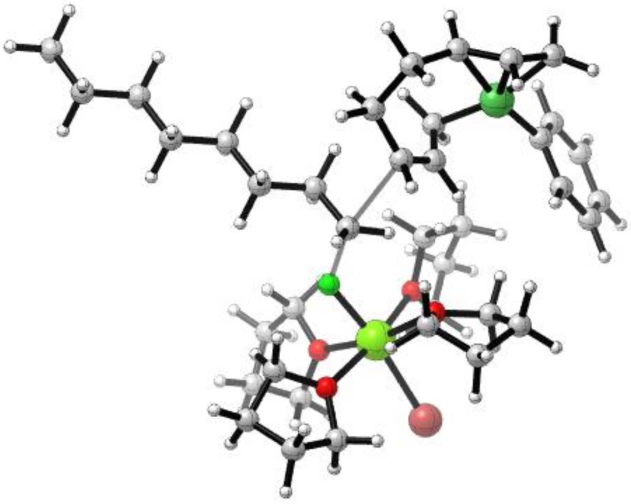

TS3-Ph-*n*-Oct

Ni	8.46359200	10.97819800	-0.52806900
C	7.35924400	9.42395000	-0.10894300
C	7.82881200	10.03108800	1.07759400
C	7.66632600	11.40975800	1.30535800
C	8.39266200	12.07709300	2.43935500
C	9.45708100	13.14258500	2.05303700
C	10.50032700	12.60199900	1.13334000
C	10.17495500	12.32319100	-0.19040500
C	9.05854500	12.80881600	-0.92679800
C	8.98460900	10.36525300	-2.24664500
C	8.27780400	10.62562800	-3.43465200
C	8.74998300	10.25139700	-4.69073500
C	9.97591300	9.60075400	-4.81839800
C	10.70536400	9.32305400	-3.66594900
C	10.21085200	9.69722200	-2.41576300
H	6.38305100	9.70295900	-0.51600700
H	7.65520800	8.40037400	-0.32657300
H	8.60451200	9.51877400	1.65678300
H	6.73648000	11.88807500	0.97763300
H	8.91515200	11.30402600	3.02455900
H	7.67324800	12.54586100	3.12754000
H	8.94077600	14.00503800	1.59506800
H	9.90587000	13.51777800	2.98423200

H	11.29508100	12.00624600	1.59392400
H	10.92997700	11.77213600	-0.76724900
H	9.17354100	12.95220400	-2.00312500
H	8.41039700	13.54722500	-0.44842800
H	7.31764400	11.14416200	-3.37555600
H	8.15860700	10.47189400	-5.57976400
H	10.34735200	9.30099100	-5.79679800
H	11.66227600	8.80205800	-3.73764500
H	10.80399800	9.43592700	-1.53205400
C	12.06123800	14.37203200	-0.04309000
H	12.61885400	14.17320700	0.86881700
H	11.98440800	13.54654300	-0.72712400
F	13.36115300	15.11183000	-0.94294400
Mg	14.47427400	14.20737700	-2.29268500
Br	16.21328100	13.17554500	-3.75999300
O	14.69300600	16.16295000	-3.07978600
C	15.90718400	16.74163600	-3.58615800
C	13.61105700	17.10528200	-3.14825100
C	15.62023600	18.22877400	-3.64725200
H	16.72157600	16.45063000	-2.91241400
H	16.11621700	16.31830900	-4.57892200
C	14.13910500	18.23429000	-4.01092100
H	12.73559300	16.58538300	-3.55833100
H	13.36674500	17.43711500	-2.12818000
H	16.25373000	18.75187200	-4.36888400
H	15.76991300	18.69090600	-2.66139900
H	14.01157100	17.99281200	-5.07479500
H	13.63795100	19.18638200	-3.81512300
O	16.04117700	14.72382200	-0.85513000
C	17.37156200	14.17812100	-0.74535000
C	15.85874600	15.78494000	0.09934300
C	17.88537800	14.69165000	0.58359000
H	17.30339800	13.08850700	-0.82366100
H	17.97270100	14.53518300	-1.59237900
C	17.24521100	16.07281900	0.63931200

H	15.38309500	16.62859200	-0.41360100
H	15.17316000	15.43540000	0.88559900
H	18.97747100	14.70566900	0.63615800
H	17.51037600	14.06714100	1.40634300
H	17.78037800	16.76312500	-0.02736900
H	17.22395000	16.51462400	1.63960800
O	13.81473500	12.32777600	-1.51164600
C	13.60348600	11.18062400	-2.38492200
C	14.37379100	11.88764800	-0.26286100
C	13.98478400	9.97042600	-1.55257200
H	12.55187300	11.16783900	-2.70044900
H	14.26036000	11.31436000	-3.25318400
C	15.00498200	10.54969500	-0.57829800
H	15.07435300	12.66057600	0.08373500
H	13.56208100	11.78784200	0.47620700
H	14.38223000	9.15903100	-2.16874400
H	13.10978900	9.58749300	-1.00857700
H	15.96908000	10.70002200	-1.08266200
H	15.16286900	9.93422200	0.31195600
O	12.86120800	14.00857100	-3.66941600
C	11.47182500	13.75576700	-3.36984700
C	13.06701900	14.00213800	-5.09855600
C	10.85243800	13.32495500	-4.68198400
H	11.40319000	12.98653000	-2.59328800
H	11.02772800	14.68589800	-2.97845000
C	11.67629300	14.12231800	-5.68649500
H	13.75149900	14.82576000	-5.33609600
H	13.55494500	13.05997400	-5.38266900
H	9.77713400	13.52253200	-4.71814900
H	10.98994600	12.24348200	-4.82737400
H	11.35300200	15.17305100	-5.70109600
H	11.62099200	13.73450900	-6.70750100
C	11.06334600	15.47002800	-0.04650600
H	11.11132700	15.96991600	-1.02660600
H	10.05467700	15.03197200	0.00765000
C	11.29090400	16.47671100	1.06784500
H	11.30196500	15.94312000	2.03237700
H	12.29319200	16.92330100	0.95111700
C	10.23835300	17.57051800	1.11107300
H	10.20632800	18.09288600	0.14037500
H	9.24460200	17.10977800	1.23524800
C	10.47230000	18.57952800	2.22286700
H	11.46414600	19.04393700	2.09336000
H	10.51558700	18.05156400	3.18954300
C	9.41059000	19.66489000	2.28931500
H	8.41987200	19.20018300	2.42164800
H	9.36318600	20.19355700	1.32277400
C	9.64790600	20.67174000	3.40390300
H	10.63704600	21.13545200	3.26786400
H	9.69792400	20.13929200	4.36586700
C	8.57548000	21.74570800	3.46238100
H	7.58469800	21.30305500	3.62423300
H	8.75448300	22.46338800	4.26988900
H	8.53002700	22.30863600	2.52153700

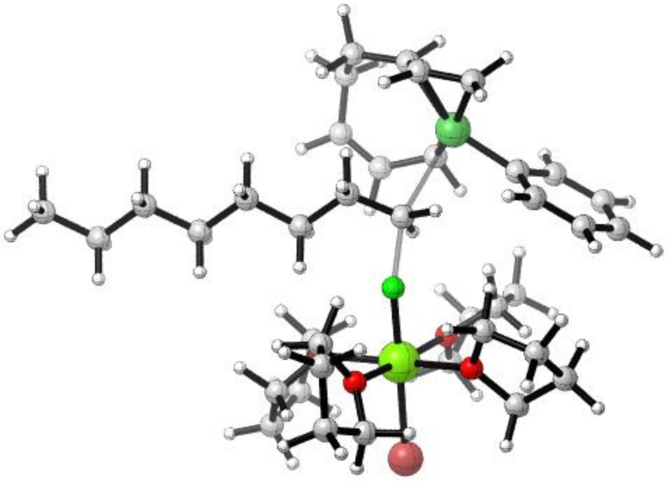

TS10-Ph-*n*-Oct

Ni	8.39532400	11.86274200	0.10011200
C	6.73306500	11.56678500	-0.93909300

C	6.43953900	11.56103900	0.43856800	C	14.04426800	11.51265000	0.80733900
C	6.88639400	12.61223000	1.25036000	H	14.51226600	9.64208500	1.90664500
C	6.68638600	12.65265800	2.73100600	H	15.07507800	9.68435100	0.22172100
C	7.81490700	13.36993500	3.49525700	C	13.29640000	11.61253300	-0.52330100
C	9.05830700	12.55112000	3.64164200	H	12.98616100	9.71242300	-1.57344400
C	9.89984200	12.26908700	2.63177900	H	11.54074200	10.28049100	-0.71168900
C	9.79263200	12.67115100	1.22340600	H	14.96606800	12.10087800	0.83479700
C	9.45561100	11.62895300	-1.47674100	H	13.40161200	11.83839100	1.63782500
C	10.27231000	12.68935100	-1.91424400	H	13.99135500	11.79275900	-1.35155100
C	11.03882500	12.62893200	-3.07796400	H	12.55589300	12.41792800	-0.53916200
C	11.02839500	11.48487600	-3.86976800	O	12.26232100	7.50794600	3.00368000
C	10.20824700	10.42514000	-3.49313200	C	12.57881300	6.37372400	3.81863200
C	9.43895500	10.51240200	-2.33401900	C	12.69301000	8.63840000	3.76360000
H	6.65479200	12.49716500	-1.50670700	C	13.92819100	6.69086100	4.45780800
H	6.52554500	10.67407600	-1.52640800	H	11.78237000	6.27079600	4.57577300
H	6.14765800	10.62327900	0.92245900	H	12.57856000	5.48932500	3.17529700
H	6.93416200	13.60928800	0.78799900	C	14.03729300	8.22463800	4.35002400
H	6.56297600	11.63189000	3.12990500	H	12.71411900	9.50585000	3.09217600
H	5.74086700	13.17479200	2.94801700	H	11.94966700	8.83326400	4.55450400
H	8.03202200	14.31095400	2.96282300	H	14.73199600	6.21580500	3.88657400
H	7.44291900	13.66160200	4.48558600	H	13.97227400	6.33626900	5.49224300
H	9.27310500	12.14248600	4.63130100	H	14.85490200	8.48555100	3.66876400
H	10.75439600	11.62678800	2.88509500	H	14.22554400	8.71338500	5.31072900
H	10.77277700	12.57474600	0.73478500	O	11.44097000	5.48432300	1.02578700
H	9.48698900	13.73058500	1.14754900	C	10.10785600	5.22079400	1.49221600
H	11.65753900	13.48028600	-3.36136800	C	12.01597500	4.30088200	0.43798800
H	10.14911900	9.53301100	-4.12002500	C	9.71152900	3.92078900	0.82049900
C	9.48108200	9.64827600	0.89137000	H	10.12905400	5.11808100	2.59049300
H	10.37570200	10.23509400	1.05760800	H	9.49420500	6.08908000	1.23633800
F	10.48983900	8.06324300	0.77416400	C	11.04507900	3.18429500	0.77224400
Mg	12.31979700	7.45611400	0.82869200	H	12.11039800	4.47673600	-0.64530200
Br	14.66692500	6.56892500	0.89645300	H	13.02508000	4.16798600	0.84329300
O	13.02380500	9.47685400	0.49119100	H	9.34419100	4.11326000	-0.19676500
C	14.28315400	10.02407500	0.90835400	H	8.92986800	3.38410700	1.36594300
C	12.63369900	10.23846400	-0.67460600	H	11.07513600	2.36947900	0.04334700

H	11.27850300	2.76358500	1.75950700
O	12.16032000	7.14288200	-1.26891300
C	10.88958400	7.01558000	-1.93296700
C	13.23245000	7.11817900	-2.22841700
C	11.23002300	6.66210600	-3.36814100
H	10.30480700	6.25374100	-1.40334800
H	10.36203000	7.97627500	-1.86246600
C	12.55511500	7.39168000	-3.55485900
H	13.97811600	7.86154200	-1.92432800
H	13.71229000	6.12901800	-2.19904200
H	10.45091500	6.97650600	-4.06973100
H	11.37346900	5.57823100	-3.47895900
H	12.37025200	8.46962000	-3.67699000
H	13.13966300	7.03891200	-4.40926300
H	10.32935600	13.60157600	-1.31584400
H	9.16896600	9.49993500	-0.13118400
C	8.58346700	9.30455300	2.01678500
H	7.83161500	8.57777600	1.67409200
H	8.03725100	10.22518800	2.27975800
C	9.26876200	8.81976700	3.28497100
H	10.00055500	9.58215500	3.59578200
H	9.83911000	7.90409000	3.08320800
C	8.27942900	8.58353300	4.41176300
H	7.74667800	9.52118800	4.64041600
H	7.50670400	7.86956300	4.08032200
C	8.94239800	8.05469400	5.67207300
H	9.51351200	7.14173600	5.42471200
H	9.68673200	8.78723900	6.02865900
C	7.96045300	7.74694000	6.79039500
H	7.38589900	8.65568900	7.03319200
H	7.21851600	7.01446300	6.43156800
C	8.62289600	7.21596300	8.05168800
H	9.20542000	6.31564800	7.80213400
H	9.35428100	7.95402800	8.41464900
C	7.62157000	6.89641200	9.14826500
H	8.10786600	6.51635300	10.05280300
H	7.04754100	7.78834100	9.42897900
H	6.90166900	6.13875900	8.81401100
H	8.78565800	9.66320900	-2.10580100
H	11.62837600	11.42901100	-4.77623100

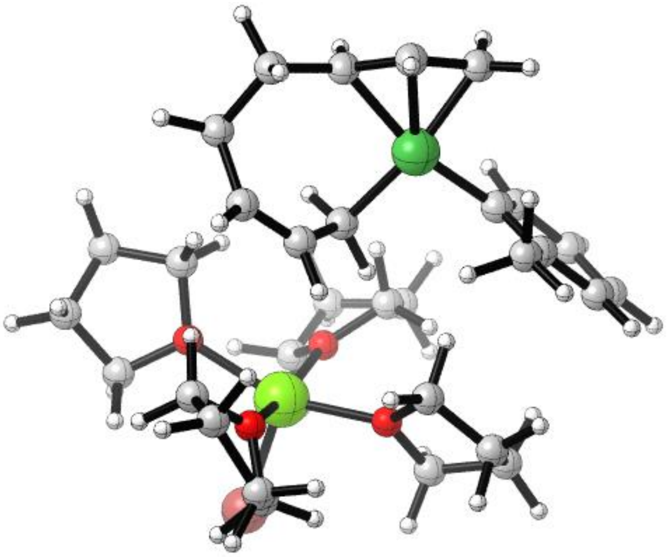

INT1-2-methylphenyl

Ni	9.44887300	12.74459800	-0.67412600
C	7.52519000	12.61352200	-0.32531600
C	8.27125400	12.26775200	0.82320700
C	9.25867300	13.13515900	1.31948800
C	10.13184300	12.74910400	2.47226400
C	11.63495400	13.03607300	2.26679800
C	12.24291100	12.08613700	1.28718000
C	12.04769500	12.16722200	-0.05086300
C	11.36014500	13.21160900	-0.79895900
C	9.16386100	12.67743900	-2.54947600
C	8.96194600	13.86337400	-3.28751800
C	8.85775400	13.85303600	-4.68107300
C	8.88799200	12.65341500	-5.38605200
C	9.01375400	11.46399900	-4.67417500

C	9.15910800	11.47225900	-3.28327800	H	16.95025200	13.88956100	-0.56801500
H	7.14719800	13.63330500	-0.43844900	H	16.76385300	15.63293800	-0.83252900
H	6.88573400	11.85706800	-0.77646400	C	14.90912000	15.81309400	1.23426200
H	8.30904100	11.22054700	1.14359100	H	13.34811800	15.72302100	-0.32607500
H	9.07996400	14.21668700	1.23204000	H	13.51603700	14.23397300	0.65561700
H	10.01532100	11.67397800	2.68044100	H	17.06670100	15.54687500	1.60877200
H	9.80302000	13.27744100	3.38126400	H	16.09041400	14.06479200	1.72227300
H	11.73553900	14.08602700	1.94006300	H	15.09050300	16.85083000	0.92426700
H	12.14822500	12.96650000	3.23621600	H	14.43796500	15.82906800	2.22135800
H	12.68798200	11.17718700	1.69844600	O	15.09810400	11.76025400	-1.69379000
H	12.40741900	11.31758700	-0.64775600	C	15.85049900	10.81944900	-2.47853900
H	11.70370000	13.26419100	-1.84264800	C	15.35930800	11.39941300	-0.33115200
H	11.44026000	14.20460400	-0.32944500	C	15.63719800	9.48331200	-1.77419700
H	8.72282000	14.79671700	-5.21516100	H	15.48312900	10.87388300	-3.50817900
H	8.78228900	12.64270500	-6.46976400	H	16.90498300	11.13318800	-2.47426700
H	8.99380900	10.50919700	-5.20548900	C	15.25940100	9.88007000	-0.33137800
Mg	14.55329100	13.60263900	-2.54078900	H	16.37628800	11.73635300	-0.06762600
Br	16.62061900	14.06090400	-3.76424200	H	14.61634800	11.90190000	0.29849700
O	13.43891800	15.27677400	-3.07213000	H	16.53863700	8.86516100	-1.82018500
C	14.10299700	16.54437700	-3.27631200	H	14.82822100	8.91570700	-2.24660600
C	12.04564800	15.37661300	-3.45875300	H	15.91535500	9.43150400	0.42015600
C	12.97808100	17.53565100	-3.48650800	H	14.23353500	9.57131400	-0.10135500
H	14.73938300	16.73413100	-2.40295300	O	13.44391300	12.52256100	-3.90598600
H	14.75092500	16.45990100	-4.15961300	C	12.51717900	11.42421700	-3.69125100
C	11.95696300	16.67723800	-4.22501900	C	13.60178500	12.76507700	-5.32532100
H	11.77314100	14.48145500	-4.02864000	C	12.37113300	10.77532600	-5.04829500
H	11.43529100	15.39479100	-2.54705700	H	12.93897500	10.77936500	-2.91044600
H	13.30091000	18.41900300	-4.04452400	H	11.56045900	11.83914100	-3.34319900
H	12.56799200	17.86739700	-2.52298300	C	12.47871800	11.98344200	-5.97274000
H	12.26611600	16.53347000	-5.26927300	H	13.57445300	13.85050700	-5.48438600
H	10.94407600	17.08962300	-4.22194700	H	14.59321100	12.40197300	-5.63184800
O	14.99780600	14.54351400	-0.74795900	H	11.41415200	10.25188500	-5.13905900
C	16.35133700	14.76544800	-0.29741000	H	13.18711300	10.06337500	-5.23767800
C	14.05647500	15.08293500	0.21501700	H	11.53367500	12.54616900	-5.94428300
C	16.20816400	15.01702400	1.18696200	H	12.70103500	11.72516000	-7.01203400

C	9.32689200	10.16927500	-2.55158700
H	10.27964600	10.13709900	-2.00036600
H	8.54192000	10.03489000	-1.79511600
H	9.29552600	9.30406200	-3.22742700
H	8.83640017	14.78764312	-2.76324246

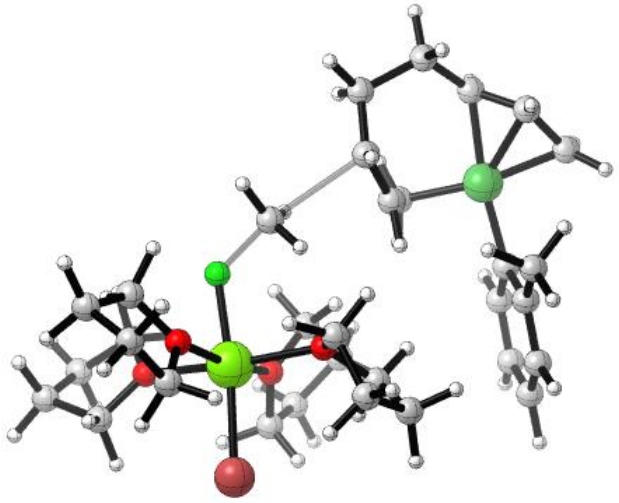

TS3-2-methylphenyl-Me

Ni	8.54804400	11.24685200	-0.35723600
C	7.25876600	9.87839800	0.18665900
C	7.67573200	10.63567900	1.30360100
C	7.63367500	12.04200600	1.28604300
C	8.33454900	12.83164600	2.35682400
C	9.49446600	13.75293500	1.89169000
C	10.56793000	13.01309700	1.16127900
C	10.31277800	12.54669500	-0.12573600
C	9.26549300	12.96434500	-0.99076400
C	9.23175000	10.29985500	-1.86834500
C	8.90973600	10.64091900	-3.19211800
C	9.49528500	10.03599000	-4.30470700
C	10.45074800	9.04217200	-4.12576900
C	10.79110300	8.67080600	-2.82808900
C	10.20751500	9.28589600	-1.71583700
H	6.36242300	10.17133300	-0.36679100
H	7.45707300	8.80862200	0.16969800
H	8.34695700	10.16620100	2.03173800
H	6.78335400	12.53348200	0.80024700
H	8.76264700	12.12834500	3.08864300
H	7.60822400	13.44511100	2.91149900
H	9.08145700	14.55178400	1.25293700
H	9.89813600	14.25678600	2.78173500
H	11.28425800	12.45020500	1.76931900
H	11.07963900	11.90334300	-0.57578300
H	9.42344400	12.90475900	-2.06734100
H	8.65019000	13.80964900	-0.67337300
H	9.19564600	10.33504600	-5.30984300
H	11.52587100	7.87740300	-2.67222900
C	12.20626400	14.49567600	-0.07426600
H	12.39404200	15.03409800	0.84895400
H	12.61056600	13.50275100	-0.16098900
H	11.27572200	14.70535300	-0.58630700
F	13.24603600	15.24410000	-1.13163000
Mg	14.45815000	14.24424600	-2.40842300
Br	16.16657500	13.16771900	-3.86146100
O	14.85134600	16.20364500	-3.17592200
C	16.10213800	16.65669800	-3.72534700
C	13.84036500	17.21477200	-3.32784400
C	15.92970500	18.15395000	-3.87440200
H	16.90307600	16.35290900	-3.04139900
H	16.26939300	16.15397400	-4.68796500
C	14.45535600	18.25007500	-4.25004600
H	12.93487800	16.73525700	-3.71891000
H	13.61017100	17.63031100	-2.33685500
H	16.60600200	18.58465700	-4.61788700
H	16.10880800	18.65703400	-2.91380900
H	14.31449300	17.96213100	-5.30051600
H	14.02556300	19.24590700	-4.11033700
O	15.89094400	14.60341500	-0.84604800

C	17.21137300	14.03321300	-0.74248900
C	15.81650300	15.81095400	-0.05847900
C	17.73755100	14.56854900	0.56937900
H	17.11452100	12.94509400	-0.79775200
H	17.81499000	14.35760500	-1.60125200
C	17.20277100	15.99529100	0.53837000
H	15.50454400	16.63444800	-0.71265400
H	15.04334900	15.66803200	0.70628000
H	18.82670200	14.50864400	0.64525400
H	17.30131600	14.01195500	1.40951400
H	17.82713700	16.61340700	-0.12057900
H	17.17216700	16.47791600	1.51931800
O	13.68149700	12.34242600	-1.71373400
C	13.18664400	11.32915000	-2.63111900
C	14.32249500	11.65218700	-0.63290000
C	13.99011900	10.05986200	-2.33882700
H	12.11086400	11.18066600	-2.44816600
H	13.33431100	11.71773000	-3.64308600
C	15.03250600	10.50126600	-1.30955200
H	14.96730600	12.37416800	-0.11768600
H	13.55125500	11.29896500	0.07484900
H	14.44968600	9.65458200	-3.24484100
H	13.32583200	9.29242400	-1.92380600
H	15.93231900	10.87349800	-1.81550000
H	15.31286200	9.70578200	-0.61282500
O	12.87483000	14.21892800	-3.80002600
C	11.48005200	14.12131900	-3.44767700
C	13.03687400	14.19747400	-5.23186900
C	10.78706500	13.69467600	-4.72314100
H	11.37427000	13.41145100	-2.62236000
H	11.13830900	15.10756000	-3.09377100
C	11.63650100	14.39678300	-5.77945600
H	13.76382900	14.97626900	-5.49503100
H	13.46126800	13.22844700	-5.52898200
H	9.73107100	13.97998000	-4.73729000
H	10.83772100	12.60159100	-4.83166500
H	11.38574400	15.46621400	-5.81887800
H	11.52033600	13.98314000	-6.78507300
C	10.65304500	8.86827500	-0.34066700
H	11.15173400	9.69260700	0.19398300
H	9.80228500	8.57533600	0.28732900
H	11.35267900	8.02291900	-0.37439700
H	10.91303600	8.54951900	-4.97931800
H	8.16041300	11.41785000	-3.36654100

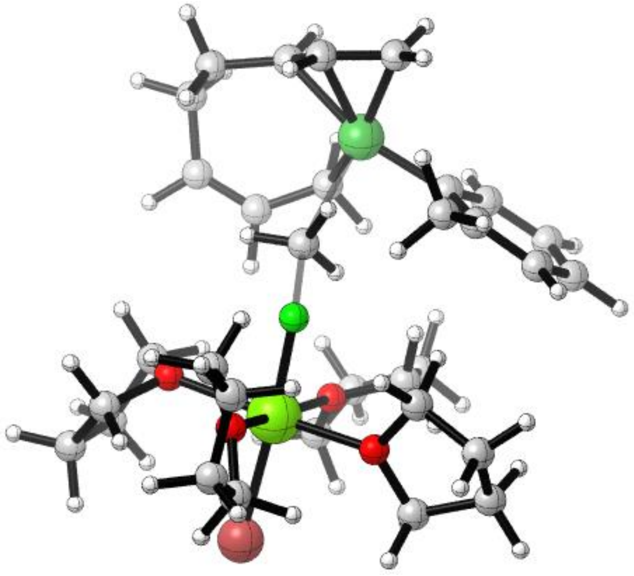

TS10-2-methylphenyl -Me

Ni	8.27209500	11.55862600	0.21797800
C	6.48104800	11.53335700	-0.64580600
C	6.39516600	11.09330000	0.68837700
C	6.93997600	11.89712000	1.70269400
C	6.99027700	11.48385300	3.13992500
C	8.05238100	12.23840900	3.95688300
C	9.44832000	11.72837900	3.77092500
C	10.13697300	11.76701600	2.61873800
C	9.73720000	12.30656000	1.30834300
C	9.22481400	11.52319400	-1.46787500

C	9.97049100	12.66651000	-1.81167700	H	14.00845100	11.80976200	-1.30154400
C	10.76956500	12.76365400	-2.94950700	H	12.43554100	12.33477900	-0.69676300
C	10.87751400	11.67732500	-3.80813200	O	11.55112900	7.49481100	2.87881800
C	10.12602200	10.54064100	-3.53152600	C	11.69720900	6.34499700	3.72607800
C	9.29308200	10.45782100	-2.40671800	C	11.86293800	8.62262000	3.70061800
H	6.34493000	12.59471000	-0.86864600	C	12.82955000	6.67321700	4.70335700
H	6.17340200	10.87539400	-1.45424100	H	10.74152100	6.18805100	4.24942600
H	6.20544300	10.03436100	0.89909400	H	11.89583700	5.48380400	3.07956000
H	6.84459500	12.98375700	1.56582400	C	13.07041300	8.18021000	4.50801300
H	7.15987400	10.39792200	3.22452000	H	12.03084900	9.48079700	3.03996500
H	6.00857700	11.67554300	3.60297700	H	10.99505500	8.85340100	4.33942100
H	7.98760100	13.30533300	3.68274200	H	13.73183600	6.10525600	4.46017500
H	7.78542000	12.18874100	5.02014400	H	12.53815200	6.43306300	5.73109900
H	9.92638000	11.27504000	4.64175800	H	13.98563300	8.32997800	3.92300800
H	11.13165800	11.30383100	2.64757000	H	13.16310200	8.72960100	5.44940800
H	10.63094900	12.42987200	0.68096400	O	11.30024400	5.40241600	0.76737400
H	9.27864600	13.30526700	1.43141700	C	9.90438700	5.07881900	0.88494800
H	11.31627500	13.68378000	-3.15442900	C	12.03991200	4.25403000	0.30278600
H	10.16226300	9.69467600	-4.22394100	C	9.71848300	3.86445700	0.00029700
C	9.59246800	9.55252900	0.75655000	H	9.67946400	4.84325200	1.93807400
H	10.43610200	10.10936900	0.38745000	H	9.33665200	5.96489000	0.59210200
F	10.30644000	7.96918100	0.41147000	C	11.02904000	3.12166200	0.23234100
Mg	12.13108200	7.40786800	0.76549400	H	12.46064300	4.50952300	-0.68129000
Br	14.42750700	6.62943500	1.35950600	H	12.87418600	4.07109200	0.98899900
O	12.80722600	9.47114000	0.44442300	H	9.63002000	4.16889400	-1.05147900
C	13.96151400	10.05010000	1.07653700	H	8.83015400	3.28176900	0.26073800
C	12.67288500	10.16908900	-0.81367900	H	11.27025300	2.39666900	-0.55015900
C	13.77020100	11.53352800	0.85414700	H	10.99100700	2.58313800	1.18812600
H	13.98014900	9.72259300	2.11955200	O	12.43873900	7.09656300	-1.32632000
H	14.87124800	9.66973400	0.58532900	C	11.35590500	7.04637500	-2.27356700
C	13.22019600	11.58146900	-0.57605300	C	13.70494000	7.05221400	-2.01681700
H	13.25665600	9.63321200	-1.57642300	C	11.99449800	6.60874300	-3.57363400
H	11.61901900	10.15366000	-1.11471700	H	10.59153800	6.36127900	-1.88660700
H	14.69704700	12.10061600	0.98048800	H	10.91415800	8.04947200	-2.35870100
H	13.03145000	11.92630600	1.56525400	C	13.35646400	7.28445500	-3.47413400

H	14.36657300	7.80515300	-1.57434700
H	14.16296300	6.06726300	-1.84914300
H	11.41128800	6.91479100	-4.44747200
H	12.11058000	5.51629700	-3.60066800
H	13.25283500	8.35938900	-3.68043900
H	14.10752800	6.87514700	-4.15548500
H	11.50667700	11.72196600	-4.69520900
C	8.48691900	9.18880400	-2.26355000
H	8.95670200	8.45671900	-1.59091700
H	7.48631500	9.38727600	-1.86531300
H	8.36278400	8.69070300	-3.23369000
H	9.93660800	13.53482700	-1.14943200
H	8.73426000	9.39459800	0.12232400
H	9.47921400	9.46906200	1.83354400

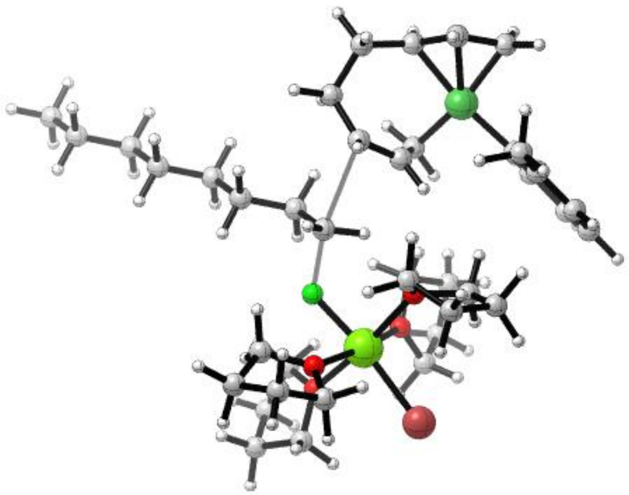

TS3-2-methylphenyl-*n*-Oct

Ni	8.54333400	11.13320100	-0.35658300
C	7.34329600	9.69245900	0.20602000
C	7.71802300	10.48336900	1.31361000
C	7.59244600	11.88534600	1.28349500
C	8.25139300	12.72067200	2.34644200
C	9.32974500	13.72776800	1.86378500
C	10.44413700	13.07354400	1.11491600
C	10.21467100	12.57239600	-0.16207900
C	9.13049300	12.88573700	-1.02651900
C	9.28871000	10.19579200	-1.84285200
C	8.95950600	10.48379400	-3.17735900
C	9.57069700	9.86454700	-4.26811000
C	10.55650900	8.90819000	-4.05512000
C	10.90812100	8.59312200	-2.74556900
C	10.30443900	9.22780900	-1.65572700
H	6.43043700	9.92601300	-0.34861100
H	7.60654800	8.63670000	0.19702000
H	8.41743100	10.06046800	2.04381300
H	6.70813300	12.32080200	0.80507400
H	8.74248200	12.04857300	3.06763100
H	7.49247500	13.27831400	2.91626200
H	8.83463200	14.49232300	1.23858300
H	9.70962100	14.26045500	2.74830000
H	11.22344700	12.59272500	1.71461300
H	11.02018200	11.97000700	-0.60133600
H	9.29717700	12.83106100	-2.10328800
H	8.44493400	13.67930900	-0.71907800
H	9.26635400	10.12196300	-5.28319200
H	11.67027200	7.83195400	-2.56269900
C	12.02217800	14.65263000	-0.25411400
H	11.88642600	13.80423400	-0.90133600
F	13.33752200	15.29103900	-1.20327600
Mg	14.40529100	14.24206900	-2.50190400
Br	16.03736100	13.04168900	-3.95042800
O	14.84136600	16.16190300	-3.29275200
C	16.12931900	16.58421400	-3.77324900
C	13.89986200	17.24503900	-3.33643100
C	16.04374900	18.09704200	-3.81237900
H	16.88711700	16.17590900	-3.09410700
H	16.29412900	16.15016200	-4.76941900
C	14.57772100	18.30809300	-4.17853900

H	12.96028300	16.85953000	-3.75277600	C	10.75238500	13.68219300	-4.91984400
H	13.70461700	17.58741900	-2.30967000	H	11.25943100	13.34786200	-2.81470200
H	16.74479000	18.54017900	-4.52509100	H	10.99819400	15.05573700	-3.24195100
H	16.25036600	18.52179900	-2.82021900	C	11.61842100	14.43784400	-5.92239600
H	14.42350500	18.10408400	-5.24673100	H	13.72162700	15.04698100	-5.55201000
H	14.20828700	19.31528700	-3.96656300	H	13.45202800	13.29496300	-5.63024900
O	15.93297500	14.55625200	-0.98988400	H	9.68899800	13.93401100	-4.96515100
C	17.18700600	13.86199100	-0.82841000	H	10.84815400	12.59552800	-5.05651200
C	15.86561900	15.67799800	-0.08901800	H	11.34313900	15.50193400	-5.93604500
C	17.72444600	14.35376700	0.49876100	H	11.54814800	14.05447200	-6.94414300
H	16.99067900	12.78568100	-0.86987400	C	10.77458800	8.88580000	-0.26767900
H	17.84472100	14.11497900	-1.67106500	H	11.20732500	9.76295500	0.24045200
C	17.26006100	15.80413900	0.49214300	H	9.94877300	8.54619300	0.37027900
H	15.53506900	16.55697600	-0.65358000	H	11.53714400	8.09590600	-0.27484000
H	15.10901500	15.46482400	0.67997000	H	11.03691300	8.40361400	-4.89141300
H	18.80798800	14.23484100	0.58516400	H	8.18159600	11.22527000	-3.37747200
H	17.25184900	13.80837800	1.32717700	C	11.06833100	15.78919700	-0.30005300
H	17.89879300	16.40161100	-0.17304300	H	10.04150000	15.39300300	-0.28138700
H	17.26266300	16.27700000	1.47836500	H	11.17943400	16.27828200	-1.28092400
O	13.51251500	12.43358400	-1.76116100	H	12.59460300	14.47218500	0.65188400
C	13.11952700	11.34364600	-2.63688500	C	11.29537700	16.79712100	0.81192400
C	14.04104400	11.83419300	-0.57333400	H	11.21574200	16.27774100	1.78070200
C	13.99873200	10.15190400	-2.25182600	H	12.33089000	17.17311800	0.74310800
H	12.05170200	11.12675500	-2.47242000	C	10.31703500	17.95784300	0.77345100
H	13.26671800	11.69118800	-3.66381800	H	10.38504500	18.46472400	-0.20366400
C	14.85615500	10.66638000	-1.08963300	H	9.28908200	17.56434400	0.83518200
H	14.61236100	12.60525000	-0.04031800	C	10.53993900	18.96944800	1.88476000
H	13.20260200	11.50539500	0.06559400	H	11.57343600	19.35045100	1.83203200
H	14.61718000	9.81971300	-3.09048700	H	10.45892800	18.46273100	2.86010900
H	13.36510400	9.31374800	-1.94207500	C	9.56891300	20.13815500	1.83990600
H	15.81885200	11.03140900	-1.47096100	H	8.53550600	19.75780300	1.88851000
H	15.04195200	9.90911200	-0.32206000	H	9.65196300	20.64688500	0.86508600
O	12.78945500	14.23816600	-3.90186100	C	9.78766000	21.14835700	2.95488100
C	11.38652500	14.09085700	-3.60816100	H	10.82238700	21.52099500	2.90759900
C	13.00060400	14.25031900	-5.32980100	H	9.69771500	20.63926200	3.92635800

C	8.81456600	22.31293900	2.88863000
H	7.77733400	21.96297100	2.96247600
H	8.97950000	23.03497600	3.69522900
H	8.90906400	22.85056200	1.93668600

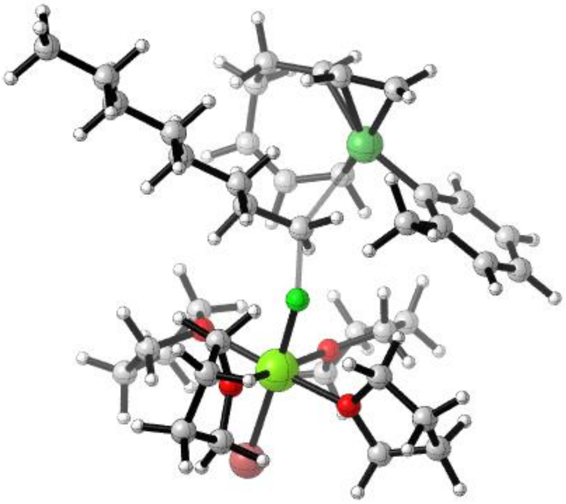

TS10-2-methylphenyl -*n*-Oct

Ni	8.21710100	11.57494400	0.30510900
C	6.48030300	11.65937000	-0.64976600
C	6.27086900	11.37677900	0.71426400
C	6.86315200	12.20115300	1.68097400
C	6.77194200	11.94427800	3.15068900
C	7.89564700	12.61696000	3.95613500
C	9.21107200	11.90305600	3.89730700
C	9.96768800	11.75992900	2.79517500
C	9.71563300	12.22343000	1.42473200
C	9.18816300	11.45871300	-1.36928300
C	9.95502500	12.59145000	-1.70229300
C	10.74036500	12.68850600	-2.84922700
C	10.80708600	11.61500500	-3.72807800
C	10.03525500	10.49074100	-3.45846400
C	9.21888300	10.40537100	-2.32221100
H	6.47543200	12.69775600	-0.99056200
H	6.13710600	10.95125400	-1.39997300
H	5.91873400	10.38584500	1.01998200
H	6.93421400	13.27040200	1.43368700
H	6.76328200	10.85934400	3.35352200
H	5.80918100	12.32966600	3.52363400
H	7.99822200	13.64954500	3.58104400
H	7.57833300	12.70955800	5.00238100
H	9.57246900	11.46160300	4.82790200
H	10.88301300	11.17022100	2.93320100
H	10.64924900	12.19190000	0.84598000
H	9.36328500	13.27153400	1.43311600
H	11.30716300	13.59807700	-3.04655500
H	10.04198600	9.65638000	-4.16542100
C	9.48799900	9.39618100	0.94581800
H	10.42209100	9.91393900	1.11359100
F	10.43751300	7.87909300	0.25091000
Mg	12.22914900	7.32195000	0.64959700
Br	14.53036200	6.51045600	1.23042900
O	12.88986600	9.37925600	0.45539300
C	14.07898200	9.97073900	1.00002100
C	12.50864900	10.18093300	-0.68903600
C	13.79501200	11.45167400	0.90186900
H	14.22429300	9.58007700	2.01140500
H	14.95029500	9.67379800	0.39442700
C	13.15808800	11.55258300	-0.48383700
H	12.86742300	9.68669100	-1.60238200
H	11.41479900	10.21630900	-0.72955300
H	14.69035800	12.06955600	1.01573600
H	13.07412600	11.74333300	1.67939100
H	13.92082500	11.73296200	-1.25041100
H	12.41938600	12.35633400	-0.55977300
O	11.70760000	7.44595200	2.77289400
C	11.79100700	6.30323500	3.63428500
C	12.05981000	8.57039700	3.58264700
C	12.93715800	6.58846000	4.60849300

H	10.82381300	6.20223400	4.15483400
H	11.94973400	5.42694400	2.99723000
C	13.23701200	8.08542900	4.41059700
H	12.27164000	9.40965200	2.90922400
H	11.19441300	8.84482900	4.21130200
H	13.81588200	5.98725900	4.35809400
H	12.64309600	6.35733100	5.63742800
H	14.16575200	8.19863100	3.83907500
H	13.33536400	8.63509700	5.35126800
O	11.40161900	5.32132200	0.67178500
C	9.99072400	5.03926800	0.70411300
C	12.14336300	4.14061800	0.31244500
C	9.84957800	3.63970700	0.13462700
H	9.65359600	5.09309400	1.75296700
H	9.47577100	5.82219000	0.13843800
C	11.17293800	3.00022100	0.53688400
H	12.44873000	4.22872300	-0.74332500
H	13.05036800	4.11654600	0.92517700
H	9.76793600	3.68033300	-0.95999300
H	8.96846400	3.11754100	0.51871000
H	11.42746000	2.10760000	-0.04161400
H	11.15616400	2.72474000	1.60041600
O	12.51632600	7.07301000	-1.44429900
C	11.41530500	7.06083000	-2.37025800
C	13.76921700	7.13828200	-2.15193600
C	12.04146600	6.75003300	-3.71302700
H	10.68607900	6.31449500	-2.03168500
H	10.93310500	8.04818600	-2.35931700
C	13.38914300	7.45055700	-3.58774000
H	14.39692700	7.89845100	-1.67261600
H	14.27613500	6.16846100	-2.05083400
H	11.43394400	7.11156300	-4.54829900
H	12.18389300	5.66737700	-3.83297900
H	13.26164000	8.53364800	-3.72775200
H	14.13786400	7.10135400	-4.30449000
H	11.42302200	11.65998900	-4.62446200
C	8.39454900	9.14658400	-2.18049500
H	8.88287500	8.38408500	-1.55324600
H	7.41794300	9.35068900	-1.72798800
H	8.21788300	8.68273100	-3.15899400
H	9.95208700	13.44865900	-1.02443900
H	9.02204000	9.51487400	-0.01728000
C	8.75999900	8.77758400	2.06449300
H	8.47199600	9.58599300	2.76435400
H	9.47114700	8.15568800	2.63038100
C	7.56059700	7.95441800	1.62874600
H	7.90764900	7.13328100	0.98270600
H	6.90200900	8.57068800	0.99718300
C	6.77241200	7.40565100	2.80381400
H	7.43490700	6.78968900	3.43660400
H	6.44835300	8.24511900	3.44242700
C	5.55908400	6.59035700	2.38863900
H	4.91220400	7.20753200	1.74348900
H	5.88159700	5.74223400	1.76157200
C	4.74978300	6.07310000	3.56595400
H	5.39307900	5.45145600	4.21078100
H	4.43568400	6.92371300	4.19289800
C	3.52428700	5.27232000	3.15336500
H	2.88856800	5.89498900	2.50584200
H	3.83976300	4.42052000	2.53155100
C	2.72213500	4.77613700	4.34375500
H	1.84080900	4.20304500	4.03711200
H	3.33205100	4.12973700	4.98749300
H	2.37565300	5.61496300	4.96032300

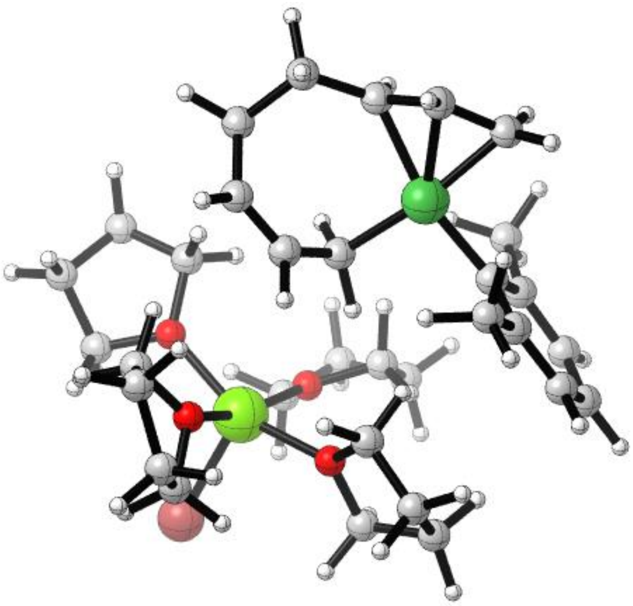

INT1-2,6-dimethylphenyl

Ni	9.44887300	12.74459800	-0.67412600
C	7.52519000	12.61352200	-0.32531600
C	8.27125400	12.26775200	0.82320700
C	9.25867300	13.13515900	1.31948800
C	10.13184300	12.74910400	2.47226400
C	11.63495400	13.03607300	2.26679800
C	12.24291100	12.08613700	1.28718000
C	12.04769500	12.16722200	-0.05086300
C	11.36014500	13.21160900	-0.79895900
C	9.16386100	12.67743900	-2.54947600
C	8.96194600	13.86337400	-3.28751800
C	8.85775400	13.85303600	-4.68107300
C	8.88799200	12.65341500	-5.38605200
C	9.01375400	11.46399900	-4.67417500
C	9.15910800	11.47225900	-3.28327800
H	7.14719800	13.63330500	-0.43844900
H	6.88573400	11.85706800	-0.77646400
H	8.30904100	11.22054700	1.14359100
H	9.07996400	14.21668700	1.23204000
H	10.01532100	11.67397800	2.68044100
H	9.80302000	13.27744100	3.38126400
H	11.73553900	14.08602700	1.94006300
H	12.14822500	12.96650000	3.23621600
H	12.68798200	11.17718700	1.69844600
H	12.40741900	11.31758700	-0.64775600
H	11.70370000	13.26419100	-1.84264800
H	11.44026000	14.20460400	-0.32944500
H	8.72282000	14.79671700	-5.21516100
H	8.78228900	12.64270500	-6.46976400
H	8.99380900	10.50919700	-5.20548900
Mg	14.55329100	13.60263900	-2.54078900
Br	16.62061900	14.06090400	-3.76424200
O	13.43891800	15.27677400	-3.07213000
C	14.10299700	16.54437700	-3.27631200
C	12.04564800	15.37661300	-3.45875300
C	12.97808100	17.53565100	-3.48650800
H	14.73938300	16.73413100	-2.40295300
H	14.75092500	16.45990100	-4.15961300
C	11.95696300	16.67723800	-4.22501900
H	11.77314100	14.48145500	-4.02864000
H	11.43529100	15.39479100	-2.54705700
H	13.30091000	18.41900300	-4.04452400
H	12.56799200	17.86739700	-2.52298300
H	12.26611600	16.53347000	-5.26927300
H	10.94407600	17.08962300	-4.22194700
O	14.99780600	14.54351400	-0.74795900
C	16.35133700	14.76544800	-0.29741000
C	14.05647500	15.08293500	0.21501700
C	16.20816400	15.01702400	1.18696200
H	16.95025200	13.88956100	-0.56801500
H	16.76385300	15.63293800	-0.83252900
C	14.90912000	15.81309400	1.23426200
H	13.34811800	15.72302100	-0.32607500
H	13.51603700	14.23397300	0.65561700
H	17.06670100	15.54687500	1.60877200
H	16.09041400	14.06479200	1.72227300

H	15.09050300	16.85083000	0.92426700
H	14.43796500	15.82906800	2.22135800
O	15.09810400	11.76025400	-1.69379000
C	15.85049900	10.81944900	-2.47853900
C	15.35930800	11.39941300	-0.33115200
C	15.63719800	9.48331200	-1.77419700
H	15.48312900	10.87388300	-3.50817900
H	16.90498300	11.13318800	-2.47426700
C	15.25940100	9.88007000	-0.33137800
H	16.37628800	11.73635300	-0.06762600
H	14.61634800	11.90190000	0.29849700
H	16.53863700	8.86516100	-1.82018500
H	14.82822100	8.91570700	-2.24660600
H	15.91535500	9.43150400	0.42015600
H	14.23353500	9.57131400	-0.10135500
O	13.44391300	12.52256100	-3.90598600
C	12.51717900	11.42421700	-3.69125100
C	13.60178500	12.76507700	-5.32532100
C	12.37113300	10.77532600	-5.04829500
H	12.93897500	10.77936500	-2.91044600
H	11.56045900	11.83914100	-3.34319900
C	12.47871800	11.98344200	-5.97274000
H	13.57445300	13.85050700	-5.48438600
H	14.59321100	12.40197300	-5.63184800
H	11.41415200	10.25188500	-5.13905900
H	13.18711300	10.06337500	-5.23767800
H	11.53367500	12.54616900	-5.94428300
H	12.70103500	11.72516000	-7.01203400
C	8.78552900	15.16215700	-2.55080600
H	7.79253800	15.20970000	-2.08207300
H	9.50838800	15.25944500	-1.72841100
H	8.86848700	16.03769600	-3.21024800
C	9.32689200	10.16927500	-2.55158700
H	10.27964600	10.13709900	-2.00036600
H	8.54192000	10.03489000	-1.79511600
H	9.29552600	9.30406200	-3.22742700

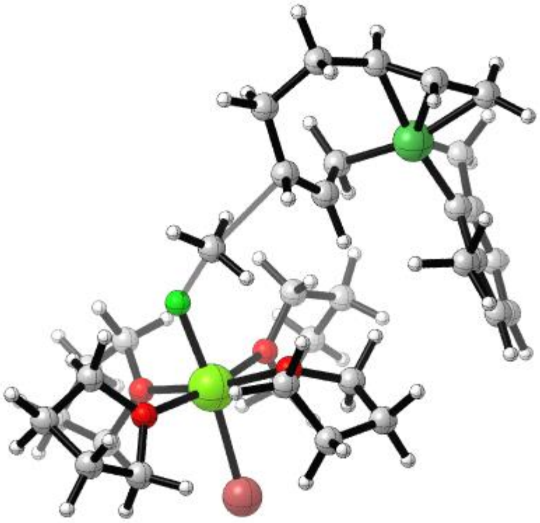

TS3-2,6-dimethylphenyl-Me

Ni	8.45762300	11.31014100	-0.38612700
C	7.18128400	9.93431600	0.15738300
C	7.65498400	10.64310300	1.28602100
C	7.62113500	12.04757200	1.33345800
C	8.36068600	12.78785800	2.41237500
C	9.51561600	13.71580300	1.94780300
C	10.55971000	12.98769800	1.16643700
C	10.26996100	12.57224600	-0.12968900
C	9.22138500	13.03195000	-0.96958400
C	9.04998300	10.42729600	-1.97166000
C	8.53930000	10.72122900	-3.25423500
C	9.13565000	10.21061900	-4.41018200
C	10.24277700	9.37223700	-4.33288900
C	10.72635900	9.02144400	-3.07880300
C	10.14317600	9.53485800	-1.91365400
H	6.25219000	10.24668500	-0.32834100
H	7.37676900	8.86589000	0.09042100
H	8.35349400	10.13973400	1.96398700

H	6.76008800	12.56476200	0.89482500	H	17.23426500	13.02005800	-0.89229100
H	8.80293200	12.05383900	3.10441100	H	17.92866100	14.46632100	-1.63774800
H	7.65725900	13.38647200	3.01085100	C	17.20428100	15.99928800	0.58592200
H	9.09364800	14.53852000	1.34679000	H	15.40540100	16.61348300	-0.53499900
H	9.94745900	14.18602100	2.84304400	H	15.10013700	15.45003700	0.77512600
H	11.27432800	12.38628400	1.73933800	H	18.89953200	14.58810000	0.61675700
H	11.01113200	11.92515800	-0.61474800	H	17.39907300	13.98262400	1.34920900
H	9.39503200	13.02078200	-2.04580700	H	17.77793900	16.67815200	-0.05971900
H	8.62253700	13.87826100	-0.62312300	H	17.16798000	16.43560000	1.58810200
H	8.71520900	10.46711400	-5.38492200	O	13.80847600	12.33391700	-1.62548600
H	11.56599600	8.32737200	-2.99584800	C	13.36068800	11.27309500	-2.51412200
C	12.22210800	14.45390000	-0.05108900	C	14.49033700	11.72464700	-0.52094500
H	12.43894400	14.96436500	0.88173100	C	14.12445000	10.01842100	-2.09256300
H	12.59383300	13.45168800	-0.16717300	H	12.27239100	11.15176400	-2.40584700
H	11.29788000	14.71463300	-0.55046600	H	13.59051400	11.59348500	-3.53543300
F	13.28531400	15.18878200	-1.10389900	C	15.20162000	10.54491200	-1.14472100
Mg	14.49100200	14.22347800	-2.40301700	H	15.13404000	12.49103200	-0.06861900
Br	16.20908600	13.15764800	-3.86744100	H	13.74532700	11.40040900	0.22730400
O	14.80149100	16.17336700	-3.19802800	H	14.54419700	9.49121500	-2.95393900
C	16.05381800	16.66728600	-3.70505300	H	13.45301900	9.32819400	-1.56579700
C	13.76852700	17.15880100	-3.35588100	H	16.06918800	10.89993700	-1.71571800
C	15.83395100	18.15666400	-3.87636900	H	15.52959600	9.80437900	-0.40936200
H	16.83555400	16.39512700	-2.98606800	O	12.90175900	14.09669700	-3.78157400
H	16.27258800	16.16158200	-4.65579200	C	11.51133600	13.90748500	-3.44832300
C	14.36465900	18.19981100	-4.28281600	C	13.08141000	14.05956600	-5.21373000
H	12.87488200	16.65260200	-3.74098500	C	10.87850900	13.38425800	-4.71827800
H	13.52812500	17.58092900	-2.36997000	H	11.44111900	13.22052000	-2.60003500
H	16.51064600	18.60102900	-4.61137200	H	11.08527400	14.87759400	-3.14482600
H	15.97587500	18.67729700	-2.91908600	C	11.67661800	14.13694200	-5.77900200
H	14.25297700	17.89136300	-5.33096500	H	13.75145600	14.88610300	-5.48282200
H	13.90124600	19.18343300	-4.16550000	H	13.58401200	13.12079900	-5.48372800
O	15.98832700	14.66943300	-0.92222100	H	9.79858700	13.56084700	-4.75110100
C	17.31306200	14.10776400	-0.80213600	H	11.03549100	12.29890400	-4.79993300
C	15.82342100	15.75789200	0.00712100	H	11.33956900	15.18152200	-5.83966100
C	17.80896600	14.60200700	0.53946000	H	11.60608600	13.69815100	-6.77812500

C	10.69791400	9.09681700	-0.58324800
H	11.23497900	9.90335100	-0.05822600
H	9.89493300	8.78110300	0.09476900
H	11.39356100	8.25364300	-0.69175000
C	7.30844100	11.57352400	-3.38944100
H	6.45193300	11.09887600	-2.89253400
H	7.43478400	12.55228700	-2.90742100
H	7.03466100	11.73937300	-4.43939000
H	10.69728000	8.96877400	-5.23613500

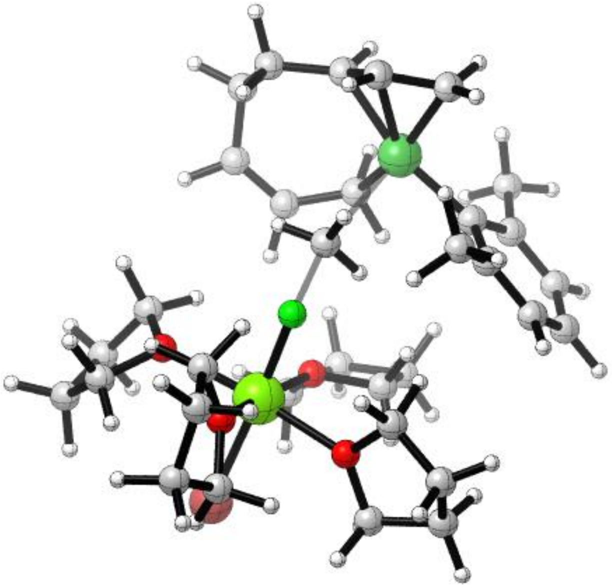

TS10-2,6-dimethylphenyl -Me

Ni	8.30421800	11.70802200	0.18268600
C	6.49111500	11.83123600	-0.61587100
C	6.42097200	11.31246900	0.69170600
C	7.04903700	12.01079100	1.73593100
C	7.13120600	11.49719100	3.13955000
C	8.26083700	12.14139300	3.96175000
C	9.61882600	11.56576300	3.69971900
C	10.27198500	11.63474100	2.52761300
C	9.85588200	12.27956600	1.27258300
C	9.19485900	11.69415700	-1.54429800
C	9.90920500	12.84095200	-1.97499000
C	10.75876300	12.80828400	-3.08390800
C	10.90976400	11.65194000	-3.83920300
C	10.12248100	10.55609700	-3.52027500
C	9.26011900	10.57516200	-2.41357800
H	6.40183500	12.91042900	-0.76965500
H	6.12830000	11.24028300	-1.45283800
H	6.17596800	10.25447200	0.84077600
H	7.01621900	13.10881100	1.67668600
H	7.24766400	10.40056300	3.14516400
H	6.17857200	11.70356400	3.65390700
H	8.24967400	13.22453900	3.75160400
H	8.02443200	12.04331300	5.02879000
H	10.09864800	11.03849400	4.52706400
H	11.23611100	11.11255600	2.48540000
H	10.72902700	12.37185600	0.61305700
H	9.48346700	13.29674300	1.48791800
H	11.30045900	13.71347400	-3.36355700
H	10.13981400	9.67073400	-4.16205100
C	9.41158800	9.53143600	0.64737100
H	10.16015700	10.01912100	0.05060700
F	10.19207900	7.93271500	0.49937200
Mg	12.04905700	7.45176700	0.80528600
Br	14.42592700	6.84670400	1.32896000
O	12.58539400	9.59740700	0.63416500
C	13.66682700	10.20929500	1.36073900
C	12.67981500	10.13785300	-0.70253300
C	13.63387600	11.65873800	0.92106000
H	13.48468900	10.04646900	2.42836700
H	14.60695500	9.70458400	1.09091000
C	13.21505700	11.56385300	-0.55323100
H	13.37087900	9.50855900	-1.28220400
H	11.68828500	10.08953000	-1.16982800
H	14.60189100	12.14888100	1.06148600

H	12.88524600	12.21525800	1.49712800
H	14.06184000	11.72385000	-1.22848500
H	12.44719300	12.30153700	-0.80935900
O	11.50701800	7.19918400	2.88627600
C	11.90685800	6.05766100	3.65421600
C	11.55141000	8.30567800	3.78937700
C	12.97571600	6.55674200	4.62641300
H	11.01824300	5.68163800	4.18651300
H	12.25605000	5.29487000	2.95043900
C	12.82028800	8.08922600	4.60107700
H	11.52294800	9.22435800	3.19200900
H	10.64923500	8.28523300	4.42128800
H	13.97066400	6.27016700	4.27434100
H	12.82417000	6.13801300	5.62639000
H	13.68022300	8.53683900	4.08938400
H	12.74693600	8.53406200	5.59791900
O	11.33554800	5.39227300	0.65615800
C	9.99935700	4.94822000	0.95123400
C	12.10875200	4.32107900	0.09408700
C	9.90131800	3.56086700	0.34216700
H	9.87974400	4.92352400	2.04496700
H	9.29640100	5.68188000	0.54599500
C	11.33983500	3.06875900	0.45610500
H	12.16450100	4.46087900	-0.99800500
H	13.12191100	4.39256100	0.50614300
H	9.60892500	3.62654600	-0.71482800
H	9.17230300	2.92724300	0.85520600
H	11.57170400	2.22310600	-0.19749100
H	11.56288400	2.77385400	1.49062700
O	12.38228300	7.15158900	-1.31009000
C	11.27515100	7.08460200	-2.23066800
C	13.62746600	7.02450200	-2.02618400
C	11.88007200	6.67163600	-3.55765300
H	10.53687600	6.37965300	-1.82772300
H	10.81588300	8.08137200	-2.29408100
C	13.26160400	7.30637000	-3.46825600
H	14.35419000	7.71556600	-1.58757900
H	14.01687800	6.00565700	-1.88478200
H	11.28691400	7.02068900	-4.40827000
H	11.96700900	5.57866200	-3.62600600
H	13.18647500	8.39061800	-3.63546400
H	13.98705200	6.89667900	-4.17667900
H	11.57650300	11.63019200	-4.69929600
C	8.37186700	9.36181000	-2.25910500
H	8.85223200	8.52839200	-1.72675400
H	7.45125500	9.60211300	-1.71754600
H	8.08110700	8.97506100	-3.24404100
H	8.48615600	9.22040900	0.18672900
C	9.75409000	14.16323100	-1.26812200
H	8.71912700	14.32089400	-0.93866200
H	10.38350600	14.23766000	-0.37236000
H	10.02283700	14.99689700	-1.92760500
H	9.44808400	9.62259800	1.72664900

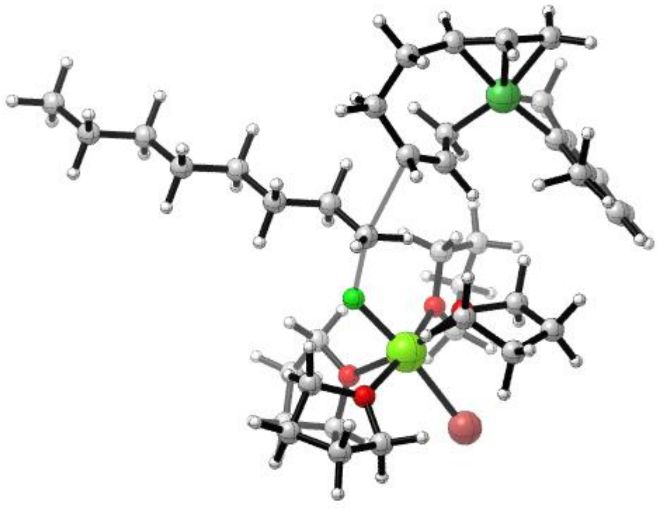

TS3-2,6-dimethylphenyl -*n*-Oct

Ni	8.42163800	11.17494100	-0.42189200
C	7.14202700	9.78659000	0.08272100

C	7.57412000	10.50051700	1.22353800	C	15.86034700	18.16886100	-3.82654600
C	7.53083900	11.90633700	1.26579900	H	16.84441200	16.33003200	-3.07886700
C	8.23744600	12.65160800	2.36370500	H	16.23240500	16.22249500	-4.74502300
C	9.34860300	13.64329200	1.92420800	C	14.37981200	18.26998000	-4.17973300
C	10.44148700	12.97895200	1.15312000	H	12.87131800	16.72317300	-3.70129700
C	10.19897200	12.53075400	-0.13869600	H	13.57923400	17.53423900	-2.28349100
C	9.13973900	12.91356800	-1.00434200	H	16.52226800	18.64400700	-4.55580300
C	9.11689800	10.27794200	-1.95704800	H	16.04668000	18.62694500	-2.84514800
C	8.71664100	10.56042700	-3.28058200	H	14.22869600	18.03063700	-5.24108200
C	9.41557700	10.04682200	-4.37757500	H	13.94382400	19.25413800	-3.98673800
C	10.50218700	9.19755900	-4.19972900	O	16.03042000	14.65369400	-0.98425200
C	10.86729300	8.84601500	-2.90620900	C	17.31973800	14.02304100	-0.84753200
C	10.19674700	9.38006700	-1.79972700	C	15.91209000	15.74967800	-0.05842100
H	6.23207400	10.09423000	-0.44085800	C	17.85084500	14.52758400	0.47769700
H	7.34484200	8.71905100	0.02457000	H	17.18016000	12.93889400	-0.90224300
H	8.25367900	10.00196200	1.92390500	H	17.95268900	14.31942700	-1.69491500
H	6.67392400	12.41697000	0.81147300	C	17.30833300	15.95073500	0.49571800
H	8.71100200	11.92101900	3.03823300	H	15.50741600	16.61344400	-0.59773000
H	7.50913700	13.20586900	2.97526500	H	15.19118800	15.47151400	0.72415900
H	8.88041800	14.45029500	1.33270000	H	18.94033100	14.46546700	0.54703200
H	9.74446000	14.12405300	2.83092200	H	17.42029600	13.94856500	1.30636100
H	11.20306300	12.45313600	1.73711500	H	17.89979500	16.58835900	-0.17589300
H	10.97344700	11.90087900	-0.59398400	H	17.30465600	16.41367200	1.48665800
H	9.34653600	12.90532800	-2.07529400	O	13.67880400	12.41616800	-1.66611800
H	8.48619700	13.73021300	-0.68393300	C	13.28271400	11.31322600	-2.51980200
H	9.08398500	10.30108000	-5.38670900	C	14.23068300	11.84839200	-0.47558400
H	11.68479900	8.13935000	-2.74685800	C	14.15146500	10.12299000	-2.10741800
C	12.05479400	14.56221100	-0.17837500	H	12.21219300	11.11059800	-2.35976100
H	11.94980000	13.71225900	-0.82906300	H	13.43694500	11.63590600	-3.55405400
F	13.38854800	15.22138900	-1.11709300	C	15.03374100	10.66574100	-0.97643200
Mg	14.46947700	14.23470800	-2.44227500	H	14.81205600	12.63341800	0.02540000
Br	16.13053000	13.08066600	-3.89909900	H	13.40683000	11.53617400	0.18965800
O	14.80130000	16.16874000	-3.24824800	H	14.75283300	9.75691100	-2.94436100
C	16.05040800	16.66651600	-3.75597900	H	13.51597600	9.30079600	-1.75905400
C	13.78630300	17.18290800	-3.30534100	H	15.99006400	11.01767800	-1.38382600

H	15.23312900	9.92801500	-0.19358800
O	12.84587400	14.14393900	-3.83347800
C	11.45122100	13.93761600	-3.53279700
C	13.04933900	14.14590800	-5.26335500
C	10.82777100	13.49253300	-4.83763800
H	11.35916500	13.19158100	-2.73773000
H	11.02666900	14.88690200	-3.16567300
C	11.65809400	14.27338400	-5.85086700
H	13.73903300	14.96622700	-5.49848400
H	13.53583000	13.20484600	-5.55419100
H	9.75331000	13.69679000	-4.87826400
H	10.96513100	12.40866800	-4.96310400
H	11.34148700	15.32586000	-5.87374600
H	11.59889600	13.87762900	-6.86860200
C	10.64706200	8.96784300	-0.42266600
H	11.07111700	9.80655600	0.15170100
H	9.80784600	8.58780300	0.17356600
H	11.40880900	8.17786900	-0.46361100
H	11.03283600	8.78745800	-5.05714100
C	11.10164500	15.69732400	-0.24726700
H	10.07356400	15.30431300	-0.22652700
H	11.21408200	16.16836900	-1.23632900
H	12.61602800	14.39068600	0.73649700
C	11.32822900	16.72411600	0.84759900
H	11.22430000	16.22702600	1.82581100
H	12.37030100	17.08252000	0.78884200
C	10.37357800	17.90257600	0.76969000
H	10.46974500	18.38867800	-0.21574200
H	9.33623300	17.53289700	0.82085100
C	10.60180100	18.93017200	1.86511800
H	11.64627200	19.28182800	1.82522000
H	10.48910800	18.44513800	2.84835700
C	9.66510100	20.12425000	1.78005000
H	8.62043200	19.77439800	1.81520000
H	9.78111000	20.61252900	0.79810600
C	9.89219100	21.14792600	2.88100200
H	10.93842800	21.48868000	2.84774800
H	9.76864800	20.65923800	3.85928800
C	8.95600300	22.33942600	2.77536200
H	7.90743500	22.02183100	2.83361100
H	9.12635200	23.07011400	3.57299000
H	9.08552500	22.85748300	1.81667900
C	7.48190500	11.37971400	-3.53577800
H	6.58069800	10.81162300	-3.26603300
H	7.46123400	12.29091100	-2.92502600
H	7.38489300	11.66311900	-4.59229700

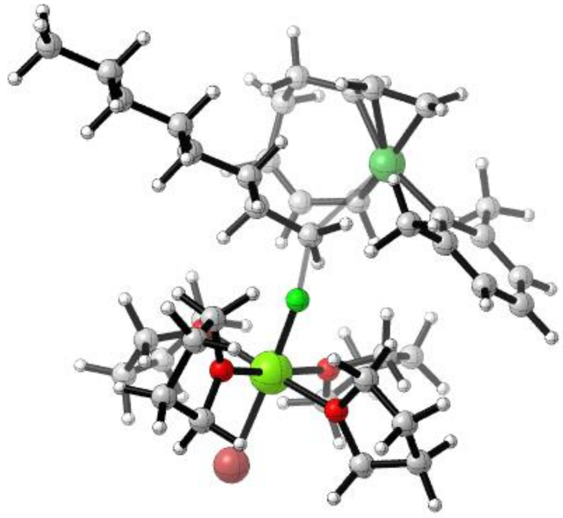

TS10-2,6-dimethylphenyl -*n*-Oct

Ni	8.30004400	11.80625900	0.28132400
C	6.63459100	12.09168500	-0.74473200
C	6.33395000	11.71052700	0.57910500
C	6.91969700	12.42499300	1.63373900
C	6.74829400	12.07919600	3.07823000
C	7.84612900	12.67631400	3.97449700
C	9.14425200	11.92761600	3.95892300
C	9.95012800	11.77616700	2.89318100

C	9.78983400	12.27570900	1.52293500	H	14.80519400	11.95494500	0.90913800
C	9.27908900	11.63465800	-1.39792000	H	13.20739700	11.71926700	1.65011600
C	10.06842000	12.72305200	-1.84977000	H	13.91306800	11.65142100	-1.31389800
C	10.87035100	12.63117300	-2.99065300	H	12.45923500	12.30706300	-0.56074000
C	10.90015400	11.47191900	-3.75487700	O	11.62382000	7.51334600	2.82140400
C	10.06117700	10.42861400	-3.39563200	C	11.62570800	6.36903400	3.68224700
C	9.24909300	10.50445100	-2.25486400	C	12.03976700	8.60894100	3.64101500
H	6.69096400	13.15347400	-0.99880300	C	12.86500700	6.52728400	4.55437800
H	6.31165800	11.46177300	-1.57017900	H	10.69448500	6.38648500	4.27479500
H	5.91454800	10.71866800	0.77780900	H	11.63146400	5.47946200	3.04306300
H	7.06048600	13.50376200	1.46825800	C	13.12571100	8.04667200	4.55537600
H	6.70132800	10.98529200	3.21563500	H	12.36397600	9.41563800	2.97258300
H	5.77802100	12.46823400	3.42744300	H	11.16772100	8.96787300	4.21488700
H	7.99635800	13.72307800	3.65840800	H	13.70733600	6.01027800	4.08281700
H	7.47357800	12.72959000	5.00518200	H	12.70959800	6.11934700	5.55775300
H	9.44881200	11.45933100	4.89686800	H	14.12303300	8.25315300	4.15277600
H	10.83406300	11.14937200	3.06600800	H	13.06685400	8.48993800	5.55417300
H	10.75005500	12.20973000	0.99101400	O	11.17974100	5.38120700	0.67678600
H	9.48480800	13.33661900	1.54994800	C	9.75595500	5.16909200	0.69870500
H	11.46823100	13.49389700	-3.28941900	C	11.86654400	4.15129600	0.37467400
H	10.00132300	9.53946500	-4.02959700	C	9.55248700	3.76709300	0.15607200
C	9.46063300	9.53607700	0.99474900	H	9.40991000	5.25889600	1.74255200
H	10.42363000	10.00372300	1.14036300	H	9.28679300	5.96517700	0.11347300
F	10.34456100	7.96352300	0.29251300	C	10.83141700	3.06977500	0.60208700
Mg	12.11002900	7.33183800	0.70082500	H	12.20732900	4.19252300	-0.67313900
Br	14.36862000	6.41033700	1.30852200	H	12.75210400	4.09145200	1.01562300
O	12.85783500	9.34696500	0.47925800	H	9.49580000	3.78773700	-0.94070100
C	14.09354700	9.88797500	0.96690500	H	8.63674000	3.30160100	0.53235800
C	12.49615400	10.12905800	-0.68454500	H	11.05165600	2.15116400	0.05086900
C	13.87700900	11.37949000	0.84716200	H	10.77663700	2.82153300	1.67108500
H	14.25975800	9.50686800	1.97893400	O	12.42795100	7.05875700	-1.38218000
H	14.92530000	9.53740100	0.33449100	C	11.32901100	7.00576800	-2.30914400
C	13.18320800	11.48735200	-0.51272300	C	13.68329400	7.04469600	-2.08527200
H	12.84083600	9.59648600	-1.58209700	C	11.95640900	6.63656900	-3.63755900
H	11.40407600	10.19185200	-0.72633300	H	10.60218800	6.27393300	-1.93495100

H	10.85005400	7.99464500	-2.33891500
C	13.31688200	7.31575400	-3.53197000
H	14.33874500	7.79677000	-1.63098300
H	14.15225000	6.05996300	-1.94891200
H	11.35965900	6.97781400	-4.48892100
H	12.08064100	5.54810500	-3.71865300
H	13.21229900	8.39577600	-3.70784400
H	14.06065100	6.92735200	-4.23356900
H	11.52320200	11.40717400	-4.64519900
C	8.32627100	9.32515700	-2.03735200
H	8.81783800	8.48856400	-1.51682300
H	7.44257100	9.60152900	-1.45073800
H	7.97076600	8.93464500	-2.99898300
H	8.98553700	9.68086500	0.03923200
C	8.72931600	8.93235800	2.11905300
H	8.46064500	9.74196000	2.82420000
H	9.43820400	8.29950900	2.67495800
C	7.51633800	8.12380200	1.69438700
H	7.82764700	7.40265200	0.92355400
H	6.77941600	8.78528100	1.21160100
C	6.86132900	7.38417100	2.84799200
H	7.62261100	6.78644300	3.37906200
H	6.48389700	8.10895100	3.58812200
C	5.73291700	6.47225600	2.39661200
H	4.98749400	7.06161800	1.83831700
H	6.13117100	5.73848000	1.67419000
C	5.04608300	5.73736500	3.53541000
H	5.79040900	5.14622900	4.09462100
H	4.64663300	6.46917800	4.25656300
C	3.92283900	4.82422000	3.06953800
H	3.18336100	5.41852800	2.51195000
H	4.32465500	4.09614100	2.34799700
C	3.24325400	4.09569600	4.21582800
H	2.43786200	3.44074300	3.86713400
H	3.96007600	3.47562800	4.76886200
H	2.80832400	4.80545000	4.93058400
C	10.04231000	14.04993600	-1.13524800
H	9.02590600	14.30904500	-0.81117800
H	10.66638700	14.05761600	-0.23216200
H	10.39942300	14.85696000	-1.78588100

Table 9

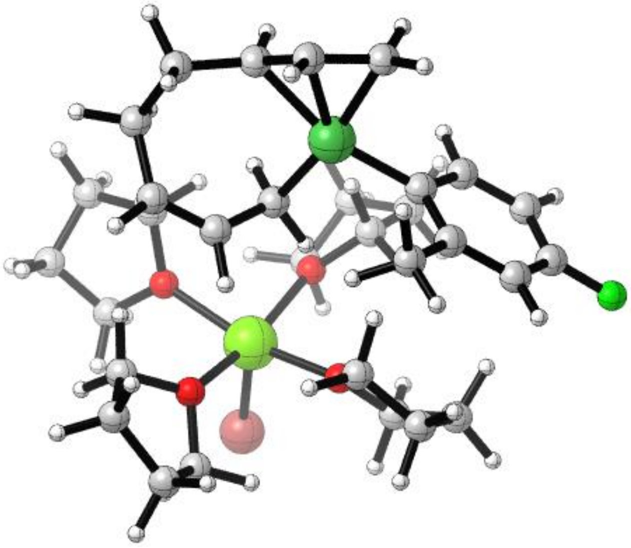

INT1-4-fluoro-2-methyphenyl

Ni	9.51010400	12.80004800	-0.73858500
C	7.57520800	12.85403400	-0.45238800
C	8.24689100	12.48149700	0.73098000
C	9.30132700	13.26597100	1.22999600
C	10.10242900	12.82790200	2.41623100
C	11.62645100	13.01584100	2.26511400
C	12.21617200	12.01559600	1.32422300
C	12.06726300	12.07391300	-0.01968000
C	11.44986800	13.13061800	-0.81238300
C	9.23887000	12.67681900	-2.61513800
C	9.08011900	13.82836100	-3.40305500
C	8.91785300	13.80754800	-4.79073800
C	8.87994600	12.57699500	-5.41918500
C	8.98520100	11.39846100	-4.69863400
C	9.16302100	11.44895000	-3.31241800
H	7.31720400	13.90170400	-0.63295000
H	6.88047300	12.14727000	-0.90273300
H	8.17478200	11.44668000	1.08378200
H	9.22541900	14.35674600	1.11700600
H	9.90649600	11.76385300	2.62151800
H	9.77594800	13.38160100	3.31098800
H	11.80268100	14.05259500	1.92777600
H	12.09829400	12.93204400	3.25401500
H	12.61955800	11.10158400	1.76596700
H	12.41276300	11.19739800	-0.58537400
H	11.82326100	13.13971300	-1.84640200
H	11.57239200	14.12932800	-0.36518500
H	8.80809100	14.71771600	-5.37808700
H	8.91575000	10.44746100	-5.22817400
Mg	14.50495000	13.58146500	-2.50803500
Br	16.53982900	14.08617300	-3.77318900
O	13.34665100	15.23838400	-2.95950100
C	13.96036100	16.52877300	-3.16978300
C	11.94675400	15.28350400	-3.33103900
C	12.79272100	17.48126100	-3.31847000
H	14.62388500	16.72616600	-2.31871700
H	14.57619400	16.48012600	-4.07890500
C	11.78222400	16.60388100	-4.05072800
H	11.71689600	14.39757900	-3.93220000
H	11.34406500	15.23044600	-2.41500000
H	13.06329500	18.39203700	-3.85986100
H	12.40211600	17.76815100	-2.33267100
H	12.06097800	16.50699100	-5.10881200
H	10.75284600	16.97111200	-4.00154700
O	15.01568800	14.51979300	-0.71853500
C	16.38755700	14.78407200	-0.35080300
C	14.11886500	15.04374600	0.29091600
C	16.32569300	15.05897100	1.13504500
H	16.99033600	13.91840200	-0.64406700
H	16.74637800	15.65188400	-0.92272500
C	15.00784400	15.81675900	1.24594200
H	13.36004000	15.65566800	-0.21287500
H	13.62573400	14.18870500	0.77520600
H	17.19095400	15.62353200	1.49331200
H	16.27175900	14.11498100	1.69449300
H	15.13903800	16.85213100	0.90402900

H	14.59618500	15.84244300	2.25881700
O	15.07215500	11.74771100	-1.67422900
C	15.84266900	10.82097800	-2.45885900
C	15.36399100	11.40915600	-0.31082600
C	15.65883700	9.48632900	-1.74615300
H	15.47330800	10.86568500	-3.48857300
H	16.89093200	11.15577400	-2.45690800
C	15.32722800	9.88630300	-0.29269900
H	16.36747400	11.79384900	-0.06242600
H	14.60574400	11.88167100	0.32320000
H	16.55939200	8.86988900	-1.82129200
H	14.83528600	8.91658900	-2.18976600
H	16.03475100	9.47555900	0.43338500
H	14.32681300	9.53843000	-0.01118000
O	13.44771700	12.48966700	-3.92519400
C	12.53507400	11.37402800	-3.74670600
C	13.59773800	12.78176400	-5.33652900
C	12.38193600	10.77658500	-5.12641200
H	12.96749700	10.70035700	-2.99668700
H	11.57867400	11.76575400	-3.37367200
C	12.47469100	12.01883700	-6.00632600
H	13.56671300	13.87167200	-5.45893900
H	14.58916000	12.43330000	-5.65902000
H	11.42991500	10.24582100	-5.22866600
H	13.20333700	10.08117900	-5.34981300
H	11.52926200	12.57803500	-5.95682600
H	12.69081500	11.79974700	-7.05561200
C	9.29417800	10.16421300	-2.54498400
H	10.23666700	10.13700500	-1.97736100
H	8.49398300	10.06965500	-1.79833400
H	9.25787300	9.28048300	-3.19538000
H	9.08217800	14.80708000	-2.91316300
F	8.74761400	12.51709600	-6.76097300

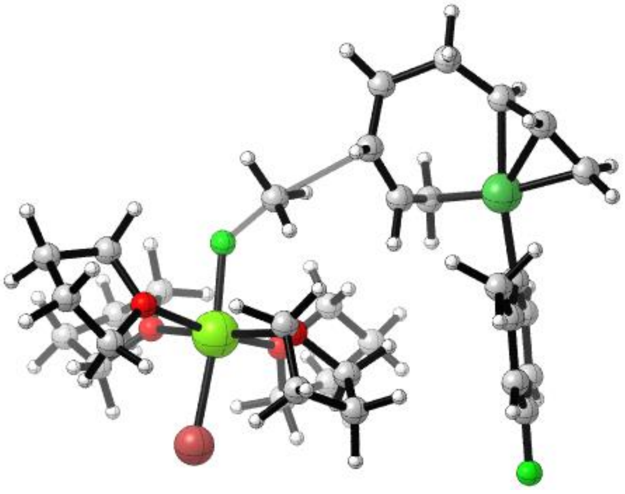

TS3-4-fluoro-2-methyphenyl

Ni	8.55487300	11.21476700	-0.34512500
C	7.26799400	9.85762100	0.23225000
C	7.67962600	10.64525300	1.32922100
C	7.63445800	12.05061100	1.27257100
C	8.32800100	12.86918000	2.32589400
C	9.48894500	13.77827600	1.84163200
C	10.56598800	13.01998200	1.13598700
C	10.32116000	12.52200800	-0.14068400
C	9.27922600	12.91247800	-1.02529800
C	9.22845300	10.22218500	-1.83354600
C	8.87957200	10.51368300	-3.16161200
C	9.43751600	9.87264200	-4.27009800
C	10.39000100	8.89744900	-4.04365900
C	10.77955400	8.55914100	-2.75896300
C	10.20902600	9.21699500	-1.66334800
H	6.37303600	10.13310200	-0.33231500
H	7.46982600	8.78839100	0.24375800
H	8.34812300	10.19668900	2.07287300
H	6.78383300	12.52631900	0.77183700
H	8.75293200	12.18607000	3.07835300
H	7.59800300	13.49628000	2.85993200
H	9.07793600	14.55942300	1.17993500

H	9.88880000	14.30633200	2.71932000	H	15.03957100	15.65291800	0.71515900
H	11.28156900	12.47563800	1.76136300	H	18.82094900	14.49200400	0.64419000
H	11.09103500	11.86834000	-0.56966000	H	17.29549300	13.99219600	1.40594400
H	9.44828400	12.81985200	-2.09828200	H	17.82331000	16.60290000	-0.10773500
H	8.66136800	13.76774500	-0.74072000	H	17.16953300	16.45753500	1.53177800
H	9.13788300	10.10817300	-5.28941200	O	13.66194100	12.35702800	-1.73936900
H	11.52106800	7.77132700	-2.62435900	C	13.15891600	11.35656700	-2.66552400
C	12.19918600	14.50349300	-0.09246800	C	14.31309000	11.65304600	-0.67250800
H	12.37433400	15.04159700	0.83344200	C	13.95600400	10.07937400	-2.38939200
H	12.60968000	13.51296300	-0.17595800	H	12.08212500	11.21223200	-2.48050200
H	11.26968100	14.70539200	-0.60990900	H	13.30644700	11.75542400	-3.67369700
F	13.24206800	15.25941400	-1.13664100	C	15.01217700	10.50734000	-1.36863400
Mg	14.45626900	14.26345300	-2.41648900	H	14.96474600	12.36797500	-0.15649800
Br	16.15654500	13.18728100	-3.87476200	H	13.54955900	11.29481300	0.04071800
O	14.86936200	16.23030700	-3.16052100	H	14.40168600	9.67530000	-3.30268000
C	16.12527300	16.67213000	-3.70750500	H	13.29080300	9.31465400	-1.97057900
C	13.87103200	17.25514600	-3.30390100	H	15.90747700	10.88165700	-1.88108000
C	15.97530100	18.17374000	-3.83224900	H	15.29736000	9.70377200	-0.68326900
H	16.92356300	16.34435500	-3.03175600	O	12.87834400	14.27271200	-3.81334200
H	16.28157500	16.18217500	-4.67860800	C	11.48201800	14.19258800	-3.46530100
C	14.50279900	18.29724800	-4.20735200	C	13.04411700	14.26316200	-5.24533700
H	12.96227500	16.79239500	-3.70756800	C	10.78920600	13.77055100	-4.74219600
H	13.63926400	17.65768200	-2.30795400	H	11.36867400	13.48752600	-2.63690500
H	16.65813000	18.60652200	-4.56851200	H	11.14831900	15.18376300	-3.11770500
H	16.16122600	18.65855800	-2.86355500	C	11.64550000	14.46888400	-5.79579400
H	14.35958900	18.02942700	-5.26278700	H	13.77169600	15.04390300	-5.50076800
H	14.08697600	19.29656700	-4.05146300	H	13.46984000	13.29668300	-5.54805600
O	15.88572300	14.60154300	-0.84731800	H	9.73522600	14.06230600	-4.76010800
C	17.20496000	14.02719100	-0.74605400	H	10.83676500	12.67823400	-4.85533800
C	15.81203200	15.80232200	-0.04918000	H	11.39874900	15.53911100	-5.83526200
C	17.73184700	14.55367200	0.56917300	H	11.52980600	14.05625100	-6.80179600
H	17.10576000	12.93961900	-0.80805400	C	10.67756900	8.84308400	-0.28383400
H	17.80980400	14.35473800	-1.60264000	H	11.17352200	9.68771200	0.21947200
C	17.19879300	15.98120600	0.54772900	H	9.83563800	8.56226800	0.36118500
H	15.49944700	16.63115900	-0.69606600	H	11.38412000	8.00355700	-0.30234100

H	8.12671800	11.28266000	-3.35099100
F	10.95048500	8.25737400	-5.08880000

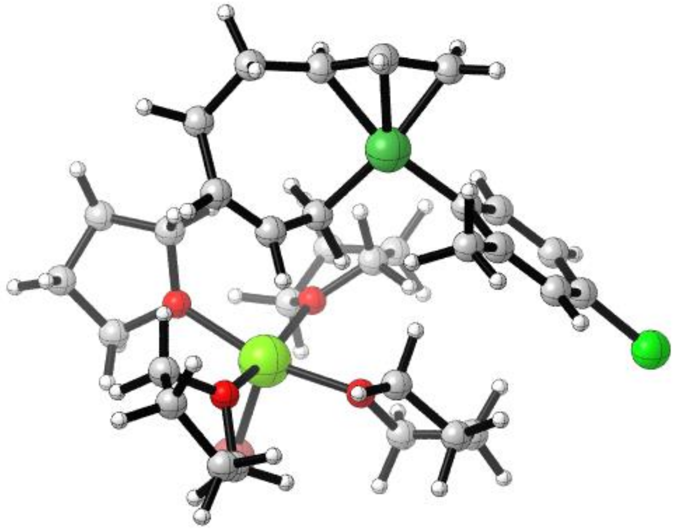

INT1-4-chloro-2-methyphenyl

Ni	9.51784500	12.83782800	-0.78443200
C	7.58417000	12.98477700	-0.51661300
C	8.23085600	12.61360400	0.68036600
C	9.31901500	13.35939200	1.16823800
C	10.09791200	12.89530300	2.35977300
C	11.62703400	13.03927400	2.21947200
C	12.20724900	12.02075400	1.29041700
C	12.07203000	12.06515800	-0.05524900
C	11.46599200	13.11995300	-0.86127000
C	9.25779700	12.56540800	-2.64063000
C	9.12105000	13.61169900	-3.56714500
C	8.96656400	13.41770600	-4.94160800
C	8.91777100	12.11771700	-5.41900300
C	8.99654300	11.03771100	-4.54848800
C	9.16663700	11.26019200	-3.18080300
H	7.37491700	14.03759000	-0.72787900
H	6.86318700	12.29678900	-0.95467500
H	8.10990300	11.59102900	1.05505200
H	9.28908200	14.45113100	1.04507600
H	9.86830700	11.83713300	2.56037700
H	9.78281800	13.45640100	3.25404200
H	11.83042300	14.07052800	1.88069700
H	12.08837500	12.94901900	3.21272200
H	12.60708100	11.11185400	1.74526400
H	12.42130800	11.18353000	-0.61199200
H	11.83044700	13.10814400	-1.89832000
H	11.60741200	14.12227200	-0.42817300
H	8.87670100	14.25654900	-5.62968600
H	8.92703500	10.02303200	-4.94207900
Mg	14.47922400	13.60618500	-2.48799200
Br	16.54225800	14.10119200	-3.71965800
O	13.34826900	15.29654200	-2.91055100
C	14.00129300	16.57188800	-3.10320800
C	11.93864700	15.40441800	-3.22786700
C	12.86581800	17.56749800	-3.21334100
H	14.68378200	16.73048500	-2.25917300
H	14.60164800	16.52115800	-4.02221800
C	11.80606600	16.73764100	-3.93030900
H	11.64697700	14.53356900	-3.82548300
H	11.37044600	15.37526800	-2.28783400
H	13.15724300	18.47412700	-3.75078700
H	12.50905300	17.85760000	-2.21569100
H	12.05514000	16.64174000	-4.99579200
H	10.79429000	17.14581300	-3.85176100
O	15.01769600	14.51791100	-0.68929400
C	16.39136000	14.76669300	-0.31805100
C	14.12631200	15.03529100	0.32712000
C	16.33218400	15.00043800	1.17465300
H	16.98909800	13.90792600	-0.63901900
H	16.75270900	15.64886000	-0.86594500
C	15.02256000	15.76911700	1.30669900
H	13.38064500	15.67369900	-0.16454900

H	13.61711200	14.17762700	0.78837800
H	17.20313700	15.54512500	1.54946500
H	16.26749000	14.04141500	1.70677800
H	15.16590400	16.81319700	0.99806200
H	14.60888000	15.76761000	2.31918200
O	15.02617700	11.75626000	-1.69486600
C	15.71734600	10.80452900	-2.52052900
C	15.36183700	11.39702900	-0.34712300
C	15.47036100	9.47718000	-1.82036400
H	15.31532700	10.89077100	-3.53581700
H	16.78300600	11.07852900	-2.54763500
C	15.31150300	9.87093300	-0.33684100
H	16.37662700	11.77018800	-0.13067500
H	14.63077800	11.87260600	0.31665600
H	16.28768300	8.77076400	-1.99125700
H	14.55093900	9.01323500	-2.19517100
H	16.09911900	9.45216000	0.29638500
H	14.35113100	9.52232400	0.05839400
O	13.43194400	12.57241000	-3.95612400
C	12.54285100	11.42558300	-3.86470900
C	13.61094100	12.94496700	-5.34610300
C	12.40488300	10.92982000	-5.28768400
H	12.98729600	10.70777700	-3.16316700
H	11.57856900	11.76438600	-3.46308000
C	12.49157100	12.23411300	-6.07193000
H	13.59486700	14.04046400	-5.40105200
H	14.60358800	12.60286400	-5.67202300
H	11.45966500	10.39939300	-5.44049900
H	13.23586500	10.26253300	-5.55697900
H	11.54566600	12.78480600	-5.97237400
H	12.70044100	12.09491500	-7.13618300
C	9.28001900	10.08052900	-2.25815400
H	10.23929900	10.09414000	-1.71945100
H	8.49960500	10.11038000	-1.48580500
H	9.19712800	9.12328500	-2.78762000
H	9.12542400	14.64397100	-3.20482800
Cl	8.77347900	11.82149900	-7.15365300

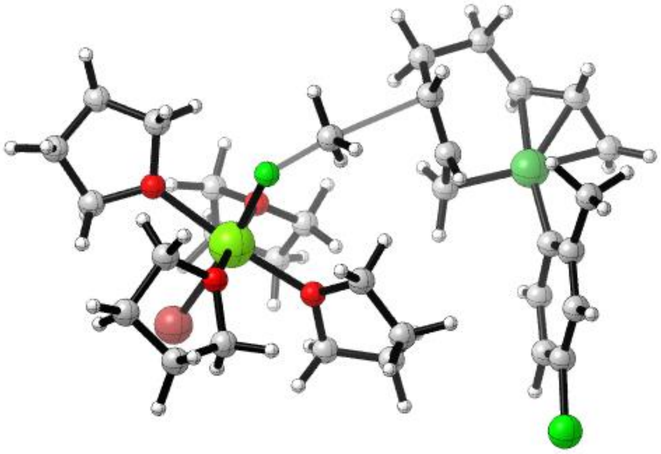

TS3-4-chloro-2-methyphenyl

Ni	7.87026500	11.83904000	-0.67231700
C	6.26571500	10.96814900	0.02807700
C	6.91965500	11.69395800	1.04818700
C	7.26988500	13.04402000	0.86574500
C	8.17985000	13.73076300	1.84483800
C	9.54057600	14.22824000	1.28932000
C	10.35222500	13.12821200	0.68793600
C	9.97112300	12.58613600	-0.53379200
C	9.08204200	13.15888800	-1.48568400
C	8.15632900	10.60957400	-2.09537300
C	7.77613400	10.89764400	-3.41480600
C	8.15517400	10.12700700	-4.51338400
C	8.96436400	9.02516000	-4.28946600
C	9.34172500	8.66560200	-3.00277200
C	8.92797200	9.43915000	-1.91400500
H	5.46462300	11.43905900	-0.54815700
H	6.15619200	9.89127700	0.13636200
H	7.45336100	11.13875800	1.82789800
H	6.57739800	13.69120000	0.31524900

H	8.40827800	13.03145800	2.66419600	H	14.58827400	14.05233600	-5.82248700
H	7.66186700	14.58441200	2.30768600	C	17.16844200	14.70936000	-4.64692800
H	9.35396500	15.02194300	0.54398800	H	16.23926200	15.56212800	-2.82707300
H	10.08412700	14.71218100	2.11421400	H	16.75115400	13.86093700	-2.69066100
H	10.89728900	12.48187400	1.38156800	H	16.83218700	13.53748600	-6.47986400
H	10.51128100	11.69074200	-0.86403600	H	17.13398700	12.57808500	-5.02090000
H	9.24612600	12.92427600	-2.53985500	H	16.91374700	15.62696600	-5.19421700
H	8.73375200	14.17890000	-1.30297900	H	18.24701800	14.69200200	-4.46581500
H	7.85165700	10.38796000	-5.52556500	O	12.20729300	13.75601700	-4.09517200
H	9.96024900	7.78164600	-2.85060500	C	11.56801000	13.61151300	-5.38661000
C	12.25498200	14.13994100	-0.57794500	C	12.21163900	12.47332600	-3.41441100
H	12.43284300	14.71316800	0.32562800	C	10.73832200	12.35439000	-5.26026300
H	12.56819900	13.10659300	-0.56290400	H	11.00161000	14.52635600	-5.58789000
H	11.39797600	14.41479000	-1.17974500	H	12.34678600	13.52126300	-6.15798400
F	13.45358400	14.72026600	-1.56648100	C	11.63283200	11.47333100	-4.39696400
Mg	13.30376300	15.44904600	-3.40599000	H	13.24135400	12.26144800	-3.09978500
Br	13.41858300	16.57686200	-5.62151100	H	11.57351100	12.56968700	-2.52945400
O	14.45624800	17.07072800	-2.49022200	H	10.49691200	11.90374400	-6.22780100
C	15.23458500	18.08362000	-3.16613400	H	9.79323500	12.55597500	-4.73672100
C	14.51331700	17.27393100	-1.06303200	H	12.42134600	11.01393500	-5.00837200
C	16.05474200	18.73340400	-2.07114100	H	11.07823200	10.67230900	-3.89312100
H	15.81673500	17.59812600	-3.95576900	O	11.51672800	16.49720900	-2.76269400
H	14.54879000	18.79431900	-3.64895100	C	10.19556400	16.07712200	-3.14212300
C	15.09665400	18.66062400	-0.88902800	C	11.45378500	17.93132600	-2.78348000
H	13.50386600	17.14709900	-0.65708900	C	9.80099000	17.00640700	-4.28162400
H	15.15909300	16.49944600	-0.62538000	H	10.23804400	15.01145400	-3.38754800
H	16.36629700	19.74943700	-2.32857900	H	9.53430400	16.19819300	-2.26934000
H	16.95692600	18.14147400	-1.86315200	C	10.61211000	18.28726800	-4.00551700
H	14.31192500	19.42290500	-0.98917200	H	10.97836600	18.26374200	-1.84719200
H	15.57818900	18.79462700	0.08368800	H	12.48014400	18.30513200	-2.81806900
O	15.04909800	14.22235000	-3.82172400	H	8.72062500	17.17634000	-4.30215900
C	15.16850500	13.50198400	-5.07089100	H	10.09435200	16.58168400	-5.24815500
C	16.35021500	14.62178300	-3.37745500	H	9.97766900	19.15727900	-3.81066800
C	16.65402900	13.48820000	-5.40227000	H	11.26340600	18.51072900	-4.85607500
H	14.73608300	12.50137700	-4.93702400	C	9.30940600	8.99356000	-0.52833200

H	9.86381900	9.76778900	0.01971500
H	8.41349400	8.78190200	0.07019500
H	9.92325300	8.08499900	-0.54183500
H	7.17053200	11.78672800	-3.60671700
Cl	9.55994500	8.08885400	-5.66315900

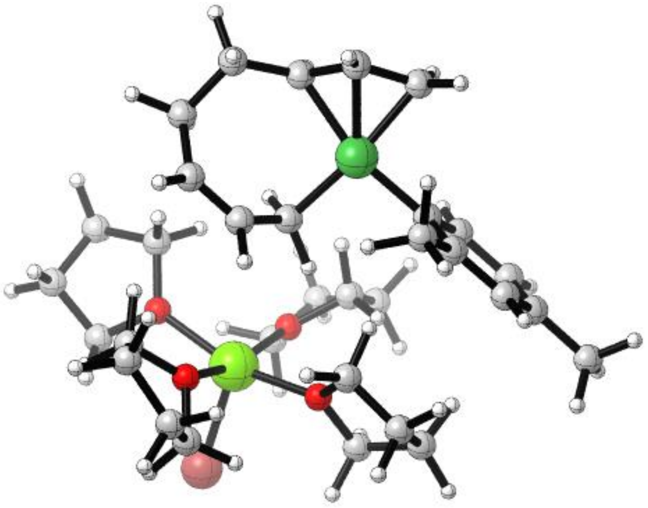

INT1-2,4-dimethyphenyl

Ni	9.53459100	12.82775100	-0.76520300
C	7.59997400	12.93384100	-0.49572300
C	8.25299000	12.55292200	0.69541700
C	9.32420800	13.31271700	1.19798900
C	10.10929900	12.85406400	2.38705700
C	11.63651800	13.01894500	2.24496800
C	12.22317100	12.01181600	1.30829600
C	12.08572700	12.06642300	-0.03723500
C	11.48053600	13.12565600	-0.83646200
C	9.26775400	12.64153000	-2.63441500
C	9.13106000	13.74270100	-3.49222600
C	8.96328600	13.62796600	-4.87416000
C	8.88833200	12.37374300	-5.47540100
C	8.98003800	11.25736900	-4.63777700
C	9.16774200	11.37642200	-3.26086200
H	7.37021000	13.98615600	-0.68706900
H	6.89281300	12.24058000	-0.94770500
H	8.15219300	11.52184700	1.05248700
H	9.27448700	14.40499100	1.08516300
H	9.89446800	11.79200200	2.58372200
H	9.78757800	13.40717300	3.28408900
H	11.82733800	14.05396000	1.91037700
H	12.10149400	12.92870600	3.23668200
H	12.61834500	11.09735800	1.75618600
H	12.43357300	11.18744700	-0.59904100
H	11.84527600	13.12307200	-1.87320600
H	11.61372800	14.12593400	-0.39617300
H	8.88202000	14.52534100	-5.49287700
H	8.90060900	10.25915200	-5.08153600
Mg	14.46691100	13.60388800	-2.47693300
Br	16.51561600	14.09567600	-3.73699500
O	13.33480000	15.28811700	-2.90103200
C	13.97100900	16.56876800	-3.10486200
C	11.93393100	15.36189200	-3.26557800
C	12.82113800	17.54255200	-3.25605700
H	14.63469700	16.75284500	-2.25088800
H	14.58972400	16.51250400	-4.01156100
C	11.79488800	16.68341300	-3.98809900
H	11.68178200	14.47863800	-3.86113500
H	11.33343300	15.32745900	-2.34660700
H	13.10959900	18.44742000	-3.79819900
H	12.43452700	17.83826000	-2.27133800
H	12.07246200	16.57935800	-5.04583900
H	10.77290300	17.07068300	-3.94040600
O	15.01831800	14.52282400	-0.68602700
C	16.39471700	14.77946700	-0.33258500
C	14.13579800	15.04190700	0.33790200
C	16.35241900	15.02314000	1.15916900
H	16.99212300	13.92063400	-0.65430100
H	16.74616600	15.65928700	-0.89077000

C	15.04068500	15.78634300	1.30151200
H	13.38190800	15.67379200	-0.14911800
H	13.63495900	14.18484500	0.80990400
H	17.22514800	15.57466900	1.51968000
H	16.29880300	14.06743600	1.69842600
H	15.17544100	16.82876200	0.98333100
H	14.63960700	15.79069600	2.31897400
O	15.03232300	11.76072800	-1.67973200
C	15.72134300	10.80778300	-2.50496200
C	15.37097000	11.40575500	-0.33165000
C	15.48063400	9.48181200	-1.79984600
H	15.31397400	10.88949500	-3.51843100
H	16.78635000	11.08377800	-2.53825200
C	15.32399500	9.87956700	-0.31730500
H	16.38517100	11.78214100	-0.11769200
H	14.63905800	11.88035400	0.33197700
H	16.30004100	8.77770300	-1.97059900
H	14.56187100	9.01354600	-2.17093700
H	16.11338800	9.46391800	0.31579800
H	14.36487800	9.53022100	0.08020600
O	13.41545600	12.55093300	-3.93264800
C	12.52452600	11.40798200	-3.80971200
C	13.57901600	12.89772200	-5.32913100
C	12.36457100	10.88707000	-5.22067500
H	12.97717900	10.70082600	-3.10299200
H	11.56532400	11.75828600	-3.40586400
C	12.44931000	12.17665000	-6.03013800
H	13.56355500	13.99201500	-5.40453000
H	14.56743600	12.54927100	-5.66183400
H	11.40912900	10.36581800	-5.34394000
H	13.18388600	10.20552800	-5.48983400
H	11.50482500	12.73136400	-5.92830800
H	12.65139000	12.01761400	-7.09341300
C	9.28903800	10.13659800	-2.42076900
H	10.22927800	10.13562100	-1.84861700
H	8.48462400	10.08437800	-1.67386400
H	9.25189100	9.21837300	-3.02160300
H	9.15699100	14.75057100	-3.06476500
C	8.70527000	12.21112700	-6.95587300
H	9.42631800	11.49623600	-7.37744300
H	7.70533300	11.83394700	-7.20822000
H	8.83657300	13.16434900	-7.48155700

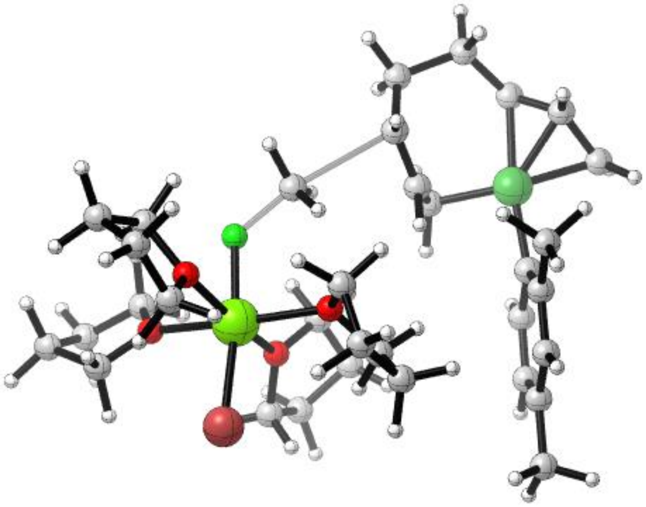

TS3-2,4-dimethyphenyl

Ni	8.55035500	11.22691700	-0.33275100
C	7.26474100	9.86999500	0.24738200
C	7.66234700	10.67212800	1.33930000
C	7.61249800	12.07691500	1.26786700
C	8.29872300	12.90749500	2.31706800
C	9.45968700	13.81607600	1.83099500
C	10.54288500	13.05697100	1.13533100
C	10.30375600	12.54671000	-0.13763700
C	9.26348300	12.92261300	-1.03032400
C	9.27154900	10.21789400	-1.78499000
C	8.98892600	10.48624400	-3.13217700
C	9.62859900	9.84384200	-4.19424800
C	10.59549600	8.87199700	-3.95490600

C	10.88363600	8.57409700	-2.62139400	H	14.11358200	19.32600300	-3.96463400
C	10.25949500	9.22930200	-1.55835800	O	15.85329800	14.54724200	-0.86666900
H	6.37408100	10.13464700	-0.32903800	C	17.15881000	13.94252900	-0.76145400
H	7.47226400	8.80211500	0.27298200	C	15.79864700	15.74193900	-0.05805700
H	8.32656900	10.23453000	2.09338500	C	17.69517000	14.45751800	0.55478600
H	6.76351100	12.54524900	0.75764000	H	17.03387400	12.85672900	-0.82143700
H	8.72228400	12.23206800	3.07730900	H	17.77178400	14.25545300	-1.61772000
H	7.56464300	13.53715600	2.84268800	C	17.18910300	15.89490400	0.53619900
H	9.04902200	14.59117100	1.16180600	H	15.49497500	16.58016200	-0.69689300
H	9.85348100	14.35203900	2.70673800	H	15.02671600	15.59696500	0.70790900
H	11.25919900	12.52046300	1.76663900	H	18.78293000	14.37510900	0.62961600
H	11.07750500	11.89360800	-0.55924000	H	17.24853200	13.90329300	1.39098800
H	9.43573100	12.81552600	-2.10147700	H	17.82365300	16.50600900	-0.11959400
H	8.63795300	13.77624900	-0.75830700	H	17.17037100	16.37008800	1.52105800
H	9.36732400	10.09666200	-5.22448200	O	13.62607600	12.35986100	-1.77973200
H	11.62855200	7.80083100	-2.40671800	C	13.11595800	11.37215500	-2.71500600
C	12.18661200	14.51994400	-0.11383200	C	14.26998700	11.62118900	-0.73356000
H	12.36305800	15.06292900	0.80892300	C	14.00965700	10.13815200	-2.56026400
H	12.59252100	13.52597800	-0.18926900	H	12.06881600	11.15155300	-2.45231400
H	11.25514000	14.71833800	-0.62910200	H	13.15212100	11.82764300	-3.70886900
F	13.22595400	15.26657100	-1.16130700	C	15.01362600	10.52723900	-1.46854100
Mg	14.43033000	14.25568800	-2.44691000	H	14.89122200	12.32421100	-0.16631100
Br	16.12542000	13.20494900	-3.93325400	H	13.49784300	11.20884300	-0.06002500
O	14.85967100	16.22833100	-3.15893400	H	14.51234200	9.88159600	-3.49768000
C	16.12002000	16.66877100	-3.69567700	H	13.39684100	9.28212200	-2.25372200
C	13.87099700	17.26520800	-3.27960100	H	15.92240500	10.94241300	-1.92358100
C	15.98862300	18.17494000	-3.77726300	H	15.28690100	9.69088900	-0.81837600
H	16.91385300	16.30903300	-3.03096500	O	12.84834400	14.29502000	-3.83872600
H	16.26821200	16.20453300	-4.68063600	C	11.45322700	14.20762300	-3.48536200
C	14.51780000	18.32686500	-4.14941000	C	13.00843400	14.30283100	-5.27121100
H	12.96116500	16.82241300	-3.70326900	C	10.75722300	13.78980600	-4.76171800
H	13.63500100	17.63732500	-2.27313100	H	11.34433200	13.49719500	-2.66059600
H	16.67655000	18.62026100	-4.50124800	H	11.11821500	15.19600500	-3.13142400
H	16.18019500	18.63030200	-2.79545400	C	11.60536100	14.49852700	-5.81508000
H	14.37349200	18.09215500	-5.21256400	H	13.72544900	15.09516200	-5.52162100

H	13.44591800	13.34501900	-5.58459700
H	9.70223500	14.07818600	-4.77318000
H	10.80599500	12.69768100	-4.87938000
H	11.34949400	15.56674100	-5.84953500
H	11.48947000	14.08953900	-6.82259800
C	10.67922300	8.88575900	-0.15479300
H	11.08290200	9.76171400	0.37590300
H	9.82970000	8.53738100	0.44682900
H	11.44693400	8.10115600	-0.13687600
H	8.23470700	11.24065500	-3.37165700
C	11.30372900	8.15918100	-5.06887500
H	12.39266100	8.16337800	-4.92242400
H	11.00021400	7.10619000	-5.13955700
H	11.09589100	8.62422300	-6.03935300

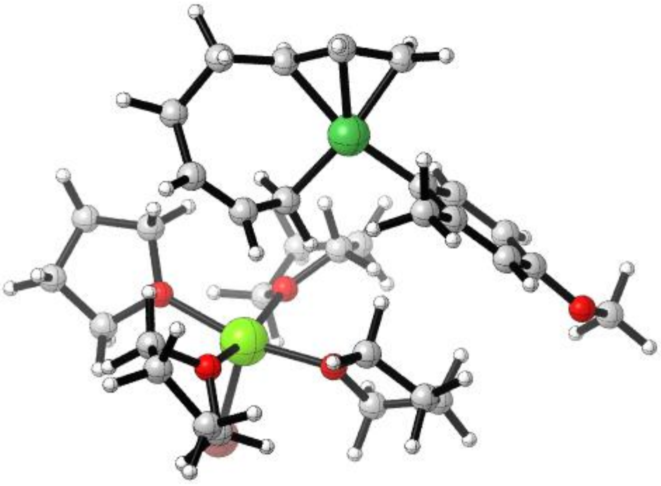

INT1-4-methoxy-2-methyphenyl

Ni	9.52605600	12.89956100	-0.74452000
C	7.59717500	13.08141300	-0.47500700
C	8.23673600	12.68750400	0.71861200
C	9.33973000	13.40770400	1.21285200
C	10.11039000	12.91838900	2.39969200
C	11.64206100	13.03399000	2.25811100
C	12.19975400	12.01032000	1.32094500
C	12.06109700	12.06634300	-0.02435800
C	11.48120300	13.13962100	-0.82349000
C	9.25907800	12.64830800	-2.60867400
C	9.12897400	13.69362400	-3.52881700
C	8.98300000	13.51328200	-4.91228500
C	8.93236200	12.21875900	-5.41594500
C	8.99667300	11.14179900	-4.52996400
C	9.15854600	11.34778100	-3.16295000
H	7.40772000	14.13958800	-0.67803800
H	6.86490800	12.41011000	-0.92012300
H	8.09624200	11.66386300	1.08380500
H	9.32980300	14.50121000	1.10125900
H	9.86089600	11.86293100	2.59093300
H	9.80738600	13.47719900	3.29979900
H	11.86423400	14.06333800	1.92528900
H	12.10345100	12.92867800	3.25002900
H	12.57603000	11.08730600	1.76772700
H	12.38301800	11.17722700	-0.58443500
H	11.83749800	13.12345100	-1.86387500
H	11.64624400	14.13522800	-0.38383100
H	8.90309300	14.37885200	-5.56737700
H	8.91931600	10.13467100	-4.94368600
Mg	14.44754000	13.63223200	-2.47202300
Br	16.50722500	14.14628400	-3.71385400
O	13.33612700	15.34785300	-2.82327100
C	14.00017100	16.62056500	-2.99217200
C	11.92133100	15.47996500	-3.11181500
C	12.87436500	17.63103800	-3.06076300
H	14.69545700	16.75105800	-2.15377800
H	14.58859700	16.58647500	-3.91974100
C	11.79358000	16.83087500	-3.78000500
H	11.60593400	14.62761100	-3.72316100
H	11.36885700	15.43496900	-2.16313700
H	13.16806500	18.54744300	-3.58018200

H	12.53672700	17.90020000	-2.05068000
H	12.02464900	16.75799000	-4.85155300
H	10.78793000	17.24824700	-3.67519400
O	15.04113700	14.49162600	-0.65666900
C	16.42287500	14.72564700	-0.31035900
C	14.17430500	14.99670900	0.38673000
C	16.39783000	14.93075800	1.18769800
H	17.00789000	13.87036900	-0.66278700
H	16.77802800	15.61689100	-0.84767400
C	15.09574000	15.70369800	1.36298400
H	13.42506500	15.65180400	-0.07704900
H	13.66512300	14.13487100	0.84017900
H	17.27966600	15.46409600	1.55343100
H	16.33961800	13.96272700	1.70375500
H	15.23931000	16.75337100	1.07379000
H	14.70301800	15.68318400	2.38358600
O	14.98989800	11.76107400	-1.72960800
C	15.67817500	10.82586700	-2.57573400
C	15.33110300	11.37486200	-0.39001800
C	15.42929900	9.48509500	-1.90224300
H	15.27645900	10.93387100	-3.58929800
H	16.74465100	11.09764800	-2.59797200
C	15.27951200	9.84912700	-0.41008300
H	16.34699800	11.74350400	-0.17110800
H	14.60346800	11.83632500	0.28766300
H	16.24295400	8.77911900	-2.09176000
H	14.50585100	9.03305200	-2.28161800
H	16.07082100	9.41709000	0.20957400
H	14.32111500	9.49488000	-0.01483000
O	13.40055700	12.65235900	-3.97459200
C	12.50845800	11.50368100	-3.92496100
C	13.56661800	13.08452500	-5.34771000
C	12.33148000	11.08672700	-5.36959900
H	12.97188200	10.74331900	-3.28392100
H	11.55767800	11.82120400	-3.47594700
C	12.42414800	12.42692200	-6.08779700
H	13.56782000	14.18140400	-5.35400200
H	14.54782700	12.73985600	-5.70454500
H	11.37249200	10.58372200	-5.52792600
H	13.14567800	10.42172100	-5.69100900
H	11.48897600	12.98551500	-5.93738400
H	12.61385800	12.33932300	-7.16152700
C	9.25264500	10.15947100	-2.24930500
H	10.21352200	10.15105400	-1.71291700
H	8.47556100	10.19858500	-1.47389500
H	9.15086800	9.20785700	-2.78576800
H	9.13212100	14.72483300	-3.16167600
O	8.84514200	11.89388600	-6.74637700
C	8.70717200	12.95315600	-7.65075300
H	9.58836600	13.61596400	-7.64979100
H	8.60134300	12.51141000	-8.64486900
H	7.81809000	13.56491300	-7.43526000

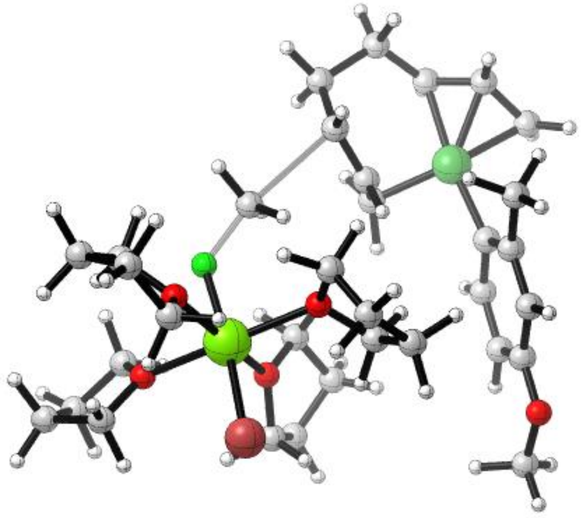

TS3-4-methoxy-2-methyphenyl

Ni	8.48663200	11.26300700	-0.31223100
C	7.15778500	9.93604700	0.24126700
C	7.60027500	10.68564100	1.35279600

C	7.60366700	12.09315100	1.32670600	C	13.81788500	17.20254800	-3.32730400
C	8.33608500	12.86309400	2.39103700	C	15.90790300	18.13641800	-3.88054900
C	9.51518400	13.75480700	1.91702600	H	16.87832700	16.32189500	-3.07291100
C	10.56571700	12.98765500	1.18076200	H	16.22990300	16.14221500	-4.71559400
C	10.28842100	12.52018700	-0.10080900	C	14.43132300	18.24350200	-4.24429100
C	9.24424200	12.95965500	-0.95897100	H	12.90741200	16.73108200	-3.71651800
C	9.17964500	10.28766700	-1.80583500	H	13.59642600	17.61074900	-2.33120500
C	8.93151400	10.63052700	-3.13938600	H	16.58082800	18.57069100	-4.62501800
C	9.59566400	10.06576400	-4.23627300	H	16.09669300	18.62965900	-2.91669100
C	10.55956000	9.09112500	-4.00544100	H	14.28148700	17.96624700	-5.29638000
C	10.80741600	8.68237300	-2.69560100	H	14.00752100	19.24007400	-4.09211000
C	10.15134900	9.27307600	-1.61717500	O	15.87436000	14.55610000	-0.88496000
H	6.27201800	10.25542200	-0.31454200	C	17.19347700	13.98023700	-0.79555600
H	7.32157900	8.86040700	0.22928800	C	15.81399400	15.76417500	-0.09679300
H	8.25717000	10.19889600	2.08259900	C	17.73361100	14.50955800	0.51307200
H	6.76472600	12.60910500	0.84651300	H	17.09133900	12.89266400	-0.85328900
H	8.75351600	12.14699100	3.11662700	H	17.79050700	14.30490900	-1.65885900
H	7.63176800	13.49396800	2.95468000	C	17.20578700	15.93909000	0.49030600
H	9.11709000	14.56312100	1.28049400	H	15.50330800	16.59009000	-0.74874900
H	9.93694300	14.24945700	2.80390000	H	15.04548300	15.62628900	0.67336900
H	11.27708600	12.41418700	1.78465400	H	18.82310100	14.44429300	0.57911000
H	11.03175700	11.85104200	-0.55334600	H	17.30225400	13.95275300	1.35548700
H	9.39348600	12.89012400	-2.03629800	H	17.82865000	16.55606600	-0.17105800
H	8.65587200	13.82406100	-0.64174900	H	17.18501100	16.41869700	1.47298100
H	9.34861700	10.39222100	-5.24489600	O	13.65701500	12.31249400	-1.77052600
H	11.54929400	7.89739800	-2.54097300	C	13.15097800	11.31200500	-2.69632800
C	12.20303700	14.45101900	-0.08037600	C	14.29969100	11.60250200	-0.70354400
H	12.41185100	14.97856400	0.84460800	C	13.95261500	10.03683000	-2.42495300
H	12.59622200	13.45495600	-0.18323700	H	12.07633900	11.16699700	-2.50489200
H	11.26474100	14.67246600	-0.57294500	H	13.29275300	11.71108600	-3.70567100
F	13.22885700	15.20230500	-1.14714500	C	15.00846900	10.46575100	-1.40488700
Mg	14.43207300	14.21593100	-2.44115400	H	14.94377600	12.31504000	-0.17463000
Br	16.12569200	13.15837500	-3.92568600	H	13.52717300	11.23388200	-0.00390100
O	14.82494200	16.18537700	-3.19116700	H	14.39187000	9.63140500	-3.34094800
C	16.07348800	16.63700400	-3.74707300	H	13.29052700	9.27223500	-2.00239000

H	15.90089700	10.85014500	-1.91520300
H	15.29980000	9.66041400	-0.72413200
O	12.83408000	14.21785400	-3.81988300
C	11.44229500	14.13056800	-3.45454000
C	12.98351800	14.20254900	-5.25258500
C	10.73797500	13.70331100	-4.72304400
H	11.33947400	13.42467200	-2.62522600
H	11.10836000	15.12004000	-3.10214300
C	11.57822600	14.40332000	-5.78787200
H	13.70801000	14.98260100	-5.51904500
H	13.40650300	13.23473400	-5.55676700
H	9.68122200	13.98609100	-4.72860200
H	10.79215500	12.60986500	-4.82886400
H	11.32791600	15.47280700	-5.82673400
H	11.45277800	13.98858700	-6.79210300
C	10.54832500	8.85061600	-0.22917800
H	11.07236400	9.66273900	0.30037400
H	9.67361200	8.60313600	0.38395700
H	11.21432100	7.97855600	-0.24030000
H	8.18567100	11.40017700	-3.35504200
O	11.31971900	8.48993000	-4.97454200
C	11.20517800	8.99816500	-6.27257100
H	11.45085500	10.07342300	-6.31243200
H	11.91676000	8.44867800	-6.89394900
H	10.19487600	8.86260000	-6.68784000

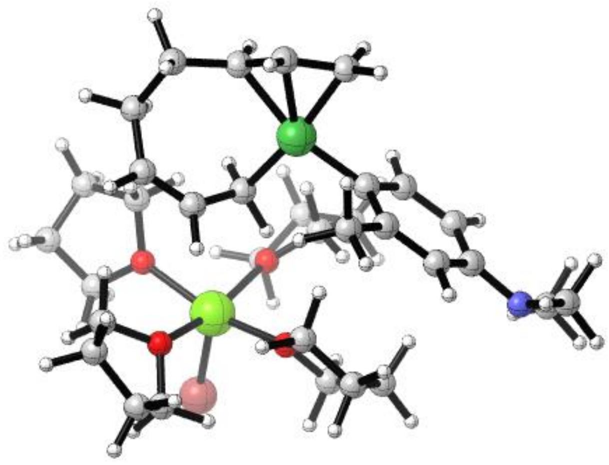

INT1-2-methy-4-dimethylaminophenyl

Ni	9.52448200	12.81471800	-0.69383400
C	7.59495300	12.90485000	-0.38624000
C	8.27387800	12.52536000	0.79060100
C	9.34921300	13.29163100	1.27454000
C	10.16055400	12.83396000	2.44642800
C	11.68410000	13.00321500	2.27327200
C	12.25297800	11.99992100	1.32173900
C	12.08820200	12.05983500	-0.02061500
C	11.46830600	13.12265000	-0.80315900
C	9.22590900	12.62943100	-2.56089600
C	9.06780700	13.72109900	-3.42105600
C	8.87730700	13.61389200	-4.80370100
C	8.81867800	12.35555900	-5.40498700
C	8.93427100	11.23878500	-4.56347400
C	9.12720500	11.36537300	-3.19028200
H	7.35418700	13.95595800	-0.57035400
H	6.88435300	12.20766300	-0.82667400
H	8.18802300	11.49242100	1.14636400
H	9.28914500	14.38393200	1.16737300
H	9.95323600	11.77099000	2.64635100
H	9.85563200	13.38484700	3.35070100
H	11.86533400	14.03958300	1.93767600
H	12.16931600	12.91145500	3.25514500
H	12.65635500	11.08350400	1.75817100

H	12.42352900	11.18311900	-0.59344600	C	15.37202100	11.41969100	-0.37866800
H	11.81083400	13.12314900	-1.84742500	C	15.47955500	9.50712100	-1.86140300
H	11.61124900	14.12121200	-0.36181200	H	15.28104300	10.92579400	-3.56807300
H	8.76462800	14.51990400	-5.39790400	H	16.76268000	11.12512900	-2.60331900
H	8.89489400	10.23872600	-5.00181900	C	15.33282000	9.89343800	-0.37468500
Mg	14.41805400	13.62097100	-2.49950400	H	16.38727700	11.79982500	-0.17612900
Br	16.43735100	14.13582400	-3.79884400	H	14.64704900	11.88625900	0.29821800
O	13.26470100	15.29747300	-2.88358800	H	16.30480900	8.81300300	-2.04458300
C	13.88465200	16.58442900	-3.09507400	H	14.56240200	9.03164100	-2.22715500
C	11.85683200	15.35757600	-3.22479000	H	16.12902400	9.47702300	0.24933400
C	12.72235800	17.54837400	-3.20747400	H	14.37824200	9.53715200	0.02777300
H	14.56938700	16.76737800	-2.25765900	O	13.34682400	12.56594800	-3.93549900
H	14.47975800	16.54008200	-4.01831600	C	12.47095200	11.41171600	-3.80292300
C	11.68776000	16.68753400	-3.92570600	C	13.47367900	12.92740700	-5.33279300
H	11.60707400	14.48028400	-3.83015300	C	12.28274200	10.90396600	-5.21536000
H	11.27249300	15.29835000	-2.29675000	H	12.94872900	10.70317700	-3.11444700
H	12.98870600	18.46287000	-3.74475100	H	11.51890600	11.74848700	-3.37137200
H	12.35649300	17.82733500	-2.21000200	C	12.33367000	12.20263300	-6.01158000
H	11.94074500	16.60068700	-4.99127800	H	13.44530600	14.02243000	-5.39603700
H	10.66337900	17.06362200	-3.84842100	H	14.45739800	12.59223600	-5.69222900
O	15.00526600	14.53182800	-0.71286600	H	11.32943300	10.37750400	-5.32716400
C	16.38729500	14.79591400	-0.38921100	H	13.10334900	10.23459200	-5.51040300
C	14.14240600	15.04204000	0.33196000	H	11.38709600	12.74369100	-5.87382900
C	16.37688800	15.03481600	1.10389700	H	12.50190700	12.05834000	-7.08248700
H	16.98223900	13.94181900	-0.72785200	C	9.27252500	10.12486800	-2.35480300
H	16.72078800	15.67998000	-0.95176200	H	10.22224900	10.13229400	-1.79892800
C	15.06384300	15.78933500	1.27750800	H	8.48144300	10.06684500	-1.59442300
H	13.37328400	15.67049200	-0.13539000	H	9.23316700	9.20632500	-2.95488400
H	13.65721100	14.18041800	0.81168900	H	9.08549900	14.73162100	-2.99926900
H	17.25388800	15.59099400	1.44651100	N	8.70223400	12.16709700	-6.81002900
H	16.34115800	14.07742200	1.64152700	C	8.75197500	13.36196200	-7.61678300
H	15.18497500	16.83338800	0.95911100	H	8.78279300	13.07876500	-8.67540300
H	14.68554600	15.78866100	2.30365400	H	7.87826600	14.02795000	-7.47279700
O	15.01343400	11.78155400	-1.71972400	H	9.65689000	13.93941400	-7.39490500
C	15.70036200	10.84016600	-2.55988400	C	7.61410100	11.30404400	-7.23356200

H	7.73857100	11.05077100	-8.29349600
H	7.60835200	10.37099000	-6.66610200
H	6.62441400	11.78287200	-7.10825100

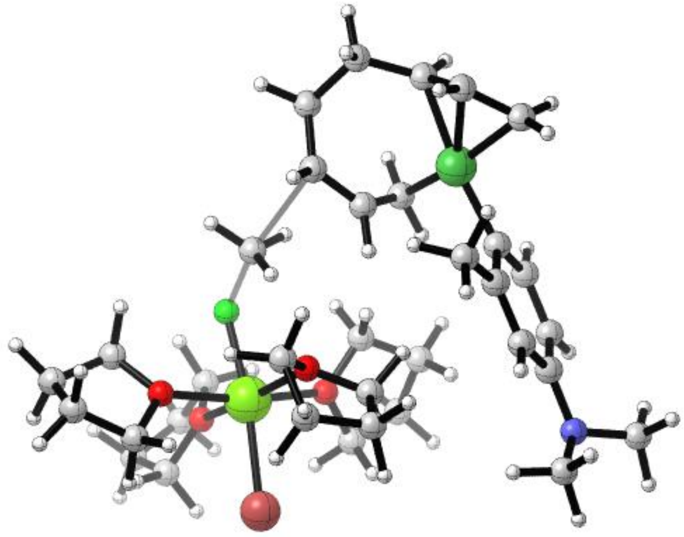

TS3-2-methy-4-dimethylaminophenyl

Ni	8.51044000	11.29767300	-0.31478700
C	7.25427200	9.90990700	0.25692300
C	7.69105400	10.66716700	1.36581100
C	7.62950100	12.07307300	1.35543100
C	8.34650000	12.86686300	2.41233000
C	9.48935100	13.79742000	1.92418000
C	10.54849000	13.06757500	1.16383300
C	10.26498300	12.60894700	-0.12078100
C	9.19706400	13.02563800	-0.95979800
C	9.16525600	10.35869800	-1.84572200
C	8.82337500	10.71027400	-3.16189500
C	9.38597500	10.12647700	-4.29182600
C	10.34259200	9.11344400	-4.16471300
C	10.70297300	8.73619300	-2.86786100
C	10.14229500	9.34764300	-1.73718900
H	6.33986000	10.19393000	-0.27101700
H	7.46603700	8.84298600	0.22863900
H	8.38781200	10.20287000	2.07309700
H	6.75893900	12.55416000	0.89575800
H	8.79577100	12.16594800	3.13371000
H	7.62749000	13.47355300	2.98378100
H	9.05696400	14.59707400	1.29946400
H	9.91135400	14.29907000	2.80702700
H	11.27828300	12.49999900	1.75153300
H	11.02438100	11.97458300	-0.59532500
H	9.33446500	12.97517500	-2.04010700
H	8.58289000	13.86425900	-0.62355300
H	9.09051400	10.46704200	-5.28501900
H	11.42886400	7.93631900	-2.71768500
C	12.18495900	14.53189800	-0.09378500
H	12.40105700	15.06024300	0.82906200
H	12.56026900	13.52870100	-0.19193500
H	11.25148900	14.76819200	-0.58870300
F	13.22734900	15.25694300	-1.16383000
Mg	14.42036400	14.20121700	-2.43001100
Br	16.10742300	13.08407800	-3.87965900
O	14.85841200	16.13981500	-3.20378300
C	16.11901500	16.55463600	-3.76023000
C	13.87993800	17.18519800	-3.33565800
C	16.00250400	18.06014800	-3.87172100
H	16.91427200	16.19802000	-3.09571500
H	16.25293200	16.06986400	-4.73740300
C	14.53007900	18.21983000	-4.23484600
H	12.96059900	16.74298500	-3.73892800
H	13.65945600	17.58202600	-2.33522700
H	16.68900800	18.48357900	-4.61006200
H	16.20674300	18.53323400	-2.90093800
H	14.37388600	17.96314800	-5.29124700
H	14.13825400	19.22717100	-4.06888600
O	15.84618000	14.51986100	-0.85771700
C	17.13910800	13.89315000	-0.72931600

C	15.80204700	15.72569700	-0.06620100
C	17.68178100	14.43422300	0.57395500
H	16.99228300	12.80862400	-0.75823600
H	17.76106300	14.16950200	-1.59160800
C	17.19643400	15.87768800	0.51725800
H	15.49941300	16.55647600	-0.71465400
H	15.03459500	15.59612500	0.70739800
H	18.76810500	14.33751900	0.65236800
H	17.22609100	13.90907500	1.42411800
H	17.83506600	16.46032300	-0.16032000
H	17.18947700	16.38147700	1.48794500
O	13.61099800	12.32760800	-1.71359800
C	13.15072000	11.30069200	-2.63321500
C	14.21105500	11.64100300	-0.60776600
C	14.03067500	10.07406300	-2.37547300
H	12.08923400	11.09254600	-2.42410400
H	13.25169100	11.70780600	-3.64335300
C	14.98190300	10.51288400	-1.25623000
H	14.81244500	12.37109200	-0.05306200
H	13.41340700	11.26407100	0.05607600
H	14.58240600	9.77873400	-3.27305500
H	13.40426400	9.22941500	-2.06317100
H	15.91073100	10.90619700	-1.68889100
H	15.22460900	9.70860000	-0.55556800
O	12.83441200	14.19375700	-3.81616000
C	11.43874100	14.08919200	-3.46622700
C	12.99836200	14.18291000	-5.24823800
C	10.74850100	13.68058300	-4.74909300
H	11.33066400	13.36412700	-2.65338400
H	11.09849400	15.07005100	-3.09716600
C	11.59999500	14.39415900	-5.79545200
H	13.72923600	14.96059100	-5.50375600
H	13.41811300	13.21417300	-5.55283000
H	9.69225300	13.96521600	-4.76152800
H	10.80145200	12.58929800	-4.87147700
H	11.35283600	15.46483300	-5.82221100
H	11.48402100	13.99216300	-6.80577800
C	10.61843700	8.90348900	-0.38012000
H	11.04810600	9.73937800	0.19339300
H	9.79166000	8.51648800	0.22961800
H	11.38048900	8.11572700	-0.44706300
H	8.07922300	11.49546800	-3.32051100
N	10.91834200	8.53670300	-5.32757800
C	12.03072500	7.64304300	-5.12121700
H	11.74809000	6.69002700	-4.63099800
H	12.47655800	7.39625800	-6.09219600
H	12.80063700	8.12495600	-4.50859800
C	9.97271700	7.97929800	-6.27659800
H	9.15330900	8.67599600	-6.46672900
H	10.47869700	7.78505500	-7.23058500
H	9.53300600	7.02769300	-5.92120300

Table 10

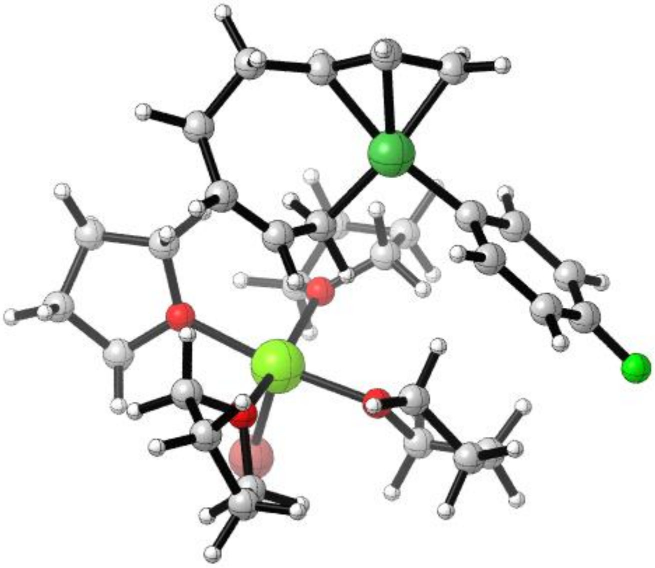

INT1-4-F-Ph

Ni	9.54102800	13.05045900	-0.72757700
C	7.63868500	13.47660300	-0.55156400
C	8.20447300	13.16948700	0.70146500
C	9.37583200	13.81357800	1.14625900
C	10.06057400	13.35596400	2.39833700
C	11.59234300	13.21314000	2.28107700
C	11.99246400	12.04722700	1.43293100
C	11.88670500	12.02996900	0.08291100
C	11.50712800	13.12514900	-0.79893500
C	9.31582700	12.46282600	-2.50886200
C	9.12818500	13.29311200	-3.62894400
C	9.04296500	12.81129400	-4.93728400
C	9.11396100	11.44427100	-5.14231600
C	9.24289100	10.56245400	-4.08302700
C	9.34540400	11.08222300	-2.79242800
H	7.58572000	14.51424300	-0.89635000
H	6.84159900	12.84251300	-0.93290000
H	7.93735500	12.22182100	1.18091000
H	9.49226400	14.88407100	0.92702100
H	9.64860900	12.38245400	2.70639500
H	9.84705900	14.05623100	3.22163000
H	11.97636800	14.16754400	1.88289200
H	12.01907400	13.11144800	3.28895000
H	12.19879300	11.10984300	1.95201700
H	12.05772200	11.06666000	-0.41646500
H	11.87559400	12.98010400	-1.82660900
H	11.79230500	14.11521200	-0.41748700
H	9.04014000	14.37384300	-3.48335200
H	8.91090100	13.47285700	-5.79221100
H	9.27505900	9.49239300	-4.28158400
H	9.47159800	10.37054800	-1.97272000
Mg	14.34979900	13.70018900	-2.48388300
Br	16.33941300	14.39573900	-3.76089500
O	13.15719900	15.37475800	-2.67841000
C	13.75183400	16.68693000	-2.78326000
C	11.76661100	15.41980100	-3.09487100
C	12.56644100	17.62594700	-2.81316600
H	14.43727400	16.80711200	-1.93495900
H	14.34305900	16.73360300	-3.70915000
C	11.57313600	16.81860600	-3.64327900
H	11.59281200	14.61432000	-3.81838700
H	11.13932400	15.21747400	-2.21601500
H	12.81276900	18.59958300	-3.24581000
H	12.17610700	17.78493000	-1.79899800
H	11.84959400	16.86190900	-4.70539600
H	10.53760400	17.15726500	-3.54710200
O	15.04407300	14.41921600	-0.64350200
C	16.45335500	14.52997400	-0.34344600
C	14.25845000	14.82681300	0.49894500
C	16.51148600	14.66074300	1.16391800
H	16.96025400	13.64907300	-0.75338800
H	16.84953800	15.41657900	-0.85670800
C	15.24887400	15.46452700	1.45079100
H	13.47174000	15.50368800	0.14418000
H	13.79112800	13.92860500	0.92650900
H	17.43072300	15.14563800	1.50419900

H	16.44726700	13.67294600	1.64025700
H	15.40640600	16.52175900	1.19834700
H	14.91332500	15.40776800	2.49032500
O	14.91885900	11.78540000	-1.87177100
C	15.72245800	10.97769100	-2.75039700
C	15.11640800	11.22929900	-0.56412600
C	15.53251900	9.54947700	-2.24360800
H	15.38357700	11.16598100	-3.77384000
H	16.76727300	11.31330500	-2.66889200
C	15.01997600	9.73044100	-0.79923100
H	16.11919900	11.52118700	-0.20770700
H	14.34672300	11.63348000	0.10450000
H	16.47279000	8.99176300	-2.28753400
H	14.80331800	9.00554500	-2.85352500
H	15.59710200	9.16225900	-0.06432700
H	13.97419800	9.41283700	-0.71747200
O	13.36972800	12.75274000	-4.05783000
C	12.50562300	11.58698600	-4.00855200
C	13.57444200	13.15664700	-5.43468200
C	12.53099300	11.04016000	-5.41811600
H	12.89783800	10.91585300	-3.23330600
H	11.49992000	11.90764500	-3.71424500
C	12.58717500	12.32896400	-6.23127700
H	13.43176900	14.24324000	-5.49230000
H	14.61836800	12.94217600	-5.70101900
H	11.64933500	10.43276600	-5.64158900
H	13.43333400	10.43511100	-5.59049700
H	11.59356200	12.79756200	-6.23710600
H	12.90898300	12.18481700	-7.26637200
F	9.09143200	10.96048500	-6.40438500

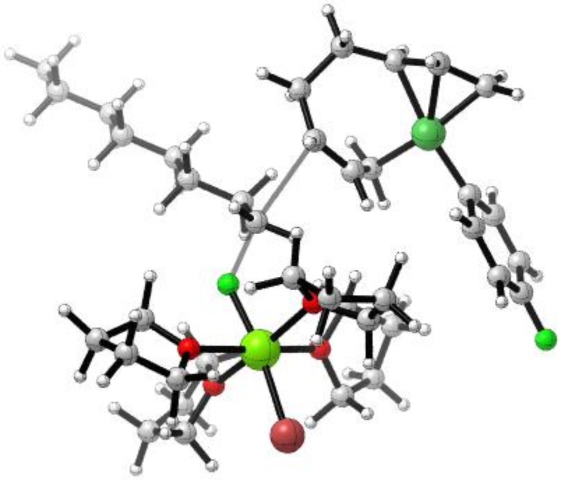

TS3-4-F-Ph

Ni	8.46054800	10.97048500	-0.51963300
C	7.35895500	9.41608800	-0.09102000
C	7.83268800	10.02685500	1.09134700
C	7.67062100	11.40659800	1.31375300
C	8.39951700	12.07760500	2.44411700
C	9.46315200	13.14167200	2.05268300
C	10.50371400	12.59841000	1.13159800
C	10.17497400	12.31734900	-0.19062900
C	9.05522900	12.80065600	-0.92338300
C	8.97865600	10.35312300	-2.24093900
C	8.27903000	10.62804500	-3.42964400
C	8.74480700	10.26259700	-4.69209700
C	9.96113800	9.60648100	-4.78788500
C	10.70191900	9.30119600	-3.65975400
C	10.19738300	9.67376700	-2.41115300
H	6.38129100	9.69371400	-0.49539300
H	7.65461700	8.39209500	-0.30719100
H	8.61035100	9.51675900	1.66977300
H	6.73865600	11.88252100	0.98852000
H	8.92274300	11.30633500	3.03093700
H	7.68124100	12.54853100	3.13186300
H	8.94599700	14.00325400	1.59422900
H	9.91466600	13.51880000	2.98167500

H	11.30064000	12.00501300	1.59124400	H	15.17148300	15.43665300	0.89136100
H	10.92847100	11.76593600	-0.76932800	H	18.97732500	14.71177600	0.64074600
H	9.16707400	12.94189100	-2.00043900	H	17.51167300	14.07071900	1.41153000
H	8.41010500	13.54110200	-0.44428200	H	17.77691700	16.76732800	-0.02186900
H	7.32493700	11.15666300	-3.37299000	H	17.22107500	16.51763100	1.64503700
H	8.18479700	10.48065400	-5.59941400	O	13.81033700	12.32807400	-1.51580100
H	11.64858000	8.77252800	-3.76599900	C	13.59455500	11.18602900	-2.39385000
H	10.78747100	9.40181200	-1.52950600	C	14.37366600	11.88081000	-0.27132200
C	12.05861500	14.36727800	-0.04048300	C	13.97497700	9.97047200	-1.56901600
H	12.61864000	14.16934800	0.87013600	H	12.54218400	11.17809700	-2.70671600
H	11.98591700	13.54347800	-0.72686300	H	14.24886400	11.32184500	-3.26363000
F	13.35991400	15.11199800	-0.94183200	C	14.99983400	10.54244300	-0.59523100
Mg	14.47536600	14.21117000	-2.28983100	H	15.07789700	12.65023100	0.07574800
Br	16.21390400	13.17748100	-3.75392700	H	13.56472400	11.78039400	0.47075900
O	14.69498600	16.16657400	-3.07598100	H	14.36868300	9.16144400	-2.19069800
C	15.91000400	16.74502700	-3.58085000	H	13.10101800	9.58670100	-1.02378700
C	13.61350100	17.10937400	-3.14516100	H	15.96253400	10.69239600	-1.10218500
C	15.62416900	18.23241800	-3.64009800	H	15.15891500	9.92221200	0.29146700
H	16.72364000	16.45261900	-2.90683200	O	12.86279300	14.01477800	-3.67023900
H	16.11940800	16.32273300	-4.57396300	C	11.47261500	13.76683000	-3.37429700
C	14.14340800	18.23956500	-4.00520200	C	13.07271600	14.00726800	-5.09953900
H	12.73868600	16.59046000	-3.55789600	C	10.85739400	13.33045100	-4.68654400
H	13.36711700	17.43943900	-2.12500000	H	11.39912300	13.00222800	-2.59341300
H	16.25866300	18.75606900	-4.36043200	H	11.02777800	14.69949100	-2.98971100
H	15.77334600	18.69304300	-2.65346300	C	11.68363400	14.12380000	-5.69204400
H	14.01681200	18.00015100	-5.06963600	H	13.75589300	14.83227300	-5.33579800
H	13.64274300	19.19166800	-3.80824300	H	13.56388900	13.06612700	-5.38091200
O	16.04003700	14.72448700	-0.84879400	H	9.78198400	13.52640900	-4.72766300
C	17.37176600	14.18163600	-0.74007600	H	10.99894300	12.24909200	-4.82757100
C	15.85668200	15.78626700	0.10484300	H	11.35869900	15.17386200	-5.71186000
C	17.88524200	14.69590700	0.58865700	H	11.63131400	13.73076300	-6.71106300
H	17.30586500	13.09192600	-0.81873000	C	11.06124000	15.46564500	-0.04475100
H	17.97164900	14.54018100	-1.58733600	H	11.10979700	15.96417100	-1.02548500
C	17.24286800	16.07603300	0.64466200	H	10.05196000	15.02916700	0.01033500
H	15.38029900	16.62915000	-0.40868000	C	11.28926100	16.47385700	1.06817100

H	11.30034400	15.94202900	2.03370200
H	12.29153100	16.92007300	0.95067400
C	10.23692600	17.56800300	1.10938500
H	10.20512300	18.08851600	0.13769100
H	9.24299300	17.10782100	1.23438500
C	10.47108800	18.57909700	2.21920200
H	11.46328300	19.04257300	2.08915000
H	10.51361400	18.05310500	3.18698300
C	9.41004100	19.66531400	2.28286400
H	8.41899000	19.20156800	2.41612700
H	9.36315200	20.19169500	1.31505700
C	9.64780900	20.67467700	3.39504800
H	10.63713500	21.13764600	3.25789900
H	9.69760000	20.14451100	4.35828200
C	8.57581500	21.74922700	3.45090000
H	7.58484100	21.30736600	3.61374000
H	8.75505200	22.46873700	4.25671000
H	8.53064500	22.30996300	2.50873900
F	10.43279700	9.26100400	-6.00294700

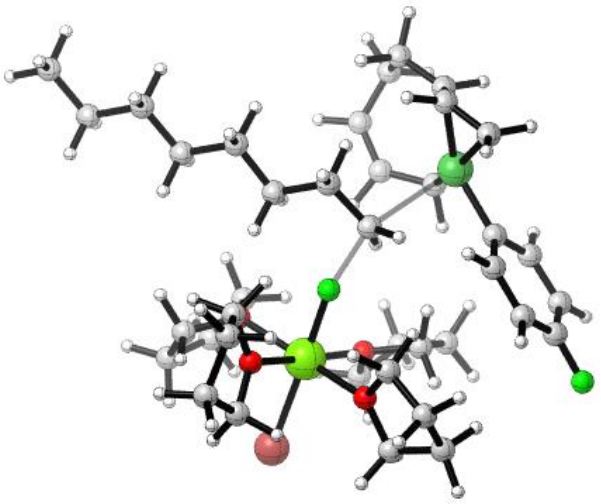

TS10-4-F-Ph

Ni	8.36539400	11.84675000	0.09148800
C	6.68587800	11.55615000	-0.92276000
C	6.41219200	11.56193000	0.45861200
C	6.88040200	12.61603100	1.25489600
C	6.69914100	12.67251000	2.73727100
C	7.83815500	13.39653200	3.47831800
C	9.08086100	12.57546100	3.61729500
C	9.90661100	12.27294400	2.60062200
C	9.78501800	12.65236700	1.18642900
C	9.41101400	11.57339900	-1.49192900
C	10.27051900	12.58847900	-1.95317000
C	11.05949300	12.46736400	-3.09890000
C	11.00245600	11.29007100	-3.82305500
C	10.14456300	10.26803800	-3.45753700
C	9.36062600	10.43440400	-2.31692800
H	6.60746800	12.48370200	-1.49486500
H	6.46268400	10.66176000	-1.50172600
H	6.11982000	10.63024300	0.95377100
H	6.92896900	13.60864300	0.78288000
H	6.57947600	11.65639100	3.14910300
H	5.75698800	13.19821000	2.95988800
H	8.04980900	14.33161700	2.93342100
H	7.48007000	13.69958900	4.47026100
H	9.30930600	12.18187400	4.60992600
H	10.75994300	11.62875400	2.85287600
H	10.75688700	12.52918000	0.68698700
H	9.49584400	13.71526700	1.09560700
H	11.72735500	13.26276000	-3.42328400
H	10.08760100	9.37045500	-4.07273400
C	9.45705100	9.64626300	0.90988400
H	10.35230900	10.23269800	1.06957400
F	10.49217400	8.06195700	0.79034900
Mg	12.32455000	7.46874500	0.84107600
Br	14.67448200	6.59896800	0.91636000
O	13.01787700	9.50670800	0.53397600
C	14.28401500	10.04376700	0.94562800

C	12.62631500	10.27576600	-0.62541200	H	11.01113500	2.48405400	-0.26184000
C	14.05460700	11.53419500	0.85199100	H	11.15809100	2.66573700	1.49323400
H	14.51714700	9.65599700	1.94063100	O	12.19120900	7.19101000	-1.26336500
H	15.06892200	9.70133100	0.25258400	C	10.92588200	7.04724800	-1.93343500
C	13.30296900	11.64359900	-0.47551700	C	13.27097400	7.16429900	-2.21541200
H	12.96669100	9.74990900	-1.52877500	C	11.27634300	6.69257300	-3.36660900
H	11.53344400	10.32751200	-0.65211500	H	10.34877900	6.27775200	-1.40596300
H	14.98013300	12.11650200	0.87823100	H	10.38530600	8.00073300	-1.86290400
H	13.41641900	11.86100300	1.68539800	C	12.60647900	7.41382900	-3.55376800
H	13.99750500	11.81781700	-1.30535000	H	14.00744900	7.91792100	-1.91438800
H	12.57204200	12.45784300	-0.48449800	H	13.75914700	6.18003000	-2.16909800
O	12.26069000	7.51954500	3.01325200	H	10.50238500	7.00623900	-4.07473900
C	12.56678000	6.37707100	3.82070300	H	11.41503200	5.60785400	-3.47471400
C	12.68981700	8.64374000	3.78266600	H	12.43675600	8.48829100	-3.71546000
C	13.90469000	6.68922900	4.48754000	H	13.19735100	7.03238700	-4.39136600
H	11.75802000	6.26290700	4.56284500	H	10.35376700	13.51825200	-1.38665500
H	12.58103800	5.50104200	3.16633300	H	9.14728300	9.47782100	-0.10968300
C	14.02816700	8.22131800	4.37480300	C	8.56937600	9.30157000	2.04160500
H	12.71748700	9.51459800	3.11591100	H	7.81848900	8.56976200	1.70781400
H	11.94270200	8.83603100	4.57070700	H	8.01979400	10.22073300	2.30410600
H	14.71843700	6.20429300	3.93986500	C	9.26632400	8.82687700	3.30777700
H	13.92219500	6.34104800	5.52498500	H	10.00113800	9.59158800	3.60540700
H	14.84983100	8.47320500	3.69490800	H	9.83559000	7.90997100	3.10714300
H	14.21779300	8.71132200	5.33461200	C	8.28496900	8.60136800	4.44394100
O	11.45683100	5.47482400	1.01008800	H	7.75826800	9.54319500	4.66968300
C	10.11836400	5.20254000	1.45162700	H	7.50670400	7.88890700	4.12245800
C	12.02725400	4.30671500	0.38702300	C	8.95277700	8.07734900	5.70374000
C	9.68500000	3.99805900	0.64207500	H	9.52256500	7.16338600	5.45829200
H	10.13373500	4.97250300	2.53064600	H	9.69820300	8.81090700	6.05568700
H	9.53496600	6.11272800	1.28944600	C	7.97427300	7.77254700	6.82599500
C	10.98769100	3.21035200	0.55549000	H	7.40105100	8.68189600	7.06967200
H	12.22056400	4.55447400	-0.66753400	H	7.23076900	7.03998700	6.47058700
H	12.99111700	4.08837500	0.85980800	C	8.64078000	7.24219700	8.08543600
H	9.35357700	4.30897800	-0.35829100	H	9.22375500	6.34273400	7.83391000
H	8.86715100	3.44395500	1.11189100	H	9.37222100	7.98099500	8.44675500

C	7.64285100	6.92060700	9.18449900
H	8.13228900	6.54142300	10.08769200
H	7.06767900	7.81128200	9.46682700
H	6.92374100	6.16143800	8.85200600
H	8.67193700	9.61894400	-2.07478400
F	11.79260100	11.12721900	-4.90179600

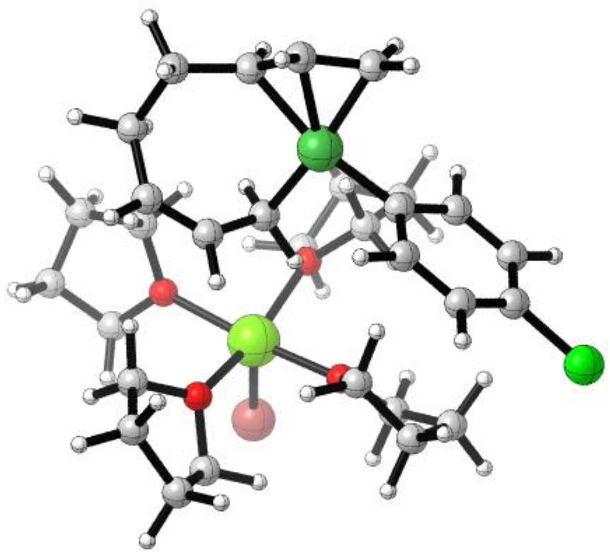

INT1-4-Cl-Ph

Ni	9.54042600	13.04214100	-0.73973900
C	7.63697700	13.46273300	-0.55768900
C	8.20418500	13.14433200	0.69189800
C	9.37427700	13.78668200	1.14165900
C	10.05995500	13.32087200	2.39011700
C	11.59258500	13.18880500	2.27405200
C	12.00024800	12.03101400	1.41857300
C	11.89519200	12.02135100	0.06887200
C	11.50614200	13.11968600	-0.80609600
C	9.30765900	12.47974900	-2.52468900
C	9.11790700	13.32642000	-3.63307200
C	9.00324300	12.86379900	-4.94394300
C	9.04926900	11.49553800	-5.17589800
C	9.18550600	10.60118000	-4.12232800
C	9.31592200	11.10295300	-2.82840300
H	7.58231600	14.50370200	-0.89191700
H	6.84021700	12.83146400	-0.94441800
H	7.93914200	12.19171200	1.16245300
H	9.48894000	14.85896700	0.93044200
H	9.65324100	12.34201600	2.68789900
H	9.84027200	14.01192600	3.21942900
H	11.97185100	14.14784300	1.88238400
H	12.01858700	13.08274100	3.28164300
H	12.21658000	11.09249100	1.93156200
H	12.07448600	11.06230500	-0.43552900
H	11.88025600	12.98581600	-1.83297800
H	11.78674200	14.10849900	-0.41755100
H	9.04539400	14.40641100	-3.47424300
H	8.86633400	13.55066400	-5.77805700
H	9.19922300	9.53039400	-4.31963600
H	9.44348100	10.37729200	-2.02118800
Mg	14.36087900	13.69769200	-2.48503800
Br	16.34363400	14.39200600	-3.76868800
O	13.16779700	15.37462600	-2.67566700
C	13.76518900	16.68556100	-2.78575700
C	11.77746400	15.42132800	-3.09137300
C	12.58192900	17.62693800	-2.82543200
H	14.44737200	16.80962500	-1.93538700
H	14.36047700	16.72524400	-3.70932800
C	11.58859400	16.81585500	-3.65175800
H	11.60031500	14.61002700	-3.80744000
H	11.14989900	15.22976500	-2.21021300
H	12.83164200	18.59675200	-3.26460700
H	12.18950200	17.79449500	-1.81342500
H	11.86789900	16.84960800	-4.71345900
H	10.55369600	17.15803300	-3.56103700
O	15.04812000	14.42310400	-0.64437700
C	16.45658100	14.54490300	-0.34476000

C	14.25914700	14.83398200	0.49435400
C	16.51318600	14.68549200	1.16174300
H	16.96906500	13.66478200	-0.74942700
H	16.84711900	15.43082200	-0.86356100
C	15.24540600	15.48323400	1.44293500
H	13.46911400	15.50417300	0.13420400
H	13.79561500	13.93620700	0.92712200
H	17.42906200	15.17841700	1.49947000
H	16.45504700	13.70032300	1.64423700
H	15.39661900	16.53978300	1.18384900
H	14.91005600	15.43123900	2.48273900
O	14.93650000	11.78769600	-1.86128300
C	15.74031500	10.97842100	-2.73860900
C	15.13731400	11.23834900	-0.55140100
C	15.56081400	9.55213700	-2.22247600
H	15.39471100	11.15764000	-3.76148000
H	16.78355400	11.32063300	-2.66507600
C	15.04757500	9.73807400	-0.77911500
H	16.13855000	11.53678300	-0.19599900
H	14.36515100	11.64168500	0.11479100
H	16.50520000	9.00119900	-2.26320000
H	14.83571500	8.99879100	-2.82879100
H	15.62718400	9.17606400	-0.04143700
H	14.00318500	9.41621900	-0.69547200
O	13.36603900	12.74063000	-4.03975600
C	12.51310200	11.56699300	-3.97821900
C	13.55945100	13.13556300	-5.42185800
C	12.52864400	11.01479700	-5.38508100
H	12.91921800	10.90209500	-3.20534100
H	11.50629000	11.87921000	-3.67707900
C	12.56670400	12.30076400	-6.20358200
H	13.41494100	14.22164600	-5.48473900
H	14.60137800	12.92019600	-5.69526300
H	11.64875500	10.39892300	-5.59478500
H	13.43367400	10.41570700	-5.56416000
H	11.57036500	12.76372200	-6.19795600
H	12.87400500	12.15417900	-7.24265600
Cl	8.94904200	10.88570600	-6.83058200

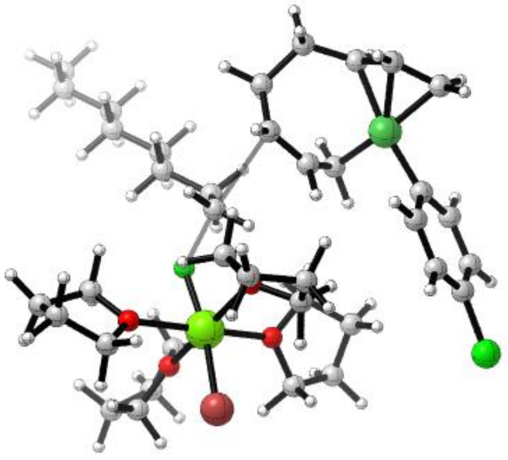

TS3-4-Cl-Ph

Ni	8.41651900	10.97226000	-0.49156200
C	7.28484100	9.44447300	-0.04561400
C	7.78185700	10.05147500	1.12925600
C	7.65242600	11.43590200	1.34423300
C	8.40142900	12.09757000	2.46683200
C	9.46992900	13.15156800	2.06384300
C	10.50323300	12.59166400	1.14464100
C	10.17068600	12.30086500	-0.17310300
C	9.05252000	12.78560200	-0.90827600
C	8.91765700	10.34975900	-2.21095700
C	8.24334600	10.66324100	-3.40494400
C	8.73688800	10.34266400	-4.66753500
C	9.95832200	9.68874700	-4.76413500
C	10.66308000	9.32989300	-3.62469600
C	10.13038000	9.65788800	-2.37709900
H	6.30948800	9.74003600	-0.44254700
H	7.55592800	8.41269300	-0.25696000
H	8.55262700	9.52756300	1.70449800

H	6.72890500	11.93055700	1.02268800	C	17.87758800	14.72395400	0.60501500
H	8.92315800	11.32063700	3.04736400	H	17.31579500	13.11154100	-0.80035300
H	7.69537200	12.57537000	3.16236300	H	17.97646900	14.56132500	-1.56958900
H	8.95614600	14.01190200	1.59952200	C	17.22577800	16.10000200	0.65281900
H	9.92740600	13.53402300	2.98757000	H	15.36423400	16.63650500	-0.41146200
H	11.30167900	12.00289100	1.60696700	H	15.15820800	15.44660500	0.89164600
H	10.91706700	11.73406500	-0.74591000	H	18.96926400	14.74723100	0.66259100
H	9.17093500	12.92112300	-1.98567300	H	17.50391900	14.09923700	1.42817500
H	8.41948100	13.54089500	-0.43604100	H	17.75845900	16.79249200	-0.01359000
H	7.29267200	11.19825700	-3.35328600	H	17.19595500	16.54509600	1.65144300
H	8.19166900	10.60824700	-5.57145000	O	13.79898900	12.32540700	-1.52301400
H	11.61239900	8.80246200	-3.71432800	C	13.57699500	11.18417500	-2.39969700
H	10.69689500	9.34973500	-1.49278800	C	14.39433000	11.87856200	-0.29383600
C	12.04888400	14.34560200	-0.04714000	C	14.03857400	9.97261400	-1.60828500
H	12.61670400	14.14594100	0.85814700	H	12.51134600	11.14623200	-2.66234400
H	11.97942500	13.52745700	-0.74106400	H	14.18059700	11.34666800	-3.30044400
F	13.35565300	15.10137600	-0.95311300	C	15.06679100	10.57293600	-0.65504300
Mg	14.48118800	14.20740000	-2.29099100	H	15.07387900	12.66805100	0.05740400
Br	16.23535200	13.15345700	-3.72049600	H	13.59851900	11.73788900	0.45657000
O	14.70458800	16.15703700	-3.08520700	H	14.44951700	9.19413200	-2.25715200
C	15.92159000	16.73585200	-3.58436400	H	13.20351400	9.53858300	-1.04128400
C	13.62131900	17.09723200	-3.16501700	H	16.00481700	10.77536800	-1.18988000
C	15.63501400	18.22296900	-3.64588300	H	15.27922500	9.94603500	0.21569200
H	16.73211500	16.44485100	-2.90611800	O	12.88210500	14.00494900	-3.68823100
H	16.13642000	16.31290000	-4.57607700	C	11.48828000	13.75765400	-3.41088200
C	14.15639500	18.22930200	-4.01967100	C	13.11540900	13.97316700	-5.11426700
H	12.75219100	16.57621300	-3.58681200	C	10.89256700	13.30760900	-4.72811800
H	13.36375600	17.42524000	-2.14697200	H	11.40488800	12.99972300	-2.62430300
H	16.27324200	18.74687900	-4.36272400	H	11.03646700	14.69278200	-3.04016900
H	15.77849900	18.68391800	-2.65855700	C	11.73708900	14.08407500	-5.73121700
H	14.03631100	17.99206300	-5.08529300	H	13.80435200	14.79279600	-5.35229100
H	13.65395200	19.18074100	-3.82403400	H	13.60871100	13.02593800	-5.37091700
O	16.03969700	14.73544300	-0.84218900	H	9.81915000	13.50897900	-4.79020400
C	17.37424400	14.20174700	-0.72442500	H	11.03236600	12.22427800	-4.85546000
C	15.84428400	15.79878600	0.10706500	H	11.41578400	15.13463500	-5.77284300

H	11.69973500	13.67340200	-6.74390100
C	11.05616900	15.44735800	-0.05107900
H	11.10447300	15.94141800	-1.03417500
H	10.04549700	15.01475600	0.00731800
C	11.28758200	16.45829700	1.05861200
H	11.30984900	15.92837000	2.02508900
H	12.28576200	16.91074200	0.93277800
C	10.22857800	17.54575100	1.10714200
H	10.18704300	18.06650500	0.13598000
H	9.23837200	17.07924400	1.23841700
C	10.46359400	18.55744100	2.21622800
H	11.45240700	19.02653800	2.08078000
H	10.51476100	18.03096000	3.18334900
C	9.39687100	19.63759700	2.28709100
H	8.40900900	19.16819300	2.42422100
H	9.34243200	20.16552600	1.32054000
C	9.63445900	20.64603100	3.40017200
H	10.62093200	21.11406000	3.25970700
H	9.69096400	20.11428800	4.36217200
C	8.55751000	21.71522100	3.46266200
H	7.56936000	21.26832000	3.62889000
H	8.73674200	22.43405300	4.26906500
H	8.50564100	22.27747700	2.52176600
Cl	10.63814400	9.34030200	-6.35593700

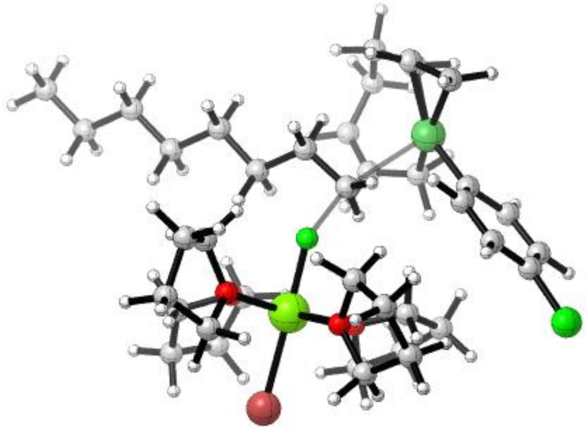

TS10-4-Cl-Ph

Ni	8.36320000	11.88123300	0.10014200
C	6.71306000	11.58131200	-0.95851600
C	6.40761100	11.55262600	0.41628000
C	6.83560200	12.59921700	1.24369600
C	6.62147400	12.62581000	2.72248500
C	7.72899400	13.36130800	3.49953800
C	8.98581000	12.56491600	3.65575300
C	9.84131200	12.29663100	2.65419400
C	9.74049300	12.69600300	1.24409900
C	9.44719800	11.66969400	-1.46254400
C	10.27902400	12.72612100	-1.87844000
C	11.10896300	12.65637700	-2.99693600
C	11.13132100	11.48811400	-3.74451300
C	10.29182400	10.43048900	-3.42501500
C	9.46111900	10.54842700	-2.31252500
H	6.62599800	12.51869000	-1.51309600
H	6.52083000	10.69471800	-1.56013700
H	6.12232600	10.60553200	0.88536800
H	6.87757900	13.60106000	0.79099800
H	6.51321700	11.60109200	3.11534400
H	5.66405800	13.12862800	2.93225900
H	7.93381000	14.30810600	2.97252100
H	7.34230000	13.64264600	4.48705500
H	9.20046600	12.16190900	4.64762600
H	10.70469300	11.66999900	2.91538400

H	10.72683600	12.61166200	0.76512400	O	11.42598100	5.51243800	1.04114400
H	9.42198000	13.75166000	1.16499400	C	10.09524900	5.25984200	1.52002900
H	11.75494600	13.48848400	-3.27053300	C	11.98808800	4.32149400	0.45569900
H	10.28255200	9.53490000	-4.04614700	C	9.68302900	3.96212500	0.85383800
C	9.46809100	9.69247200	0.89906900	H	10.12540500	5.15860100	2.61824200
H	10.36671100	10.27268800	1.06883200	H	9.48658600	6.13250700	1.26742000
F	10.48913400	8.09738500	0.76619500	C	11.00981600	3.21403400	0.79885900
Mg	12.31275600	7.47739700	0.82562600	H	12.07754700	4.49187200	-0.62880700
Br	14.65411100	6.57644900	0.88343700	H	12.99810300	4.18113800	0.85612300
O	13.02211600	9.51499400	0.53030700	H	9.31116600	4.15582000	-0.16157200
C	14.33651700	9.99214900	0.85878400	H	8.90020400	3.43330700	1.40524100
C	12.64513700	10.23938300	-0.66079200	H	11.02824300	2.39754600	0.07147800
C	14.19676700	11.48931500	0.70112900	H	11.24579200	2.79329400	1.78542300
H	14.59025500	9.64243400	1.86291600	O	12.14858600	7.15103300	-1.27825000
H	15.06305600	9.55971900	0.15300200	C	10.87037600	7.02736000	-1.92760400
C	13.34380100	11.60046200	-0.56679000	C	13.21381600	7.04208600	-2.24163500
H	12.98555000	9.67145300	-1.53992100	C	11.18713800	6.61405300	-3.35287000
H	11.55317400	10.30130400	-0.69271400	H	10.27406600	6.29268000	-1.37307300
H	15.15988500	12.00221500	0.62524300	H	10.36127600	8.00005500	-1.87793800
H	13.65714500	11.89983400	1.56566300	C	12.54186000	7.27481500	-3.57837800
H	13.96056600	11.77125400	-1.45659900	H	13.98706500	7.77438400	-1.98319300
H	12.61942000	12.41908000	-0.51453300	H	13.65969400	6.03974000	-2.16429500
O	12.26760500	7.54775800	2.99808900	H	10.41671700	6.93466600	-4.06123800
C	12.59495000	6.42250600	3.82085500	H	11.28196200	5.52184200	-3.42660300
C	12.69754800	8.68869300	3.74345500	H	12.41364500	8.35129000	-3.76261800
C	13.94603600	6.75242600	4.44947300	H	13.10582400	6.84956700	-4.41314000
H	11.80392800	6.32129300	4.58381200	H	10.31115700	13.64636900	-1.29167000
H	12.59603400	5.53285100	3.18469800	H	9.16214500	9.53728800	-0.12399700
C	14.04647500	8.28569300	4.32728900	C	8.57447300	9.33127900	2.02131200
H	12.71024300	9.54932400	3.06231600	H	7.83189900	8.59780000	1.67279300
H	11.95762100	8.88705700	4.53693800	H	8.01598100	10.24267200	2.29096200
H	14.74901200	6.27651800	3.87775600	C	9.26467000	8.84529100	3.28642400
H	13.99802300	6.40714000	5.48667200	H	9.98344300	9.61559100	3.60783700
H	14.85962300	8.54349300	3.63984800	H	9.85046900	7.94188600	3.07569000
H	14.23779200	8.78411200	5.28240000	C	8.27678200	8.57873200	4.40760300

H	7.73080600	9.50526700	4.65057800
H	7.51439100	7.85954900	4.06353300
C	8.94656500	8.03725400	5.65889800
H	9.52608100	7.13428100	5.39541000
H	9.68507700	8.77079900	6.02534100
C	7.97050300	7.70103700	6.77407900
H	7.38937200	8.60025500	7.03586900
H	7.23331600	6.96932300	6.40404400
C	8.64191800	7.15164100	8.02275200
H	9.23049500	6.26087800	7.75392300
H	9.36906600	7.88845400	8.39662000
C	7.64757100	6.80360200	9.11698800
H	8.14060900	6.41127900	10.01255700
H	7.06725500	7.68544500	9.41600000
H	6.93268500	6.04611000	8.77177700
H	8.79386400	9.70456300	-2.11059400
Cl	12.25256700	11.33276100	-5.09362300

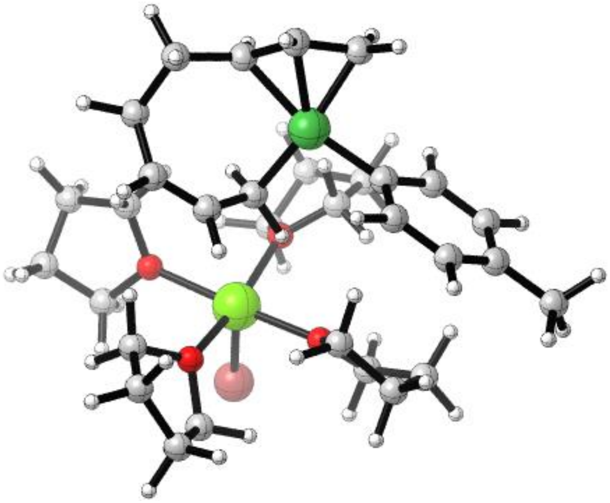

INT1-4-Me-Ph

Ni	9.59682600	13.10349100	-0.81754100
C	7.71753900	13.62975600	-0.66709500
C	8.23704600	13.25379700	0.58655500
C	9.42982900	13.82415000	1.07329900
C	10.06789600	13.29529000	2.32138300
C	11.59963000	13.14220200	2.23386200
C	12.01098600	11.98967500	1.37261400
C	11.93337200	11.99261100	0.02138700
C	11.57209100	13.10074500	-0.85434000
C	9.33177200	12.53060300	-2.59977100
C	9.12669100	13.35026600	-3.72542600
C	8.94683100	12.84744500	-5.01209700
C	8.93183600	11.47092900	-5.25339700
C	9.09711700	10.63083000	-4.15178800
C	9.29231900	11.14910200	-2.87158600
H	7.73282400	14.67883900	-0.97996100
H	6.89727200	13.05337700	-1.08854700
H	7.90998800	12.30742400	1.02989200
H	9.60100600	14.89499800	0.89310600
H	9.63448300	12.31344500	2.56697000
H	9.83941300	13.95682600	3.17248200
H	11.99536600	14.10013300	1.85752300
H	12.00776300	13.02052400	3.24714700
H	12.20772400	11.04359500	1.87968800
H	12.11087000	11.03377300	-0.48418000
H	11.93288200	12.95782000	-1.88341500
H	11.87116000	14.08583500	-0.46954700
H	9.09028800	14.43657500	-3.59467200
H	8.80029700	13.53379500	-5.85024200
H	9.07684300	9.54899300	-4.30486800
H	9.43785800	10.43831800	-2.05300100
Mg	14.26646900	13.67094500	-2.39680100
Br	16.20912300	14.42181500	-3.74001500
O	13.09469000	15.35773200	-2.59913600
C	13.69164000	16.67068800	-2.67273300
C	11.72905600	15.39945700	-3.08876600
C	12.50812300	17.60806600	-2.76485600
H	14.32977300	16.79237000	-1.78825900

H	14.33403100	16.71544900	-3.56388800
C	11.56039500	16.79846400	-3.64447500
H	11.59709500	14.59572000	-3.82325300
H	11.05794700	15.18688600	-2.24461100
H	12.77574700	18.58125900	-3.18586800
H	12.06568500	17.76818600	-1.77258000
H	11.89212600	16.84256100	-4.69075900
H	10.52042700	17.13466900	-3.60235200
O	15.02390300	14.40804800	-0.57530500
C	16.44432400	14.57536400	-0.35419600
C	14.29216500	14.80768400	0.60492900
C	16.57927800	14.74300500	1.14370500
H	16.96078000	13.70481000	-0.77276800
H	16.77858900	15.46426100	-0.90553700
C	15.30833200	15.51333600	1.47754300
H	13.45080400	15.43759300	0.29078600
H	13.89695200	13.90055400	1.08303900
H	17.49942800	15.26407300	1.42251200
H	16.57134700	13.76497400	1.64385200
H	15.41269200	16.56525600	1.17866600
H	15.03479200	15.48409200	2.53592100
O	14.96855600	11.77760800	-1.81855800
C	15.77271200	10.99654000	-2.71948000
C	15.19301600	11.22084100	-0.51657700
C	15.68000700	9.56378600	-2.19082400
H	15.37561200	11.15135200	-3.72810300
H	16.80169200	11.38453800	-2.69267300
C	15.12777500	9.72342000	-0.76057900
H	16.19176300	11.53401400	-0.16545600
H	14.41800200	11.60189400	0.15933200
H	16.66308300	9.08316100	-2.20321100
H	15.01300000	8.95101700	-2.80623800
H	15.69319800	9.15732300	-0.01501100
H	14.08365300	9.39282400	-0.70791100
O	13.32671800	12.68560900	-3.98641700
C	12.53467900	11.46928200	-3.93132000
C	13.48202900	13.10899700	-5.36254200
C	12.48696200	10.97447500	-5.35969300
H	13.01758500	10.79058800	-3.21720000
H	11.53306600	11.71804400	-3.56089600
C	12.46409300	12.29128900	-6.12804500
H	13.33400000	14.19567800	-5.39925500
H	14.51468600	12.90018200	-5.67441200
H	11.60226300	10.35447300	-5.53626000
H	13.38751100	10.39487000	-5.61074000
H	11.46398700	12.74073600	-6.04459600
H	12.72142200	12.19217200	-7.18658700
C	8.71992500	10.92436000	-6.63535800
H	7.67612600	11.02451900	-6.96167100
H	9.33348500	11.44778500	-7.38133500
H	8.97235300	9.85814300	-6.68623300

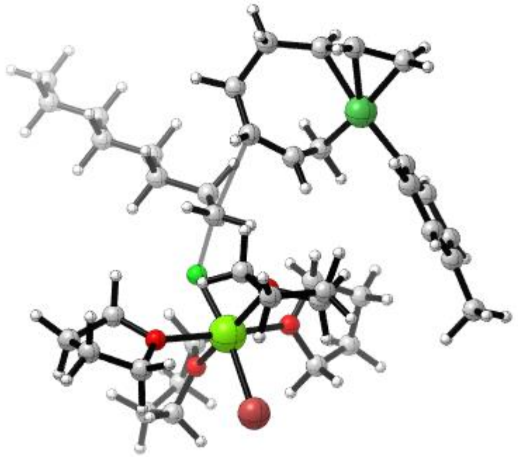

TS3-4-Me-Ph

Ni	8.42933400	10.94632000	-0.38213100
C	7.32872800	9.41168700	0.11738700
C	7.82217700	10.06101100	1.26982600
C	7.66543800	11.44983100	1.44180700
C	8.41420300	12.15857700	2.53688500

C	9.46587200	13.21327900	2.09431800	C	15.68517600	18.24845200	-3.60059000
C	10.49683300	12.64752400	1.17534900	H	16.70481700	16.40635600	-2.91077000
C	10.15150600	12.32109500	-0.13052000	H	16.10928500	16.34521100	-4.58451900
C	9.00964100	12.76638700	-0.85377900	C	14.20684900	18.32803500	-3.96696600
C	9.04460800	10.21976100	-2.02749900	H	12.73057900	16.73117500	-3.56377100
C	8.78871700	10.73636000	-3.31025100	H	13.39893500	17.50853100	-2.10819600
C	9.47451000	10.31589300	-4.44729800	H	16.34318600	18.76459400	-4.30510400
C	10.48745900	9.35727000	-4.36679500	H	15.85081200	18.67513600	-2.60142300
C	10.72973500	8.79206800	-3.11519700	H	14.07312800	18.12168900	-5.03762600
C	10.02213800	9.20856500	-1.98784900	H	13.74595900	19.29471500	-3.74562500
H	6.34473600	9.67592600	-0.27882000	O	15.90473300	14.59611000	-0.92048700
H	7.62133300	8.38083600	-0.07183600	C	17.20043100	13.97366700	-0.79705400
H	8.60941500	9.57015400	1.85263000	C	15.75965800	15.64258400	0.05765700
H	6.72337200	11.91074200	1.12427100	C	17.71913700	14.43578300	0.54902200
H	8.95139300	11.40783300	3.13770100	H	17.07242600	12.88989200	-0.88773300
H	7.70739600	12.64839400	3.22359400	H	17.83282600	14.30522700	-1.63144400
H	8.93568900	14.05674400	1.61733500	C	17.14962800	15.84621800	0.62501100
H	9.92952700	13.62327900	3.00342000	H	15.33755000	16.52274600	-0.44027000
H	11.31401200	12.08776000	1.64064800	H	15.04258800	15.31043500	0.82286100
H	10.89610800	11.75673700	-0.70797600	H	18.80955900	14.39192500	0.61755400
H	9.10484800	12.87565100	-1.93612900	H	17.29989800	13.81509800	1.35284400
H	8.37415200	13.52247400	-0.38637100	H	17.73042700	16.52239400	-0.01750200
H	8.02117600	11.50522500	-3.42964400	H	17.13449700	16.26669700	1.63456300
H	9.22823400	10.74714400	-5.42140200	O	13.57799900	12.33395100	-1.76465400
H	11.49071800	8.01290200	-3.02392400	C	13.26457900	11.26877200	-2.70884500
H	10.25198600	8.72557100	-1.03265400	C	14.15071900	11.75830100	-0.57964100
C	11.99008000	14.38606600	-0.10928200	C	13.76722700	9.98778400	-2.06060600
H	12.58974400	14.16942700	0.77098300	H	12.17863200	11.24532600	-2.88048700
H	11.86979500	13.57529100	-0.80699500	H	13.79501100	11.50498800	-3.63895700
F	13.25616400	15.13376200	-1.04833000	C	14.80993000	10.48699900	-1.06648300
Mg	14.35798100	14.22603800	-2.40294000	H	14.83182300	12.50246500	-0.14384800
Br	16.05449600	13.18662700	-3.90861800	H	13.34372800	11.55312800	0.14359300
O	14.66704900	16.20617200	-3.10248400	H	14.16534800	9.28848300	-2.80227700
C	15.90860600	16.74924500	-3.58198300	H	12.94317100	9.48806300	-1.53499400
C	13.62842100	17.19790700	-3.13863200	H	15.74915800	10.73009300	-1.58154800

H	15.01653000	9.77779800	-0.25958900
O	12.73482900	14.19918100	-3.78763300
C	11.33373600	13.99423200	-3.51332200
C	12.95961900	14.24932600	-5.21215700
C	10.72199800	13.65121900	-4.85454500
H	11.21922100	13.19800400	-2.76802500
H	10.92138700	14.92604000	-3.09252500
C	11.58478400	14.47093600	-5.80576700
H	13.69395600	15.04141800	-5.40293200
H	13.39689800	13.29459800	-5.53668500
H	9.65220100	13.87639700	-4.89691400
H	10.84722000	12.57650700	-5.04980900
H	11.30896800	15.53416500	-5.75941700
H	11.52302500	14.14651500	-6.84829300
C	10.99944800	15.48929000	-0.06342700
H	11.02594700	16.00254700	-1.03783400
H	9.99033900	15.05543400	0.01103200
C	11.25740900	16.48444800	1.05444200
H	11.28823600	15.94511900	2.01534100
H	12.25772900	16.92922400	0.91687000
C	10.20818100	17.57994700	1.12257400
H	10.15705300	18.10229000	0.15257300
H	9.21667300	17.11985300	1.26590600
C	10.46291100	18.59054400	2.22839700
H	11.45486100	19.05036200	2.08394600
H	10.51663500	18.06595700	3.19633300
C	9.40654000	19.68048800	2.30314400
H	8.41561900	19.22016700	2.44875200
H	9.34998100	20.20461000	1.33457600
C	9.65970200	20.69236700	3.40968900
H	10.64962200	21.15070000	3.26147000
H	9.71654800	20.16535200	4.37424600
C	8.59290300	21.77169900	3.47192400
H	7.60151400	21.33447500	3.64443300
H	8.78216900	22.49263200	4.27417200
H	8.54200000	22.32998600	2.52858700
C	11.31052300	8.98992300	-5.56622700
H	11.78149800	8.00705100	-5.44822500
H	10.70950300	8.96580600	-6.48340300
H	12.12159900	9.71492200	-5.73841200

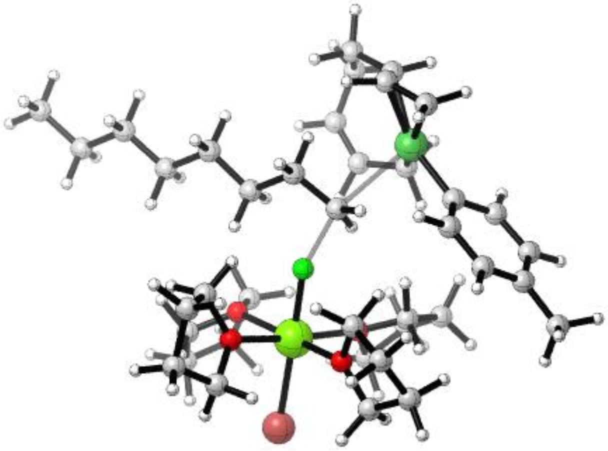

TS10-4-Me-Ph

Ni	8.36161200	11.86417600	0.10515700
C	6.70571100	11.54508700	-0.93609600
C	6.41248300	11.52394000	0.44176300
C	6.84007600	12.57784700	1.26046000
C	6.64086000	12.60603100	2.74146300
C	7.75772200	13.33876200	3.50836500
C	9.01562700	12.54163000	3.65122200
C	9.86196900	12.27969800	2.63989600
C	9.74706500	12.68613300	1.23344300
C	9.43168400	11.66112900	-1.46867900
C	10.23705100	12.73429400	-1.89497900
C	11.02901700	12.68320400	-3.04008400
C	11.08076500	11.53703900	-3.83415700
C	10.25631500	10.47403600	-3.46608400
C	9.44977800	10.55098400	-2.33139100
H	6.60821200	12.47777200	-1.49678500

H	6.51488000	10.65275200	-1.52965800	H	11.80927200	6.28741600	4.54459700
H	6.13835600	10.57752800	0.91935000	H	12.66814200	5.53324900	3.16572100
H	6.86943600	13.57857100	0.80444900	C	14.05073500	8.29583900	4.33346300
H	6.53525500	11.58113900	3.13470700	H	12.71100100	9.54732000	3.06361900
H	5.68669700	13.11053300	2.96245800	H	11.95507700	8.87091000	4.52976200
H	7.95862000	14.28548300	2.97954300	H	14.77539600	6.27609100	3.97161800
H	7.38124900	13.62002500	4.50004500	H	13.95030900	6.44977900	5.53664700
H	9.23832400	12.13341800	4.63936400	H	14.86309000	8.53778700	3.63882500
H	10.72924700	11.65310900	2.89012500	H	14.24378600	8.81441000	5.27738800
H	10.72870400	12.60778900	0.74443300	O	11.44896100	5.49818700	1.04468300
H	9.42373700	13.74084800	1.16308100	C	10.11955100	5.25016700	1.52774800
H	11.64038600	13.54604500	-3.31285200	C	11.99970200	4.30538000	0.45216700
H	10.23501700	9.57449700	-4.08880100	C	9.67789100	3.99278600	0.80741200
C	9.48533500	9.65436400	0.88723300	H	10.15748100	5.09709300	2.61985200
H	10.37269500	10.25347000	1.05159400	H	9.52644100	6.14423500	1.31803500
F	10.50660800	8.08003100	0.76687300	C	10.98498500	3.21200500	0.73652300
Mg	12.33866500	7.46814200	0.81960900	H	12.12821800	4.49640700	-0.62418100
Br	14.68675400	6.59683500	0.87955400	H	12.99178800	4.12535800	0.88110000
O	13.01982900	9.49428100	0.47217000	H	9.32304800	4.23850900	-0.20285400
C	14.27983000	10.05224300	0.87207100	H	8.87544500	3.46370400	1.32961400
C	12.62477600	10.22906900	-0.70828700	H	10.99131200	2.42747300	-0.02540900
C	14.03552800	11.53778200	0.74330300	H	11.19398400	2.74192600	1.70649600
H	14.51449000	9.68982700	1.87638200	O	12.15821900	7.15293300	-1.27435200
H	15.06884200	9.70120300	0.18766900	C	10.86839700	7.11465900	-1.91378800
C	13.28211800	11.60898200	-0.58634900	C	13.20835900	7.02244700	-2.25077600
H	12.97795400	9.68531300	-1.59746700	C	11.14502100	6.61575800	-3.31671500
H	11.53114600	10.26664800	-0.74315800	H	10.21469200	6.46921000	-1.31658100
H	14.95512900	12.12994700	0.75608800	H	10.44971500	8.13088500	-1.91617900
H	13.39390500	11.87689300	1.56939400	C	12.51905200	7.21778700	-3.58729300
H	13.97325300	11.77625600	-1.42111300	H	13.98548000	7.76248000	-2.02609600
H	12.53748000	12.40992600	-0.61541200	H	13.65741900	6.02388700	-2.15223300
O	12.29590100	7.54085600	2.98800000	H	10.37817100	6.93394700	-4.03013800
C	12.62324200	6.41522000	3.81050400	H	11.19889700	5.51843500	-3.33646200
C	12.70316700	8.68436300	3.74133700	H	12.41743200	8.28906900	-3.81376500
C	13.94593700	6.76657100	4.48899700	H	13.05761800	6.74138100	-4.41136500

H	10.26600400	13.65054700	-1.30070800
H	9.16919500	9.51030900	-0.13502200
C	8.59307200	9.29566700	2.01240300
H	7.86086400	8.54878000	1.67001000
H	8.02132400	10.20251500	2.26781800
C	9.28435600	8.83316900	3.28531100
H	9.99143100	9.61681000	3.59987500
H	9.88159500	7.93420600	3.08722400
C	8.29508300	8.56775600	4.40569800
H	7.74028900	9.49173500	4.63754300
H	7.54022800	7.83875600	4.06554500
C	8.96282800	8.04480300	5.66590200
H	9.54801300	7.14146300	5.41649400
H	9.69545700	8.78693900	6.02689100
C	7.98336200	7.71742000	6.78079900
H	7.39902100	8.61783100	7.03102900
H	7.24940600	6.98010100	6.41542100
C	8.65100700	7.18254700	8.03774200
H	9.24144800	6.28951000	7.78074400
H	9.37603200	7.92419100	8.40616200
C	7.65322200	6.84531500	9.13223900
H	8.14343300	6.46224300	10.03338400
H	7.07161700	7.72991000	9.42043900
H	6.93970300	6.08412100	8.79234800
H	8.80059800	9.69324100	-2.12509700
C	12.00371600	11.44675700	-5.01296400
H	11.66730100	10.69064000	-5.73232000
H	12.08024500	12.40351500	-5.54265800
H	13.02433500	11.17122900	-4.70835400

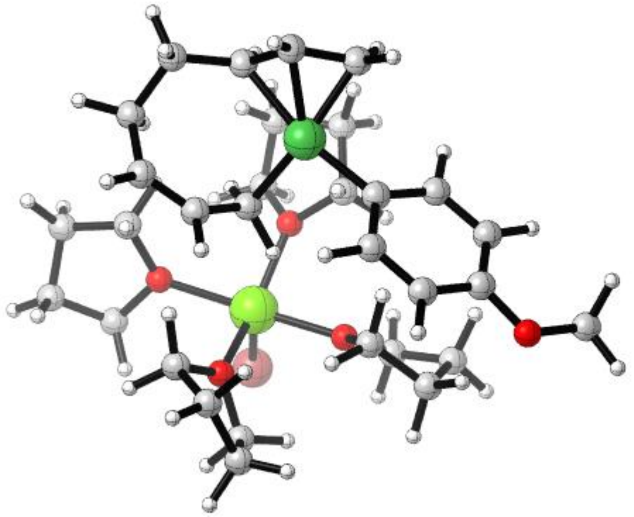

INT1-4-OMe-Ph

Ni	9.74286900	13.36216800	-0.92246300
C	7.91019900	14.05370400	-1.05835000
C	8.31117100	13.94957800	0.28712600
C	9.51200300	14.53415800	0.73187600
C	9.99874100	14.30062300	2.12982200
C	11.49445600	13.93821700	2.23814900
C	11.76866800	12.55386800	1.74708000
C	11.78593200	12.21149300	0.43959300
C	11.67918200	13.09367800	-0.72180300
C	9.63117700	12.46848100	-2.58653600
C	9.25664100	13.05420700	-3.80490200
C	9.20550100	12.36240500	-5.02118200
C	9.50271000	11.00315100	-5.04122100
C	9.84544100	10.36183500	-3.85038300
C	9.91304900	11.08813600	-2.66767300
H	8.02480900	15.00196600	-1.59439900
H	7.07449900	13.44972800	-1.40402000
H	7.87648100	13.15941700	0.90804200
H	9.79251900	15.51164500	0.31272800
H	9.41499500	13.48919600	2.59122900
H	9.81780300	15.19758300	2.74333500
H	12.05477500	14.69737100	1.66864300
H	11.81502800	14.04270900	3.28447900

H	11.77448900	11.76167000	2.49661500
H	11.84034700	11.13848600	0.21623900
H	12.12606600	12.62581300	-1.61881000
H	12.12303500	14.08626000	-0.54807100
H	8.99405400	14.11592100	-3.82737500
H	8.92173300	12.88933000	-5.93021900
H	10.06764000	9.29587300	-3.88514600
H	10.22014200	10.55216000	-1.76434900
Mg	14.55416500	13.90585600	-2.55887500
Br	16.54367900	14.75541200	-3.68498200
O	13.18316800	15.43277600	-2.57739100
C	13.47858500	16.73814700	-2.05568100
C	11.82233600	15.41477300	-3.09191400
C	12.13939100	17.21253300	-1.53799800
H	14.27186300	16.62056000	-1.30764600
H	13.86174000	17.37462700	-2.86750300
C	11.20501700	16.73743800	-2.65001000
H	11.86261600	15.30027700	-4.18291300
H	11.30654700	14.54921000	-2.65445700
H	12.10103300	18.29246700	-1.36998700
H	11.90001100	16.70173200	-0.59410400
H	11.19937500	17.46367300	-3.47188100
H	10.17217000	16.60179300	-2.31230600
O	15.22196200	14.34148600	-0.59091000
C	16.63450600	14.20358000	-0.29518300
C	14.49061700	14.60333800	0.62834600
C	16.70923500	14.10502900	1.21485300
H	17.01028900	13.32492300	-0.83153100
H	17.15375900	15.08707000	-0.68630900
C	15.54447200	14.98834000	1.64254500
H	13.76189900	15.39455900	0.41958900
H	13.94569500	13.69406600	0.92443300
H	17.67808600	14.43260000	1.60227400
H	16.53957200	13.07208400	1.54665500
H	15.81258300	16.04833600	1.54183400

H	15.20717300	14.81374500	2.66847800
O	15.00679400	11.92675900	-2.08337500
C	15.69834000	11.06356500	-3.00237400
C	14.77518800	11.15768300	-0.89593900
C	15.17458500	9.65142900	-2.70501000
H	15.49153800	11.43211300	-4.01306700
H	16.77822400	11.16405500	-2.82035500
C	14.30982100	9.82391700	-1.44273600
H	15.72673000	11.06275400	-0.34531900
H	14.03964700	11.68342500	-0.27678900
H	16.00672400	8.96239600	-2.52874700
H	14.59239400	9.24997700	-3.54097900
H	14.43114300	9.00846500	-0.72442200
H	13.24352000	9.89194400	-1.69807700
O	13.62570900	13.12625900	-4.26142200
C	12.70915600	12.00438700	-4.34306000
C	13.83472400	13.68088500	-5.58277100
C	12.74945600	11.59735300	-5.79729200
H	13.05329600	11.24288800	-3.63524500
H	11.71228300	12.34313600	-4.03694700
C	12.83154400	12.96438100	-6.46614200
H	13.70544800	14.76827600	-5.52254600
H	14.87442400	13.47971600	-5.87418900
H	11.87456700	11.00588700	-6.08551100
H	13.65603400	11.01182400	-6.01325000
H	11.84760400	13.45204600	-6.41200200
H	13.14861100	12.93425100	-7.51255700
O	9.53200400	10.22535200	-6.17391100
C	9.15612400	10.83846900	-7.37631400
H	9.82796600	11.67220400	-7.64136900
H	9.21894600	10.07473800	-8.15524800
H	8.12736100	11.22614700	-7.33650900

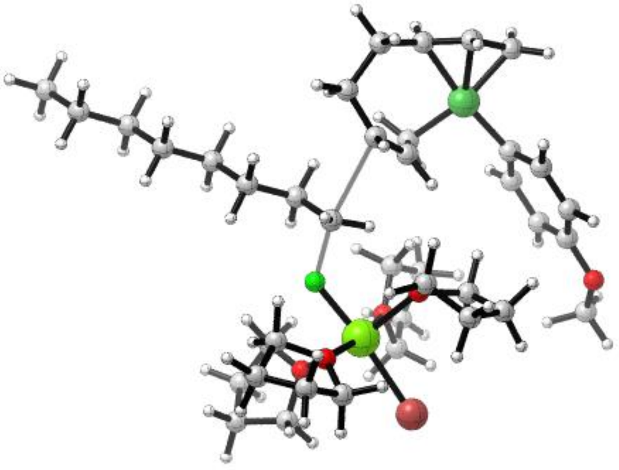

TS3-4-OMe-Ph

Ni	8.29430900	11.24102100	-0.39813700
C	7.09761300	9.80914500	0.18203100
C	7.55077300	10.55458800	1.29052700
C	7.44966200	11.96050000	1.30722300
C	8.17099100	12.74760200	2.36726900
C	9.27139600	13.72916900	1.88127200
C	10.33521700	13.05109200	1.08224500
C	10.05026300	12.60279100	-0.20080800
C	8.95389500	12.98793800	-1.02249100
C	9.03566100	10.29040900	-1.87455300
C	9.03294600	10.66780200	-3.22396200
C	9.85076900	10.08197400	-4.19805100
C	10.73185200	9.06800800	-3.83058900
C	10.73333500	8.61817900	-2.51029000
C	9.90186000	9.21821200	-1.57210000
H	6.15473200	10.07026600	-0.30554500
H	7.35485300	8.75380800	0.11631400
H	8.27929100	10.09548400	1.96812200
H	6.54348400	12.42162300	0.89857700
H	8.65960900	12.04252300	3.05800100
H	7.44953200	13.31628100	2.97348200
H	8.78954800	14.53167300	1.29503200
H	9.69690600	14.21815400	2.77039000
H	11.11155600	12.52454800	1.64458600

H	10.80639300	11.96538700	-0.67643000
H	9.10220200	12.97849700	-2.10402000
H	8.32070600	13.80557700	-0.66821500
H	9.78520500	10.41868900	-5.23155100
H	11.39989100	7.79720500	-2.24687800
C	11.97752800	14.58177200	-0.27566000
H	11.82434200	13.74406100	-0.93437400
F	13.31446400	15.20761500	-1.20117300
Mg	14.35285900	14.20924600	-2.55544900
Br	15.94146000	13.05766000	-4.09247400
O	14.75760500	16.15289300	-3.30396500
C	16.02934400	16.59105500	-3.81345200
C	13.81367900	17.23502500	-3.28885200
C	15.94171800	18.10391200	-3.80422100
H	16.80946700	16.16454500	-3.17228300
H	16.16254000	16.18653100	-4.82668000
C	14.46525500	18.32339300	-4.11932000
H	12.86275800	16.86034800	-3.68879400
H	13.64702700	17.54652800	-2.24706900
H	16.62068800	18.56968000	-4.52379800
H	16.17823700	18.49809100	-2.80604800
H	14.27885300	18.14971800	-5.18782400
H	14.10150100	19.32335600	-3.86717000
O	15.92724600	14.50841200	-1.08906800
C	17.18796000	13.81541200	-0.97367500
C	15.87054600	15.59618600	-0.14692800
C	17.76571200	14.29065600	0.34302700
H	16.99454700	12.73885100	-1.02287300
H	17.81865500	14.08187300	-1.83240000
C	17.28420600	15.73479400	0.38013900
H	15.49332500	16.48444000	-0.66526800
H	15.15534500	15.33810600	0.64775600
H	18.85284600	14.18279400	0.38915200
H	17.32913100	13.72538700	1.17779500
H	17.88770100	16.35217100	-0.29971800

H	17.31970400	16.19061900	1.37371700	C	10.36853500	17.92677700	0.77664600
O	13.50341900	12.39512300	-1.82639000	H	10.46866200	18.44450200	-0.19212700
C	13.06981800	11.32719800	-2.70687100	H	9.32780600	17.56532600	0.81578400
C	14.06612000	11.76160400	-0.67307100	C	10.60383900	18.91573800	1.90605200
C	13.97119200	10.12879800	-2.39945600	H	11.64961100	19.26451900	1.87713500
H	12.01370100	11.10545500	-2.48967700	H	10.48952100	18.39857000	2.87251700
H	13.16110600	11.70416500	-3.73108700	C	9.67073200	20.11444100	1.86080000
C	14.86672100	10.61234000	-1.25114200	H	8.62538700	19.76558500	1.88338100
H	14.64630700	12.52000000	-0.13161200	H	9.78850600	20.63481800	0.89574800
H	13.24683700	11.40760100	-0.02458300	C	9.89813400	21.10062600	2.99571300
H	14.55996200	9.83089600	-3.27184200	H	10.94469500	21.44138900	2.97469600
H	13.35802000	9.27377200	-2.09594600	H	9.77334600	20.57986200	3.95710800
H	15.81425800	10.99210900	-1.65377400	C	8.96305500	22.29569600	2.92933400
H	15.08146800	9.83361700	-0.51340200	H	7.91425400	21.97714700	2.97681500
O	12.69529000	14.22914400	-3.92082900	H	9.13401400	22.99911700	3.75097100
C	11.29494300	14.06406900	-3.62187700	H	9.09343400	22.84521800	1.98852100
C	12.89457100	14.29921100	-5.34826700	H	9.94055500	8.83718600	-0.54796200
C	10.65057300	13.72209800	-4.94742700	O	11.62761500	8.46555800	-4.67544500
H	11.17342600	13.28010300	-2.86633000	C	11.79560000	9.04089600	-5.93971600
H	10.90845300	15.00820000	-3.20449500	H	12.08699000	10.10331100	-5.86823400
C	11.50943100	14.52192000	-5.92018100	H	12.59647500	8.48857500	-6.43769000
H	13.62115600	15.09775700	-5.54325300	H	10.88616800	8.97394400	-6.55589400
H	13.33574200	13.35308700	-5.69187700				
H	9.58656900	13.97424900	-4.97185500				
H	10.74838900	12.64234400	-5.13303400				
H	11.23897000	15.58654700	-5.88105500				
H	11.42916700	14.18837300	-6.95862400				
H	8.36461100	11.46767700	-3.55296300				
C	11.05575200	15.74544300	-0.30953500				
H	10.01776200	15.37889700	-0.29765400				
H	11.18005300	16.24262100	-1.28507700				
H	12.53682400	14.36820100	0.63139000				
C	11.30882600	16.73549300	0.81330800				
H	11.20527200	16.21079400	1.77675200				
H	12.35610600	17.07917200	0.75617800				

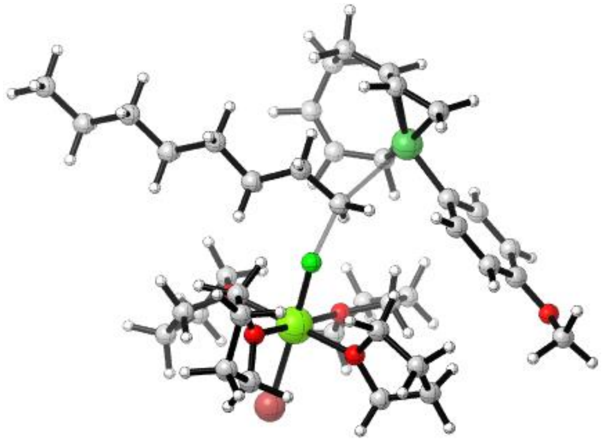

TS10-4-OMe-Ph

Ni	8.40688100	11.73024500	0.09059900	O	13.03725700	9.36281000	0.56446900
C	6.73501800	11.44016500	-0.93662400	C	14.29461800	9.89475600	1.00822700
C	6.44863400	11.45931600	0.44164800	C	12.66333700	10.14046300	-0.59714000
C	6.91537400	12.51592200	1.23594400	C	14.07936400	11.38625600	0.90216300
C	6.72180200	12.57622600	2.71686100	H	14.49676100	9.51042100	2.01173700
C	7.85840100	13.29366300	3.46814900	H	15.09752100	9.54383900	0.34057600
C	9.09361600	12.46383600	3.61966700	C	13.36297900	11.49573800	-0.44429100
C	9.92565600	12.15378000	2.61028500	H	12.99469900	9.60992100	-1.50114600
C	9.82149200	12.53312300	1.19505000	H	11.57171000	10.21573100	-0.62724500
C	9.46466900	11.45006900	-1.48466400	H	15.00878600	11.96107500	0.94906200
C	10.33672100	12.46536500	-1.92928100	H	13.42347500	11.72199000	1.71800500
C	11.08923200	12.37336900	-3.09460200	H	14.08097900	11.64819300	-1.25828200
C	11.00598000	11.23566400	-3.89642700	H	12.64793300	12.32294000	-0.48126500
C	10.14044600	10.21169800	-3.52148600	O	12.22518700	7.39919900	3.06381600
C	9.39359100	10.34469900	-2.34582500	C	12.48345600	6.24914000	3.87726000
H	6.67139800	12.36316100	-1.51783500	C	12.64590900	8.51498000	3.85015100
H	6.51074700	10.54235600	-1.50991000	C	13.79016900	6.54493800	4.61204400
H	6.14613800	10.53293800	0.94088200	H	11.63674400	6.13584200	4.57613400
H	6.97094400	13.50656000	0.76086500	H	12.52434100	5.37805500	3.21707300
H	6.59442000	11.56053000	3.12831900	C	13.95897000	8.07037800	4.47760800
H	5.78050000	13.10673000	2.93203000	H	12.70499100	9.38872200	3.18972100
H	8.08155300	14.22666300	2.92422900	H	11.87916200	8.71402400	4.61740500
H	7.49278800	13.60034000	4.45634700	H	14.62079900	6.02821000	4.12357700
H	9.30847600	12.06668600	4.61429000	H	13.74036300	6.21905700	5.65572500
H	10.77163900	11.50376400	2.87187500	H	14.79997300	8.29128200	3.81029500
H	10.79725000	12.40144000	0.70558000	H	14.14135900	8.57043400	5.43359600
H	9.54343200	13.59848500	1.10058700	O	11.43558800	5.34964300	1.04487800
H	11.75405200	13.17676300	-3.40829600	C	10.09239900	5.08290600	1.47269400
H	10.02166800	9.32135900	-4.13845100	C	12.00888100	4.17727200	0.43312800
C	9.47041000	9.50878900	0.90270400	C	9.65545000	3.89554300	0.64074000
H	10.36049100	10.10194300	1.06708000	H	10.09748600	4.83580200	2.54798700
F	10.49226900	7.93435200	0.82012100	H	9.51909900	6.00073900	1.31967600
Mg	12.32469500	7.33811500	0.89923600	C	10.94968500	3.09289100	0.56144300
Br	14.66267600	6.43154700	1.03411400	H	12.24035300	4.43100000	-0.61209000
				H	12.95371900	3.94066300	0.93485500

H	9.34140200	4.22526500	-0.35912300
H	8.82475300	3.34462800	1.09139900
H	10.97765300	2.38191200	-0.26914700
H	11.09561500	2.52764900	1.49093300
O	12.24251000	7.04758100	-1.20240100
C	11.00032700	6.94696100	-1.92289700
C	13.35584200	7.02469200	-2.11247600
C	11.39740300	6.64979900	-3.35718200
H	10.39645000	6.16507700	-1.44520400
H	10.46923500	7.90419100	-1.83751500
C	12.74452700	7.35671300	-3.45624500
H	14.10304600	7.74025400	-1.75265500
H	13.81147800	6.02387300	-2.09369400
H	10.65603100	7.01527500	-4.07610600
H	11.52251400	5.56943300	-3.51317100
H	12.59102200	8.44303400	-3.54469700
H	13.35991100	7.02398500	-4.29749300
H	10.44546900	13.37671100	-1.33715100
H	9.16704300	9.34266300	-0.11980000
C	8.56722900	9.18525800	2.02948200
H	7.81876100	8.44800100	1.70238700
H	8.01740200	10.11077400	2.26602900
C	9.25178300	8.74226500	3.31324300
H	9.98656700	9.51473500	3.58949200
H	9.81857200	7.81687000	3.14421300
C	8.26429600	8.56359800	4.45273500
H	7.67194900	9.48558500	4.57348400
H	7.54156100	7.77076800	4.19744100
C	8.94222100	8.22901000	5.77069500
H	9.60721500	7.35811400	5.63183100
H	9.60013400	9.06637500	6.05992200
C	7.96631400	7.94198400	6.89976000
H	7.28340100	8.79887500	7.01928100
H	7.32590300	7.08909000	6.62060100
C	8.64591300	7.64892000	8.22802300
H	9.34263800	6.80610400	8.09989100
H	9.26865700	8.51055000	8.51258900
C	7.65570100	7.33665000	9.33661200
H	8.15510000	7.13127200	10.28932300
H	6.96651500	8.17516900	9.49677700
H	7.04684300	6.45940200	9.08407900
H	8.70649600	9.52287200	-2.11755500
O	11.79429100	11.22133100	-5.01450600
C	11.54901600	10.21900500	-5.96198500
H	10.49818000	10.21346300	-6.28793800
H	12.18797200	10.43351900	-6.82193600
H	11.79792800	9.21332300	-5.58478700

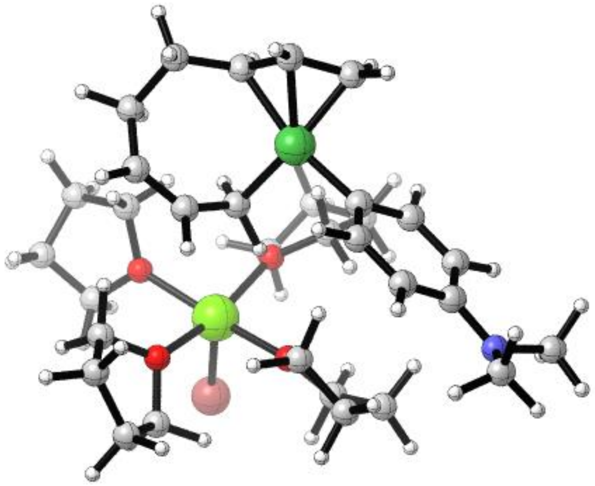

INT1-4-NMe$_2$-Ph

Ni	9.57568800	13.15894300	-0.72559800
C	7.71287400	13.73154500	-0.54166700
C	8.24788400	13.34950500	0.70346800
C	9.46386900	13.89315200	1.16340600
C	10.11519900	13.35354000	2.40007300
C	11.64183700	13.17072200	2.28472600
C	12.01799100	12.00810000	1.42031200
C	11.91162400	12.00517200	0.07077900

C	11.54948500	13.11302000	-0.80399200	H	12.07630800	17.76634300	-1.73032600
C	9.27299200	12.57466100	-2.50095700	H	11.79326700	16.86067200	-4.64592200
C	9.05381300	13.37331500	-3.63366000	H	10.46333100	17.15121900	-3.50686000
C	8.87202100	12.86911600	-4.92176600	O	15.02218700	14.38426000	-0.62993600
C	8.87794400	11.48602200	-5.16087800	C	16.44538400	14.53271900	-0.41868200
C	9.01988300	10.65518800	-4.03857100	C	14.30283200	14.79471000	0.55428200
C	9.21725100	11.19462100	-2.76838400	C	16.59247300	14.69108800	1.07921100
H	7.74737500	14.77840900	-0.86065200	H	16.94838700	13.65787000	-0.84478600
H	6.87082200	13.17310600	-0.94374600	H	16.78738600	15.42051500	-0.96734100
H	7.90712300	12.41339600	1.15826400	C	15.33528600	15.47874300	1.42503900
H	9.65593300	14.95929100	0.97610900	H	13.47287300	15.44059400	0.24260400
H	9.66743500	12.38068000	2.65554200	H	13.89224100	13.89342900	1.03032900
H	9.91607900	14.02054000	3.25441500	H	17.52206400	15.19696400	1.35478900
H	12.04846400	14.12106300	1.90031700	H	16.57313700	13.71071100	1.57444800
H	12.06556300	13.04387400	3.29103100	H	15.45466400	16.53090600	1.13273800
H	12.21142000	11.06191300	1.92837600	H	15.06711700	15.44663300	2.48479600
H	12.06486500	11.04168500	-0.43427300	O	14.90627700	11.75556000	-1.86847800
H	11.88418900	12.95347500	-1.83960200	C	15.68250000	10.96528100	-2.78514300
H	11.87620500	14.09450300	-0.43375600	C	15.14231400	11.19135900	-0.57186200
H	9.00230300	14.46115400	-3.51902800	C	15.57573800	9.53229600	-2.25962500
H	8.71034000	13.56960900	-5.73874500	H	15.27077900	11.13006500	-3.78638300
H	8.98728900	9.57297100	-4.14632700	H	16.71766900	11.33738100	-2.77497500
H	9.34872000	10.48472000	-1.94644700	C	15.05023200	9.69588200	-0.81950600
Mg	14.22371200	13.65773200	-2.43549200	H	16.15152100	11.48684500	-0.23597200
Br	16.14772700	14.39353500	-3.80888100	H	14.38469000	11.58239100	0.11856600
O	13.05515900	15.35690800	-2.58739700	H	16.55038500	9.03556800	-2.29058100
C	13.66074400	16.66300700	-2.69322500	H	14.88804500	8.93313600	-2.86563300
C	11.67901800	15.40504600	-3.04964100	H	15.61967500	9.11878500	-0.08551300
C	12.48210500	17.60930400	-2.73865900	H	14.00240400	9.38060600	-0.75054600
H	14.33823600	16.78160700	-1.83824700	O	13.23251100	12.69021200	-3.99740800
H	14.26394100	16.70081500	-3.61210600	C	12.42656700	11.48201800	-3.92737900
C	11.49928800	16.80992600	-3.58870700	C	13.35888400	13.11552600	-5.37649600
H	11.53086400	14.60928400	-3.78979700	C	12.34073800	10.99427400	-5.35640800
H	11.02552000	15.18348900	-2.19424900	H	12.91885200	10.79555800	-3.22717700
H	12.74083100	18.58303900	-3.16402900	H	11.43869400	11.74269600	-3.52955900

C	12.31124800	12.31408200	-6.11812300
H	13.22781900	14.20469000	-5.40490100
H	14.37871200	12.89023300	-5.71735900
H	11.44699000	10.38582800	-5.52521400
H	13.23171100	10.40944800	-5.62944500
H	11.31780400	12.77168200	-6.00542100
H	12.53785500	12.21561800	-7.18364500
N	8.78614700	10.95766000	-6.46380500
C	8.40958200	9.56873700	-6.59052600
H	7.43754900	9.33281200	-6.12208700
H	8.35448100	9.30594700	-7.65098800
H	9.16329700	8.91311800	-6.13612900
C	8.29846900	11.83388200	-7.50302100
H	8.98096400	12.67927400	-7.65722100
H	8.25336300	11.28208400	-8.44658100
H	7.29573900	12.24795200	-7.29469200

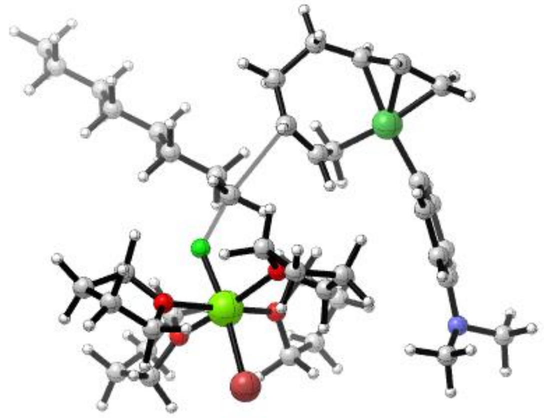

TS3-4- NMe$_2$-Ph

Ni	8.36280700	10.91928900	-0.35587500
C	7.28555700	9.36726000	0.13577400
C	7.80275900	9.99651400	1.29048900
C	7.64532700	11.37939900	1.49933500
C	8.41489100	12.06731000	2.59268700
C	9.46749500	13.12139200	2.14842700
C	10.47580100	12.55976400	1.20243600
C	10.10500200	12.26562400	-0.10418400
C	8.95822000	12.74291200	-0.79880300
C	8.86390900	10.29347700	-2.07658600
C	8.30566100	10.74021100	-3.28999800
C	8.88406400	10.49332100	-4.52739900
C	10.07869700	9.76980200	-4.62986100
C	10.62507200	9.25509700	-3.45441900
C	10.02217700	9.51710800	-2.21954900
H	6.28927100	9.63040700	-0.22979300
H	7.58045100	8.34220800	-0.07822700
H	8.60473100	9.49715600	1.84516900
H	6.69918600	11.84795800	1.20590100
H	8.95617300	11.30485800	3.17473000
H	7.72189900	12.55210500	3.29669600
H	8.93786800	13.97655500	1.69275800
H	9.95010400	13.51262300	3.05584500
H	11.28766700	11.97322300	1.64462100
H	10.83200500	11.69995800	-0.70270900
H	9.03520100	12.87488800	-1.88034000
H	8.33797900	13.49311800	-0.30189100
H	7.39929300	11.34959100	-3.26303600
H	8.44319900	10.91083300	-5.43447100
H	11.52864300	8.64636000	-3.49277800
H	10.49649200	9.09584400	-1.32656600
C	12.00379000	14.29311600	-0.06216700
H	12.57015800	14.08883500	0.84275100
H	11.90563600	13.46627600	-0.74414300
F	13.30346400	15.01687200	-0.97207800
Mg	14.40817200	14.09387800	-2.31253100
Br	16.13652200	13.01204800	-3.75513300
O	14.66308600	16.04708500	-3.09089100
C	15.88622400	16.60960800	-3.59383200
C	13.59297800	17.00284300	-3.16387600
C	15.61504300	18.09918600	-3.66908400

H	16.69389900	16.31671100	-2.91274600	O	12.80245100	13.92445700	-3.70142700
H	16.09817600	16.17603000	-4.58167100	C	11.40046200	13.68434300	-3.44242300
C	14.13481800	18.11754600	-4.03627200	C	13.03826600	13.96223700	-5.12583400
H	12.71045100	16.49099300	-3.56858400	C	10.80133900	13.35406800	-4.79354300
H	13.35483500	17.34519200	-2.14574500	H	11.29612600	12.87305400	-2.71075400
H	16.25622500	18.60891800	-4.39346700	H	10.97031400	14.60037600	-3.00529800
H	15.76705800	18.56809000	-2.68683200	C	11.66938300	14.18366400	-5.73022600
H	14.00652400	17.86641400	-5.09806600	H	13.77411900	14.75195400	-5.31933300
H	13.64396600	19.07710500	-3.85123700	H	13.47289500	13.00320100	-5.44107400
O	15.98104100	14.59862000	-0.87147300	H	9.73226300	13.58131400	-4.83887000
C	17.30426100	14.03777400	-0.75172600	H	10.91998300	12.28286500	-5.01167900
C	15.80166900	15.65685600	0.08697200	H	11.39268800	15.24711600	-5.68253400
C	17.81197700	14.54048700	0.58373500	H	11.61749300	13.86165200	-6.77395600
H	17.22546600	12.94935600	-0.83454500	C	11.01789900	15.40149200	-0.06458700
H	17.91671300	14.39139200	-1.59221100	H	11.05948600	15.88644500	-1.05275500
C	17.18634200	15.92817300	0.63963000	H	10.00584600	14.97345800	0.00684500
H	15.33813000	16.50768700	-0.42512900	C	11.26537000	16.42493300	1.02975400
H	15.10651500	15.31004800	0.86588600	H	11.26876000	15.91211900	2.00551600
H	18.90363200	14.54183700	0.64637400	H	12.27411400	16.85326100	0.90312800
H	17.42230900	13.91671900	1.40023300	C	10.23180200	17.53775100	1.04709500
H	17.73456100	16.61542900	-0.01959200	H	10.21129900	18.03764300	0.06422100
H	17.16109300	16.36618600	1.64151300	H	9.22977200	17.09755400	1.17901600
O	13.70265000	12.23202300	-1.53267500	C	10.48060600	18.56933700	2.13441000
C	13.46479600	11.09292800	-2.40830300	H	11.48470500	19.00603100	2.00268700
C	14.29176800	11.77754200	-0.30239000	H	10.50156500	18.06650300	3.11503100
C	13.88777000	9.87563900	-1.60615000	C	9.44571900	19.68233100	2.15989100
H	12.40232200	11.07401800	-2.68782400	H	8.44212300	19.24583700	2.29118300
H	14.09061600	11.23839400	-3.29753300	H	9.42350300	20.18668100	1.17953800
C	14.93245000	10.45427500	-0.65805500	C	9.69443700	20.71208400	3.25058900
H	14.99033700	12.55282500	0.04336300	H	10.69732500	21.14596500	3.11684700
H	13.49565400	11.65814500	0.45071900	H	9.71658000	20.20488000	4.22704600
H	14.27385700	9.07917600	-2.24919300	C	8.65033500	21.81528300	3.26393500
H	13.03704400	9.47378300	-1.03848300	H	7.64570400	21.40284700	3.42013300
H	15.87462000	10.63140100	-1.19501500	H	8.83514000	22.54853900	4.05601400
H	15.13056200	9.82749500	0.21616400	H	8.63502900	22.35491800	2.30855900

N	10.70157400	9.63748700	-5.90590200
C	9.94428000	8.85904700	-6.86867900
H	10.37004500	8.99195900	-7.87139300
H	9.95173100	7.77736400	-6.63262300
H	8.90229100	9.18889700	-6.89474600
C	12.09053900	9.24771800	-5.89393700
H	12.50187600	9.34891300	-6.90577600
H	12.66370400	9.90466400	-5.22648600
H	12.25796900	8.20009000	-5.57269500

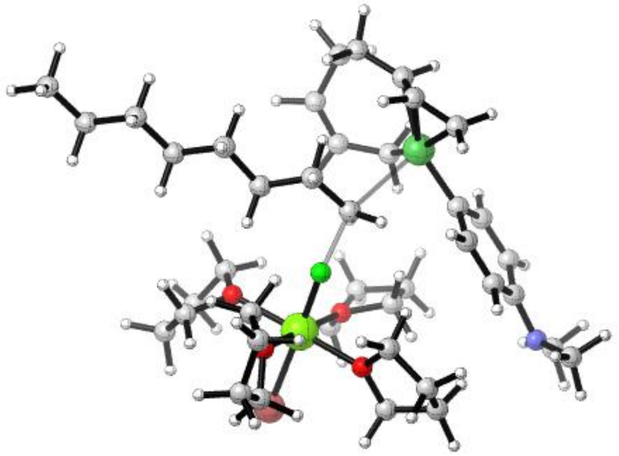

TS10-4- NMe_2-Ph

Ni	8.18886700	11.82926500	0.14047300
C	6.50577500	11.50591700	-0.85547800
C	6.25113800	11.48048900	0.52945800
C	6.69820600	12.53494100	1.33768300
C	6.53770700	12.55619500	2.82361300
C	7.66822700	13.29317600	3.56574500
C	8.93540000	12.50489300	3.66895700
C	9.75296900	12.25271400	2.63206200
C	9.59541900	12.66272600	1.23068200
C	9.22913700	11.58610100	-1.45056500
C	10.12681700	12.56642400	-1.89733100
C	10.94627300	12.41642200	-3.01972000
C	10.92358900	11.24094600	-3.77659200
C	9.97693800	10.27551000	-3.40775900
C	9.16354900	10.46324900	-2.29801000
H	6.39190400	12.44012100	-1.41040900
H	6.30131200	10.61492800	-1.44656700
H	5.99581000	10.53165000	1.01292000
H	6.70834900	13.53712700	0.88408700
H	6.45028400	11.52845300	3.21398500
H	5.58581700	13.05238700	3.07156200
H	7.84739800	14.24514700	3.03835800
H	7.31603800	13.56460500	4.56903000
H	9.18972800	12.09339800	4.64819700
H	10.63009300	11.62962000	2.85420500
H	10.56362700	12.58734000	0.71602000
H	9.26556600	13.71604600	1.17071700
H	11.62266000	13.22761700	-3.28060800
H	9.88101400	9.35507400	-3.98338800
C	9.35789500	9.62671400	0.89045100
H	10.23467500	10.24440900	1.03549600
F	10.44347500	8.08265900	0.74132300
Mg	12.28293400	7.50805800	0.73888900
Br	14.64355900	6.65463100	0.77810200
O	12.95025900	9.54054100	0.39790300
C	14.19729100	10.10364500	0.82565300
C	12.51373500	10.34879900	-0.71782700
C	13.91193500	11.58748900	0.80914900
H	14.45593500	9.67541600	1.79769700
H	14.98848300	9.82645700	0.11032000
C	13.13254500	11.73564100	-0.49841000
H	12.87312700	9.89201600	-1.65197500
H	11.41901800	10.35012300	-0.73518200
H	14.81564000	12.20243300	0.85141300
H	13.27500700	11.85017500	1.66598000
H	13.80235600	11.98755900	-1.32924400
H	12.36639300	12.51483100	-0.45093000

O	12.25947200	7.53818900	2.91795900	H	10.38972700	7.06749000	-4.14710600
C	12.60751100	6.38806500	3.69780100	H	11.33468200	5.67696200	-3.57781600
C	12.66936500	8.65309700	3.70990100	H	12.31263900	8.57987500	-3.80447600
C	13.95487500	6.71783500	4.33621100	H	13.08058600	7.13610200	-4.50108200
H	11.81902800	6.24611200	4.45696500	H	10.22577500	13.49949600	-1.33740000
H	12.62390300	5.52386000	3.02834200	H	9.03137900	9.46026300	-0.12457300
C	14.03276700	8.25552500	4.26082400	C	8.50499100	9.24523700	2.03760100
H	12.65472100	9.54546700	3.07255300	H	7.77574100	8.48612300	1.71702200
H	11.93328600	8.79813600	4.51844300	H	7.92516200	10.14227500	2.31157900
H	14.76326600	6.27138100	3.74863400	C	9.24616900	8.79787500	3.28853300
H	14.01270900	6.34211600	5.36251300	H	9.96941500	9.58316100	3.55930600
H	14.83000100	8.54671500	3.56759200	H	9.83194500	7.89371700	3.07827000
H	14.23184200	8.72765200	5.22764300	C	8.30712600	8.55688000	4.45653600
O	11.44262200	5.49833700	0.90010100	H	7.69487200	9.45647200	4.63242300
C	10.11762500	5.20636900	1.36636800	H	7.59707300	7.75251200	4.20166900
C	12.01222900	4.34397700	0.25205100	C	9.04515500	8.19675400	5.73507500
C	9.67180100	4.02222500	0.53443300	H	9.73050300	7.35305700	5.53755800
H	10.16030700	4.94669200	2.43791300	H	9.68849600	9.04345600	6.02990500
H	9.52586500	6.11732400	1.24180700	C	8.12234900	7.83954300	6.88823400
C	10.97493600	3.24120600	0.40094200	H	7.42301900	8.67233600	7.06811500
H	12.20349800	4.61469100	-0.79687700	H	7.49316900	6.98124500	6.60006100
H	12.97749500	4.11568000	0.71745700	C	8.86044800	7.51288400	8.17692100
H	9.31886800	4.35913000	-0.44996300	H	9.56817600	6.69060700	7.98967200
H	8.86544800	3.45261500	1.00567100	H	9.47678700	8.37677700	8.46861700
H	10.98575800	2.54240600	-0.44033100	C	7.92182800	7.13961700	9.31120500
H	11.16102900	2.66533400	1.31668300	H	8.46321000	6.90706200	10.23425900
O	12.11720300	7.24480600	-1.35917100	H	7.22557200	7.95848500	9.53118600
C	10.84438300	7.08908900	-2.01218100	H	7.31817900	6.26149000	9.04928800
C	13.18328000	7.25507700	-2.32688500	H	8.44366600	9.66606900	-2.08443700
C	11.18028000	6.75816600	-3.45460700	N	11.83744100	10.98418100	-4.82833500
H	10.28890300	6.30310000	-1.48536300	C	11.28288500	10.44000200	-6.05323200
H	10.28956700	8.03234900	-1.92197300	H	10.66031400	11.17014100	-6.60110100
C	12.49487900	7.50486000	-3.65351300	H	12.10109600	10.12473400	-6.71110700
H	13.90469000	8.02458500	-2.03012300	H	10.66962800	9.55696300	-5.85301100
H	13.69662500	6.28291700	-2.29714000	C	12.83146400	11.99923200	-5.08469800

H	13.38248500	12.23285400	-4.16676000
H	13.55005200	11.61897100	-5.81903600
H	12.40870900	12.94244600	-5.47985600

Figure S19

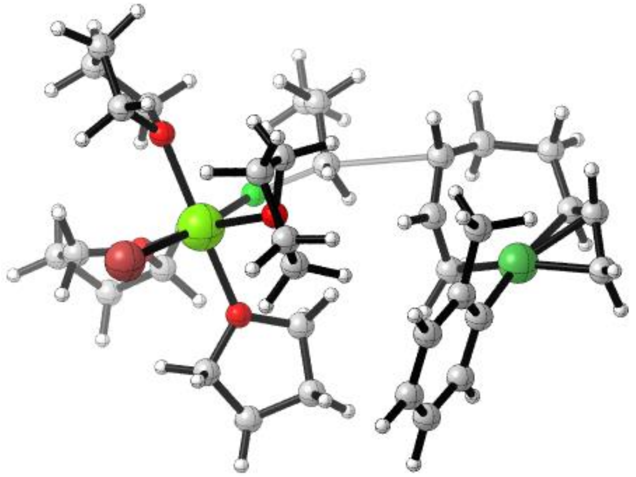

TS3-2-methyl-up

Ni	4.60904600	0.48339900	0.42282600
C	6.50761700	0.29082500	-0.01000000
C	6.07187800	1.60667900	-0.27976100
C	5.37852100	2.35693100	0.68853800
C	4.67967500	3.62906100	0.29184700
C	3.13211400	3.64240100	0.43177800
C	2.46885100	2.53120300	-0.31225700
C	2.55730300	1.23267600	0.17115600
C	2.97679800	0.82864800	1.46826700
C	4.15229900	-1.34355300	0.10813300
C	3.90341400	-2.25738100	1.14452200
C	3.46660000	-3.56470200	0.92650500
C	3.25551200	-4.01391300	-0.37175800
C	3.49902100	-3.13967400	-1.42711600
C	3.92944800	-1.82783900	-1.20361900
H	6.94163400	0.04866600	0.96396500
H	6.89298000	-0.31584300	-0.82705500
H	5.96140800	1.91576300	-1.32541700
H	5.69793600	2.28309100	1.73374100
H	4.90301400	3.83493200	-0.76704700
H	5.08515600	4.47983700	0.86030300
H	2.87116000	3.59217400	1.50312300
H	2.77377800	4.62290600	0.08059400

H	2.33064500	2.66456400	-1.39090800	H	-4.14520500	2.71280700	-3.52834100
H	2.14880900	0.44191000	-0.47097500	H	-6.12762600	2.00841700	-1.30060500
H	2.60143300	-0.11287400	1.86832700	H	-5.55302900	3.63351400	-1.71398900
H	3.12601800	1.60451200	2.22214500	O	-0.48092200	-0.43790100	-1.18986500
H	3.30011900	-4.23283800	1.77235200	C	0.26663300	-1.68283900	-1.18789500
H	3.35638900	-3.48482400	-2.45390100	C	-0.36643200	0.11042400	-2.50781200
C	-0.07165100	2.25565500	0.23552200	C	0.29060600	-2.16606000	-2.64000800
H	0.20499700	1.48352100	-0.46184300	H	1.28161900	-1.48330800	-0.80911300
H	0.39843400	2.19101600	1.21372100	H	-0.24556900	-2.36282500	-0.50125300
F	-1.56678800	1.51844400	0.68600100	C	-0.48322400	-1.09375100	-3.41791100
Mg	-2.27788200	-0.18201300	-0.05612800	H	-1.15156300	0.87122600	-2.61379500
Br	-3.35726300	-2.17913500	-1.08583700	H	0.61873000	0.60064800	-2.60652200
O	-3.83047600	0.06339200	1.40885200	H	-0.17506600	-3.15024900	-2.74351500
C	-5.19247300	-0.38393800	1.28646500	H	1.32800300	-2.24309200	-2.98562800
C	-3.60081200	0.63531700	2.70782900	H	-1.53729600	-1.38199300	-3.51547100
C	-5.92113500	0.32130900	2.41132400	H	-0.07278900	-0.90772000	-4.41491300
H	-5.54056800	-0.13156900	0.27876600	O	-1.28643000	-1.23188800	1.48949800
H	-5.22164700	-1.47773000	1.38901600	C	-0.02602600	-0.84034400	2.06747700
C	-4.85569700	0.32529000	3.50218600	C	-1.74852000	-2.46494600	2.07588400
H	-2.68409900	0.19388300	3.11665900	C	0.47492200	-2.08254900	2.77086900
H	-3.43660700	1.71609200	2.58650000	H	0.62987400	-0.48134200	1.26940500
H	-6.84604900	-0.18588300	2.69938300	H	-0.19930000	-0.00788800	2.76882300
H	-6.17125000	1.35128300	2.11914200	C	-0.83196700	-2.69752300	3.26176800
H	-4.78272300	-0.67068800	3.95925200	H	-2.81107400	-2.33918300	2.31967900
H	-5.03859200	1.05161300	4.29893800	H	-1.66822200	-3.26278200	1.32475800
O	-3.38946100	1.18069500	-1.37205800	H	1.18565100	-1.85109800	3.56971100
C	-4.07659700	0.77466600	-2.57743500	H	0.98317400	-2.74311000	2.05342100
C	-4.04426600	2.33899700	-0.81214200	H	-1.19693700	-2.15547100	4.14555900
C	-4.82176700	2.01237600	-3.02164900	H	-0.75135600	-3.75634200	3.52300600
H	-3.32292300	0.40140900	-3.27895000	C	4.13494700	-0.92252300	-2.38783400
H	-4.74950100	-0.06090000	-2.34157200	H	3.45912600	-0.05277400	-2.35981800
C	-5.26779000	2.57819800	-1.67891900	H	5.15187600	-0.51044900	-2.41003500
H	-4.27911900	2.13648700	0.23946800	H	3.96611300	-1.44541900	-3.33845800
H	-3.34321900	3.18327400	-0.84901900	H	2.92432200	-5.03271100	-0.56469800
H	-5.64954300	1.78306300	-3.69834400	H	4.06130800	-1.93558500	2.17738600

C	-0.55405100	3.57207700	-0.26995500
H	0.28248800	4.06800900	-0.77422000
H	-1.30793900	3.37427600	-1.04672900
C	-1.10723200	4.45988400	0.83086600
H	-0.32621900	4.68076200	1.56897100
H	-1.47171800	5.41320000	0.43498300
H	-1.93364100	3.96551500	1.35515800

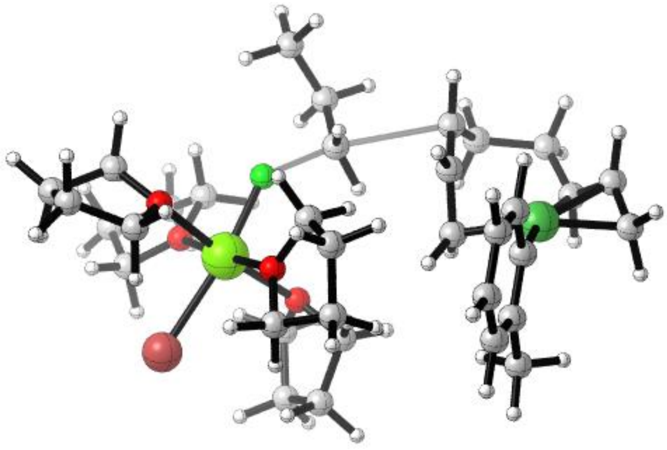

TS3-2-methyl-down

Ni	4.53592500	0.70733200	0.14947600
C	6.45133400	0.85131500	0.50345400
C	5.93120000	2.09175400	0.06924100
C	4.80748600	2.66510100	0.69019800
C	4.12548200	3.85416900	0.08033400
C	2.64328300	3.64261400	-0.31976700
C	2.48019800	2.56752400	-1.34241200
C	2.64201800	1.23657900	-0.99386600
C	2.58137800	0.67805400	0.31464000
C	4.58711800	-1.16019200	-0.18641800
C	4.35653900	-2.14261400	0.80202600
C	4.30427100	-3.49804700	0.47080900
C	4.47180000	-3.92940000	-0.84319000
C	4.70635300	-2.98316000	-1.83376600
C	4.76101700	-1.62874600	-1.49826500
H	6.54192300	0.64561900	1.57379000
H	7.20460700	0.35898400	-0.10729200
H	6.18071900	2.44523000	-0.93686500
H	4.69629500	2.55355800	1.77562200
H	4.66824300	4.14844700	-0.83138800
H	4.17928000	4.71589300	0.76311100
H	2.06399300	3.41607800	0.59259100
H	2.25466300	4.60349100	-0.69360000
H	2.67464700	2.84990700	-2.38113100
H	2.69844500	0.51401300	-1.81763300
H	2.22842300	-0.35175700	0.39914100
H	2.24737700	1.33031500	1.12838500
H	4.12922400	-4.23204300	1.26059600
H	4.85661900	-3.29666500	-2.86683600
C	-0.05705700	2.05303300	-1.29333500
H	0.31699500	1.36698400	-2.04109500
H	0.29028900	1.89061000	-0.27616000
F	-1.57625200	1.23610000	-1.11079200
Mg	-2.33969600	-0.17300800	0.02573100
Br	-3.68781800	-1.77595900	1.40575100
O	-3.86805500	1.36610600	0.40227500
C	-5.20567600	1.19052300	0.92296200
C	-3.61623700	2.76163400	0.15454300
C	-5.89747700	2.51246900	0.66378200
H	-5.66110300	0.32871400	0.42556300
H	-5.13842500	0.95660400	1.99498300
C	-4.74962400	3.49753300	0.83924900
H	-2.61964400	3.00291600	0.54253000
H	-3.61244700	2.92879200	-0.93337000
H	-6.73778100	2.68400300	1.34207600
H	-6.27672700	2.55170900	-0.36685300
H	-4.52789000	3.63528000	1.90637500
H	-4.93572100	4.48225800	0.40137900
O	-3.24989700	-0.88830400	-1.80571600

C	-3.37083100	-2.30379000	-2.07491500
C	-4.36327100	-0.19272600	-2.37790800
C	-4.64170000	-2.46096400	-2.89632400
H	-2.46668100	-2.63847100	-2.59897800
H	-3.44384500	-2.81179600	-1.10458400
C	-5.45213000	-1.23924300	-2.47878400
H	-4.60034100	0.65668100	-1.72714700
H	-4.07877900	0.19543200	-3.36818500
H	-5.14713300	-3.40863300	-2.69070400
H	-4.42308300	-2.41978600	-3.97101900
H	-5.90364000	-1.40504600	-1.49156300
H	-6.24095700	-0.97112800	-3.18763700
O	-0.81677800	-1.60656400	-0.43491100
C	-0.59253100	-2.89886500	0.17315800
C	0.16220500	-1.38981500	-1.48425200
C	0.84472200	-3.22600200	-0.15705800
H	-0.81393000	-2.81654500	1.24142900
H	-1.29859400	-3.62176700	-0.26371400
C	0.97828900	-2.66688300	-1.56814300
H	-0.37276100	-1.13472400	-2.40796300
H	0.78616600	-0.54153800	-1.18085200
H	1.06844400	-4.29471200	-0.09184100
H	1.53665500	-2.70368200	0.51965600
H	0.54175900	-3.36256700	-2.29795100
H	2.02217300	-2.47728700	-1.84650200
O	-1.23513900	0.61340100	1.72182600
C	-0.14383400	-0.12296400	2.30327300
C	-1.92865600	1.17708900	2.84694300
C	-0.75815800	-0.83664800	3.49538700
H	0.27345800	-0.76460100	1.52327500
H	0.63341300	0.59376200	2.61047600
C	-1.86792500	0.12549100	3.95576200
H	-1.40839400	2.10326000	3.13540700
H	-2.94223900	1.42108400	2.51745300
H	-0.01408600	-1.04252700	4.27047000
H	-1.20733700	-1.78652500	3.18281400
H	-1.64563200	0.58954600	4.92189900
H	-2.82221400	-0.40404700	4.03678500
H	4.43528700	-4.99066700	-1.08343300
C	4.14740300	-1.72746800	2.23136300
H	4.95959800	-1.07593600	2.57857500
H	3.22025300	-1.14415100	2.35017100
H	4.09129200	-2.58922400	2.90832500
H	4.96319100	-0.90916800	-2.29704000
C	-0.52545600	3.39620900	-1.74187200
H	-1.31041900	3.73471900	-1.05378000
H	0.30009800	4.10662900	-1.62311300
C	-1.02497800	3.38610000	-3.17541200
H	-0.21520300	3.10086600	-3.85837500
H	-1.83888900	2.66218200	-3.29723100
H	-1.38964000	4.37081000	-3.48368000

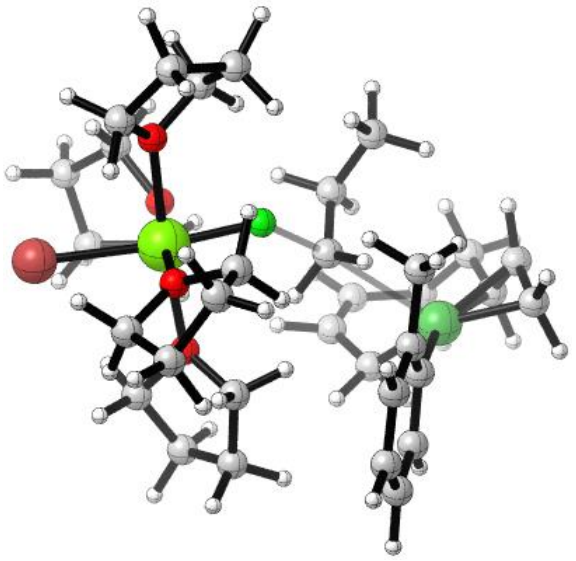

TS10-2-methyl-up

Ni	3.71795200	0.16016100	0.29198600
C	4.92605800	1.05118900	1.58810800
C	4.88227100	-0.33392100	1.83963300

C	5.11947600	-1.22574400	0.78477600	H	-1.69596700	-1.33771100	-3.08711000
C	5.01571100	-2.71121900	0.91768300	H	-2.09714900	0.33293900	-3.52862900
C	4.78526400	-3.42626800	-0.42388500	C	0.49465700	1.18971800	-3.37235700
C	3.37781400	-3.35749500	-0.93060600	H	-0.74260900	2.16835200	-1.84252500
C	2.74626700	-2.23673100	-1.32091900	H	0.63648300	1.27893600	-1.16978800
C	3.23136400	-0.85172900	-1.33762100	H	-0.14143200	-0.16769600	-4.99109200
C	2.95701200	1.86493000	-0.21544200	H	0.69136900	-0.95927500	-3.63446200
C	3.28335000	2.31628100	-1.50835400	H	0.13409400	2.01569200	-3.99653600
C	2.82588000	3.51605800	-2.04965800	H	1.58660600	1.25749300	-3.33670600
C	1.98875900	4.33268100	-1.29994600	O	-1.67839200	-2.40976700	0.07892700
C	1.67091900	3.94328700	-0.00403300	C	-2.64567400	-3.33589300	0.60579000
C	2.15060200	2.74822100	0.54953700	C	-1.01058400	-3.09348100	-0.98587800
H	5.69275400	1.45325800	0.92094900	C	-3.05351400	-4.24697700	-0.55635700
H	4.61243500	1.74617100	2.36310200	H	-2.16179200	-3.89889900	1.41977600
H	4.38847100	-0.70945700	2.74132000	H	-3.47250100	-2.74765000	1.01414200
H	5.86101800	-0.90977000	0.03682700	C	-2.11925200	-3.83775800	-1.70417000
H	4.21810500	-2.98082000	1.63002800	H	-0.48304500	-2.34059400	-1.58307600
H	5.95192600	-3.09882400	1.35094600	H	-0.26085300	-3.78283300	-0.55900200
H	5.48273900	-2.98570800	-1.15672800	H	-4.10043200	-4.08935500	-0.82993300
H	5.08406500	-4.47720500	-0.32302600	H	-2.92470400	-5.30053700	-0.28647100
H	2.82615200	-4.29821900	-0.97638300	H	-2.64954100	-3.15589500	-2.37996700
H	1.70098500	-2.37242400	-1.62645100	H	-1.74597900	-4.68768400	-2.28295200
H	2.58120300	-0.23911300	-1.97724300	O	-3.13101600	-0.49855500	1.89224200
H	4.25236600	-0.80788300	-1.76018300	C	-2.49528300	-0.97458800	3.07839100
H	3.11820400	3.80529300	-3.05884600	C	-4.21279900	0.35607600	2.28952200
H	1.04980200	4.59729700	0.61457700	C	-2.48684800	0.23720100	3.98224500
C	1.23344600	-0.56534800	0.72956100	H	-3.09414500	-1.79661000	3.50696700
H	1.00832400	-0.62870000	-0.32689200	H	-1.50617300	-1.34694700	2.79827700
F	-0.56317600	0.02242200	1.10569800	C	-3.86854100	0.84213000	3.70698200
Mg	-2.09775100	-0.26996400	-0.01370900	H	-4.27892400	1.15963500	1.54797800
Br	-4.22755900	-0.71310900	-1.26443500	H	-5.14886900	-0.21642700	2.24994500
O	-0.93999400	0.09739000	-1.81598500	H	-1.67969400	0.90735400	3.65690800
C	-1.29849000	-0.32061000	-3.14278900	H	-2.32557600	-0.00646600	5.03649900
C	-0.11327900	1.27685200	-1.96855500	H	-3.87014600	1.93476300	3.78154000
C	-0.00576400	-0.15326900	-3.90584700	H	-4.60179900	0.46734200	4.42996500

O	-2.44014600	1.84496300	0.03670500
C	-1.76698600	2.72201900	0.95876800
C	-3.23102300	2.60973300	-0.89478300
C	-2.46809600	4.05488500	0.80655900
H	-1.83952900	2.28361700	1.96057600
H	-0.70605800	2.78253300	0.67858300
C	-2.80130900	4.04879100	-0.68023700
H	-3.04423400	2.22222800	-1.90257600
H	-4.29383500	2.44910100	-0.66586800
H	-1.83798600	4.89515200	1.11371600
H	-3.38996200	4.07605100	1.40544600
H	-1.89759300	4.27575700	-1.26371000
H	-3.57838400	4.76391900	-0.96450100
H	1.61327600	5.27188200	-1.70229200
C	1.75483900	2.46250600	1.97953200
H	0.82916000	1.86926300	2.05038400
H	2.53242800	1.90330800	2.51236800
H	1.57528400	3.39376600	2.53087800
H	3.92174700	1.69263500	-2.13931500
H	1.59240800	0.37883200	1.10049100
C	1.27740400	-1.77331800	1.57068000
H	2.09597500	-2.41298800	1.19422600
H	0.35852300	-2.34532700	1.37260800
C	1.42392600	-1.45452000	3.04802200
H	2.36725700	-0.93078100	3.24423000
H	0.61272000	-0.79305900	3.37785400
H	1.40744500	-2.36002100	3.66275500

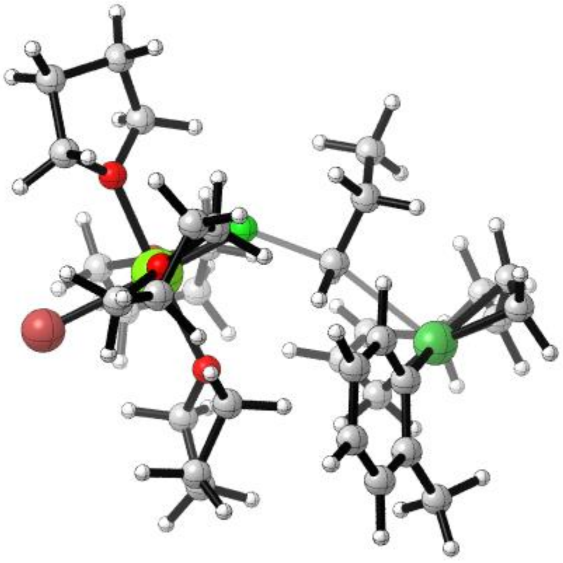

TS10-2-methyl-down

Ni	3.57724100	-0.10627600	0.50034100
C	4.80171900	0.47445500	1.94659000
C	4.55475600	-0.90919600	2.05959500
C	4.71040000	-1.74409800	0.94568400
C	4.37412600	-3.20128100	0.96778000
C	3.91074200	-3.76090200	-0.38974500
C	2.50415200	-3.39065900	-0.73492500
C	2.11527400	-2.15046800	-1.08076000
C	2.93999600	-0.94037500	-1.17885000
C	3.13152200	1.72524800	0.10720300
C	3.61385500	2.36138000	-1.06379900
C	3.21779600	3.66156100	-1.39250300
C	2.37011000	4.40118300	-0.57365000
C	1.95543300	3.83681000	0.62528200
C	2.34196600	2.53517900	0.94266600
H	5.66952800	0.82436800	1.38139000
H	4.51530900	1.13568000	2.76270600
H	3.95479600	-1.28373400	2.89581000
H	5.51077600	-1.48373800	0.23656600
H	3.59877000	-3.39826500	1.72595900
H	5.26293500	-3.76925200	1.28532500
H	4.60892100	-3.39524900	-1.16094400

H	4.01721500	-4.85289600	-0.37791600	H	-2.87200000	-4.72685700	-2.52571900
H	1.75119800	-4.17807000	-0.65749700	H	-0.16345700	-3.88172500	-1.92625900
H	1.04184800	-2.00554300	-1.25043800	H	-1.00440700	-5.19262400	-1.09874700
H	2.42937600	-0.19105200	-1.79674500	O	-3.59047400	-0.51302900	1.35001800
H	3.90422300	-1.17815600	-1.65837400	C	-3.44283200	-1.52619000	2.35459800
H	3.59708500	4.10757800	-2.31372600	C	-4.64347000	0.39746200	1.69749300
H	1.34774500	4.41211500	1.32613200	C	-4.27249100	-1.03913600	3.52718600
C	1.13411700	-0.35985400	1.27107200	H	-3.82526700	-2.47674400	1.95101800
H	1.06077200	0.56008000	0.70968300	H	-2.37368100	-1.63428500	2.56496600
F	-0.78340400	-0.54894800	1.23701700	C	-5.39646500	-0.28995700	2.81902400
Mg	-2.08960800	-0.15416800	-0.14662500	H	-4.18760300	1.34311600	2.03329400
Br	-3.82896400	0.44284700	-1.87705100	H	-5.22668300	0.59618700	0.79166500
O	-0.50722100	0.11614600	-1.57515100	H	-3.68557100	-0.34608100	4.14545200
C	-0.57580700	-0.48684400	-2.88943200	H	-4.61879200	-1.85472800	4.16864400
C	0.06237000	1.44404000	-1.68509200	H	-5.92926500	0.41568000	3.46294700
C	0.18854000	0.44968900	-3.80697100	H	-6.12836800	-0.99828300	2.41016200
H	-0.13326700	-1.48957800	-2.83101800	O	-2.12046700	1.88956800	0.60989100
H	-1.63725400	-0.56229700	-3.16524900	C	-1.42504900	2.23056800	1.82260200
C	-0.04583300	1.80438000	-3.14915000	C	-2.70880300	3.07000300	0.02418000
H	-0.49380000	2.10328700	-1.01162300	C	-1.87896200	3.63739700	2.15476000
H	1.10924500	1.41338300	-1.35410700	H	-1.66992800	1.47178500	2.57586100
H	-0.17003700	0.39589400	-4.83904000	H	-0.34149600	2.20355000	1.62503700
H	1.25946500	0.20428300	-3.79890800	C	-2.06360300	4.22610300	0.76141400
H	-1.05301000	2.17427400	-3.38355100	H	-2.52020700	3.05268900	-1.05392100
H	0.68947800	2.56357200	-3.43398300	H	-3.79689800	3.02594300	0.17508900
O	-2.19812000	-2.26726300	-0.49188200	H	-1.15137000	4.17567800	2.76956300
C	-3.17476200	-2.96187300	-1.27661200	H	-2.83655700	3.62293600	2.69352600
C	-1.22741100	-3.25289500	-0.13257700	H	-1.08399100	4.45747600	0.31819600
C	-2.33844500	-3.82716200	-2.20529400	H	-2.67484400	5.13271100	0.73886900
H	-3.79680200	-3.57401100	-0.60148200	H	2.07863700	5.41276200	-0.84936800
H	-3.79578200	-2.20565500	-1.76609300	H	1.25359800	-1.28782900	0.72744200
C	-1.07641900	-4.13546300	-1.37425100	H	2.01560200	2.14748000	1.91217200
H	-0.31789200	-2.73336700	0.17967600	C	4.57238100	1.66716100	-1.99394900
H	-1.61315100	-3.82755400	0.72620300	H	5.30972600	1.07061400	-1.44166300
H	-2.08009900	-3.25726100	-3.10518700	H	4.05822400	0.97504600	-2.67379600

H	5.12100800	2.38788500	-2.61138800
C	1.25734000	-0.36820700	2.74916100
H	2.23044300	0.05775800	3.03099300
H	0.50516200	0.31903400	3.16086800
C	1.08955900	-1.76602600	3.31932800
H	1.86284600	-2.44060100	2.92856600
H	0.11620000	-2.17350800	3.02350200
H	1.15368800	-1.77285100	4.41171000

Figure S20

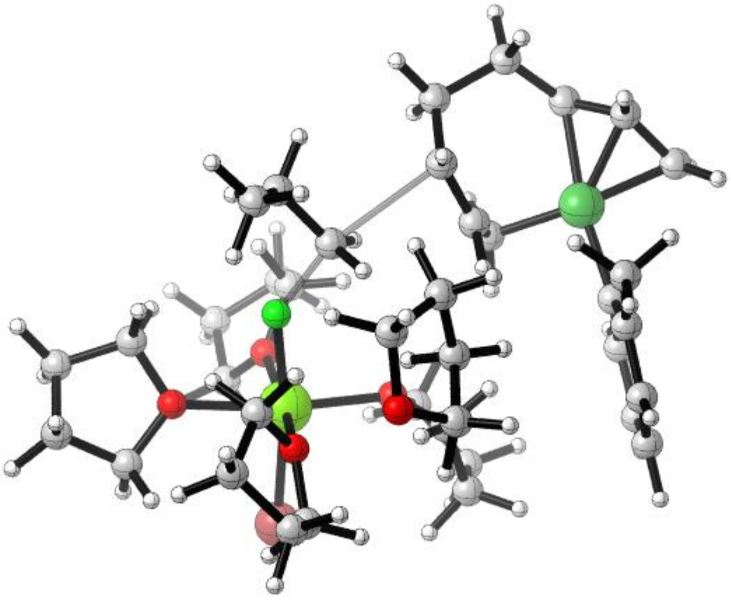

TS3-2-methyl-up·THF

Ni	-4.45102500	-1.10668000	0.00306000
C	-6.39721000	-1.12533900	0.18294800
C	-6.02368300	-1.38218600	-1.15433000
C	-5.08141900	-2.37947900	-1.46664300
C	-4.49610400	-2.44961400	-2.85050500
C	-2.97325800	-2.17105200	-2.97009500
C	-2.56996600	-0.85445800	-2.39345000
C	-2.54772400	-0.69108200	-1.01399300
C	-2.56826900	-1.70691200	-0.02135000
C	-4.15852800	0.10391700	1.44401800
C	-3.75978500	-0.31534900	2.72305400
C	-3.43623600	0.57175400	3.75038100
C	-3.46581300	1.94167500	3.51412000
C	-3.85256900	2.39511800	2.25567000
C	-4.21893500	1.50328200	1.24272500
H	-6.55899300	-1.95739100	0.87381300
H	-6.99712300	-0.24508400	0.40407200
H	-6.18920100	-0.59828000	-1.90245100
H	-5.10688600	-3.31724900	-0.90083700
H	-5.00520200	-1.70701900	-3.48474300

H	-4.70383900	-3.43076600	-3.30374300	H	2.97624700	1.51749200	-1.92233500
H	-2.42248300	-2.98897800	-2.47241400	H	1.47539500	2.47580600	-1.75551900
H	-2.71038100	-2.23514300	-4.03778800	H	3.52593400	4.82509000	0.58045800
H	-2.75224800	0.03375900	-3.00892000	H	1.84910200	4.59385200	0.03714000
H	-2.35203900	0.32029600	-0.64824800	H	4.34682900	3.37325800	-1.22264700
H	-2.09086900	-1.48719300	0.93412300	H	3.04670000	4.27523300	-2.03727200
H	-2.47970900	-2.74818500	-0.34357500	O	0.49852300	-0.49704000	1.39837600
H	-3.15256000	0.19139200	4.73295100	C	0.32698300	-1.49113100	2.43394800
H	-3.88692300	3.46879700	2.05505500	C	-0.20482200	0.68356600	1.84380600
C	-0.00456400	-0.69424900	-2.09039100	C	-0.00764100	-0.73386200	3.72343700
H	-0.36584100	0.28353200	-1.79763700	H	-0.49217800	-2.15959300	2.13072400
H	-0.30798200	-1.51894400	-1.45376400	H	1.25968400	-2.06179800	2.49428900
F	1.56375100	-0.47322600	-1.42437800	C	0.07237200	0.74248800	3.32766300
Mg	2.36205300	-0.31752100	0.38777700	H	0.18376800	1.52603100	1.26306800
Br	3.67535800	-0.10582400	2.49979700	H	-1.28109600	0.56148400	1.63981400
O	4.18532800	-0.41878800	-0.79452600	H	0.69019500	-0.98167700	4.52857900
C	5.44557400	0.18750400	-0.45138300	H	-1.02433200	-0.98002000	4.05156800
C	4.30239000	-1.14828500	-2.02485000	H	1.08062900	1.13621900	3.50892500
C	6.26589200	0.10657000	-1.72196800	H	-0.66880400	1.35704900	3.84891300
H	5.24851900	1.20453900	-0.09509600	O	2.43531100	-2.45877200	0.35388000
H	5.89787600	-0.37613400	0.37590700	C	1.43735800	-3.31873800	-0.21966300
C	5.79174100	-1.22325900	-2.29616800	C	3.50001400	-3.23814900	0.93510100
H	3.81042300	-2.12026100	-1.89690500	C	1.73464700	-4.68672100	0.35726900
H	3.77579100	-0.59828900	-2.81908800	H	0.44746400	-2.92021300	0.03676000
H	7.34206900	0.15129400	-1.53394000	H	1.54620800	-3.30742400	-1.31587900
H	6.00056900	0.92805600	-2.40243400	C	3.25685700	-4.65142200	0.44327600
H	6.24281300	-2.05623400	-1.73984000	H	4.45043700	-2.78949900	0.62233100
H	6.02078600	-1.35759500	-3.35712300	H	3.43620000	-3.15987100	2.02909500
O	2.32070400	1.81179800	0.01427900	H	1.34176400	-5.50055600	-0.25810300
C	2.73302300	2.83445000	0.95456000	H	1.29908200	-4.77279500	1.36201300
C	2.47643300	2.29640300	-1.33586000	H	3.69601200	-4.79801900	-0.55338900
C	2.82589900	4.10862100	0.14117100	H	3.67842800	-5.40502300	1.11416100
H	1.99758000	2.85719800	1.76824600	C	-4.70784800	2.06034600	-0.06859000
H	3.69583700	2.53625400	1.38957000	H	-4.19200900	1.61620000	-0.93239600
C	3.26760100	3.58307500	-1.21885800	H	-5.77638400	1.84988300	-0.21349100

H	-4.57999800	3.15115800	-0.12169400
H	-3.20301600	2.64917900	4.29902700
H	-3.69495000	-1.38805200	2.92698300
C	0.35843500	-0.92957300	-3.51962100
H	1.22432600	-1.60707300	-3.53497200
H	-0.45845800	-1.46779500	-4.01228700
C	0.67499100	0.36235800	-4.25102200
H	1.47565400	0.90802000	-3.73619000
H	0.99185700	0.18158400	-5.28270400
H	-0.21001400	1.01194300	-4.28280100
O	-0.10355500	4.63527800	-1.38990500
C	-0.66907700	4.09712100	-2.58324400
C	-0.82980500	4.13071700	-0.27969300
C	-1.68085700	3.02700800	-2.15557100
H	-1.13510600	4.89951500	-3.17210200
H	0.14910800	3.67438400	-3.18899500
C	-1.27991100	2.75386900	-0.71053000
H	-0.16873600	4.13770400	0.59786000
H	-1.70199500	4.76962600	-0.05012400
H	-1.65586900	2.13214500	-2.79031200
H	-2.70505000	3.42005800	-2.18867800
H	-0.42309600	2.06196500	-0.66968800
H	-2.08476100	2.34861700	-0.08684900

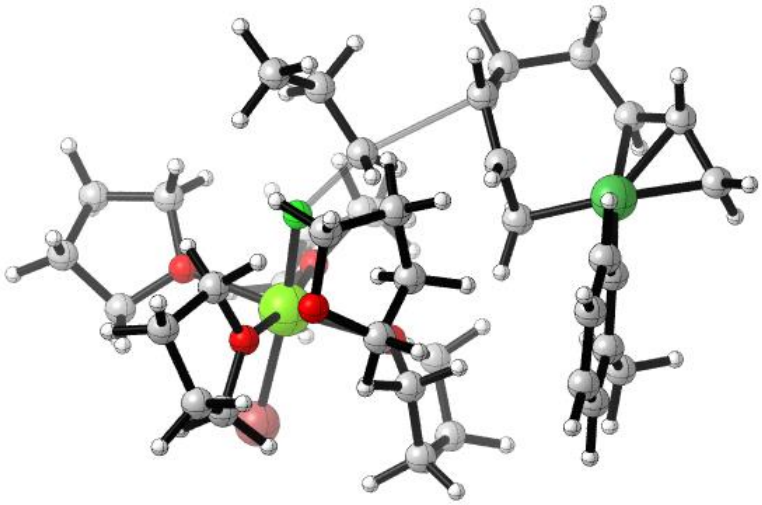

TS3-2-methyl-down·THF

Ni	-4.40390100	-1.05964000	0.06943500
C	-6.27434500	-1.48952100	0.43767500
C	-5.80277100	-2.22644700	-0.67205500
C	-4.61524500	-2.97917200	-0.59575700
C	-4.00051700	-3.55575700	-1.84030600
C	-2.56582400	-3.07291400	-2.18650400
C	-2.50158100	-1.59421000	-2.38668800
C	-2.64359700	-0.74292400	-1.30382700
C	-2.44586400	-1.06295900	0.06734000
C	-4.45167000	0.71109400	0.75840800
C	-4.79968000	1.74139300	-0.12995900
C	-4.65994400	3.09570200	0.18096600
C	-4.15752700	3.46839200	1.42170000
C	-3.83667600	2.47173800	2.34189000
C	-3.99290100	1.11822200	2.03160400
H	-6.23973200	-1.92854800	1.43907400
H	-7.08959200	-0.78513900	0.28864600
H	-6.15637800	-1.95246600	-1.67195500
H	-4.39528900	-3.51189500	0.33713800
H	-4.63809900	-3.30267900	-2.70169600
H	-3.98754100	-4.65487300	-1.78094600
H	-1.88514900	-3.39787500	-1.37814800
H	-2.23681700	-3.60854300	-3.09039100

H	-2.79074800	-1.21973100	-3.37343500	H	1.78345100	4.54796600	-0.22810200
H	-2.78173300	0.31774500	-1.52986200	H	4.20871100	3.47908400	-1.74348800
H	-2.03616400	-0.26997200	0.69174800	H	2.72685800	4.19414600	-2.42355500
H	-2.05244500	-2.05968700	0.30137100	O	0.48970500	-0.06534000	1.48424800
H	-4.93844800	3.85654100	-0.54915600	C	0.16864400	-0.97952200	2.54940900
H	-3.47035500	2.75116500	3.33213300	C	-0.02525500	1.21561800	1.89090400
C	-0.02051000	-0.99739300	-2.03770000	C	0.14286600	-0.15727800	3.84132800
H	-0.43825800	0.00072300	-1.98421200	H	-0.81297000	-1.42499200	2.32689700
H	-0.32316600	-1.66467400	-1.24363300	H	0.93624600	-1.76123900	2.54705100
F	1.49634200	-0.53955700	-1.30780300	C	0.27922200	1.29971200	3.37353000
Mg	2.34473700	-0.22181100	0.44018200	H	0.46717400	1.96781000	1.26509500
Br	3.66599300	0.15914100	2.53126400	H	-1.11060500	1.24838400	1.69925100
O	4.14738000	-0.56683100	-0.71886300	H	0.97164500	-0.43220700	4.50047000
C	5.39512800	0.11971400	-0.51255100	H	-0.79674900	-0.32387500	4.37890300
C	4.21802300	-1.39358900	-1.88874000	H	1.30717400	1.64458400	3.53119300
C	6.14840200	-0.05254200	-1.81592200	H	-0.40787700	1.97830700	3.88922000
H	5.16730600	1.15767000	-0.24587600	O	2.35034400	-2.36817600	0.67520300
H	5.91906100	-0.34235000	0.33590100	C	1.34794000	-3.28486300	0.20907000
C	5.69076300	-1.44221100	-2.24448100	C	3.40182200	-3.08158300	1.36197400
H	3.77802500	-2.37019400	-1.64971400	C	1.64064700	-4.58373000	0.93164200
H	3.61794300	-0.93553500	-2.68941800	H	0.35907800	-2.86142700	0.42857800
H	7.23112000	0.03907400	-1.69325500	H	1.44450200	-3.39743500	-0.88282600
H	5.82186000	0.69731400	-2.55022700	C	3.16142400	-4.53957300	1.02613700
H	6.20370300	-2.20857300	-1.64780400	H	4.36048800	-2.67436700	1.01968400
H	5.86307600	-1.66081200	-3.30216000	H	3.31431900	-2.88253300	2.43879200
O	2.47276500	1.85044300	-0.14435500	H	1.25080500	-5.45766400	0.40275000
C	2.95029700	2.95201400	0.65922500	H	1.19729500	-4.56505100	1.93632200
C	2.45684100	2.22414400	-1.53882500	H	3.60970200	-4.79548600	0.05624200
C	2.81157100	4.16702200	-0.23203900	H	3.57564300	-5.21516000	1.77965300
H	2.35725500	2.97917700	1.58029000	C	-3.68681700	0.08284500	3.07615900
H	3.99226100	2.75441900	0.94860200	H	-4.59944600	-0.44493500	3.38473100
C	3.12357000	3.58555100	-1.60546000	H	-3.01030600	-0.69051400	2.68358500
H	2.97619100	1.44425400	-2.10856000	H	-3.23721000	0.52151600	3.97781500
H	1.40985500	2.27163200	-1.86747700	H	-4.03546700	4.51854300	1.68206000
H	3.48542800	4.97628400	0.06371600	H	-5.18519600	1.48007000	-1.12059900

C	0.48966700	-1.51636600	-3.34038500
H	1.39782800	-2.10139100	-3.13336200
H	-0.24038000	-2.22289000	-3.75079500
C	0.77376300	-0.39715000	-4.32557700
H	1.47329200	0.32987300	-3.89261800
H	1.20240100	-0.77083100	-5.26057400
H	-0.15425100	0.13529800	-4.57196400
O	-0.12699400	4.27509800	-1.69238900
C	-0.69386600	3.72767400	-2.87771300
C	-0.92629700	3.85237200	-0.59562100
C	-1.65848100	2.61395500	-2.44171800
H	-1.20758300	4.51435100	-3.44985700
H	0.12997100	3.34841900	-3.50220000
C	-1.35230700	2.44676300	-0.95380700
H	-0.31926900	3.92610200	0.31734200
H	-1.80991600	4.50267500	-0.47146100
H	-1.52815500	1.69026600	-3.02026500
H	-2.70327000	2.92667000	-2.56780200
H	-0.51109200	1.75307500	-0.79333800
H	-2.21356000	2.09324500	-0.37458200

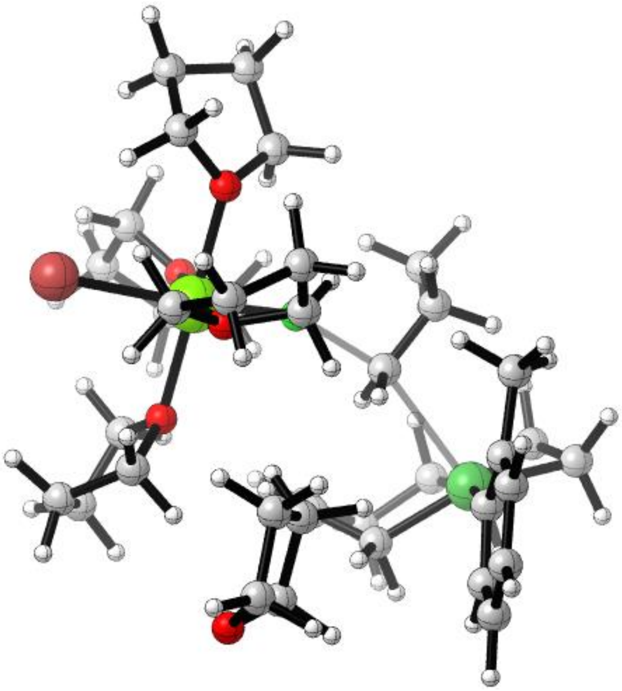

TS10-2-methyl-up·THF

Ni	3.49230400	1.57738900	-0.28933000
C	4.60588000	2.47160800	-1.66857600
C	3.69384100	3.39143600	-1.11051600
C	3.57707400	3.51976700	0.28143800
C	2.54574700	4.38345600	0.93905800
C	2.14058100	3.92083100	2.35158800
C	1.20296500	2.75262000	2.36813100
C	1.54476200	1.50259800	1.99747100
C	2.84801400	1.04774400	1.50881800
C	4.11432100	-0.18560300	-0.76070600
C	3.88015100	-1.01456700	-1.88396000
C	4.41931800	-2.30560500	-1.95550400
C	5.24568700	-2.81071100	-0.95958500
C	5.56018000	-1.98946800	0.11646500
C	4.99460700	-0.71989600	0.20207000
H	5.60098900	2.35915700	-1.23141100
H	4.57502800	2.28272900	-2.74020200
H	2.87387800	3.77097500	-1.72878200
H	4.49958000	3.41291800	0.86983300

H	1.64224400	4.45110800	0.30897700	H	-1.30525800	2.69861500	0.76588700
H	2.93555700	5.41087300	1.01842900	H	-2.30979200	3.53332200	-0.44343000
H	3.06515200	3.68051600	2.90302400	H	-4.71903700	2.46189500	2.43419000
H	1.68548200	4.76699800	2.88205400	H	-5.33269100	3.77315100	1.42312200
H	0.17904100	2.94740100	2.70032500	H	-2.66658000	3.56788800	2.55975000
H	0.74160100	0.75362600	1.99711200	H	-3.15908300	4.76935200	1.36886800
H	2.92347900	-0.03868300	1.59965600	O	-3.05832700	-0.18052700	-2.11299000
H	3.65906300	1.48979700	2.11360700	C	-2.99086500	1.04766200	-2.85920200
H	4.19295300	-2.92237900	-2.82789700	C	-4.16447900	-0.95760300	-2.59668600
H	6.23042300	-2.34087000	0.90052400	C	-4.28777000	1.13092000	-3.66517900
C	1.00675600	1.25300700	-1.00616700	H	-2.88627900	1.86042200	-2.13154800
H	1.35606400	0.23535200	-0.87040400	H	-2.09049000	1.03794000	-3.48916900
F	-0.77975900	0.78125100	-0.85075600	C	-5.17978100	0.08640100	-2.99855700
Mg	-2.22044600	-0.25020400	-0.08943200	H	-3.83594200	-1.56146700	-3.45931800
Br	-4.29692700	-1.34904600	0.85011500	H	-4.48756200	-1.60839700	-1.77828900
O	-1.39183500	-0.32821500	1.90542200	H	-4.11039800	0.86003400	-4.71307500
C	-1.93420200	0.56858000	2.89807900	H	-4.71597900	2.13807600	-3.65376800
C	-1.05230600	-1.58408600	2.54061000	H	-5.96375800	-0.30012500	-3.65582900
C	-1.64453700	-0.08517000	4.23266400	H	-5.65254600	0.48759700	-2.09177600
H	-1.45338700	1.54674600	2.77230400	O	-1.27943800	-2.06805300	-0.75009400
H	-3.01618500	0.65956800	2.72147700	C	-0.32581100	-1.96941700	-1.83305400
C	-1.76065100	-1.56214900	3.87616800	C	-1.93066500	-3.35866500	-0.79372600
H	-1.34734900	-2.39925800	1.87161200	C	-0.67669800	-3.10091300	-2.77301500
H	0.03554100	-1.60924900	2.69010800	H	-0.42011800	-0.97072700	-2.26887800
H	-2.33936100	0.23923900	5.01257600	H	0.68626400	-2.08905200	-1.41469800
H	-0.62159600	0.15365000	4.55543000	C	-1.13946500	-4.17453500	-1.79690100
H	-2.81278400	-1.85178200	3.76048600	H	-1.94797800	-3.77481600	0.22046500
H	-1.28560900	-2.22795000	4.60216300	H	-2.97232500	-3.21502300	-1.11002600
O	-3.06060300	1.74346800	0.26356500	H	0.17273100	-3.40167100	-3.39409300
C	-4.44316700	2.10685800	0.32919300	H	-1.50258300	-2.80238300	-3.43457500
C	-2.33569300	2.95422400	0.49512700	H	-0.27307600	-4.64667300	-1.31655300
C	-4.52459700	3.04295400	1.52559100	H	-1.74570100	-4.95976900	-2.25758200
H	-4.71576700	2.62235600	-0.61015200	H	5.65785000	-3.81501100	-1.03885100
H	-5.01956900	1.18143200	0.42936200	H	0.95336600	1.87639400	-0.11963700
C	-3.12777600	3.69499400	1.57391000	H	5.23599100	-0.12790500	1.08833600

C	3.05335900	-0.56171700	-3.05669500
H	1.97072700	-0.62187500	-2.86774900
H	3.27339300	0.47966900	-3.31755100
H	3.25084400	-1.17556800	-3.94359000
C	0.91899200	1.87113300	-2.35393200
H	1.94154400	2.03533900	-2.71743300
H	0.47969400	1.14464300	-3.05321900
C	0.13907600	3.17347200	-2.36772100
H	0.54928100	3.88812900	-1.64208200
H	-0.90738300	2.99492800	-2.10238300
H	0.16998600	3.64398800	-3.35562500
O	2.28588200	-3.69433800	2.87068500
C	2.53136700	-2.41598100	2.31262900
C	2.47243200	-4.58328100	1.79158600
C	1.93547100	-2.44285800	0.89484300
H	2.09345700	-1.65982500	2.97422500
H	3.61722800	-2.22417700	2.25482300
C	1.70260600	-3.94104200	0.64301000
H	3.54571200	-4.65448700	1.52980500
H	2.11420300	-5.57414700	2.08887600
H	2.63730000	-2.00904200	0.17594800
H	0.99678200	-1.87510100	0.82767400
H	2.05514400	-4.26563600	-0.34417800
H	0.63591400	-4.19254100	0.73463000

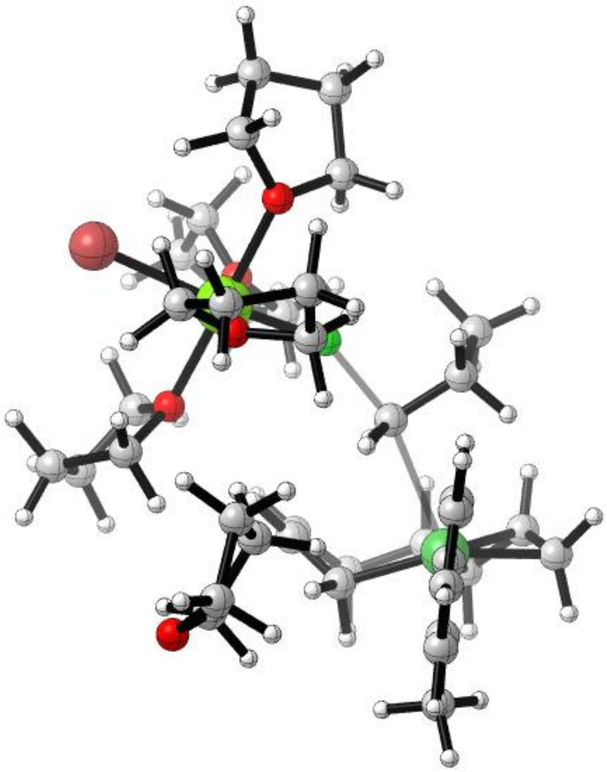

TS10-2-methyl-down·THF

Ni	-3.35104200	-1.61967100	-0.47917600
C	-4.37947600	-2.44185100	-1.96567300
C	-3.49837400	-3.38618000	-1.39838400
C	-3.47071200	-3.59567000	-0.01191900
C	-2.46794000	-4.48678400	0.65152900
C	-2.12074700	-4.07292300	2.09474000
C	-1.17471100	-2.91555100	2.17124000
C	-1.49009200	-1.64936400	1.83350800
C	-2.77776600	-1.14302200	1.35223600
C	-3.85722200	0.15500900	-1.00715100
C	-4.98523100	0.74132700	-0.38034000
C	-5.37049500	2.05378400	-0.66219400
C	-4.68308100	2.83441500	-1.58827500
C	-3.60961000	2.26832200	-2.26099600
C	-3.22228100	0.95916200	-1.96846700
H	-5.40906900	-2.37231100	-1.60607200
H	-4.26466200	-2.17586900	-3.01513600

H	-2.63182300	-3.71949000	-1.97892000
H	-4.42674900	-3.52499800	0.52699800
H	-1.54059200	-4.52363800	0.05308200
H	-2.85639500	-5.51747500	0.67490900
H	-3.06476600	-3.83852000	2.61453100
H	-1.69413700	-4.93722300	2.61965900
H	-0.15373200	-3.13538700	2.49745300
H	-0.66825800	-0.92583000	1.85774200
H	-2.82431100	-0.05356900	1.48172900
H	-3.60228300	-1.59211700	1.93069000
H	-6.23597600	2.47181600	-0.14497800
H	-3.07108600	2.83807600	-3.01994500
C	-0.90167100	-1.14078800	-1.09163900
H	-1.23728600	-0.16382400	-0.76085300
F	0.87720700	-0.68609400	-0.98954700
Mg	2.27352500	0.25958300	-0.02516100
Br	4.29435400	1.27358000	1.12108300
O	1.40332300	0.08092300	1.91441000
C	1.96397800	-0.88893300	2.82821700
C	0.92724900	1.22069100	2.66217900
C	1.54174800	-0.43243100	4.20864000
H	1.57723200	-1.87688800	2.55000800
H	3.05705600	-0.86974700	2.71002600
C	1.53565400	1.08184900	4.03943700
H	1.22290700	2.13045200	2.12929800
H	-0.17043800	1.16461200	2.69852500
H	2.22645000	-0.78442800	4.98564300
H	0.53378200	-0.80094500	4.44106700
H	2.56048900	1.47361400	4.03917400
H	0.95507600	1.60762500	4.80294800
O	3.05902500	-1.75682000	0.14372300
C	4.43069200	-2.15218400	0.23296900
C	2.29276000	-2.96772800	0.15522600
C	4.41936100	-3.23811300	1.29214000
H	4.75201800	-2.54598000	-0.74845900

H	5.01102100	-1.25867700	0.48729700
C	3.06032600	-3.92776400	1.07317200
H	1.27404100	-2.72910800	0.48462800
H	2.23389600	-3.35830300	-0.87393200
H	4.46841500	-2.77503100	2.28462000
H	5.26741200	-3.92298100	1.20256300
H	2.53494300	-4.08786800	2.02093900
H	3.17190500	-4.90832500	0.59952000
O	3.21077900	0.43996100	-1.99300500
C	3.16294800	-0.67009600	-2.90482400
C	4.33497100	1.26356000	-2.33583300
C	4.51548200	-0.69667500	-3.62279800
H	2.97231800	-1.56977700	-2.30958800
H	2.31848300	-0.53298400	-3.59440500
C	5.37032000	0.26717300	-2.80166100
H	4.04335700	1.95686100	-3.14250900
H	4.61571400	1.81936500	-1.43628400
H	4.41343600	-0.32690300	-4.65007900
H	4.93462200	-1.70619500	-3.67827600
H	6.18325200	0.72180400	-3.37488300
H	5.80240200	-0.23075800	-1.92356300
O	1.30251500	2.12544500	-0.53205800
C	0.40693800	2.12947100	-1.66882000
C	1.93374900	3.42133200	-0.40647800
C	0.80948700	3.33785200	-2.48429000
H	0.53007700	1.17491100	-2.18648200
H	-0.62807400	2.20471700	-1.29999900
C	1.22651800	4.32347100	-1.40029200
H	1.85239900	3.75013200	0.63638300
H	3.00258500	3.31452700	-0.63544600
H	-0.00560300	3.70168600	-3.11783700
H	1.66494100	3.09147300	-3.12931900
H	0.34106700	4.78616100	-0.94796300
H	1.87941600	5.12308900	-1.76224600
H	-4.99936300	3.85569400	-1.79367900

H	-0.78732200	-1.90848100	-0.33159900
H	-2.38339500	0.56129300	-2.54380300
C	-5.79971200	-0.03992500	0.61428500
H	-5.98777100	-1.06269000	0.26147400
H	-5.28775400	-0.13126600	1.58098500
H	-6.77166800	0.43179000	0.80101600
C	-0.91131200	-1.51129200	-2.53116100
H	-1.95880400	-1.61571900	-2.84332500
H	-0.51426400	-0.66994800	-3.11666100
C	-0.13172200	-2.78019200	-2.83349200
H	-0.44767000	-3.60499800	-2.18078000
H	0.94009900	-2.62343300	-2.67141700
H	-0.27877200	-3.09863300	-3.87024500
O	-2.84989500	4.00739800	2.67147400
C	-2.62929600	2.63476100	2.39194500
C	-2.95317300	4.66163700	1.42522400
C	-1.99492300	2.55483300	0.99027300
H	-1.99354400	2.22959300	3.19189700
H	-3.58253200	2.08053500	2.41636400
C	-1.86349300	4.02368500	0.58138800
H	-3.94148900	4.48149200	0.96092200
H	-2.83394700	5.73746800	1.59236500
H	-2.65919200	2.02376300	0.30007000
H	-1.02977800	2.03010800	0.97927300
H	-2.00343300	4.17710500	-0.49678400
H	-0.88607200	4.43142100	0.88023300

References

S1. T. Iwasaki, X. Min, A. Fukuoka, H. Kuniyasu and N. Kambe, *Angew. Chem. Int. Ed.*, 2016, **55**, 5550.

S2. R. Benn, B. Büssemeier, S. Holle, P. W. Jolly, R. Mynott, I. Tkatchenko and G. Wilke, *J. Organomet. Chem.*, 1985, **279**, 63.

S3. S. Tobisch, *Chem. Eur. J.*, 2003, **9**, 1217.

S4. D. Alberti, R. Goddard, A. Rufińska and K.-R. Pörschke, *Organometallics*, 2003, **22**, 4025.

S5. P. W. Jolly, *Angew. Chem. Int. Ed.*, 1985, **24**, 283.

S6. G. M. Sheldrick, *Acta. Cryst. A*, 2008, **64**, 112.

S7. T. Iwasaki, A. Fukuoka, X. Min, W. Yokoyama, H. Kuniyasu and N. Kambe, *Org. Lett.*, 2016, **18**, 4868.

S8. P. H. M. Budzelaar, *gNMR*, version 5.06; University of Manitoba: Canada, 2006;https://home.cc.umanitoba.ca/~budzelaa/gNMR/gNMR-license.

S9. A. M. Pangborn, M. A. Giardello, R. H. Grubbs, R. K. Rosen and F. J. Timmers, *Organometallics*, 1996, **15**, 1518.

S10. T. Iwasaki, R. Shimizu, R. Imanishi, H. Kuniyasu and N. Kambe, *Angew. Chem. Int. Ed.*, 2015, **54**, 9347.

S11. Z. Mo, Q. Zhang and L. Deng, *Organometallics*, 2012, **31**, 6518.

S12. G. Cahiez, C. Chaboche, C. Duplais and A. Moyeux, *Org. Lett.*, 2009, **11**, 277.

S13. Z. Jia, Q. Liu, X.-S. Peng and H. N. C. Wong, *Nat. Commun.*, 2016, **7**, 10614.

S14. H. Hamaguchi, M. Uemura, H. Yasui, H. Yorimitsu and K. Oshima, *Chem. Lett.*, 2008, **37**, 1178.

^{1}H NMR of **3aa**

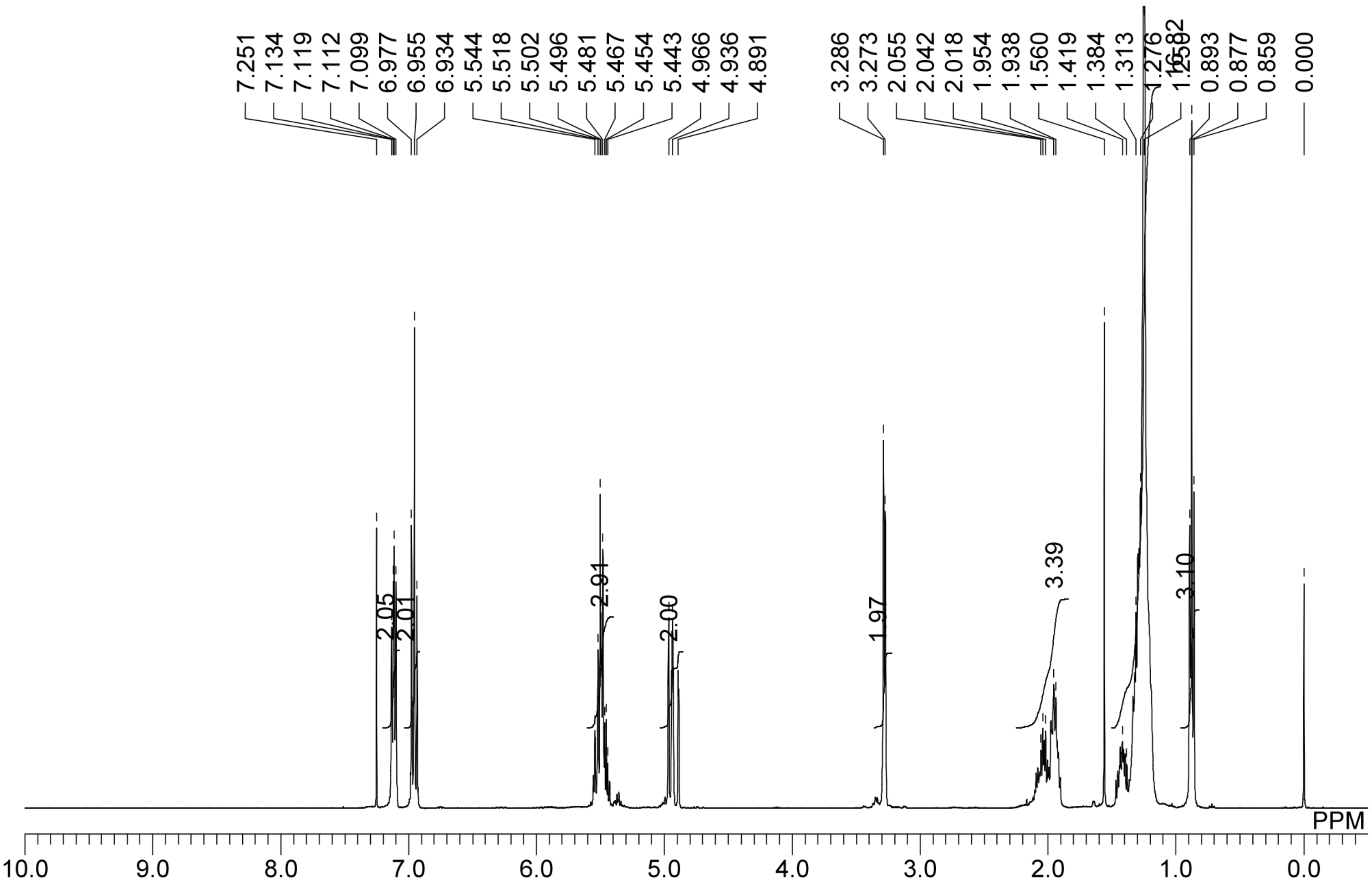
7.251
7.134
7.119
7.112
7.099
6.977
6.955
6.934
5.544
5.518
5.502
5.496
5.481
5.467
5.454
5.443
4.966
4.936
4.891
3.286
3.273
2.055
2.042
2.018
1.954
1.938
1.560
1.419
1.384
1.313
1.276
0.893
0.877
0.859
0.000
2.05
2.01
2.91
2.00
1.97
3.39
3.10
PPM
10.0
9.0
8.0
7.0
6.0
5.0
4.0
3.0
2.0
1.0
0.0

$^{13}C\{^1H\}$ NMR of **3aa**

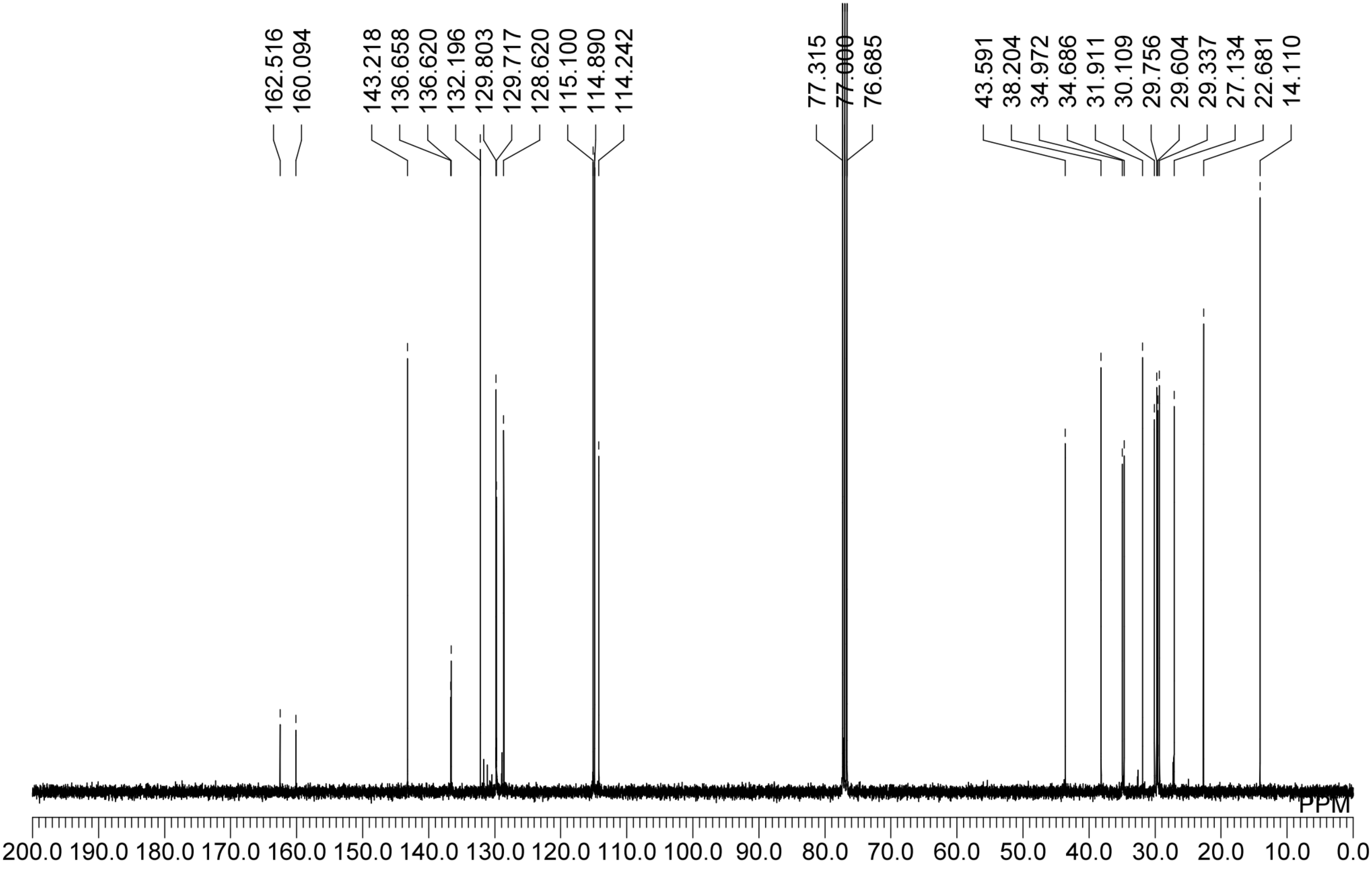

^{1}H NMR of **3ba**

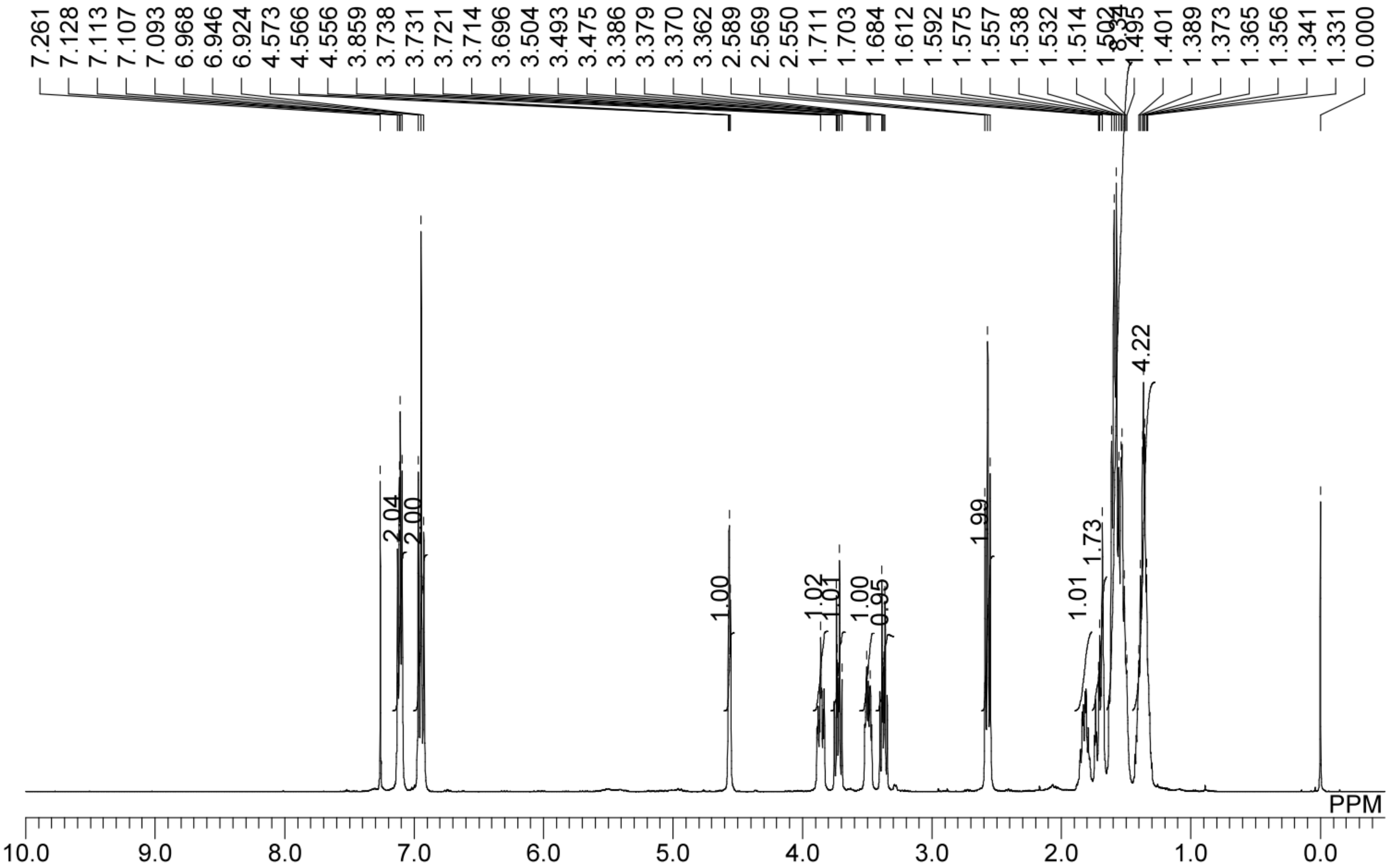
7.261
7.128
7.113
7.107
7.093
6.968
6.946
6.924
4.573
4.566
4.556
3.859
3.738
3.731
3.721
3.714
3.696
3.504
3.493
3.475
3.386
3.379
3.370
3.362
2.589
2.569
2.550
1.711
1.703
1.684
1.612
1.592
1.575
1.557
1.538
1.532
1.514
1.502
1.495
1.401
1.389
1.373
1.365
1.356
1.341
1.331
0.000
2.04
2.00
1.00
1.02
1.01
1.00
0.95
1.99
1.01
1.73
8.34
4.22
PPM
10.0
9.0
8.0
7.0
6.0
5.0
4.0
3.0
2.0
1.0
0.0

$^{13}C\{^{1}H\}$ NMR of **3ba**

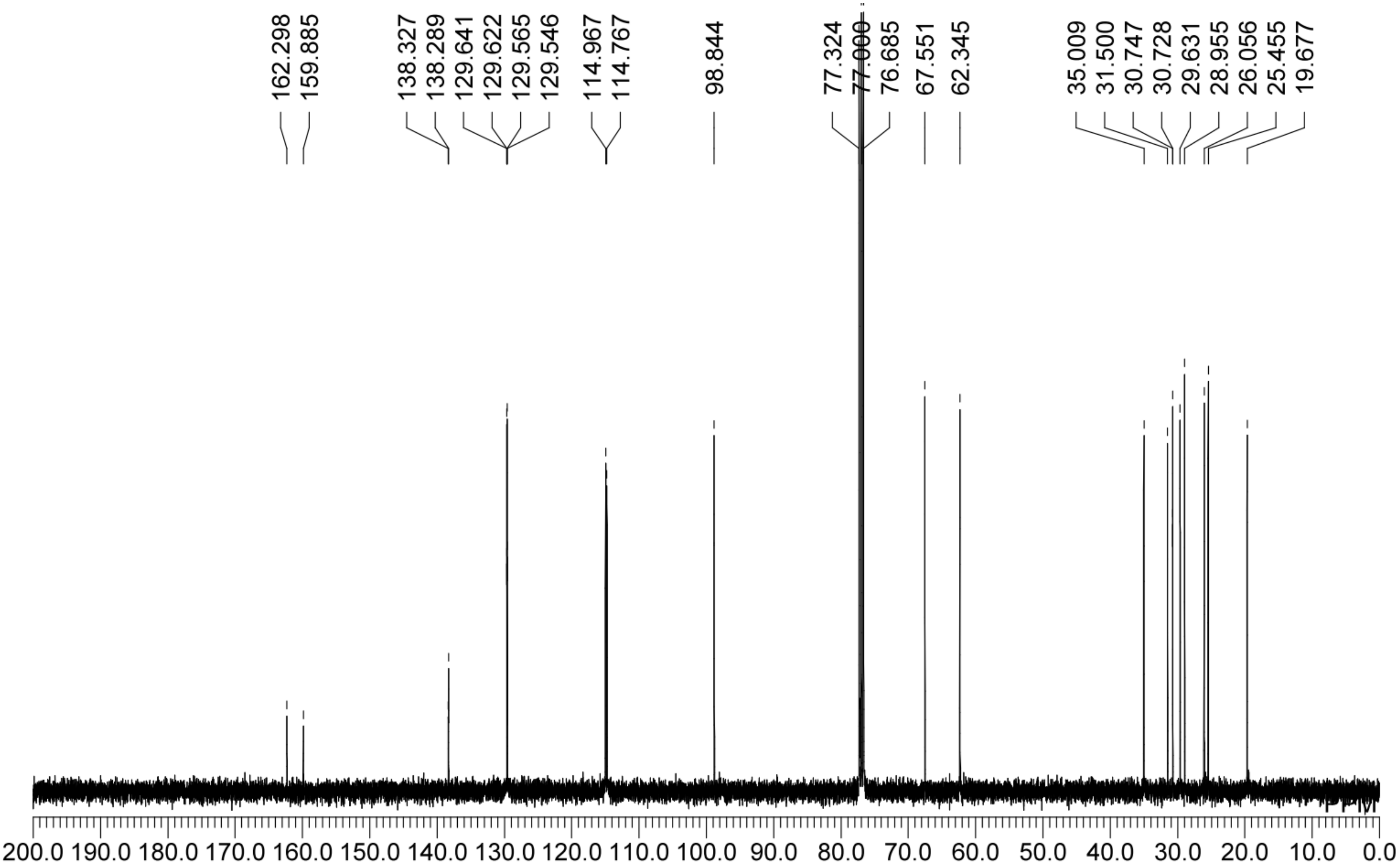

^{1}H NMR of **3ca**

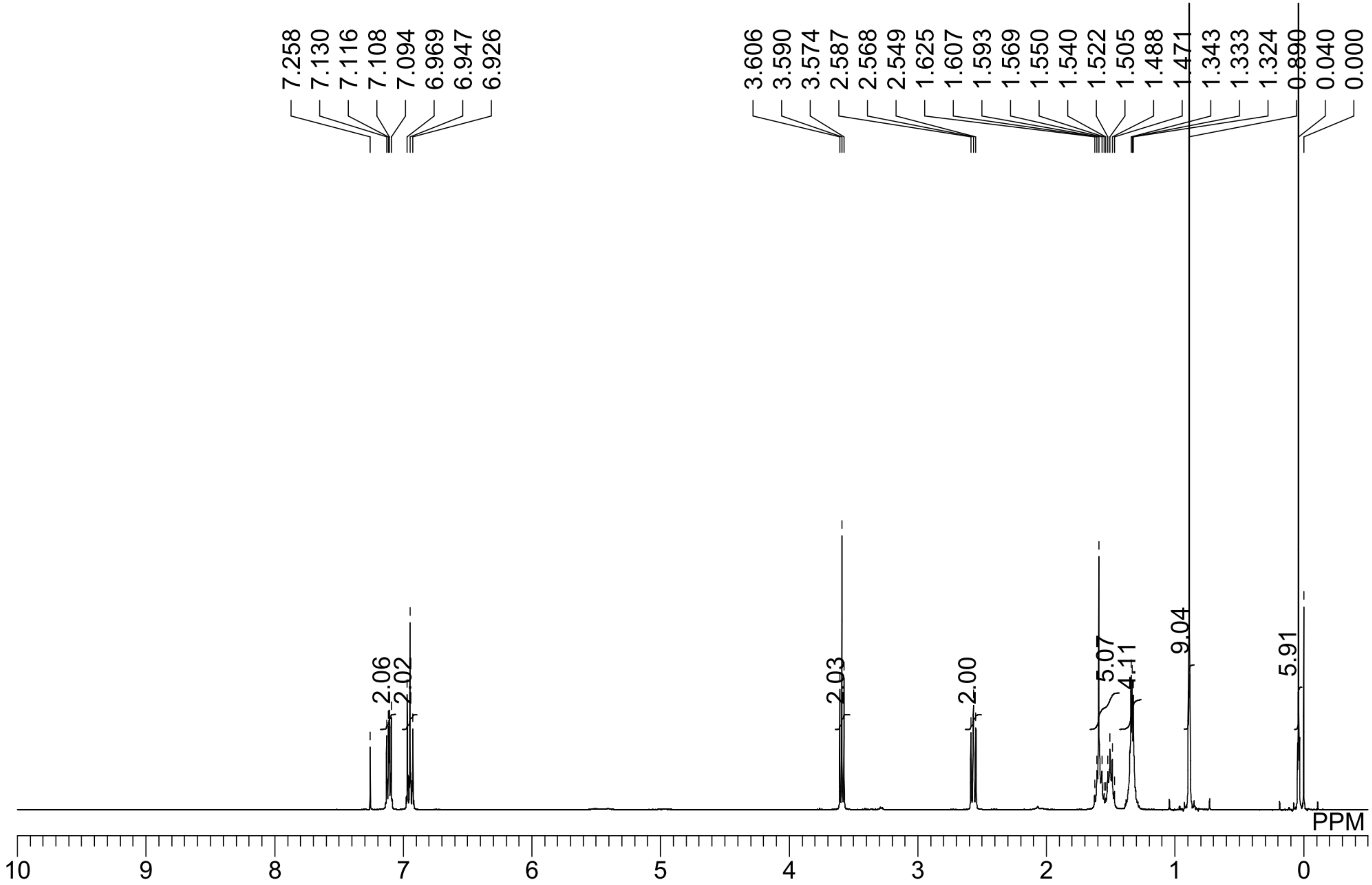

7.258
7.130
7.116
7.108
7.094
6.969
6.947
6.926
3.606
3.590
3.574
2.587
2.568
2.549
1.625
1.607
1.593
1.569
1.550
1.540
1.522
1.505
1.488
1.471
1.343
1.333
1.324
0.890
0.040
0.000
2.06
2.02
2.03
2.00
5.07
4.11
9.04
5.91
PPM
10
9
8
7
6
5
4
3
2
1
0

$^{13}C\{^{1}H\}$ NMR of **3ca**

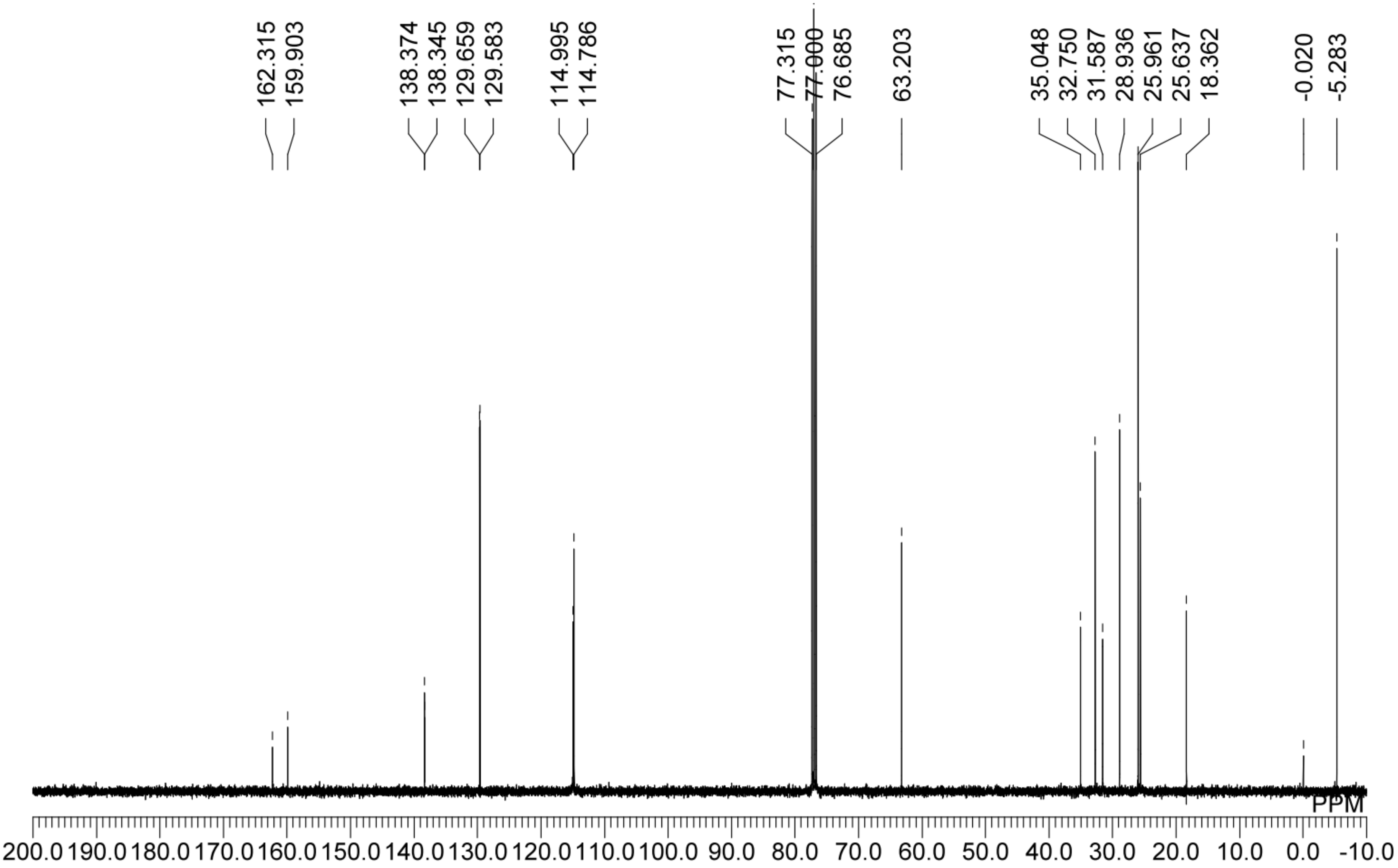

^{1}H NMR of **3da**

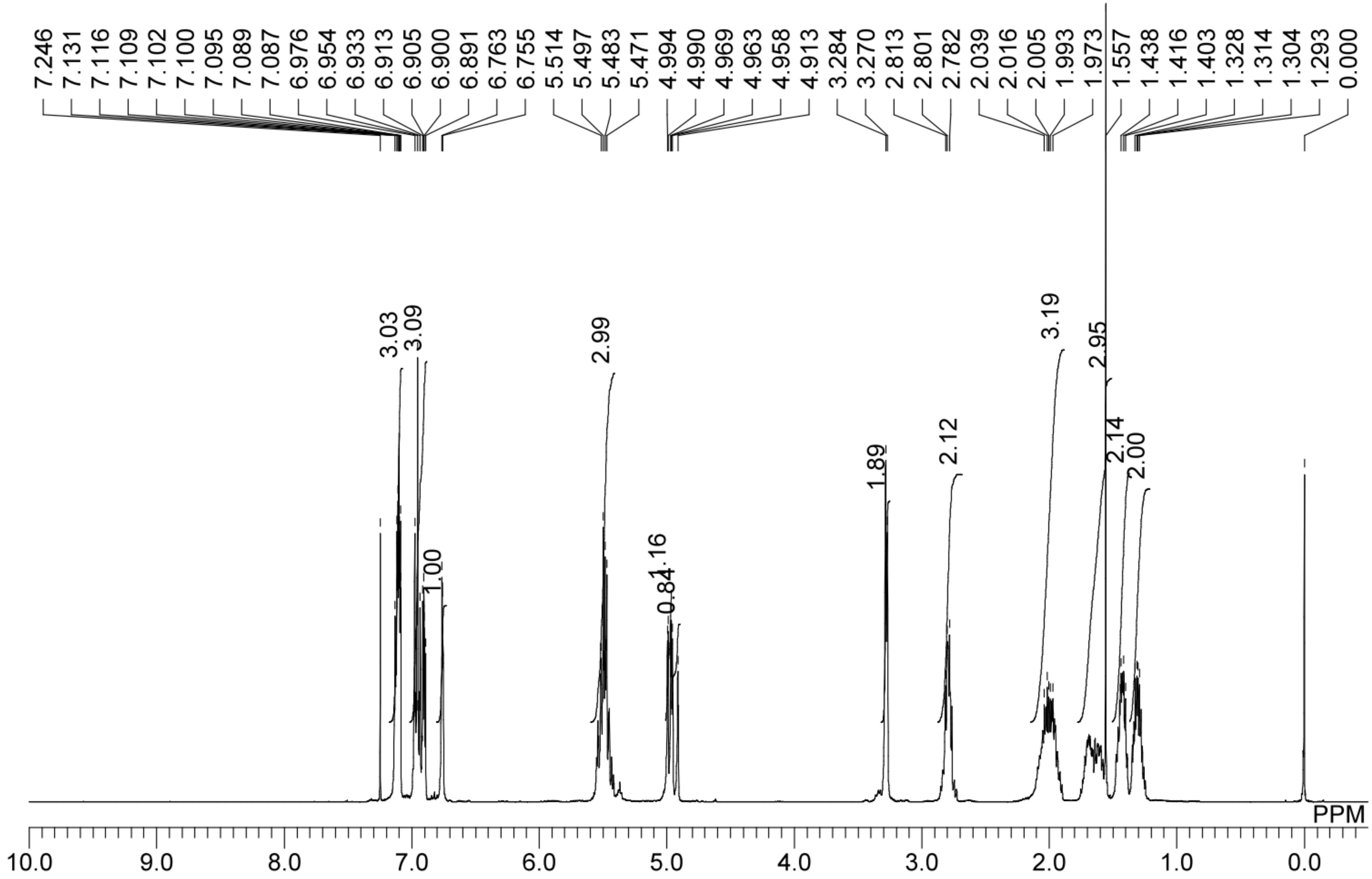
7.246
7.131
7.116
7.109
7.102
7.100
7.095
7.089
7.087
6.976
6.954
6.933
6.913
6.905
6.900
6.891
6.763
6.755
5.514
5.497
5.483
5.471
4.994
4.990
4.969
4.963
4.958
4.913
3.284
3.270
2.813
2.801
2.782
2.039
2.016
2.005
1.993
1.973
1.557
1.438
1.416
1.403
1.328
1.314
1.304
1.293
0.000
3.03
3.09
1.00
2.99
0.84
1.16
1.89
2.12
3.19
2.95
2.14
2.00
PPM
10.0
9.0
8.0
7.0
6.0
5.0
4.0
3.0
2.0
1.0
0.0

$^{13}C\{^1H\}$ NMR of **3da**

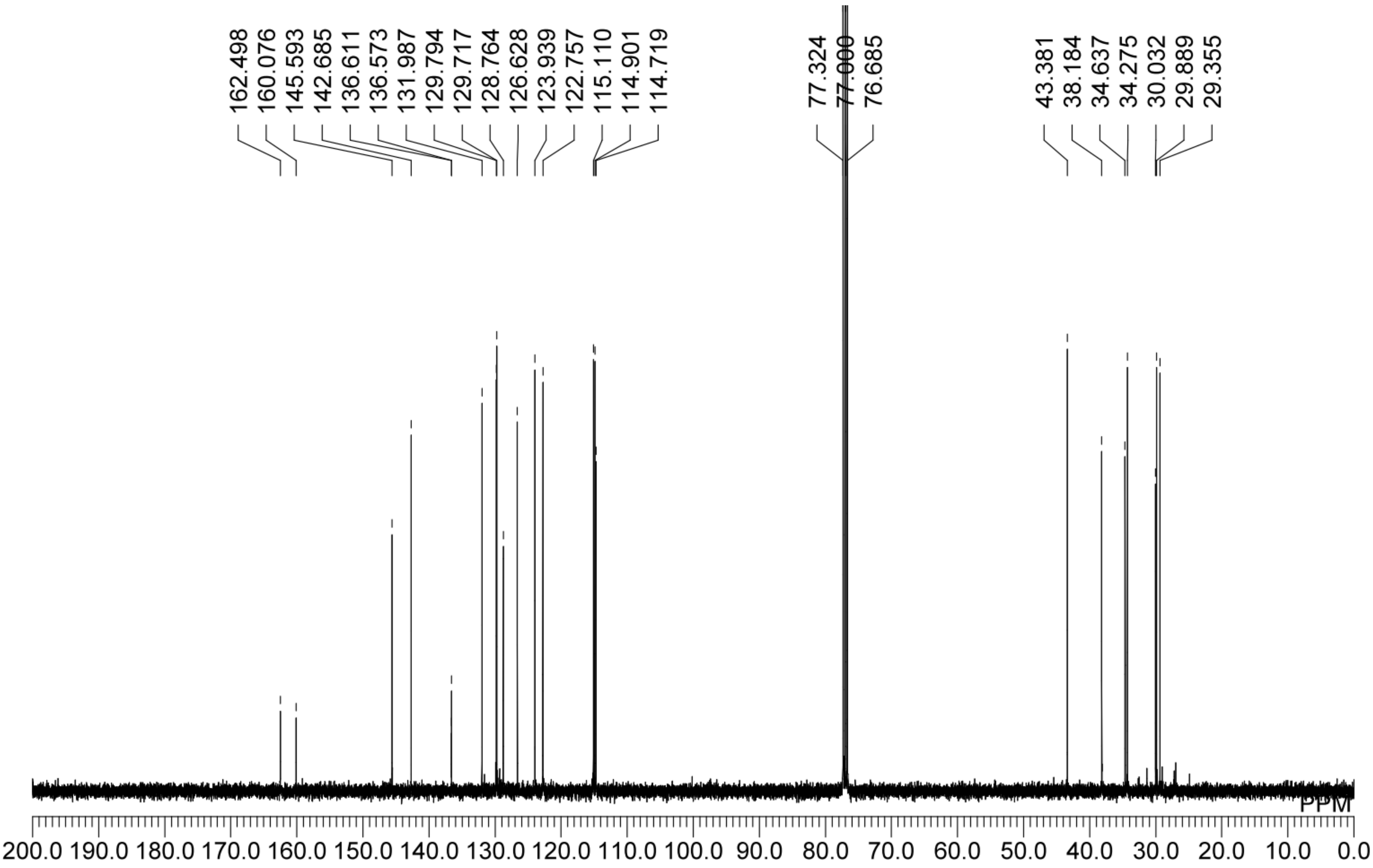

^{1}H NMR of **3ea**

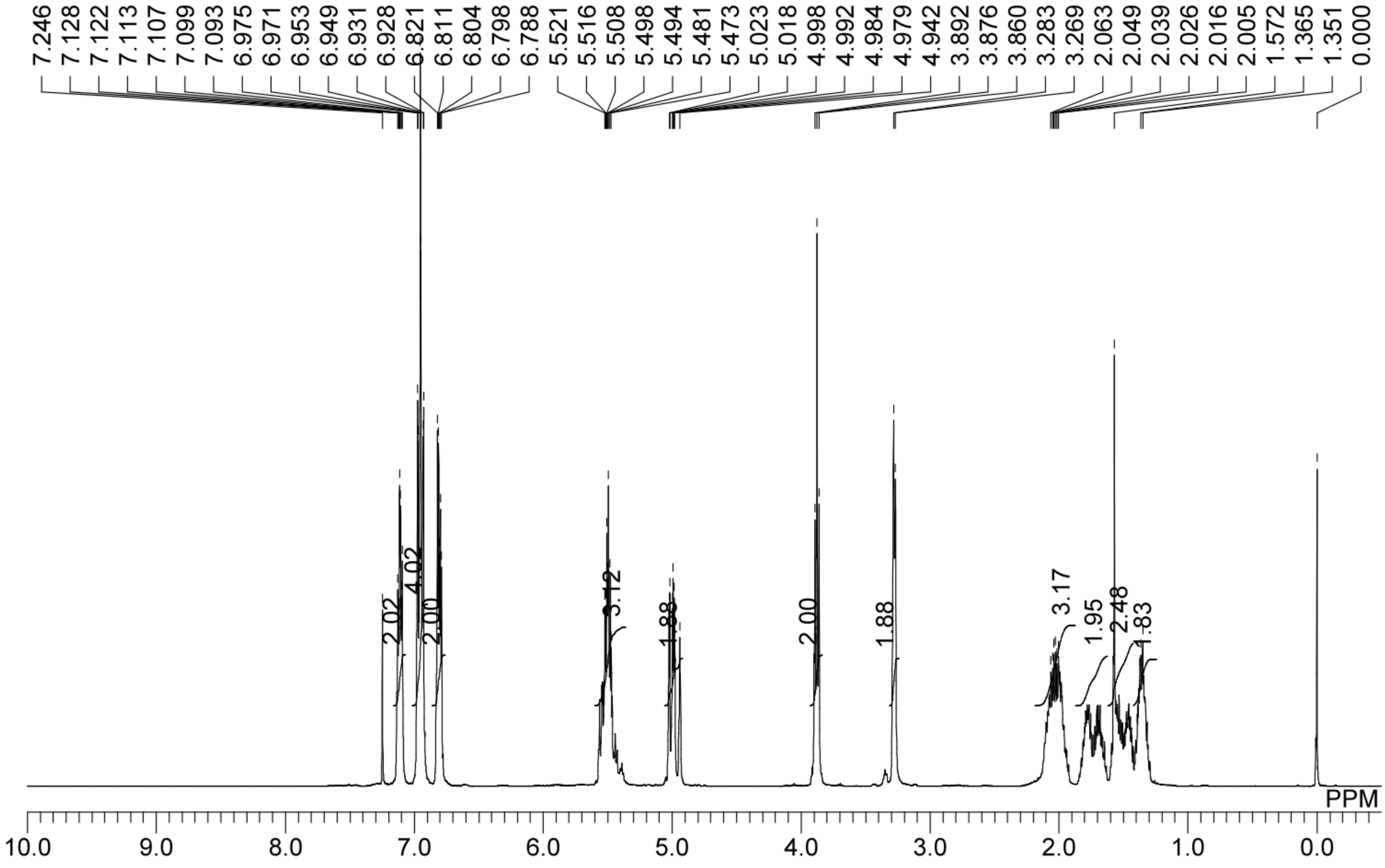

7.246
7.128
7.122
7.113
7.107
7.099
7.093
6.975
6.971
6.953
6.949
6.931
6.928
6.821
6.811
6.804
6.798
6.788
5.521
5.516
5.508
5.498
5.494
5.481
5.473
5.023
5.018
4.998
4.992
4.984
4.979
4.942
3.892
3.876
3.860
3.283
3.269
2.063
2.049
2.039
2.026
2.016
2.005
1.572
1.365
1.351
0.000
2.02
4.02
2.00
3.12
1.88
2.00
1.88
3.17
1.95
2.48
1.83
PPM
10.0
9.0
8.0
7.0
6.0
5.0
4.0
3.0
2.0
1.0
0.0

$^{13}C\{^{1}H\}$ NMR of **3ea**

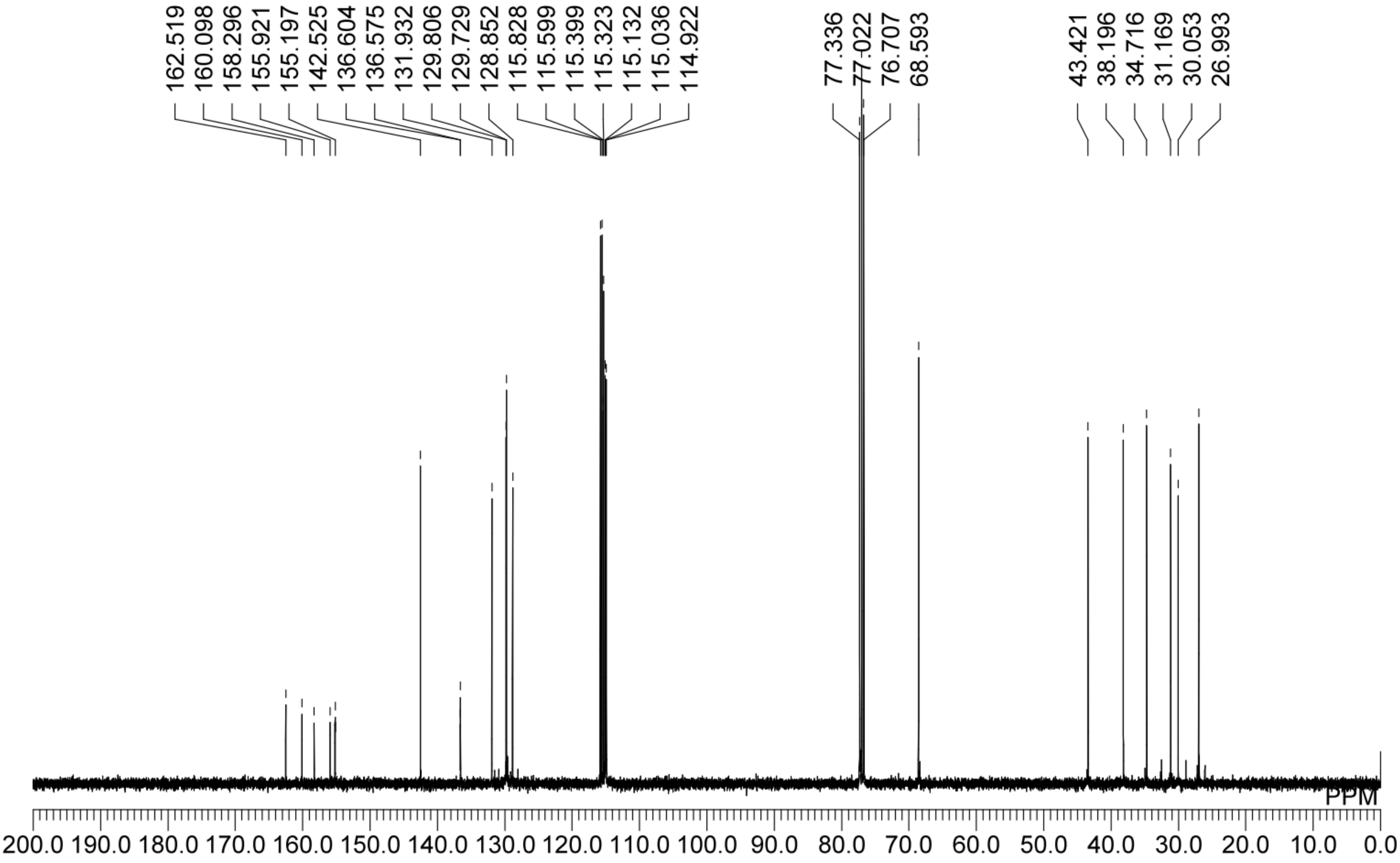

^{1}H NMR of **3fa**

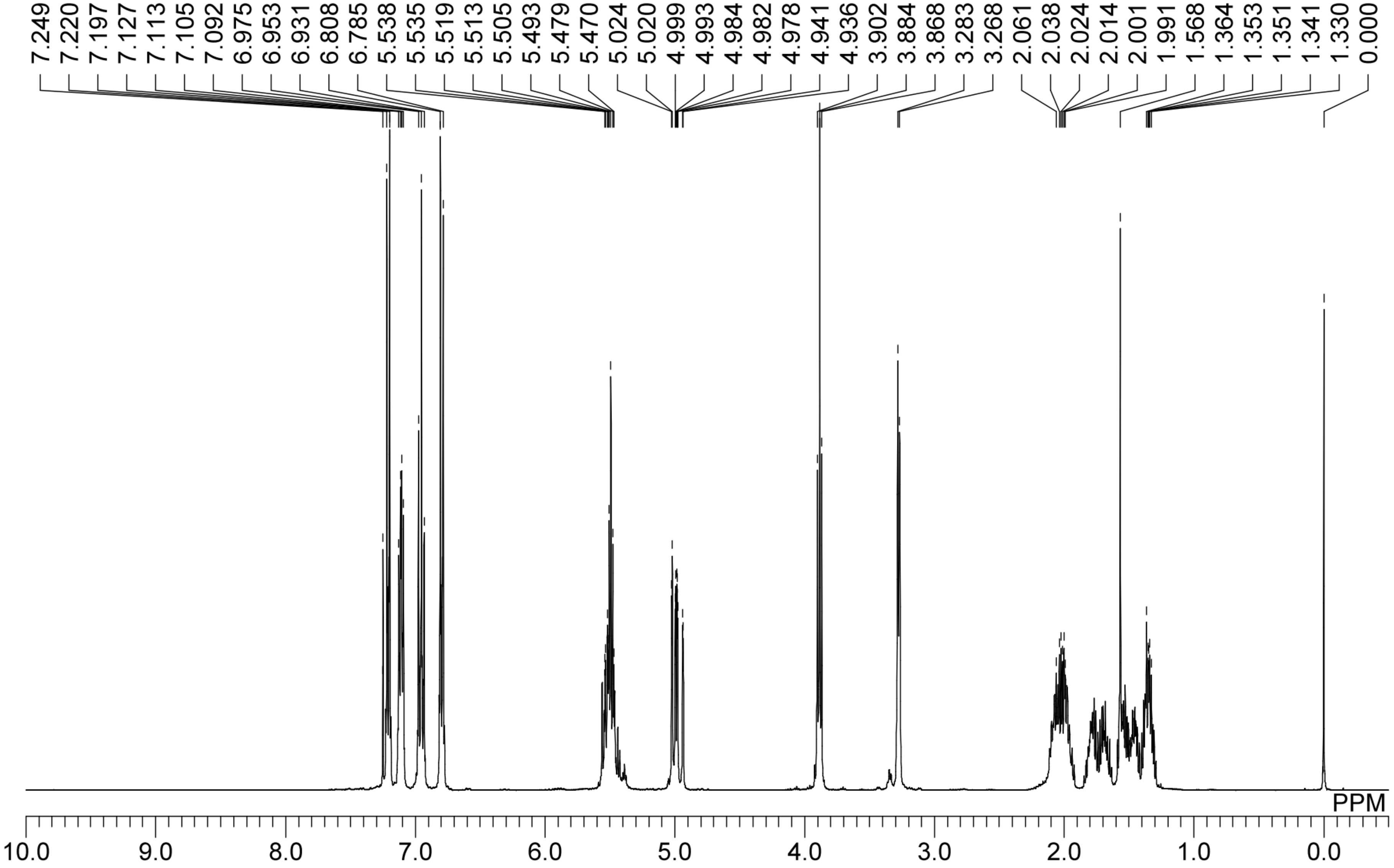

7.249
7.220
7.197
7.127
7.113
7.105
7.092
6.975
6.953
6.931
6.808
6.785
5.538
5.535
5.519
5.513
5.505
5.493
5.479
5.470
5.024
5.020
4.999
4.993
4.984
4.982
4.978
4.941
4.936
3.902
3.884
3.868
3.283
3.268
2.061
2.038
2.024
2.014
2.001
1.991
1.568
1.364
1.353
1.351
1.341
1.330
0.000
PPM
10.0
9.0
8.0
7.0
6.0
5.0
4.0
3.0
2.0
1.0
0.0

$^{13}C\{^1H\}$ NMR of **3fa**

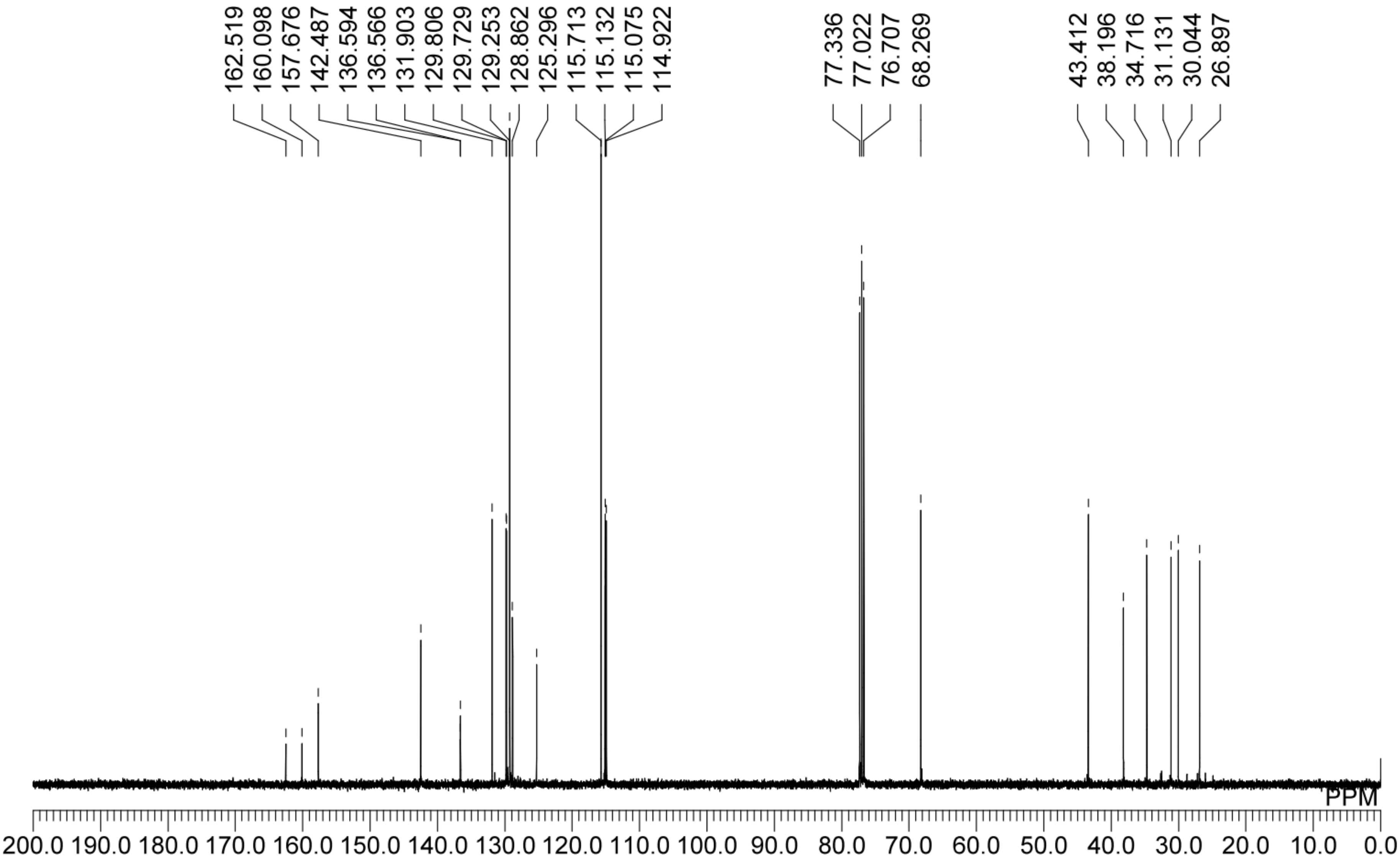

^{1}H NMR of **3ga**

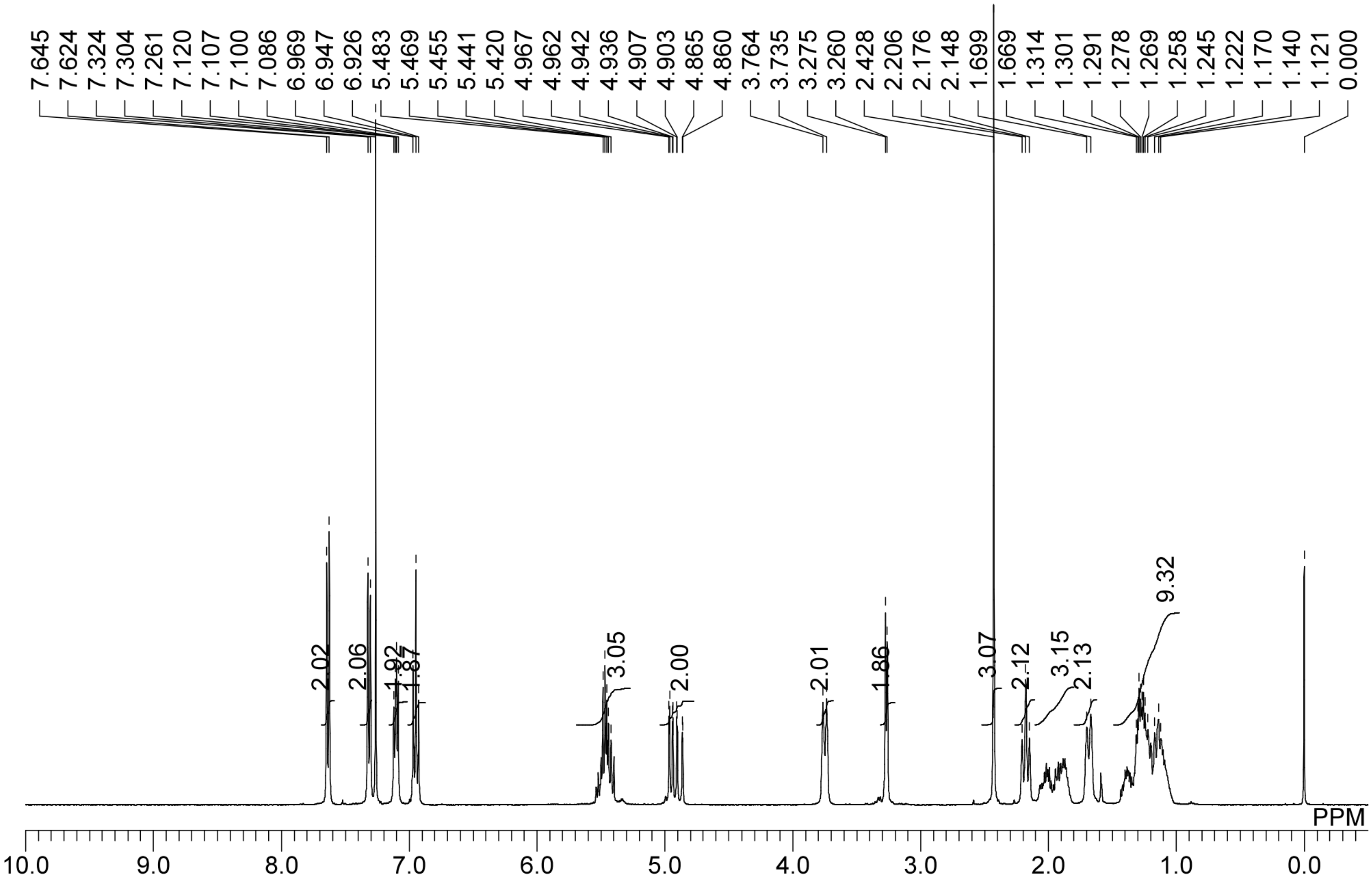

$^{13}C\{^{1}H\}$ NMR of **3ga**

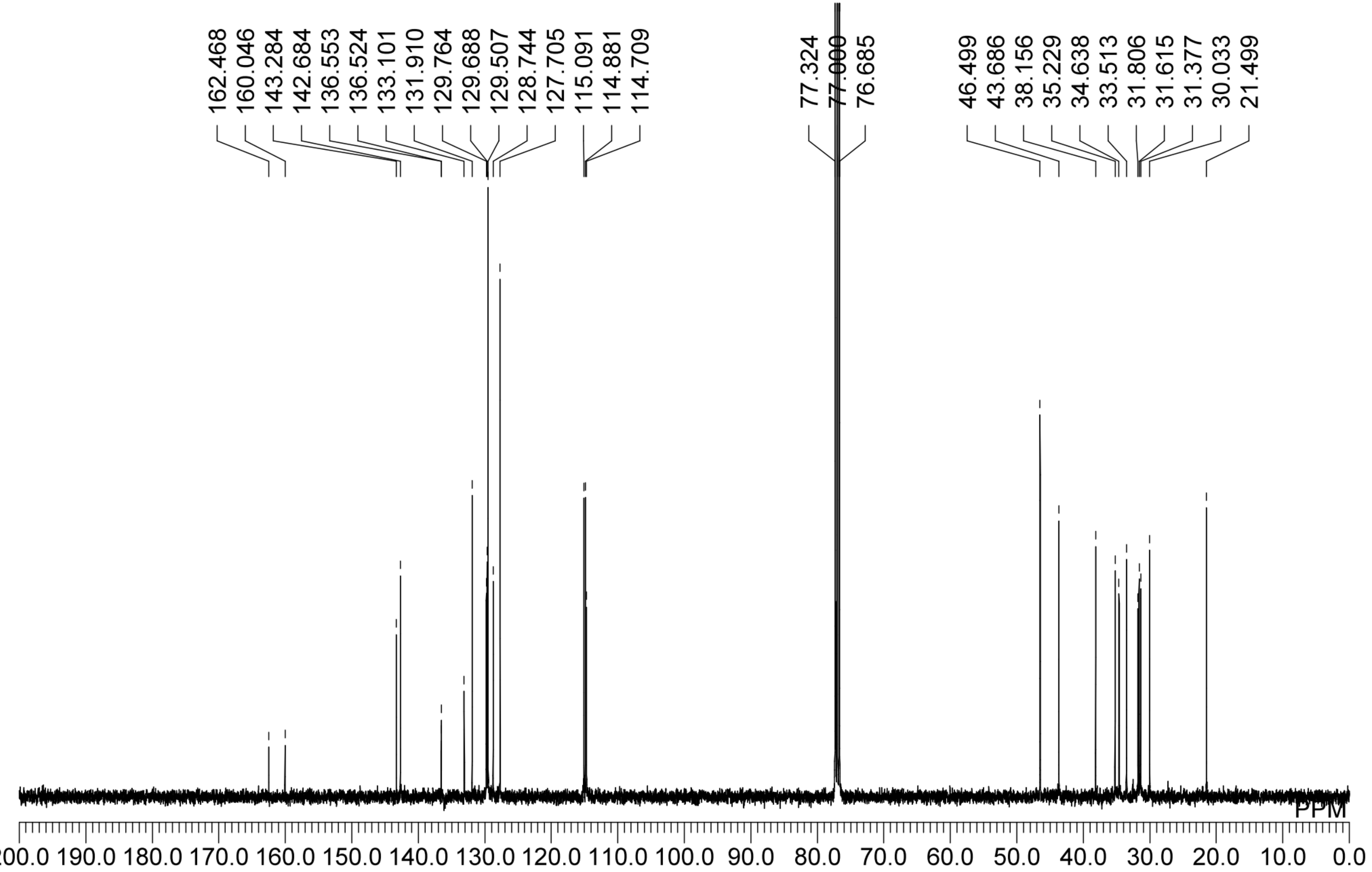

^{1}H NMR of **3ha**

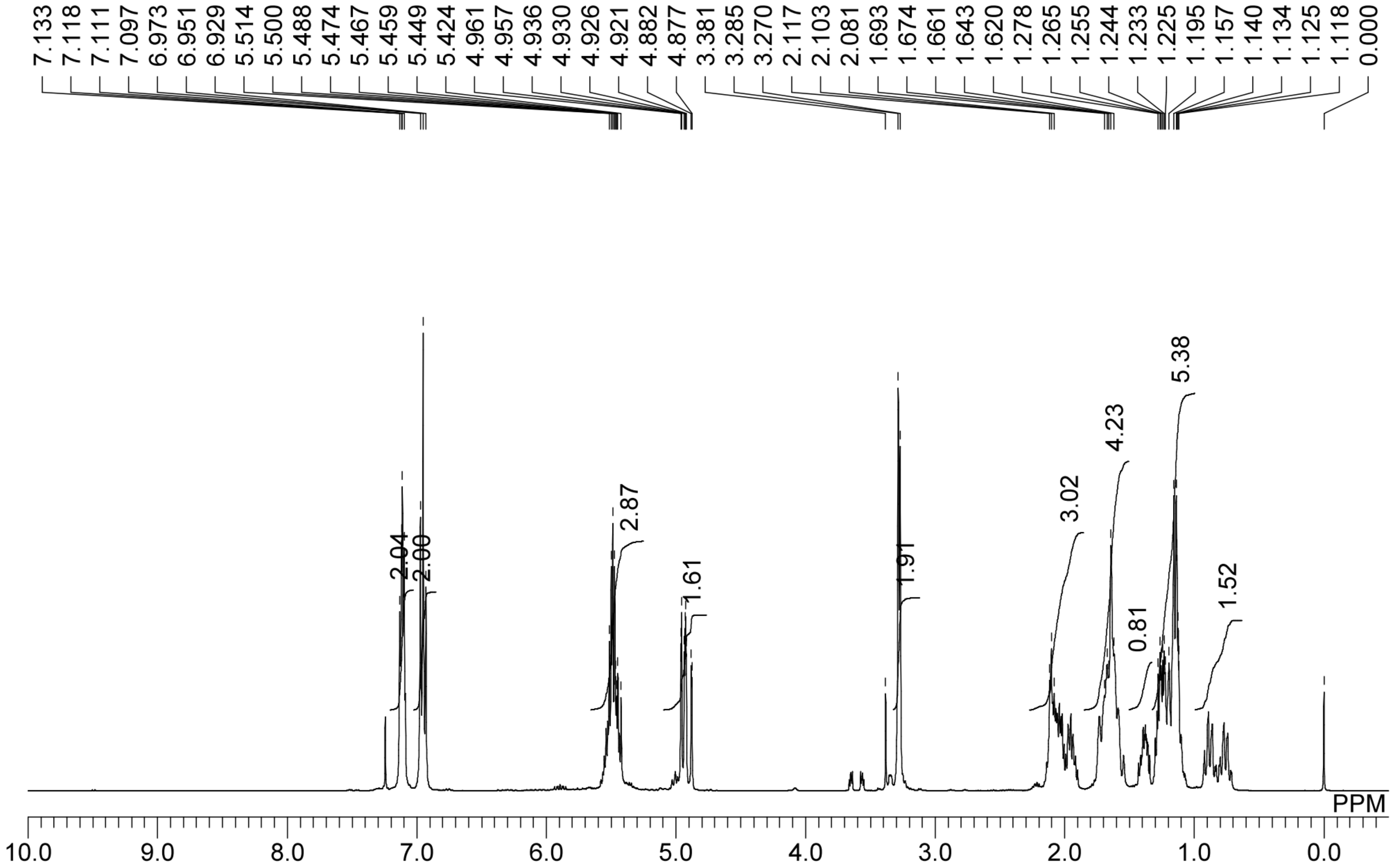

7.133
7.118
7.111
7.097
6.973
6.951
6.929
5.514
5.500
5.488
5.474
5.467
5.459
5.449
5.424
4.961
4.957
4.936
4.930
4.926
4.921
4.882
4.877
3.381
3.285
3.270
2.117
2.103
2.081
1.693
1.674
1.661
1.643
1.620
1.278
1.265
1.255
1.244
1.233
1.225
1.195
1.157
1.140
1.134
1.125
1.118
0.000
2.04
2.00
2.87
1.61
1.91
3.02
4.23
0.81
5.38
1.52
PPM
10.0
9.0
8.0
7.0
6.0
5.0
4.0
3.0
2.0
1.0
0.0

$^{13}C\{^{1}H\}$ NMR of **3ha**

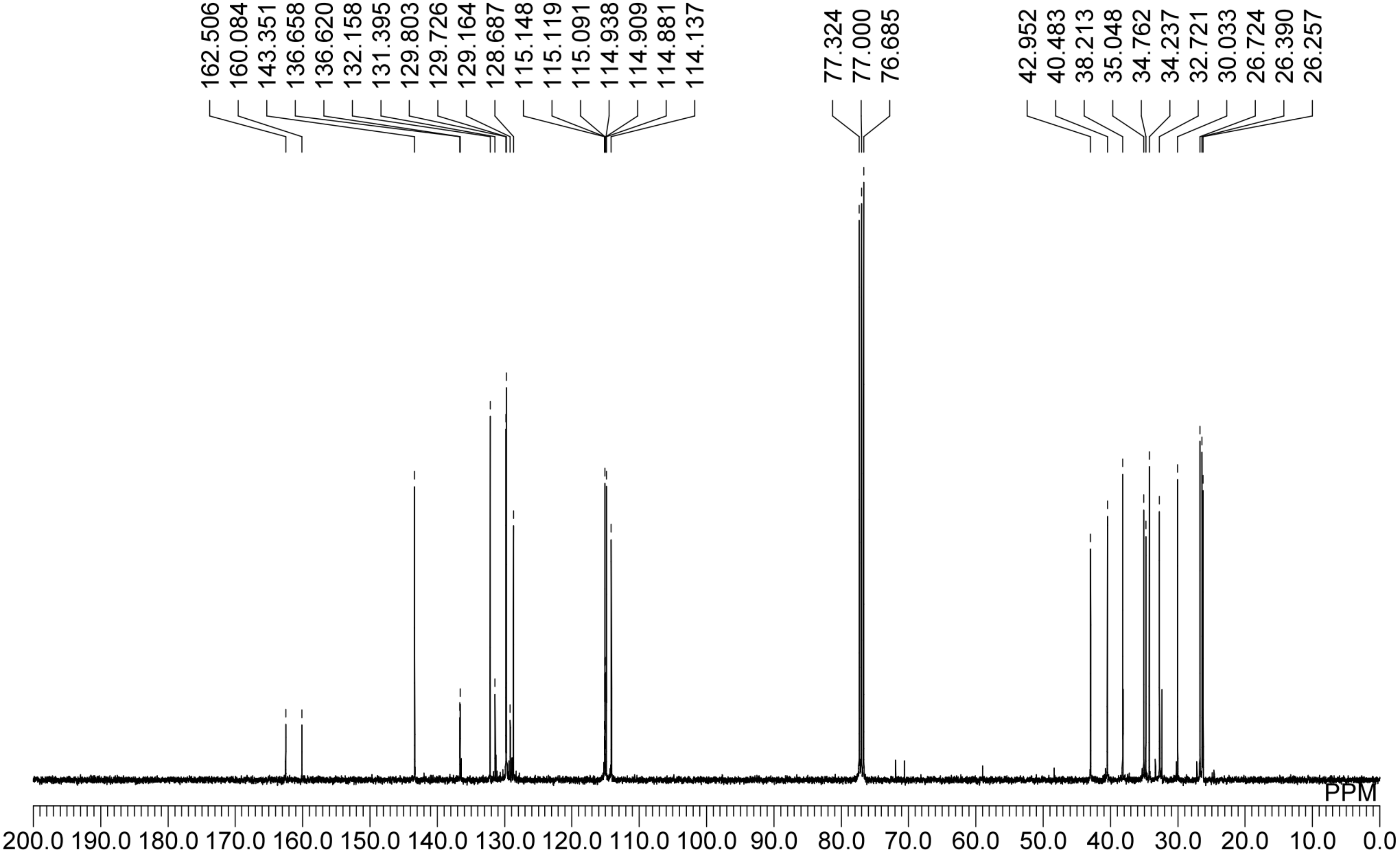

^{1}H NMR of **3ab**

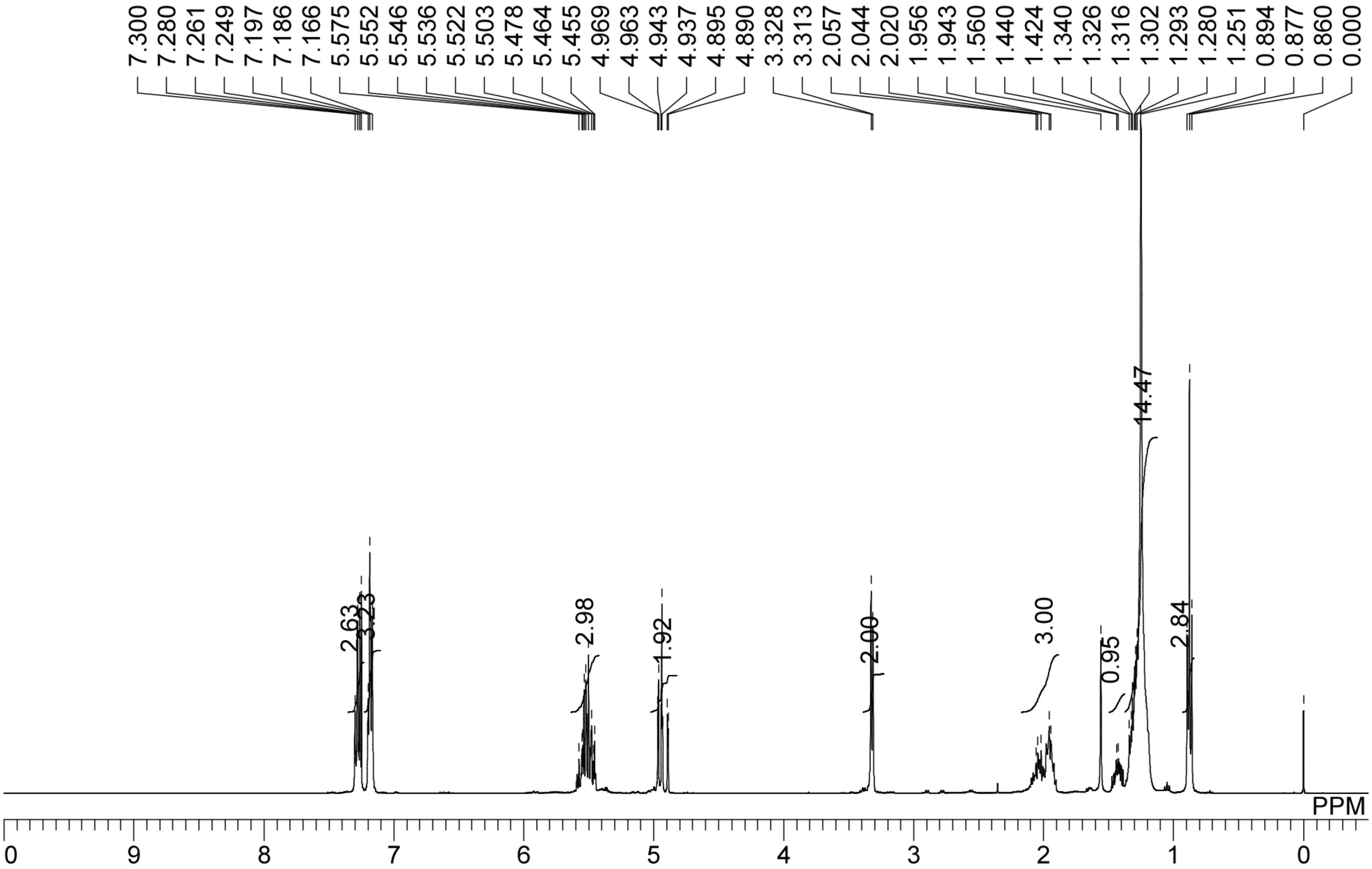
7.300
7.280
7.261
7.249
7.197
7.186
7.166
5.575
5.552
5.546
5.536
5.522
5.503
5.478
5.464
5.455
4.969
4.963
4.943
4.937
4.895
4.890
3.328
3.313
2.057
2.044
2.020
1.956
1.943
1.560
1.440
1.424
1.340
1.326
1.316
1.302
1.293
1.280
1.251
0.894
0.877
0.860
0.000
2.63
3.23
2.98
1.92
2.00
3.00
0.95
14.47
2.84
PPM
10
9
8
7
6
5
4
3
2
1
0

$^{13}C\{^1H\}$ NMR of **3ab**

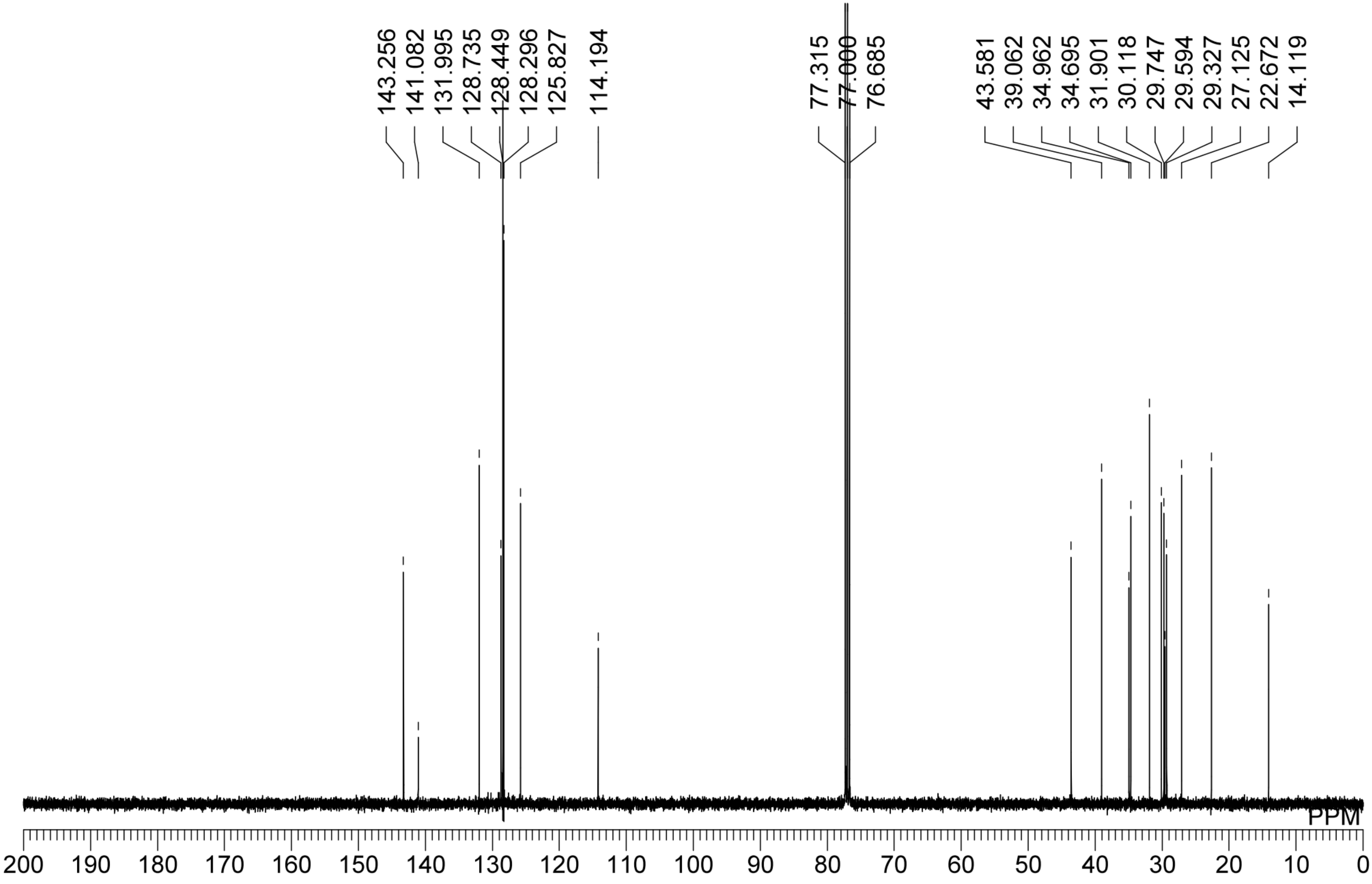

^{1}H NMR of **3ac**

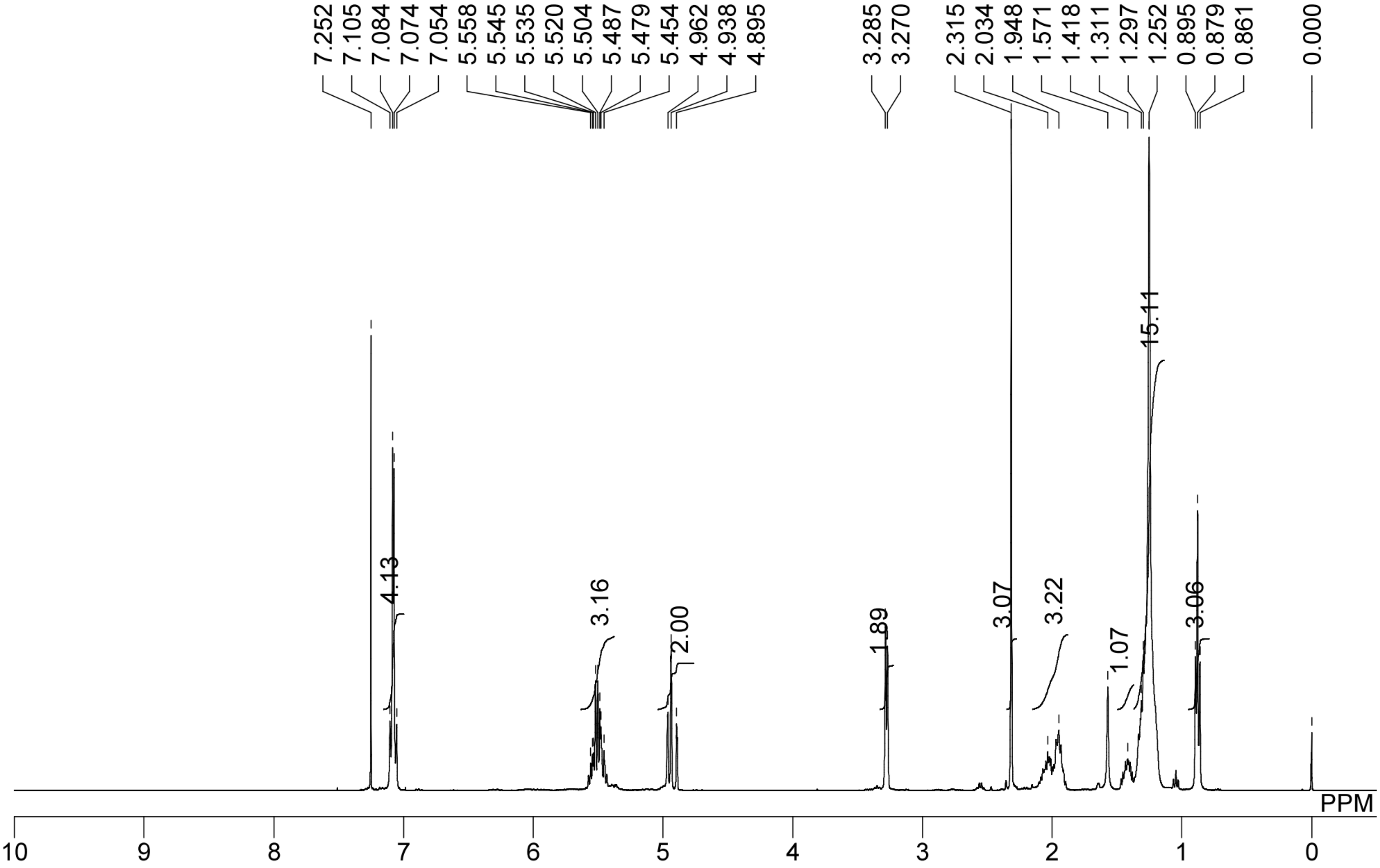

7.252
7.105
7.084
7.074
7.054
5.558
5.545
5.535
5.520
5.504
5.487
5.479
5.454
4.962
4.938
4.895
3.285
3.270
2.315
2.034
1.948
1.571
1.418
1.311
1.297
1.252
0.895
0.879
0.861
0.000
4.13
3.16
2.00
1.89
3.07
3.22
1.07
15.11
3.06
PPM
10
9
8
7
6
5
4
3
2
1
0

$^{13}C\{^1H\}$ NMR of **3ac**

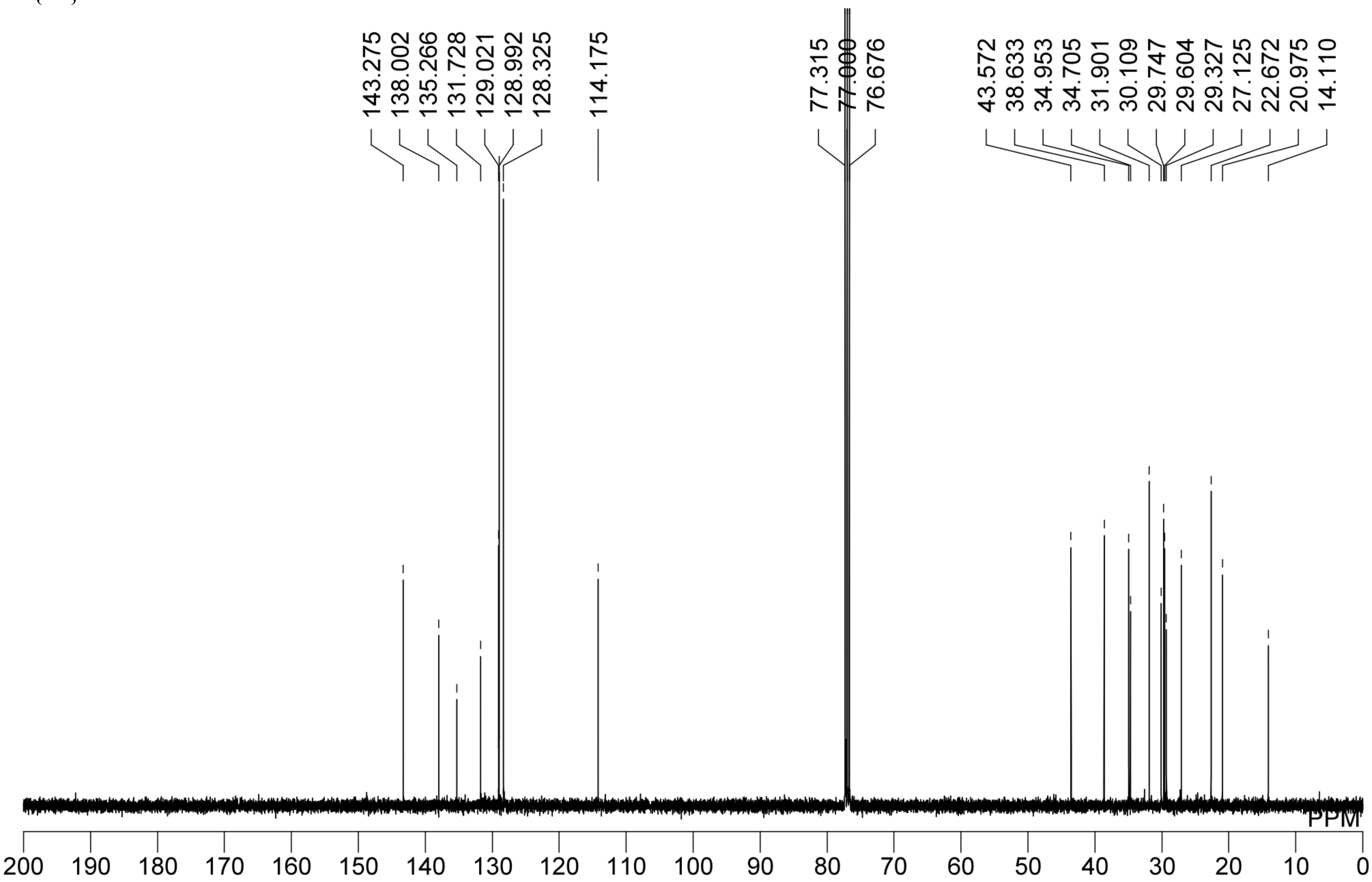

^{1}H NMR of **3ad**

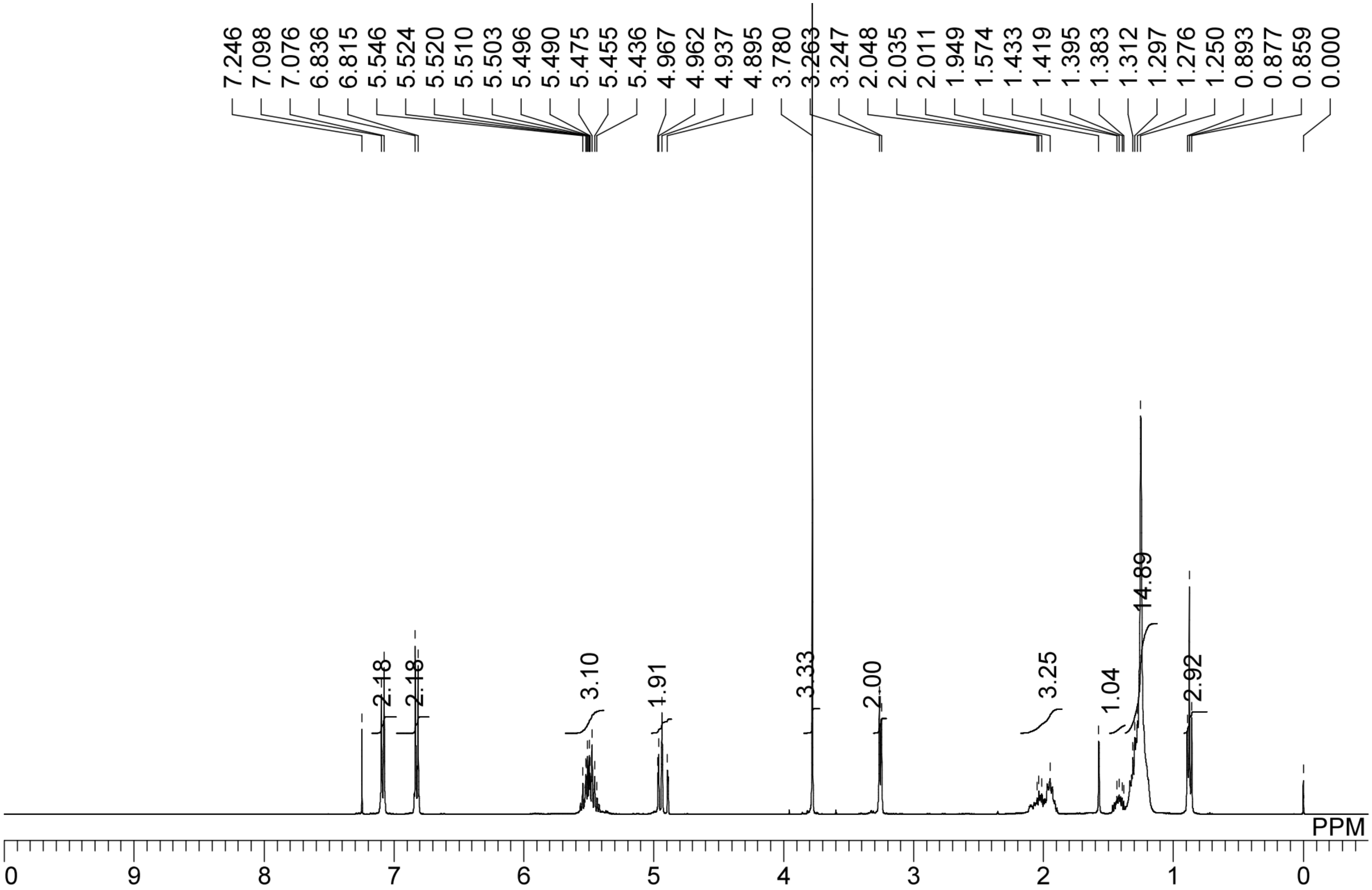

7.246
7.098
7.076
6.836
6.815
5.546
5.524
5.520
5.510
5.503
5.496
5.490
5.475
5.455
5.436
4.967
4.962
4.937
4.895
3.780
3.263
3.247
2.048
2.035
2.011
1.949
1.574
1.433
1.419
1.395
1.383
1.312
1.297
1.276
1.250
0.893
0.877
0.859
0.000
2.18
2.18
3.10
1.91
3.33
2.00
3.25
1.04
14.89
2.92
PPM
10
9
8
7
6
5
4
3
2
1
0

$^{13}C\{^{1}H\}$ NMR of **3ad**

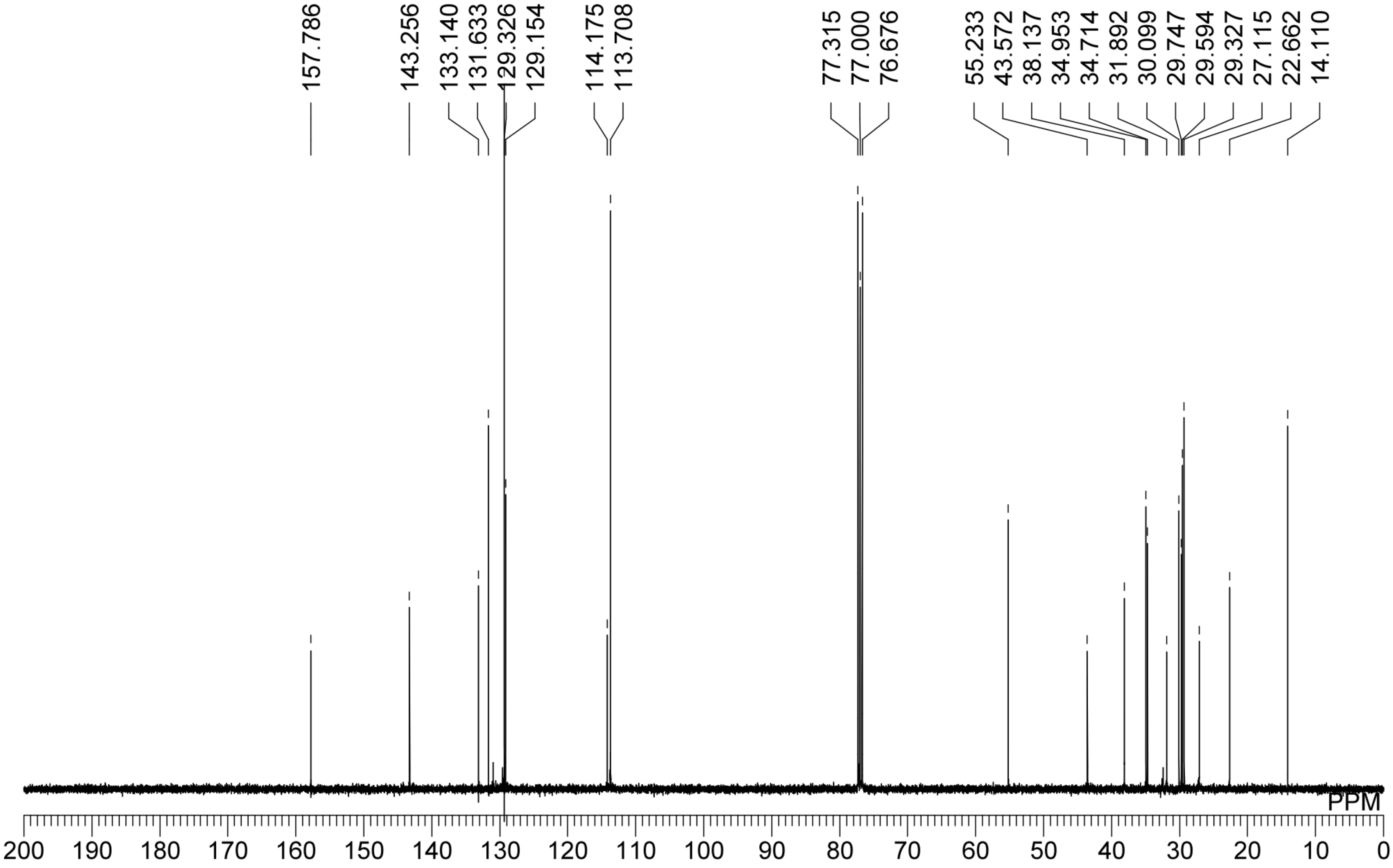

^{1}H NMR of **3ae**

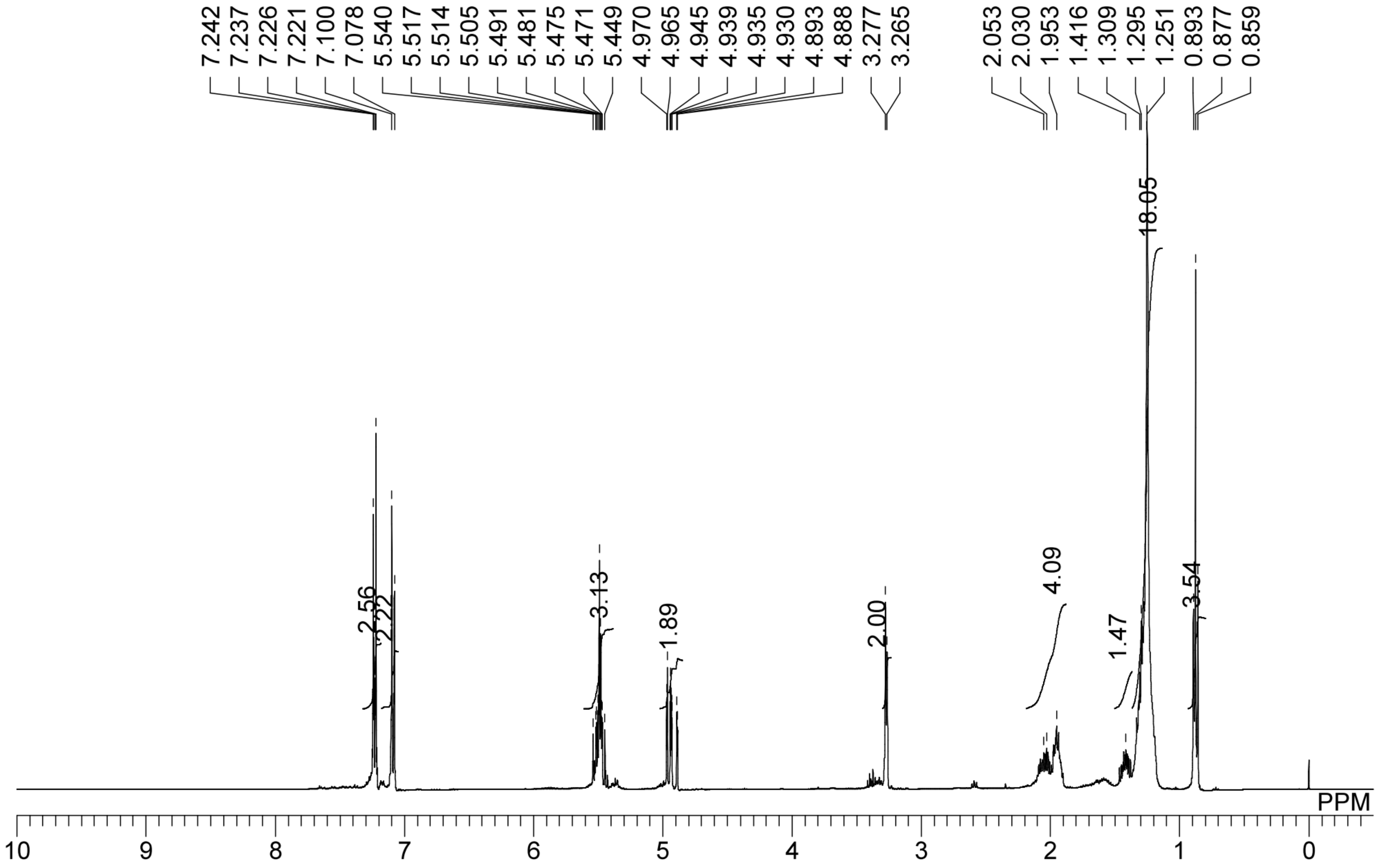
7.242
7.237
7.226
7.221
7.100
7.078
5.540
5.517
5.514
5.505
5.491
5.481
5.475
5.471
5.449
4.970
4.965
4.945
4.939
4.935
4.930
4.893
4.888
3.277
3.265
2.053
2.030
1.953
1.416
1.309
1.295
1.251
0.893
0.877
0.859
18.05
2.56
2.22
3.13
1.89
2.00
4.09
1.47
3.54
PPM
10
9
8
7
6
5
4
3
2
1
0

$^{13}C\{^{1}H\}$ NMR of **3ae**

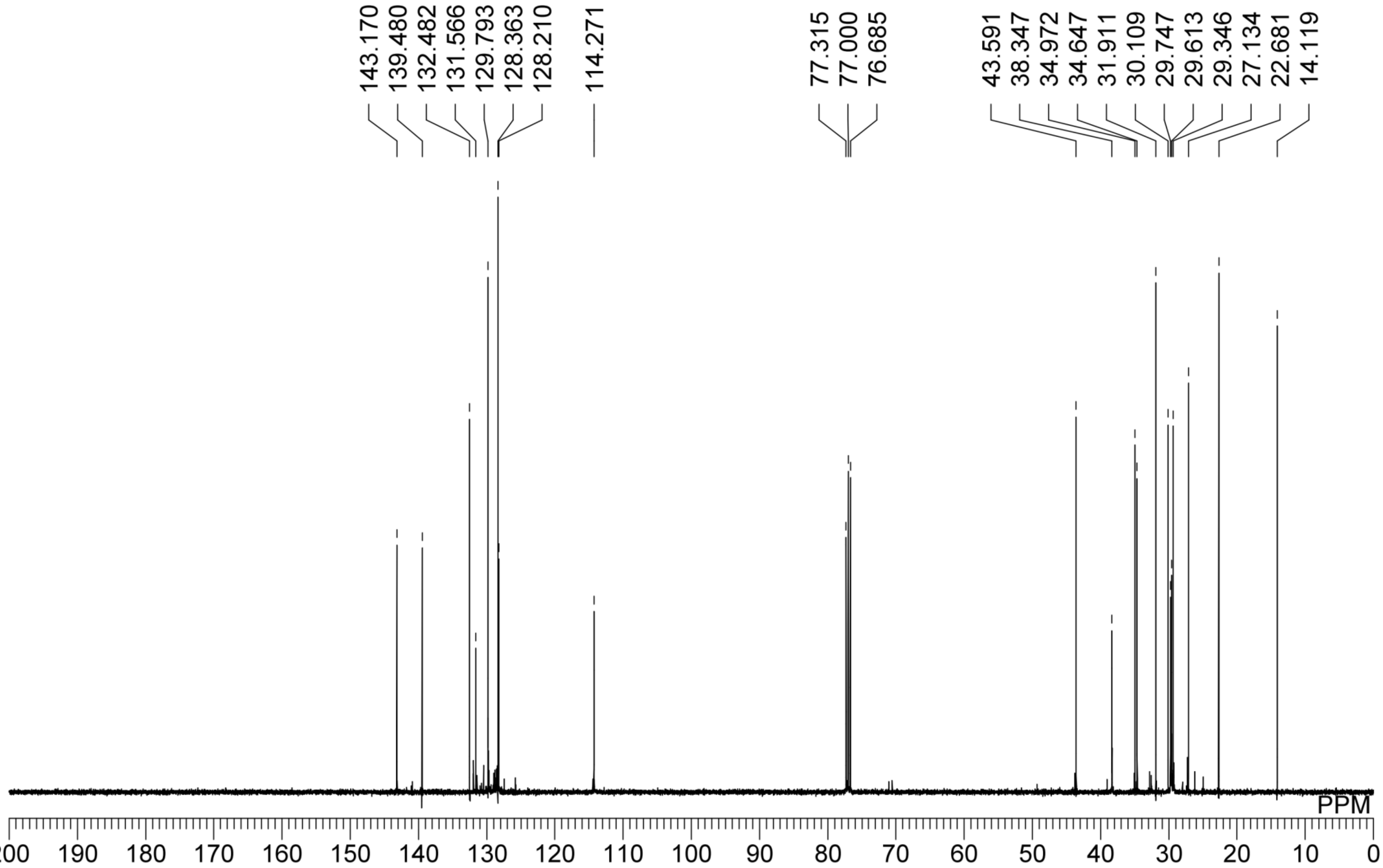

^{1}H NMR of **3af**

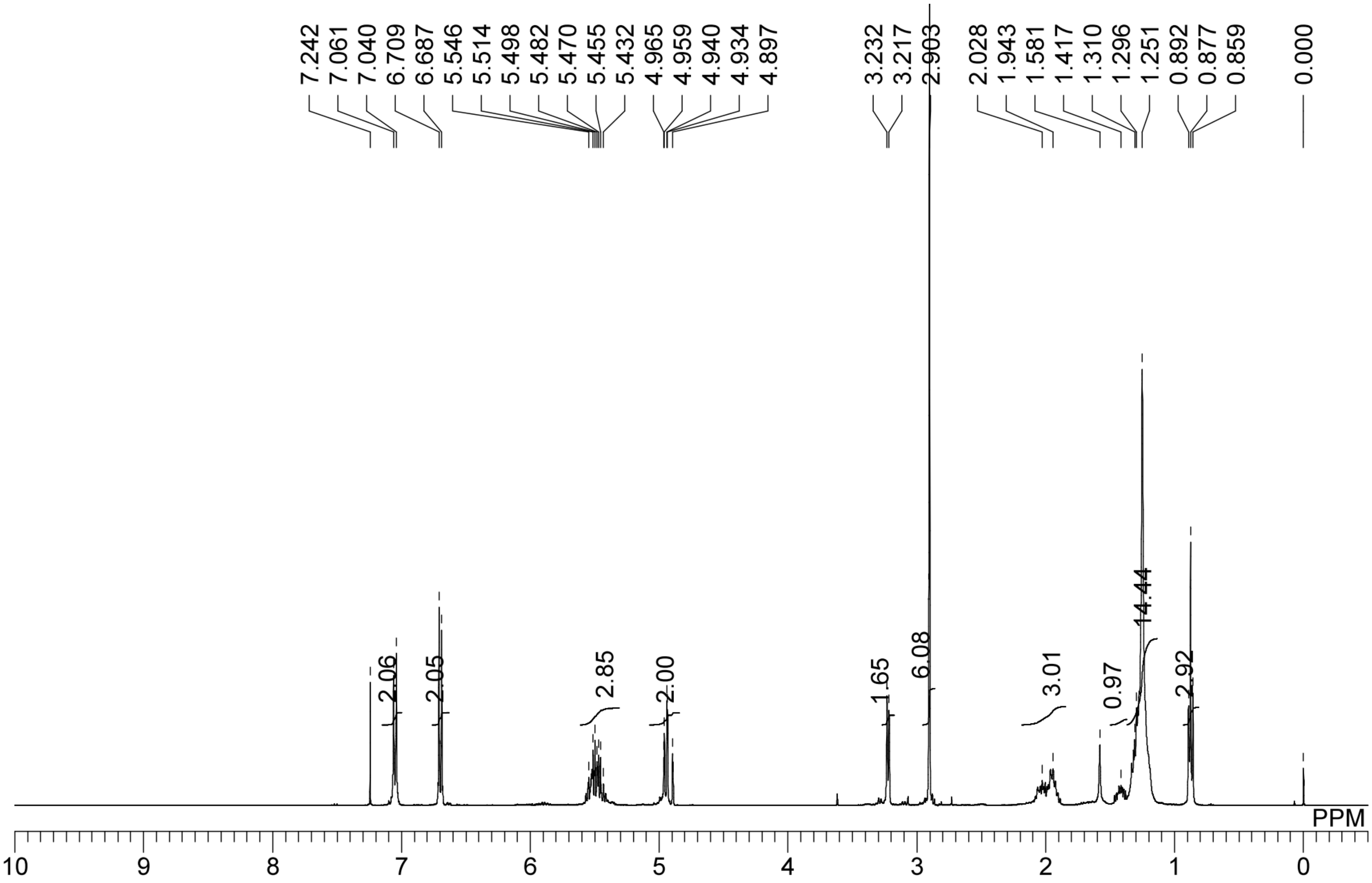

7.242
7.061
7.040
6.709
6.687
5.546
5.514
5.498
5.482
5.470
5.455
5.432
4.965
4.959
4.940
4.934
4.897
3.232
3.217
2.903
2.028
1.943
1.581
1.417
1.310
1.296
1.251
0.892
0.877
0.859
0.000
2.06
2.05
2.85
2.00
1.65
6.08
3.01
0.97
14.44
2.92
PPM
10
9
8
7
6
5
4
3
2
1
0

$^{13}C\{^1H\}$ NMR of **3af**

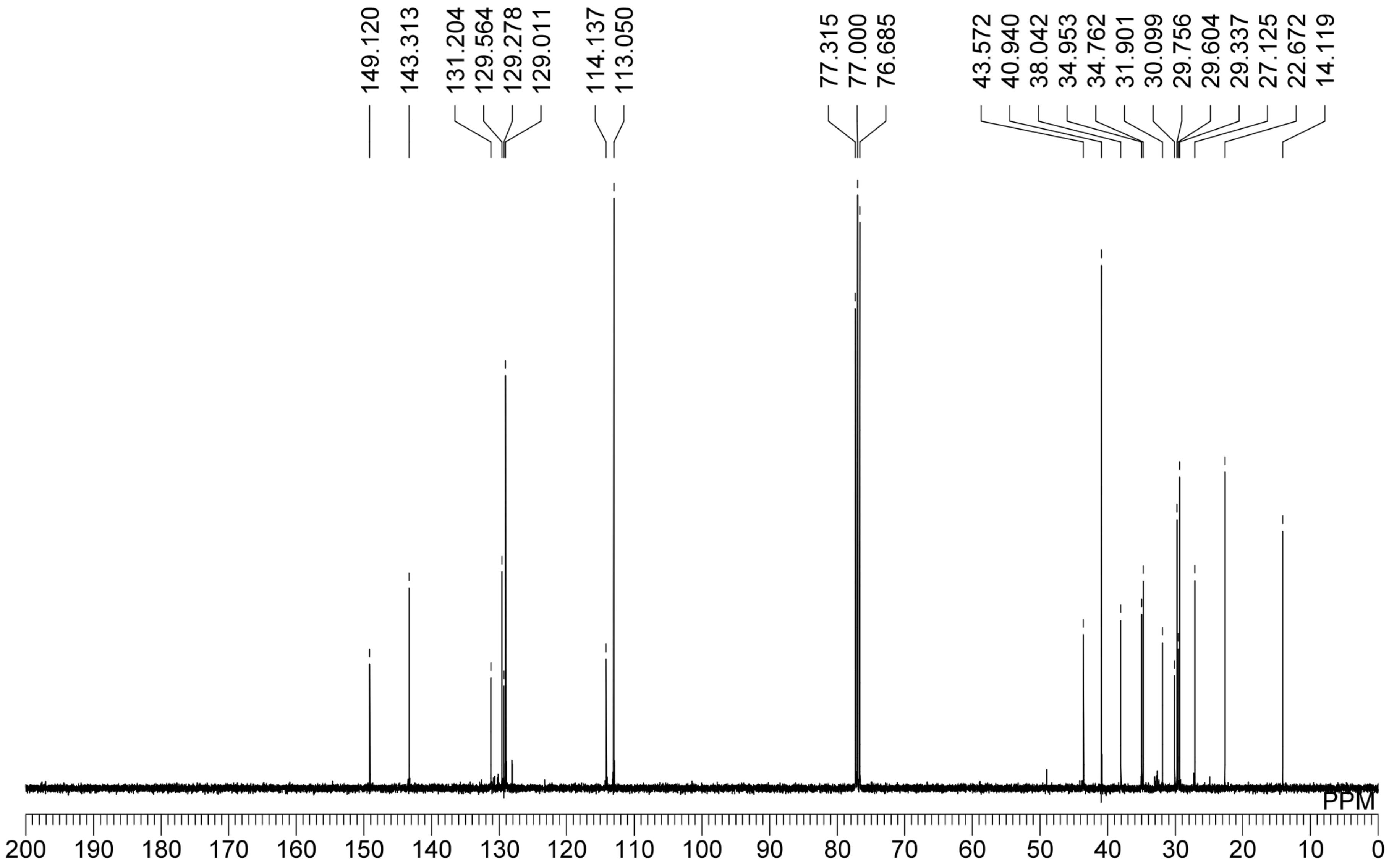

^{1}H NMR of **3ag**

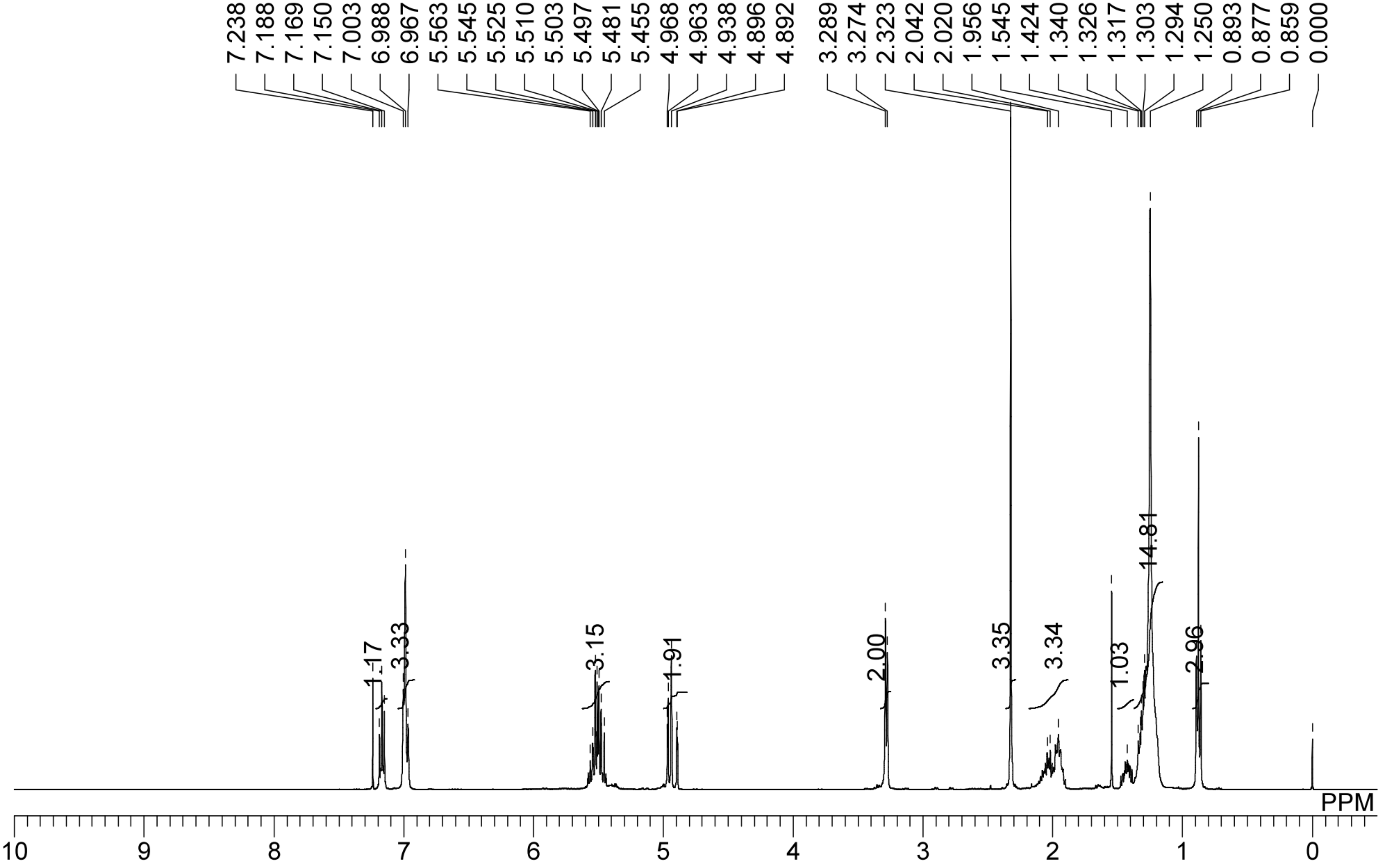

7.238
7.188
7.169
7.150
7.003
6.988
6.967
5.563
5.545
5.525
5.510
5.503
5.497
5.481
5.455
4.968
4.963
4.938
4.896
4.892
3.289
3.274
2.323
2.042
2.020
1.956
1.545
1.424
1.340
1.326
1.317
1.303
1.294
1.250
0.893
0.877
0.859
0.000
1.17
3.33
3.15
1.91
2.00
3.35
3.34
1.03
14.81
2.96
PPM
10
9
8
7
6
5
4
3
2
1
0

$^{13}C\{^{1}H\}$ NMR of **3ag**

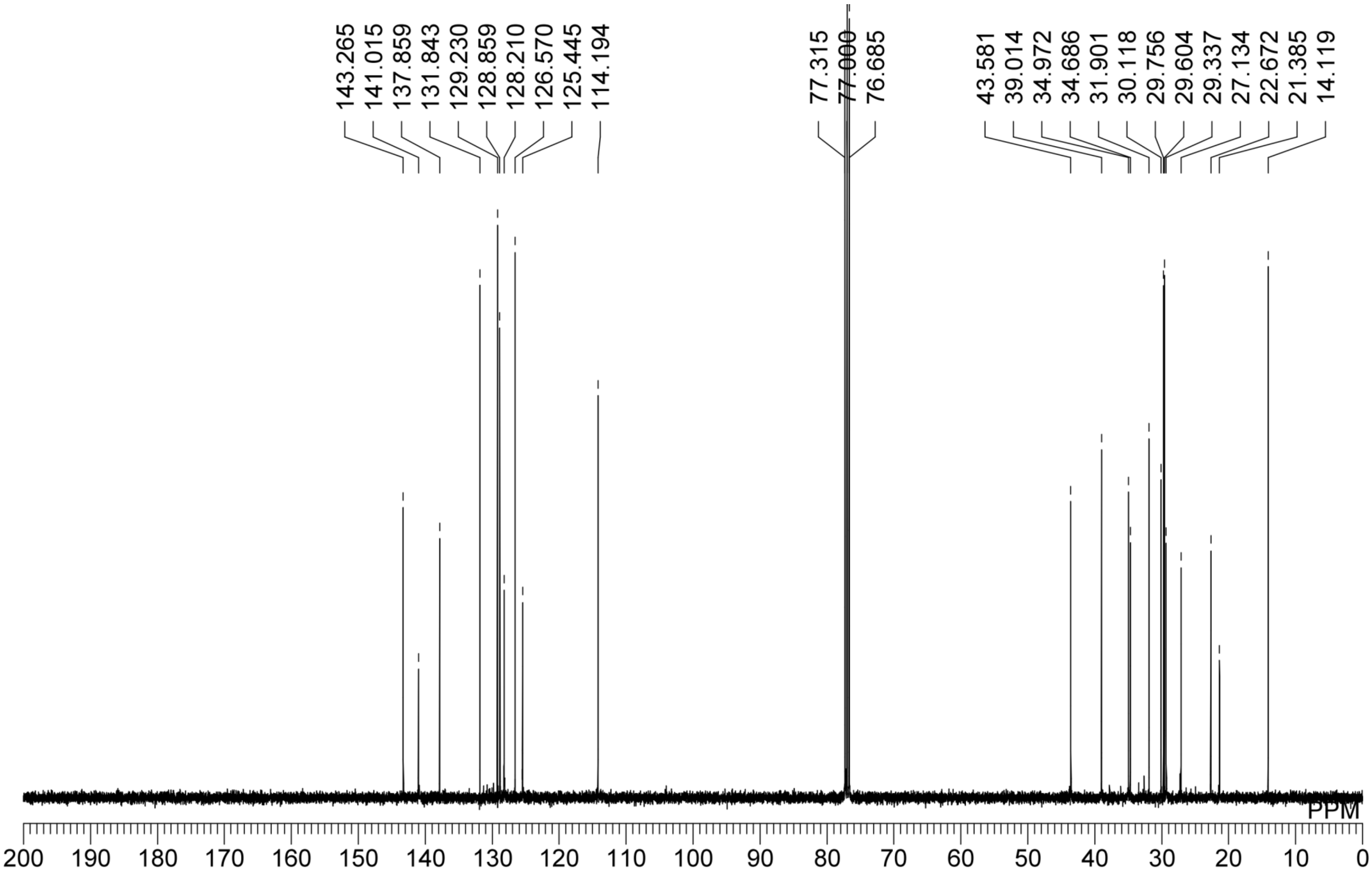

^{1}H NMR of **3ah**

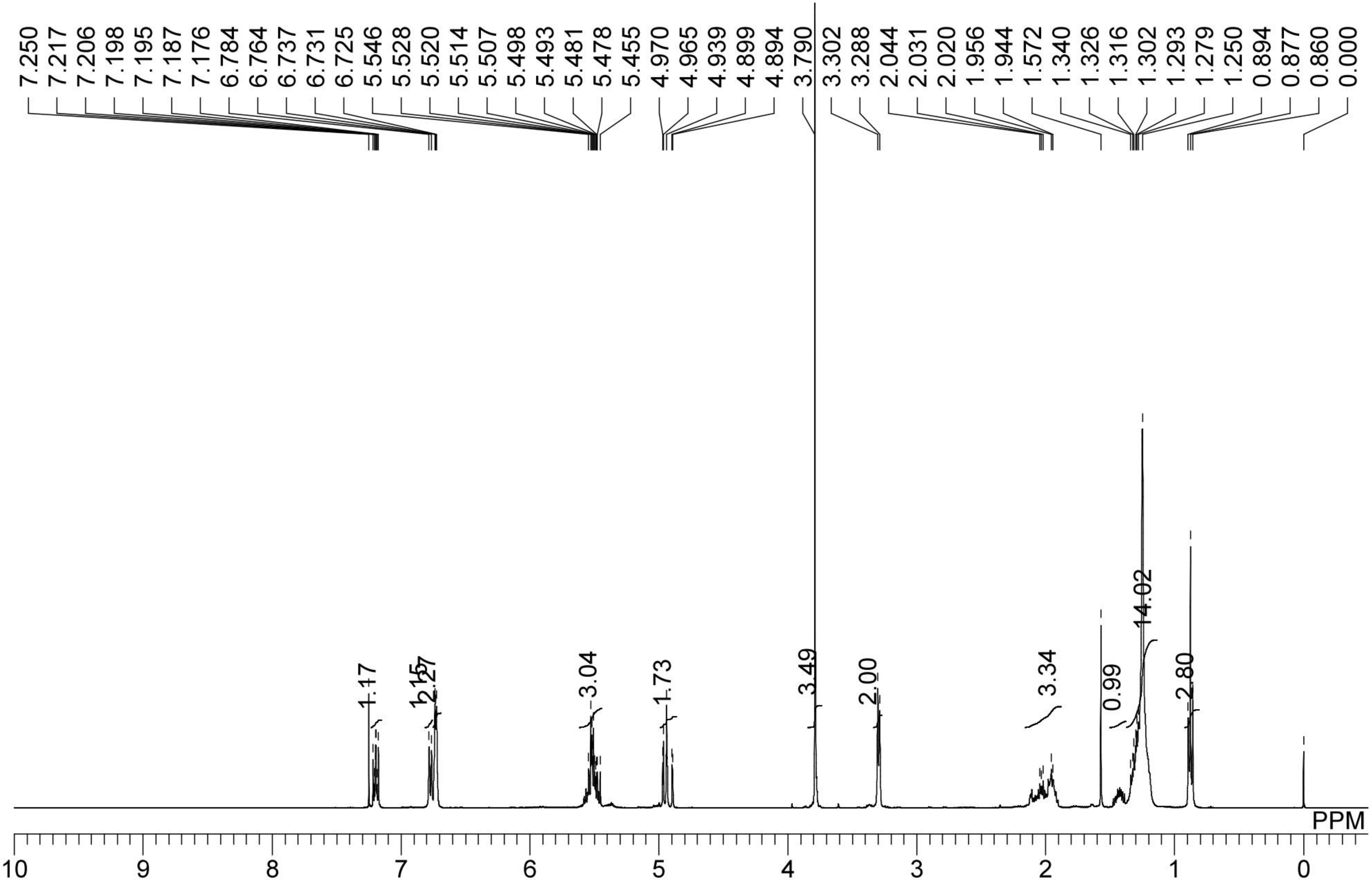
7.250
7.217
7.206
7.198
7.195
7.187
7.176
6.784
6.764
6.737
6.731
6.725
5.546
5.528
5.520
5.514
5.507
5.498
5.493
5.481
5.478
5.455
4.970
4.965
4.939
4.899
4.894
3.790
3.302
3.288
2.044
2.031
2.020
1.956
1.944
1.572
1.340
1.326
1.316
1.302
1.293
1.279
1.250
0.894
0.877
0.860
0.000
1.17
1.15
2.27
3.04
1.73
3.49
2.00
3.34
0.99
14.02
2.80
PPM
10
9
8
7
6
5
4
3
2
1
0

$^{13}C\{^{1}H\}$ NMR of **3ah**

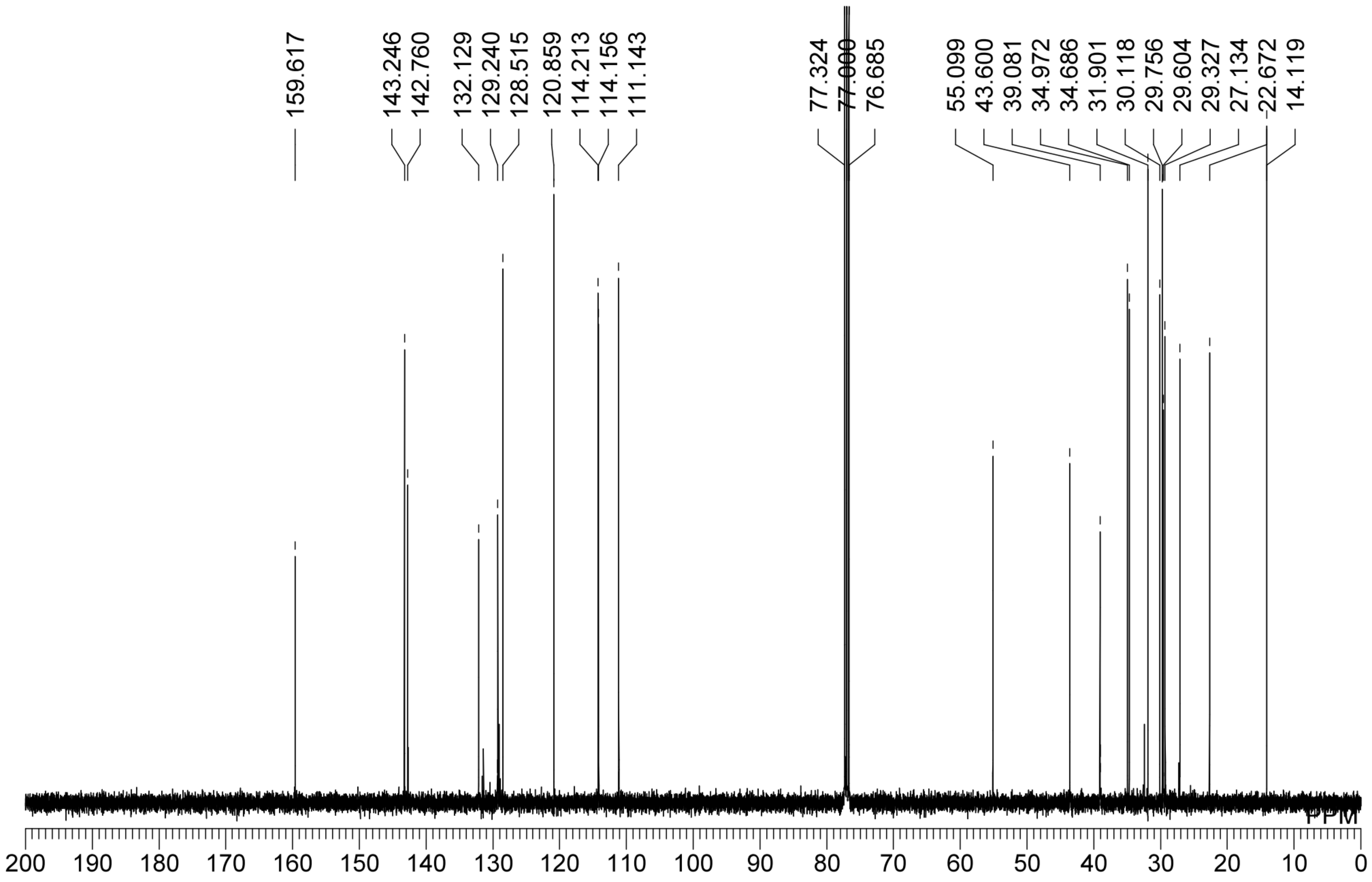

^1H NMR of **3gb**

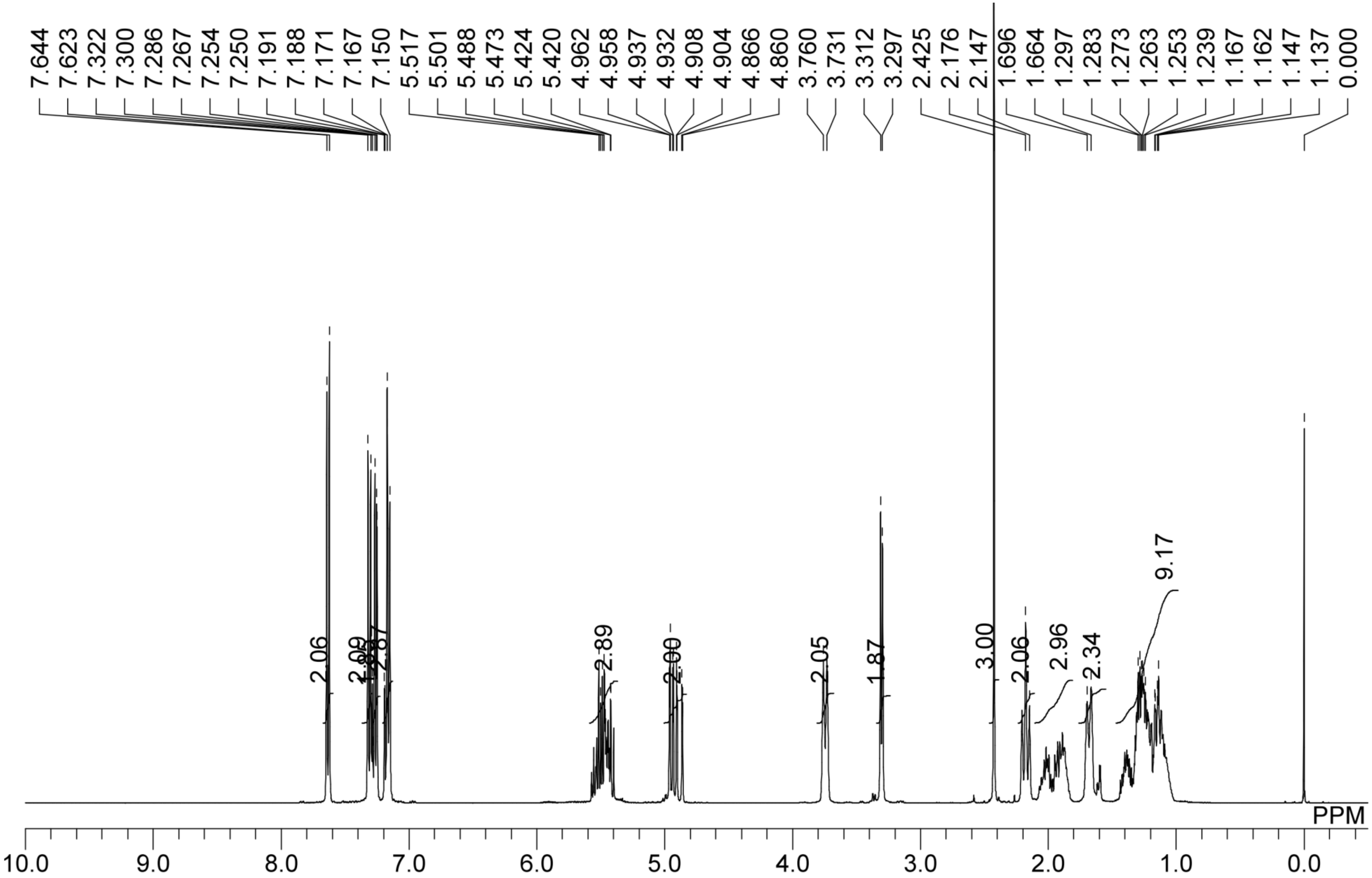
7.644
7.623
7.322
7.300
7.286
7.267
7.254
7.250
7.191
7.188
7.171
7.167
7.150
5.517
5.501
5.488
5.473
5.424
5.420
4.962
4.958
4.937
4.932
4.908
4.904
4.866
4.860
3.760
3.731
3.312
3.297
2.425
2.176
2.147
1.696
1.664
1.297
1.283
1.273
1.263
1.253
1.239
1.167
1.162
1.147
1.137
0.000
2.06
2.89
2.00
2.05
1.87
3.00
2.06
2.96
2.34
9.17
PPM
10.0
9.0
8.0
7.0
6.0
5.0
4.0
3.0
2.0
1.0
0.0

$^{13}C\{^1H\}$ NMR of **3gb**

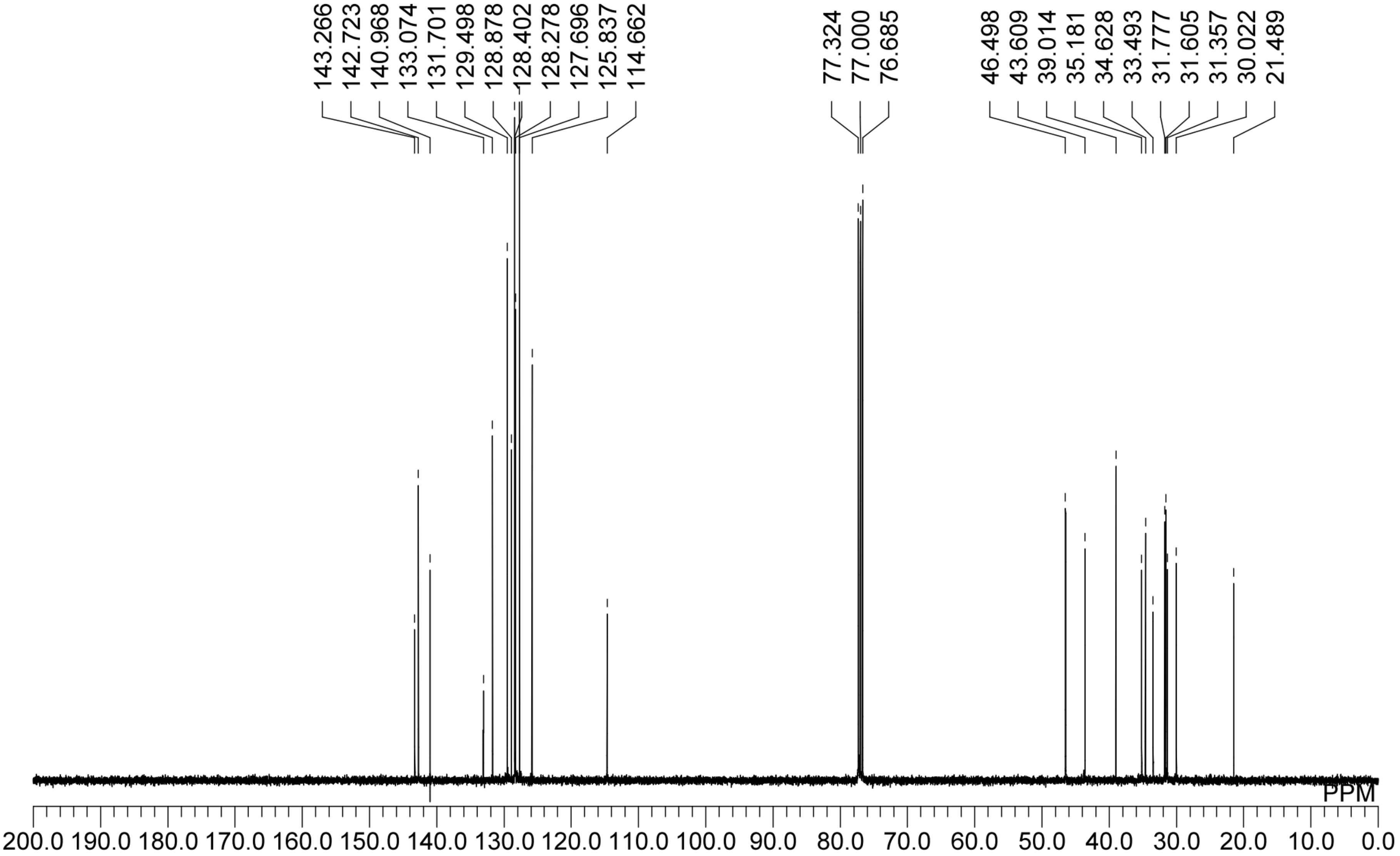

^{1}H NMR of **3gc**

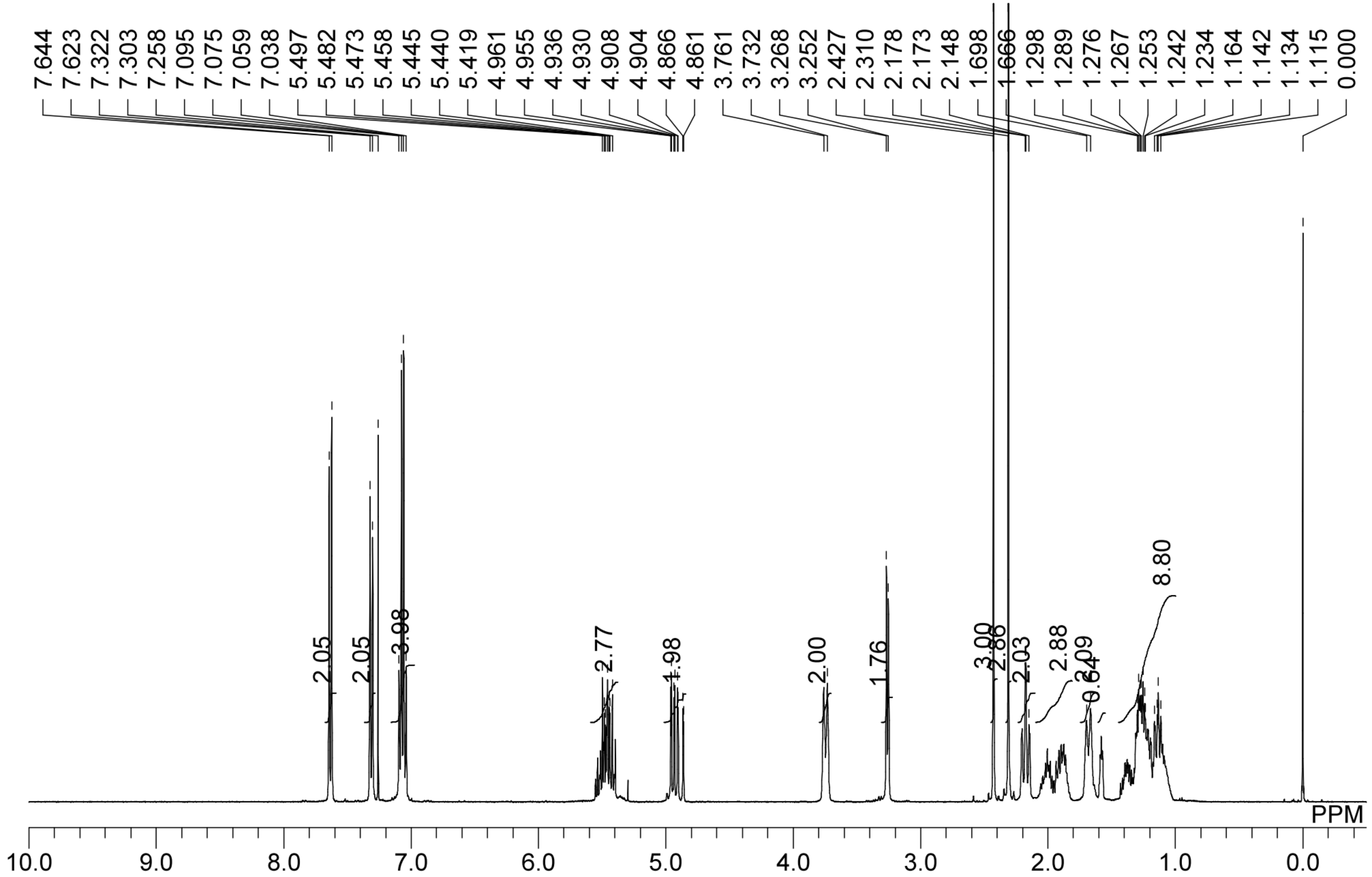
7.644
7.623
7.322
7.303
7.258
7.095
7.075
7.059
7.038
5.497
5.482
5.473
5.458
5.445
5.440
5.419
4.961
4.955
4.936
4.930
4.908
4.904
4.866
4.861
3.761
3.732
3.268
3.252
2.427
2.310
2.178
2.173
2.148
1.698
1.666
1.298
1.289
1.276
1.267
1.253
1.242
1.234
1.164
1.142
1.134
1.115
0.000
2.05
2.05
3.98
2.77
1.98
2.00
1.76
3.00
2.86
2.03
2.88
2.09
0.64
8.80
PPM
10.0
9.0
8.0
7.0
6.0
5.0
4.0
3.0
2.0
1.0
0.0

$^{13}C\{^{1}H\}$ NMR of **3gc**

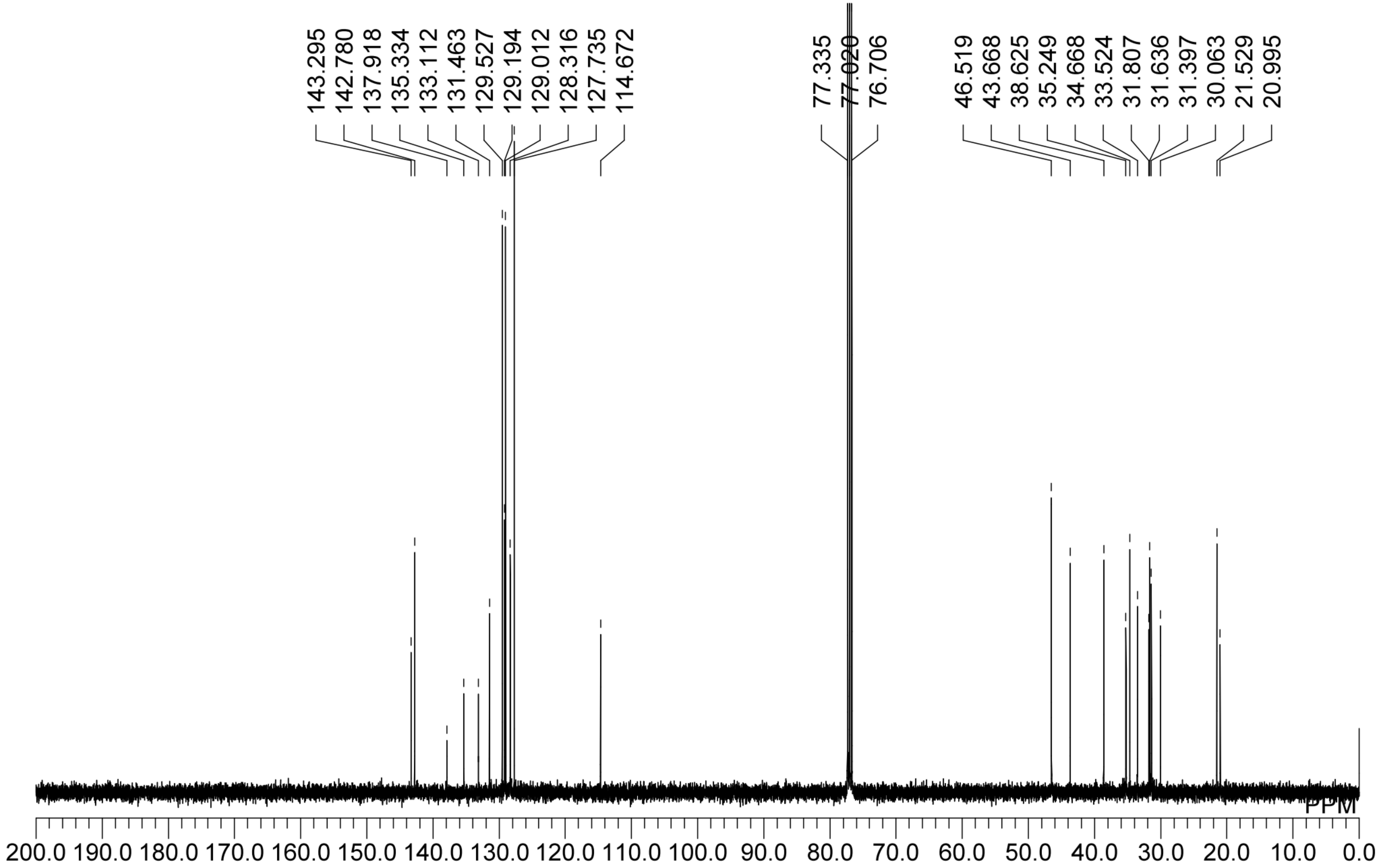

^{1}H NMR of **3gg**

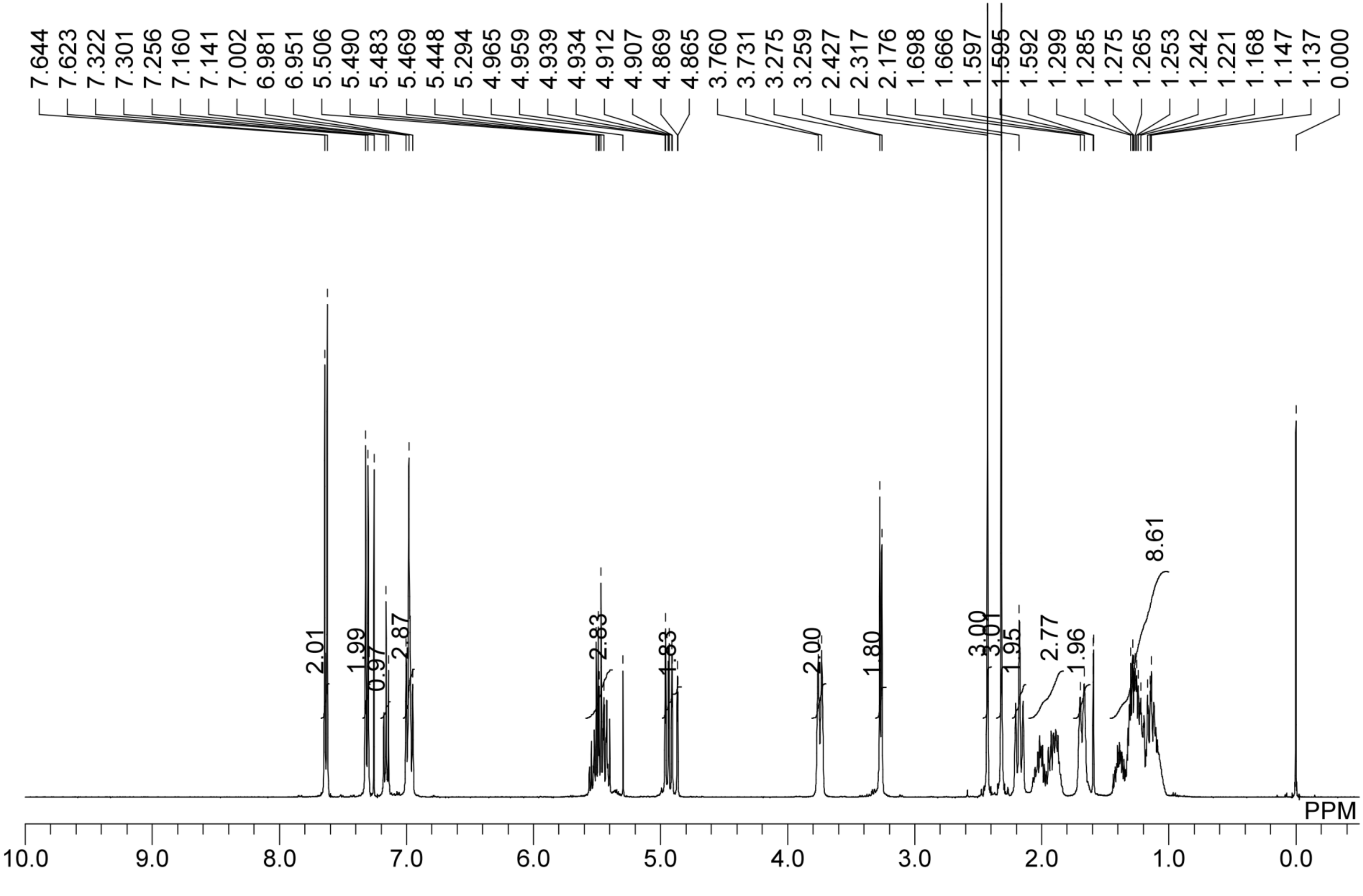
7.644
7.623
7.322
7.301
7.256
7.160
7.141
7.002
6.981
6.951
5.506
5.490
5.483
5.469
5.448
5.294
4.965
4.959
4.939
4.934
4.912
4.907
4.869
4.865
3.760
3.731
3.275
3.259
2.427
2.317
2.176
1.698
1.666
1.597
1.595
1.592
1.299
1.285
1.275
1.265
1.253
1.242
1.221
1.168
1.147
1.137
0.000
2.01
1.99
0.97
2.87
2.83
1.83
2.00
1.80
3.00
3.01
1.95
2.77
1.96
8.61
PPM
10.0
9.0
8.0
7.0
6.0
5.0
4.0
3.0
2.0
1.0
0.0

$^{13}C\{^1H\}$ NMR of **3gg**

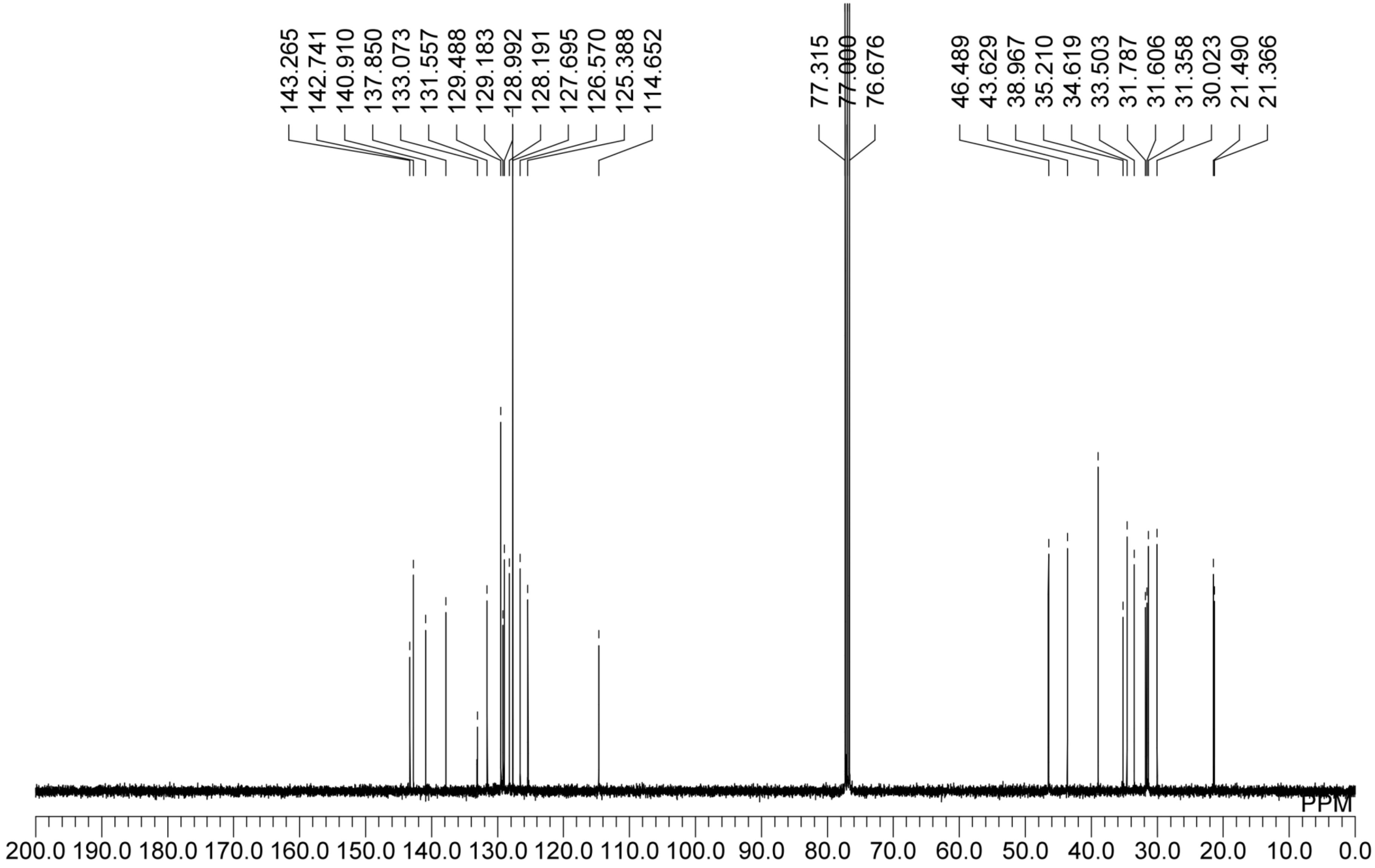

^{1}H NMR of **3gj**

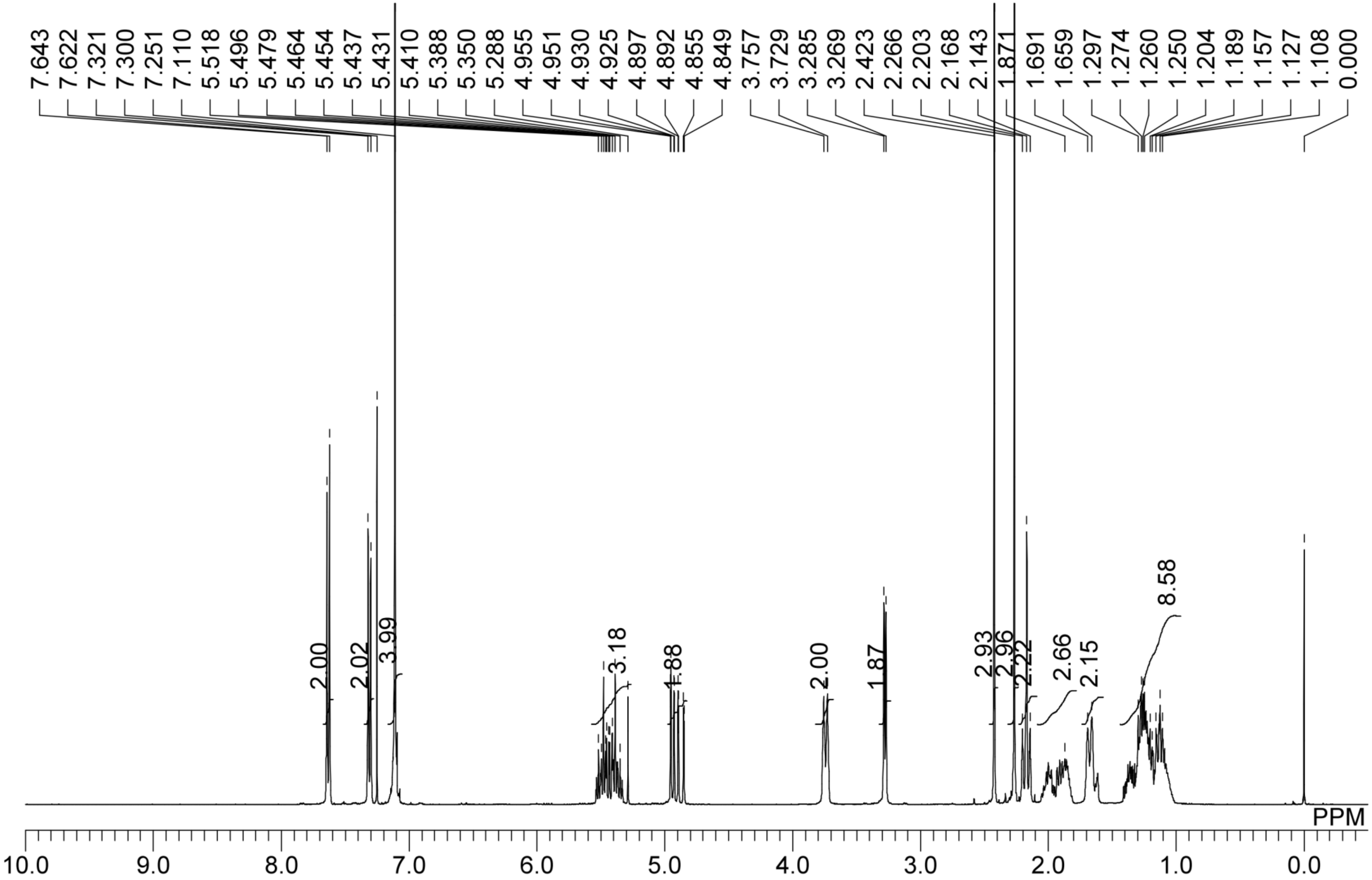
7.643
7.622
7.321
7.300
7.251
7.110
5.518
5.496
5.479
5.464
5.454
5.437
5.431
5.410
5.388
5.350
5.288
4.955
4.951
4.930
4.925
4.897
4.892
4.855
4.849
3.757
3.729
3.285
3.269
2.423
2.266
2.203
2.168
2.143
1.871
1.691
1.659
1.297
1.274
1.260
1.250
1.204
1.189
1.157
1.127
1.108
0.000
2.00
2.02
3.99
3.18
1.88
2.00
1.87
2.93
2.96
2.22
2.66
2.15
8.58
PPM
10.0
9.0
8.0
7.0
6.0
5.0
4.0
3.0
2.0
1.0
0.0

$^{13}C\{^{1}H\}$ NMR of **3gj**

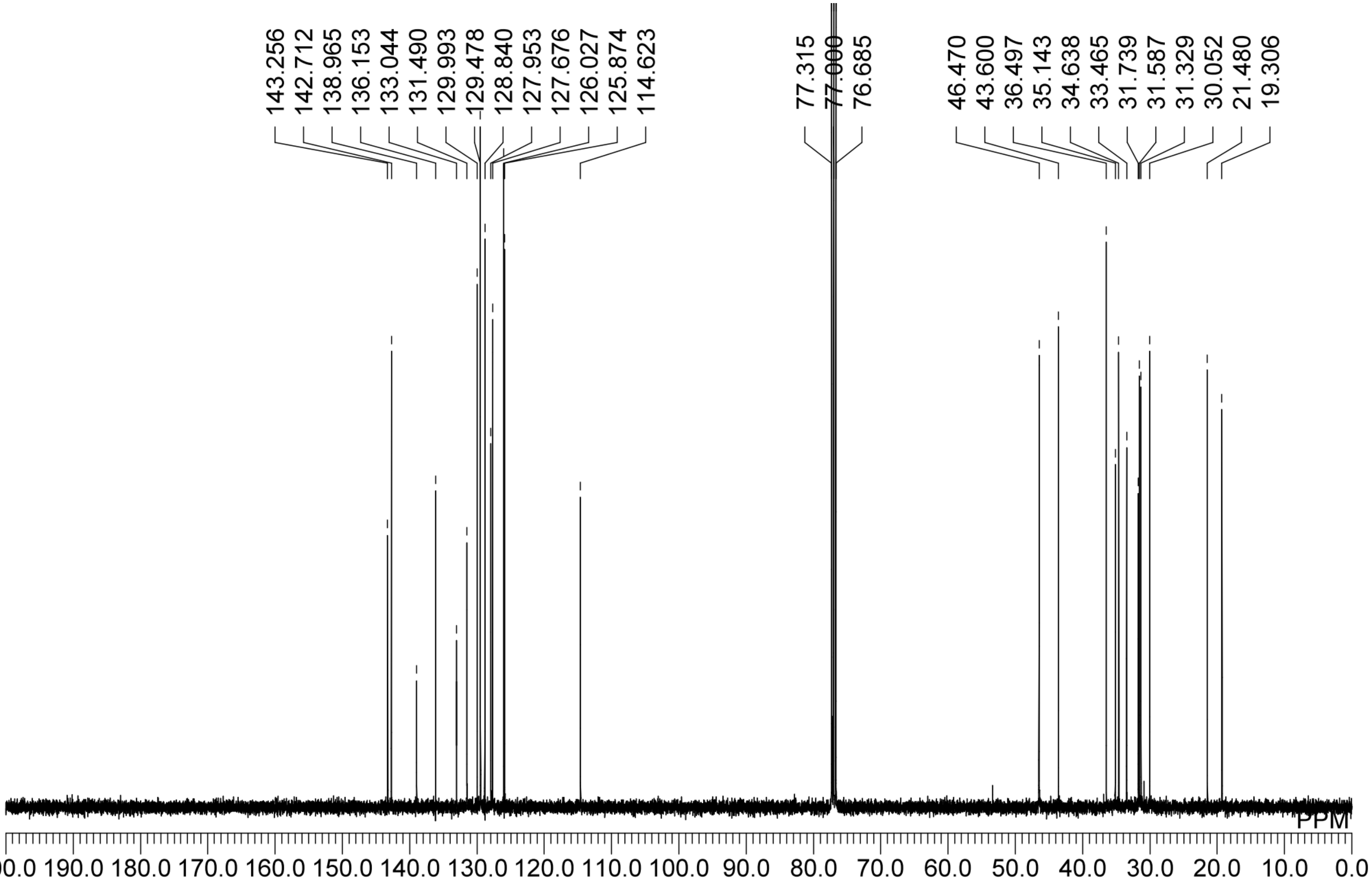

^{1}H NMR of **3na**

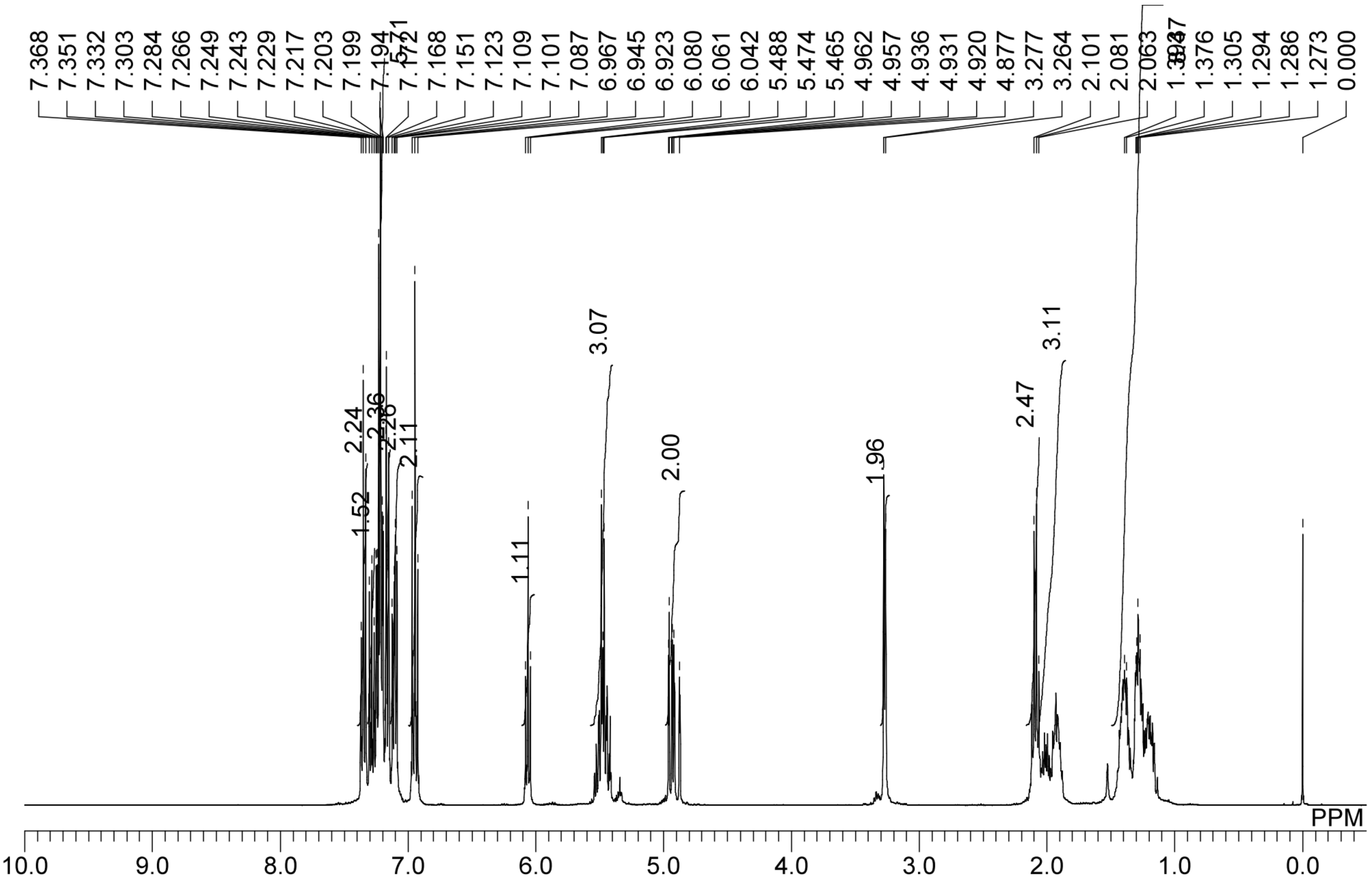

$^{13}C\{^{1}H\}$ NMR of **3na**

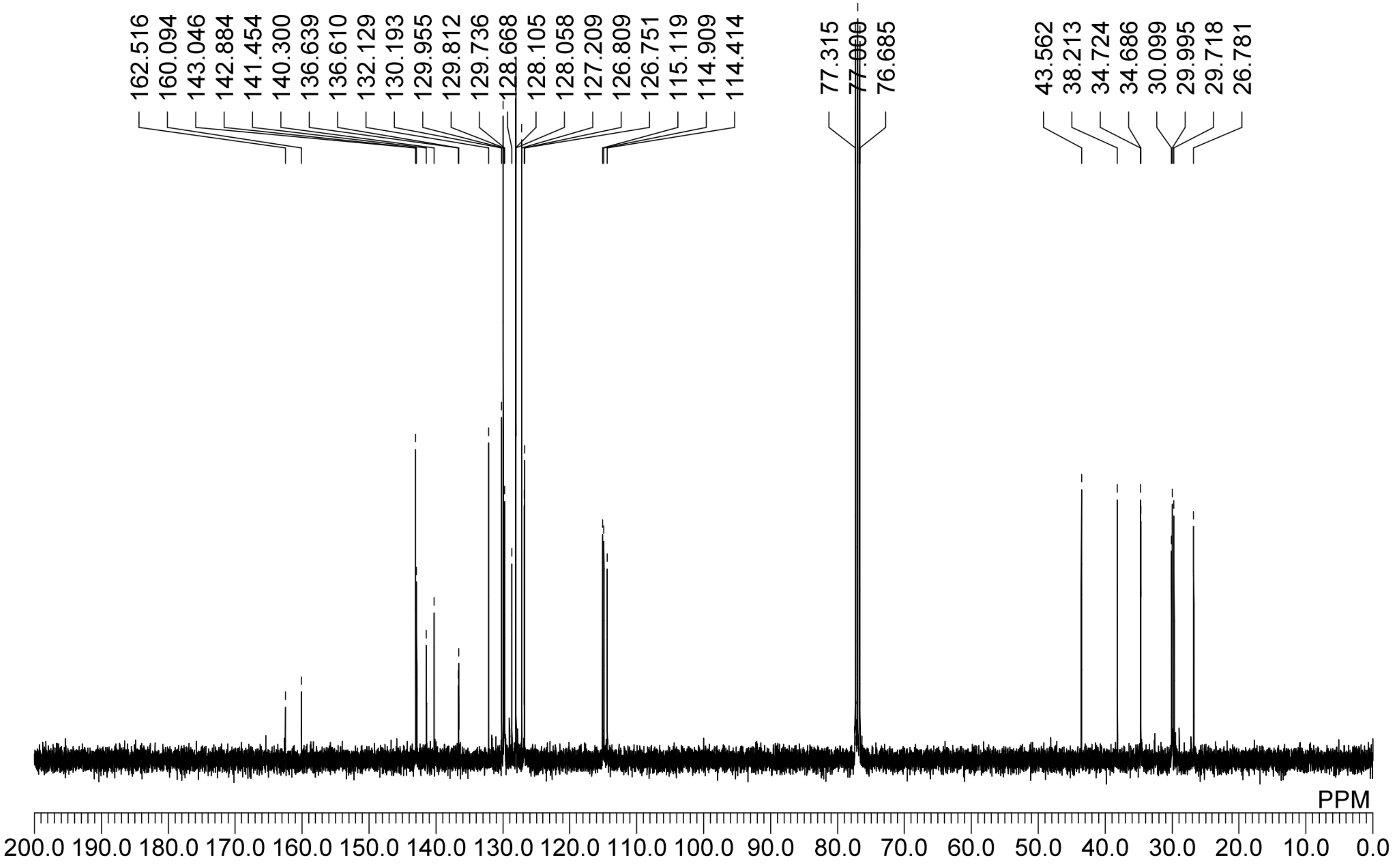

^{1}H NMR of **4ba**

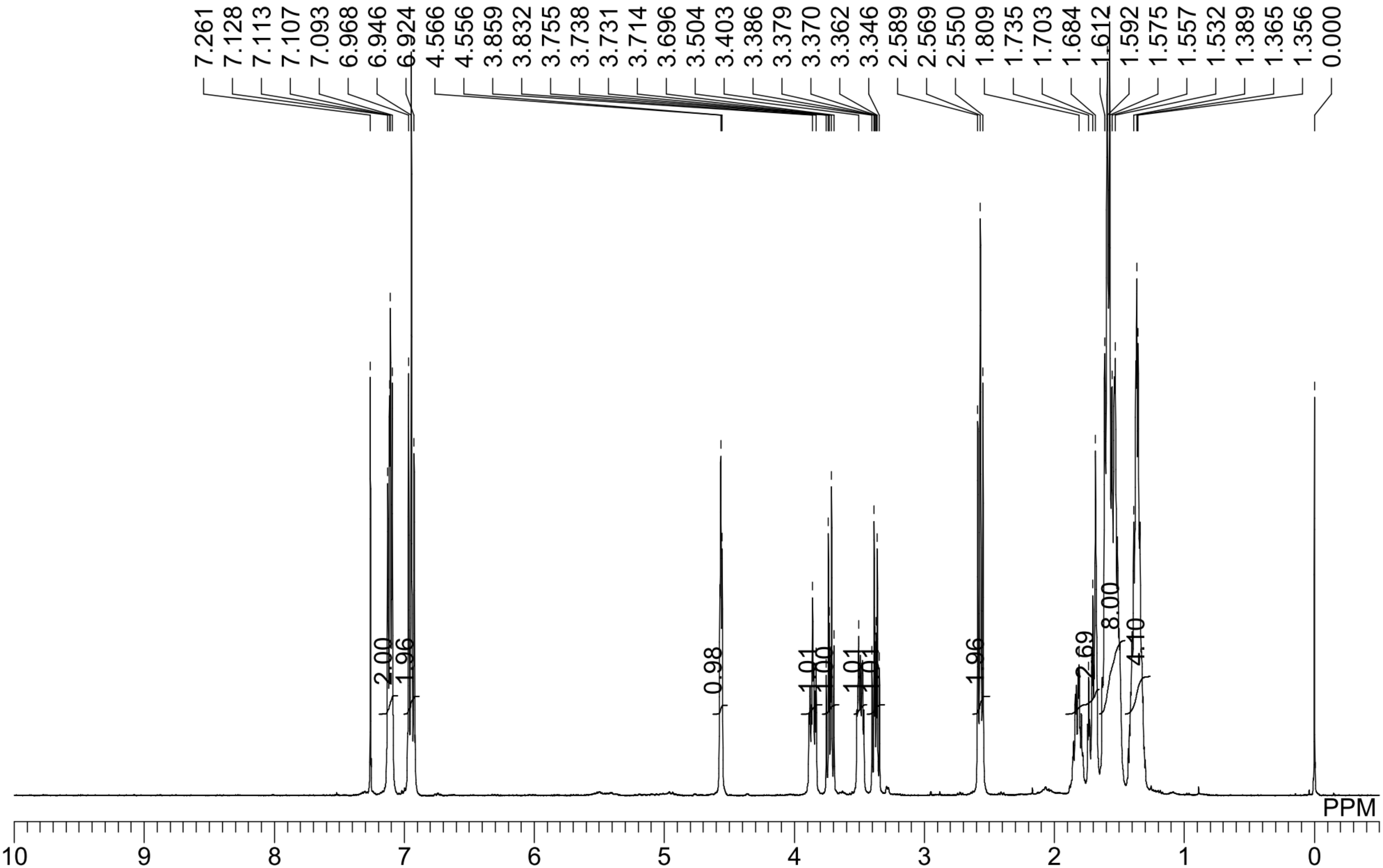
7.261
7.128
7.113
7.107
7.093
6.968
6.946
6.924
4.566
4.556
3.859
3.832
3.755
3.738
3.731
3.714
3.696
3.504
3.403
3.386
3.379
3.370
3.362
3.346
2.589
2.569
2.550
1.809
1.735
1.703
1.684
1.612
1.592
1.575
1.557
1.532
1.389
1.365
1.356
0.000
2.00
1.96
0.98
1.01
1.00
1.01
1.01
1.96
2.69
8.00
4.10
PPM
10
9
8
7
6
5
4
3
2
1
0

$^{13}C\{^1H\}$ NMR of **4ba**

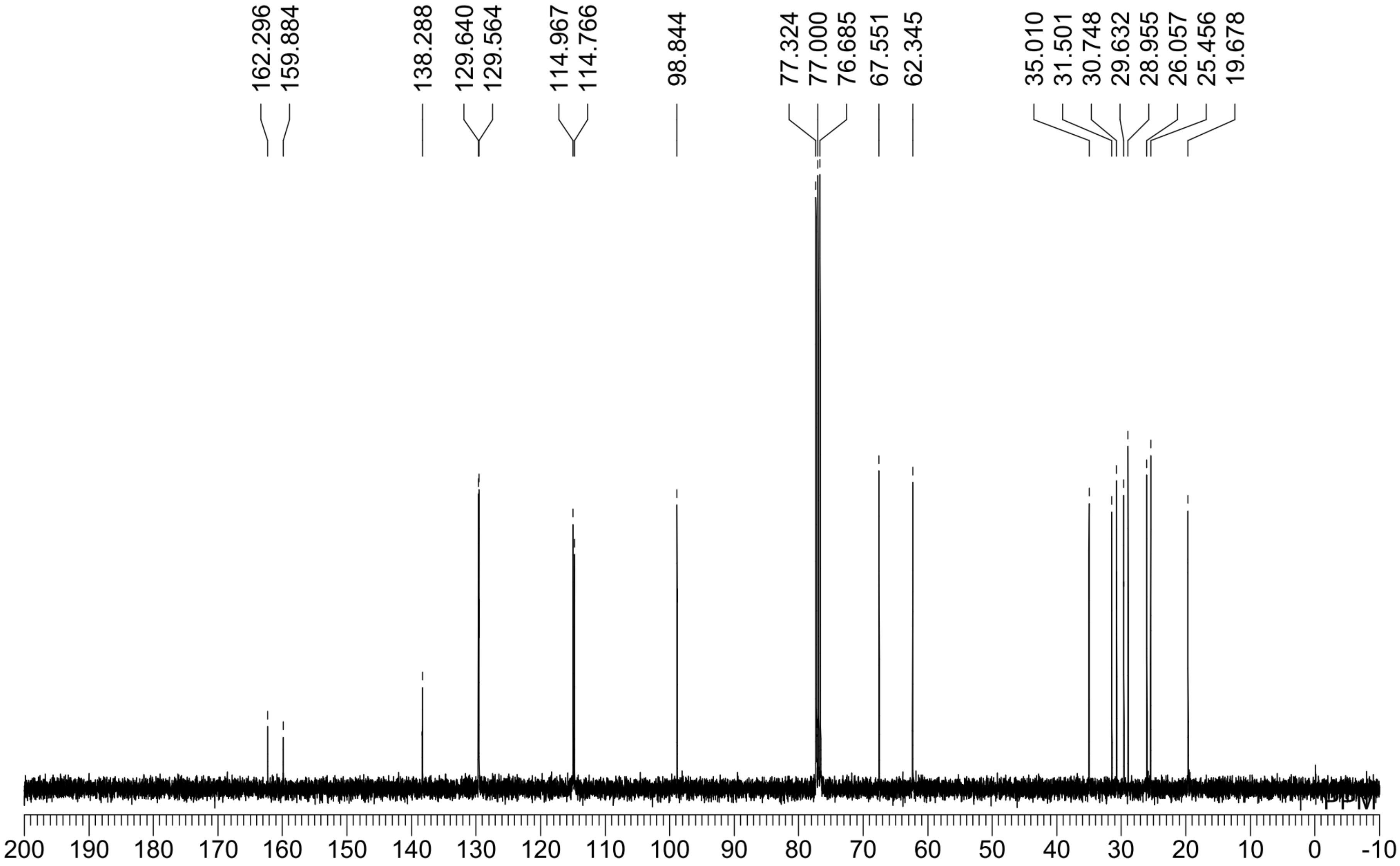

^{1}H NMR of **4ca**

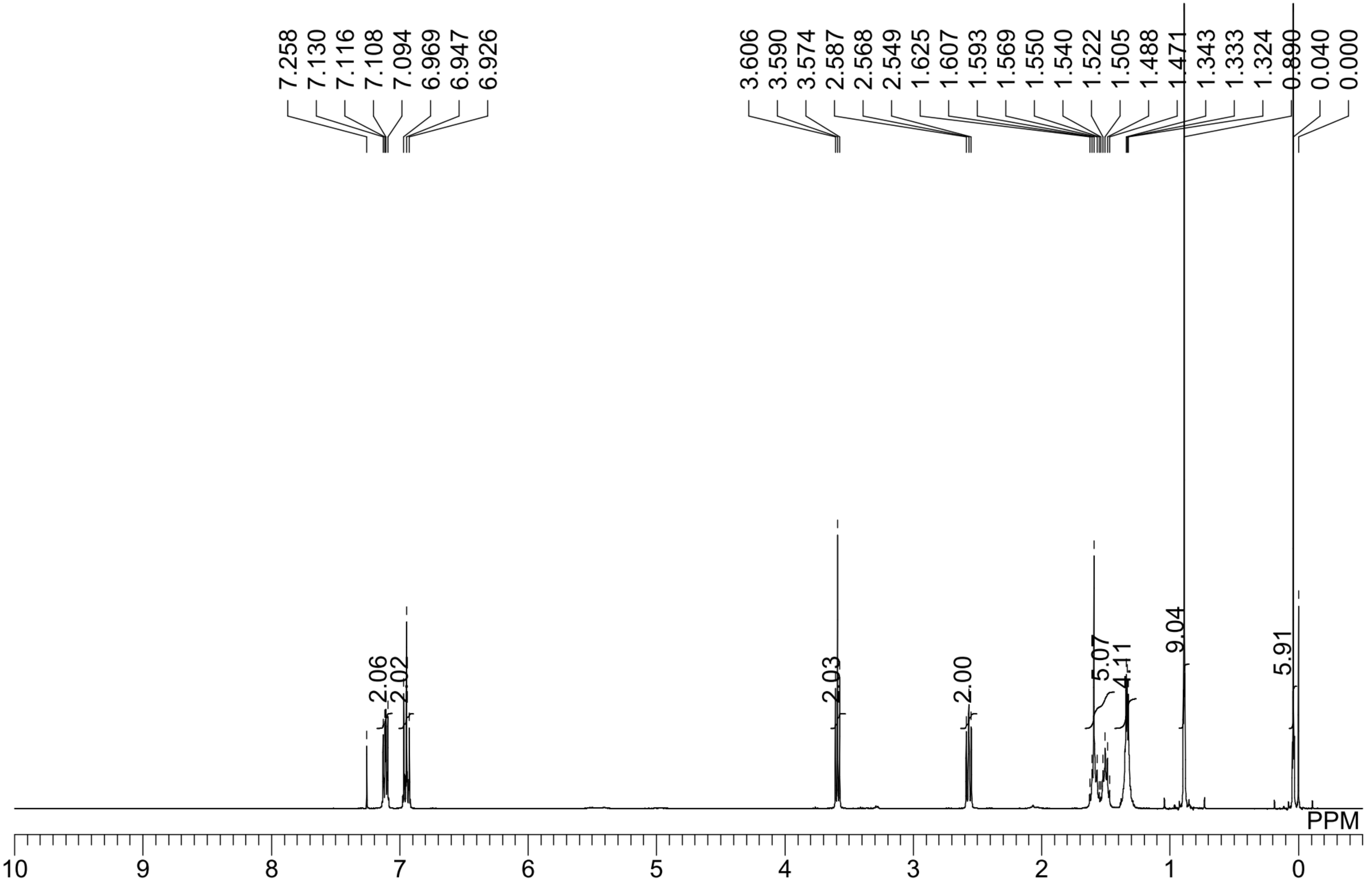
7.258
7.130
7.116
7.108
7.094
6.969
6.947
6.926
3.606
3.590
3.574
2.587
2.568
2.549
1.625
1.607
1.593
1.569
1.550
1.540
1.522
1.505
1.488
1.471
1.343
1.333
1.324
0.890
0.040
0.000
2.06
2.02
2.03
2.00
5.07
4.11
9.04
5.91
PPM
10
9
8
7
6
5
4
3
2
1
0

$^{13}C\{^{1}H\}$ NMR of **4ca**

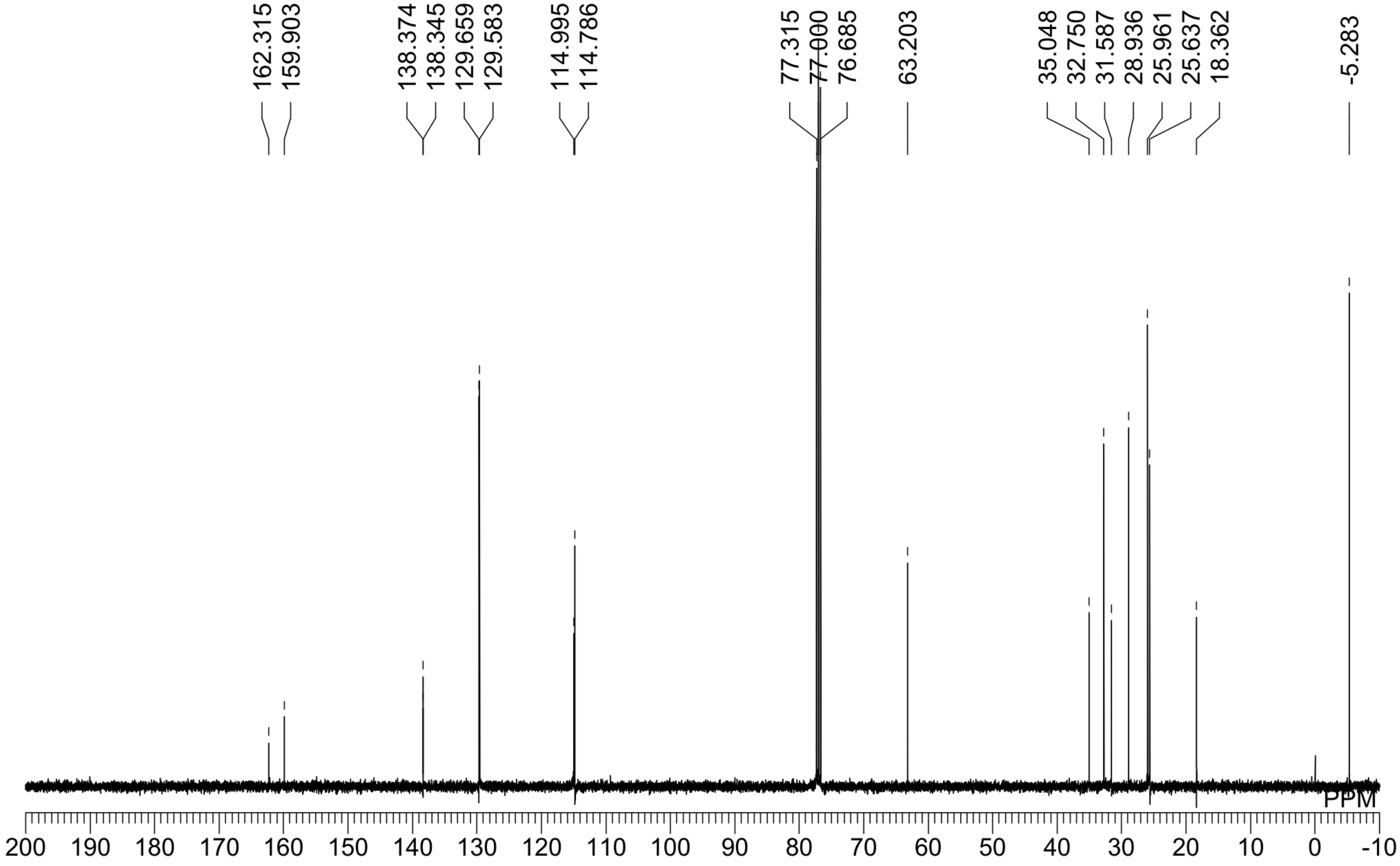

^1H NMR of **4da**

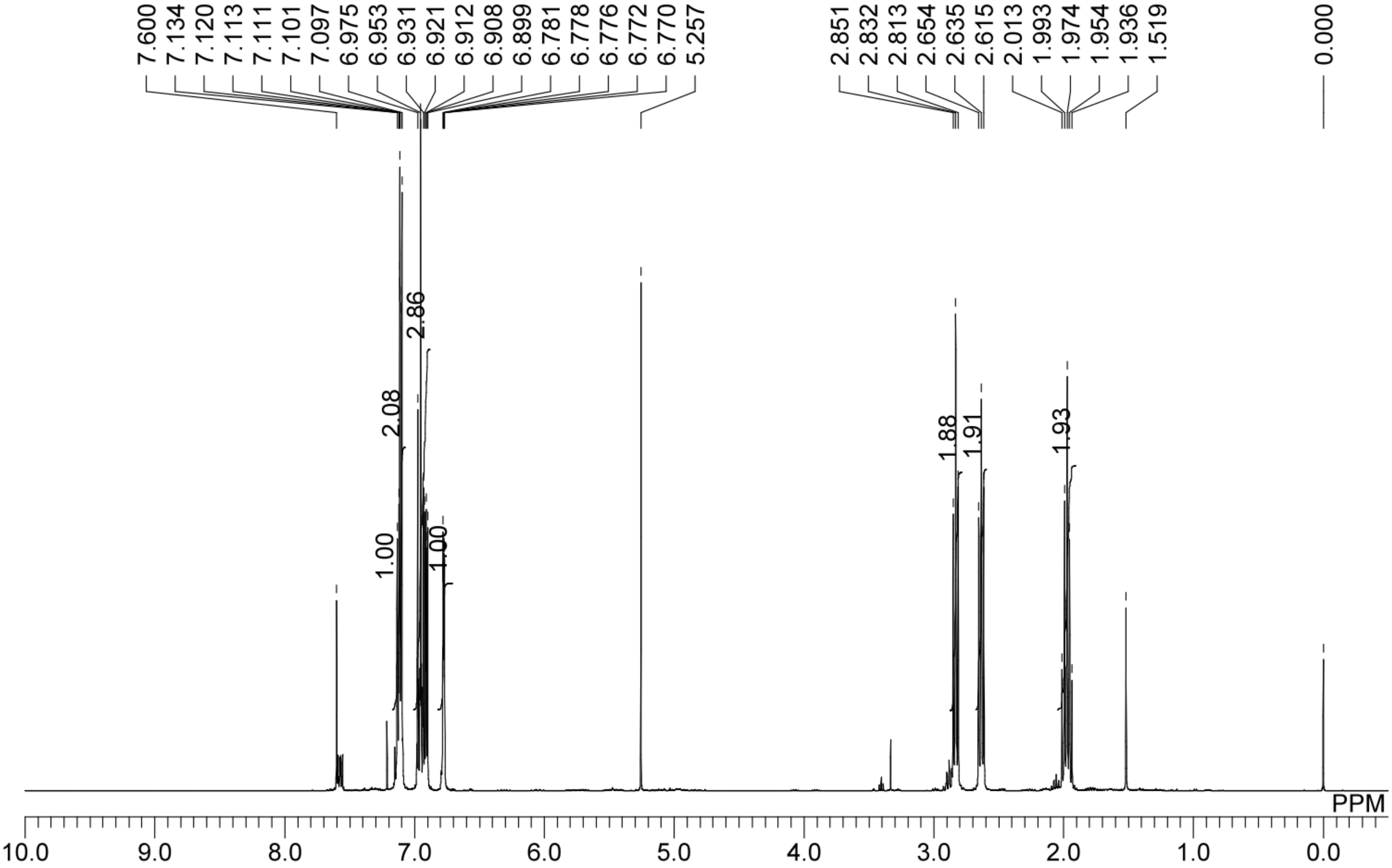
7.600
7.134
7.120
7.113
7.111
7.101
7.097
6.975
6.953
6.931
6.921
6.912
6.908
6.899
6.781
6.778
6.776
6.772
6.770
5.257
2.851
2.832
2.813
2.654
2.635
2.615
2.013
1.993
1.974
1.954
1.936
1.519
0.000
2.86
2.08
1.00
1.00
1.88
1.91
1.93
PPM
10.0
9.0
8.0
7.0
6.0
5.0
4.0
3.0
2.0
1.0
0.0

$^{13}C\{^1H\}$ NMR of **4da**

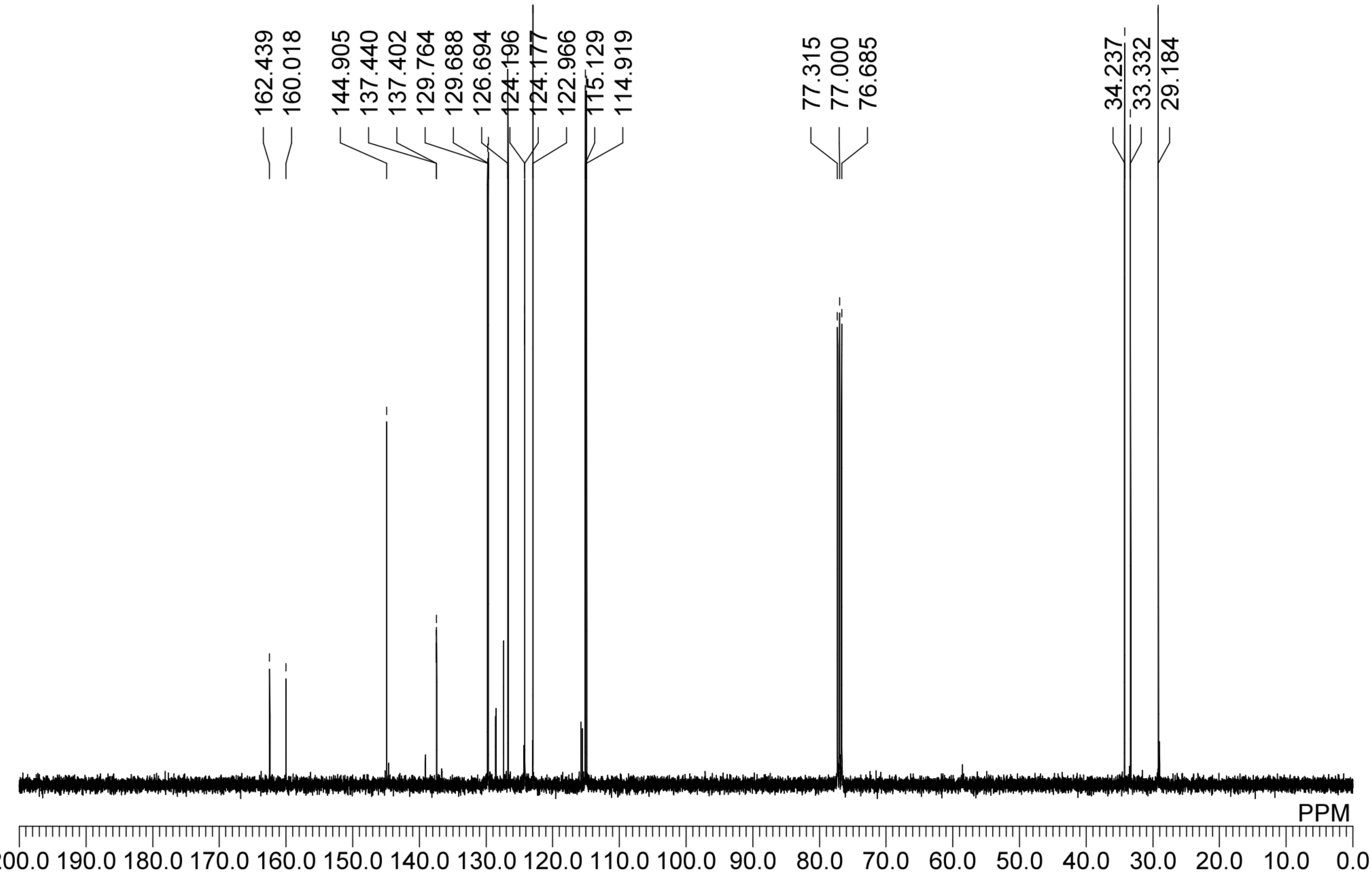

^{1}H NMR of **4ea**

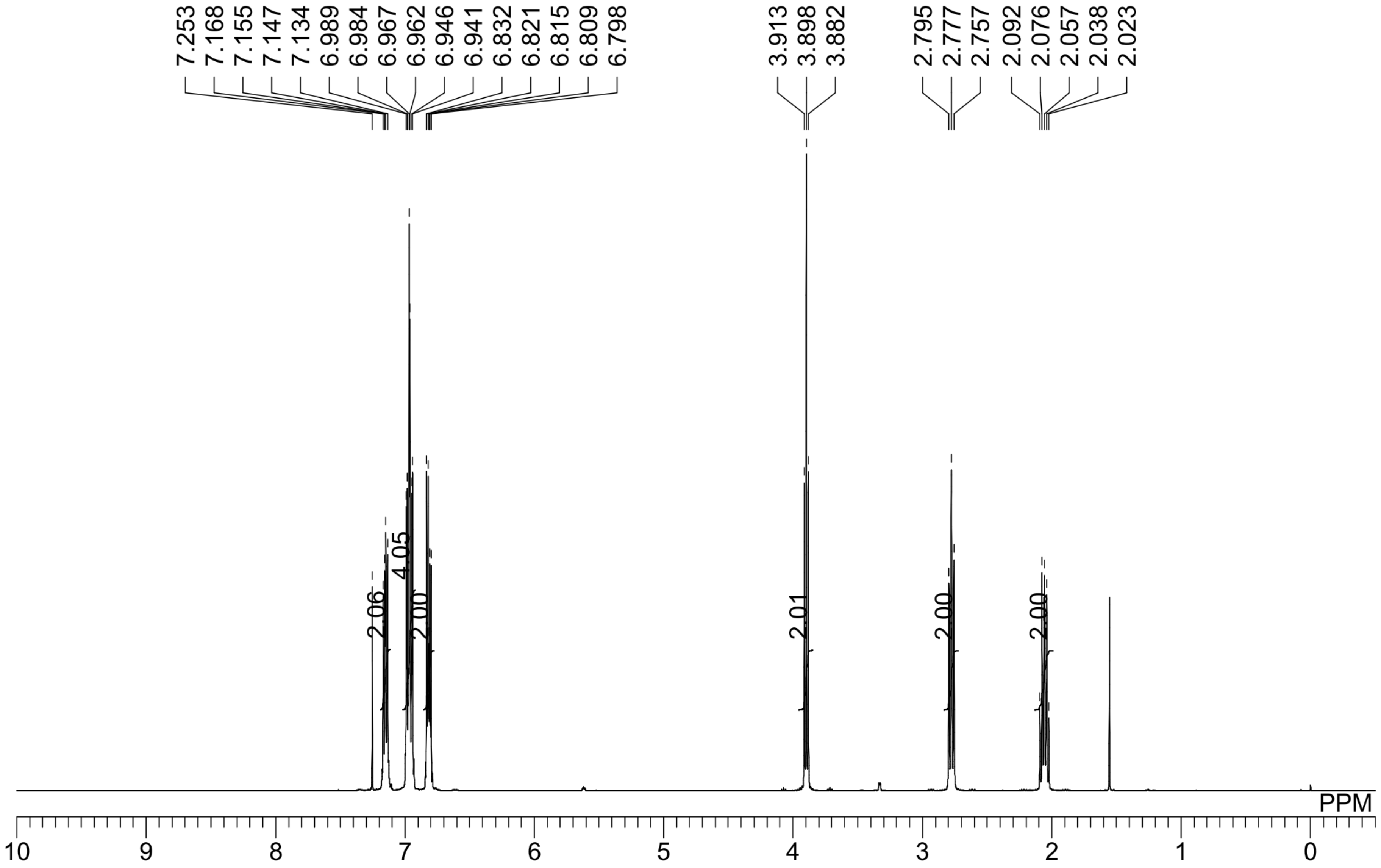
7.253
7.168
7.155
7.147
7.134
6.989
6.984
6.967
6.962
6.946
6.941
6.832
6.821
6.815
6.809
6.798
3.913
3.898
3.882
2.795
2.777
2.757
2.092
2.076
2.057
2.038
2.023
2.06
4.05
2.00
2.01
2.00
2.00
PPM
10
9
8
7
6
5
4
3
2
1
0

$^{13}C\{^{1}H\}$ NMR of **4ea**

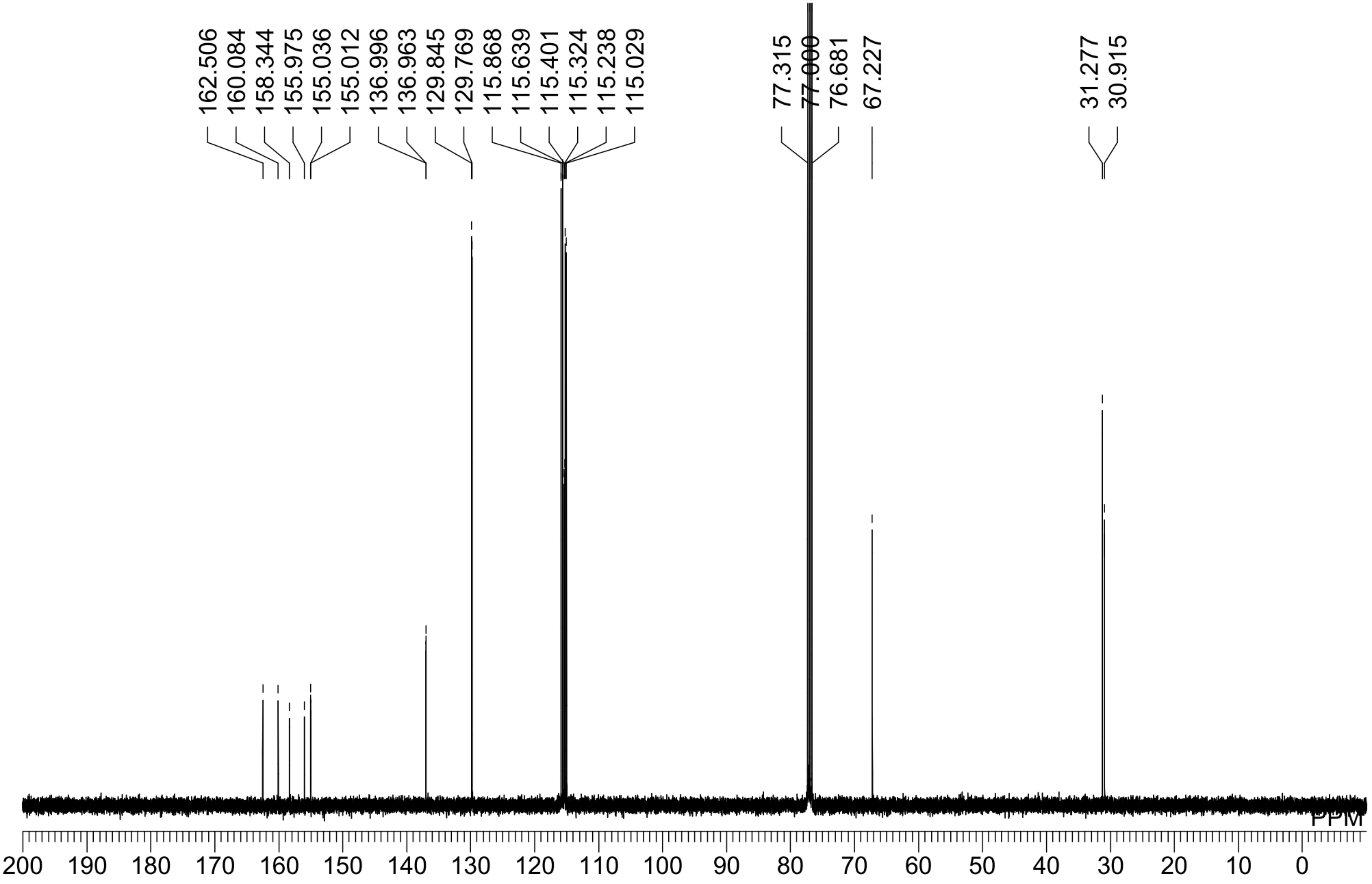

^{1}H NMR of **4fa**

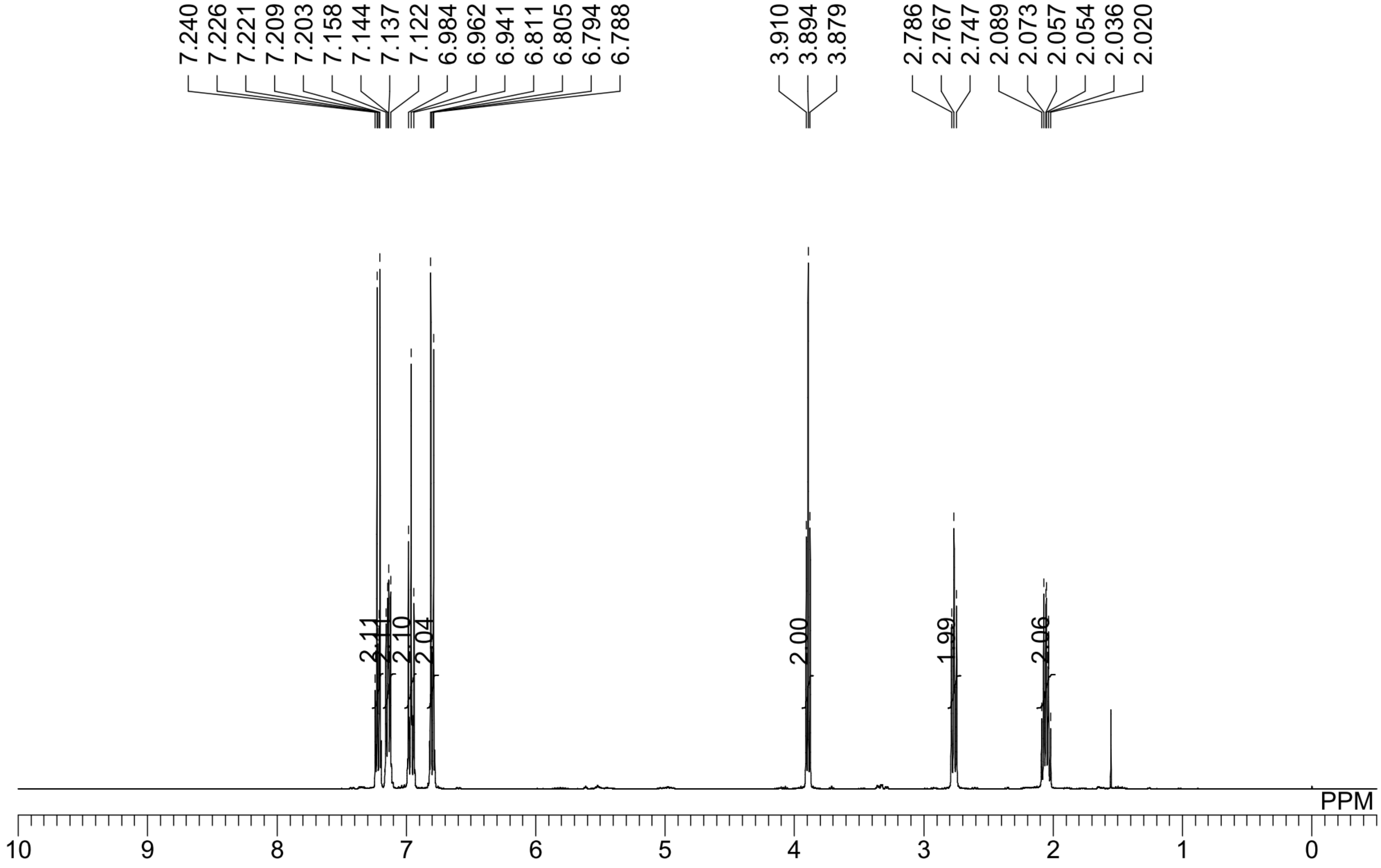
7.240
7.226
7.221
7.209
7.203
7.158
7.144
7.137
7.122
6.984
6.962
6.941
6.811
6.805
6.794
6.788
3.910
3.894
3.879
2.786
2.767
2.747
2.089
2.073
2.057
2.054
2.036
2.020
2.11
2.10
2.04
2.00
1.99
2.06
PPM
10
9
8
7
6
5
4
3
2
1
0

$^{13}C\{^1H\}$ NMR of **4fa**

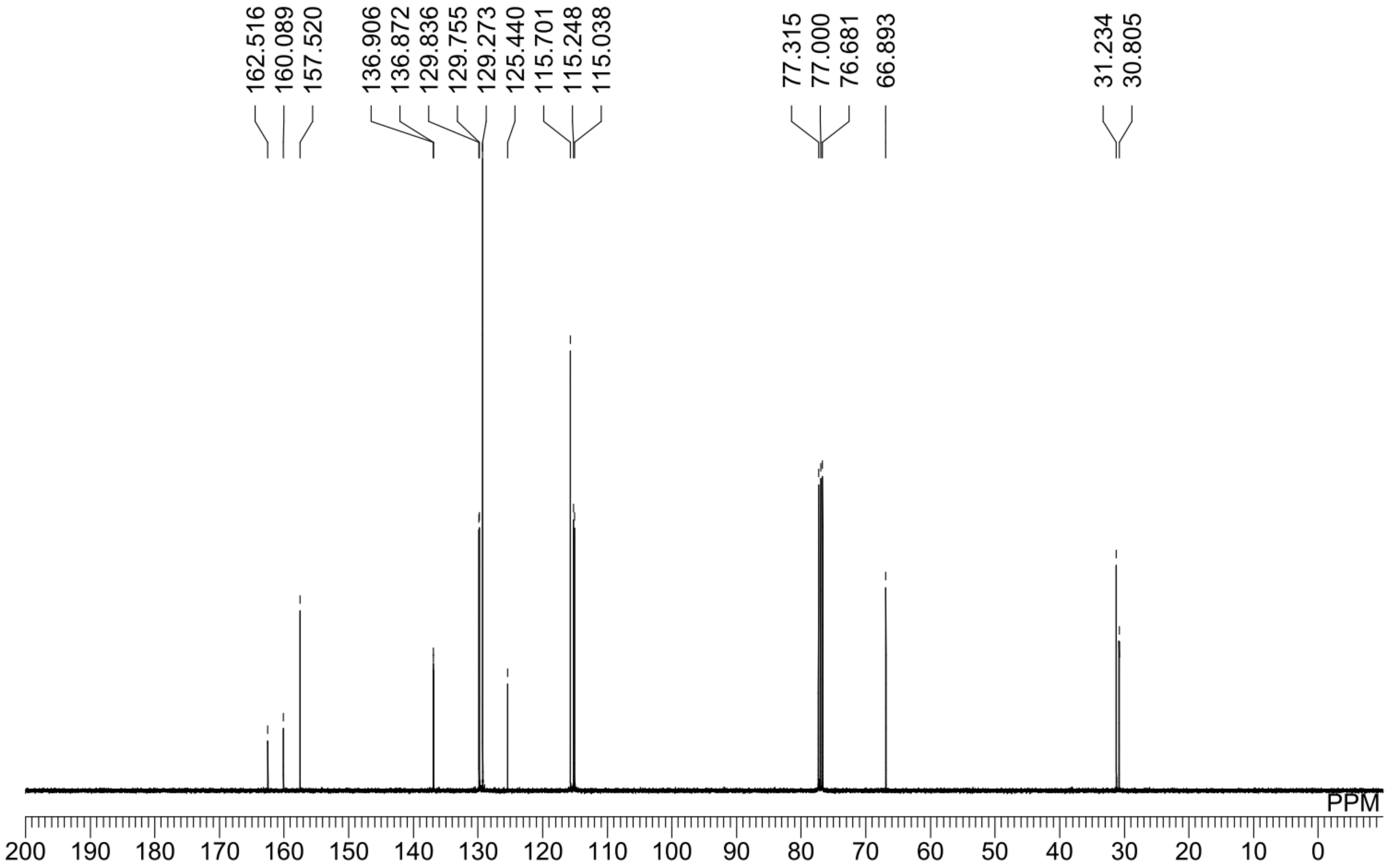

^{1}H NMR of **4ga**

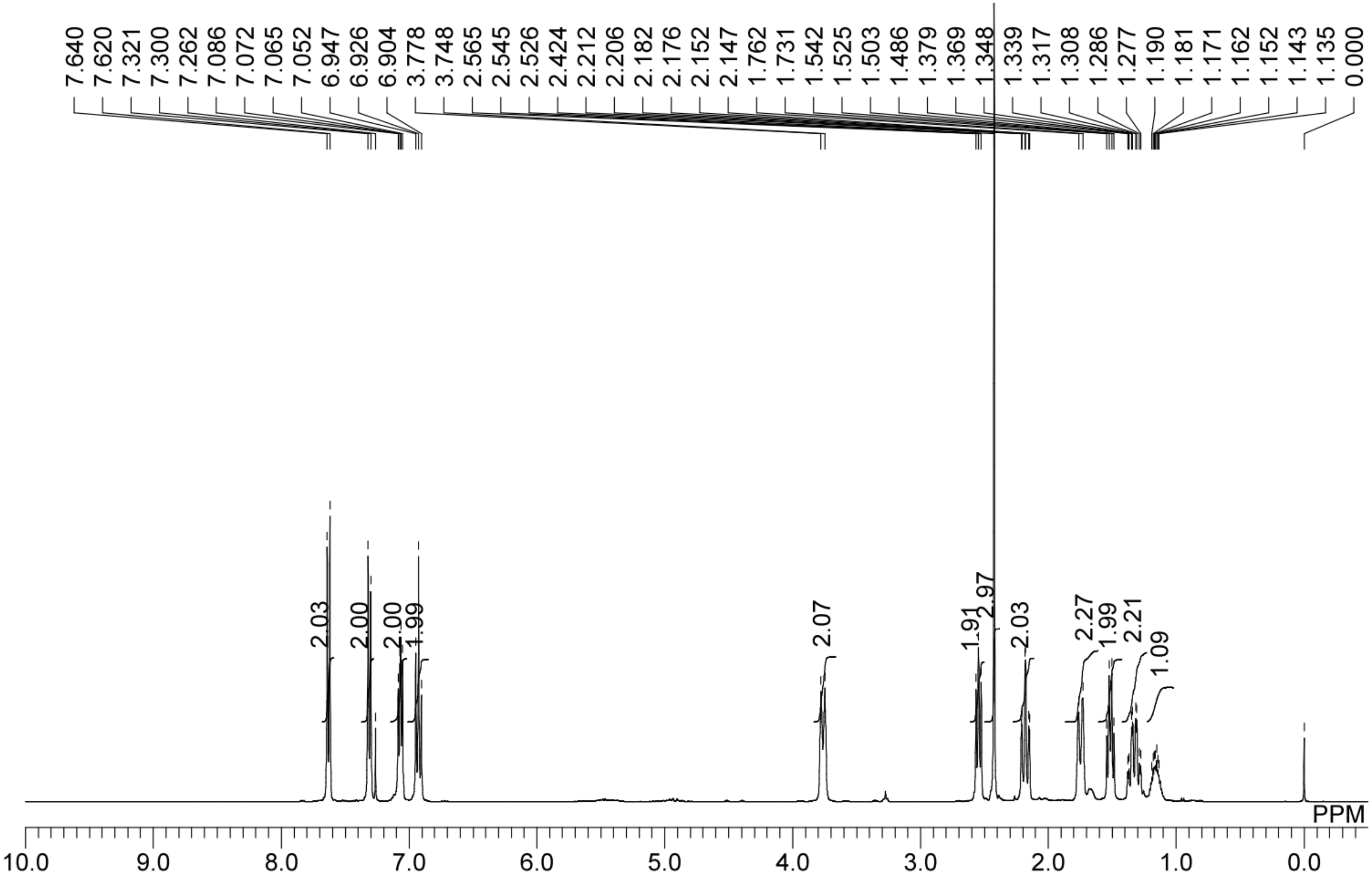
7.640
7.620
7.321
7.300
7.262
7.086
7.072
7.065
7.052
6.947
6.926
6.904
3.778
3.748
2.565
2.545
2.526
2.424
2.212
2.206
2.182
2.176
2.152
2.147
1.762
1.731
1.542
1.525
1.503
1.486
1.379
1.369
1.348
1.339
1.317
1.308
1.286
1.277
1.190
1.181
1.171
1.162
1.152
1.143
1.135
0.000
2.03
2.00
2.00
1.99
2.07
1.91
2.97
2.03
2.27
1.99
2.21
1.09
PPM
10.0
9.0
8.0
7.0
6.0
5.0
4.0
3.0
2.0
1.0
0.0

$^{13}C\{^{1}H\}$ NMR of **4ga**

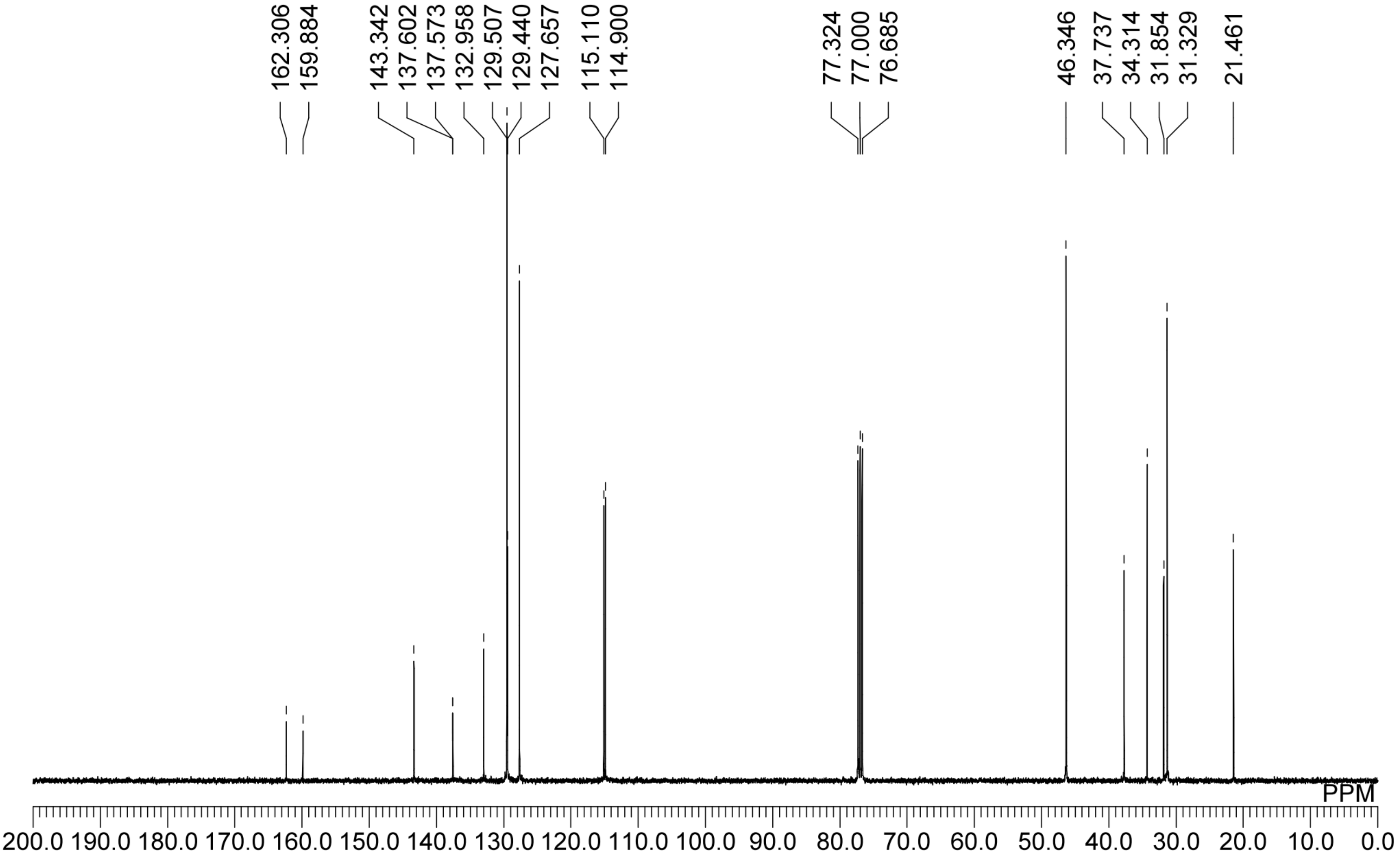

^{1}H NMR of **4ha**

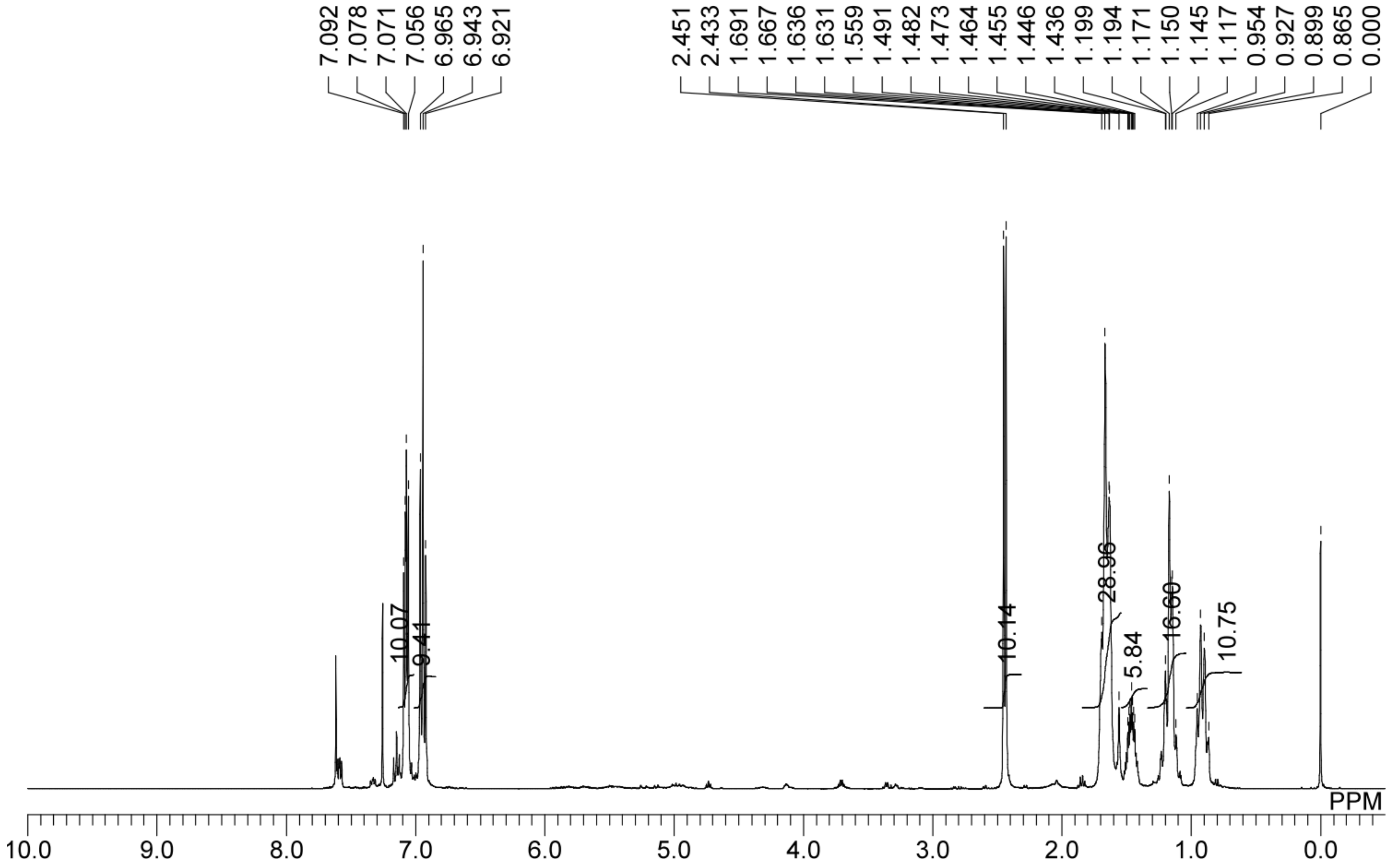
7.092
7.078
7.071
7.056
6.965
6.943
6.921
2.451
2.433
1.691
1.667
1.636
1.631
1.559
1.491
1.482
1.473
1.464
1.455
1.446
1.436
1.199
1.194
1.171
1.150
1.145
1.117
0.954
0.927
0.899
0.865
0.000
10.07
9.41
10.14
28.96
5.84
16.60
10.75
PPM
10.0
9.0
8.0
7.0
6.0
5.0
4.0
3.0
2.0
1.0
0.0

$^{13}C\{^{1}H\}$ NMR of **4ha**

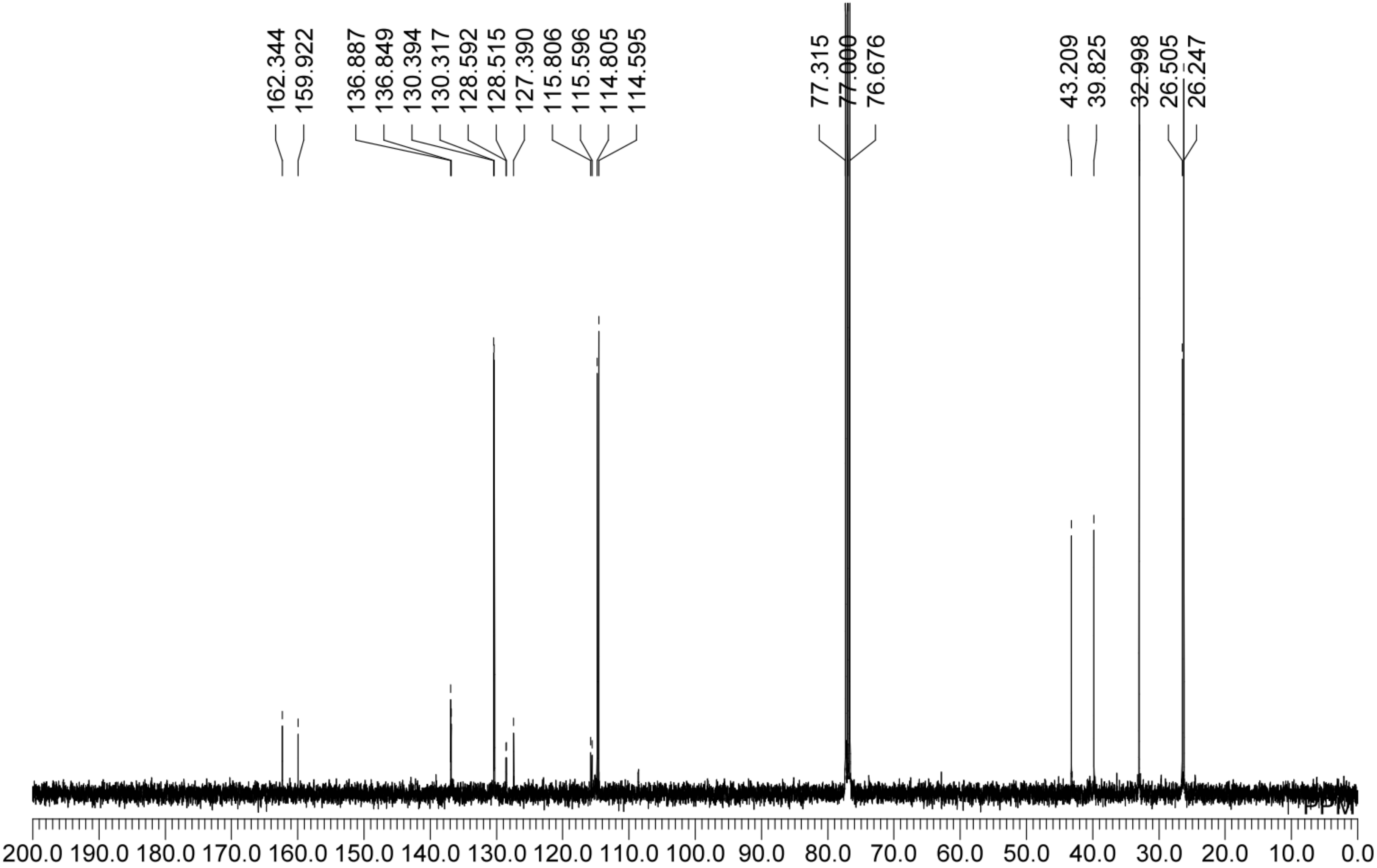

^{1}H NMR of **4gb**

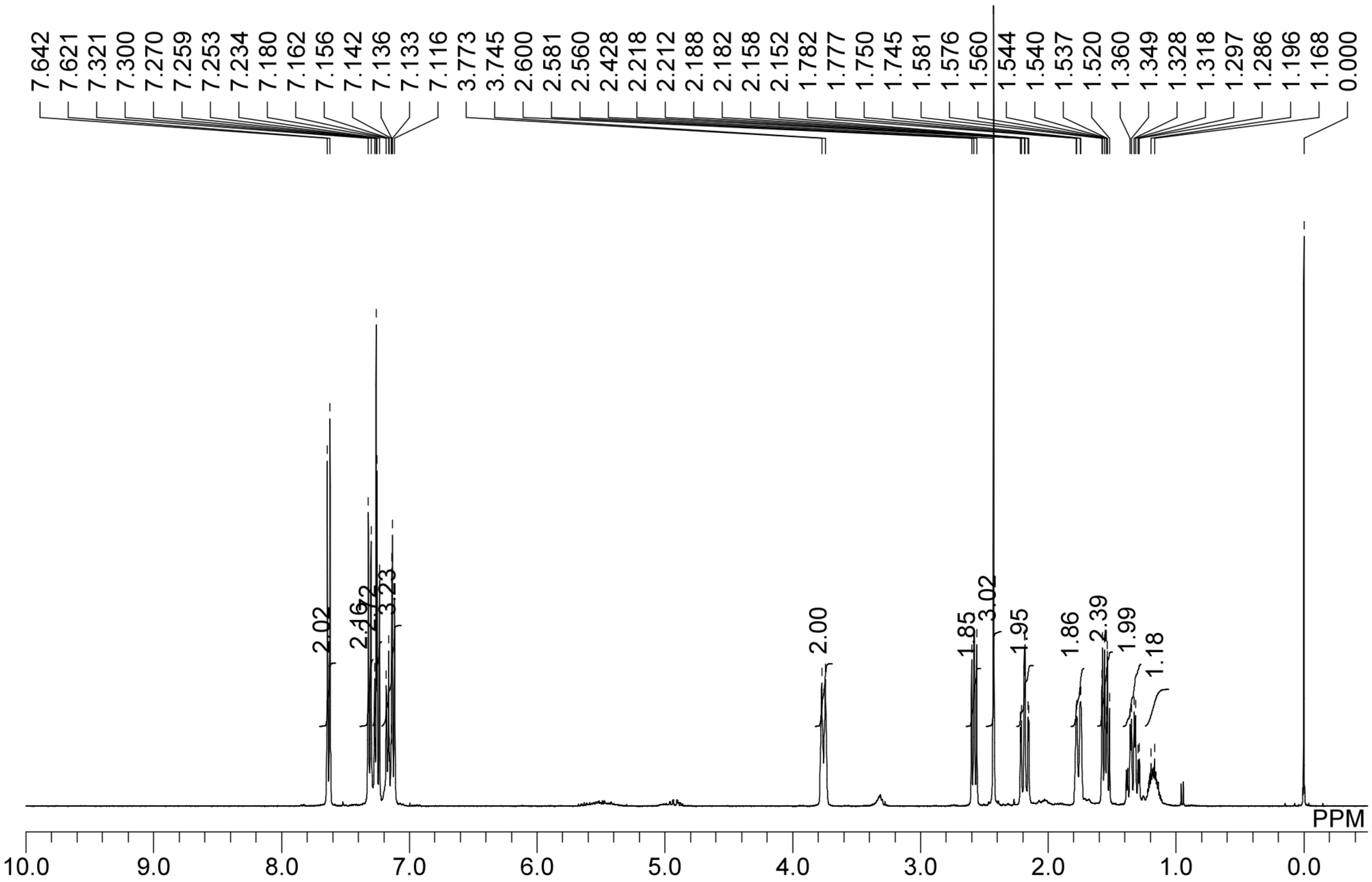

7.642
7.621
7.321
7.300
7.270
7.259
7.253
7.234
7.180
7.162
7.156
7.142
7.136
7.133
7.116
3.773
3.745
2.600
2.581
2.560
2.428
2.218
2.212
2.188
2.182
2.158
2.152
1.782
1.777
1.750
1.745
1.581
1.576
1.560
1.544
1.540
1.537
1.520
1.360
1.349
1.328
1.318
1.297
1.286
1.196
1.168
0.000
2.02
2.16
2.72
2.15
3.23
2.00
1.85
3.02
1.95
1.86
2.39
1.99
1.18
PPM
10.0
9.0
8.0
7.0
6.0
5.0
4.0
3.0
2.0
1.0
0.0

$^{13}C\{^{1}H\}$ NMR of **4gb**

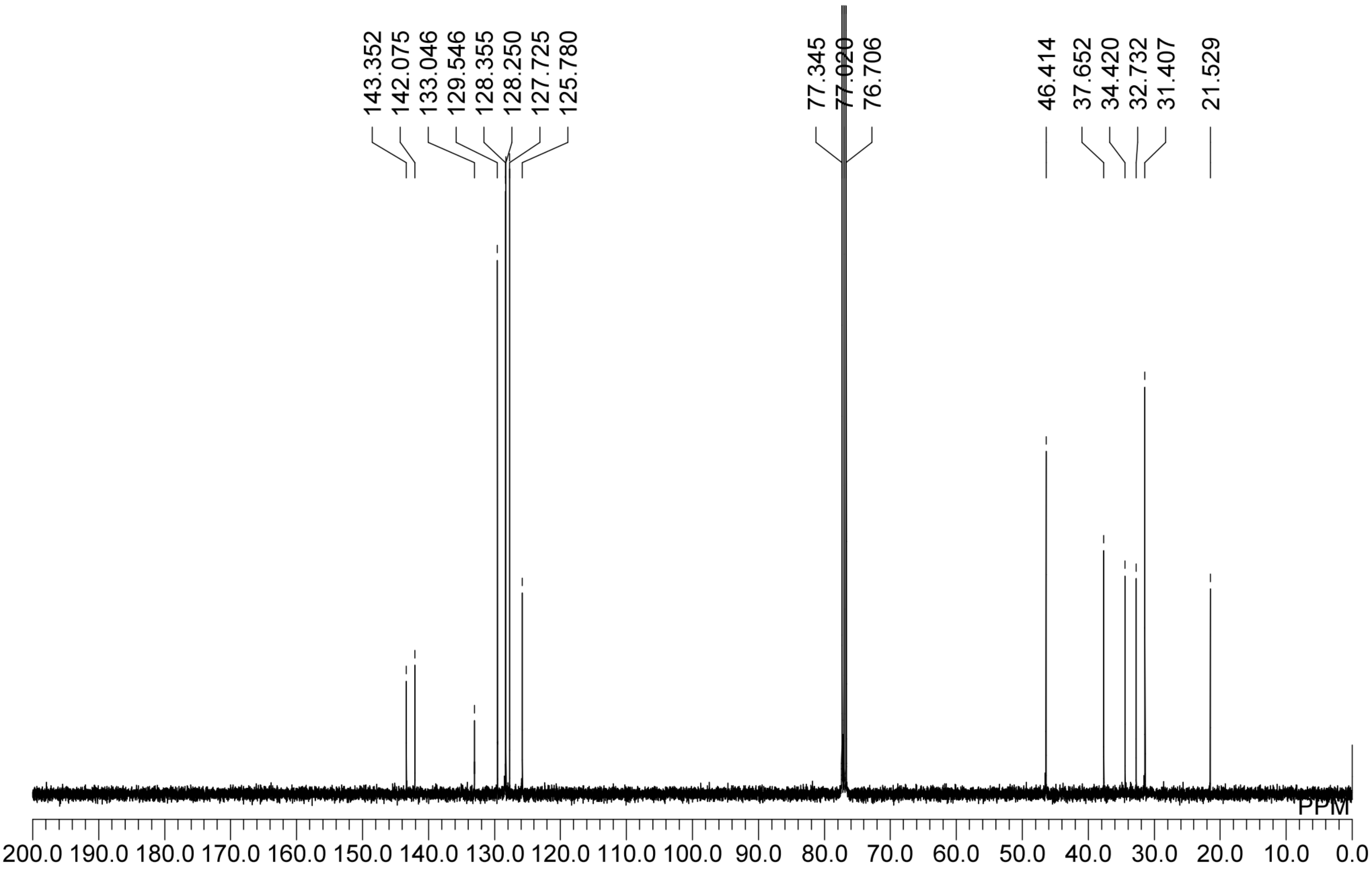

^{1}H NMR of **4gc**

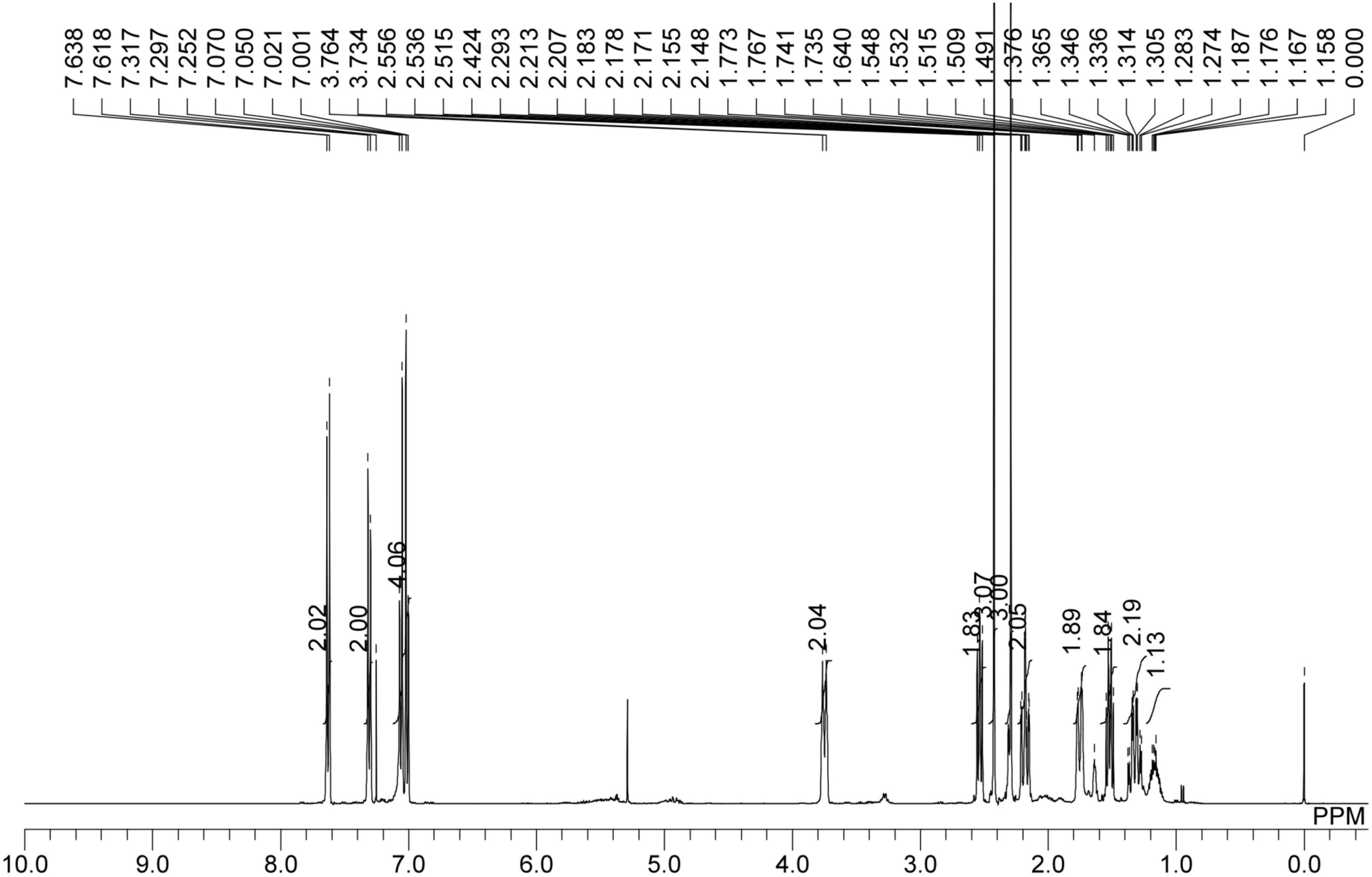
7.638
7.618
7.317
7.297
7.252
7.070
7.050
7.021
7.001
3.764
3.734
2.556
2.536
2.515
2.424
2.293
2.213
2.207
2.183
2.178
2.171
2.155
2.148
1.773
1.767
1.741
1.735
1.640
1.548
1.532
1.515
1.509
1.491
1.376
1.365
1.346
1.336
1.314
1.305
1.283
1.274
1.187
1.176
1.167
1.158
0.000
2.02
2.00
4.06
2.04
1.83
3.07
3.00
2.05
1.89
1.84
2.19
1.13
PPM
10.0
9.0
8.0
7.0
6.0
5.0
4.0
3.0
2.0
1.0
0.0

$^{13}C\{^{1}H\}$ NMR of **4gc**

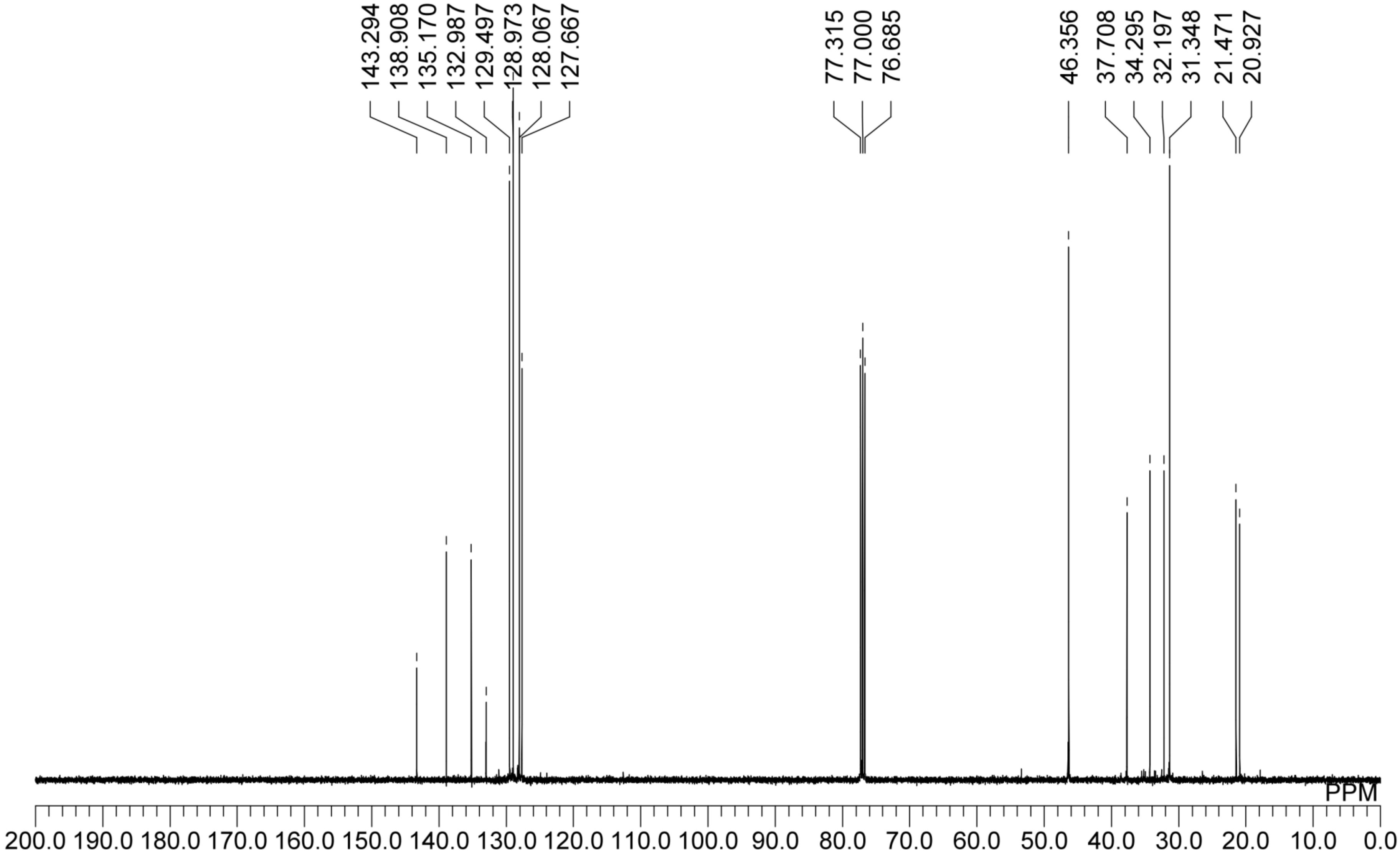

^{1}H NMR of **4gg**

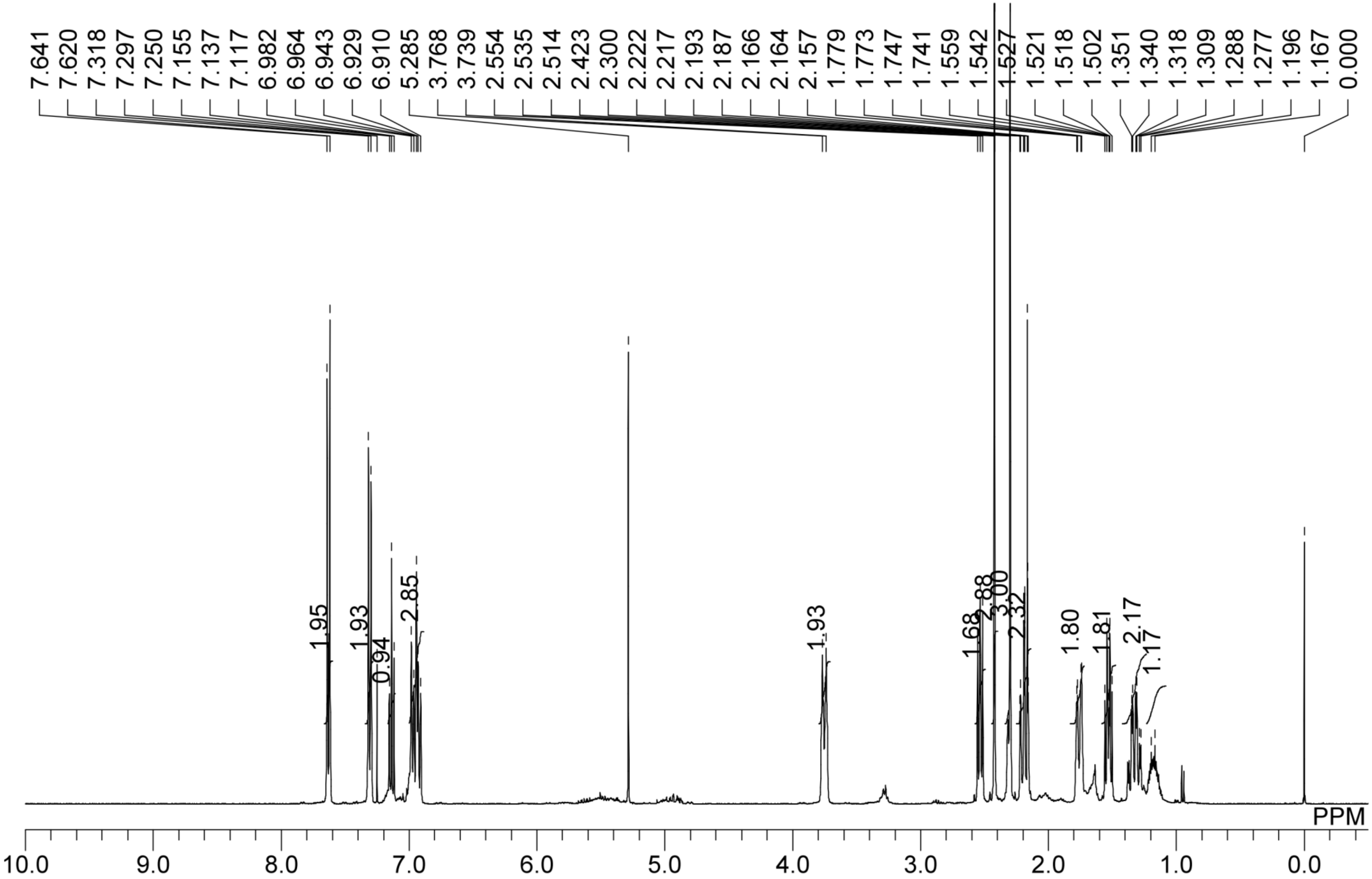
7.641
7.620
7.318
7.297
7.250
7.155
7.137
7.117
6.982
6.964
6.943
6.929
6.910
5.285
3.768
3.739
2.554
2.535
2.514
2.423
2.300
2.222
2.217
2.193
2.187
2.166
2.164
2.157
1.779
1.773
1.747
1.741
1.559
1.542
1.527
1.521
1.518
1.502
1.351
1.340
1.318
1.309
1.288
1.277
1.196
1.167
0.000
1.95
1.93
0.94
2.85
1.93
1.68
2.88
3.00
2.32
1.80
1.81
2.17
1.17
PPM
10.0
9.0
8.0
7.0
6.0
5.0
4.0
3.0
2.0
1.0
0.0

$^{13}C\{^{1}H\}$ NMR of **4gg**

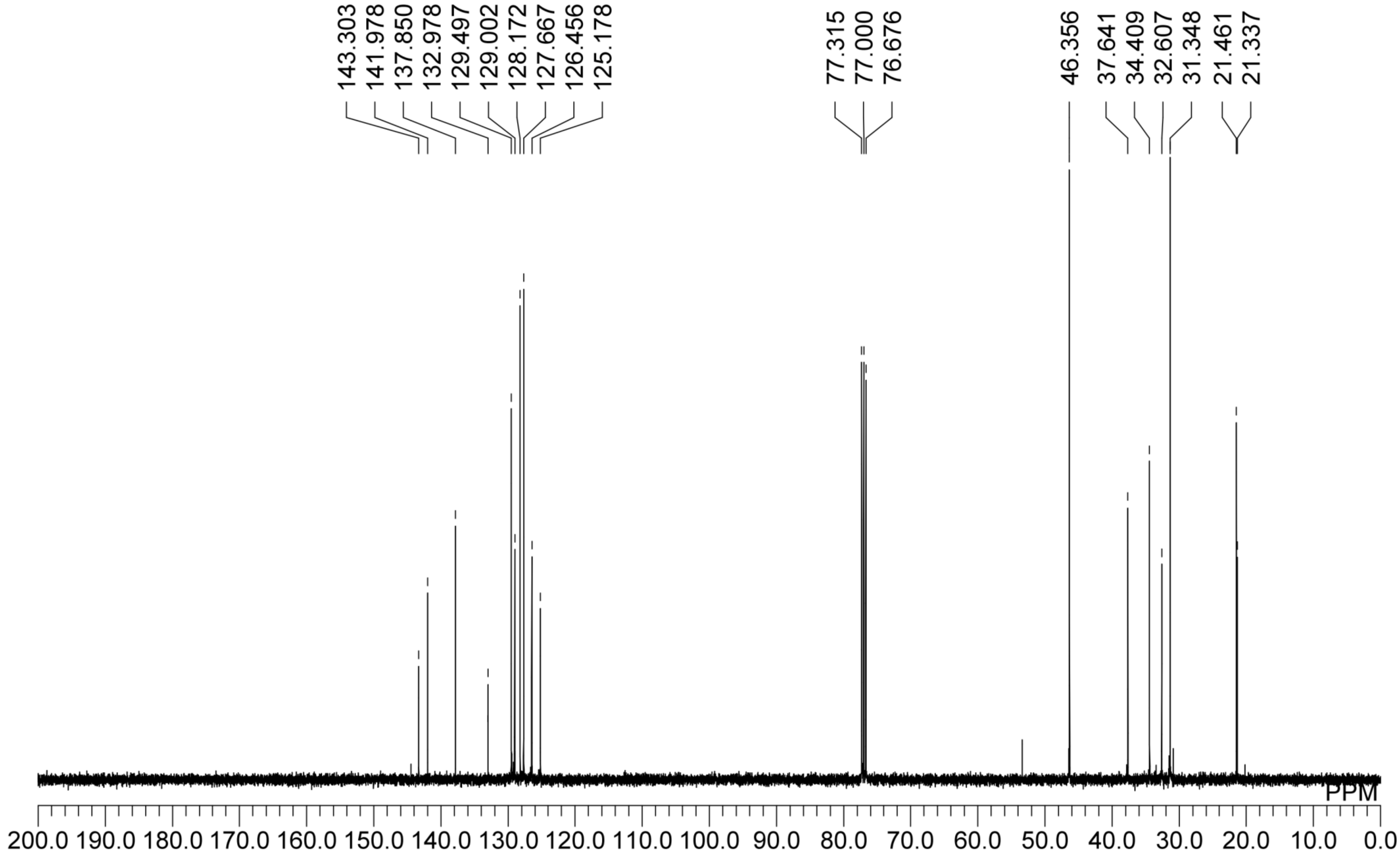

^{1}H NMR of **4gi**

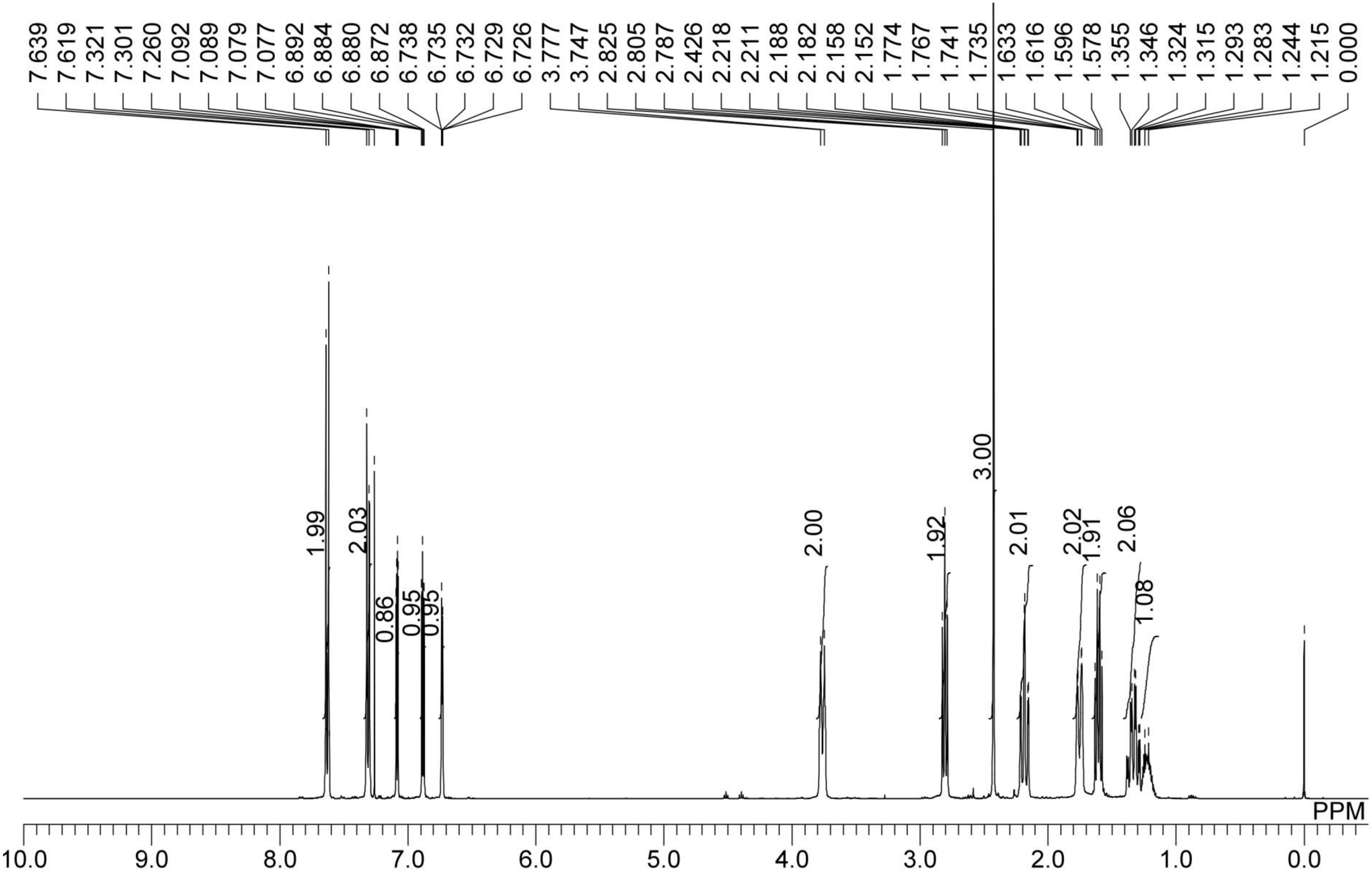
7.639
7.619
7.321
7.301
7.260
7.092
7.089
7.079
7.077
6.892
6.884
6.880
6.872
6.738
6.735
6.732
6.729
6.726
3.777
3.747
2.825
2.805
2.787
2.426
2.218
2.211
2.188
2.182
2.158
2.152
1.774
1.767
1.741
1.735
1.633
1.616
1.596
1.578
1.355
1.346
1.324
1.315
1.293
1.283
1.244
1.215
0.000
1.99
2.03
0.86
0.95
0.95
2.00
1.92
3.00
2.01
2.02
1.91
2.06
1.08
PPM
10.0
9.0
8.0
7.0
6.0
5.0
4.0
3.0
2.0
1.0
0.0

$^{13}C\{^1H\}$ NMR of **4gi**

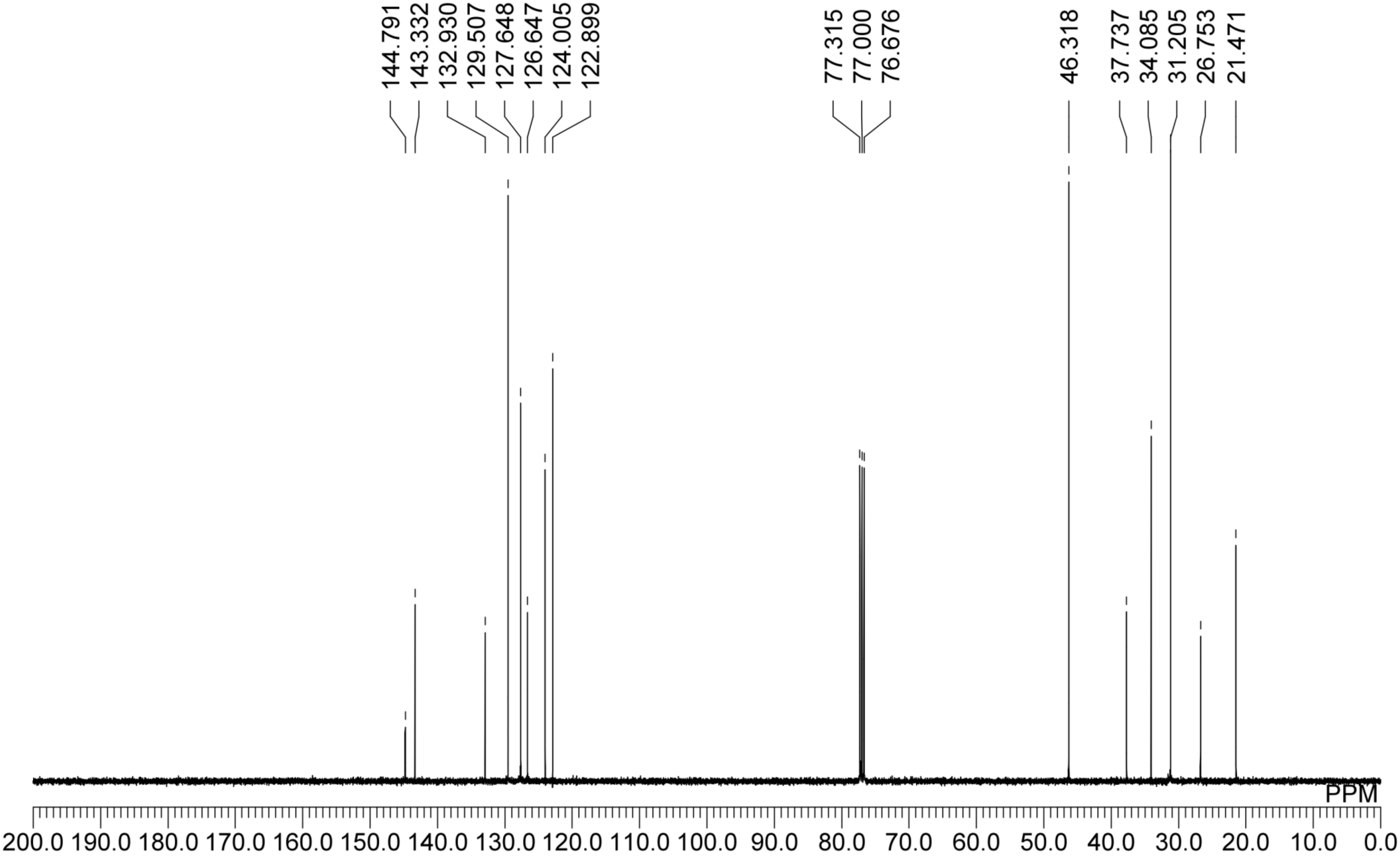

^{1}H NMR of **3aj’** (a mixture of four isomers in a 1:1.3:1.4:1.5 ratio)

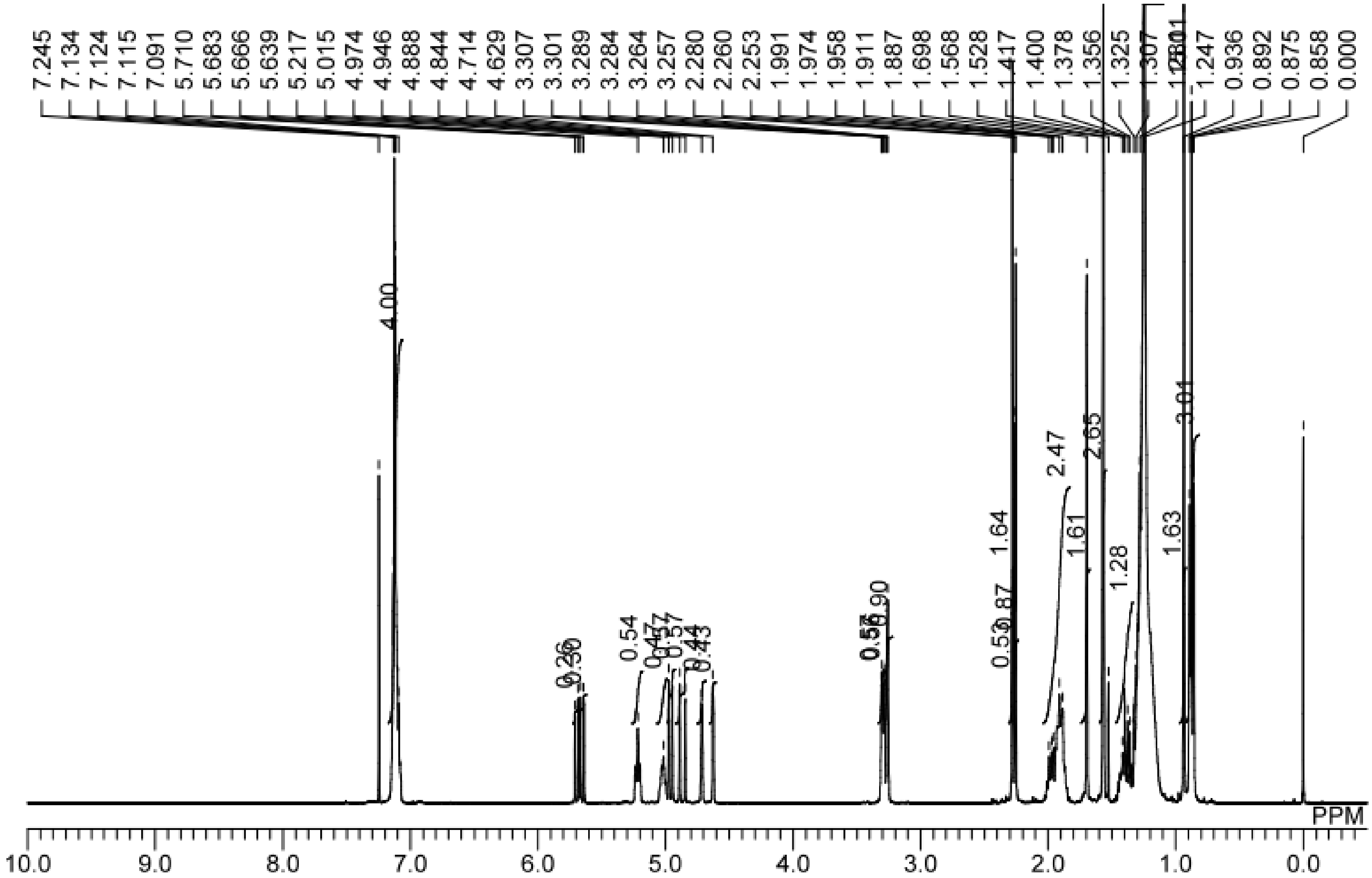

$^{13}C\{^1H\}$ NMR of **3aj'** (a mixture of four isomers in a 1:1.3:1.4:1.5 ratio)

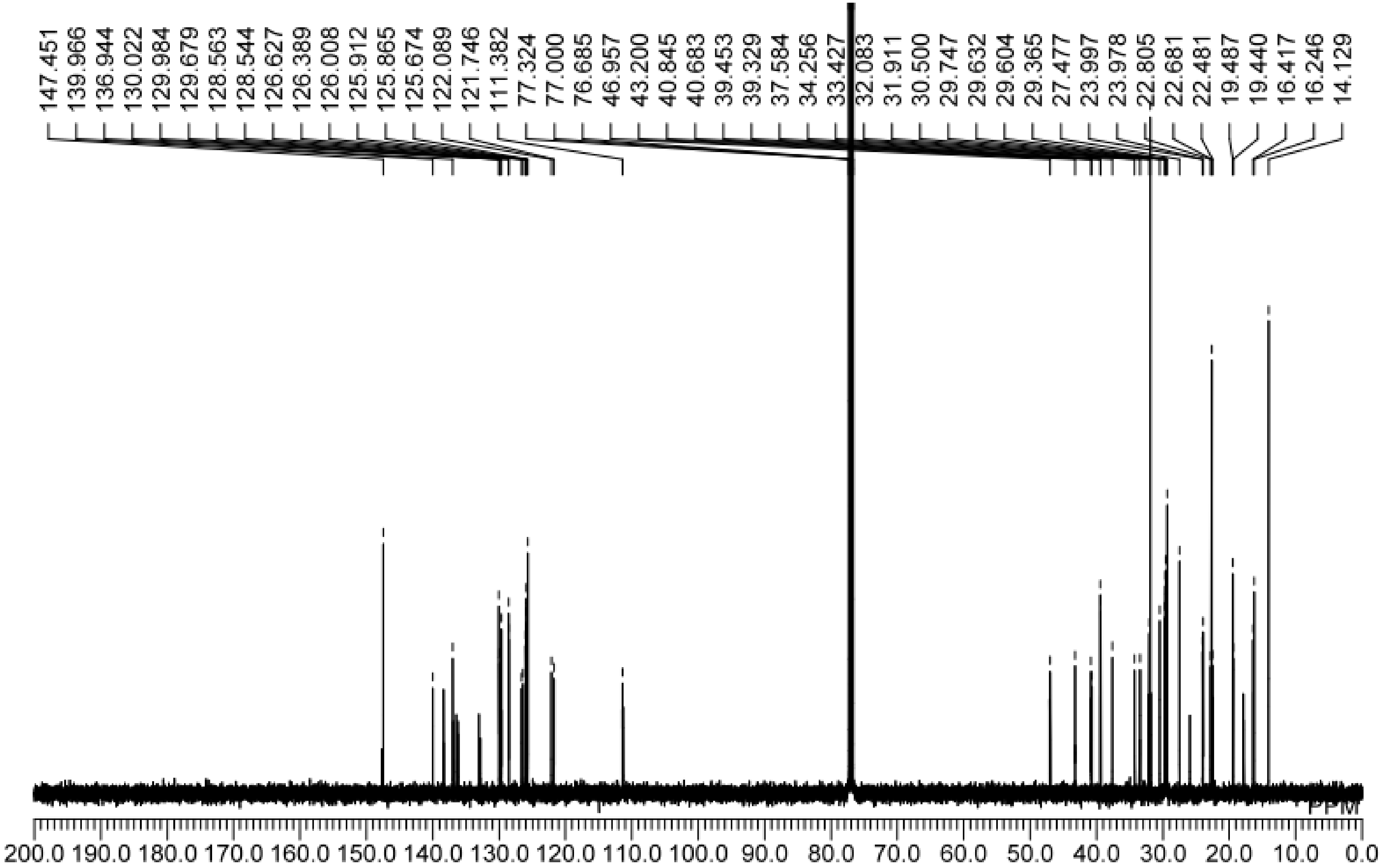

^{1}H NMR of **4na**

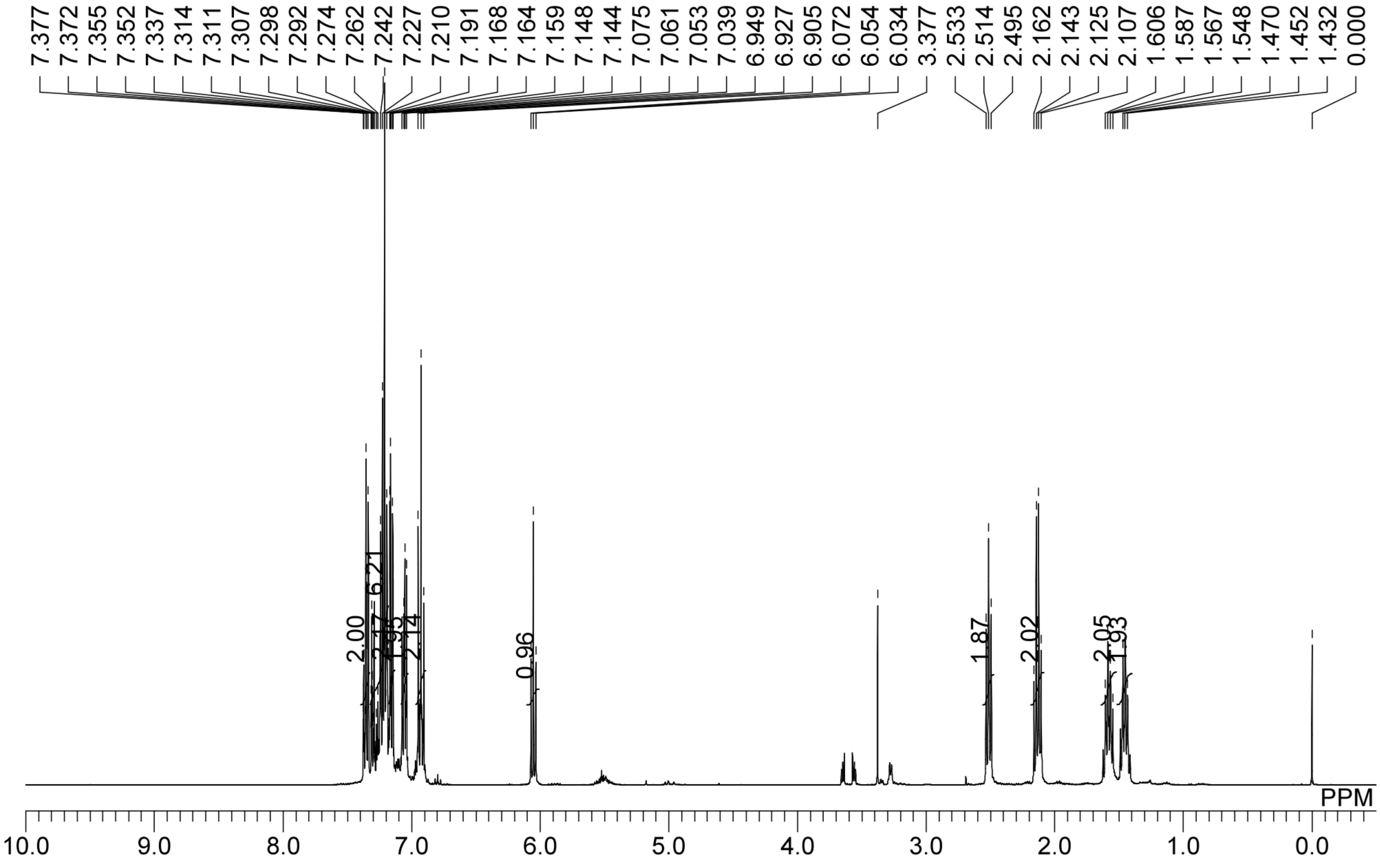

7.377
7.372
7.355
7.352
7.337
7.314
7.311
7.307
7.298
7.292
7.274
7.262
7.242
7.227
7.210
7.191
7.168
7.164
7.159
7.148
7.144
7.075
7.061
7.053
7.039
6.949
6.927
6.905
6.072
6.054
6.034
3.377
2.533
2.514
2.495
2.162
2.143
2.125
2.107
1.606
1.587
1.567
1.548
1.470
1.452
1.432
0.000
2.00
6.21
0.96
1.87
2.02
2.05
1.93
PPM
10.0
9.0
8.0
7.0
6.0
5.0
4.0
3.0
2.0
1.0
0.0

$^{13}C\{^1H\}$ NMR of **4na**

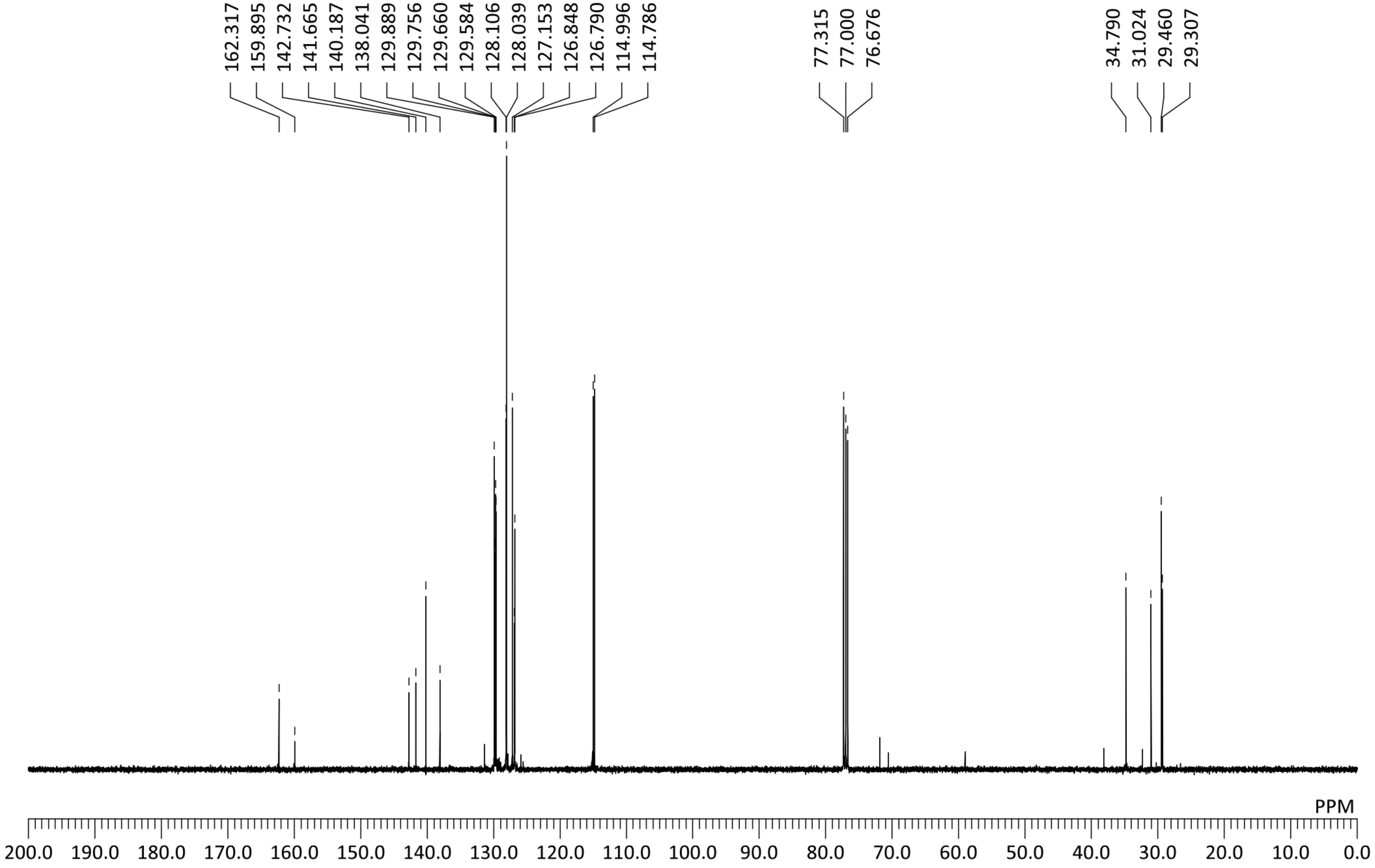

Supplement: Supplementary file 1 [file SC-009-C7SC04675H-s001.pdf]
